# Cu(i) catalysis for selective condensation/bicycloaromatization of two different arylalkynes: direct and general construction of functionalized C–N axial biaryl compounds†

**DOI:** 10.1039/d1sc03865f

**Published:** 2021-12-13

**Authors:** Qian Shang, Haifang Tang, Yongping Liu, MingMing Yin, Lebin Su, Shimin Xie, Lixin Liu, Wen Yang, Yi Chen, Jianyu Dong, Yongbo Zhou, Shuang-Feng Yin

**Affiliations:** Advanced Catalytic Engineering Research Center of the Ministry of Education, College of Chemistry and Chemical Engineering, Hunan University Changsha 410082 China zhouyb@hnu.edu.cn; Department of Educational Science, Hunan First Normal University Changsha 410205 China djyustc@hotmail.com; School of Medicine, Hunan University of Chinese Medicine Changsha 410208 China

## Abstract

Selective condensation/bicycloaromatization of two different arylalkynes is firstly developed under ligand-free copper(i)-catalysis, which allows the direct synthesis of C–N axial biaryl compounds in high yields with excellent selectivity and functional group tolerance. Due to the critical effects of Cu(i) catalyst and HFIP, many easily occurring undesired reactions are suppressed, and the coupled five–six aromatic rings are constructed *via* the selective formation of two C(sp^2^)–N(sp^2^) bonds and four C(sp^2^)–C(sp^2^) bonds. The achievement of moderate enantioselectivity verifies its potential for the simplest asymmetric synthesis of atropoisomeric biaryls. Western blotting demonstrated that the newly developed compounds are promising targets in biology and pharmaceuticals. This unique reaction can construct structurally diverse C–N axial biaryl compounds that have never been reported by other methods, and might be extended to various applications in materials, chemistry, biology, and pharmaceuticals.

## Abstract 2

Selective condensation/bicycloaromatization of two different arylalkynes is firstly developed under ligand-free copper(i)-catalysis, which allows the direct synthesis of C–N axial biaryl compounds in high yields with excellent selectivity and functional group tolerance.

## Graphical abstract

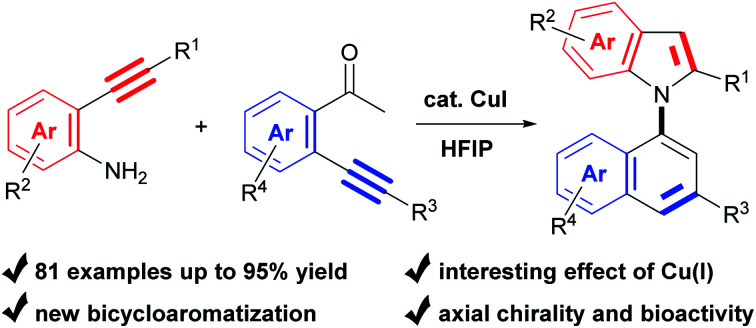

## Introduction

Biaryl compounds that contain an axis and two aryl rings are common chemicals. These structural scaffolds, such as binaphthyls, biindoles, and naphthylindoles, show diverse physical, chemical, and biological properties,^1–8^ thus, they are prevalent in natural products,^2^ pharmaceuticals,^3^ agrochemicals,^4^ functional materials,^5^ and dyestuffs,^6^ as well as ligands^7^ and synthetic reagents.^8^ In fact, a search in SCI Finder indicates that there are more than 10 000 references with regard to this kind of compound.

**Scheme 1:**
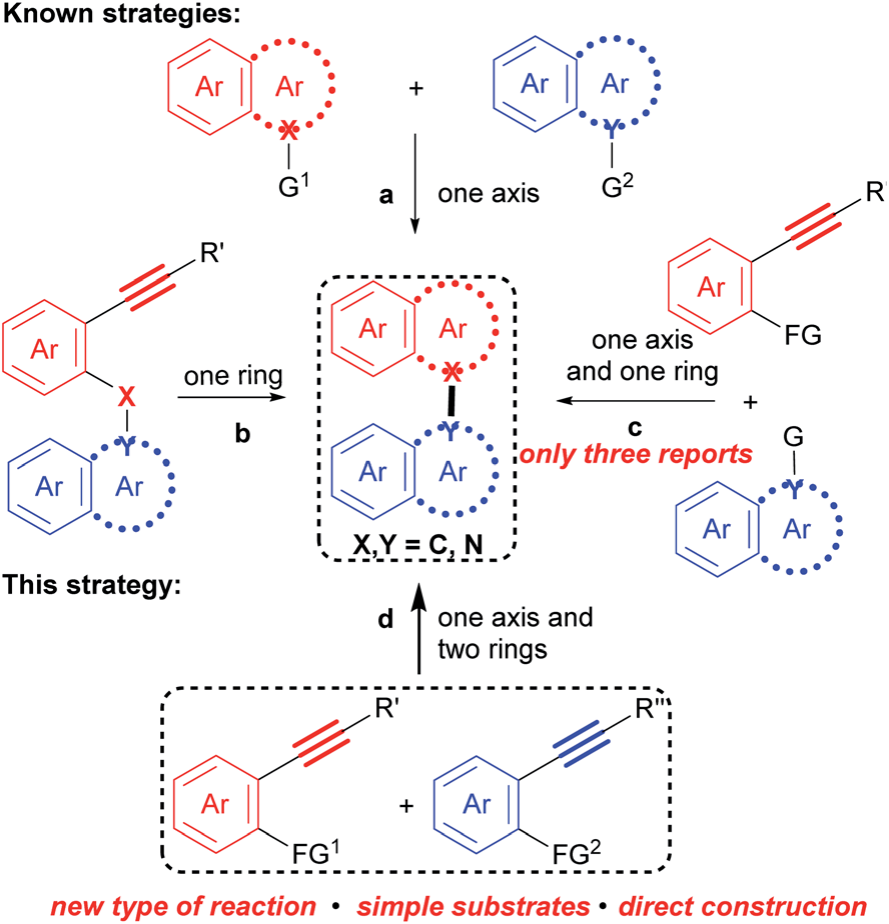
Strategies to construct biaryl compounds.

Correspondingly, numerous methods for their preparation have been developed in recent decades.^9^ However, these methods are generally based on the cross-coupling reaction between two polyaryls (Scheme 1a), such as two naphthalenes,^10^ two indoles^11^ and a naphthalene with an indole,^12^ and the intramolecular cyclization of polyaryl alkynes (Scheme 1b),^13^ in which an axis and an aromatic ring are constructed, respectively. Recently, a more step economical synthesis has been developed by the cross cyclization of prefunctionalized polyaryls with aryl alkynes which constructs both an axis and an aromatic ring (Scheme 1c).^14^ Despite notable advances, challenges and tediousness in the preparation of the prefunctionalized precursors, along with limited substrate scope, have seriously restricted the applications of these methods.

**Fig. 1:**
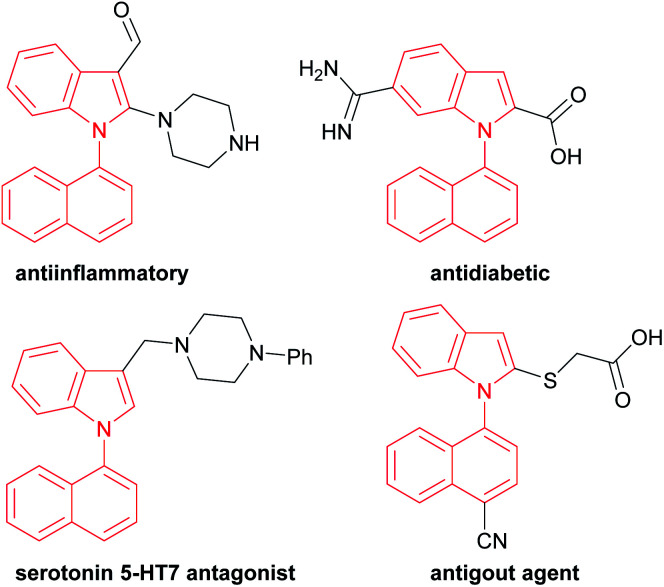
Biologically active 1,1′-naphthylindole derivatives.

For example, 1,1′-naphthylindoles, containing C–N bond-fused five–six rings, are one type of biaryl compounds. It seems that 1,1′-naphthylindoles can be easily synthesized by transition-metal-catalyzed C–N coupling between indoles and naphthalenes. Due to limited substrate availability, there are fewer than 100 entries for 1,1′-naphthylindoles available by a search in SCI Finder. Among the limited reported compounds, 35 examples of this type of compound exhibit a broad spectrum of bioactivities,^15^ such as anti-inflammatory, antidiabetic, receptor antagonist, and antigout activities (Fig. 1), demonstrating that this kind of compound is a promising target in chemistry, biology, and pharmaceuticals.

Therefore, facile, efficient and general synthesis of biaryl compounds directly from easily available substrates are highly desirable, which could not only enrich such valuable compounds, but also provide a window for improving the properties and discovering new functions for this kind of compound, and thus may promote the development of other fields, such as chemistry, biology, pharmaceuticals, and materials science.

*Undoubtedly, considering substrate availability and step economy, condensation/bicycloaromatization between two internal alkynes is the most direct and synthetically powerful approach towards biaryl compounds* (Scheme 1d). However, this strategy is extremely challenging because it requires not only selective formation of a series of C(sp^2^)–X(sp^2^) (X = C and N) bonds to construct an axis and two aromatic rings, but also suppression of many types of easily occurring undesired reactions, such as addition, dimerization and intramolecular cyclization of the two different aryl alkynes.^16^ Indeed, it has not been achieved.

**Scheme 2:**
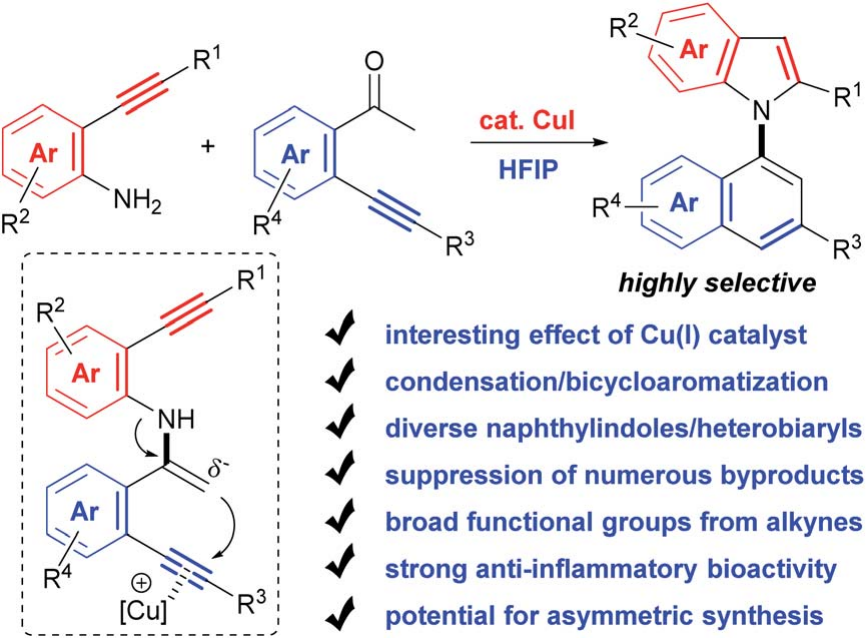
Condensation/bicycloaromatization of *o*-amino and *o*-carbonyl arylalkynes.

Herein, with the continuous interest in the selective transformation of alkynes,^17^ we develop a condensation/bicycloaromatization reaction of easily available *o*-amino and *o*-carbonyl arylalkynes under ligand-free copper(i) catalysis, which successfully suppresses many easily occurring undesired reactions and produces diverse functionalized 1,1′-naphthylindoles in high to excellent yields with excellent selectivity (Scheme 2). In this tandem reaction, the C–N axis is formed by the condensation of the amino and carbonyl groups from two different arylalkynes. Two different aromatic rings, *i.e.*, naphthalene and indole, are exclusively constructed *via* tandem 6-*endo-dig* carbocyclization and 5-*endo-dig* nitrocyclization, respectively, without observation of any other C–N or C–C axial biaryl compounds. In addition to naphthylindoles, this method is also applicable to construct axial heterobiaryl scaffolds such as benzothiophenelindoles, quinolinelindoles, benzofuranindoles, benzothiophenyl-pyrrolopyridine, naphthyl-pyrrolopyridine, and benzofuranyl-pyrrolopyridine, which are not or hardly synthesized by other methods. Moreover, a very broad range of functional groups are readily incorporated into the C–N axial biaryl compounds due to the availability of substrates and very mild reaction conditions. Western blotting has demonstrated that functionalized 1,1′-naphthylindoles have strong anti-inflammatory bioactivity. Thus, this unique condensation/bicycloaromatization represents an ideal strategy for the synthesis of functionalized C–N axial biaryl compounds.

## Results and discussion

### Optimization studies

Although conceptually appealing, the selective execution of the condensation/bicycloaromatization between *o*-amino and *o*-carbonyl arylalkynes faces several challenges (for details, see the ESI, Tables S1–S6†): (1) diverse types of easily occurring reactions for both *o*-amino and *o*-carbonyl arylalkynes. *o*-Amino arylkynes can easily undergo a series of reactions, such as hydration, intramolecular cyclization and di/polymerizations, which produce amino aryl ketones,^18^ indoles,^19^ 2-(2-aminophenyl)quinoline,^20^*etc.* Meanwhile, more types of reactions for *o*-carbonyl arylalkynes, such as hydration, intramolecular cyclizations, aldol condensation, and di/polymerizations, could take place, producing diketones,^21^ triketones,^22^ 1-indanones,^23^ indenones,^24^ 1-naphthols,^23^ chalocones,^25^ 1*H*-isochromene^26^ and chrysene,^27^*etc.* (2) Low reactivity of the condensation between arylamino and arylcarbonyl groups. The relatively weak nucleophilicity of the N-atom of arylamine disfavors its attack at the carbonyl group; thus, other undesired reactions preferentially occur. (3) Competitive reactions of *o*-amino arylalkynes with *o*-carbonyl arylalkynes. Other types of condensation and condensation/cyclizations between the two different alkynes may take place, which reduce the cross selectivity. For example, three types of annulation reactions often occur for the two alkynes, *i.e.*, 5-*endo-dig*,^24^ 5-*exo-dig*,^23^ and 6-*endo-dig* cyclization.^23,26^ Therefore, selectively achieving 1,1′-naphthylindoles by this strategy is extremely challenging.

We realized this strategy enlightened by our previous work of Cu(i)-catalyzed 6-*endo-dig* cyclization of terminal alkynes, 2-bromoaryl ketones, and amides for the synthesis of 1-naphthylamines,^17*a*^ in which the formation of the enamine intermediate favors the carbocyclization of the C–C triple bond. We envisioned that an enamine intermediate might be produced by condensation between two arylalkynes substituted with an amino and carbonyl under suitable conditions, which may trigger 6-*endo-dig* carbocyclization of carbonyl alkyne and 5-*endo-dig* nitrocyclization of amino alkyne, and the above undesired reactions could be suppressed. Thus, C–N axial biaryl compounds would be selectively produced.

Based on the above considerations, we then screened the reaction parameters using 2-(*p*-tolylethynyl)aniline (1a) and 2′-phenylethynylacetophenone (1b) as testing substrates. In fact, many types of undesired reactions simultaneously occurred, resulting in poor selectivity in our initial investigations (for details, see the ESI, Tables S1–S6†). Fortunately, we finally achieved excellent selectivity and yield of the desired product (1c) by the treatment of the two substrates with CuI (10 mol%) and Cs_2_CO_3_ (2.0 equiv.) in 1,1,1,3,3,3-hexafluoropropan-2-ol (HFIP) at 100 °C for 24 h (94% yield, Table 1, entry 27).

**Table 1:** Optimization of reaction conditions^a^

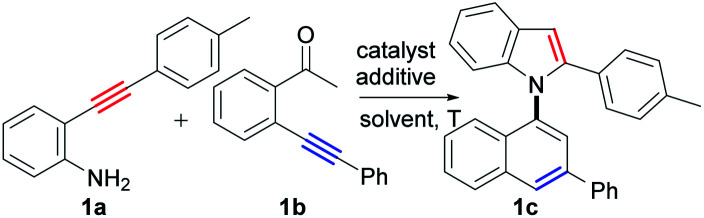					
Entry	[Cu]	Solvent	*T* (°C)	Additive	Yield^b^ (%)
1	CuCl	HFIP	100	—	64
2	CuBr	HFIP	100	—	70
3	CuOAc	HFIP	100	—	79
**4**	**CuI**	**HFIP**	**100**	**—**	**86**
5	CuCl_2_	HFIP	100	—	8
6	Cu(OAc)_2_	HFIP	100	—	10
7	Pd(OAc)_2_	HFIP	100	—	7
8	PdCl_2_	HFIP	100	—	6
9	CuI	TFE	100	—	60
10	CuI	PFTB	100	—	66
11	CuI	EtOH	100	—	4
12	CuI	Propanol	100	—	Trace
13	CuI	IPA	100	—	10
14	CuI	TBA	100	—	8
15	CuI	Toluene	100	—	Trace
16	CuI	DMF	100	—	Trace
17	CuI	THF	100	—	10
18	CuI	DCM	100	—	19
19	CuI	HFIP	60	—	53
20	CuI	HFIP	120	—	82
21	CuI	HFIP	100	Na_2_CO_3_	84
22	CuI	HFIP	100	K_3_PO_4_	87
23	CuI	HFIP	100	*t*-BuOK	88
24	CuI	HFIP	100	Cs_2_CO_3_	91
25	CuI	HFIP	100	TsOH	Trace
26	CuI	HFIP	100	HOAc	Trace
**27** c	**CuI**	**HFIP**	**100**	**Cs**_**2**_ **CO**_**3**_	**94**
28^d^	CuI	HFIP	100	Cs_2_CO_3_	95
29^e^	CuI	HFIP	100	Cs_2_CO_3_	94

a Reaction conditions: unless otherwise stated, all reactions were performed with 1a (0.05 mmol), 1b (1.0 equiv., 0.05 mmol), catalyst (10 mol%), and additive (2.0 equiv., 0.1 mmol) in the solvent (0.5 mL) at 100 °C for 24 h under N_2_ atmosphere.

b GC yield based on 1a using dodecane as an internal standard.

c 1a (0.05 mmol), 1b (0.06 mmol).

d HFIP (1.0 mL).

e HFIP (1.5 mL). TFE (2,2,2-trifluoroethanol), PFTB (perfluoro-*tert*-butyl alcohol), IPA (iso-propyl alcohol), TBA (*tert*-butyl alcohol).

The Cu(i) catalyst and HFIP are the most pertinent to the successful realization of the transformation. The reaction parameters are shown in Table 1 and summarized as follows: (1) the choice of Cu(i) catalyst was critical to this condensation/bicycloaromatization reaction (Table 1, entries 1–4). Cu(i) salt could activate the ketone by the interaction of Cu(i) with its O-atom, which improves its electrophilicity and facilitates the addition of amino to carbonyl groups, which favors the condensation between two different alkynes. Although Cu(i) could also catalysze the cyclization reactions of the two alkynes, respectively (see ESI, Scheme S3†), the condensation reaction preferentially occurred in the reaction system (the intermediate 1e′ was formed in 10 min, *vide infra*). Among Cu(i) catalysts, CuI exhibited the best efficiency (Table 1, entry 4). In comparison, Cu(ii) and Pd(ii) catalysts have much stronger metal–π interactions with C–C triple bond than Cu(i) catalysts, which strongly activate the C–C triple bond and lead to the preferential occurrence of many undesired reactions of the substrates (Table 1, entries 5–8), such as hydration of 1a, intramolecular cyclization of 1a and 1b, and di/polymerizations of 1a and 1b. For example, when CuCl_2_ and PdCl_2_ were used as the catalysts, more than 10 products were detected, and the desired product was only observed in 8% and 6% yields, respectively (Table 1, entries 5 and 8; for details, see the ESI, Table S1,† entries 5 and 9). Overall, the Cu(i) salt could catalyze the condensation between two amino alkynes and ketone alkynes, 6-*endo-dig* carbocyclization and 5-*endo-dig* nitrocyclization, avoiding the occurrence of the undesired reactions of the substrates. (2) Fluorinated alcohols are essential for the reaction as solvents (Table 1, entries 4, 9 and 10). Remarkably, the yield of the desired product was increased to 86% when HFIP was employed as the solvent (Table 1, entry 4). Other solvents, such as EtOH, propanol, IPA, TBA, toluene, DMF, THF, and DCM, gave very low yields and selectivities (Table 1, entries 11–18; see the ESI, Table S2†). (3) The reaction temperature was an important parameter for the reaction (see the ESI, Table S3†). Low temperatures, such as 60 °C, lowered the conversion of 1a (76%) and the selectivity of 1c (53% yield, Table 1, entry 19). High temperatures, such as 120 °C, slightly reduced the reaction selectivity, and an 82% yield of 1c was observed (Table 1, entry 20). (4) The addition of Na_2_CO_3_ (84%), K_3_PO_4_ (87%), *t*-BuOK (88%) and Cs_2_CO_3_ (91%) slightly improved the yield of the desired product. In comparison, the reaction failed with acidic additives, such as TsOH and acetic acid, and other reactions, such as hydration, dimerization, and intramolecular cyclization of the substrates, predominantly occurred (Table 1, entries 25 and 26; see the ESI, Table S5†). Finally, by improving the ratio of 1a and 1b to 1 : 1.2, a 94% yield of 1c was obtained with excellent selectivity (Table 1, entry 27). It was noted that any of the other products of the cross-coupling and cross-coupling/bicycloaromatization between 1a and 1b were not detected under the optimal reaction conditions, which also strongly facilitated the production isolation.

### Substrate scope

**Scheme 3:**
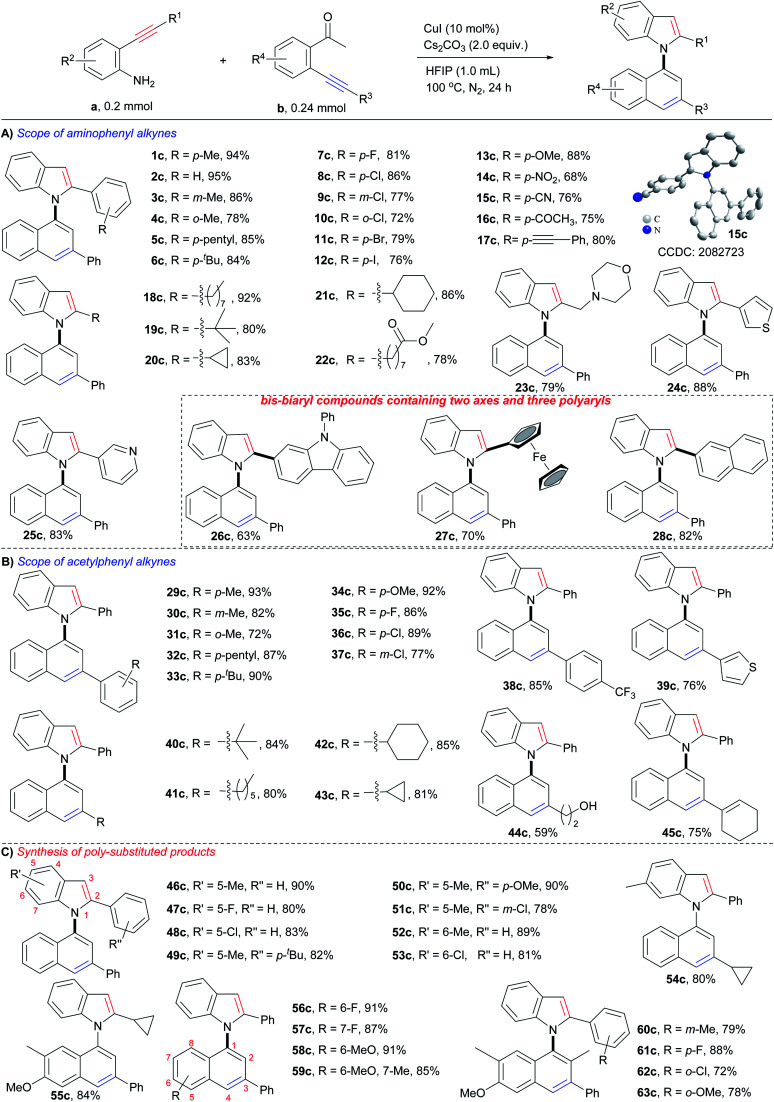
Substrate scope.

With the optimum reaction conditions in hand, the substrate scope of the reaction was examined. As shown in Scheme 3, each type of *o*-aminophenyl alkyne (*o*-aminophenyl aryl alkynes, *o*-aminophenyl alkyl alkynes, and *o*-aminophenyl heteroaryl alkynes) and *o*-acetylphenyl alkyne (*o*-acetylphenyl aryl alkynes, *o*-acetylphenyl alkyl alkynes, and *o*-acetylphenyl heteroaryl alkynes) were viable substrates for this cyclizative condensation reaction, and a variety of 1,1′-naphthylindoles were efficiently produced in synthetically useful yields with outstanding functional group tolerance. With respect to *o*-aminophenyl arylalkynes (Scheme 3A), aryl rings with various substitutions performed well (1c–17c). Alkyl groups, such as *p*-methyl (1c, 94%), *p*-pentyl (5c, 85%), and *p*-tertiary butyl (6c, 84%), gave high to excellent yields of the desired products. Halogens, especially iodine, which is a ready leaving group in coupling reactions, were tolerable in this reaction system, and halogenated products were obtained in high yields (*p*-F, 7c, 81%; *p*-Cl, 8c, 86%; *p*-Br, 11c, 79%; *p*-I, 12c, 76%), which allows for further functionalization. Steric hindrance had a slight effect on the reaction, and *m*-methyl (3c, 86%) and *o*-methyl (4c, 78%) substituted products were observed in slightly lower yields than that of the *p*-methyl substituted product (1c, 94%), as well as halogen (8c, *p*-Cl, 86%; 9c, *m*-Cl, 77%; 10c, *o*-Cl, 72%). Other electron donating and electron withdrawing groups were tolerated in this reaction and 13c (*p*-OMe, 88% yield), 14c (*p*-NO_2_, 68% yield), and 15c (*p*-CN, 76% yield) were produced in satisfactory yields. There is no significant electronic effect because the electron-donating (electron withdrawing) groups improving (lowering) the nucleophilicity of the N-atom but lowering (improving) the electrophilicity of the C–C triple bond. It was noted that acetyl (16c, 75% yield) and alkynyl (17c, 80% yield) groups, which are potentially reactive in the reaction, survived. This result suggested that the formation of an enamine intermediate (*vide infra*) favored bicycloaromatization and suppressed the other reactions of carbonyl and alkynyl groups.

*o*-Aminophenyl alkyl alkynes containing long chain (18c, 92% yield), sterically hindered *tert*-butyl (19c, 80% yield), cycles (cyclopropyl, 20c, 83% yield; cyclohexyl, 21c, 86% yield), ester (22c, 78% yield), and N,O-heterocycle (morpholine, 23c, 79% yield), were viable substrates for the reaction, and the corresponding *N*-naphthyl 2-alkyl substituted indoles were smoothly furnished.

Heteroaromatic alkynes that contain thiophene (24c, 88%), pyridine (25c, 83%), carbazole (26c, 63%), and ferrocene (27c, 70%) readily reacted with 2′-phenylethynylacetophenone, affording the corresponding *N*-naphthyl 2-heteroaryl indoles in satisfactory yields. Polycyclic aromatic alkynes, as exemplified by *o*-aminophenyl naphthyl alkynes, exhibited high reactivity (28c, 82% yield). *Notably,26c–28care bis-biaryl compounds that contain a C–N axis, a C–C axis, and three polyaryl rings, demonstrating that this method could prepare more structurally complex biaxial arylcompounds.*

Next, the scope of *o*-acetylphenyl alkynes was investigated (Scheme 3B). All three kinds of *o*-acetylphenyl alkynes (*o*-acetylphenyl aryl alkyne, *o*-acetylphenyl alkyl alkyne, and *o*-acetylphenyl heteroaryl alkyne) exhibited high reactivity in the reaction, and a variety of *N*-(3-aryl, -alkyl, and -heteroaryl)naphthyl indoles (29c–43c) were readily furnished in high to excellent yields (72–93%). Similar compatibility of functional groups to that of *o*-aminophenyl alkynes (1c–28c) was observed, as well as electronic and steric effects. The successful incorporation of reactive hydroxyl (44c, 59% yield) and alkenyl (45c, 75% yield) groups into the desired products further verified the outstanding functional group tolerance of the reaction.

In addition, substrates with multiple substituents on the *o*-aminophenyl and *o*-acetylphenyl rings also worked well (Scheme 3C), delivering the desired naphthylindoles in 78–91% yields (46c–59c). This feature makes the reaction practical for the synthesis of polysubstituted naphthylindoles, which cannot be achieved by other methods. Notably, *o*-aminophenyl alkynes also reacted smoothly with an *o*-propionylphenyl alkyne, producing naphthylindoles in 72–88% yields (60c–63c) with incorporation methyl group into 2-position of naphthyl ring.

### Synthesis of C–N axial heterobiaryl compounds

**Scheme 4:**
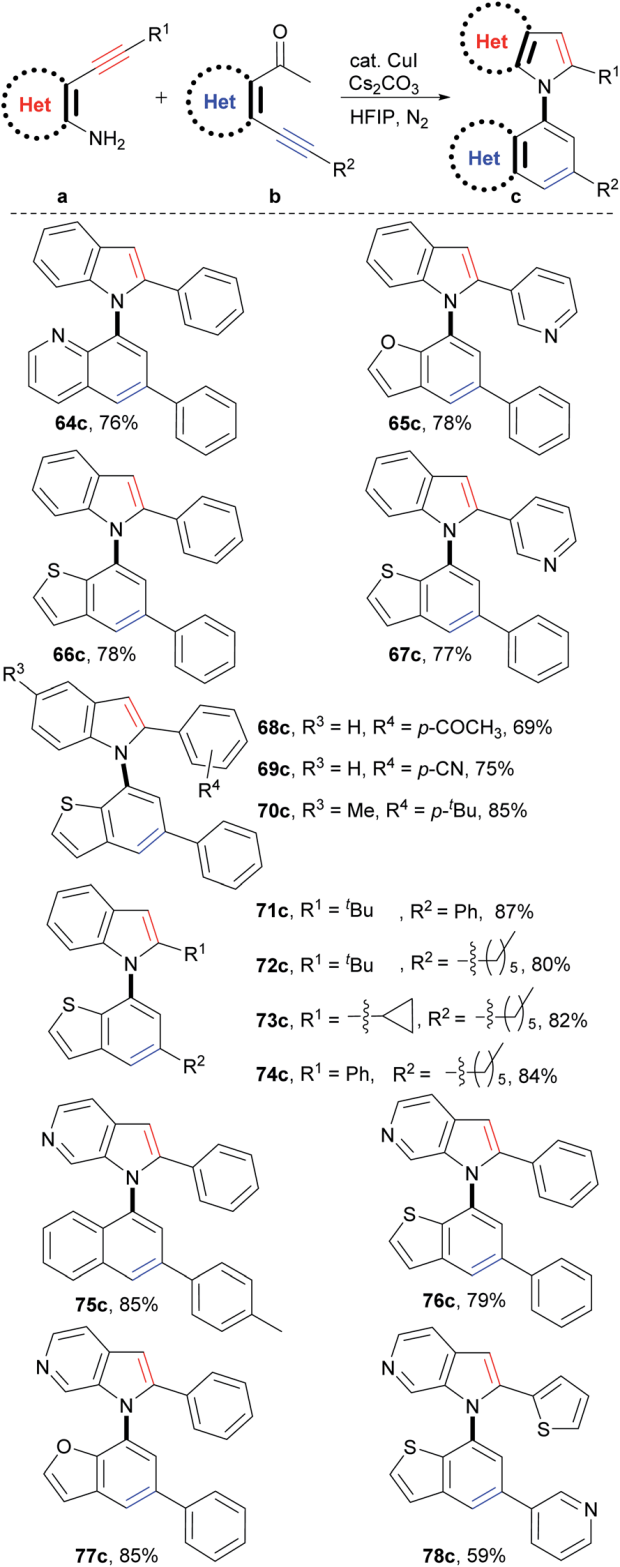
Scope of heteroaryl alkynes. ^a^Reaction conditions: a (0.05 mmol), b (1.2 equiv., 0.06 mmol), CuI (10 mol%), Cs_2_CO_3_ (2.0 equiv.), HFIP (1.0 mL), 100 °C, 24 h, N_2_. Isolated yields are provided.

Theoretically, C–N axial heterobiaryl compounds are structurally diverse. For example, *N*-quinolylindoles, *N*-benzofurylindoles, *N*-benzothienylindoles, *N*-naphthylpyrrolopyridines, benzothienyl pyrrolopyridines, and 1-benzofuryl pyrrolopyridines are this kind of compound. However, unlike naphthylindoles, they cannot or can rarely be constructed by traditional methods. In fact, only a few simple quinolylindoles and naphthylpyrrolopyridines have been reported by multiple-step synthesis.^28^ Despite limited examples, they actually exhibit a broad spectrum of bioactivities, such as antidiabetic, antigout, anti-inflammatory, antiangiogenic, antitumor, and transport protein inhibitors. We then applied this reaction to synthesize C–N axial heterobiaryl compounds (Scheme 4). Fortunately, *o*-acetylheteroaryl alkynes, such as *o*-acetylphridyl phenyl alkyne, *o*-acetylfuryl phenyl alkyne, and *o*-acetylthienyl phenyl alkyne, reacted smoothly with *o*-aminophenyl phenyl alkyne, and *N*-quinolylindole 64c, *N*-benzofurylindole 65c, and *N*-benzothienylindole 66c were readily produced in 76%, 78%, and 78% yields, respectively. Functional groups, as exemplified by pyridyl (67c), substituted phenyl (68c–70c) and alkyl (71c–74c) groups were readily incorporated into the C–N axial heterobiaryl skeletons (69–87% yields). The similar scope of substrates and tolerance of functional groups to those of the reaction for the synthesis of 1,1′-naphthylindoles demonstrated that C–N axial heterobiaryl compounds attaching various functional groups could be synthesized by this strategy. *o*-Aminoheteroaryl alkyne, exemplified by *o*-aminopyridyl phenyl alkyne, also worked well, which gave *N*-naphthylpyrrolopyridine 75c in 85% yield. To our delight, C–N axial biheteroaryl compounds, such as benzothienyl pyrrolopyridine 76c (79% yield) and 1-benzofuryl pyrrolopyridine 77c (85% yield), were also prepared in high yields by the reaction of 4-(phenylethynyl)pyridin-3-amine with 1-(3-(phenylethynyl)thiophen-2-yl)ethan-1-one and 1-(3-(pyridin-3-ylethynyl)thiophen-2-yl)ethan-1-one, respectively. Product 78c, which contains five heteroaryl rings, was obtained in moderate yield (59%). Therefore, the successful construction of the six types of heterobiaryl compounds greatly enriches the types of axial biaryl compounds, which makes it possible to develop new functions and diverse applications of biaryl compounds in chemistry, biology, and material science.

### Mechanism investigations

**Scheme 5:**
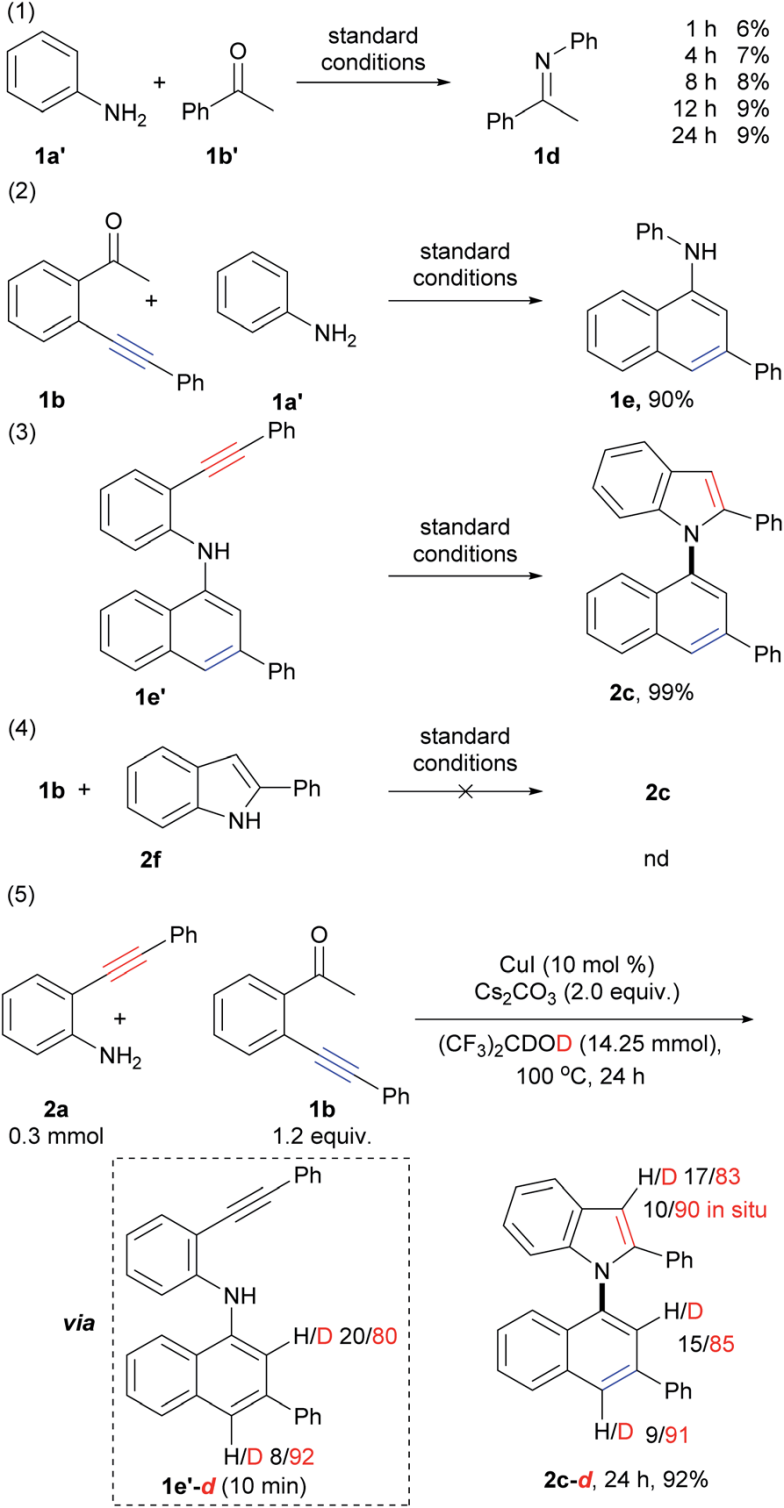
Control experiments.

To gain additional insights into the reaction mechanism, a series of control experiments were carried out (Scheme 5; for details, see the ESI, Schemes S3–S6†). Initially, by the treatment of aniline (1a′) with acetophenone (1b′) under standard conditions for 1 h, 4 h, 8 h, 12 h, and 24 h, imine 1d was observed in 6%, 7%, 8%, 9%, and 9% yields, respectively (Scheme 5, eqn (1)). This result suggested that condensation of amino and carbonyl groups occurred, and imine does not be formed as the major product but takes dynamic equilibrium with the substrates. The reaction of 1b with aniline (1a′) instead of 1a gave 1-naphthylamine 1e in 90% yield (Scheme 5, eqn (2)). This result demonstrated that 6-*endo-dig* carbocyclization of 1b with arylamines *via* the formation of the enamine intermediate could proceed smoothly. The above results also suggested that imine, formed from the condensation of amino and carbonyl groups, could tautomerize to the enamine form, which triggers nucleophilic attack to the adjacent C–C triple bond to form 1-naphthylamine. Compound 1e′ was subjected to intramolecular cyclization to produce 1,1′-naphthylindole (2c) in almost quantitative yield (99%, Scheme 5, eqn (3)). However, the treatment of 1b with 2-phenylindole (2f) did not give the desired product (Scheme 5, eqn (4)). The above results indicated that 6-*endo-dig* carbocyclization occurred prior to 5-*endo-dig* nitrocyclization. Indeed, intermediate 1e′ could be isolated at the initial stage (10 min) of the reaction of 2a with 1b, which further confirmed the suggestion (ESI, Scheme S5,† eqn (F)).

Next, deuterium labeling experiments were performed (Scheme 5, eqn (5), for details, see the ESI, Schemes S7–S10†). The reaction was carried out by replacing HFIP with (CF_3_)_2_CDOD (HFIP-*d*_2_) under standard conditions, and the corresponding deuterated product (2c-*d*, 24 h) was obtained in 92% yield with deuterium incorporation at the C-2 (85% deuteration) and C-4 (91% deuteration) positions on the naphthalene ring and the C-3 position (83% deuteration, 90% deuteration *in situ*) on the indole ring (Scheme S7,† eqn (K), Fig. S41†). The different D contents suggested that they were derived from different pathways. It was also confirmed that the H/D exchange did not occur between the solvent (HFIP-*d*_2_) and the C–H bond of the naphthalene ring in intermediate E and product c (ESI, Scheme S10,† eqn (S)–(V)). This result suggested that the D atom of C-2 and C-4 positions of naphthalene ring in product 2c-*d* did not directly derived from solvent by H/D exchange between the solvent with product 2c and intermediate 1e′.

As shown in eqn (5) of Scheme 5, the D content of C2 and C4 on the naphthalene ring of intermediate 1e′-*d* (isolated at 10 min, ESI, Scheme S7,† eqn (K)) are 80% and 92%, respectively. The D content of C4 on the naphthalene ring of intermediate 1e′-*d* (92%) and product 2c-*d* (91%) were close to that of the reaction system (deuterium content of the total reactive H and D that could be exchanged, 89.5–93.0%), which suggested that the D atom of C-4 position on the naphthalene ring was derived from the reaction system *via* protonation of vinyl copper intermediate D (*vide infra*).

The D content of C-2 position on the naphthalene ring of intermediate 1e′-*d* (80%, 10 min) and product 2c-*d* (85%) were lower than those of C4 (92% and 91%) and the reaction system, but higher than that of substrate 1b-*d* (44% D, 10 min, Scheme S7,† eqn (K)). These results suggested that the D atom of C-2 was derived from two pathways: (1) substrate 1b-*d* by H/D exchange of substrate 1b with the reaction system; (2) direct H/D exchange during condensation and 6-*endo-dig* carbocyclization (A to D, *vide infra*). The *in situ* deuterium content (90%) of the C-3 position on the indole ring of 2c-*d* was also close to that of the reaction system (89.5–93.0%), which was much higher than D content of substrate 2a-*d* (about 30%, see ESI, Scheme S7,† eqn (K)). Direct cyclization of intermediate 1e′ using HFIP-*d* as solvent (ESI, Scheme S10,† eqn (S)) showed that the D content of the C-3 position on the indole ring of the product was about 90% from 10 min to 24 h, which was also close to the reaction system (89.5–93.0%). Whereas, the D content of the N–H of the 1e′-*d* were *ca.* 31% D and 44% D at 10 min and 1 h, respectively. These results suggested D atom of C3 mainly derived from the protonation of vinyl copper intermediate F with the reaction system.

**Scheme 6:**
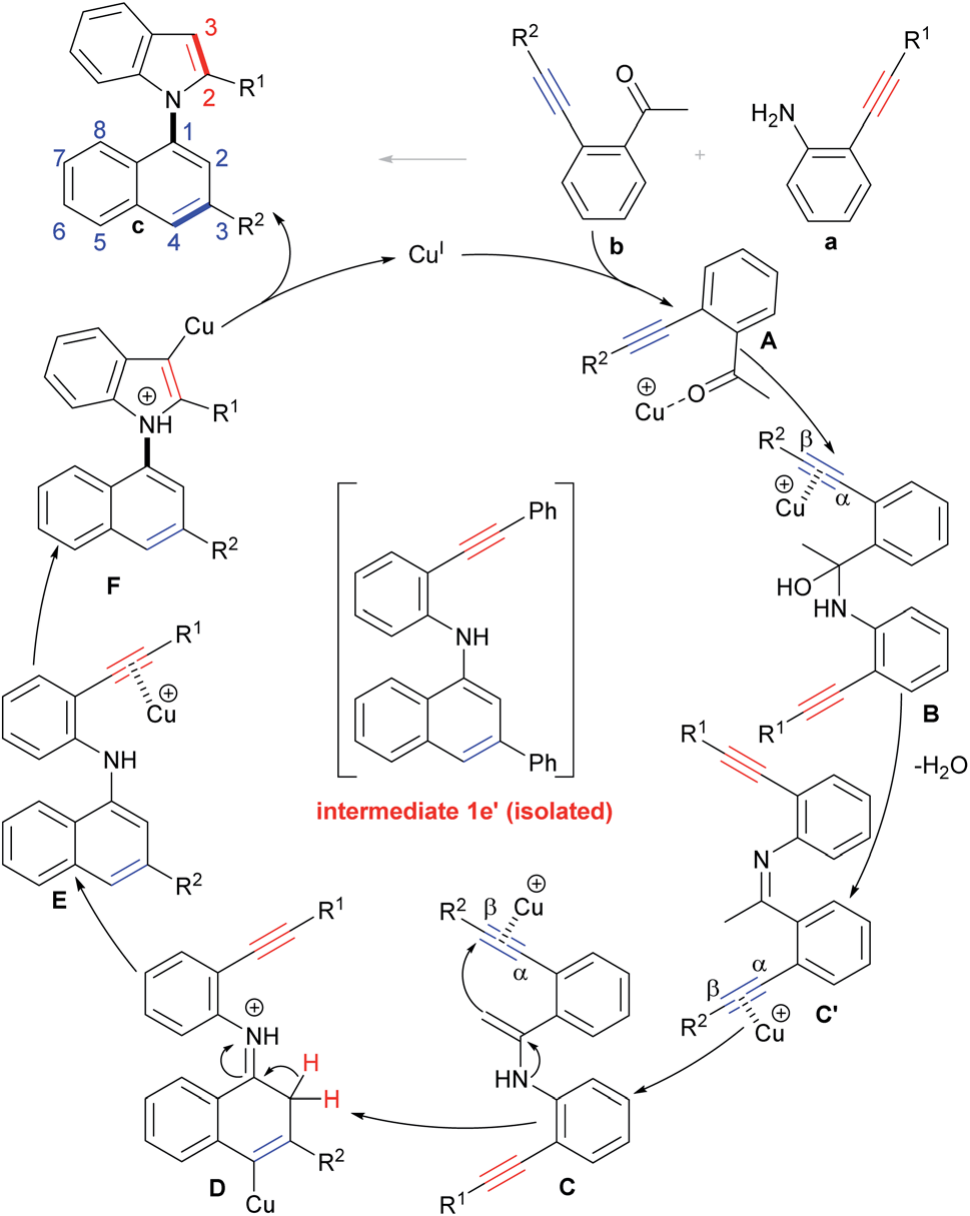
Possible reaction mechanism.

On the basis of the above results and literature reports,^17*a*,29^ a tentative mechanism for the selective condensation/bicycloaromatization reaction is proposed (Scheme 6). First, the interaction of Cu(i) with the O-atom of the carbonyl of *o*-acetylphenyl alkyne b forms intermediate A, and then the nucleophilic attack of the N-atom of *o*-aminophenyl alkyne a on its carbonyl group leads to the formation of α-amino alcohol B, followed by dehydration to produce imine intermediate C′, then the Cu(i) promotes tautomerization of imine C′ to enamine C.^29^ Intramolecular 6-*endo-dig* carbocyclization of C gives intermediate D, which simultaneously proceeds with deprotonation and protonation to produce (*N*-naphthyl)aminophenyl alkyne E. Intermediate E undergoes 5-*endo-dig* nitrocyclization with subsequent protonation to produce desired product c.

### Asymmetric synthesis of axially chiral naphthylindoles

Axially chiral biaryl compounds are important structural skeletons in natural products and chiral ligands,^30^ and asymmetric synthesis of atropoisomeric biaryls has been booming in recent years.^14*a*,31–33^

**Scheme 7:**
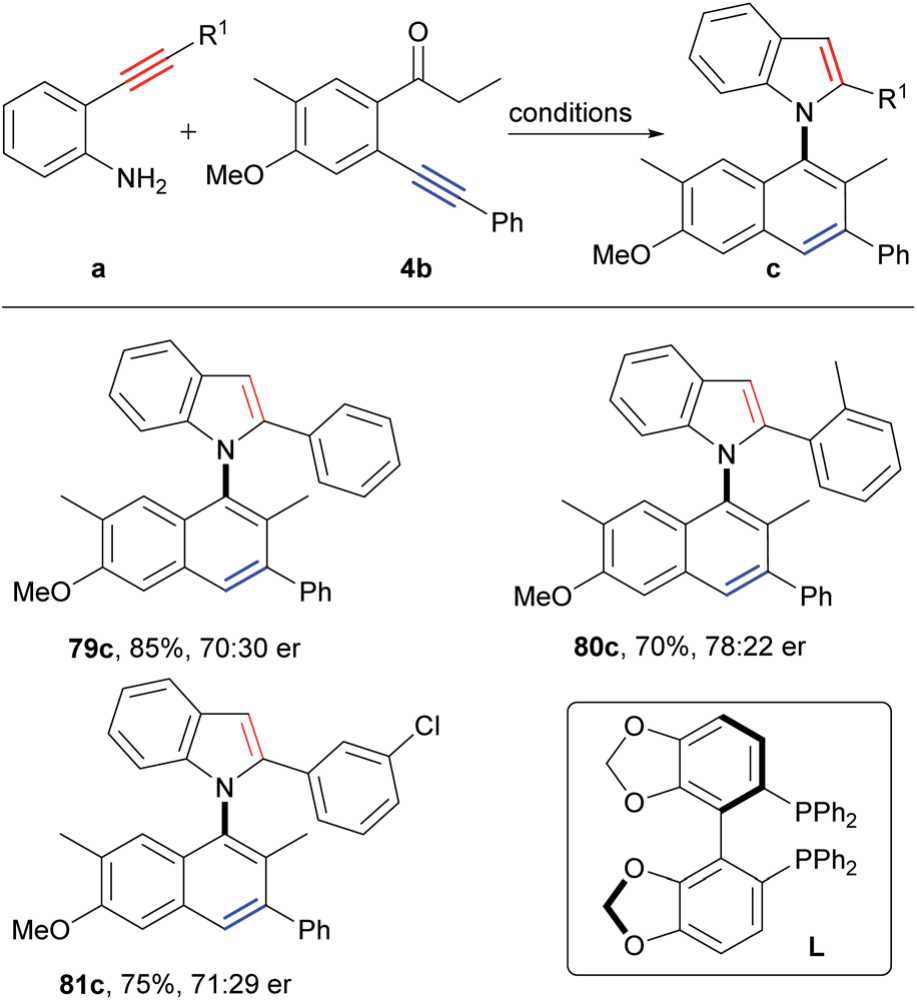
Substrate scope of axially chiral compounds. Reaction conditions: (1) a (0.2 mmol), 4b (1.2 equiv.), CuI (5 mol%), Cs_2_CO_3_ (2.0 equiv.) HFIP, 80 °C, 50 min; (2) PdCl_2_ (5 mol%), L (6 mol%) HFIP, 80 °C, 15 h.

Clearly, the asymmetric synthesis of axially chiral biaryl compounds *via* direct construction of an axis and two aromatic rings from two simple aryl alkynes is extremely appealing, which provides a new dimension for the direct synthesis of atropoisomeric biaryls from easily available starting materials. Then, we attempted to construct axially chiral naphthylindoles by this strategy. Firstly, many chiral ligands, such as chiral phosphoric acid ligands, N, N, N-tridentate ligands, N, N, P-tridentate ligands, N, N-bidentate ligands, N, P-bidentate ligands and P, P-bidentate phosphine ligands under copper catalysis (Table S9,† entries 1–19), however, only chiral bis(oxazoline) ligand (L2) gave the enantioselectivity of 23% ee at 40 °C (Table S9,† entry 2), 15% ee at 60 °C (Table S9,† entry 22), and 7% ee at 80 °C (Table S9,† entry 24), respectively. According to reaction mechanism, the key step for asymmetric synthesis is the 5-*endo-dig* nitrocyclization of intermediate E. We proposed that a suitable asymmetric catalyst that favored asymmetric nitrocyclization of alkynes would be beneficial for the asymmetric reactions. By further optimization of reaction conditions, we found that the addition of PdCl_2_/(*R*)-SEGPHOS to the reaction after stirring the reaction mixture for 50 minutes at 80 °C could significantly improve the enantioselectivity to 40% ee (Table S10,† entry 34), which is slightly lower than that (51% ee, at 80 °C) of the direct asymmetric nitrocyclization of the corresponding intermediate 2e′ (Scheme S11†). Slightly higher enantioselectivities were observed by the employment of *o*-aminophenyl *o*-substituted arylalkynes as the substrates (80c, 56% ee; 81c, 42% ee; Scheme 7). Although moderate enantioselectivity was observed at this stage, it verified the potential of the simplest asymmetric synthesis of atropoisomeric biaryls.

### Bioactivity properties

**Fig. 2:**
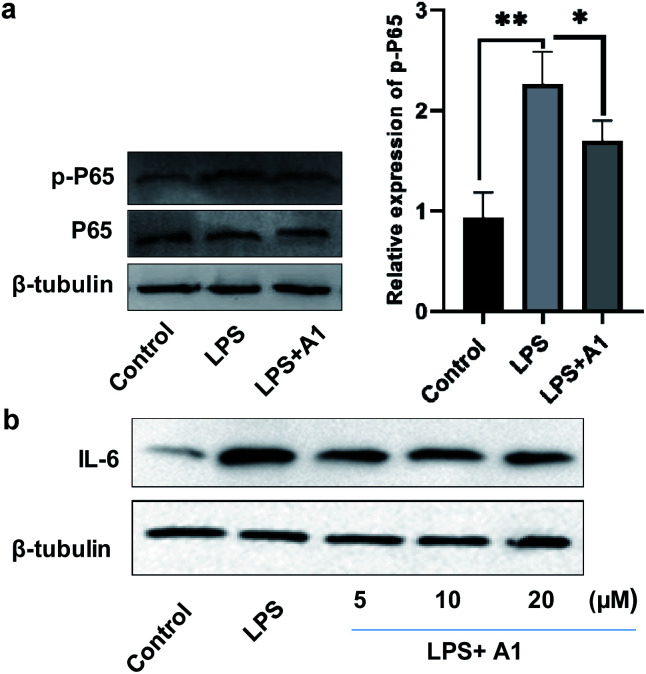
BV2 cells were pretreated with 10 μM A1 for 2 h and then treated with LPS (100 ng mL^−1^) for 6 h. (a) The levels of P65 and the corresponding phosphorylated form were measured by western blotting (*n* = 3 per group). Compared with the control group, **P < 0.01, *vs.* LPS group, *P < 0.05. (b) BV2 cells were pretreated with different concentrations (5–20 μM) of A1 for 2 h and were then treated with 100 ng mL^−1^ LPS for 6 h. The levels of IL-6 were detected by western blotting (A1 = 15c).

Finally, to evaluate the bioactivity of this new type of naphthylindole, we initially tested the potential cytotoxicity of compounds 15c (A1), 44c, and 69c toward BV-2 cells by the MTT assay. Compounds 15c, 44c, and 69c at the indicated concentrations (0, 3, 10, 30, and 100 μM) showed no significant cytotoxicity on BV-2 cells (Fig. S46†). Then, we detected the effect of compound 15c on the expression of phosphorylated NF-κB (p-P65) and IL-6 in BV-2 microglial cells by western blotting. BV-2 microglial cells were pretreated with 10 μM A1 (15c) for 2 h before being stimulated with 100 ng mL^−1^ LPS for another 6 h. A1 (15c) attenuated LPS-induced NF-κB activation in BV-2 microglial cells (LPS 100 ng mL^−1^) compared with the control group, **P < 0.01, *vs.* the LPS group, *P < 0.05 (Fig. 2a). Moreover, we found that A1 (15c) also attenuated LPS-induced IL-6 production in BV-2 microglial cells in a concentration-dependent manner (Fig. 2b). In view of the above results, we speculate that compound 15c has anti-inflammatory bioactivity. Indeed, compound 15c has comparable anti-inflammatory effect to indomethacin (an indole derivative, non-steroidal anti-inflammatory drug) on the expression of 1L-1β and TNF-α in BV-2 microglial cells (see ESI, Fig. S47†). These results demonstrate that this method has great potential application in drug and biology.

## Conclusions

We have developed a condensation/bicycloaromatization reaction of two different arylalkynes under ligand-free copper catalysis for simple, efficient, and step-economic synthesis of C–N axial biaryl compounds. Due to the critical effects of Cu(i) catalyst and HFIP, many easily occurring undesired reactions are suppressed, and the coupled five–six aromatic rings are constructed *via* the selective formation of two C(sp^2^)–N(sp^2^) bonds and four C(sp^2^)–C(sp^2^) bonds. The formation of the enamine intermediate between the two different alkynes might be the key step for the reaction, which triggers selective tandem 6-*endo-dig* carbocyclization and 5-*endo-dig* nitrocyclization. Many types of biaryl compounds, such as naphthylindole, benzothio-phenelindole, quinolinelindole, benzofuranindole, benzothiophenyl-pyrrolopyridine, naphthyl-pyrrolopyridine, and benzofuranyl-pyrrolopyridine, are obtained in high yields (up to 95%), which are not or rarely synthesized by other methods. The compatibility of a very broad range of functional groups, such as alkyl, halogens (F, Cl, Br, and I), OMe, OH, vinyl, ester, NO_2_, CF_3_, CN, N,O-heterocycle, aryl, heteroaryl (pyridine, thiophene, furan, ferrocene, and carbazole), and even reactive functional groups [*i.e.*, acetyl and alkynyl], demonstrates that structurally and functionally diverse C–N axial biaryl compounds can be easily prepared from simple starting materials. The realization of moderate enantioselectivity of chiral naphthylindoles verifies its potential for the most direct asymmetric synthesis of atropoisomeric biaryls. Western blotting experiments demonstrated that these newly developed 1,1′-naphthylindole derivatives, such as 15c, have strong anti-inflammatory bioactivity and are promising targets in biology and pharmaceuticals. In summary, this unique reaction can prepare structurally diverse C–N axial biaryl compounds that have not been reported by other methods, represents an ideal strategy for the synthesis of functionalized C–N axial biaryl compounds, and may be highly useful in materials, chemistry, biology, and pharmaceuticals. Further work is in progress to exploit the related bioactivity properties of C–N axial biaryls and improve the enantioselectivity of this transformation.

## Author contributions

Y. Z. and J. D. conceived the work and coordinated the project. Q. S. conducted the experiments. J. D. initiated the project. Q. S. advanced and completed the project. Y. L., M. Y., and Y. C. carried out the western blotting experiment. W. Y. and Q. S. carried out asymmetric synthesis. H. T., L. S., S. X., and L. L. assisted the project. Y. Z. wrote the manuscript with Q. S., J. D., and S. Y.

## Conflicts of interest

The authors declare that there are no conflicts to declare.

## Supplementary Material

SC-013-D1SC03865F-s001

SC-013-D1SC03865F-s002
