# Supplementary material for: Cu(i) catalysis for selective condensation/bicycloaromatization of two different arylalkynes: direct and general construction of functionalized C–N axial biaryl compounds
Source: Chem Sci. 2021 Dec 13;13(1):263–73. doi: 10.1039/d1sc03865f (PMC8694356; doi:10.1039/d1sc03865f)
Supplement: SC-013-D1SC03865F-s001 [file SC-013-D1SC03865F-s001.pdf]

Electronic Supplementary Material (ESI) for Chemical Science.
This journal is © The Royal Society of Chemistry 2021

Cu(I) Catalysis for Selective Condensation/Bicycloaromatization of Two Different Arylalkynes: Direct and General Construction of Functionalized C−N Axial Biaryl Compounds

Qian Shang,[a] Haifang Tang,[a] Yongping Liu,[c] MingMing Yin,[c] Lebin Su,[a] Shimin Xie,[a] Lixin Liu,[a] Wen Yang,[a] Yi Chen,[c] Jianyu Dong,*[,b] Yongbo Zhou,*[,a] and Shuang-Feng Yin[a]

[a]Advanced Catalytic Engineering Research Center of the Ministry of Education, College of Chemistry and Chemical Engineering, Hunan University, Changsha 410082, China

[b]Department of Educational Science, Hunan First Normal University, Changsha 410205, China

[c]School of Medicine, Hunan University of Chinese Medicine, Changsha, 410208, China

E-mail: zhouyb@hnu.edu.cn

djyustc@hotmail.com

Tables of Contents

1. General Information S2

2. Possible Products from *o*-Amino Arylkyne 1a and/or *o*-Carbonyl Arylalkyne 1b S2

3. General Experimental Procedure S5

4. Investigations of the Reaction Mechanism S47

5. Asymmetric Synthesis S80

6. MTT assay S86

7. X-ray Crystallographic Data of 15c S88

8. Preparation of Substrates S90

9. Characterization Data for the Products S97

10. Copies of ^{1}H, ^{13}C and ^{19}C NMR Spectra of the Products S132

11. References S237

1. General Information

1.1 Materials and Instruments

Except noted otherwise, all reactions were carried out in Schlenk tubes under N_2 atmosphere. Reagents and solvents were obtained from commercial sources (Energy Chemical, Aladdin, Alfa Aesar, Sigma-Aldrich, and J&K Scientific) and used without further purification. The ^{1}H, ^{13}C and ^{19}F NMR spectra were recorded on a Brucker ADVANCE III spectrometer at 400 MHz, 101 MHz, and 376 MHz respectively, and chemical shifts were reported in parts per million (ppm). Flash column chromatography was performed using silica gel of 300–400 μm. The GC-MS results were recorded on a GC-MS QP 2010 equipment, GC analysis was performed on GC 2014 plus. The electron ionization (EI) method was used for HRMS measurement, and the mass analyzer type is TOF for EI. The single-crystal X-ray diffraction was recorded on Brucker D8 QUEST. The conversion of starting materials was monitored by thin layer chromatography (TLC) using silica gel plates (silica gel 60 F254 0.25 mm), and components were visualized by observation under UV light (254 and 365 nm). The enantiomeric excesses were determined by chiral HPLC using a Shimadzu Prominence LC-20A instrument with a Daicel Chiralcel OD-H column.

1.2 Cell Culture and Western Blot Analysis

BV-2 microglia cell line was grown in Dulbecco's modified Eagle's medium (DMEM) (PAN Biotech, Aidenbach, DE) supplemented with 10% (v/v) fetal calf serum (FCS), 2 mM L-glutamine, 100 mg streptomycin, and 100 U penicillin (GIBCO, Gran Island, NY, USA). The cells were cultured in Petri dishes at 37 °C with 5% CO_2 and saturated humidity. Cell viability was determined by the CCK-8 assay (Dojindo Molecular Technologies, Shanghai, China). The viability was assessed before each experiment as part of standard protocol and cell counting. We proceeded with the experiment if the cell viability was ≥ 95% in both cultivation conditions. Cellular extracts were prepared in RIPA lysis buffer supplemented with protease inhibitor. Proteins were separated by sodium dodecyl sulfate polyacrylamide gel electrophoresis (SDS-PAGE) and blotted onto a PVDF Western blotting membrane (GE Healthcare Limited, Amersham, UK).

2. Possible Products from o-Amino Arylkyne 1a and/or o-Carbonyl Arylalkyne 1b

As described in the main text, diverse types of reactions of both o-amino and o-carbonyl arylalkynes could occur. *o*-Amino arylkynes easily occur a series of reactions, such as hydration, intramolecular cyclization and dimerization, which produce amino aryl ketones,[1] indoles,[2] 2-(2-aminophenyl)quinoline,[3] etc. More types of reactions could also take place for *o*-carbonyl arylalkynes, producing diketone,[4] triketone,[5] 1-indanones,[6]

indenones,[7] 1-naphthols,[6] chalocones,[8] 1*H*-isochromene,[9] and chrysene,[10] etc. polymerizations of the two alkynes also could be occur.

Many possible products of the reactions of o-amino arylalkyne 1a and/or o-carbonyl arylalkyne 1b could be figured out. Simple possible products (except polymerization products) are as follows:

Scheme S1. Possible products from *o*-amino arylkynes 1a and/or *o*-carbonyl arylalkynes 1b

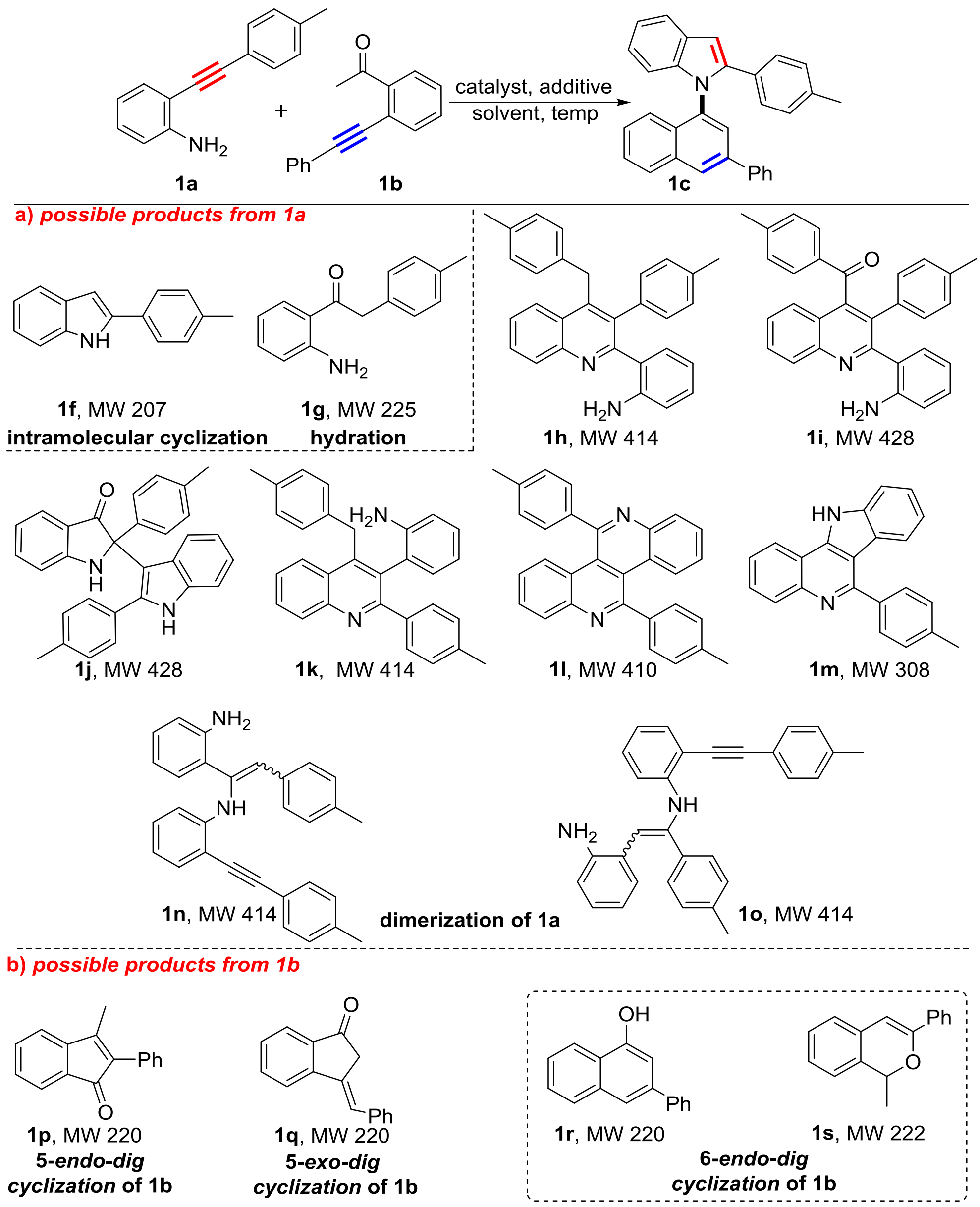

b) ***possible products from 1b*--continued**

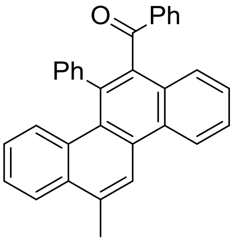

1t, MW 422

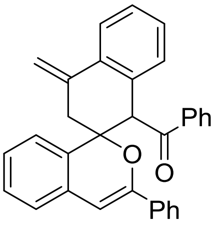

1u, MW 440

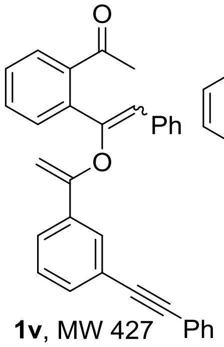

1v, MW 427

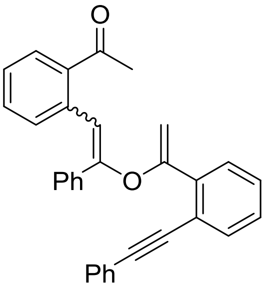

1w, MW 427

dimerization of 1b

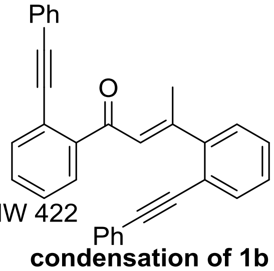

1x, MW 422

condensation of 1b

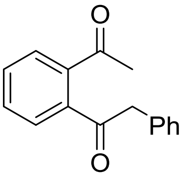

1y, MW 238

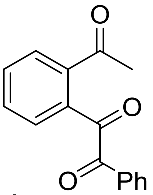

1z, MW 252

hydration of 1b

c) ***possible* cross products from1a and 1b**

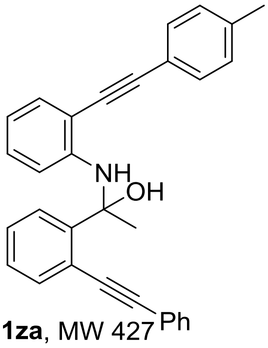

1za, MW 427

dimerization of 1a and 1b

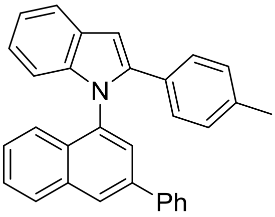

1c, MW 409

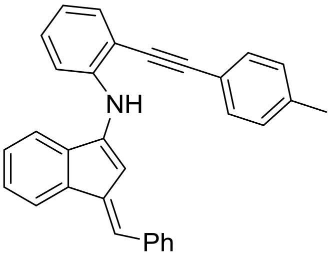

1zb, MW 409

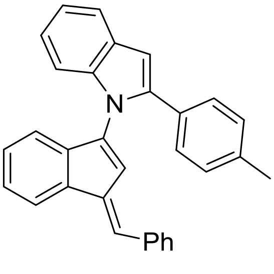

1zc, MW 409

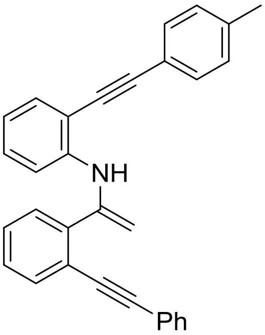

1zd, MW 409

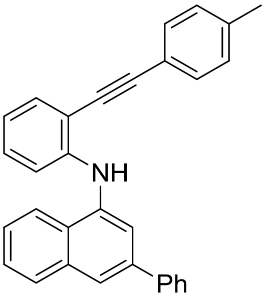

1ze, MW 409

condensation of 1a and 1b

3. General Experimental Procedure

3.1. Optimization of the Reaction Conditions

Table S1. Catalyst Screening

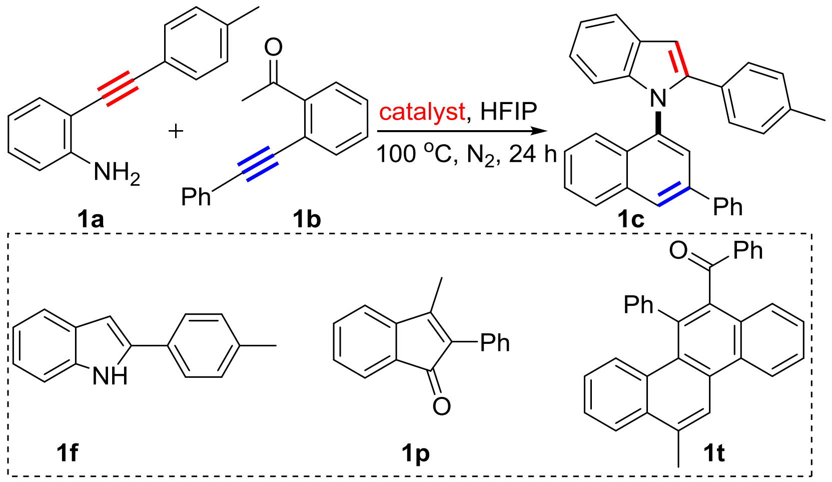

entry	catalyst	conversion [%][b]		yield [%][b]			
		**1a**	**1b**	**1c**	**1f**	**1p**	**1t**
1	CuCl	>99	>99	64	16	trace	trace
2	CuBr	>99	>99	70	21	trace	trace
3	CuOAc	>99	>99	79	12	trace	trace
**4**	**CuI**	**>99**	**>99**	**86**	**11**	**trace**	**trace**
5	$CuCl_2$	>99	>99	8	77	trace	trace
6	$Cu(OAc)_2$	>99	>99	10	32	trace	trace
7	$Pd(OAc)_2$	>99	>99	7	45	trace	trace
8[c]	$Pd(OAc)_2$	>50	>99	trace	48	trace	5
9	$PdCl_2$	>99	>99	6	56	trace	trace
10	Cu	>99	>90	38	20	trace	trace
11	Fe	>99	>85	27	25	trace	5
12	Mg	>99	>80	20	25	trace	7
13	$FeCl_3$	>99	>70	trace	40	trace	4
14	$ZnBr_2$	>99	>99	26	19	trace	trace
15	AgOAc	>99	>80	4	49	trace	8

16	$NiSO_4$	>99	>80	21	36	trace	4
17	$MnCO_3$	>99	>85	11	50	trace	trace
18	$Co(OAc)_2$	>99	>75	8	52	trace	5

[a]Reactions:**1a** (0.05 mmol), **1b** (0.05 mmol), Catalyst (10 mol %), in HFIP (0.5 mL) at 100 °C under N_2 for 24 h.

[b]GC yield using dodecane as an internal standard.

[c]DMF as Solvent.

*Notes: Many byproducts have been detected, and different **catalysts** gave different types and selectivities of products (Figures S1-S9). The byproducts of the intramolecular cyclization product (**1f**) of **1a**, intramolecular cyclization (**1p**) of **1b**, dimerization (**1t**) of **1b** were selected to be shown in Table S1.*

Selected GC and/or GC-MS charts of the reactions catalyzed by different catalysts.

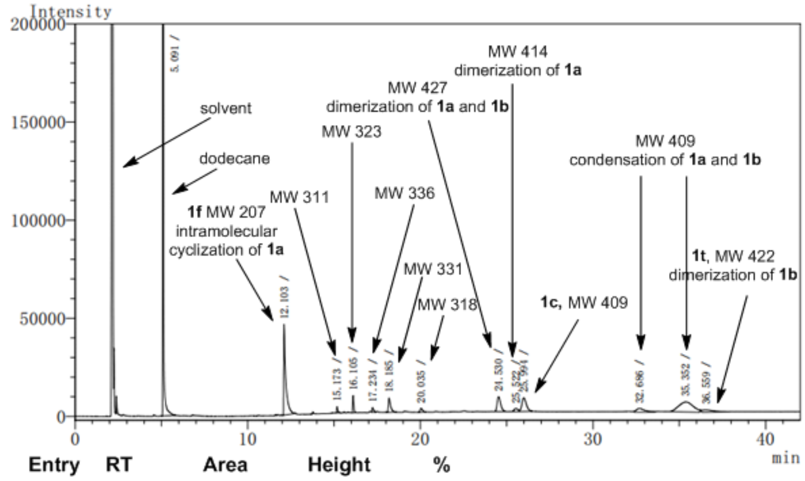

Entry	RT	Area	Height	%
1	5. 091	1772459	939707	59. 601
2	12. 103	373811	46160	12. 570
3	15. 173	15378	3208	0. 517
4	16. 105	37980	8806	1. 277
5	17. 234	13895	2345	0. 467
6	18. 185	65916	7294	2. 217
7	20. 035	18562	2012	0. 624
8	24. 530	107511	7549	3. 615
9	25. 522	23169	1530	0. 779
10	25. 994	122126	6877	4. 107
11	32. 686	69314	1857	2. 331
12	35. 352	296334	5057	9. 965
13	36. 559	57398	1173	1. 930
All		2973853	1033575	

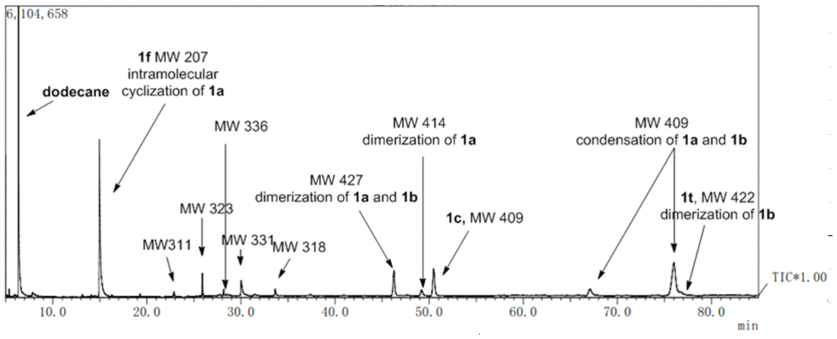

Figure S1. The GC and GC-MS charts of the crude reaction mixture by $PdCl_2$ catalysis (Table S1, entry 9)

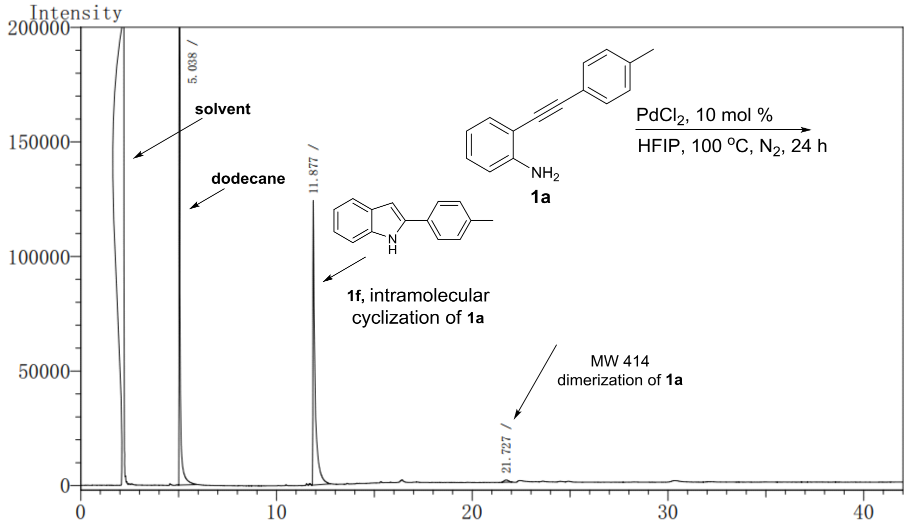

Entry	RT	Area	Height	%
1	5.038	1721694	867090	65.782
2	11.877	880466	123363	33.641
3	21.727	15117	950	0.578
All		2617277	991403	

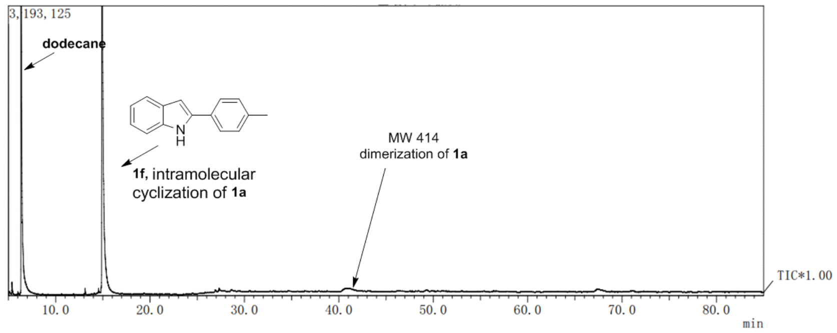

Figure S2. The GC and GC-MS charts of the crude reaction mixture of 1a by $PdCl_2$ catalysis

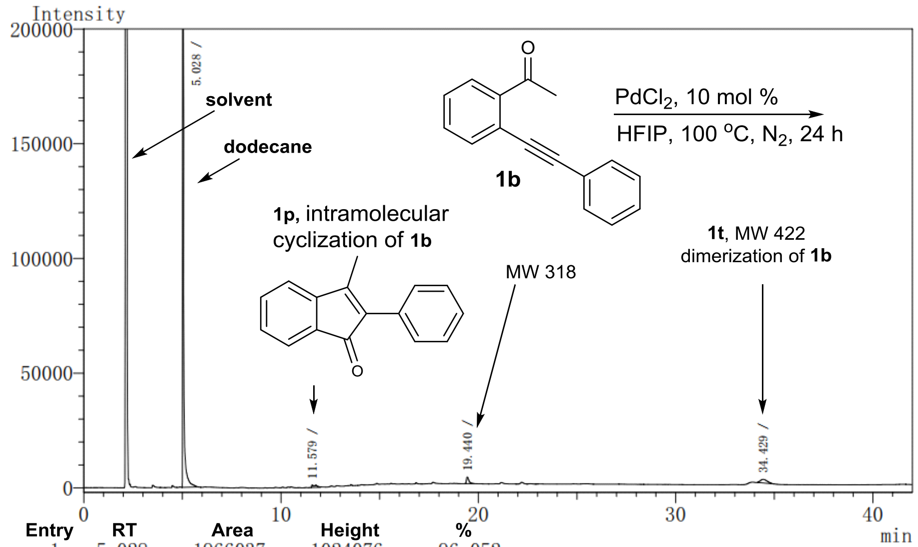

Entry	RT	Area	Height	%
1	5.028	1966037	1024076	96.052
2	11.579	17531	1107	0.856
3	19.440	24344	2913	1.189
4	34.429	38938	1567	1.902
All		2046850	1029663	

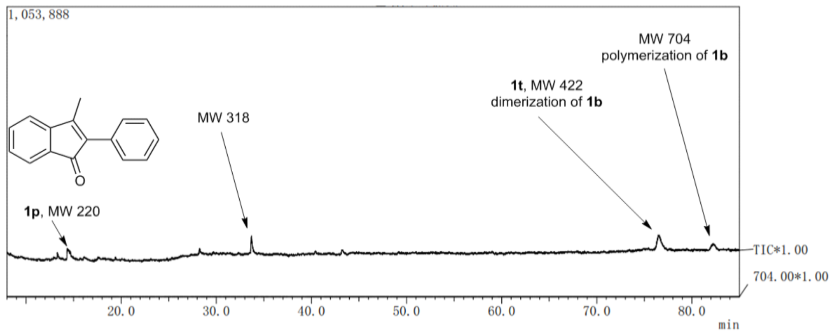

Figure S3. The GC and GC-MS charts of the crude reaction mixture of 1b by PdCl$_2$ catalysis

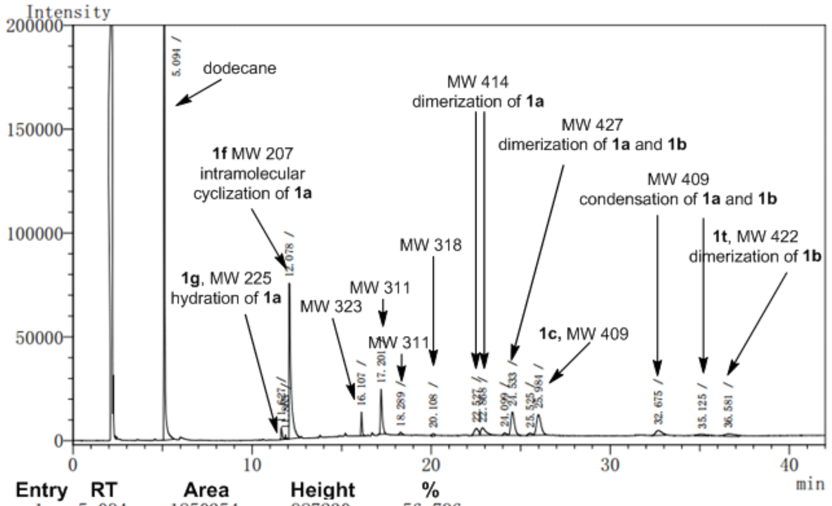

Entry	RT	Area	Height	%
1	5.094	1850354	987230	56.796
2	11.627	28567	5837	0.877
3	11.865	6793	2248	0.209
4	12.078	536690	74564	16.473
5	16.107	47902	11400	1.470
6	17.201	142381	21513	4.370
7	18.289	14197	1442	0.436
8	20.108	11845	1311	0.364
9	22.527	54569	3302	1.675
10	22.868	72637	3494	2.230
11	24.099	13153	1108	0.404
12	24.533	158090	11202	4.852
13	25.525	15565	1094	0.478
14	25.984	174033	9925	5.342
15	32.675	58578	2327	1.798
16	35.125	25137	741	0.772
17	36.581	47412	1170	1.455
All		3257903	1139908	

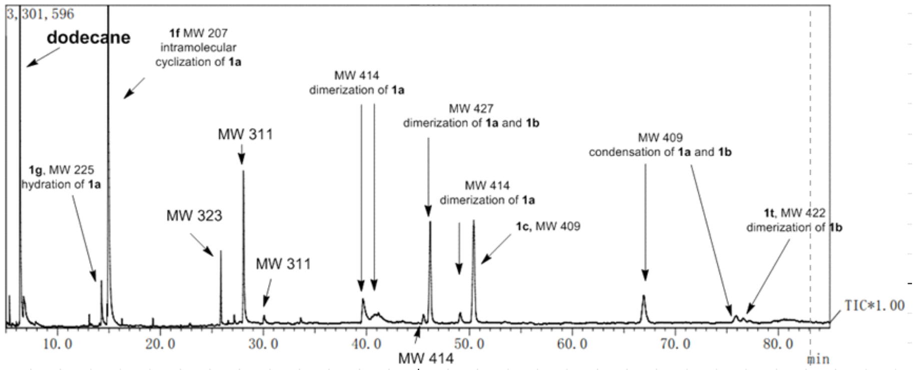

Figure S4. The GC and GC-MS charts of the crude reaction mixture by $CuCl_2$ catalysis (Table S1, entry 5)

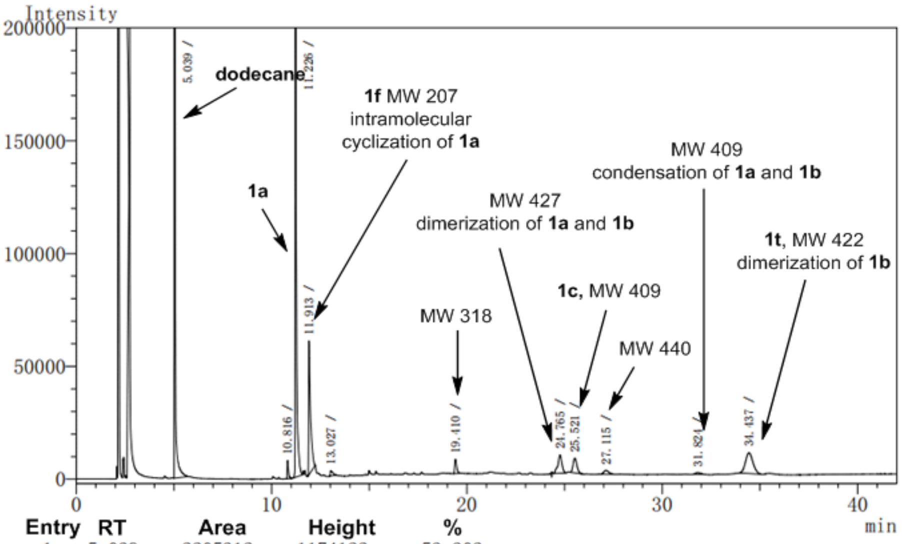

Entry	RT	Area	Height	%
1	5.039	2205312	1174122	53.302
2	10.816	33939	8143	0.820
3	11.226	938240	256126	22.677
4	11.913	398299	59346	9.627
5	13.027	20911	2433	0.505
6	19.410	45372	6359	1.097
7	24.765	117079	8136	2.830
8	25.521	88990	6543	2.151
9	27.115	29471	1678	0.712
10	31.824	14336	777	0.347
11	34.437	245424	9221	5.932
All		4137373	1532884	-

Figure S5. The GC chart of the crude reaction mixture by $Pd(OAc)_2$ catalysis in DMF (Table S1, entry 8)

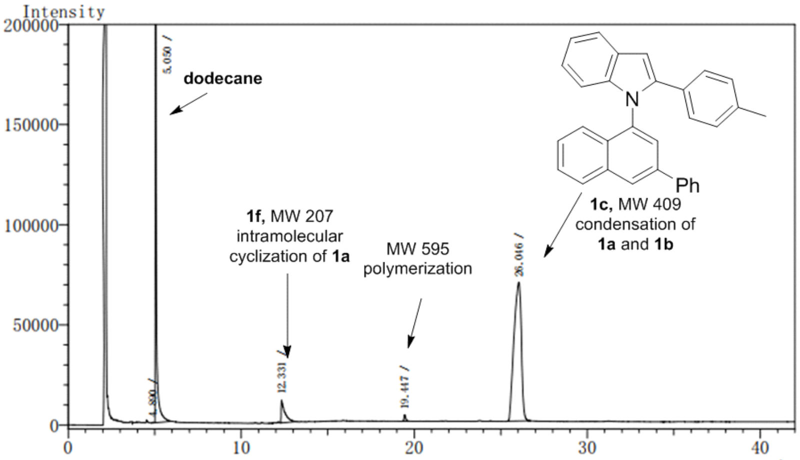

Entry	RT	Area	Height	%
1	4.890	1383	308	0.028
2	5.050	2703066	1309384	54.573
3	12.331	168571	11420	3.403
4	19.447	22452	3198	0.453
5	26.046	2057608	69353	41.542
All		4953080	1393663	

Figure S6. The GC chart of the crude reaction mixture by CuCl catalysis(Table S1, entry 1)

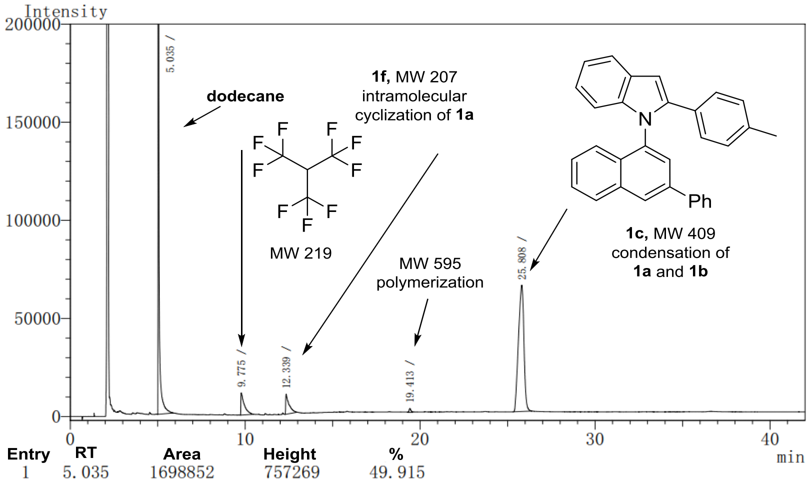

Entry	RT	Area	Height	%
1	5.035	1698852	757269	49.915
2	9.775	154726	11353	4.546
3	12.339	134519	10073	3.952
4	19.413	15717	2005	0.462
5	25.808	1399688	64367	41.125
All		3403502	845067	

Figure S7. The GC chart of the crude reaction mixture by CuBr catalysis(Table S1, entry 2)

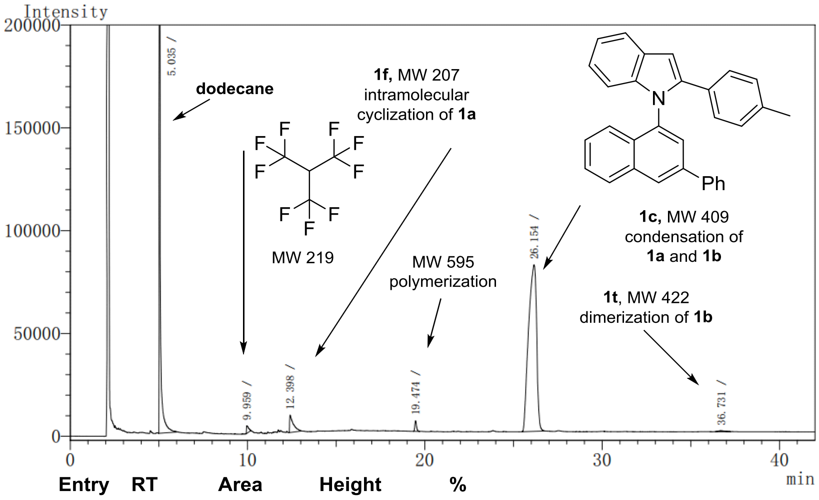

Entry	RT	Area	Height	%
1	5.035	2734359	1325619	49.800
2	9.959	30100	3691	0.548
3	12.398	123736	8519	2.254
4	19.474	34105	5144	0.621
5	26.154	2553583	80966	46.508
6	36.731	14787	468	0.269
All		5490670	1424407	

Figure S8. The GC chart of the crude reaction mixture by CuOAc catalysis (Table S1, entry 3)

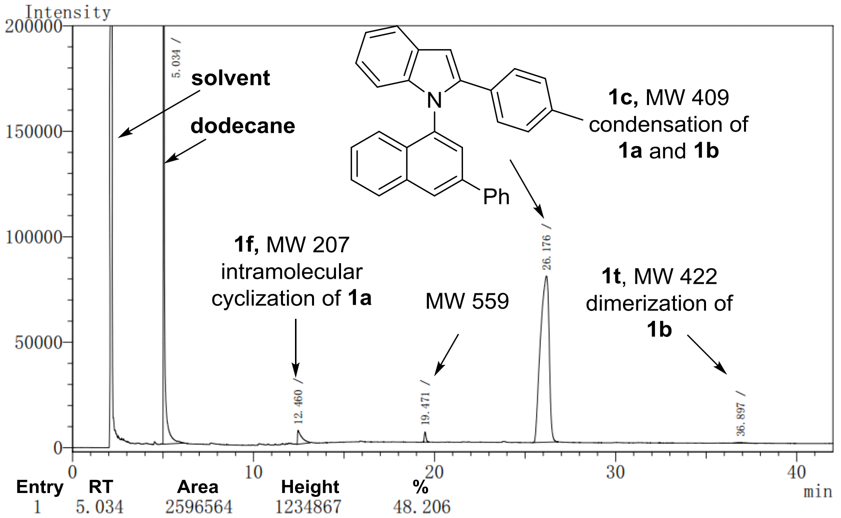

Entry	RT	Area	Height	%
1	5.034	2596564	1234867	48.206
2	12.460	107435	6594	1.995
3	19.471	32742	4870	0.608
4	26.176	2637495	78810	48.966
5	36.897	12176	310	0.226
All		5386412	1325451	

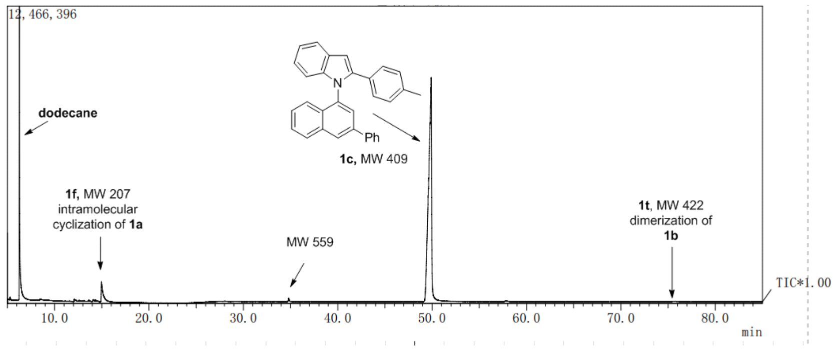

Figure S9. The GC chart of the crude reaction mixture by CuI catalysis (Table S1, entry 4)

Table S2. Solvent Screening

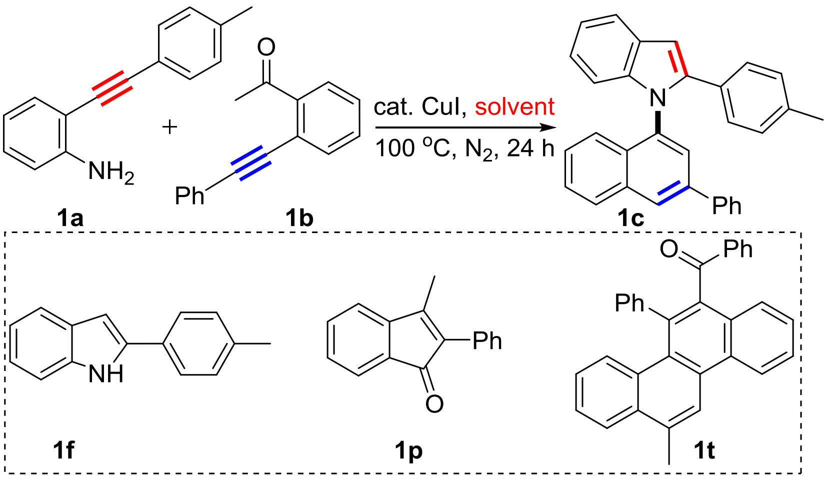

entry	solvent	conversion [%][b]		yield [%][b]			
		1a	1b	1c	1f	1p	1t
**1**	**HFIP**	**>99**	**>99**	**86**	**11**	**trace**	**trace**
2	TFE	>99	>99	60	19	trace	trace
3	TFP	>84	>99	trace	30	trace	11
4	TFB	>90	>99	5	32	trace	14
5	PFTB	>99	>99	66	23	trace	trace
6	MeOH	>50	>99	10	23	trace	5
7	EtOH	>76	>99	4	38	trace	11
8	Propanol	>65	>99	trace	35	trace	6
9	IPA	>83	>99	10	40	trace	7
10	TBA	>80	>99	8	38	trace	trace
11	Toluene	>50	>30	trace	47	trace	trace
12	THF	>73	>37	10	38	trace	trace
13	DMF	>99	>99	trace	55	trace	8
14	DCM	>60	>99	19	33	trace	trace

[a]Reactions:**1a** (0.05 mmol), **1b** (0.05 mmol), CuI (10 mol %), in Solvent (0.5 mL) at 100 °C under N_2 for 24 h.

[b]GC yield using dodecane as an internal standard.

[c]TFE (2,2,2-Trifluoroethanol); TFP (3,3,3-Trifluoro-1-propanol); TFB (4,4,4-Trifluorobutanol); PFTB (Perfluoro-tert-butyl Alcohol); IPA(iso-Propyl alcohol); TBA (Tert- butyl alcohol).

*Notes: Many byproducts have been detected, and different solvents gave different types and selectivities of products (Figures S10-S12). The products of the intramolecular cyclization product (**1f**) of **1a**, intramolecular cyclization (**1p**) of **1b**, dimerization (**1t**) of **1b** were selected to be shown in Table S2.*

Selected GC and/or GC-MS charts of the reactions in different solvents

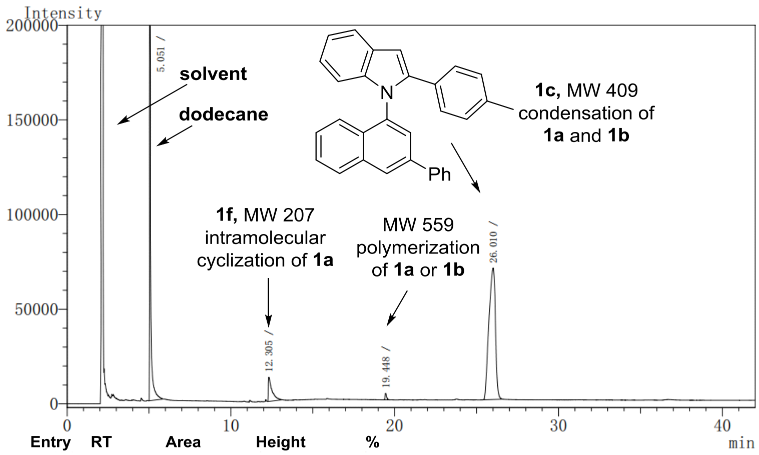

Entry	RT	Area	Height	%
1	5.051	2706269	1318444	55.927
2	12.305	194921	12722	4.028
3	19.448	24293	3498	0.502
4	26.010	1913476	69502	39.543
All		4838959	1404166	

Figure S10. The GC chart of the crude reaction mixture in TFE (Table S2, entry 2)

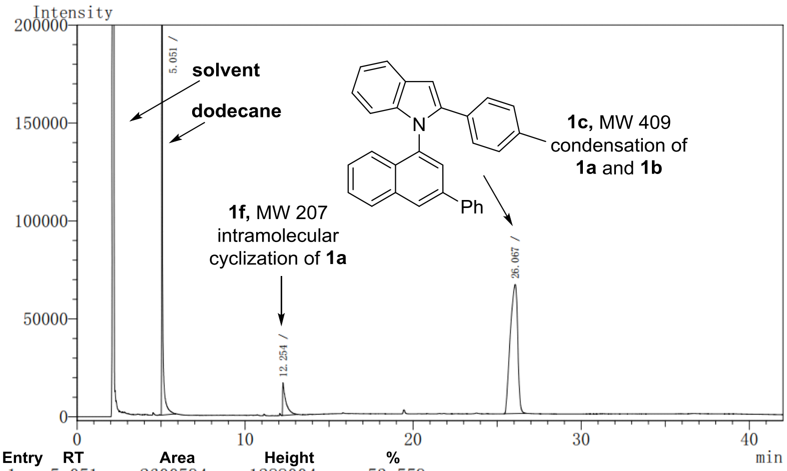

Entry	RT	Area	Height	%
1	5.051	2600594	1288004	53.559
2	12.254	225600	16733	4.646
3	26.067	2029351	65934	41.795
All		4855545	1370671	

Figure S11. The GC chart of the crude reaction mixture in PFTB (Table S2, entry 5)

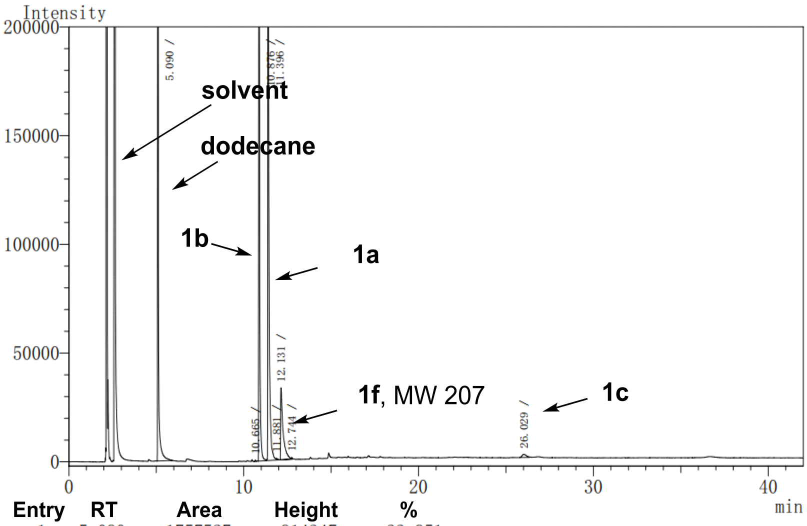

Entry	RT	Area	Height	%
1	5.090	1757527	914347	33.951
2	10.665	1932	590	0.037
3	10.876	1400481	540286	27.054
4	11.396	1677033	561937	32.396
5	11.881	1011	282	0.020
6	12.131	310976	33127	6.007
7	12.744	3372	791	0.065
8	26.029	24277	1384	0.469
All		5176609	2052744	

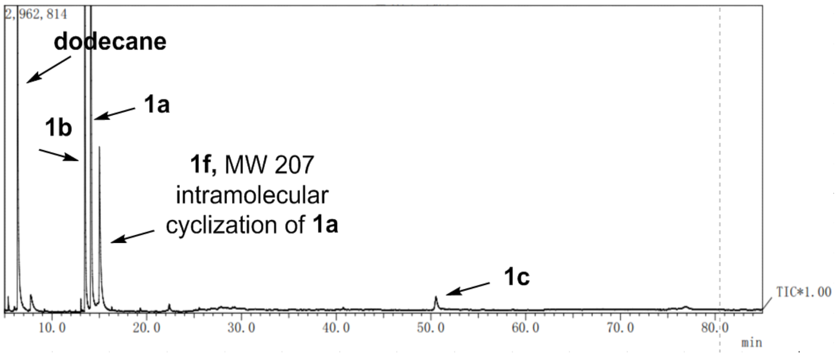

Figure S12. The GC and GC-MS charts of the crude reaction mixture in Toluene (Table S2, entry 11)

Table S3. Temperature Screening

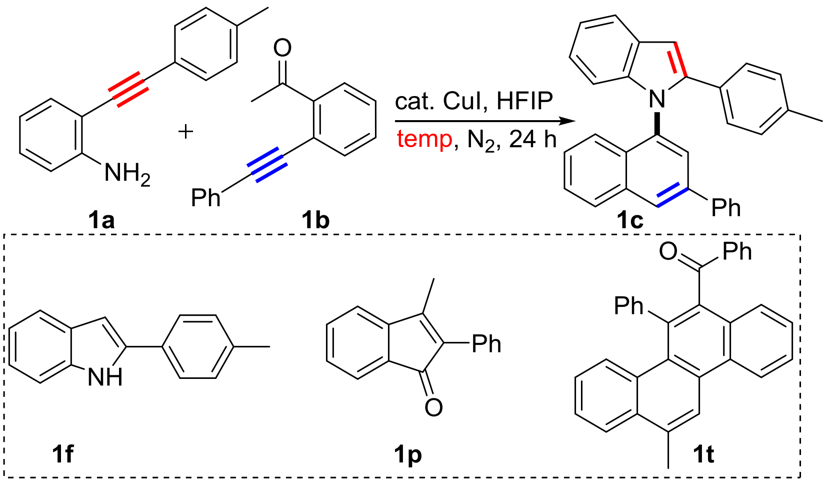

Entry	T(°C)	conversion [%][b]		yield [%][b]			
		**1a**	**1b**	**1c**	**1f**	**1p**	**1t**
**1**	**100**	**>99**	**>99**	**86**	**11**	**trace**	**trace**
2	60	>76	>99	53	trace	trace	trace
3	80	>99	>99	75	15	trace	trace
4	120	>99	>99	82	17	trace	trace
5	140	>99	>99	78	20	trace	trace

[a]Reactions:**1a** (0.05 mmol), **1b** (0.05 mmol), CuI (10 mol %), in HFIP (0.5 mL) at 100 °C under N_2 for 24 h.

[b]GC yield using tridecane as an internal standard.

*Notes: Different temperatures gave different selectivities of the product (Figures S13-14). The products of the intramolecular cyclization product (**1f**) of **1a**, intramolecular cyclization (**1p**) of **1b**, dimerization (**1t**) of **1b** were selected to be shown in Table S3.*

Selected GC charts of the reactions at different temperatures

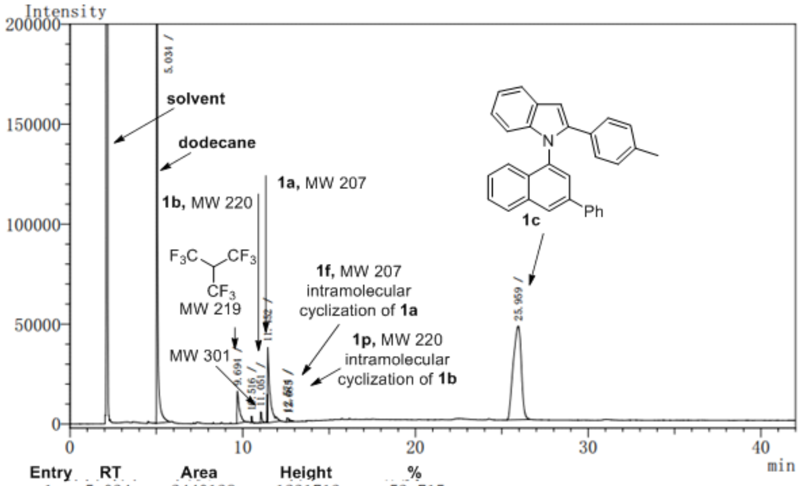

Entry	RT	Area	Height	%
1	5.034	2440138	1231713	53.715
2	9.694	152375	16105	3.354
3	10.516	11866	3338	0.261
4	11.051	28714	5523	0.632
5	11.452	359534	37634	7.914
6	12.574	13392	1991	0.295
7	12.685	10515	1386	0.231
8	25.959	1526247	46855	33.597
All		4542781	1344545	

Figure S13. The GC chart of the crude reaction mixture at 60 °C (Table S3, entry 2)

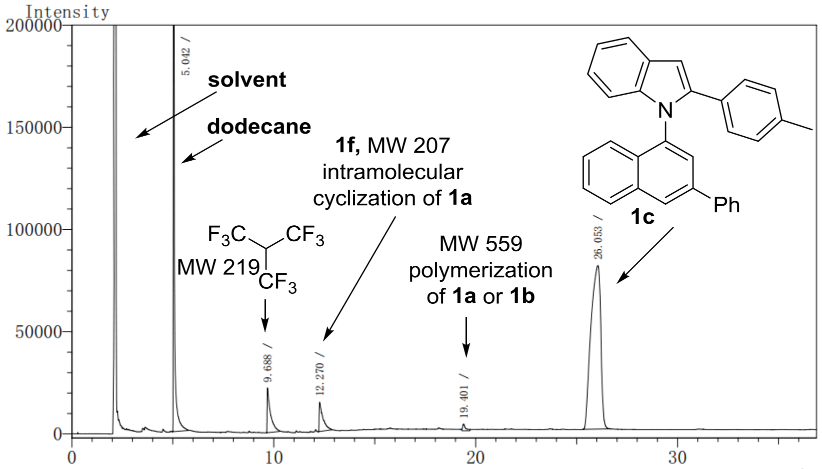

Entry	RT	Area	Height	%
1	5.042	2767687	1384802	46.642
2	9.688	247905	21877	4.178
3	12.270	182713	14362	3.079
4	19.401	34824	3326	0.587
5	26.053	2700772	80016	45.514
All		5933901	1504383	

Figure S14. The GC chart of the crude reaction mixture at 120 °C (Table S3, entry 4)

Table S4. Ligand Screening

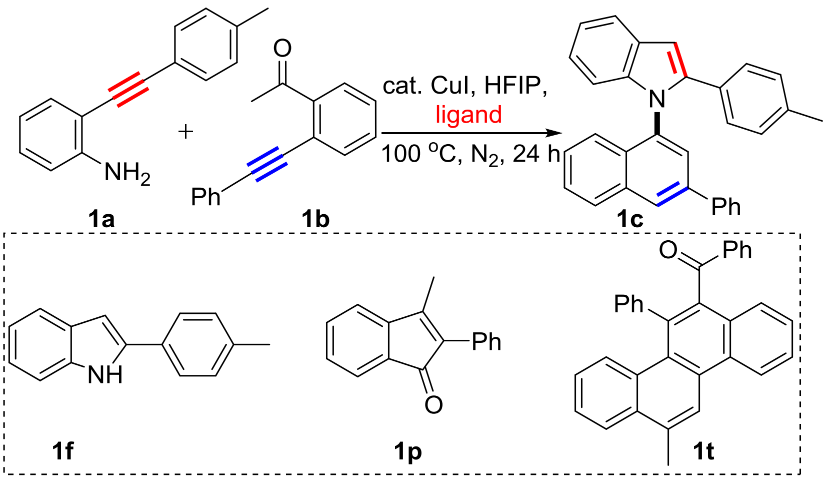

Entry	Ligand	conversion [%][b]		yield [%][b]			
		**1a**	**1b**	**1c**	**1f**	**1p**	**1t**
1	/	**>99**	**>99**	**86**	**11**	**trace**	**trace**
2	PHEN	>92	>99	74	16	trace	trace
3	MEDA	>99	>99	78	18	trace	trace
4	DPPF	>99	>99	79	20	trace	trace
5	BINAP	>99	>99	83	14	trace	trace

[a]Reactions:**1a** (0.05 mmol), **1b** (0.05 mmol), CuI (10 mol %), in HFIP (0.5 mL) at 100 °C under N_2 for 24 h.

[b]GC yield using tridecane as an internal standard.

Notes: Additions of the ligands did not improve the yield of the desired products.

Table S5. Additive Screening

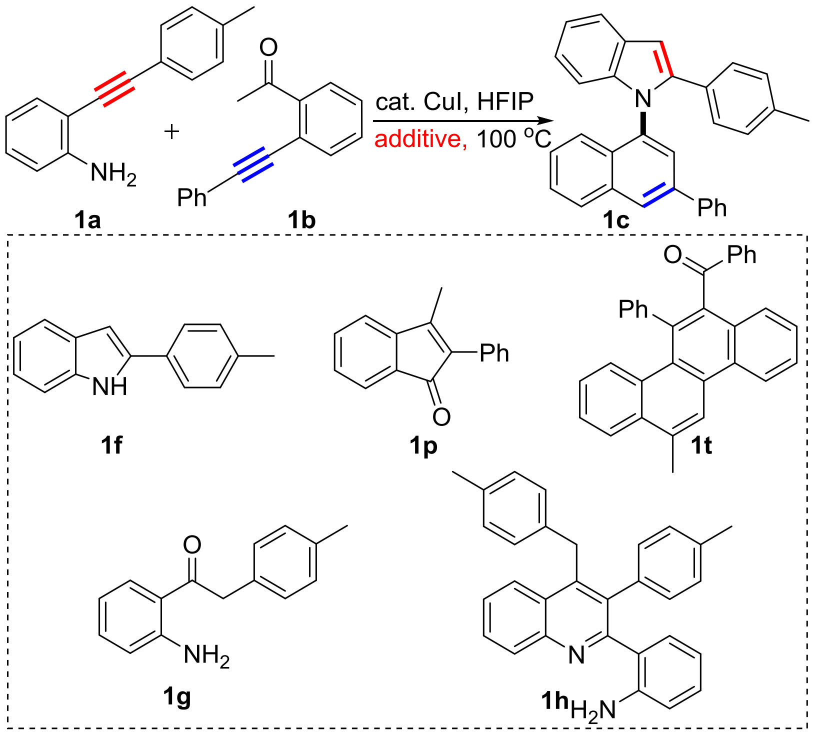

Entry	Additive	Conversion [%][b]		yield [%][b]					
		**1a**	**1b**	**1c**	**1f**	**1p**	**1t**	**1g**	**1h**
1	/	>99	>99	86	11	trace	trace	nd	nd
2	TsOH	>99	>99	trace	31	trace	5	27	10
3	HOAc	>99	>99	trace	25	trace	trace	15	trace
4	Na_2CO_3	>99	>99	84	11	trace	trace	nd	nd
5	K_3PO_4	>99	>99	87	9	trace	trace	nd	nd
6	*t*-BuOK	>99	>99	88	7	trace	trace	nd	nd
**7**	**Cs_2CO_3**	**>99**	**>99**	**91**	**5**	**trace**	**nd**	**nd**	**nd**
8[c]	Cs_2CO_3	>99	>99	88	8	trace	nd	nd	nd
9[d]	Cs_2CO_3	>99	>99	92	6	trace	trace	nd	nd

[a]Reactions:**1a** (0.05 mmol), **1b** (0.05 mmol), CuI (10 mol %), additive (2.0 equiv) in HFIP (0.5 mL) at 100 °C under N_2 for 24 h. [b]Conversion and GC yield using dodecane as an internal standard. [c]additive (1.0 equiv). [d]additive (3.0 equiv).

*Notes: Many byproducts have been detected, and different additives gave different types and selectivities of products (Figures S15-S16). The byproducts of the intramolecular cyclization product (**1f**) of **1a**, intramolecular cyclization (**1p**) of **1b**, dimerization (**1t**) of **1b** were selected to be shown in Table S5.*

Selected GC and GC-MS charts of the reaction in the presence of different additives

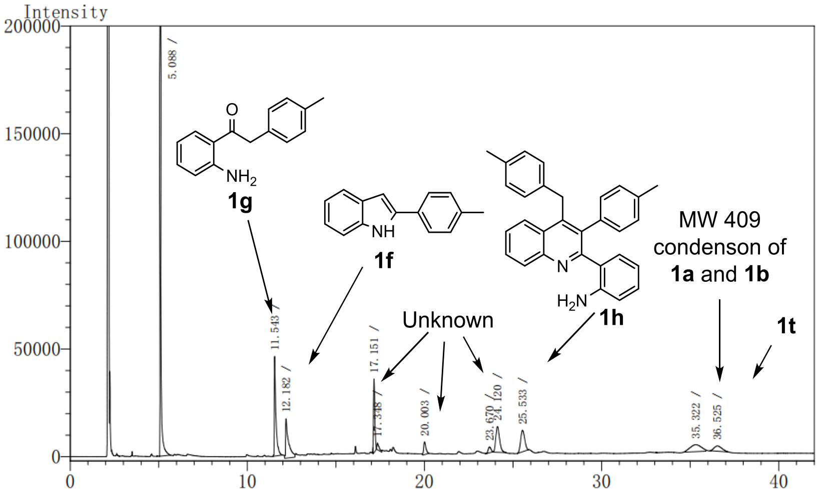

Entry	RT	Area	Height	%
1	5.088	1773920	927398	71.371
2	11.543	270598	46171	10.887
3	12.182	205524	18344	8.269
4	25.533	158456	9933	6.375
5	36.525	76991	2470	3.098
All		2485489	1004316	

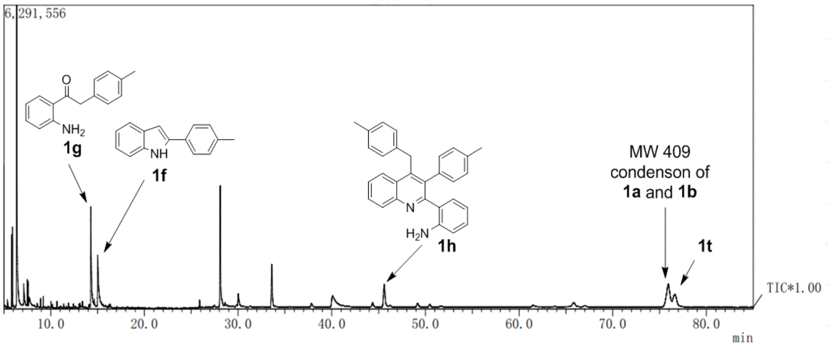

Figure S15. The GC and GC-MS charts of the crude reaction mixture in the presence of TsOH (Table S5, entry 2)

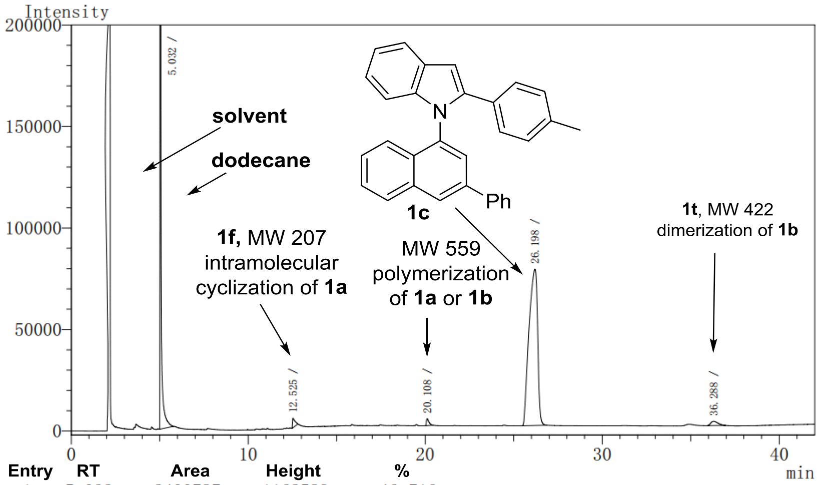

Entry	RT	Area	Height	%
1	5. 032	2400787	1162589	46. 716
2	12. 525	45177	4623	0. 879
3	20. 108	34666	3499	0. 675
4	26. 198	2598497	77002	50. 563
5	36. 288	59989	2139	1. 167
All		5139116	1249852	

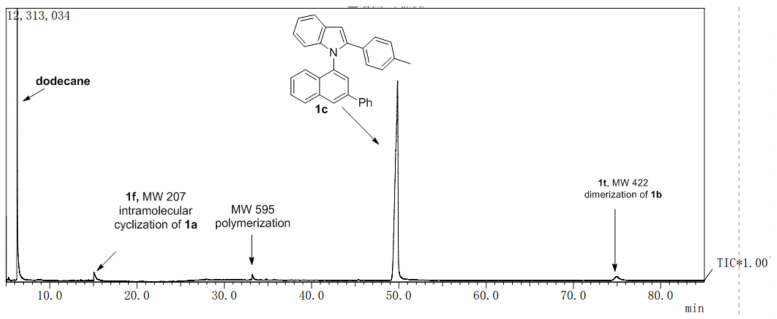

Figure S16. The GC and GC-MS charts of the crude reaction mixture in the presence of (2.0 equiv) Cs_2CO_3 as additive (Table S5, entry 7)

Table S6. Ratio of 1a and 1b Screening

Entry	**1a** (mmol)	**1b** (mmol)	conversion [%][b]		yield [%][b]			
			**1a**	**1b**	**1c**	**1f**	**1p**	**1t**
1	0.12	0.10	>99	>99	85	10	trace	nd
2	0.10	0.10	>99	>99	91	5	trace	nd
3	0.10	0.11	>99	>99	92	7	trace	nd
4	0.10	0.12	>99	>99	94	5	2	nd
5	0.10	0.13	>99	>99	91	7	6	nd

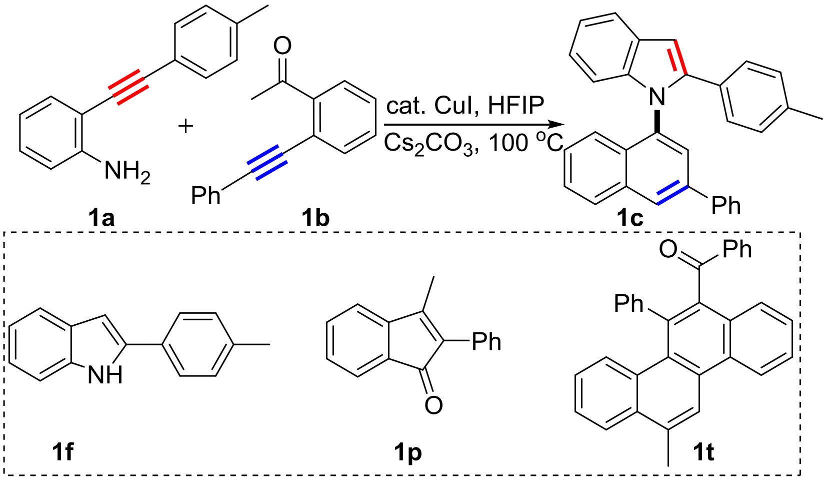

[a]Reactions:**1a**, **1b**, catalyst (10 mol %), Cs_2CO_3(2.0 equiv) in HFIP (0.5 mL) at 100 °C under N_2 for 24 h.

[b]Conversion and GC yield using dodecane as an internal standard.

*Notes: The different ratio of **1a** and **1b** gave different selectivities of products. The byproducts of the intramolecular cyclization product (**1f**) of **1a**, intramolecular cyclization (**1p**) of **1b**, dimerization (**1t**) of **1b** were selected to be shown in Table S6.*

Scheme S2. The optimal reaction conditions

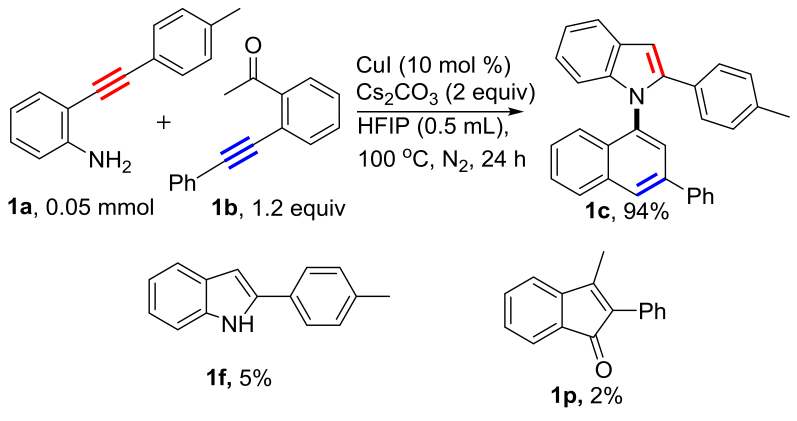

Optimal reaction conditions: treatment with 2-(p-tolylethynyl)aniline (1a, 0.05 mmol) and 2'-phenylethynylacetophenone (1b, 1.2 equiv) in the presence of CuI (10 mol %), and Cs_2CO_3 (2 equiv) in 1,1,1,3,3,3-hexafluoropropan-2-ol (HFIP) (0.5 mL) at 100 °C for 24 h.

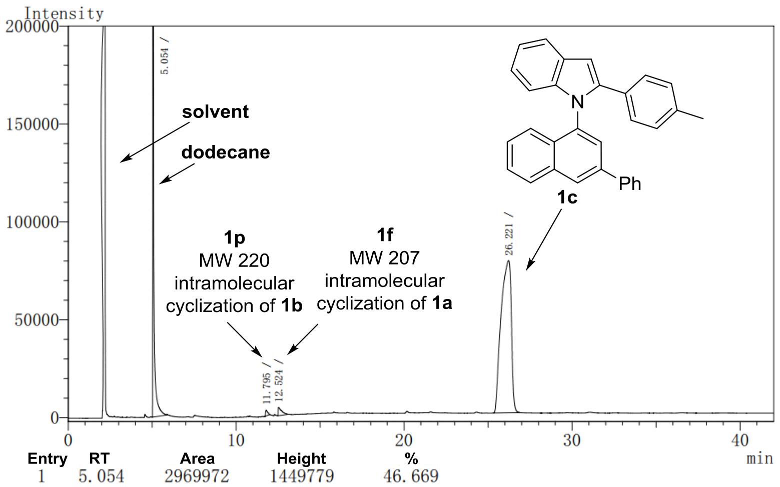

Entry	RT	Area	Height	%
1	5.054	2969972	1449779	46.669
2	11.795	24087	2808	0.378
3	12.524	63837	4290	1.003
4	26.221	3306016	77733	51.949
All		6363912	1534610	

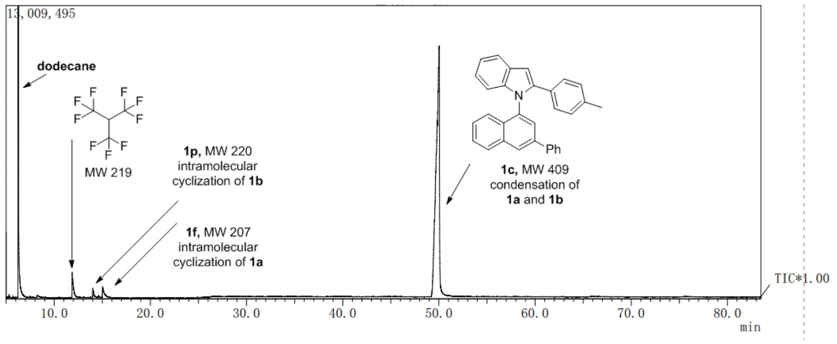

Figure S17. The GC and GC-MS charts of the crude reaction mixture under the optimal reaction conditions (Table S6, entry 4)

3.2. GC, GC-MS Charts and NMR Spectrum of 1a, 1b, and Byproducts

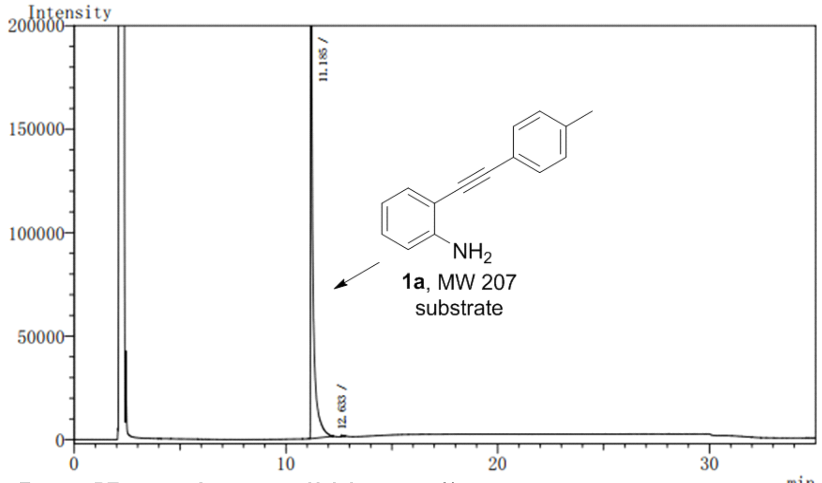

Entry	RT	Area	Height	%
1	11.185	2534313	683527	99.776
2	12.633	5699	723	0.224
All		2540012	684250	

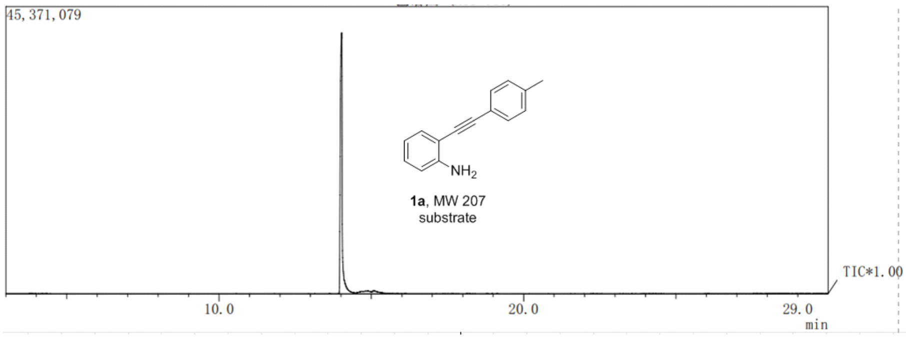

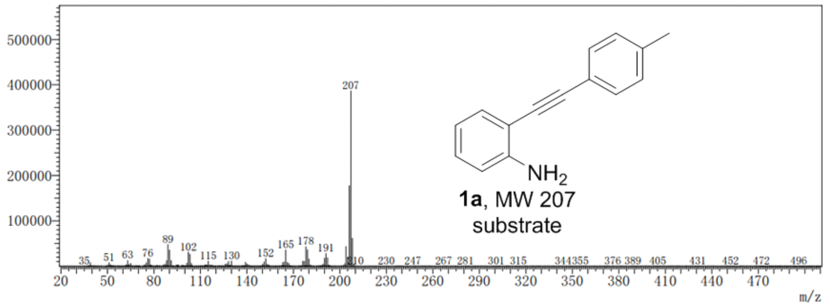

Figure S18. The GC and GC-MS charts 1a.

2-(p-tolylethynyl)aniline (**1a**)

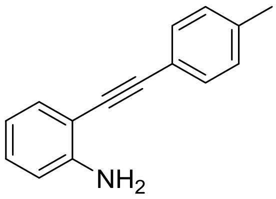

^{1}H NMR (400 MHz, $CDCl_3$) δ 7.39 (dd, *J* = 25.4, 7.2 Hz, 3H), 7.14 (dd, *J* = 14.5, 7.4 Hz, 3H), 6.71 (t, *J* = 7.2 Hz, 2H), 4.26 (s, 2H), 2.37 (s, 3H).

^{1}H NMR Spectrum of **1a**

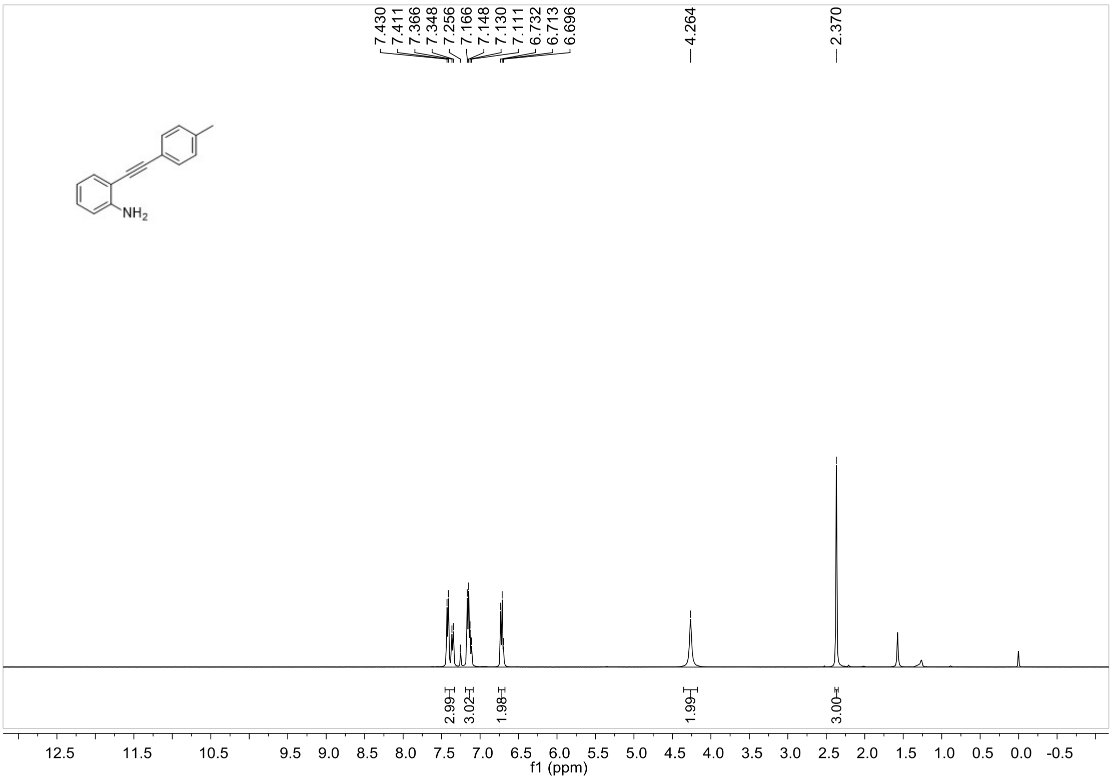

Figure S19. The ^{1}H and ^{13}C NMR Spectrum of 1a.

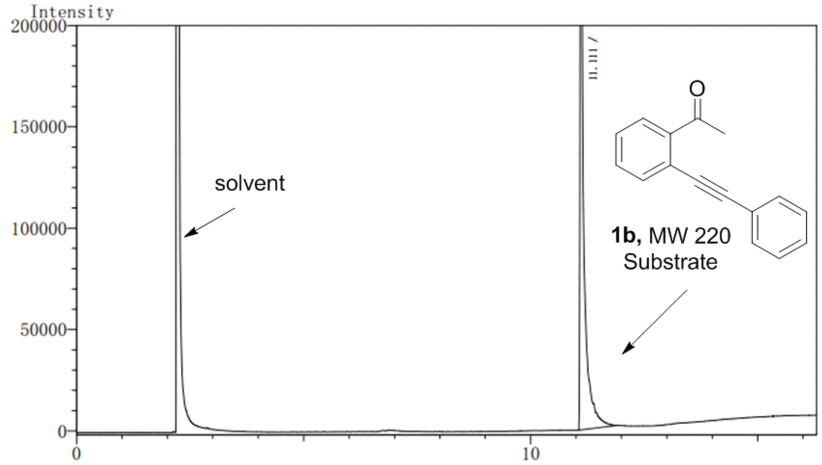

Entry	RT	Area	Height	%
1	11.111	1996446	523523	100.000
All		1996446	523523	

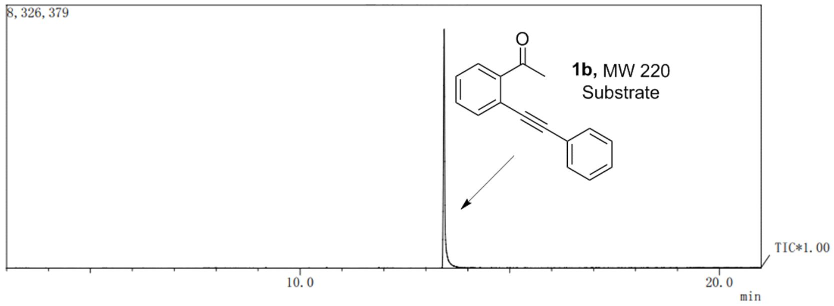

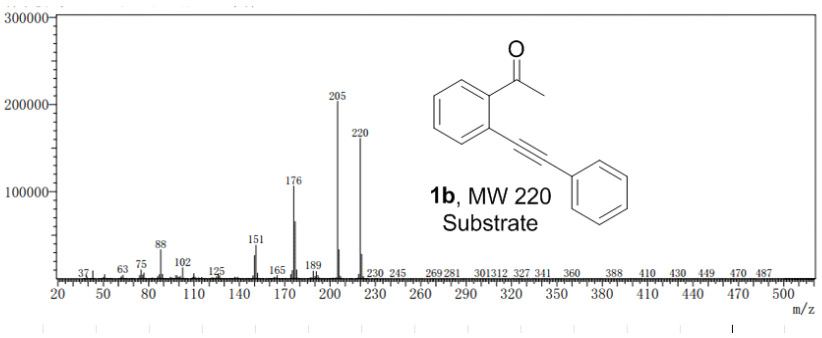

Figure S20. The GC and GC-MS charts of 1b.

1-(2-(phenylethynyl)phenyl)ethan-1-one (**1b**)

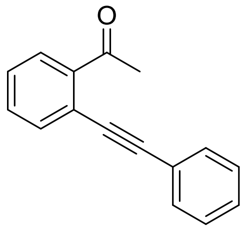

^{1}H NMR (400 MHz, $CDCl_3$) δ 7.75 (dd, *J* = 7.8, 1.0 Hz, 1H), 7.63 (dd, *J* = 7.7, 0.8 Hz, 1H), 7.57 – 7.52 (m, 2H), 7.47 (td, *J* = 7.5, 1.2 Hz, 1H), 7.43–7.34 (m, 4H), 2.79 (s, 3H).

^{1}H NMR Spectrum of **1b**

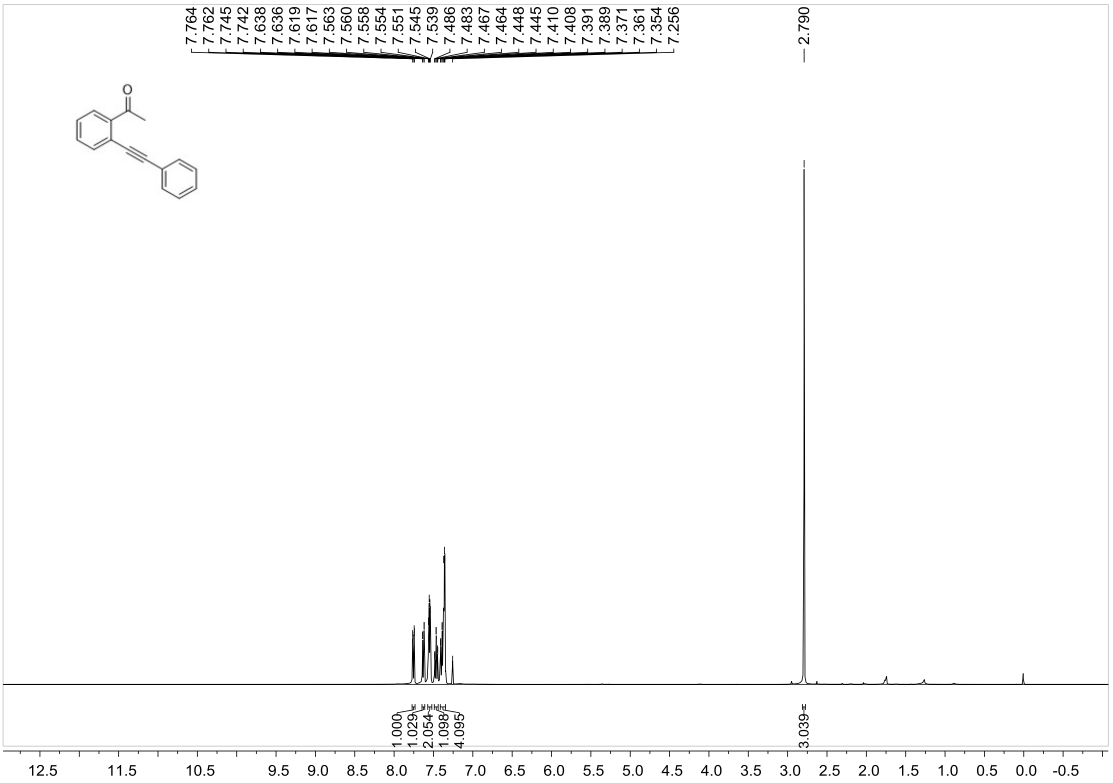

Figure S21. The ^{1}H and ^{13}C NMR Spectrum of 1b.

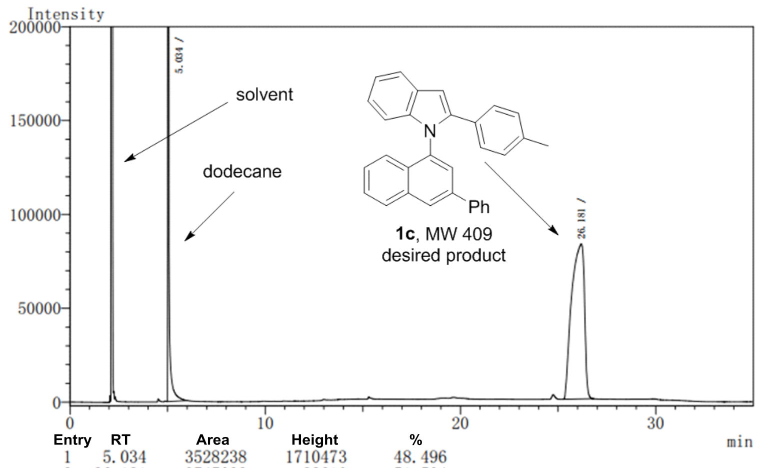

Entry	RT	Area	Height	%
1	5.034	3528238	1710473	48.496
2	26.181	3747032	82610	51.504
All		7275270	1793083	

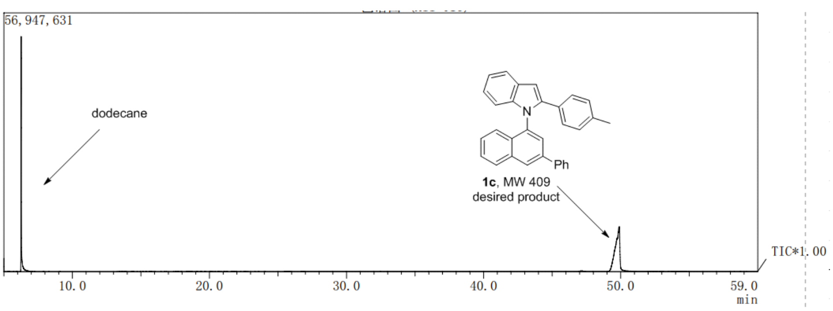

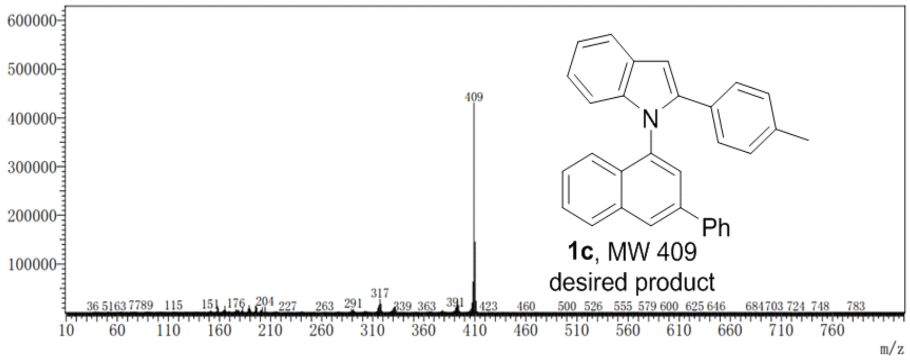

Figure S22. The GC and GC-MS charts of 1c

1-(3-phenylnaphthalen-1-yl)-2-(p-tolyl)-1H-indole (1c)

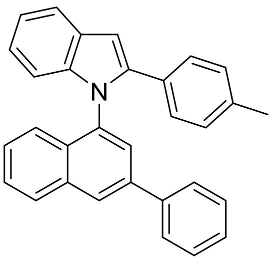

The title compound was prepared according to the general procedure and purified by column chromatography on silica gel and eluted with petroleum ether/dichloromethane (10/1) to afford a white powder in 94% yield (76.9 mg). mp 192–193 °C. ^{1}H NMR (400 MHz, $CDCl_3$) δ 8.19 (s, 1H), 8.04 (d, *J* = 8.2 Hz, 1H), 7.83 (d, *J* = 7.8 Hz, 1H), 7.75–7.71 (m, 1H), 7.68 (d, *J* = 7.5 Hz, 2H), 7.57 (t, *J* = 7.5 Hz, 1H), 7.51 (t, *J* = 7.9 Hz, 3H), 7.42 (t, *J* = 7.4 Hz, 2H), 7.30–7.21 (m, 3H), 7.16 (t, *J* = 7.6 Hz, 1H), 7.01-6.94 (m, 4H), 2.26 (s, 3H). ^{13}C NMR (101 MHz, $CDCl_3$) δ 142.2, 140.1, 139.9, 138.2, 137.1, 135.9, 134.5, 130.4, 129.5, 128.9, 128.8, 128.5, 128.3, 128.0, 127.6, 127.2, 127.0, 126.9, 126.7, 126.0, 123.5, 122.0, 120.5, 120.3, 111.1, 102.8, 21.0. HRMS (EI) m/z: $[M]^+$ calcd for $C_{31}H_{23}N$ 409.1830; found 409.1828.

^{1}H NMR Spectrum of **1c**

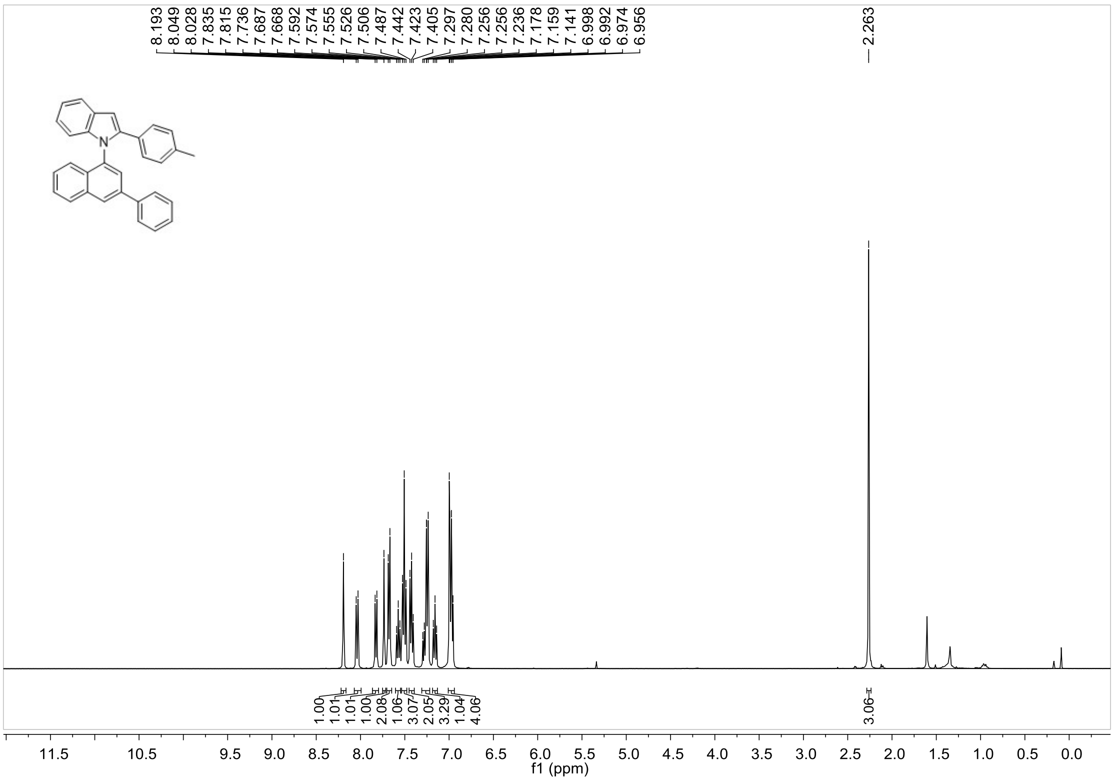

^{13}C NMR Spectrum of **1c**

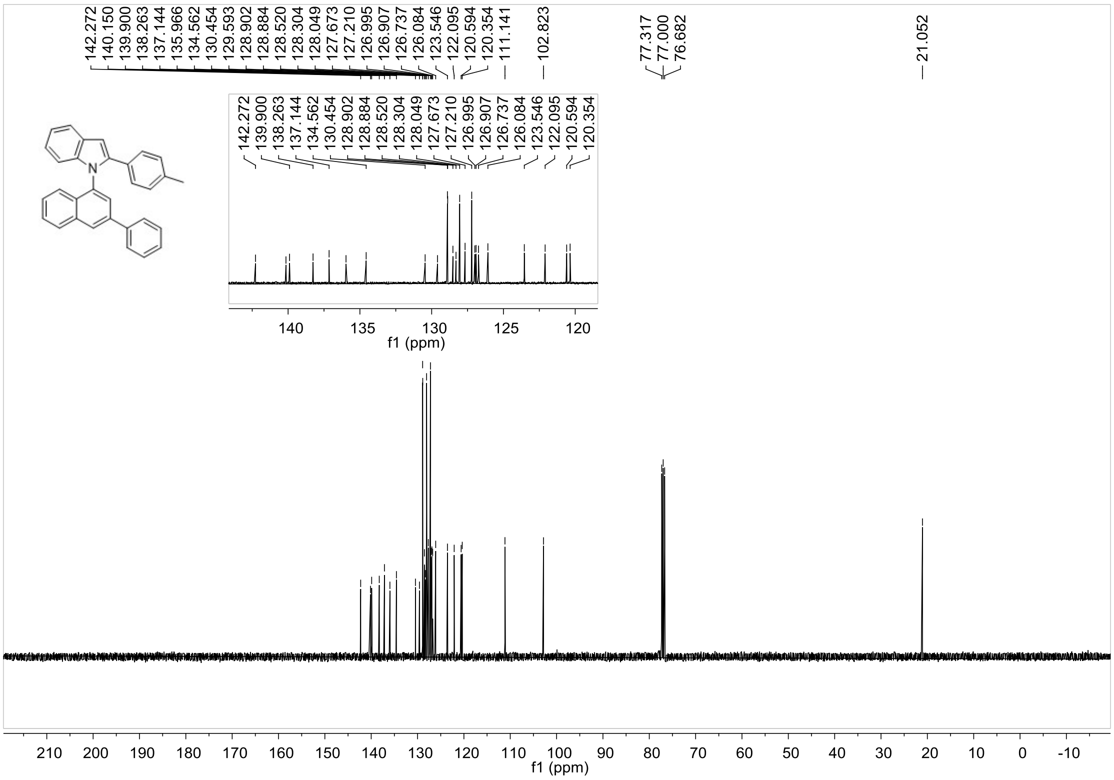

Figure S23. The ^{1}H and ^{13}C NMR Spectrum of 1c

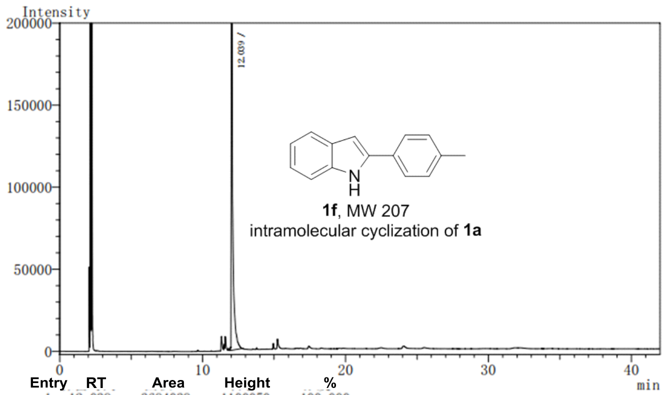

Entry	RT	Area	Height	%
1	12.039	3694039	1100050	100.000
All		3694039	1100050	

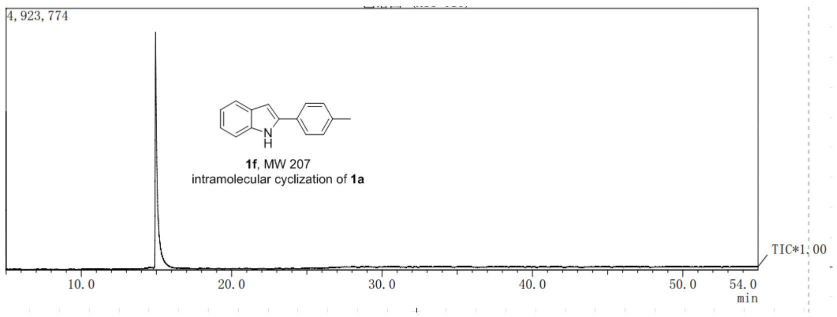

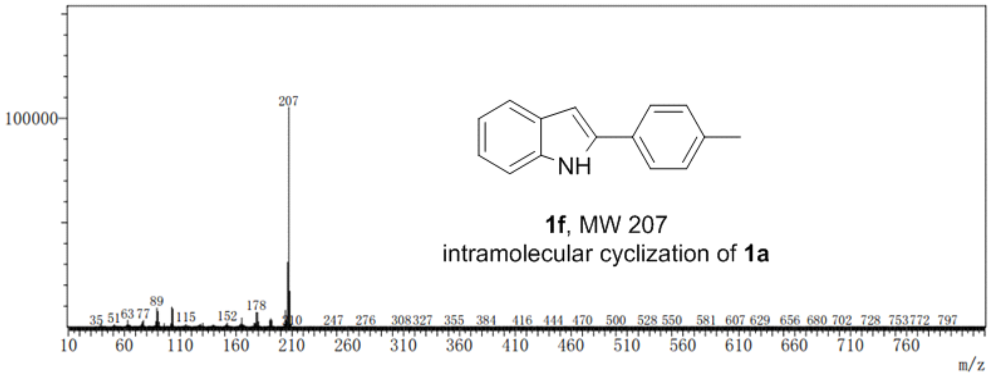

Figure S24. The GC and GC-MS charts of 1f

2-(p-tolyl)-1H-indole (1f)

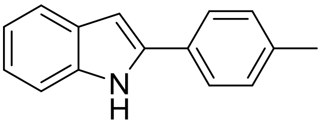

^{1}H NMR (400 MHz, $CDCl_3$) δ 8.32 (s, 1H), 7.64 (d, *J* = 7.7 Hz, 1H), 7.57 (d, *J* = 7.4 Hz, 2H), 7.39 (d, *J* = 7.9 Hz, 1H), 7.29–7.24 (m, 2H), 7.20 (t, *J* = 7.4 Hz, 1H), 7.14 (t, *J* = 7.3 Hz, 1H), 6.80 (s, 1H), 2.41 (s, 3H). ^{13}C NMR (101 MHz, $CDCl_3$) δ 138.0, 137.6, 136.6, 129.6, 129.5, 129.3, 125.0, 122.0, 120.4, 120.1, 110.7, 99.3, 21.2.

^{1}H NMR Spectrum of **1f**

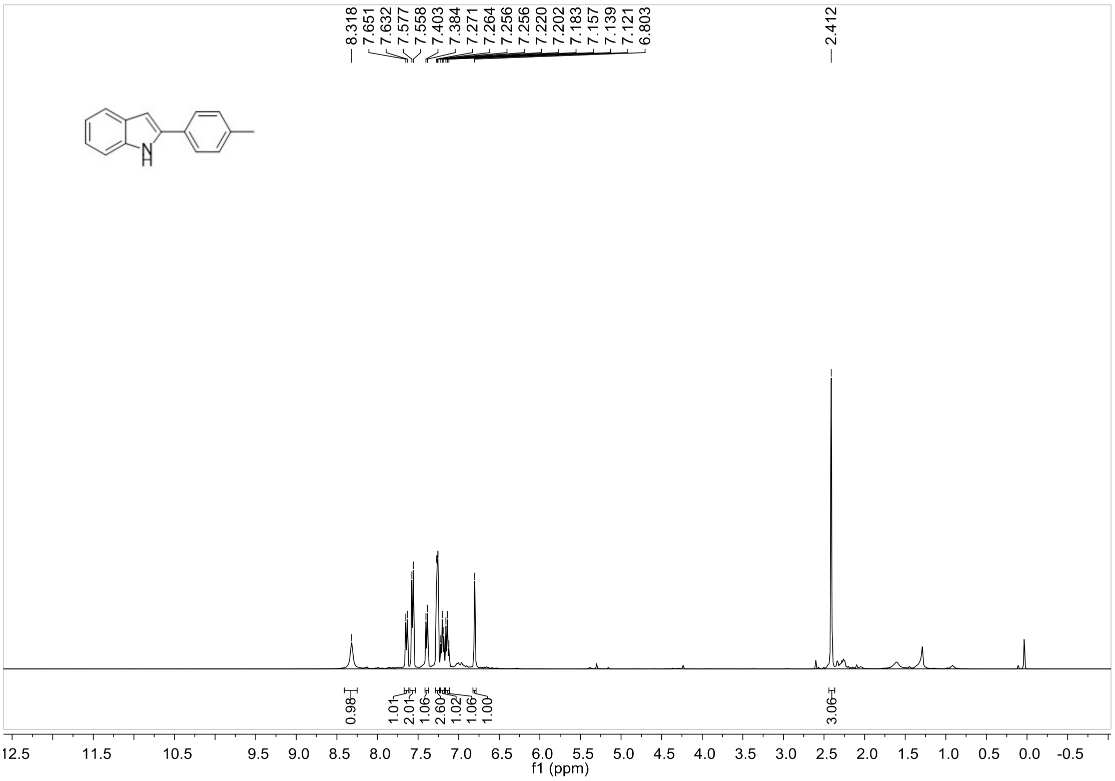

^{13}C NMR Spectrum of **1f**

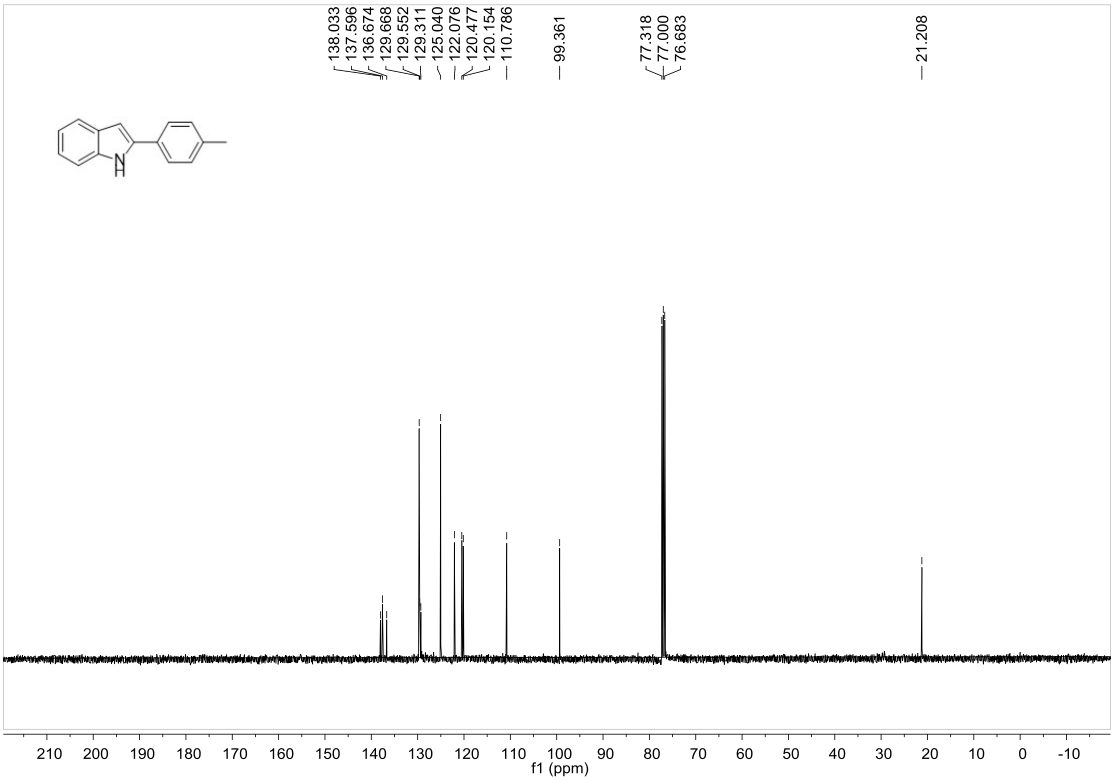

Figure S25. The ^{1}H and ^{13}C NMR Spectrum of 1f

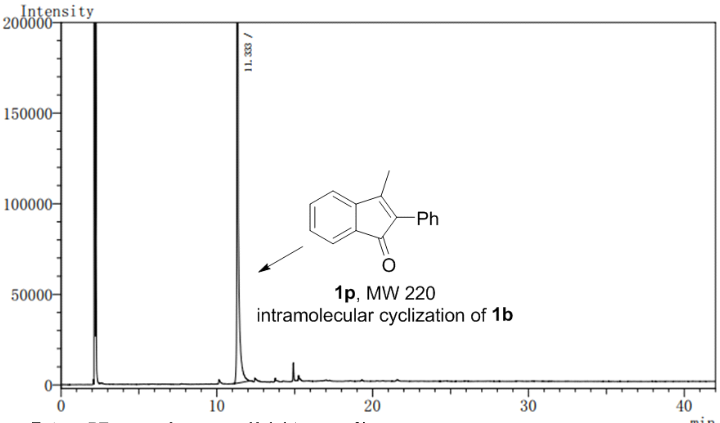

Entry	RT	Area	Height	%
1	11.333	4147642	1211905	100.000
All		4147642	1211905	

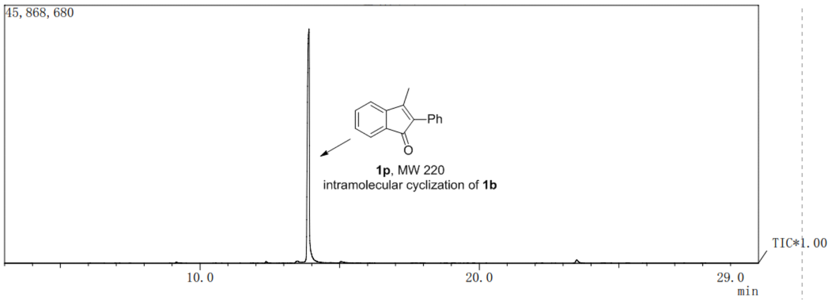

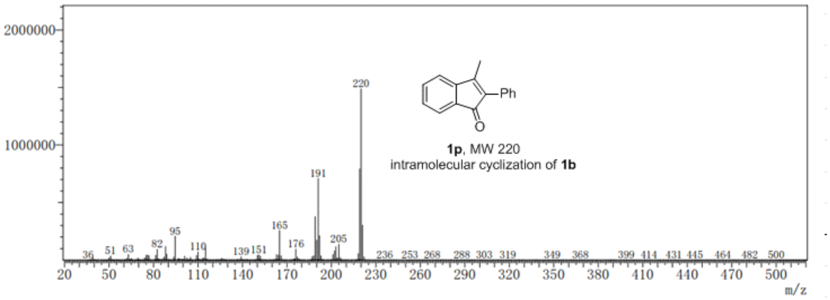

Figure S26. The GC and GC-MS charts of 1p

3-methyl-2-phenyl-1H-inden-1-one (1p)[15]

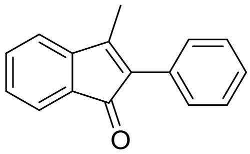

The title compound was prepared according to the procedure (Table S6, entry 5, six parallel experiments were conducted) and purified by column chromatography on silica gel and eluted with petroleum ether/dichloromethane (10/1) to afford a sticky yellow liquid. ^{1}H NMR (400 MHz, $CDCl_3$) δ 7.47 (d, J = 7.0 Hz, 1H), 7.44–7.36 (m, 5H), 7.35–7.30 (m, 1H), 7.27–7.20 (m, 1H), 7.14 (d, J = 7.1 Hz, 1H), 2.30 (s, 3H). ^{13}C NMR (101 MHz, $CDCl_3$) δ 196.3, 154.6, 145.8, 133.5, 133.4, 131.1, 130.3, 129.5, 128.8, 128.2, 127.6, 122.0, 119.3, 12.5.

^{1}H NMR Spectrum of **1p**

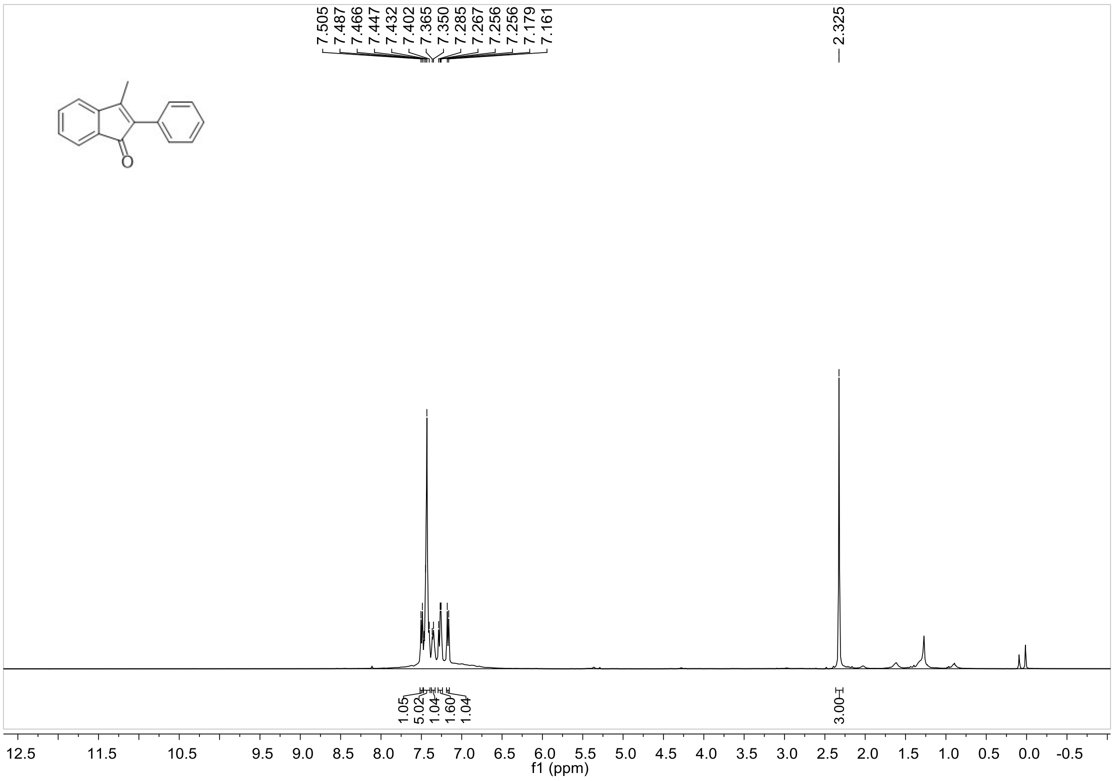

^{13}C NMR Spectrum of **1p**

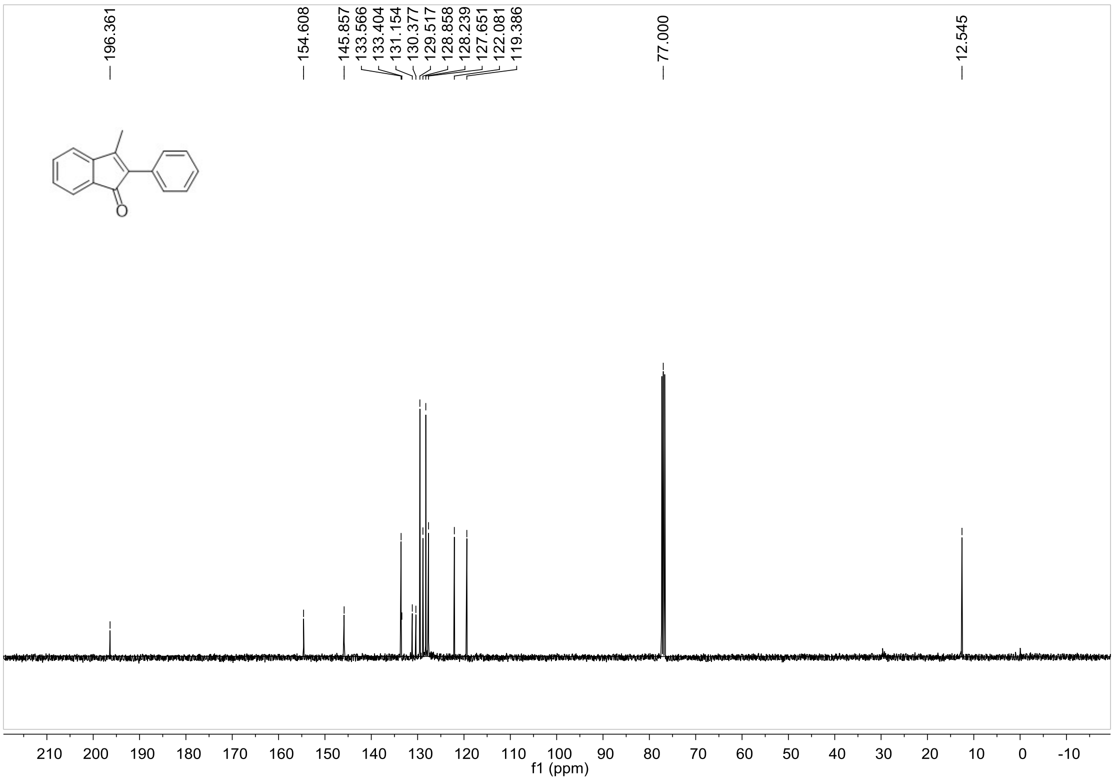

Figure S27. The ^{1}H and ^{13}C NMR Spectrum of 1p

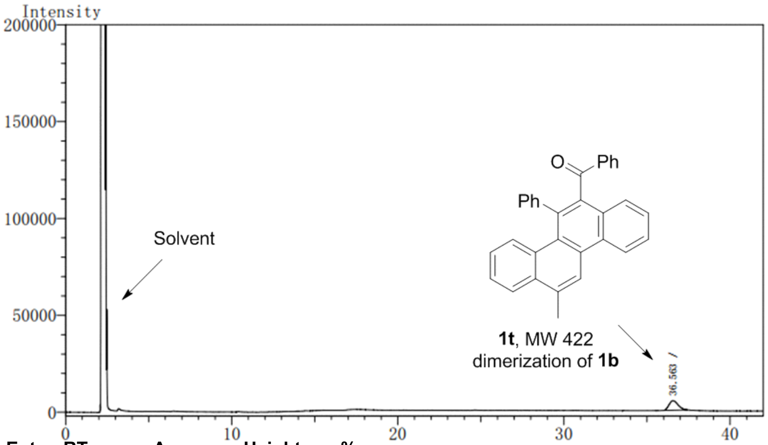

Entry	RT	Area	Height	%
1	36.563	186678	4970	100.000
All		186678	4970	

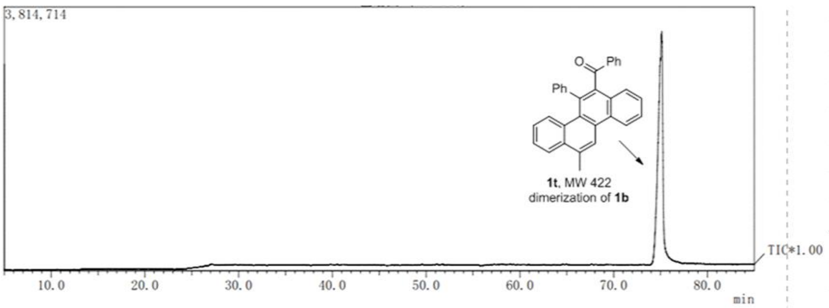

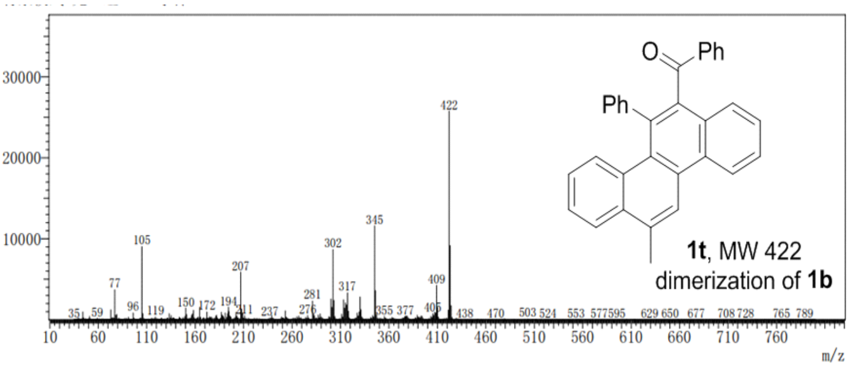

Figure S28. The GC and GC-MS charts of 1t

(12-methyl-5-phenylchrysen-6-yl)(phenyl)methanone (1t)

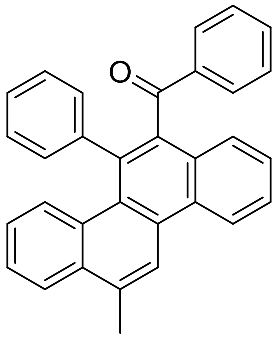

The title compound was prepared according to the procedure (Table S2, entry 4, six parallel experiments were conducted) and purified by column chromatography on silica gel and eluted with petroleum ether/dichloromethane (20/1) to afford a white solid. ^{1}H NMR (400 MHz, $CDCl_3$) δ 8.89 (d, *J* = 8.5 Hz, 1H), 8.68 (s, 1H), 8.10 (d, *J* = 8.2 Hz, 1H), 7.78 (d, *J* = 8.2 Hz, 1H), 7.76–7.70 (m, 2H), 7.65 (d, *J* = 7.5 Hz, 1H), 7.58–7.52 (m, 1H), 7.51–7.44 (m, 3H), 7.47 (td, *J* = 8.6, 1.1 Hz, 3H), 7.42 (t, *J* = 7.6 Hz, 1H), 7.35 (t, *J* = 7.4 Hz, 1H), 7.21–7.13 (m, 3H), 7.09–7.03 (m, 1H), 6.93 (t, *J* = 7.5 Hz, 1H), 6.77 (d, *J* = 7.7 Hz, 1H), 2.93 (s, 3H). ^{13}C NMR (101 MHz, $CDCl_3$) δ 200.0, 141.4, 137.9, 137.0, 134.8, 134.5, 133.1, 132.8, 131.4, 131.0, 130.3, 130.0, 129.3, 129.2, 129.0, 128.7, 128.7, 128.1, 128.0, 127.2, 127.2, 126.9, 126.0, 125.9, 125.8, 124.5, 124.1, 123.5, 121.4, 20.6.

^{1}H NMR Spectrum of **1t**

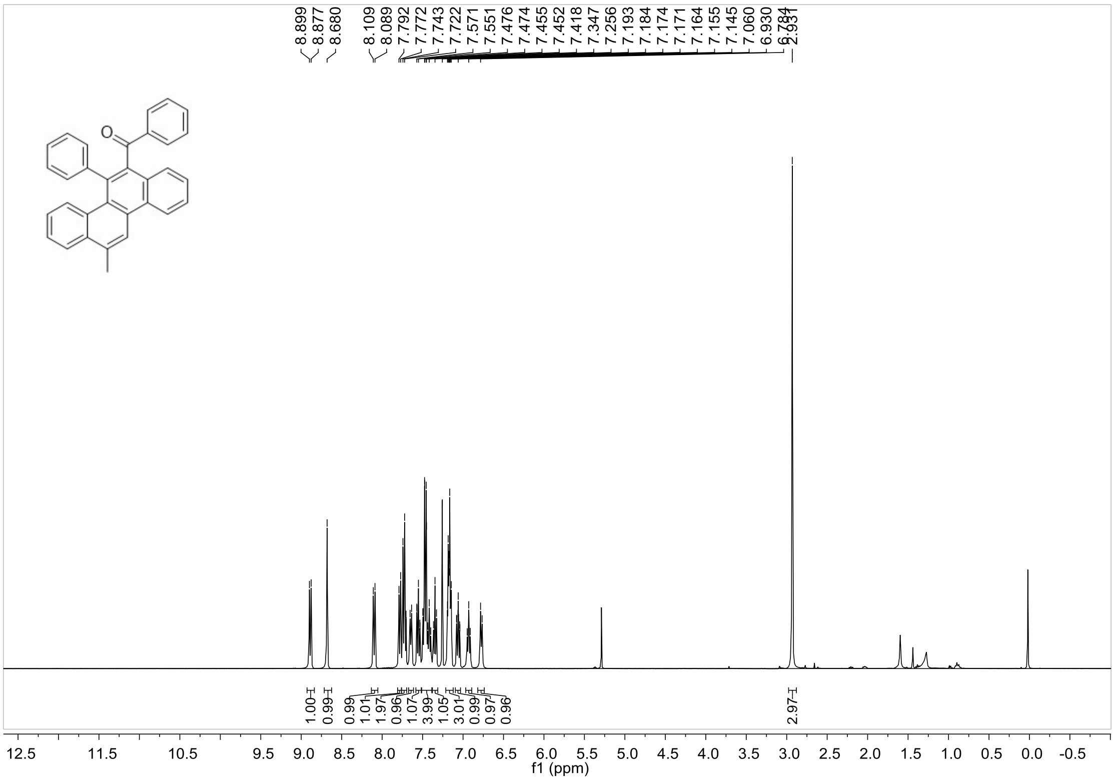

^{13}C NMR Spectrum of **1t**

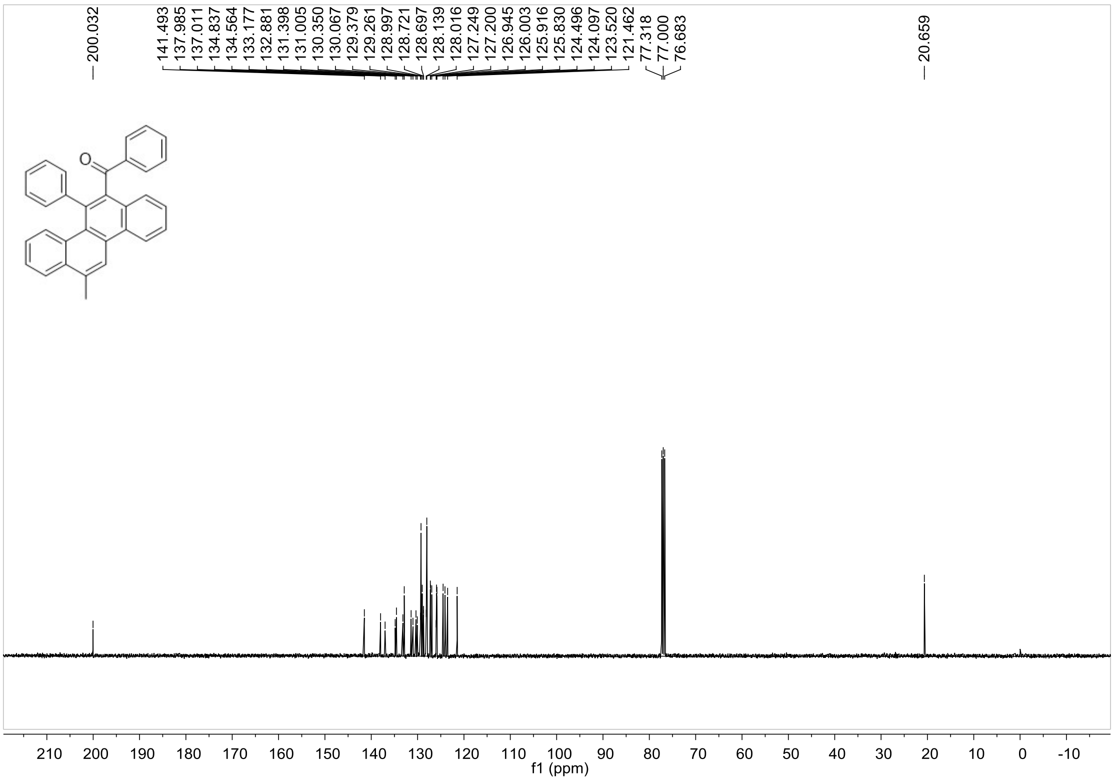

Figure S29. The ^{1}H and ^{13}C NMR Spectrum of 1t

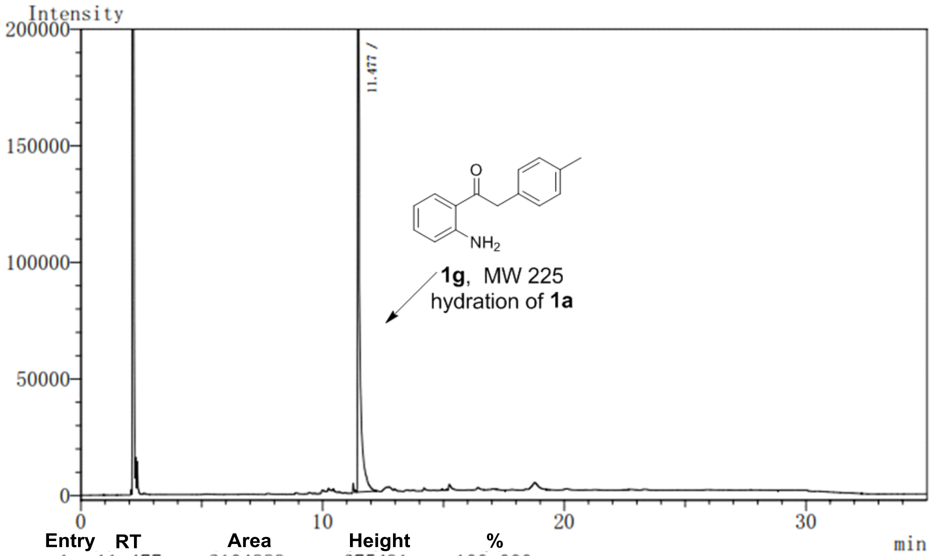

Entry	RT	Area	Height	%
1	11.477	2104889	675491	100.000
All		2104889	675491	

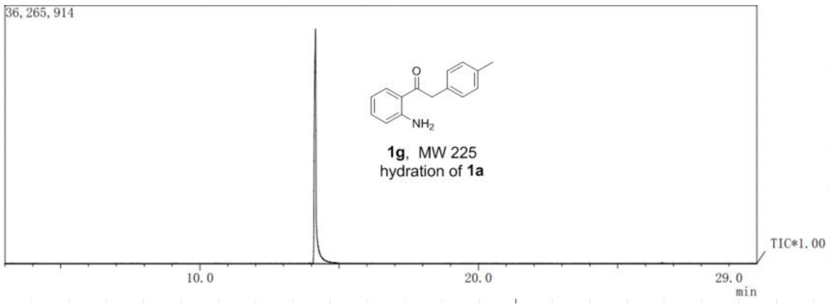

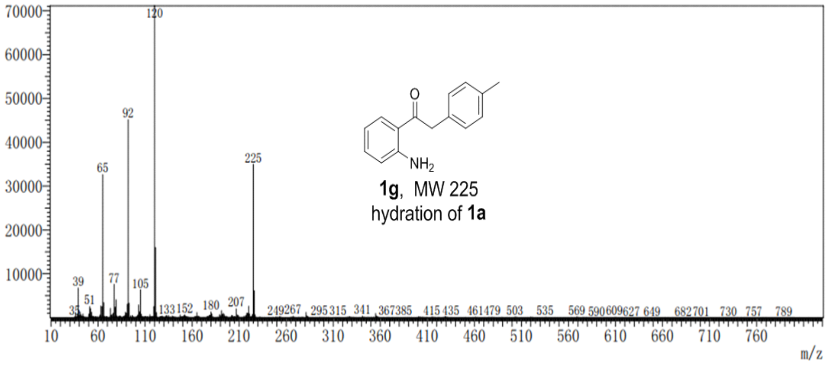

Figure S30. The GC and GC-MS charts of 1g

1-(2-aminophenyl)-2-(p-tolyl)ethan-1-one (1g)[16]

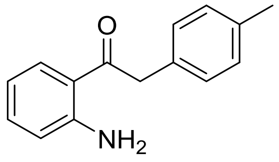

The title compound was prepared according to the procedure (Table S5, entry 2) and purified by column chromatography on silica gel and eluted with petroleum ether/dichloromethane(10/1) to afford a white solid. ^{1}H NMR (400 MHz, $CDCl_3$) δ 7.84 (d, J = 8.1 Hz, 1H), 7.28–7.22 (m, 1H), 7.14 (s, 4H), 6.69–6.57 (m, 2H), 6.28 (s, 2H), 4.22 (s, 2H), 2.33 (s, 3H). ^{13}C NMR (101 MHz, $CDCl_3$) δ 200.1, 150.8, 136.2, 134.3, 132.2, 131.5, 129.2, 129.2, 117.5, 117.3, 115.7, 45.6, 21.0. HRMS (EI) m/z: $[M]^+$ calcd for $C_{15}H_{15}NO$ 225.1154.

^{1}H NMR Spectrum of **1g**

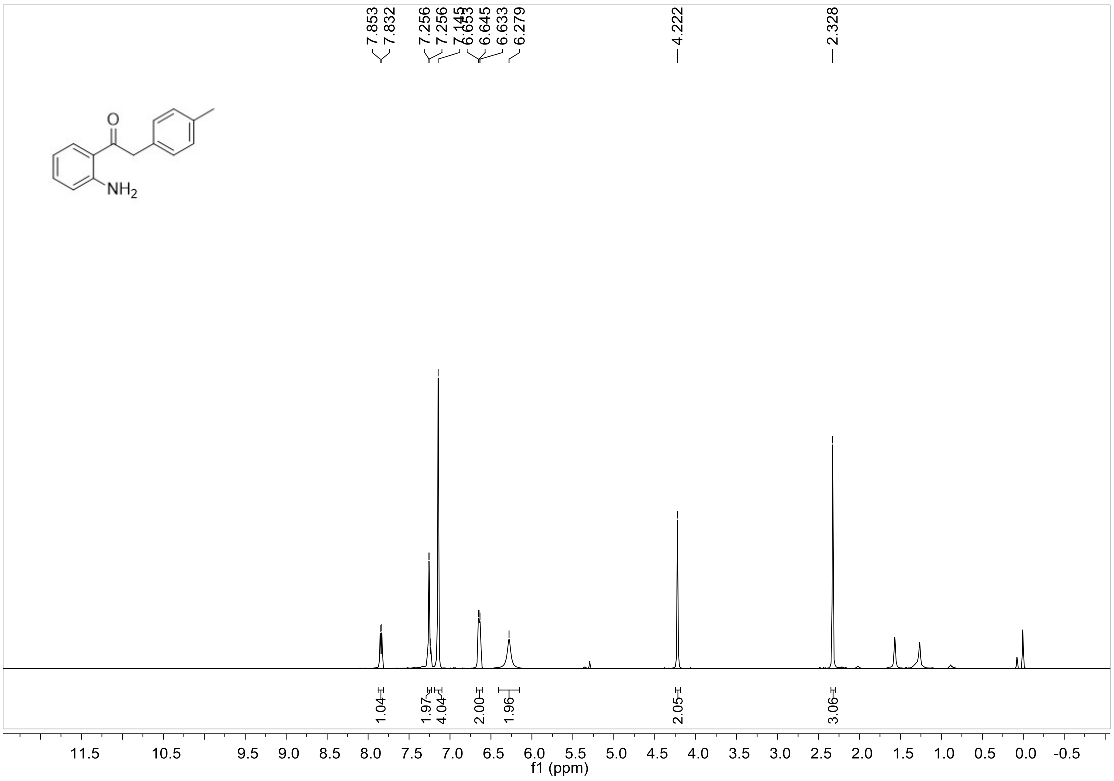

^{13}C NMR Spectrum of **1g**

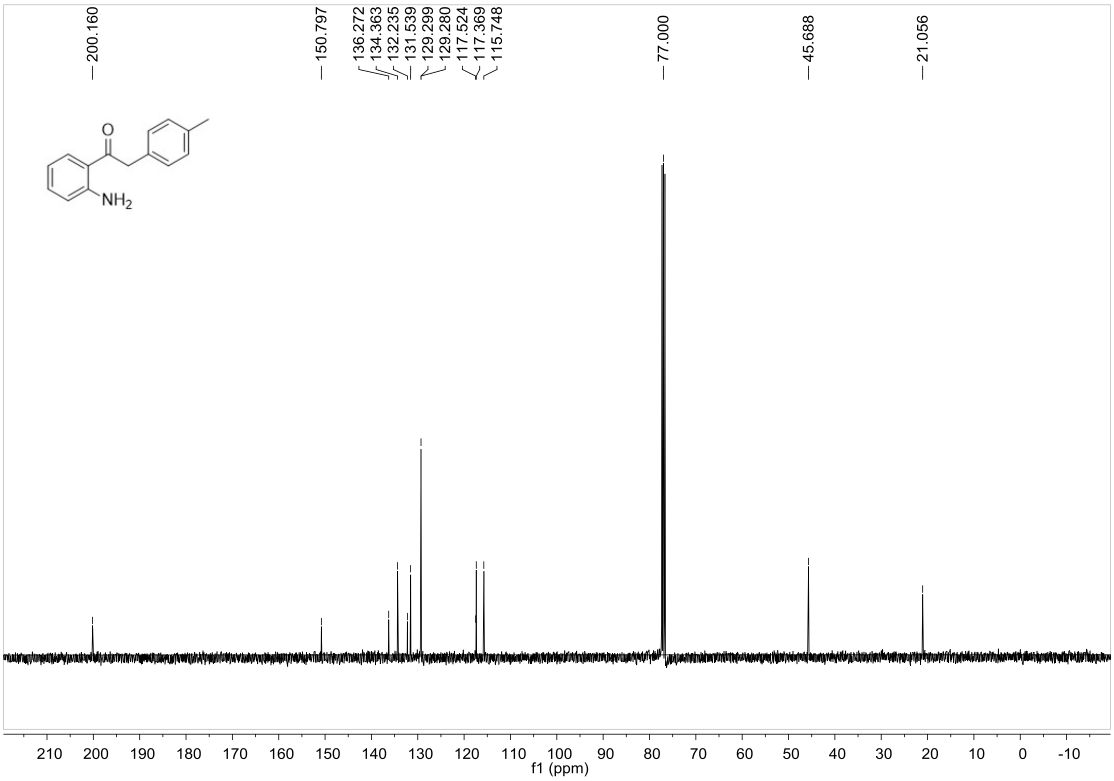

Figure S31. The ^{1}H and ^{13}C NMR Spectrum of 1g

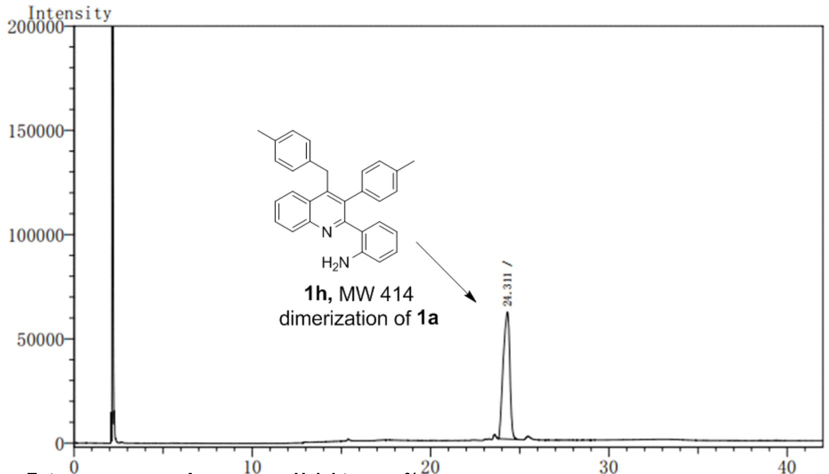

Entry	RT	Area	Height	%
1	24.311	1575299	60780	100.000
All		1575299	60780	

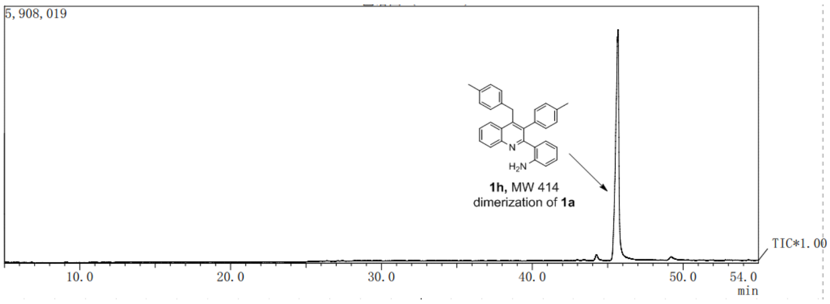

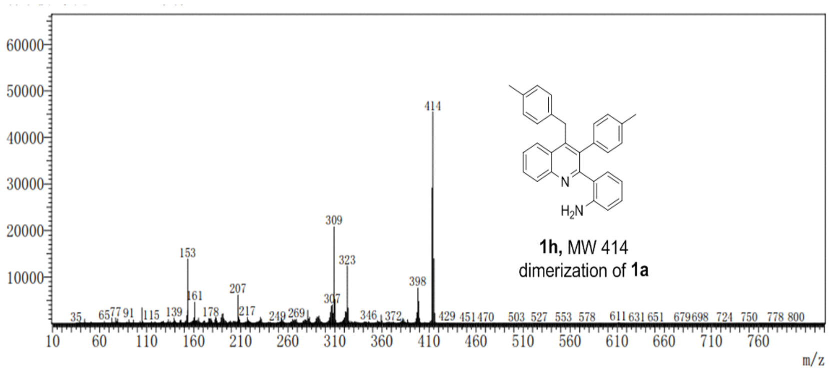

Figure S32. The GC and GC-MS charts of 1h

2-(4-(4-methylbenzyl)-3-(p-tolyl)quinolin-2-yl)aniline (1h)[3]

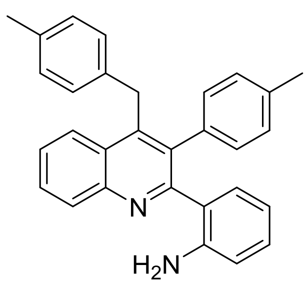

The title compound was prepared according to the procedure of Table S5, entry 2 (six parallel experiments were conducted), and purified by column chromatography on silica gel and eluted with petroleum ether/dichloromethane(10/1) to afford a light yellow solid, mp 200-201 ºC. ^{1}H NMR (400 MHz, $CDCl_3$) δ 8.19 (d, *J* = 8.4 Hz, 1H), 7.96 (d, *J* = 8.4 Hz, 1H), 7.69 (t, *J* = 7.5 Hz, 1H), 7.48 (t, *J* = 7.5 Hz, 1H), 7.09–6.97 (m, 7H), 6.94 (d, *J* = 7.4 Hz, 2H), 6.80 (d, *J* = 7.5 Hz, 1H), 6.71 (d, *J* = 8.0 Hz, 1H), 6.48 (t, *J* = 7.4 Hz, 1H), 4.39 (s, 2H), 4.29 (s, 2H), 2.30 (d, *J* = 12.8 Hz, 6H).^{13}C NMR (101 MHz, $CDCl_3$) δ 158.7, 146.8 144.8, 144.6, 137.0, 136.5, 135.8, 135.3, 135.1, 131.1, 129.8, 129.6, 129.1, 128.9, 128.4, 128.4, 127.9, 126.7, 126.6, 126.2, 125.2, 117.5, 116.2, 35.1, 21.1, 20.8.

^{1}H NMR Spectrum of **1h**

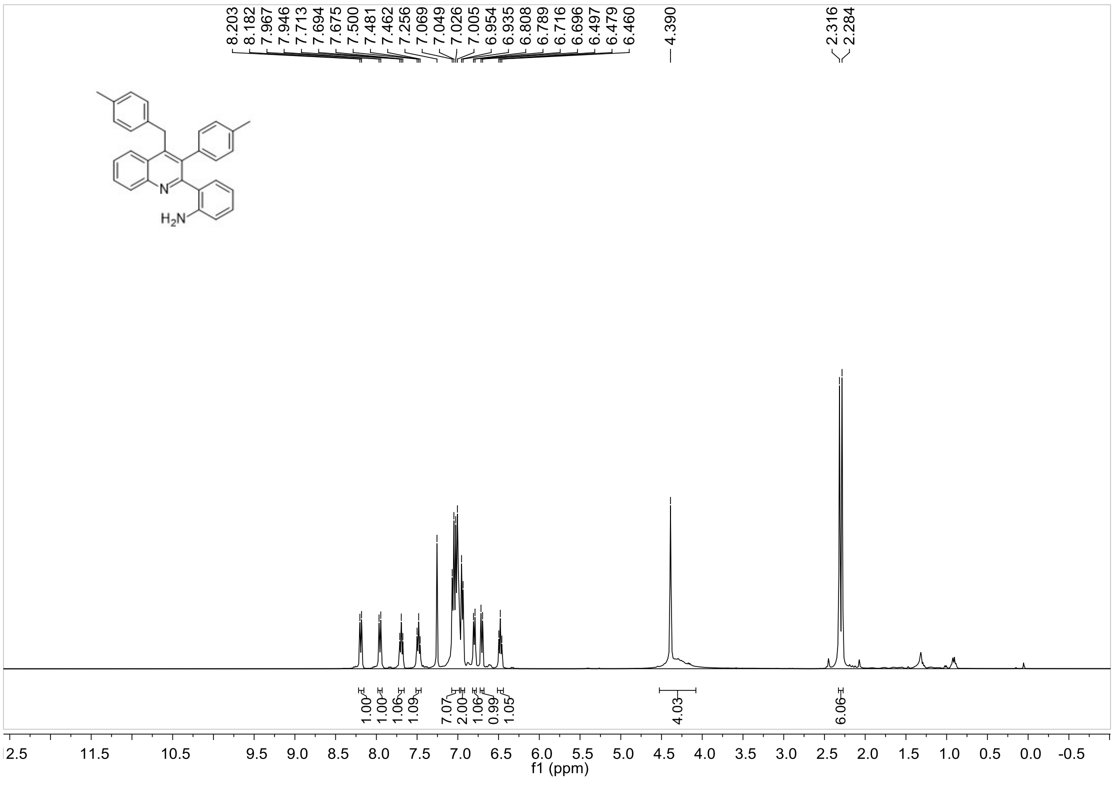

^{13}C NMR Spectrum of **1h**

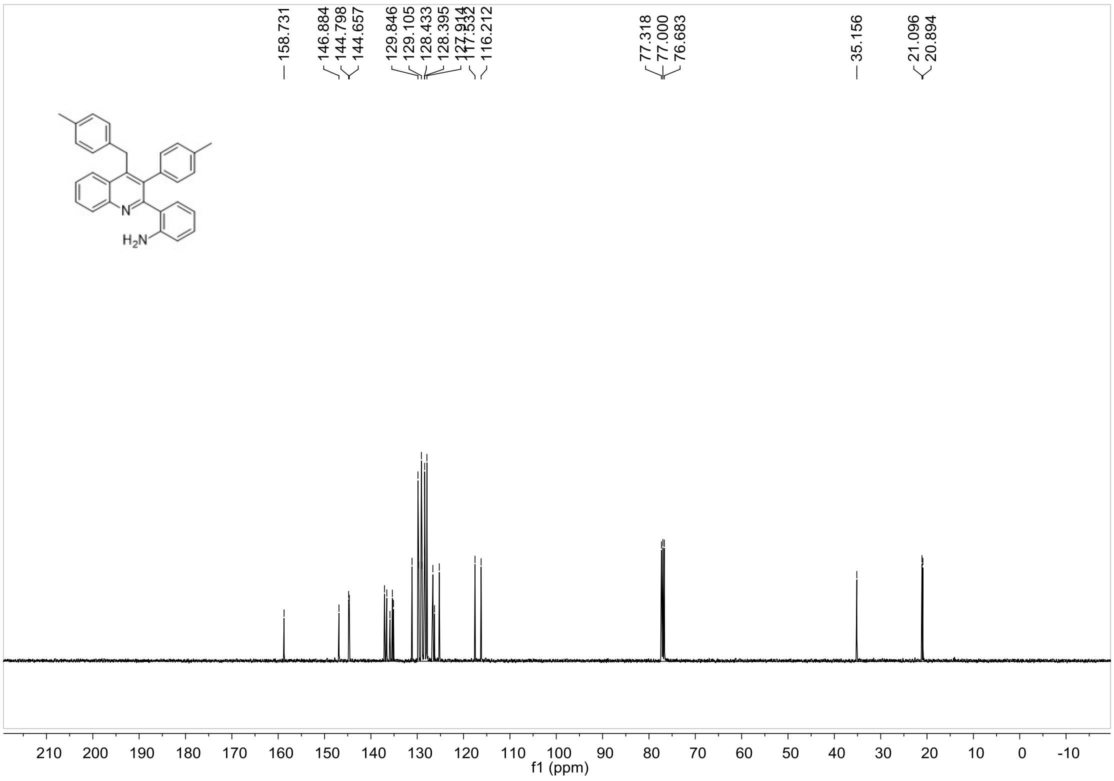

Figure S33. The ^{1}H and ^{13}C NMR Spectrum of 1h

3.3. General Procedure for the Synthesis of C−N Axial Biaryl Compounds

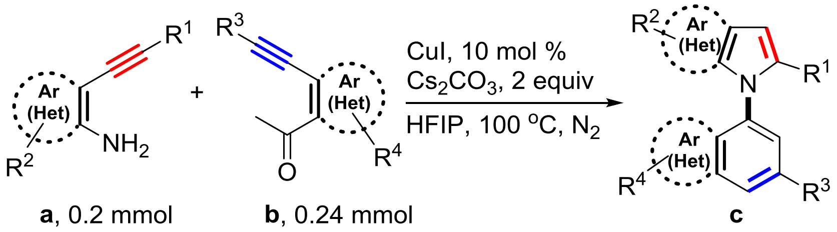

A Schlenk tube of 25 mL equipped with a magnetic stir bar was charged with CuI (3.8 mg, 0.02 mmol, 10 mol %), Cs_2CO_3 (130 mg, 0.4 mmol, 2.0 equiv) in HFIP (1.0 mL); then, *o*-amino arylkyne **a** (0.2 mmol), and *o*-carbonyl arylalkyne **b** (0.24 mmol, 1.2 equiv) were added. The reaction mixture was stirred (800 r/min in IKA RCT basic) at 100 ºC for 24 h under N_2. After completion of the reaction, the reaction mixture was cooled to room temperature. The crude product was purified by flash column chromatography on silica gel to give the desired C−N axial biaryl compounds.

3.4. Gram-scale Synthesis

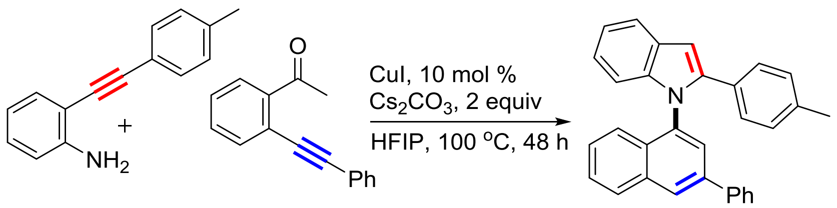

1a, 4 mmol **1b**, 4.8 mmol **1c**, 1.391 g, 85%

A Schlenk tube of 100 mL equipped with a magnetic stir bar was charged with CuI (0.4 mmol, 10 mol %), Cs_2CO_3 (8 mmol, 2.0 equiv) in HFIP (20 mL); then, 2-(*p*-tolylethynyl)aniline **1a** (4.0 mmol) and 2'-phenylethynylacetophenone **1b** (4.8 mmol, 1.2 equiv) were added. The reaction mixture was heated at 100°C for 48 h under N_2. After completion of the reaction, the reaction mixture was cooled to room temperature. The solvent was evaporated under the reduced pressure and the residue was purified by column chromatography on silica gel and eluted with petroleum ether/ethyl acetate (20/1) to afford the desired 2-phenyl-1-(3-phenylnaphthalen-1-yl)-1H-indole **1c** in 85% yield (1.391 g).

4. Investigations of the Reaction Mechanism

4.1 Control Experiments

Scheme S3. Investigations of the cyclization of 1a and 1b

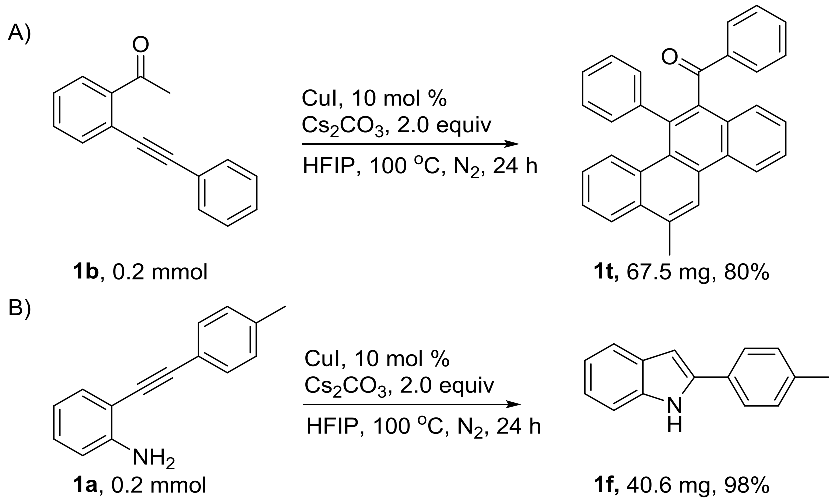

A) A schlenk tube of 25 mL equipped with a magnetic stir bar was charged with CuI (3.8 mg, 0.02 mmol, 10 mol %), Cs_2CO_3 (130 mg, 0.4 mmol, 2.0 equiv) in HFIP (1.0 mL); then, *ortho*-alkynylacetophenone (**1b**, 0.2 mmol) was added. The reaction mixture was stirred (800 r/min in IKA RCT basic) at 100 ºC for 24 h under N_2. After completion of the reaction, the reaction mixture was cooled to room temperature. The solvent was evaporated under the reduced pressure and the residue was purified by column chromatography on silica gel and eluted with petroleum ether/ethyl acetate (20/1) to afford the (12-methyl-5-phenylchrysen-6-yl)(phenyl)methanone[10] in 80% yield (**1t**, 67.5 mg) as a off-white powder. ^{1}H NMR (400 MHz, $CDCl_3$) δ 8.89 (d, *J* = 8.5 Hz, 1H), 8.68 (s, 1H), 8.10 (d, *J* = 8.2 Hz, 1H), 7.78 (d, *J* = 8.2 Hz, 1H), 7.76–7.70 (m, 2H), 7.65 (d, *J* = 7.5 Hz, 1H), 7.58–7.52 (m, 1H), 7.51–7.44 (m, 3H), 7.47 (td, *J* = 8.6, 1.1 Hz, 3H), 7.42 (t, *J* = 7.6 Hz, 1H), 7.35 (t, *J* = 7.4 Hz, 1H), 7.21–7.13 (m, 3H), 7.09–7.03 (m, 1H), 6.93 (t, *J* = 7.5 Hz, 1H), 6.77 (d, *J* = 7.7 Hz, 1H), 2.93 (s, 3H). ^{13}C NMR (101 MHz, $CDCl_3$) δ 200.0, 141.4, 137.9, 137.0, 134.8, 134.5, 133.1, 132.8, 131.4, 131.0, 130.3, 130.0, 129.3, 129.2, 129.0, 128.7, 128.7, 128.1, 128.0, 127.2, 127.2, 126.9, 126.0, 125.9, 125.8, 124.5, 124.1, 123.5, 121.4, 20.6.

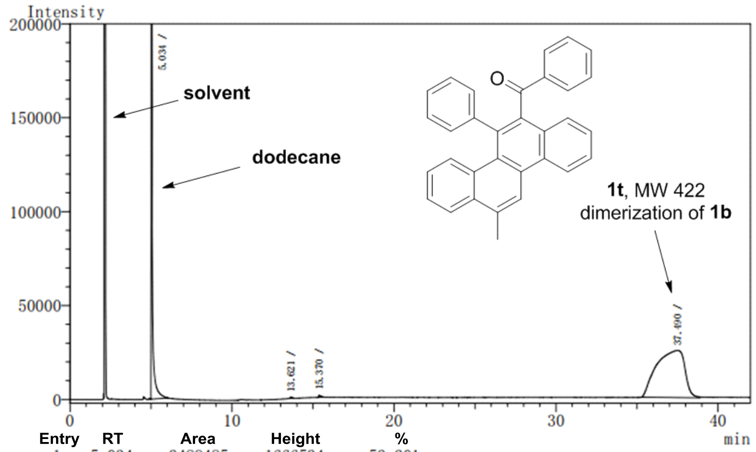

Entry	RT	Area	Height	%
1	5.034	3489485	1666534	53.301
2	13.621	5696	866	0.087
3	15.370	10992	1201	0.168
4	37.490	3040575	25119	46.444
All		6546748	1693720	

Figure S34. The GC chart of the crude reaction mixture of A.

B) A Schlenk tube of 25 mL equipped with a magnetic stir bar was charged with CuI (3.8 mg, 0.02 mmol, 10 mol %), Cs_2CO_3 (130 mg, 0.4 mmol, 2.0 equiv) in HFIP (1.0 mL); then, 2-(*p*-tolylethynyl)aniline (**1a**, 0.2 mmol) was added. The reaction mixture was stirred (800 r/min in IKA RCT basic) at 100 ºC for 24 h under N_2. After completion of the reaction, the reaction mixture was cooled to room temperature. The solvent was evaporated under the reduced pressure and the residue was purified by column chromatography on silica gel and eluted with petroleum ether/ethyl acetate (20/1) to afford the 2-(*p*-tolyl)-1H-indole[2] in 98% yield (**1f**, 40.6 mg) as a off-white powder. ^{1}H NMR (400 MHz, $CDCl_3$) δ 8.32 (s, 1H), 7.64 (d, *J* = 7.7 Hz, 1H), 7.57 (d, *J* = 7.4 Hz, 2H), 7.39 (d, *J* = 7.9 Hz, 1H), 7.29–7.24 (m, 2H), 7.20 (t, *J* = 7.4 Hz, 1H), 7.14 (t, *J* = 7.3 Hz, 1H), 6.80 (s, 1H), 2.41 (s, 3H). ^{13}C NMR (101 MHz, $CDCl_3$) δ 138.0, 137.6, 136.6, 129.6, 129.5, 129.3, 125.0, 122.0, 120.4, 120.1, 110.7, 99.3, 21.2.

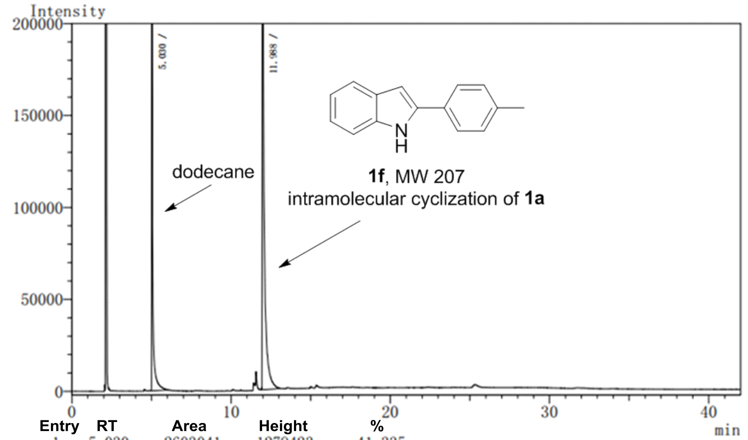

Entry	RT	Area	Height	%
1	5.030	2603041	1279423	41.335
2	11.988	3694388	971028	58.665
All		6297429	2250451	

Figure S35. The GC chart of the crude reaction mixture of B

Scheme S4. Investigations of the condensation reaction of amines and ketones

C)

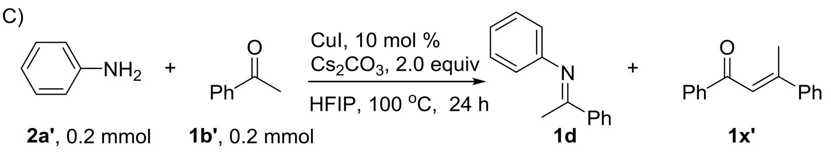

	1d	1x'
1 h	6%	trace
4 h	7%	trace
8 h	8%	trace
12 h	9%	trace
24 h	9%	trace

D)

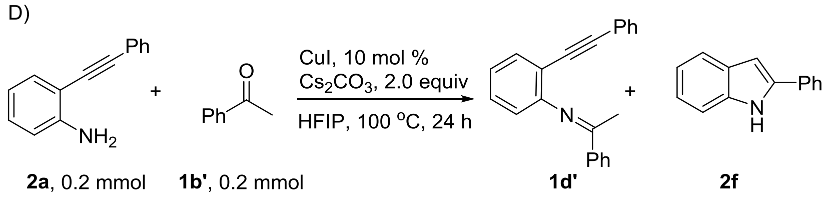

	1d'	2f
1 h	trace	34%
2 h	trace	65%
4 h	trace	87%
8 h	trace	88%
12 h	trace	92%
24 h	trace	95%

C) A Schlenk tube of 25 mL equipped with a magnetic stir bar was charged with CuI (3.8 mg, 0.02 mmol, 10 mol %), Cs_2CO_3 (130 mg, 0.4 mmol, 2.0 equiv) in HFIP (1.0 mL); then, anilines (**2a'**, 0.2 mmol) and acetophenone (**1b'**, 0.24 mmol, 1.2 equiv) were added. The reaction mixture was stirred (800 r/min in IKA RCT basic) at 100 ºC for 1 h, 2 h, 4 h, 8 h, 12 h, and 24 h under N_2.

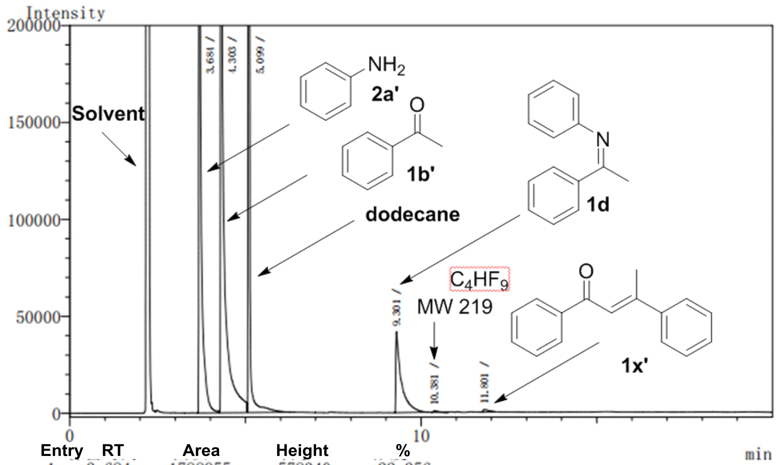

Entry	RT	Area	Height	%
1	3. 684	1788955	578340	22. 356
2	4. 303	2707907	795809	33. 840
3	5. 099	3037020	1553079	37. 953
4	9. 301	444667	41729	5. 557
5	10. 381	8122	759	0. 101
6	11. 801	15422	1257	0. 193
All		8002093	2970973	

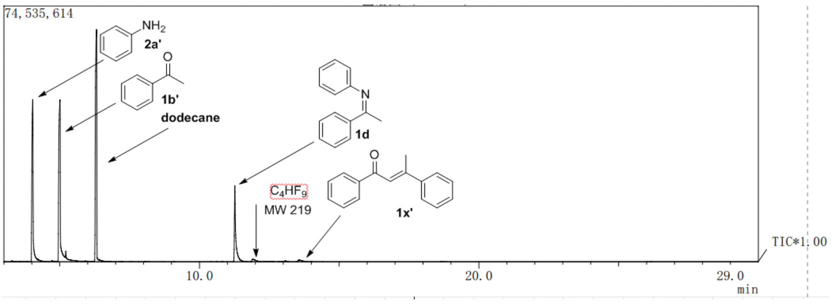

Figure S36. The GC and GC-MS charts of the crude reaction mixture of C for 24 h

D) A Schlenk tube of 25 mL equipped with a magnetic stir bar was charged with CuI (3.8 mg, 0.02 mmol, 10 mol %), Cs_2CO_3 (130 mg, 0.4 mmol, 2.0 equiv) in HFIP (1.0 mL); then, *ortho*-alkynylanilines (**2a**, 0.2 mmol) and acetophenone (**1b'**, 0.24 mmol, 1.2 equiv) were added. The reaction mixture was stirred (800 r/min in IKA RCT basic) at 100 ºC for 1 h, 2 h, 4 h, 8 h, 12 h, and 24 h under N_2.

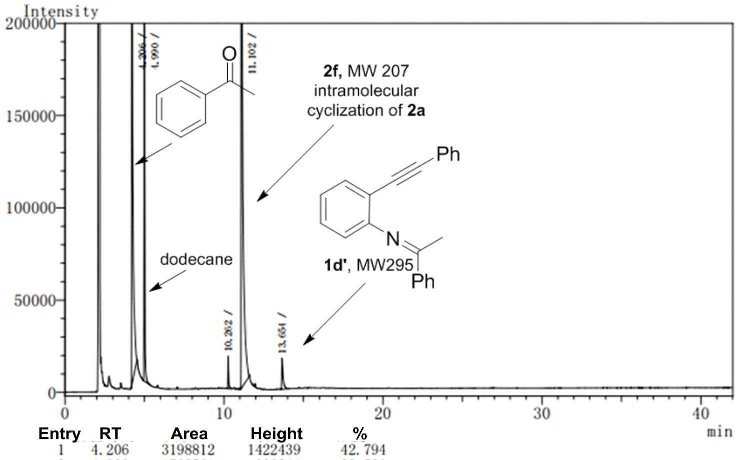

Entry	RT	Area	Height	%
1	4.206	3198812	1422439	42.794
2	4.990	1762561	986244	23.580
3	10.262	55163	17603	0.738
4	11.102	2361030	498314	31.586
5	13.654	97331	16635	1.302
All		7474897	2941235	

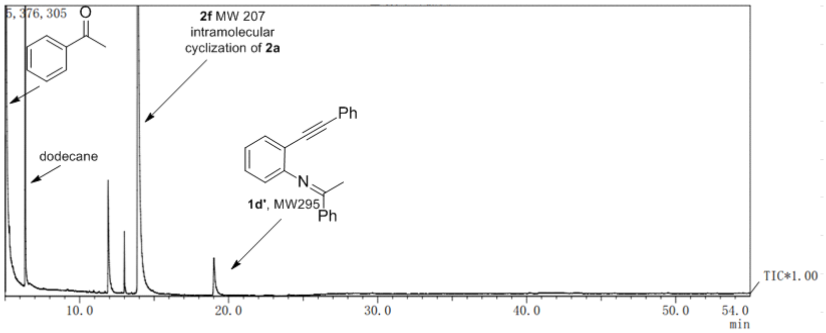

Figure S37. The GC and GC-MS charts of the crude reaction mixture of D for 24 h

Scheme S5. Investigations of the cyclization sequence

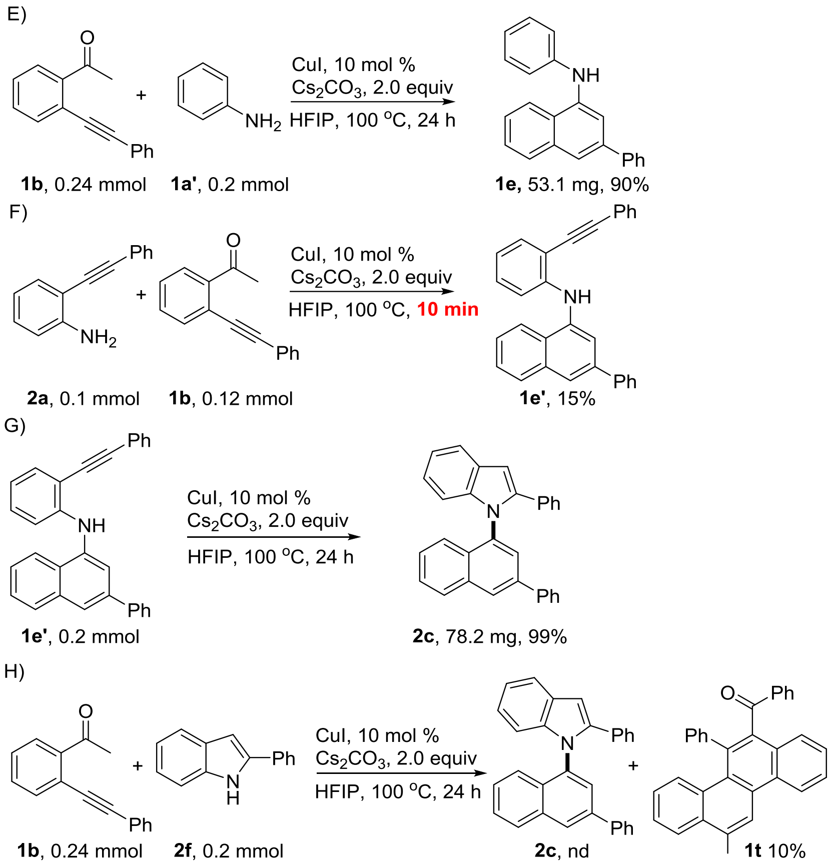

E) A Schlenk tube of 25 mL equipped with a magnetic stir bar was charged with CuI (3.8 mg, 0.02 mmol, 10 mol %), Cs_2CO_3 (130 mg, 0.4 mmol, 2.0 equiv) in HFIP (1.0 mL); then, aniline (**1a'**, 0.2 mmol), and *ortho*-alkynylacetophenone (**1b**, 0.24 mmol, 1.2 equiv) were added. The reaction mixture was stirred (800 r/min in IKA RCT basic) at 100 °C for 24 h under N_2. After completion of the reaction, the reaction mixture was cooled to room temperature. The crude product was purified by flash column chromatography on silica gel to give the desired N,3-diphenylnaphthalen-1-amine[11] in 90% yield (**1e**, 53.1 mg). ^{1}H NMR (400 MHz, $CDCl_3$) δ 8.06 (d, *J* = 8.3 Hz, 1H), 7.96 (d, *J* = 8.1 Hz, 1H), 7.83–7.81 (m, 1H), 7.73 (d, *J* = 7.2 Hz, 3H), 7.60–7.54 (m, 1H), 7.51 (t, *J* = 7.7 Hz, 3H), 7.42 (t, *J* = 7.3 Hz, 1H), 7.34 (t, *J* = 7.8 Hz, 2H), 7.11 (d, *J* = 7.8 Hz, 2H), 7.00 (t, *J* = 7.3 Hz, 1H), 6.02 (s, 1H). ^{13}C NMR (101 MHz, $CDCl_3$) δ 144.5, 140.9, 139.2, 138.8, 134.9, 129.3, 128.8, 128.7, 127.3, 127.2, 126.7, 126.5, 125.6, 121.6, 120.7, 120.5, 117.4, 115.1.

F) A Schlenk tube of 25 mL equipped with a magnetic stir bar was charged with CuI (1.9 mg, 0.01 mmol, 10 mol %), Cs_2CO_3 (66 mg, 0.2 mmol, 2.0 equiv) in HFIP (1.0 mL); then, (0.1 mmol), and 2'-phenylethynylacetophenone **1b** (0.12 mmol, 1.2 equiv) were added. The reaction mixture was stirred (800 r/min in

IKA RCT basic) at 100 ºC for 10min under N_2. After completion of the reaction, the reaction mixture was cooled to room temperature. The crude product was purified by flash column chromatography on silica gel to give the desired **1e'** in 15% yield (29.6 mg from five parallel experiments). mp 165-167 °C. A white solid. ^{1}H NMR (400 MHz, $CDCl_3$) δ 8.11 (d, J = 8.3 Hz, 1H), 7.96 (d, J = 7.9 Hz, 1H), 7.89 (s, 1H), 7.83 (s, 1H), 7.73 (d, J = 7.6 Hz, 2H), 7.58–7.44 (m, 7H), 7.42-7.33 (m, 4H), 7.17 (t, J = 7.7 Hz, 1H), 7.04 (d, J = 8.3 Hz, 1H), 6.91 (s, 1H), 6.84 (t, J = 7.4 Hz, 1H). ^{13}C NMR (101 MHz, $CDCl_3$) δ 146.3, 140.8, 138.8, 137.8, 135.0, 132., 131.5, 129.6, 128.8, 128.4, 128.4, 128.0, 127.5, 127.3, 126.6, 126.1, 123.0, 122.2, 121.9, 118.8, 118.4, 113.5, 109.6, 95.8, 85.7. HRMS (EI) m/z: $[M]^+$ calcd for $C_{30}H_{20}FN$ 395.1674; found 395.1671.

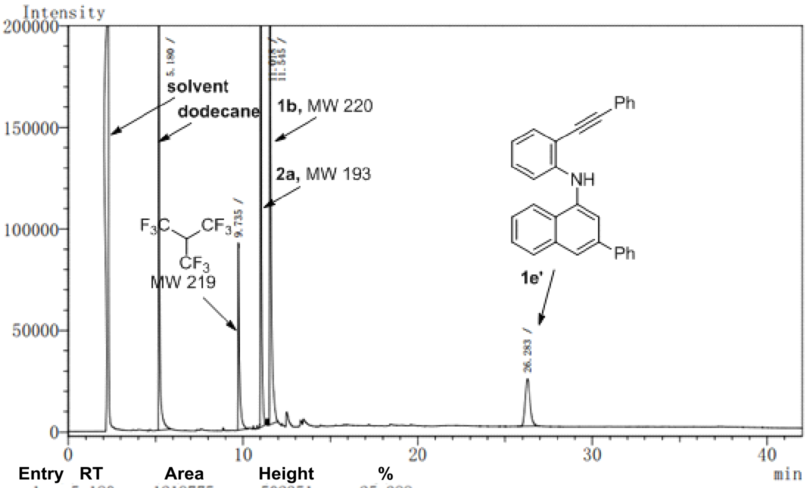

Entry	RT	Area	Height	%
1	5. 180	1219775	502351	25. 388
2	9. 735	481360	91737	10. 019
3	11. 018	1389091	437828	28. 912
4	11. 545	1254298	250677	26. 107
5	26. 283	459942	23424	9. 573
All		4804466	1306017	

Figure S38. The GC and GC-MS charts of the crude reaction mixture of F for 10 min

G) A Schlenk tube of 25 mL equipped with a magnetic stir bar was charged with CuI (3.8 mg, 0.02 mmol, 10 mol %), Cs_2CO_3 (130 mg, 0.4 mmol, 2.0 equiv) in HFIP (1.0 mL); then, 3-phenyl-N-(2-(phenylethynyl)phenyl)naphthalen-1-amine (**1e'**, 0.2 mmol) was added. The reaction mixture was stirred (800 r/min in IKA RCT basic) at 100 ºC for 24 h under N_2. After completion of the reaction, the reaction mixture was cooled to room temperature. The crude product was purified by flash column chromatography on silica gel to give the desired product **2c** in 99% yield (78.2 mg).

H) A Schlenk tube of 25 mL equipped with a magnetic stir bar was charged with CuI (3.8 mg, 0.02 mmol, 10 mol %), Cs_2CO_3 (130 mg, 0.4 mmol, 2.0 equiv) in HFIP (1.0 mL); then, 2-phenyl indoles (**2f**, 0.2 mmol), and *ortho*-

alkynylacetophenone (**1b**, 0.24 mmol, 1.2 equiv) were added. The reaction mixture was stirred (800 r/min in IKA RCT basic) at 100 ºC for 24 h under N_2.

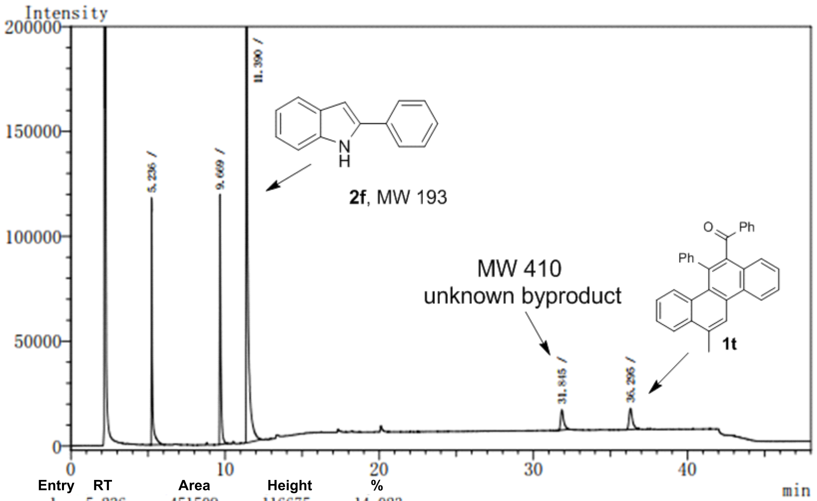

Entry	RT	Area	Height	%
1	5.236	451509	116675	14.023
2	9.669	549336	118964	17.061
3	11.390	1929382	356076	59.923
4	31.845	134007	9788	4.162
5	36.295	155517	10139	4.830
All		3219751	611642	

Figure S39. The GC and GC-MS charts of the crude reaction mixture of H

Scheme S6. The synthesis of 3-phenyl-N-(2-(phenylethynyl)phenyl)naphthalen-1-amine (1e')

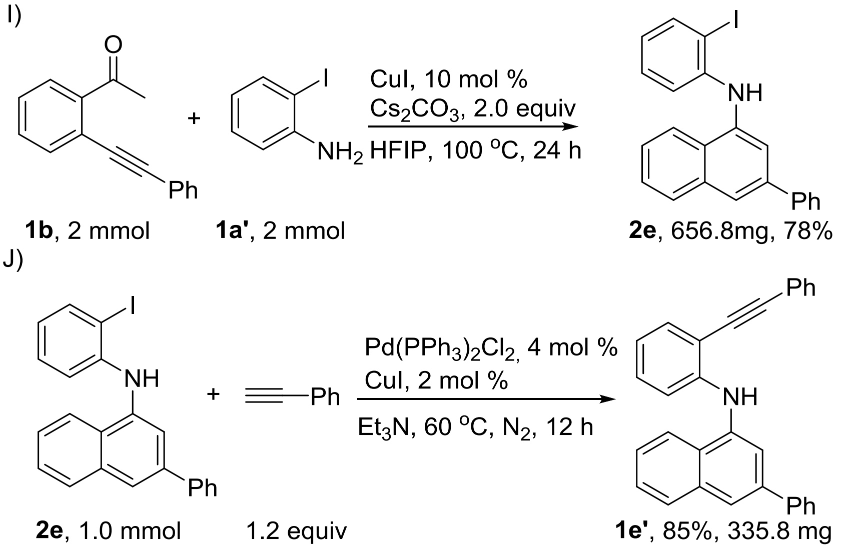

I) A Schlenk tube of 25 mL equipped with a magnetic stir bar was charged with CuI (38 mg, 0.2 mmol, 10 mol %), Cs_2CO_3 (1.3 g, 4 mmol, 2.0 equiv) in HFIP (10 mL); then, *o*-Iodoaniline (**1a'**, 2 mmol), and *ortho*-alkynylacetophenone (**1b**, 2 mmol) were added. The reaction mixture was stirred (800 r/min in IKA RCT basic) at 100 ºC for 24 h under N_2. After completion of the reaction, the reaction mixture was cooled to room temperature. The crude product was purified by flash column chromatography on silica gel to give the desired N-(2-iodophenyl)-3-phenylnaphthalen-1-amine in 78% yield (**2e**, 656.8 mg). Light yellow solid; mp 114–117 °C; ^{1}H NMR (400 MHz, $CDCl_3$) δ 8.03 (d, *J* = 7.4 Hz, 1H), 7.96 (d, *J* = 7.9 Hz, 1H), 7.90 (s, 1H), 7.86–7.78 (m, 1H), 7.75–7.65 (m,3H), 7.62–7.42 (m, 4H), 7.42–7.35 (m, 1H), 7.15 (t, *J* = 7.6 Hz, 1H), 6.92 (d, *J* = 8.2 Hz, 1H), 6.68–6.52 (m, 1H), 6.33 (s, 1H). ^{13}C NMR (101 MHz, $CDCl_3$) δ 145.3, 140.6, 139.3, 138.8, 138.1, 135.0, 129.1, 128.8, 128.8, 127.9, 127.5, 127.3, 126.7, 126.1, 122.4, 122.1, 121.2, 118.7, 115.4, 87.4. HRMS (EI) m/z: $[M]^+$ calcd for $C_{30}H_{20}FN$ 421.0327; found 421.0325.

J) To a 25 mL Schlenk tube charged with N-(2-iodophenyl)-3-phenylnaphthalen-1-amin (**2e**, 1.0 mmol, 1.0 equiv), $Pd(PPh_3)_2Cl_2$ (0.04 mmol, 2 mol %), and CuI (0.08 mmol, 4 mol %) in degassed Et_3N (8 mL) was added aryl alkynes (1.2 mmol, 1.2 equiv), and the resulting solution was stirred at 60 °C for 12 h. Upon completion, the solvent was removed under reduced pressure, and the residue was extracted with EtOAc (3 x 5 mL). The combined organic layer was dried over Na_2SO_4 and concentrated. The residue was purified by a silica gel flash chromatography (petroleum ether/dichloromethane = 10/1) to afford a white powder in 85% yield (335.8 mg).

4.2 Deuterium Labeling Experiments

Scheme S7. The deuterium labeling experiments (Eq. K and Eq. L)

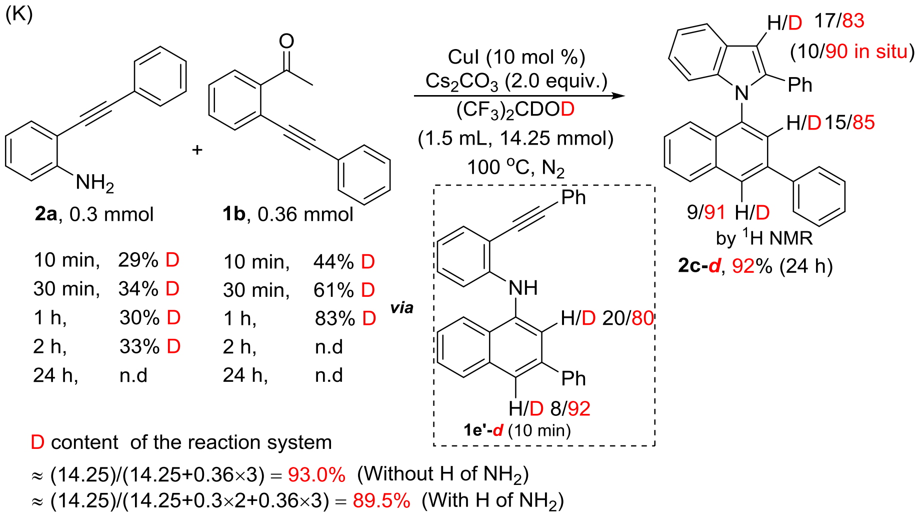

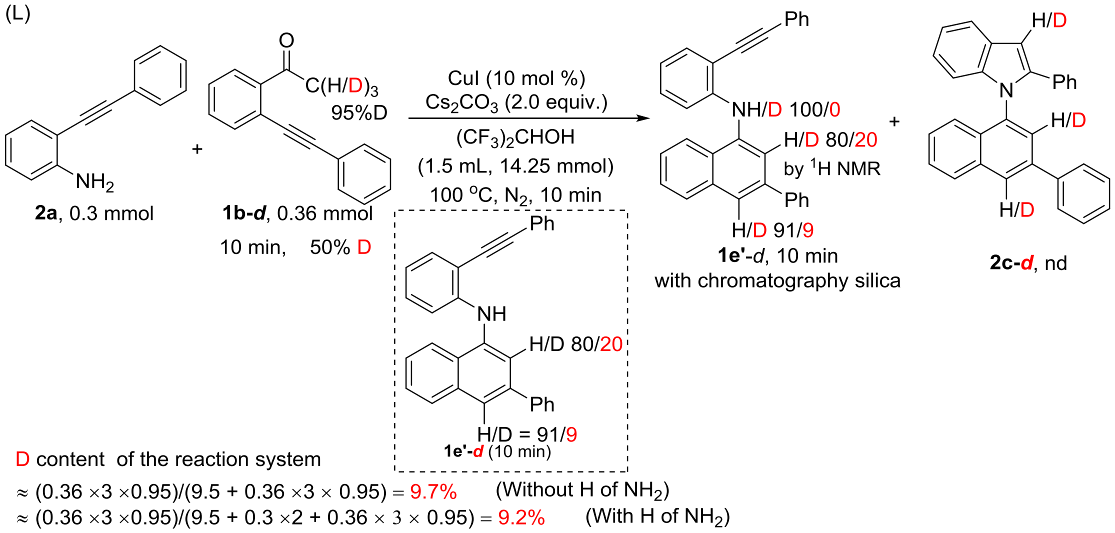

***Note:** Deuterium content of the "reaction system: deuterium content of the total H and D that could be exchanged.*

***2a** and **1b** cannot be detected at 4 h by GC and ^{1}H NMR.*

K) A Schlenk tube of 25 mL equipped with a magnetic stir bar was charged with CuI (5.7 mg, 0.03 mmol, 10 mol %), Cs_2CO_3 (195 mg, 0.6 mmol, 2.0 equiv.) in HFIP-*d*$_2$ (1.5 mL, 14.25 mmol, >98% D), then, 2-(phenylethynyl)aniline **2a** (57.9 mg, 0.3 mmol), and 2'-phenylethynylacetophenone **1b** (79.2 mg, 0.36 mmol, 1.2 equiv.) were added. The reaction mixture was stirred (800 r/min in IKA RCT basic) at 100 °C under N_2, and was extracted 100 ul for monitoring by ^{1}H NMR at 10 min, 30 min, 1 h, 2 h, and 24 h, respectively. In addition, the reaction mixture of 10 min and 24 h were extracted additional 200 ul for isolating the deuteration intermediate **1e'-*d*** and desired deuteration product **2c-*d*** with flash column chromatography on silica gel.

The parallel experiment was conducted with 24 h, after completion of the reaction, the reaction mixture was cooled to room temperature, the crude product was purified by flash column chromatography on silica gel to give the desired **2c-*d*** in 92% yield (109.0 mg).

L) A Schlenk tube of 25 mL equipped with a magnetic stir bar was charged with CuI (5.7 mg, 0.03 mmol, 10 mol %), Cs_2CO_3 (195 mg, 0.6 mmol, 2.0 equiv.) in HFIP (1.5 mL, 14.25 mmol,), then, 2-(phenylethynyl)aniline **2a** (57.9 mg, 0.3 mmol), and 2'-phenylethynylacetophenone **1b-*d*** (79.2 mg, 0.36 mmol, 1.2 equiv., 95% D) were added. The reaction mixture was stirred (800 r/min in IKA RCT basic) at 100 ºC under N_2, and was extracted 100 ul for monitoring by ^1H NMR at 10 min. In addition, the reaction mixture of 10 min was extracted additional 200 ul for isolating the deuteration intermediate **1e'-*d*** with flash column chromatography on silica gel.

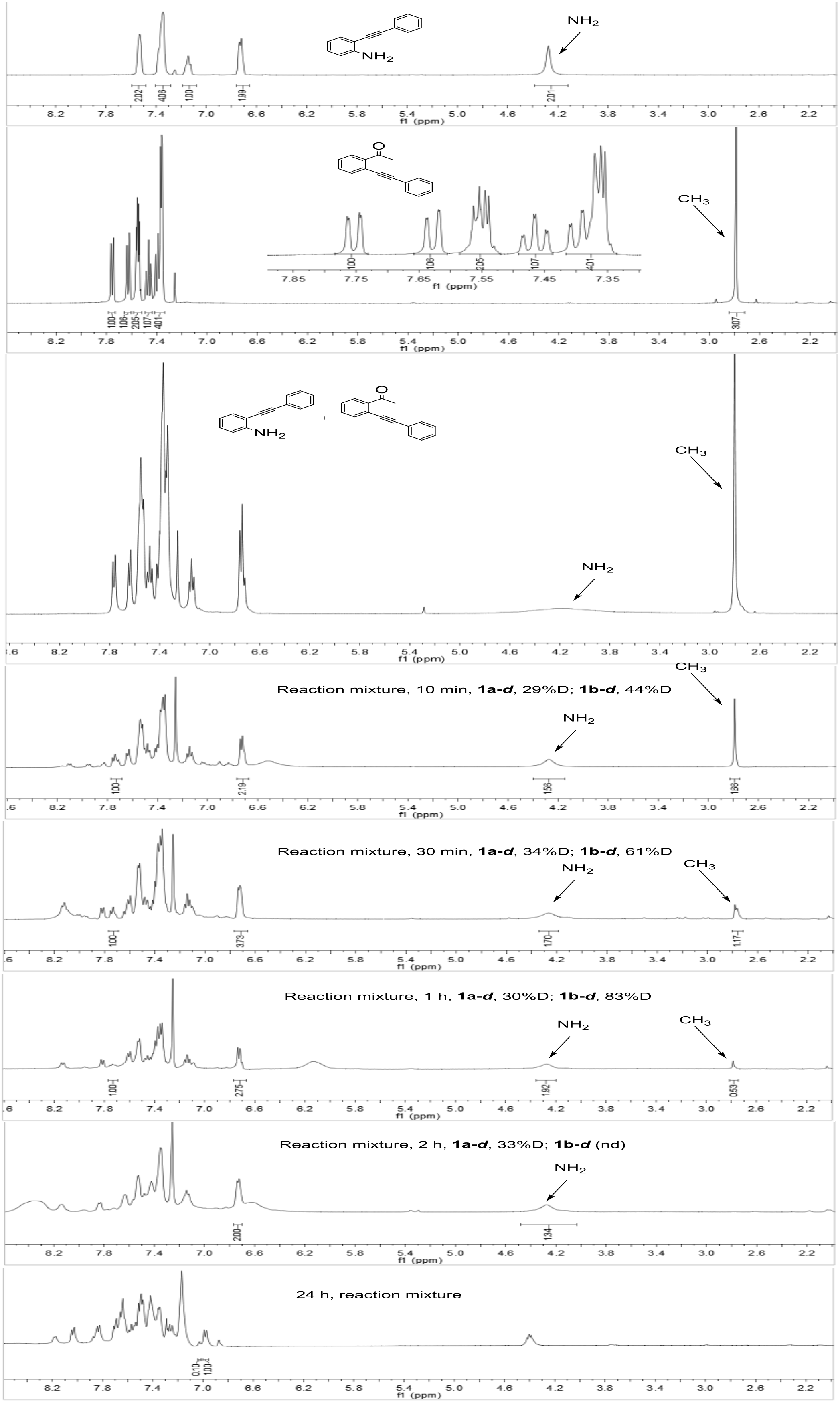

Figure S40. ^{1}H NMR Spectrum of Eq. (K) at 10 min, 30 min, 1 h, 2 h, and 24 h.

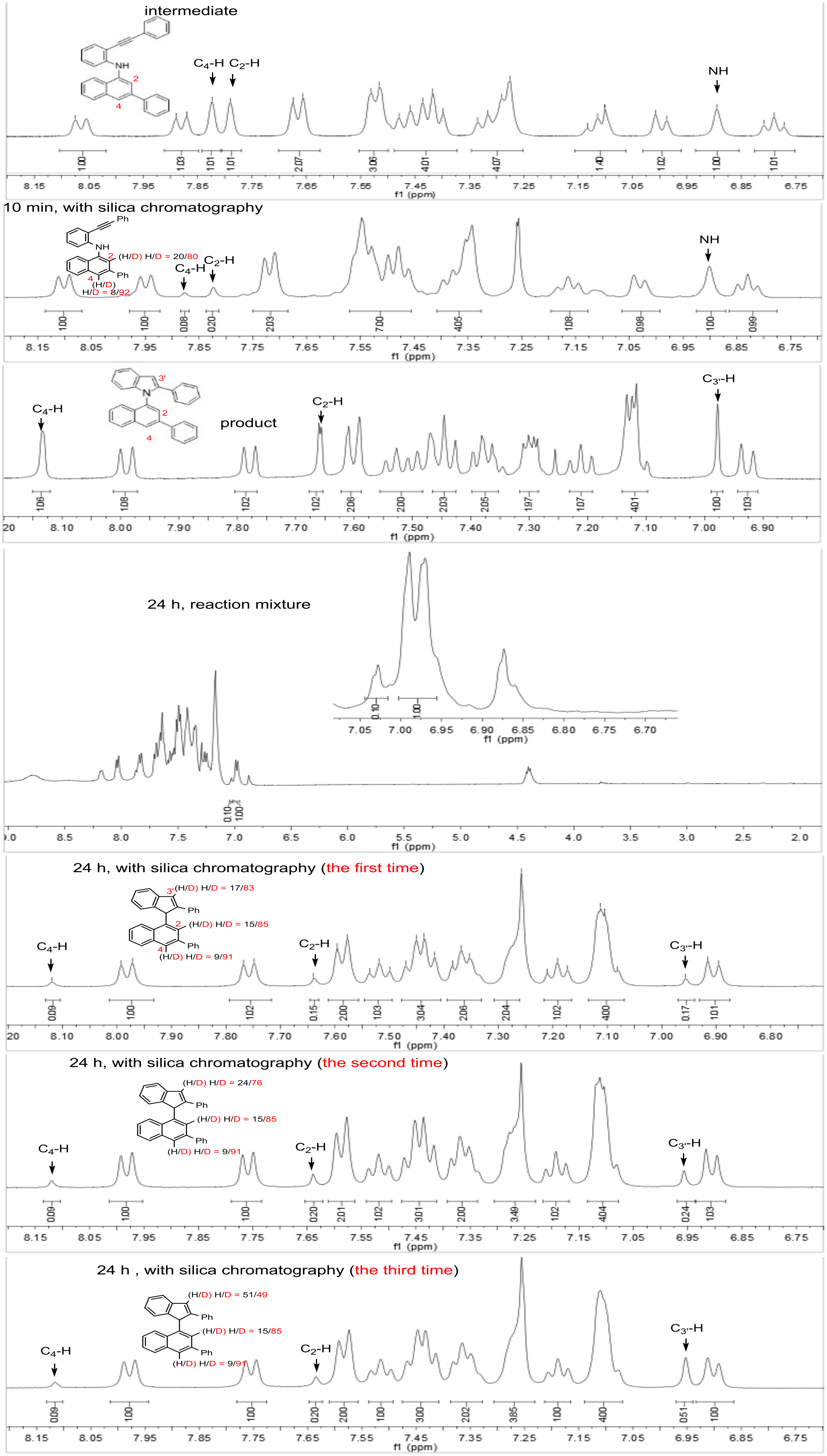

Figure S41. The ^{1}H NMR spectrum of Eq. (K) with silica chromatography at 10 min and 24 h.

3-Phenyl-N-(2-(phenylethynyl)phenyl)naphthalen-1-amine (1e'-*d*, Eq. K, 10 min, with silica chromatography)

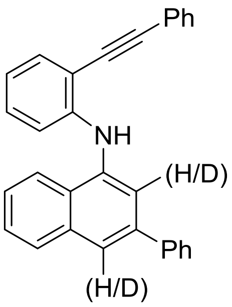

^{1}H NMR (400 MHz, $CDCl_3$) δ 8.10 (d, *J* = 8.0 Hz, 1H), 7.95 (d, *J* = 8.0 Hz, 1H), 7.88 (s, 0.08H), 7.82 (s, 0.20H), 7.72 (d, *J* = 8.0 Hz, 2H), 7.58–7.43 (m, 7H), 7.41–7.32 (m, 4H), 7.16 (t, *J* = 7.7 Hz, 1H), 7.03 (d, *J* = 8.2 Hz, 1H), 6.90 (s, 1H), 6.83 (t, *J* = 7.4 Hz, 1H).

^{1}H NMR Spectrum of **1e'-*d*** (Eq. K, 10 min, with silica chromatography)

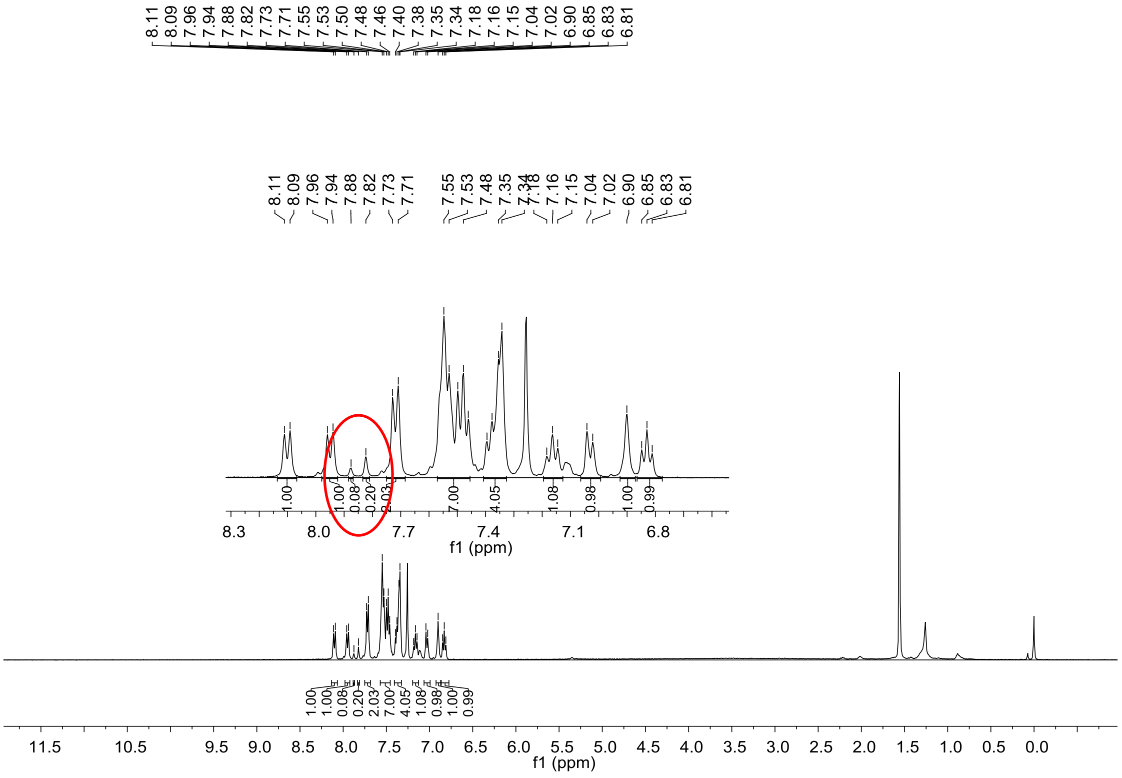

2-phenyl-1-(3-phenylnaphthalen-1-yl)-1H-indole (**2c-*d***, Eq. K, 24 h, with silica chromatography first time)

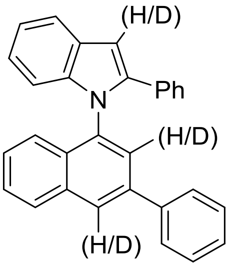

^{1}H NMR (400 MHz, $CDCl_3$) δ 8.12 (s, 0.09H), 7.98 (d, *J* = 8.1 Hz, 1H), 7.76 (d, *J* = 7.7 Hz, 1H), 7.64 (s, 0.15H), 7.58 (d, *J* = 7.6 Hz, 2H), 7.52 (t, *J* = 7.4 Hz, 1H), 7.44 (dd, *J* = 13.3, 7.7 Hz, 3H), 7.39–7.33 (m, 2H), 7.26 (s, 2H), 7.19 (t, *J* = 7.4 Hz, 1H), 7.15–7.07 (m, 4H), 6.95 (s, 0.17H), 6.90 (d, *J* = 8.0 Hz, 1H).

^{1}H NMR Spectrum of **2c-*d*** (Eq. K, 24 h, with silica chromatography first time)

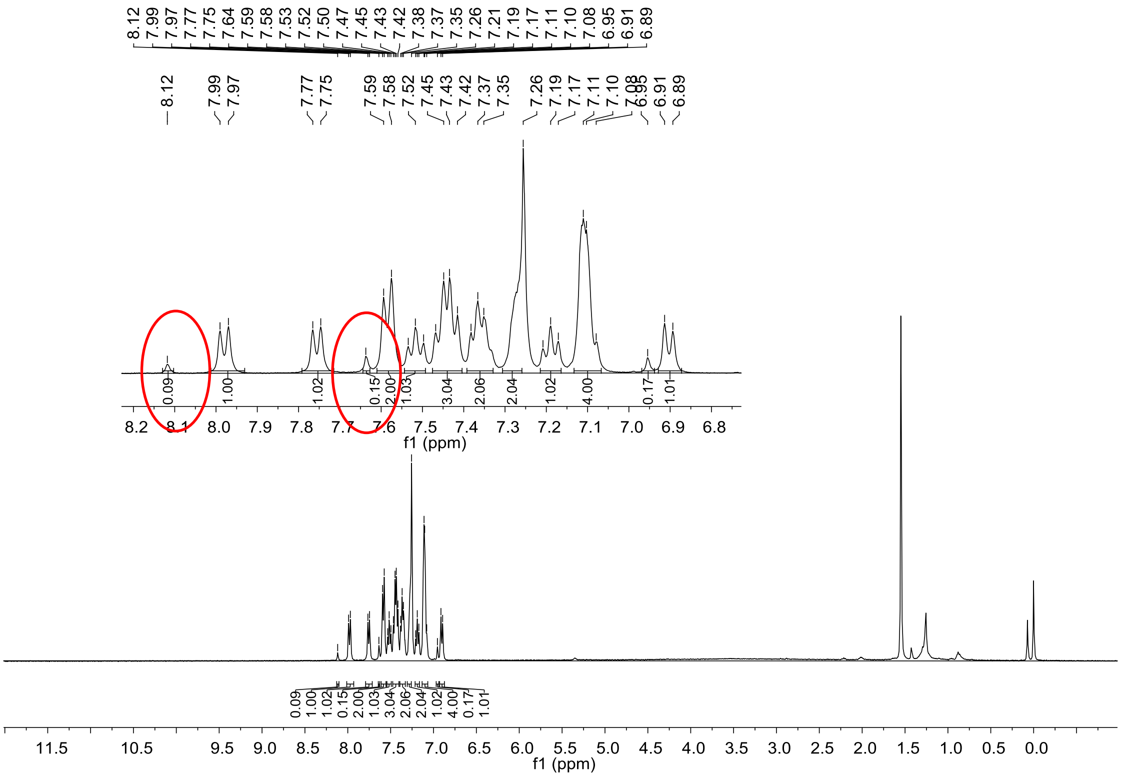

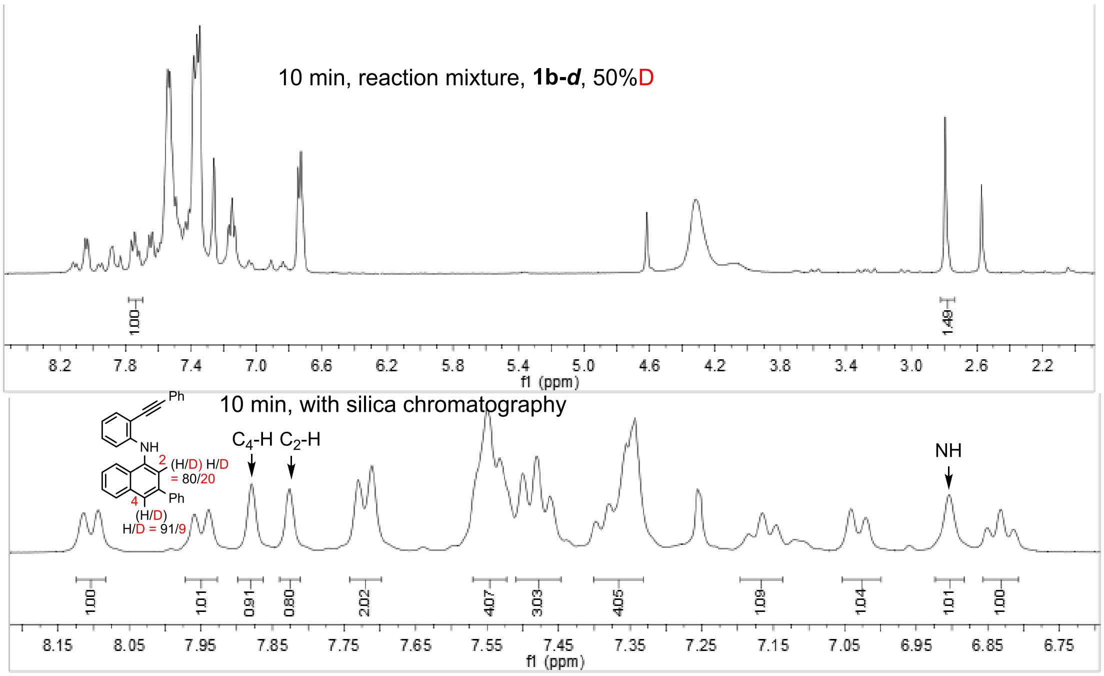

Figure S42. The ^{1}H NMR spectrum of reaction mixture and with silica chromatography of Eq. (L) at 10 min.

3-Phenyl-N-(2-(phenylethynyl)phenyl)naphthalen-1-amine (**1e'-*d***, Eq. L, 10 min, with silica chromatography)

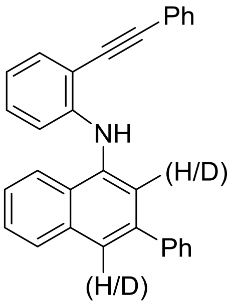

^{1}H NMR (400 MHz, $CDCl_3$) δ 8.11 (d, *J* = 8.2 Hz, 1H), 7.95 (d, *J* = 7.9 Hz, 1H), 7.88 (s, 0.91H), 7.83 (s, 0.80H), 7.73 (d, *J* = 7.4 Hz, 2H), 7.58–7.53 (m, 4H), 7.52–7.45 (m, 3H), 7.41–7.35 (m, 4H), 7.17 (t, *J* = 7.7 Hz, 1H), 7.04 (d, *J* = 8.3 Hz, 1H), 6.91 (s, 1H), 6.84 (t, *J* = 7.4 Hz, 1H).

^{1}H NMR Spectrum of **1e'-*d*** (Eq. L, 10 min, with silica chromatography)

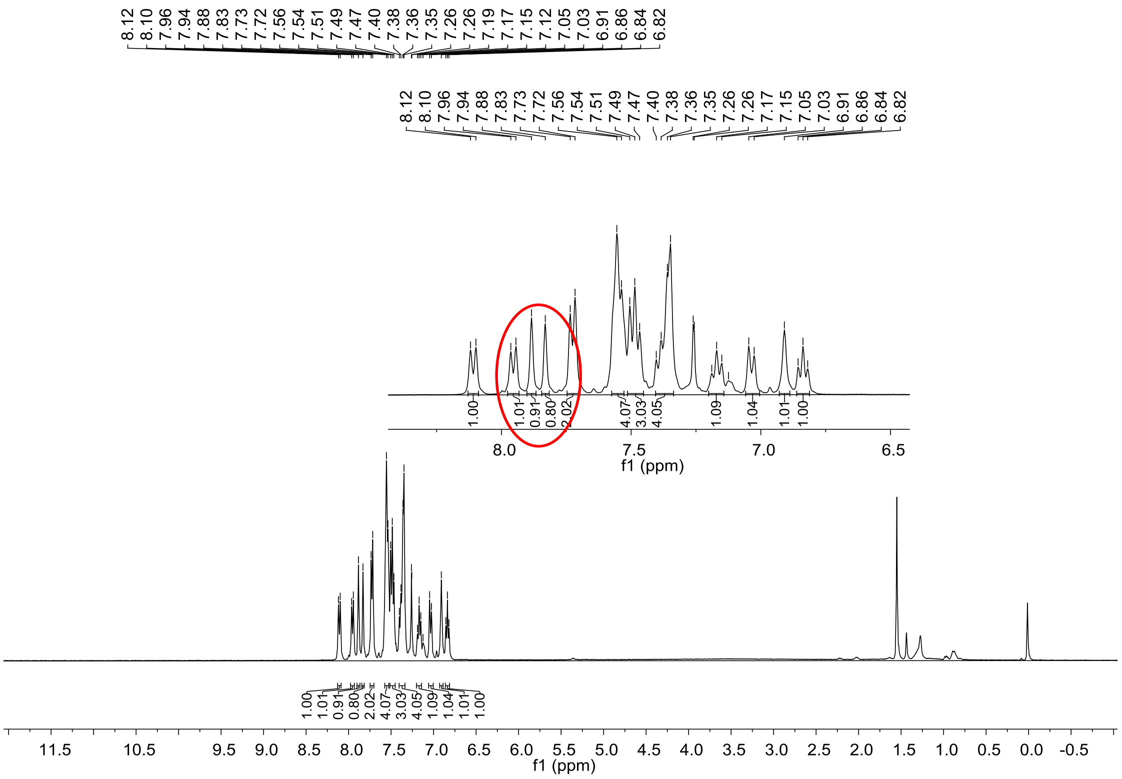

Scheme S8. Deuterium labeling experiments (Eq. M-Q)

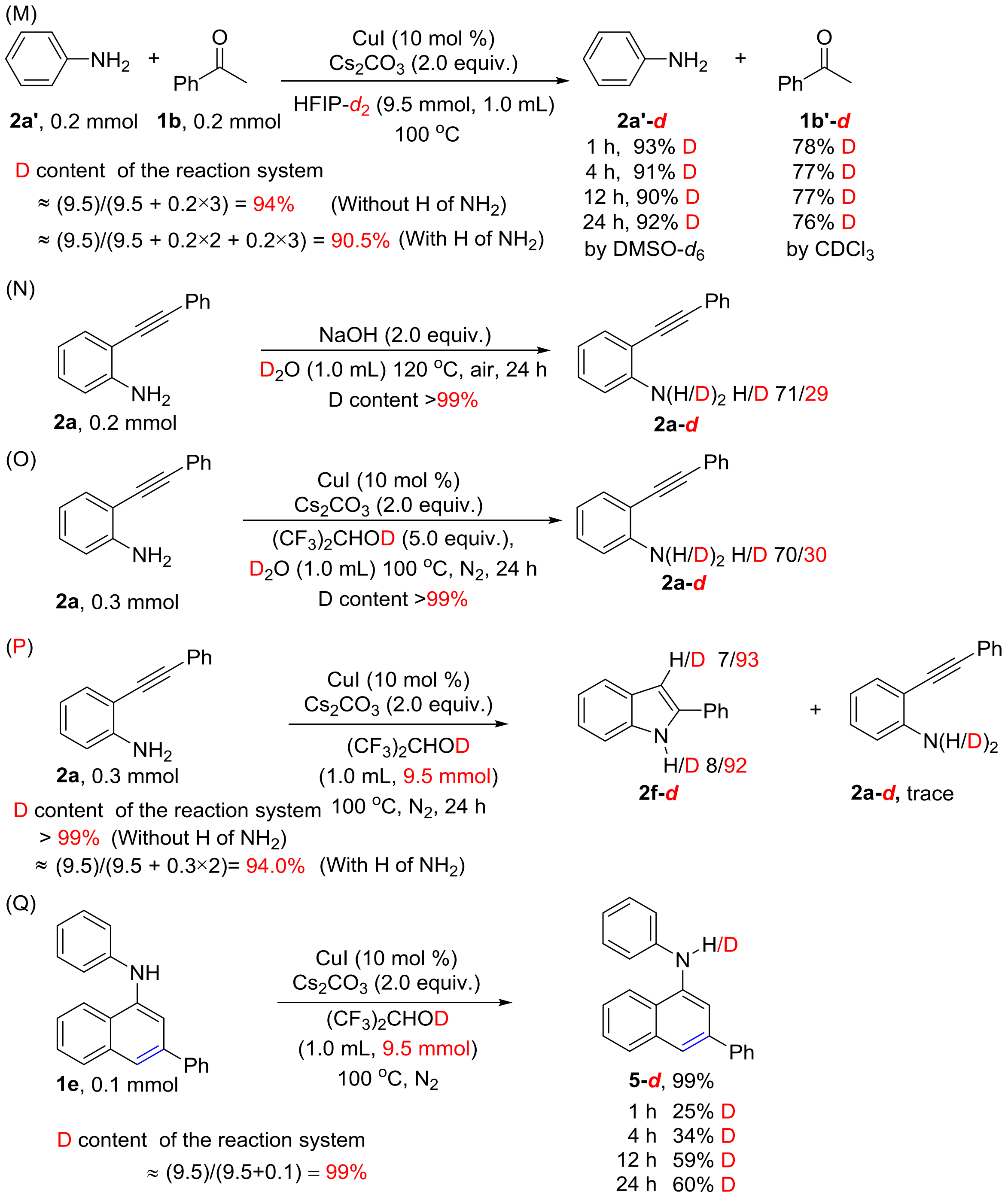

M) A Schlenk tube of 25 mL equipped with a magnetic stir bar was charged with CuI (3.8 mg, 0.02 mmol, 10 mol%), Cs_2CO_3 (132 mg, 0.4 mmol, 2.0 equiv.) in HFIP-d_2 (7.5 mmol), then, anilines **2a'** (0.2 mmol), and acetophenone **1b'** (0.2 mmol) were added. The reaction mixture was stirred (800 r/min in IKA RCT basic) at 100 ºC under N_2, and was extracted 100 ul to monitor by ^{1}H NMR at 0 h, 1 h, 4 h, 12 h, and 24 h respectively.

We can observed the change about methyl of ***1b'-d*** in $CDCl_3$, but the NH_2 of ***2a'-d*** cannot be identified, as supplementary, ^{1}H NMR in DMSO-d_6 gave the change of NH_2 of ***2a'-d***.

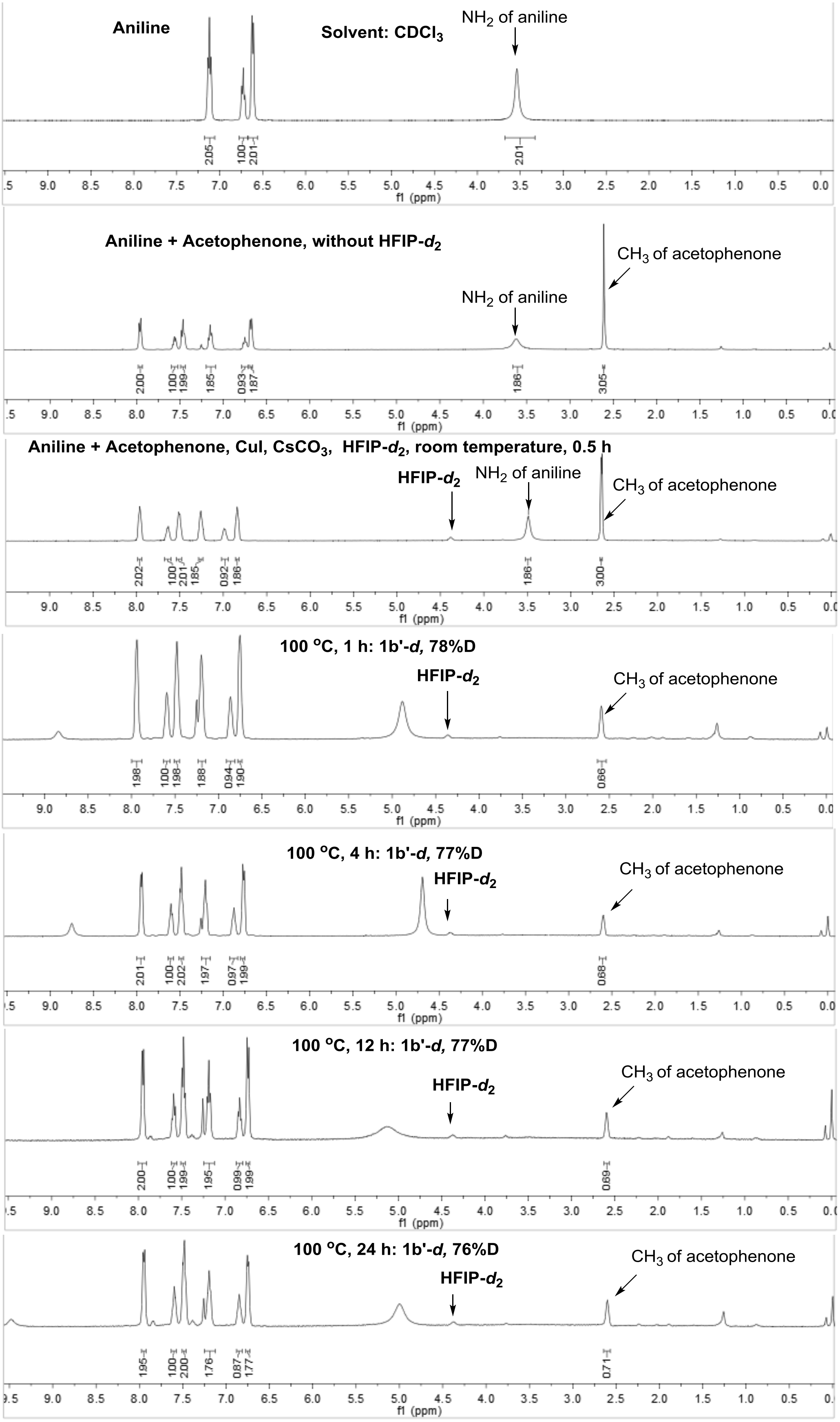

Figure 42. The ^{1}HNMR Spectrum of Eq. (M) in $CDCl_3$

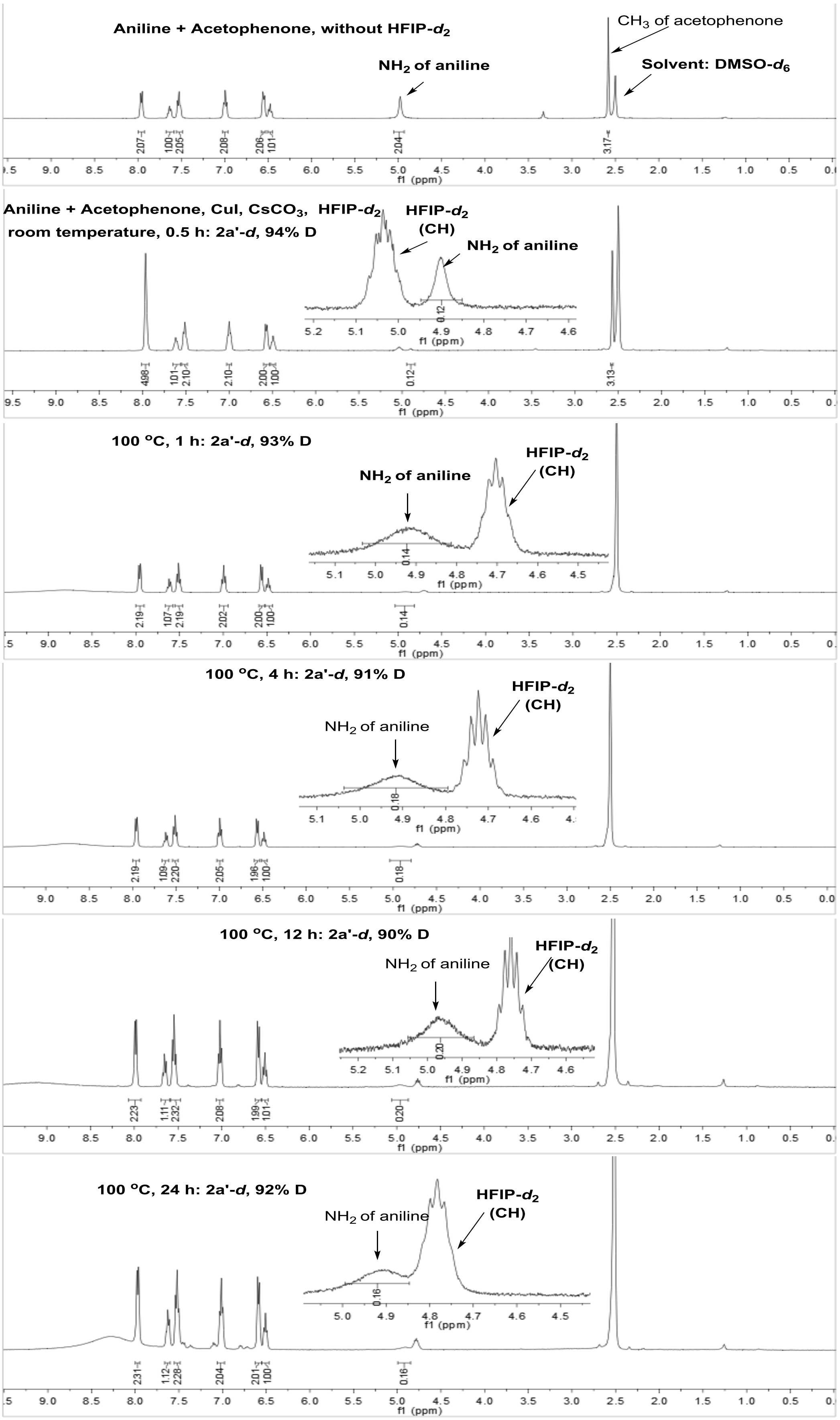

Figure S43. The ^{1}HNMR Spectrum of Eq. (M) in DMSO-d_6

N) A Schlenk tube of 25 mL equipped with a magnetic stir bar was charged with D_2O (1.0 mL), then, 2-(phenylethynyl)aniline **2a** (0.2 mmol) and NaOH (2.0 equiv.) were added. The reaction mixture was stirred (800 r/min in IKA RCT basic) at 100 ºC under N_2. After completion of the reaction, the reaction mixture was cooled to room temperature. The crude product was extracted by $CDCl_3$, for identifying by ^{1}HNMR directly without column chromatography.

2-(phenylethynyl)aniline (2a-*d*) (Eq. N)

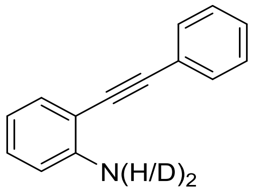

^{1}H NMR (400 MHz, $CDCl_3$) δ 7.58–7.49 (m, 2H), 7.42–7.30 (m, 4H), 7.15 (t, *J* = 7.6 Hz, 1H), 6.74 (d, *J* = 6.8 Hz, 2H), 4.28 (s, **1.42**H).

^{1}H NMR Spectrum of ***2a-d*** (Eq. N, 29% D)

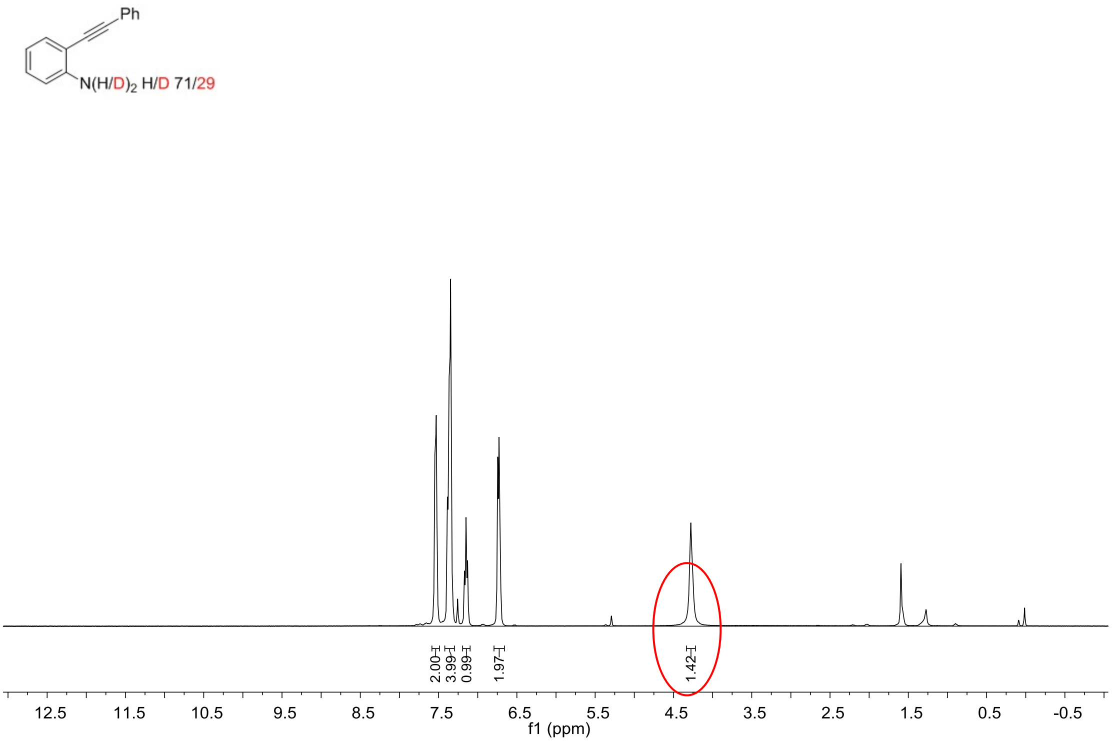

O) A Schlenk tube of 25 mL equipped with a magnetic stir bar was charged with CuI (5.7 mg, 0.03 mmol, 10 mol %), Cs_2CO_3 (195 mg, 0.6 mmol, 2.0 equiv.), HFIP-OD (1.5 mmol, 5.0 equiv.) in D_2O (1.0 mL), then, 2-(phenylethynyl)aniline **2a** (0.3 mmol) was added. The reaction mixture was stirred (800 r/min in IKA RCT basic) at 100 ºC for 24 h under N_2. After completion of the reaction, the reaction mixture was cooled to room temperature. The crude product was extracted by $CDCl_3$, for identifying by ^{1}HNMR directly without column chromatography.

2-(phenylethynyl)aniline (2a-*d*) (Eq. O)

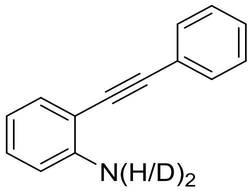

^{1}H NMR (400 MHz, $CDCl_3$) δ 7.58–7.50 (m, 2H), 7.41–7.31 (m, 4H), 7.15 (t, J = 7.7 Hz, 1H), 6.77–6.69 (m, 2H), 4.28 (s, **1.40H**).

^{1}H NMR Spectrum of ***2a-d*** (Eq. O, 30% D)

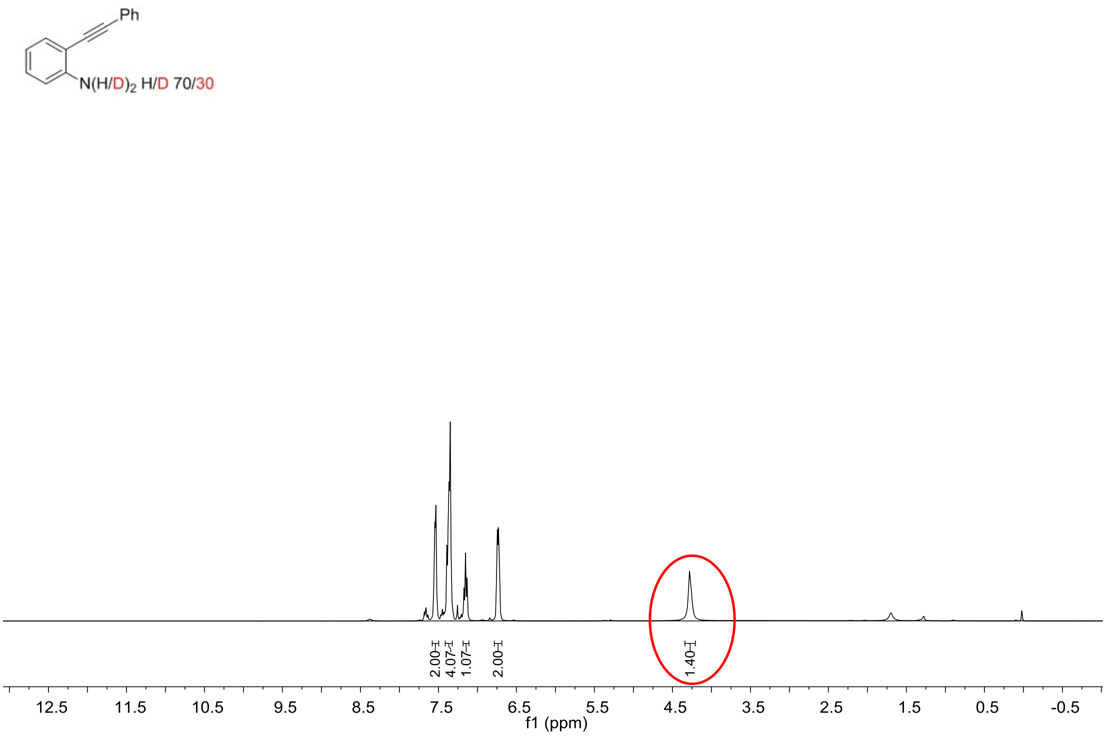

P) A Schlenk tube of 25 mL equipped with a magnetic stir bar was charged with CuI (5.7 mg, 0.03 mmol, 10 mol %), Cs_2CO_3 (195 mg, 0.6 mmol, 2.0 equiv.) in HFIP-OD (1.0 mL, 9.5 mmol), then, 2-(phenylethynyl)aniline **2a** (0.3 mmol) was added. The reaction mixture was stirred (800 r/min in IKA RCT basic) at 100 ºC for 24 h under N_2. After completion of the reaction, the reaction mixture was cooled to room temperature, the solvent was removed under reduced pressure, and the residue was extracted with $CDCl_3$, for identifying by ^{1}HNMR directly without column chromatography.

2-phenyl-1H-indole (2f-*d*) (Eq. P)

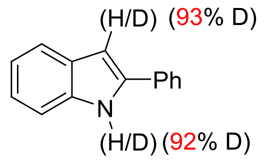

^{1}H NMR (400 MHz, $CDCl_3$) δ **8.38 (s, 0.08H)**, 7.66 (t, *J* = 9.3 Hz, 3H), 7.49–7.39 (m, 3H), 7.37–7.31 (m, 1H), 7.23–7.18 (m, 1H), 7.17–7.10 (m, 1H), **6.84 (s, 0.07H)**.

^{1}H NMR Spectrum of **2f-*d*** (Eq. P)

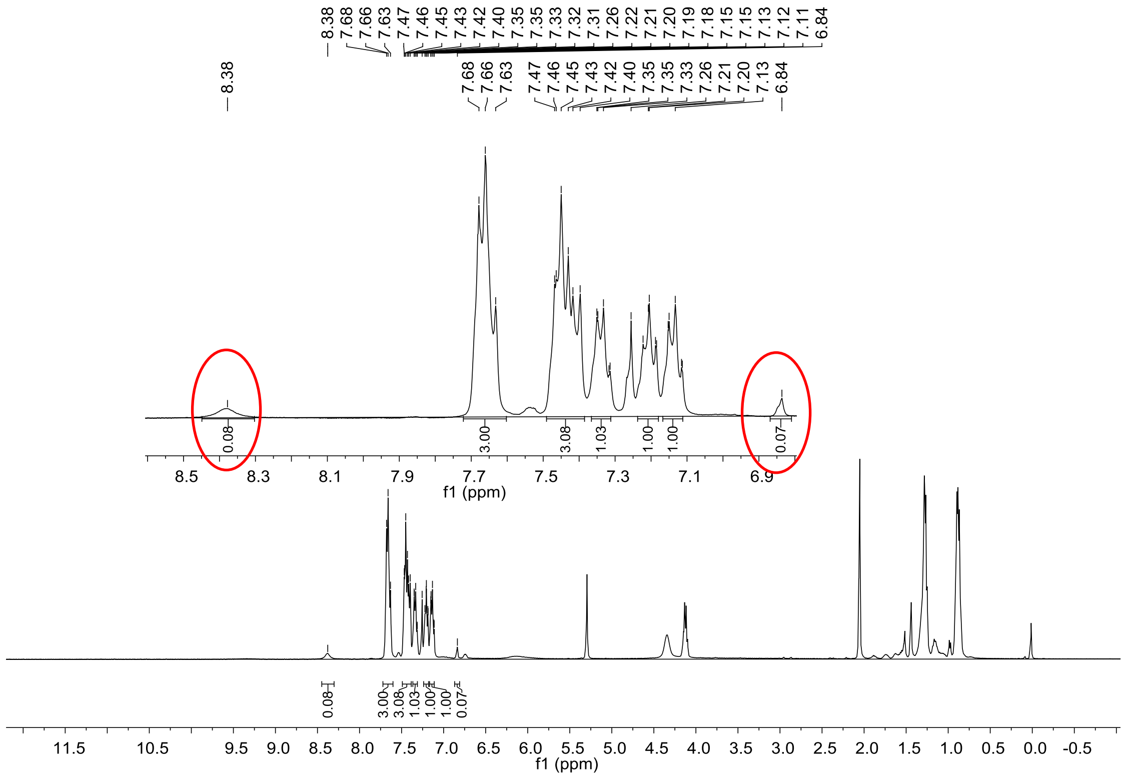

Q) A Schlenk tube of 25 mL equipped with a magnetic stir bar was charged with CuI (1.9 mg, 0.01 mmol, 10 mol %), Cs_2CO_3 (66 mg, 0.2 mmol, 2.0 equiv.) in HFIP-OD (1.0 mL, 9.5 mmol), then, **1e** (0.1 mmol) was added. The reaction mixture was stirred (800 r/min in IKA RCT basic) at 100 ºC under N_2, the reaction mixture was extracted 100 ul to monitor by ^{1}H NMR at 1 h, 4 h, 12 h, and 24 h respectively, the solvent was removed under reduced pressure, and the residue was extracted with $CDCl_3$, for identifying by ^{1}HNMR directly without column chromatography.

N,3-diphenylnaphthalen-1-amine (5-*d*, 1 h, Eq. Q**)**

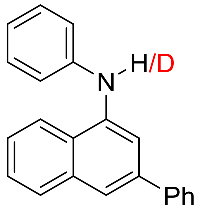

^{1}H NMR (400 MHz, $CDCl_3$) δ 8.02 (d, *J* = 8.2 Hz, 1H), 7.92 (d, *J* = 8.1 Hz, 1H), 7.77 (s, 1H), 7.67 (d, *J* = 7.6 Hz, 3H), 7.56–7.40 (m, 4H), 7.38–7.32 (m, 1H), 7.29 (d, *J* = 7.5 Hz, 2H), 7.07 (d, *J* = 7.8 Hz, 2H), 6.93 (t, *J* = 6.7 Hz, 1H), 6.02 (s, 0.75H).

N,3-diphenylnaphthalen-1-amine (**5-*d***, 4 h, Eq. Q)

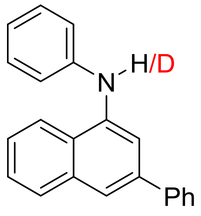

^{1}H NMR (400 MHz, $CDCl_3$) δ 8.03 (d, *J* = 8.2 Hz, 1H), 7.93 (d, *J* = 7.9 Hz, 1H), 7.78 (s, 1H), 7.69 (d, *J* = 7.7 Hz, 3H), 7.57–7.43 (m, 4H), 7.37 (t, *J* = 7.1 Hz, 1H), 7.30 (t, *J* = 7.5 Hz, 2H), 7.08 (d, *J* = 7.9 Hz, 2H), 6.95 (t, *J* = 7.1 Hz, 1H), 6.02 (s, 0.66H).

N,3-diphenylnaphthalen-1-amine (**5-*d***, 12 h, Eq. Q)

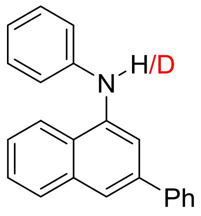

^{1}H NMR (400 MHz, $CDCl_3$) δ 7.99 (d, *J* = 7.5 Hz, 1H), 7.89 (d, *J* = 7.1 Hz, 1H), 7.74 (s, 1H), 7.64 (s, 3H), 7.53–7.39 (m, 4H), 7.33 (s, 1H), 7.30–7.18 (m, 4H), 7.04 (d, *J* = 7.2 Hz, 2H), 6.91 (s, 1H), 5.98 (s, 0.41H).

N,3-diphenylnaphthalen-1-amine (**5-*d***, 24 h, Eq. Q)

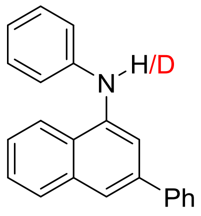

^{1}H NMR (400 MHz, $CDCl_3$) δ 8.03 (d, *J* = 8.1 Hz, 1H), 7.92 (d, *J* = 7.8 Hz, 1H), 7.78 (s, 1H), 7.68 (d, *J* = 7.5 Hz, 3H), 7.56– 7.42 (m, 4H), 7.37 (t, *J* = 7.1 Hz, 1H), 7.28 (dd, *J* = 14.0, 6.6 Hz, 2H), 7.07 (d, *J* = 7.6 Hz, 2H), 6.95 (t, *J* = 7.1 Hz, 1H), 6.00 (s, 0.40H).

^{1}H NMR Spectrum of **5-*d*** (1 h, Eq. Q)

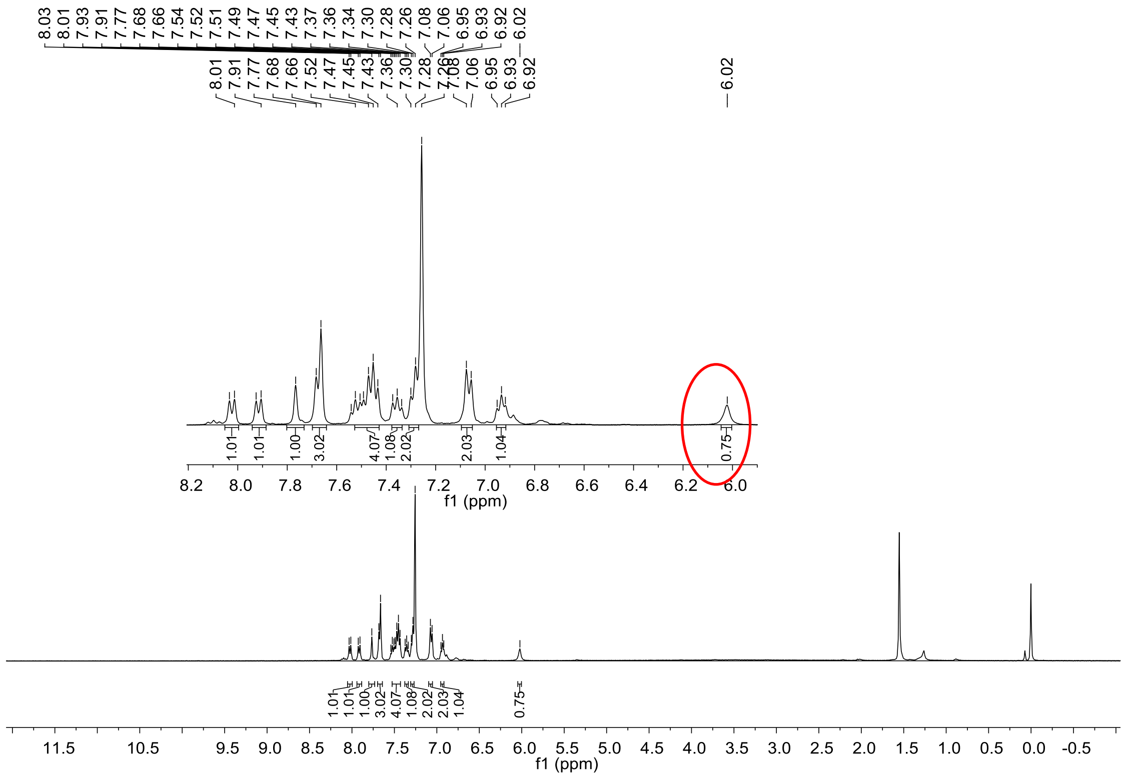

^{1}H NMR Spectrum of **5-*d*** (4 h, Eq. Q)

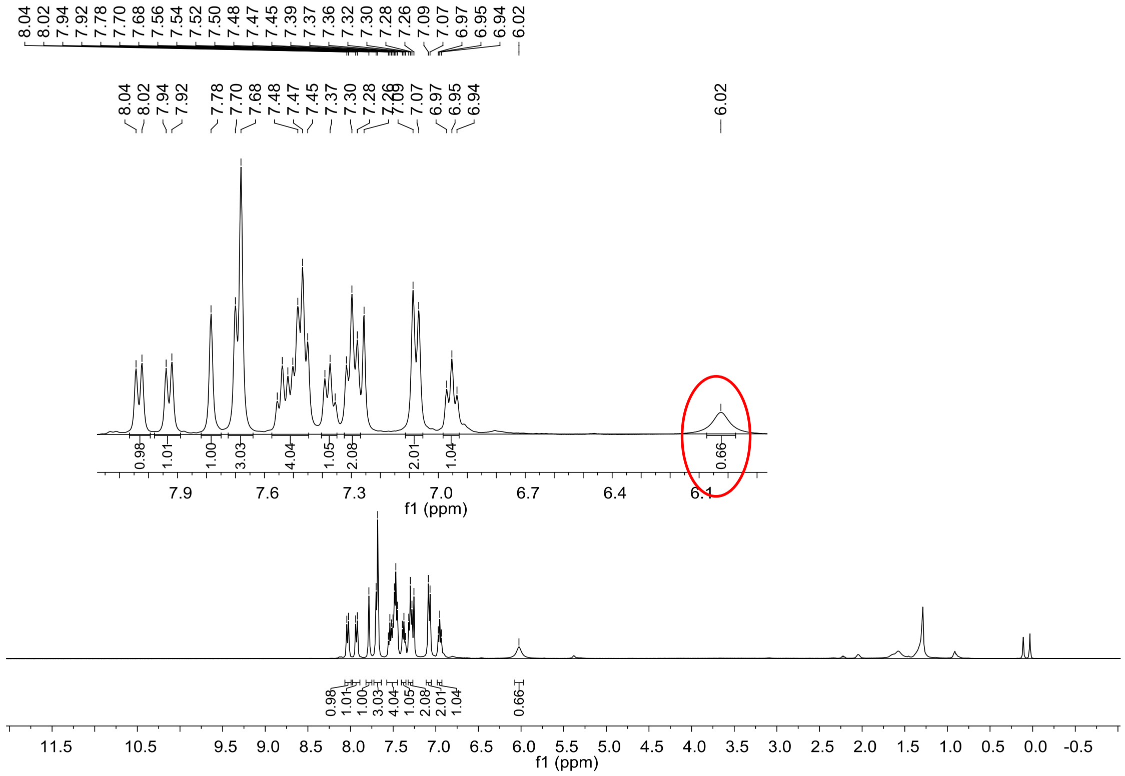

^{1}H NMR Spectrum of **5-*d*** (12 h, Eq. Q)

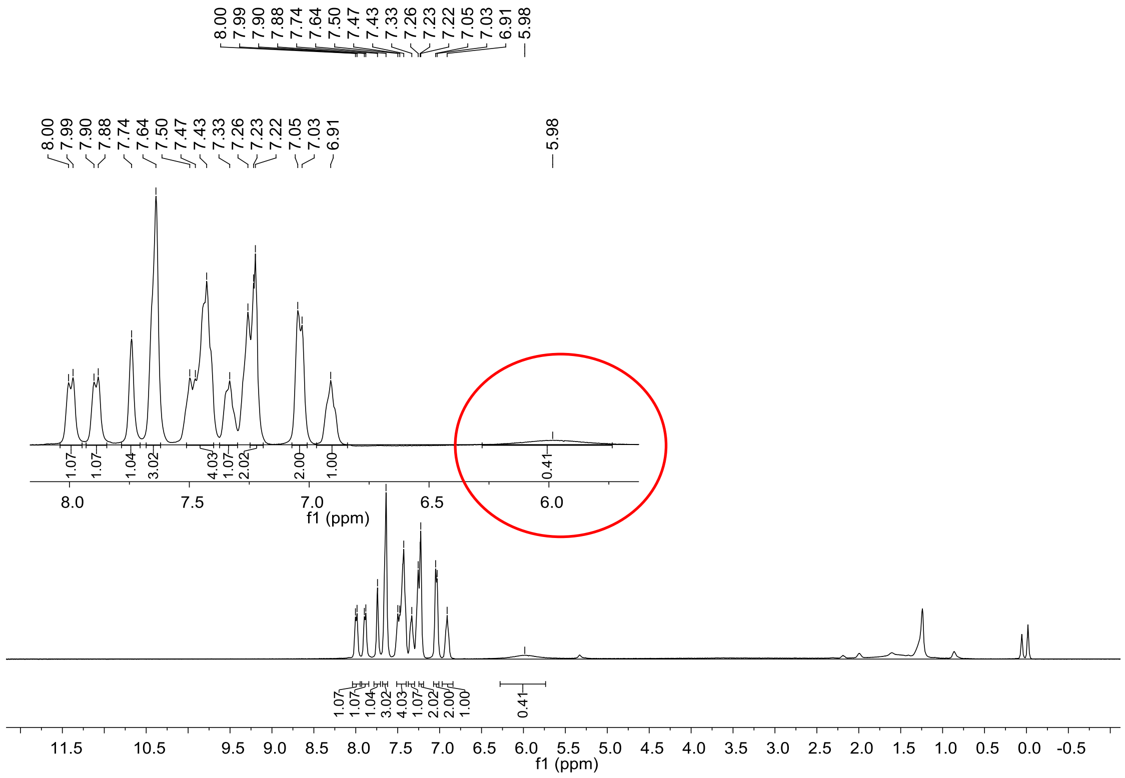

^{1}H NMR Spectrum of **5-*d*** (24 h, Eq. Q)

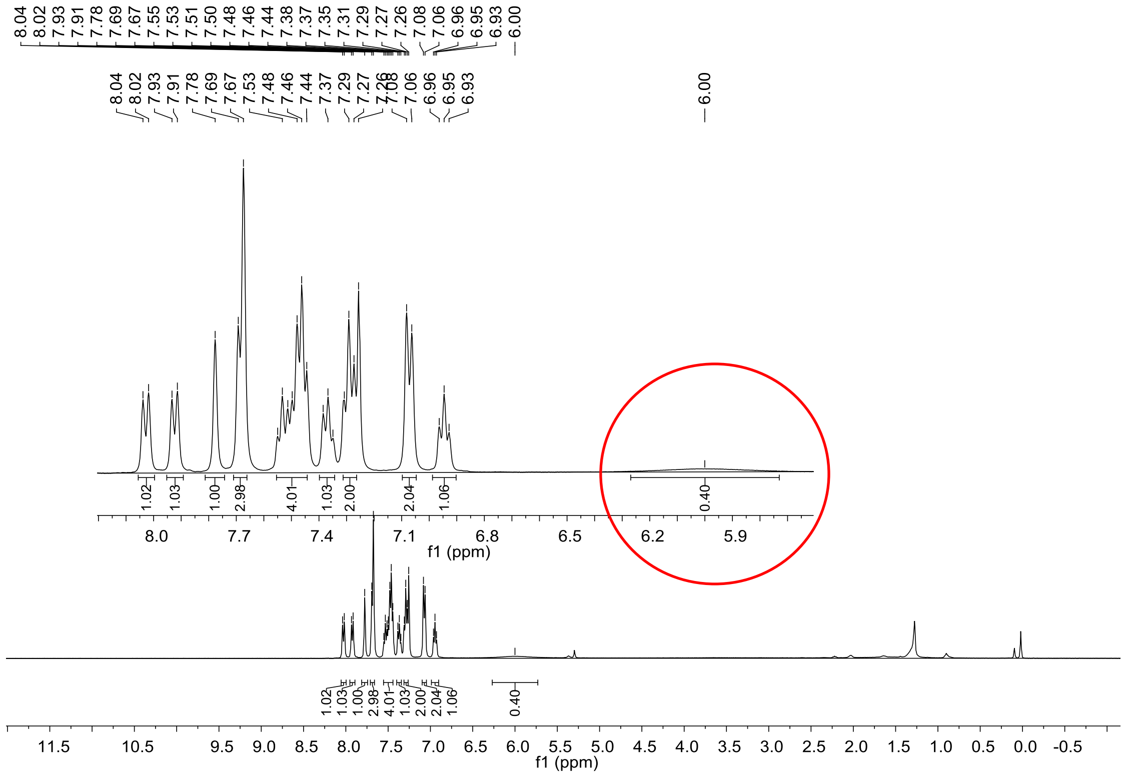

Scheme S9. Deuterium labeling experiments (Eq. R)

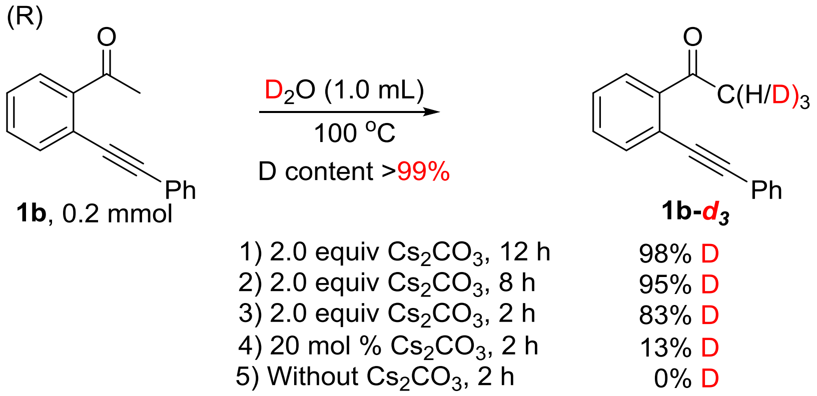

*The reactions of **1b** in D_2O under 2 equiv Cs_2CO_3, could give the **1b-d₃** with 98% deuteration of the methyl group, however, D_2O did not react with **1b** in absence of Cs_2CO_3, which indicated that the base could facilitate the keto-enol tautomerization. The above results illustrated that the reaction proceeded via keto-enol tautomerization, dehydration, and hydrolysis.*

R) A Schlenk tube of 25 mL equipped with a magnetic stir bar was charged with D_2O (1.0 mL); then, 2'-phenylethynylacetophenone **1b** (0.2 mmol) was added. The reaction mixture was stirred (800 r/min in IKA RCT basic) at 100 ºC under N_2. After completion of the reaction, the reaction mixture was cooled to room temperature. The crude product was purified by flash column chromatography on silica gel to give the desired **1b-d_3**.

2'-phenylethynylacetophenone (**1b-d_3**)

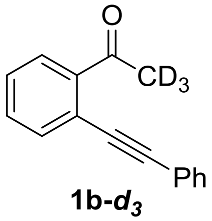

1b-d_3

(1) **2.0 equiv Cs_2CO_3, 12 h**. ^1H NMR (400 MHz, $CDCl_3$) δ 7.75 (d, *J* = 7.7 Hz, 1H), 7.63 (d, *J* = 7.7 Hz, 1H), 7.57–7.53 (m, 2H), 7.48 (t, *J* = 7.5 Hz, 1H), 7.41–7.35 (m, 4H).

(2) **2.0 equiv Cs_2CO_3, 8 h**. ^1H NMR (400 MHz, $CDCl_3$) δ 7.76 (d, *J* = 7.7 Hz, 1H), 7.63 (d, *J* = 7.6 Hz, 1H), 7.57–7.52 (m, 2H), 7.48 (t, *J* = 7.5 Hz, 1H), 7.42–7.34 (m, 4H), 2.72 (S, 0.15H).

(3) **2.0 equiv Cs_2CO_3, 4 h**. ^1H NMR (400 MHz, $CDCl_3$) δ 7.76 (d, *J* = 7.6 Hz, 1H), 7.63 (d, *J* = 7.5 Hz, 1H), 7.57–7.52 (m, 2H), 7.47 (t, *J* = 7.3 Hz, 1H), 7.42–7.34 (m, 4H), 2.81–2.72 (m, 0.5H).

(4) **20 mol % Cs_2CO_3, 2 h**. ^1H NMR (400 MHz, $CDCl_3$) δ 7.76 (d, *J* = 7.3 Hz, 1H), 7.63 (d, *J* = 7.2 Hz, 1H), 7.57–7.52 (m, 2H), 7.48 (t, *J* = 6.8 Hz, 1H), 7.43–7.33 (m, 4H), 2.80 (s, 2.60H).

(5) **Without Cs_2CO_3, 2 h**. ^1H NMR (400 MHz, $CDCl_3$) δ 7.75 (d, *J* = 7.3 Hz, 1H), (d, *J* = 7.2 Hz, 1H), 7.57–7.53 (m, 2H), 7.47 (t, *J* = 6.8 Hz, 1H)), 7.41–7.35 (m, 4H), 2.79 (s, 3H).

Scheme S10. The deuterium labeling experiments (Eq. S-V)

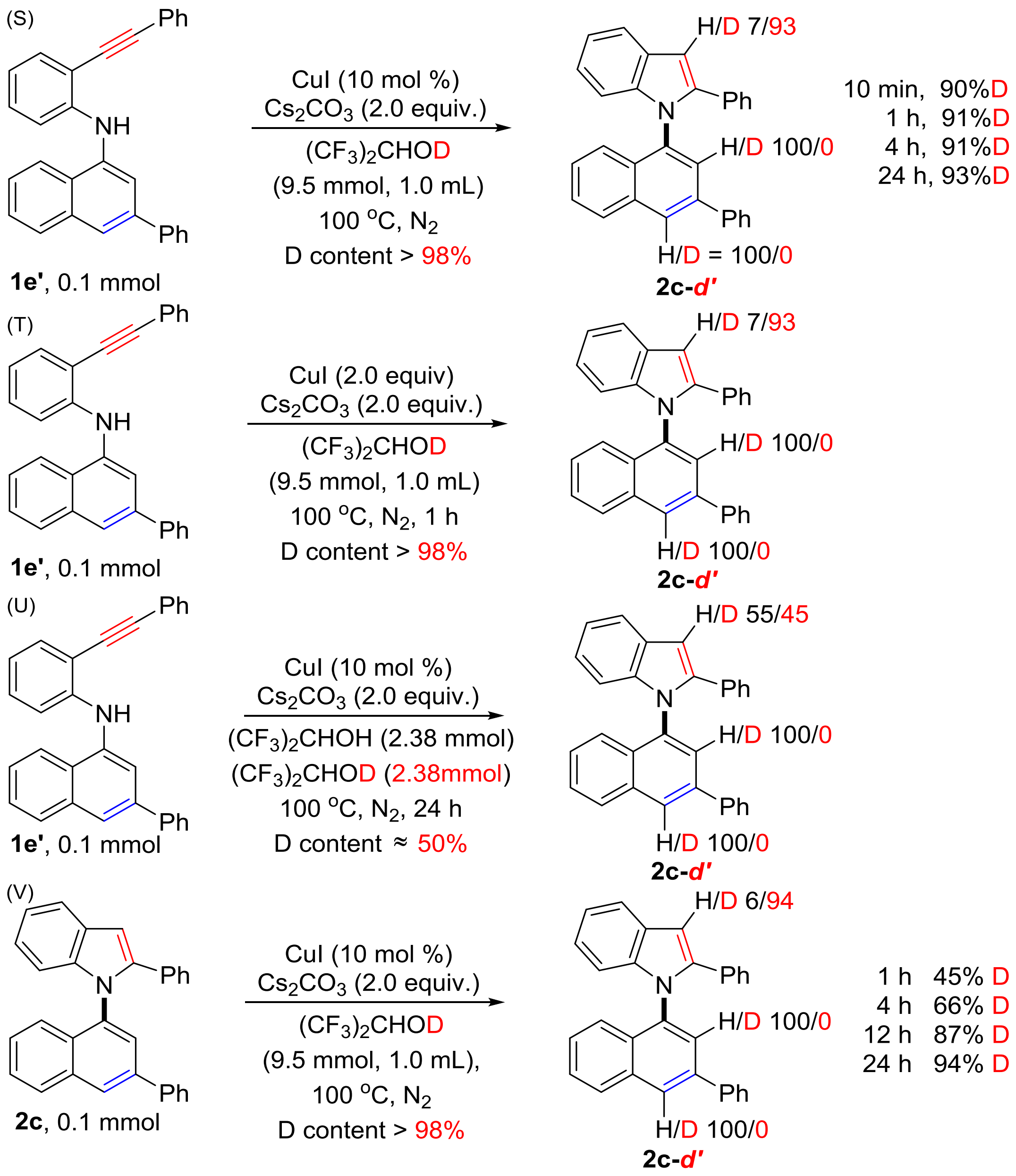

S) A Schlenk tube of 25 mL equipped with a magnetic stir bar was charged with CuI (1.9 mg, 0.01 mmol, 10 mol %), Cs_2CO_3 (66 mg, 0.2 mmol, 2.0 equiv.) in HFIP-OD (9.5 mmol), then, 3-Phenyl-N-(2-(phenylethynyl)phenyl)naphthalen-1-amine (**1e'**) (0.1 mmol) was added. The reaction mixture was stirred (800 r/min in IKA RCT basic) at 100 ºC, and was extracted 100 ul for monitoring by ^{1}H NMR at 10 min, 1 h, 4 h, and 24 h respectively, the solvent was removed under reduced pressure, and the residue was extracted with $CDCl_3$, for identifying by ^{1}HNMR directly without column chromatography.

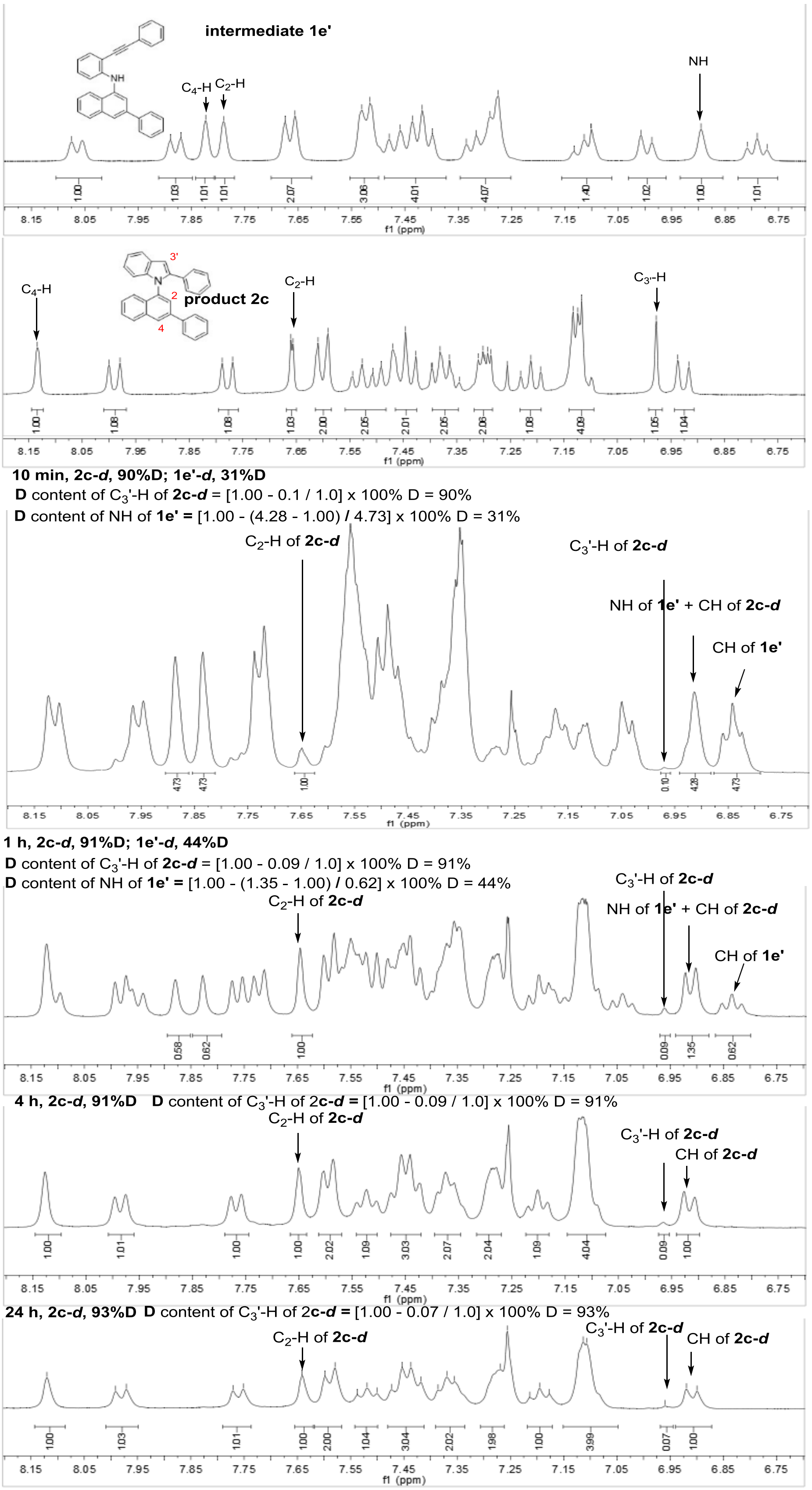

Calculation formula: D content of C3'-H of **2c-*d*** = [1.00 - (D of C3'-H of **2c-*d***) / (D of C2-H of **2c-*d***)] x 100% D

D content of NH of **1e'** = [1.00 - (D of (NH of **1e'** + CH of **2c-*d***) - D of C2-H of **2c-*d***) / (D of CH of **1e'**)] x 100% D

Figure S43. The ^{1}H NMR Spectrum of Eq. (S) at 10 min, 1 h, 2 h, and 24 h.

T) A Schlenk tube of 25 mL equipped with a magnetic stir bar was charged with CuI (38 mg, 0.2 mmol, 2.0 equiv.), Cs_2CO_3 (66 mg, 0.2 mmol, 2.0 equiv.) in HFIP-OD (1.0 mL, 9.5 mmol), then, 3-Phenyl-N-(2-(phenylethynyl)phenyl)naphthalen-1-amine (**1e'**, 0.1 mmol) was added. The reaction mixture was stirred (800 r/min in IKA RCT basic) at 100 °C for 1 h under N_2. After completion of the reaction, the reaction mixture was cooled to room temperature, the solvent was removed under reduced pressure, and the residue was extracted with $CDCl_3$, for identifying by ^{1}HNMR directly without column chromatography.

2-phenyl-1-(3-phenylnaphthalen-1-yl)-1H-indole (2c-*d'*, 1 h, Eq. T)

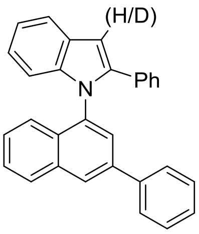

^{1}H NMR (400 MHz, $CDCl_3$) δ 8.13 (s, 1H), 7.98 (d, J = 8.0 Hz, 1H), 7.76 (d, J = 7.7 Hz, 1H), 7.65 (s, 1H), 7.59 (d, J = 7.1 Hz, 2H), 7.56–7.49 (m, 1H), 7.48–7.40 (m, 3H), 7.39–7.34 (m, 2H), 7.31–7.26 (m, 2H), 7.23–7.18 (m, 1H), 7.16–7.07 (m, 4H), 6.96 (s, 0.07H), 6.91 (d, J = 8.1 Hz, 1H).

^{1}H NMR Spectrum of **2c-*d'*** (1 h, Eq. T)

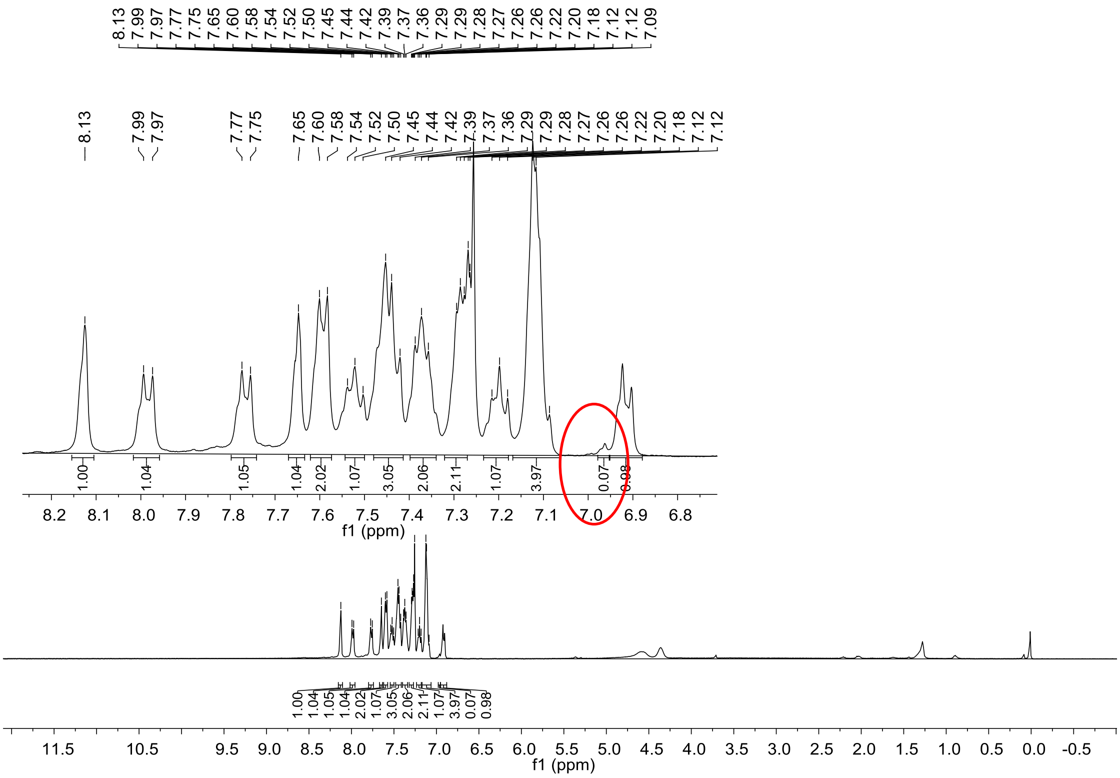

U) A Schlenk tube of 25 mL equipped with a magnetic stir bar was charged with CuI (1.9 mg, 0.01 mmol, 10 mol %), Cs_2CO_3 (66 mg, 0.2 mmol, 2.0 equiv.), HFIP-OD (2.38 mmol) in HFIP (2.38 mmol), then, 3-Phenyl-N-(2-(phenylethynyl)phenyl)naphthalen-1-amine (**1e'**) (0.1 mmol) was added. The reaction mixture was stirred (800 r/min in IKA RCT basic) at 100 ºC for 24 h under N_2. After completion of the reaction, the reaction mixture was cooled to room temperature, the solvent was removed under reduced pressure, and the residue was extracted with $CDCl_3$, for identifying by ^{1}HNMR directly without column chromatography.

2-phenyl-1-(3-phenylnaphthalen-1-yl)-1H-indole (Eq. U**, 2c-*d*'**, 24 h)

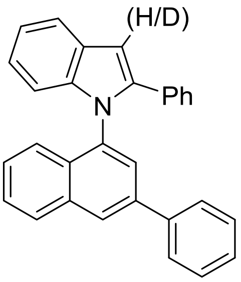

^{1}H NMR (400 MHz, $CDCl_3$) δ 8.12 (s, 1H), 7.98 (d, *J* = 7.7 Hz, 1H), 7.76 (d, *J* = 7.6 Hz, 1H), 7.64 (s, 1H), 7.59 (d, *J* = 7.3 Hz, 2H), 7.52 (t, *J* = 6.6 Hz, 1H), 7.49–7.40 (m, 3H), 7.36 (d, *J* = 4.7 Hz, 2H), 7.20 (t, *J* = 7.4 Hz, 1H), 7.11 (d, *J* = 2.9 Hz, 4H), 6.96 (s, 0.55H), 6.91 (d, *J* = 8.0 Hz, 1H).

^{1}H NMR Spectrum of **2c-*d'*** (24 h, Eq. U)

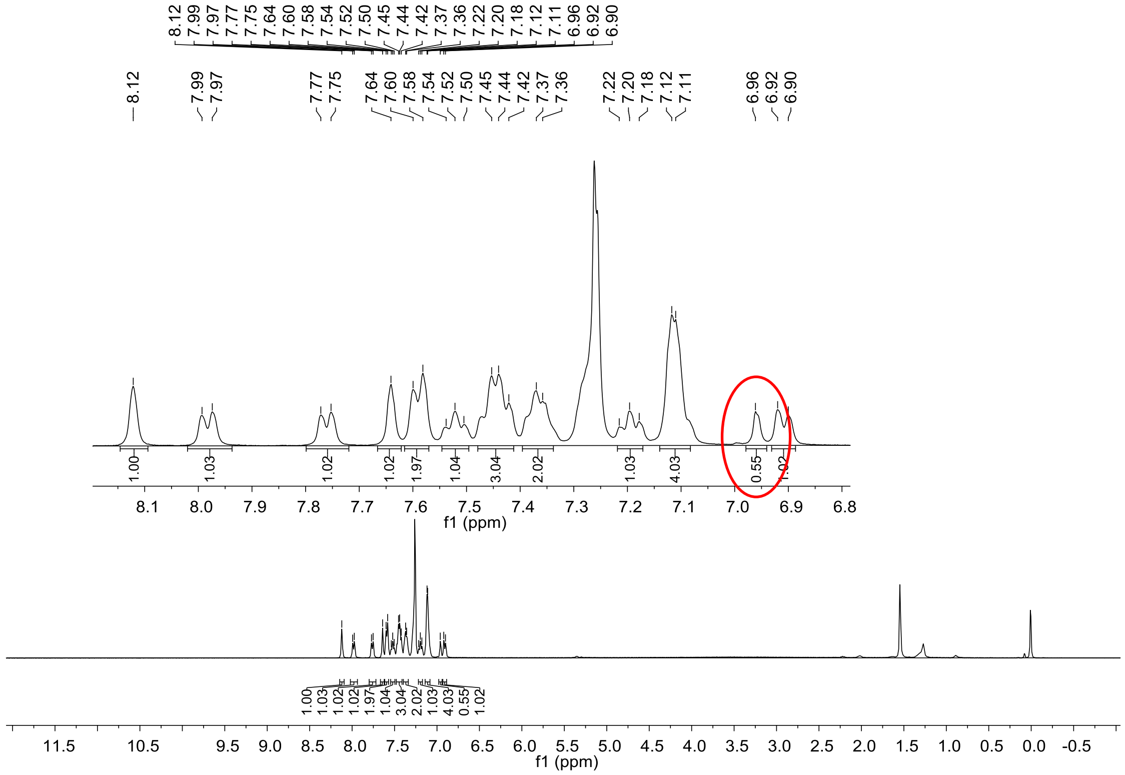

V) A Schlenk tube of 25 mL equipped with a magnetic stir bar was charged with CuI (1.9 mg, 0.01 mmol, 10 mol %), Cs_2CO_3 (66 mg, 0.2 mmol, 2.0 equiv.), in HFIP-OD (9.5 mmol), then, **2c** (0.1 mmol) was added. The reaction mixture was stirred (800 r/min in IKA RCT basic) at 100 ºC under N_2, and was extracted 100 ul to monitor by ^{1}H NMR at 1 h, 4 h, 12 h, and 24 h respectively, the solvent was removed under reduced pressure, and the residue was extracted with $CDCl_3$, for identifying by ^{1}HNMR directly without column chromatography.

2-phenyl-1-(3-phenylnaphthalen-1-yl)-1H-indole (2c-*d'*, Eq. V, 1 h**)**

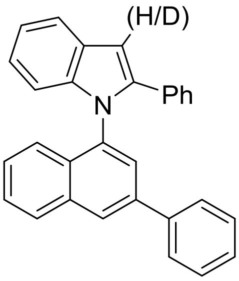

^{1}H NMR (400 MHz, $CDCl_3$) δ 8.15 (s, 1H), 8.00 (d, *J* = 8.0 Hz, 1H), 7.80 (d, *J* = 7.6 Hz, 1H), 7.67 (s, 1H), 7.61 (d, *J* = 6.8 Hz, 2H), 7.57–7.43 (m, 4H), 7.38 (d, *J* = 6.6 Hz, 2H), 7.31 (d, *J* = 3.2 Hz, 2H), 7.28–7.20 (m, 1H), 7.13 (s, 4H), 6.99 (s, 0.55H), 6.94 (d, *J* = 8.0 Hz, 1H).

2-phenyl-1-(3-phenylnaphthalen-1-yl)-1H-indole (2c-*d'*, Eq. V, 4 h**)**

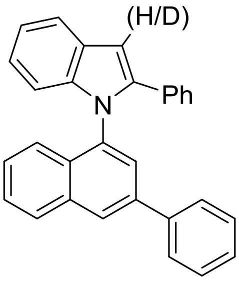

^{1}H NMR (400 MHz, $CDCl_3$) δ 8.15 (s, 1H), 8.01 (d, *J* = 8.1 Hz, 1H), 7.80 (d, *J* = 7.7 Hz, 1H), 7.67 (s, 1H), 7.62 (d, *J* = 7.5 Hz, 2H), 7.55 (t, *J* = 7.3 Hz, 1H), 7.48 (dd, *J* = 15.5, 8.0 Hz, 3H), 7.42–7.36 (m, 2H), 7.31 (d, *J* = 2.6 Hz, 2H), 7.23 (t, *J* = 7.3 Hz, 1H), 7.15 (d, *J* = 3.0 Hz, 4H), 6.99 (s, 0.34H), 6.94 (d, *J* = 8.1 Hz, 1H).

2-phenyl-1-(3-phenylnaphthalen-1-yl)-1H-indole (2c-*d'*, Eq. V, 12 h**)**

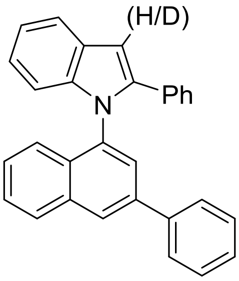

iii^{1}H NMR (400 MHz, $CDCl_3$) δ 8.12 (s, 1H), 7.98 (d, *J* = 7.7 Hz, 1H), 7.76 (d, *J* = 7.2 Hz, 1H), 7.64 (s, 1H), 7.59 (d, *J* = 6.8 Hz, 2H), 7.51 (d, *J* = 6.5 Hz, 1H), 7.48–7.41 (m, 3H), 7.39–7.33 (m, 2H), 7.31–7.26 (m, 2H), 7.22–7.17 (m, 1H) (d, *J* = 6.8 Hz, 1H), 7.14–7.07 (m, 4H), 6.96 (s, 0.13H), 6.91 (d, *J* = 7.6 Hz, 1H).

2-phenyl-1-(3-phenylnaphthalen-1-yl)-1H-indole (2c-*d'*, Eq. V, 24 h**)**

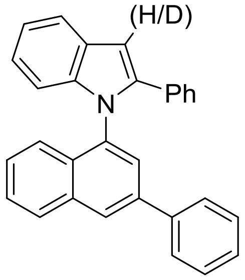

^{1}H NMR (400 MHz, $CDCl_3$) δ 8.13 (s, 1H), 7.98 (d, *J* = 8.2 Hz, 1H), 7.77 (d, *J* = 7.8 Hz, 1H), 7.65 (s, 1H), 7.59 (d, *J* = 7.3 Hz, 2H), 7.52 (t, *J* = 7.4 Hz, 1H), 7.48–7.40 (m, 3H), 7.40–7.33 (m, 2H), 7.32–7.27 (m, 2H), 7.20 (t, *J* = 7.4 Hz, 1H), 7.15–7.07 (m, 4H), 6.96 (s, 0.06H), 6.92 (d, *J* = 8.2 Hz, 1H).

^{1}H NMR Spectrum of **2c-*d'*** (1 h, Eq. V)

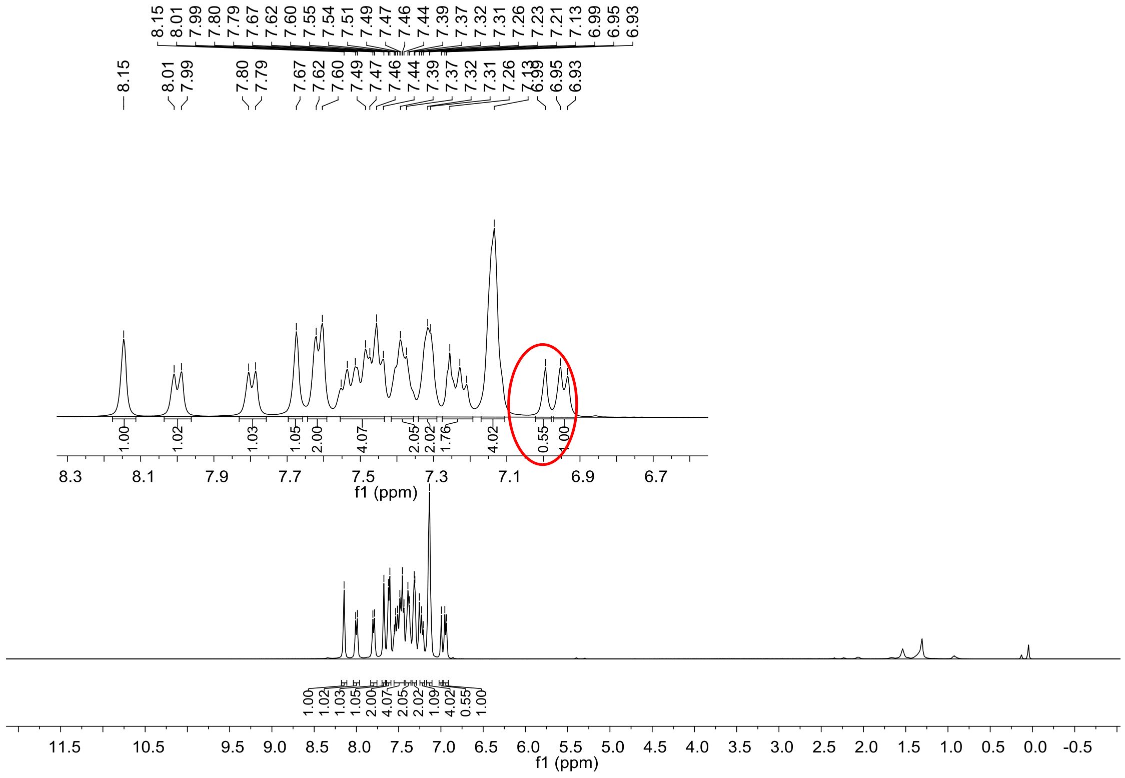

^{1}H NMR Spectrum of **2c-*d'*** (4 h, Eq. V)

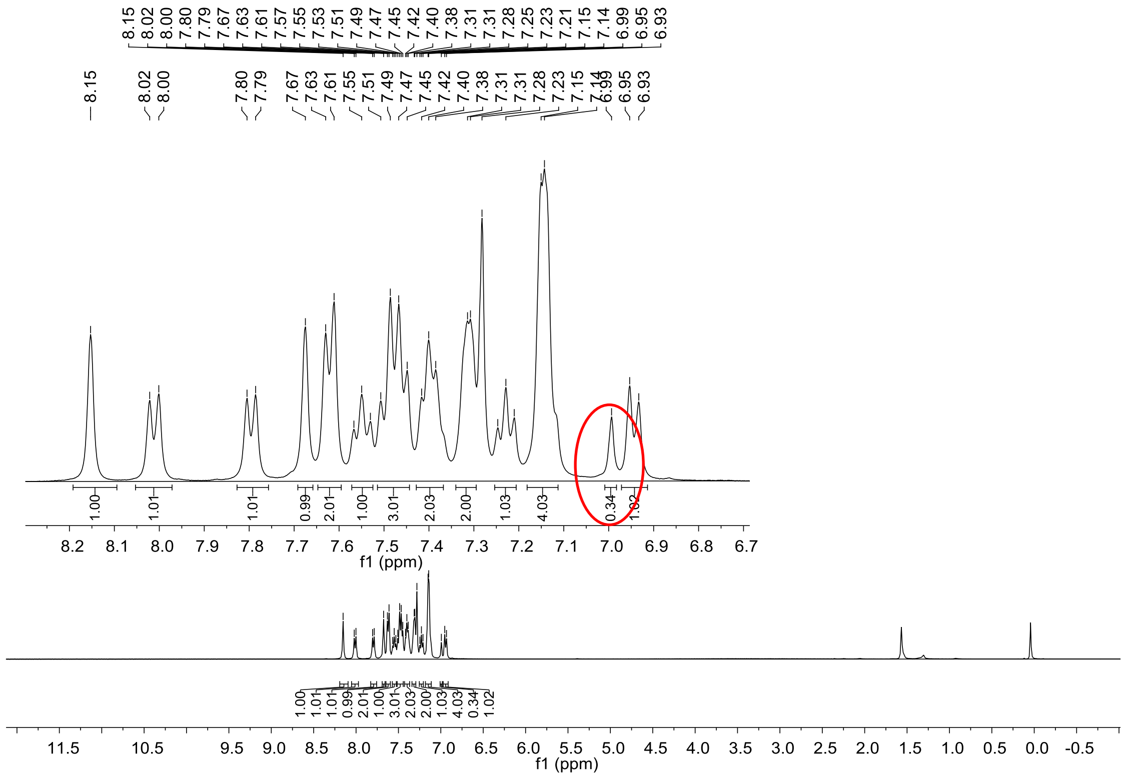

^{1}H NMR Spectrum of **2c-*d'*** (12 h, Eq. V)

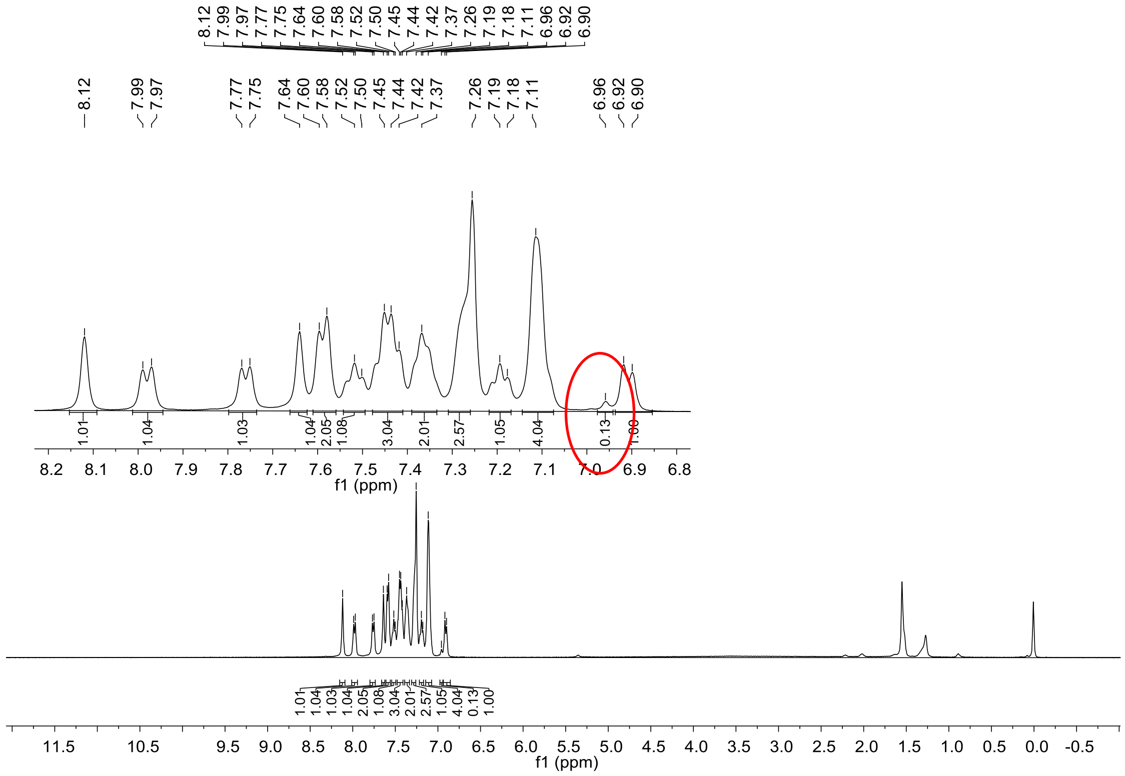

^{1}H NMR Spectrum of **2c-*d'*** (24 h, Eq. V)

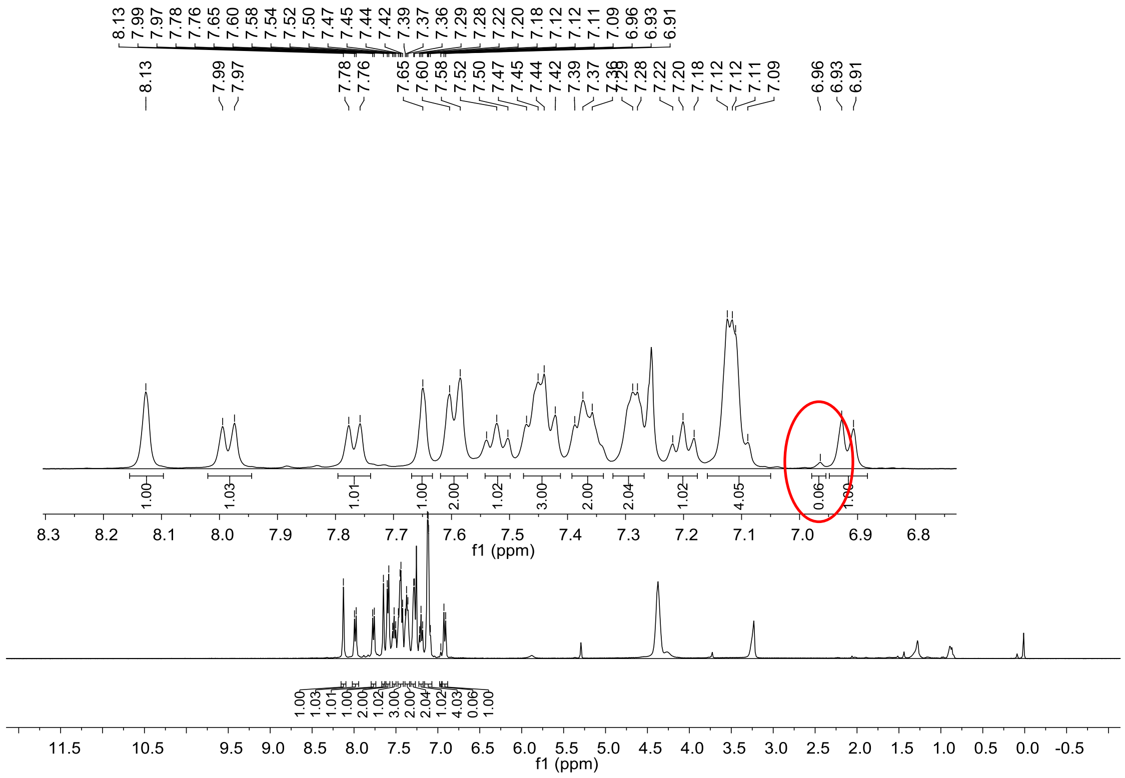

5. Asymmetric Synthesis.

Table S9. Optimization of the Conditions (Ligands and temperature)[a]

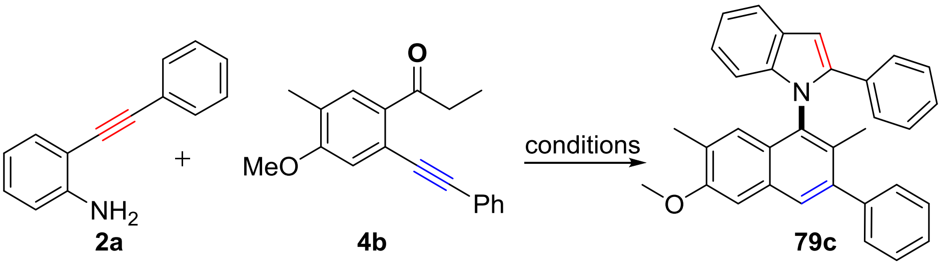

Entry	Ligand	T(°C)	t(h)	yield	ee
1	L1	40	72	31%	6%
2	L2	40	72	33%	23%
3	L3	40	72	30%	3%
4	L4	40	72	20%	11%
5	L5	40	72	17%	3%
6	L6	40	72	26%	5%
7	L7	40	72	22%	3%
8	L8	40	72	20%	2%
9	L9	40	72	18%	1%
10	L10	40	72	29%	4%
11	L11	40	72	19%	5%
12	L12	40	72	18%	5%
13	L13	40	72	25%	-9%
14	L14	40	72	30%	16%
15	L15	40	72	8%	-3%
16	L16	40	72	10%	4%
17	L17	40	72	8%	2%
18	L18	40	72	8%	8%
19	L19	40	72	8%	4%
20	L2	30	72	25%	21%
21	L2	50	72	40%	17%
22	L2	60	72	55%	15%
23	L2	70	72	67%	10%
24	L2	80	72	80%	7%
25	L2	90	72	88%	5%

[a]Reaction conditions: unless otherwise stated, all reactions were performed with **2a** (0.05 mmol), **4b** (0.06 mmol), CuI (5 mol%), Ligand (6 mol%), base (2.0 equiv, 0.1 mmol) in the solvent (0.5 mL) under N_2 protection. [b]GC yield based on **2a** using dodecane as internal standard.

Table S10. Optimization of the Conditions (catalysts, ligands, temperature, and time)[a]

Entry	Step 1	Step 2	yield	ee
1	CuI, 30 min + 100 °C	L1, 72 h + 40 °C	39%	8%
2	CuI, 30 min + 100 °C	L2, 72 h + 40 °C	42%	20%
3	CuI, 30 min + 100 °C	L4, 72 h + 40 °C	41%	12%
4	CuI, 30 min + 100 °C	L13, 72 h + 40 °C	42%	-10%
5	CuI, 30 min + 100 °C	L14, 72 h + 40 °C	40%	12%
6	CuI, 30 min + 100 °C	$Pd(OAc)_2$ + L2, 72 h + 40 °C	45%	8%
7	CuI, 30 min + 100 °C	$PdCl_2$ + L2, 72 h + 40 °C	48%	12%
8	CuI, 30 min + 100 °C	$Pd_2(dba)_3$ + L2, 72 h + 40 °C	44%	4%
9	CuI, 30 min + 100 °C	$Pd(PPh_3)_4$ + L2, 72 h + 40 °C	40%	6%
10	CuI, 30 min + 100 °C	$Pd(PPh_3)_2Cl_2$ + L2, 72 h + 40 °C	45%	2%
11	CuI, 30 min + 100 °C	$PdCl_2$ + L18, 72 h + 40 °C	52%	34%
12	CuI, 30 min + 100 °C	$PdCl_2$ + L19, 72 h + 40 °C	49%	16%
13	CuI, 30 min + 100 °C	$Pd(OAc)_2$ + L18, 72 h + 40 °C	44%	10%
14	CuI, 30 min + 100 °C	$Pd(OAc)_2$ + L19, 72 h + 40 °C	45%	12%
15	CuI, 30 min + 100 °C	$PdCl_2$ + L18, 72 h + 50 °C	55%	32%
16	CuI, 30 min + 100 °C	$PdCl_2$ + L18, 72 h + 60 °C	58%	29%
17	CuI, 30 min + 100 °C	$PdCl_2$ + L18, 72 h + 70 °C	58%	32%
18	CuI, 30 min + 100 °C	$PdCl_2$ + L18, 72 h + 80 °C	81%	33%
19	CuI, 30 min + 100 °C	$PdCl_2$ + L18, 24 h + 80 °C	80%	34%
20	CuI, 30 min + 100 °C	$PdCl_2$ + L18, 24 h + 90 °C	84%	31%
21	CuI, 30 min + 100 °C	$PdCl_2$ + L18, 24 h + 100 °C	88%	28%
22	CuI, 30 min + 90 °C	$PdCl_2$ + L18, 24 h + 80 °C	86%	32%
23	CuI, 30 min + 80 °C	$PdCl_2$ + L18, 24 h + 80 °C	87%	36%
24	CuI, 30 min + 70 °C	$PdCl_2$ + L18, 24 h + 80 °C	87%	34%
25	CuI, 30 min + 60 °C	$PdCl_2$ + L18, 24 h + 80 °C	82%	30%
26	CuI, 0 min + 80 °C	$PdCl_2$ + L18, 24 h + 80 °C	83%	20%
27	CuI, 10 min + 80 °C	$PdCl_2$ + L18, 24 h + 80 °C	82%	24%
28	CuI, 20 min + 80 °C	$PdCl_2$ + L18, 24 h + 80 °C	84%	28%
29	CuI, 40 min + 80 °C	$PdCl_2$ + L18, 24 h + 80 °C	85%	34%
30	CuI, 50 min + 80 °C	$PdCl_2$ + L18, 24 h + 80 °C	85%	38%
31	CuI, 60 min + 80 °C	$PdCl_2$ + L18, 24 h + 80 °C	86%	36%
32	CuI, 70 min + 80 °C	$PdCl_2$ + L18, 24 h + 80 °C	88%	35%
33	CuI, 50 min + 80 °C	$PdCl_2$ + L18, 20 h + 80 °C	86%	36%
34	CuI, 50 min + 80 °C	$PdCl_2$ + L18, 15 h + 80 °C	85%	40%
35	CuI, 50 min + 80 °C	$PdCl_2$ + L18, 10 h + 80 °C	76%	32%
36	CuI, 50 min + 80 °C	$PdCl_2$ + L18, 5 h + 80 °C	70%	26%
37[c]	CuI, 50 min + 80 °C	$PdCl_2$ + L18, 15 h + 80 °C	85%	38%
38[d]	CuI, 50 min + 80 °C	$PdCl_2$ + L18, 15 h + 80 °C	85%	40%

[a]GC yield based on **2a** using dodecane as internal standard. [b] Step 1: **2a** (0.05 mmol), **4b** (0.06 mmol), CuI (5 mol %), Cs_2CO_3 (2.0 equiv, 0.1 mmol) in HFIP (0.5 mL) under N_2 protection, Step 2: Pd catalyst (5 mol%) and Ligand (6 mol%) were added in the shelenk tube. [c] Ligand (5 mol%). [d] Ligand (7 mol%) .

Table S11. Further Optimization of the Reaction Conditions (P ligand)[a]

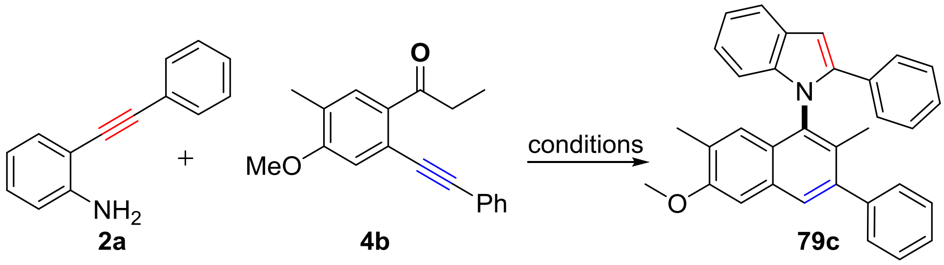

Entry	Step 1	Step 2	yield	ee
1	CuI, 50 min + 80 °C	L20, 15 h + 80 °C	86%	36%
2	CuI, 50 min + 80 °C	L21, 15 h + 80 °C	82%	32%

Figure S45. The chiral ligands in Table S9, S10 and S11

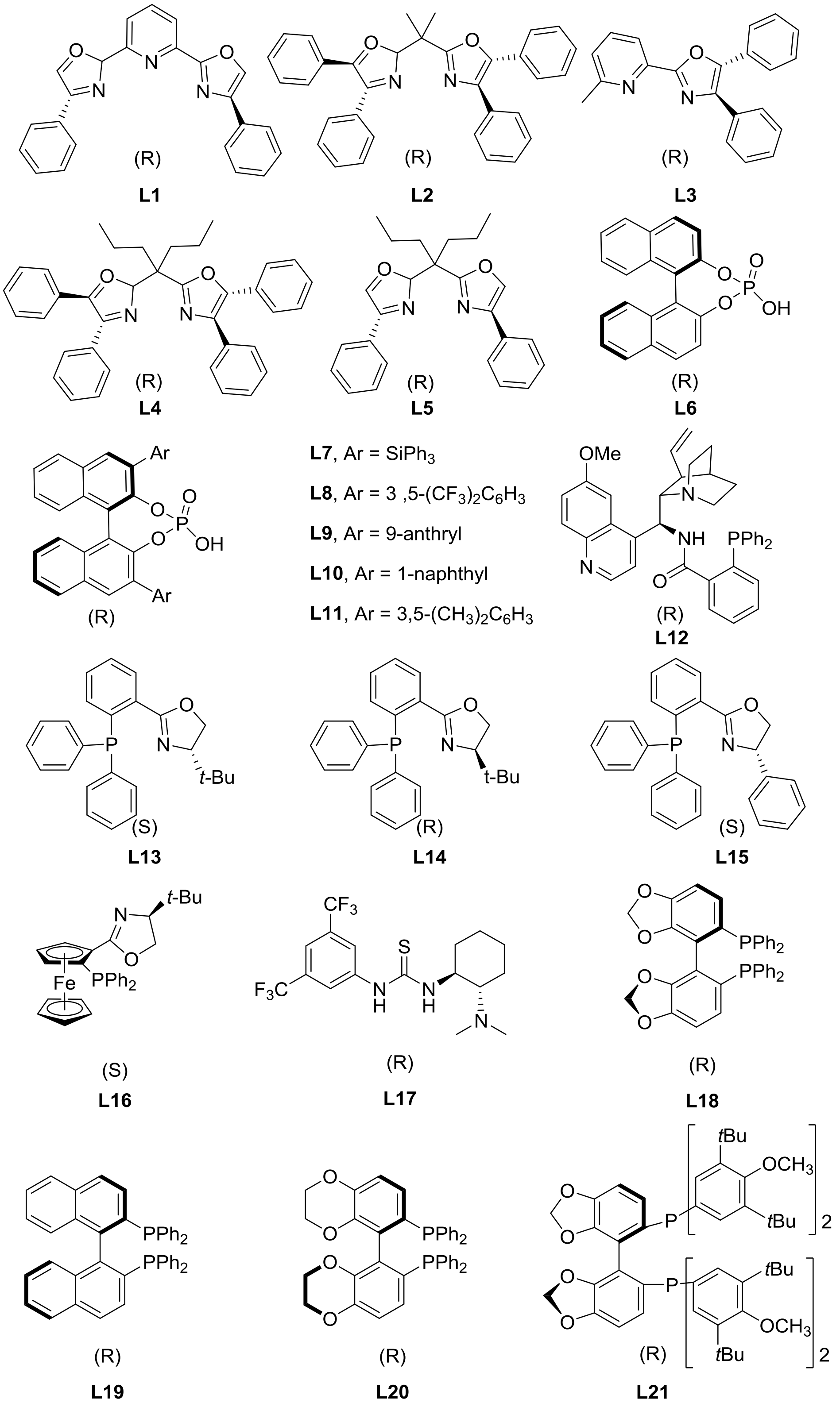

Scheme S11. The control experiment of the asymmetric synthesis of axially chiral naphthylindoles

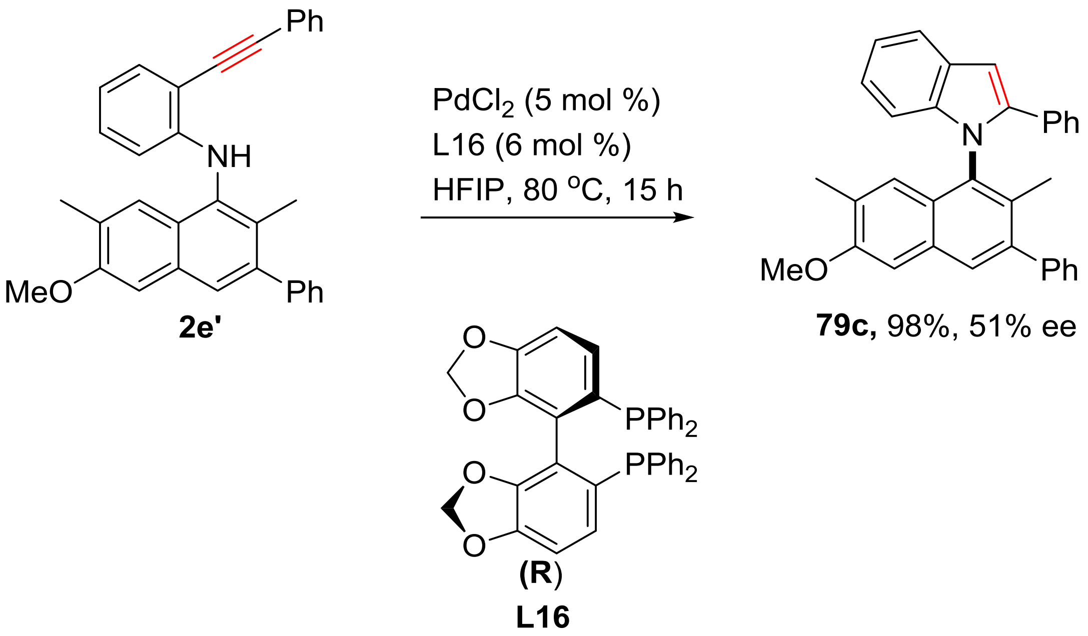

Enantioselective of Table S9, entry 2.

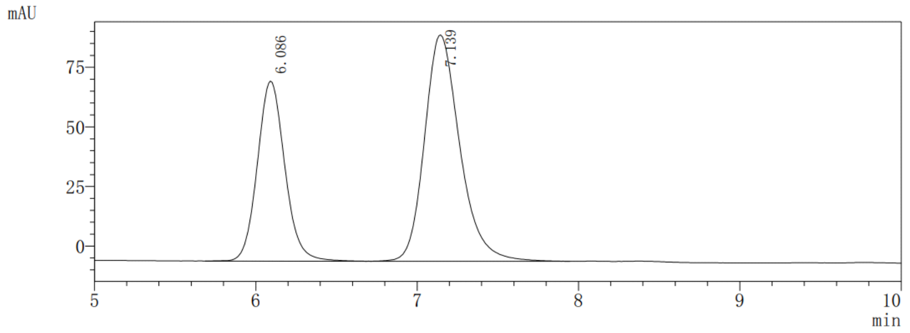

PDA Ch1 254nm 4nm

Peak	Ret Time	Width	Area	Height	Area %
1	6.086	0.318	893023	75587	38.135
2	7.139	0.399	1448740	95000	61.865

Enantioselective of Table S10, entry 38.

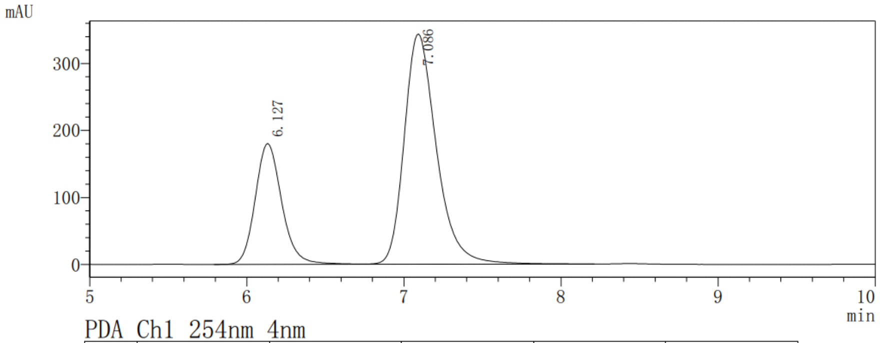

PDA Ch1 254nm 4nm

Peak	Ret Time	Width	Area	Height	Area %
1	6.127	0.314	2111986	180253	30.099
2	7.086	0.374	4904769	343611	69.901

Enantioselective of Scheme S11.

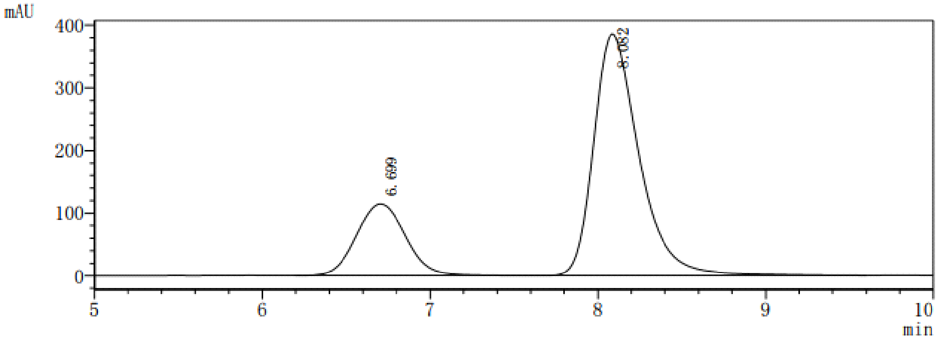

PDA Ch1 254nm 4nm

Peak	Ret Time	Width	Area	Height	Area %
1	6.699	0.538	2338066	114525	24.359
2	8.082	0.485	7260279	385690	75.641

6. MTT assay and Enzyme-linked immunosorbent assay

6.1 MTT assay

Cell viability was determined by the MTT method following the manufacturer's instructions. In all assays, the compounds were quickly dissolved in DMSO and diluted in sterile culture medium before being added to the cell culture (DMSO concentration < 0.1% (v/v)). Microglial BV-2 cells were seeded in 96-well plates at a density of 1×10^4 cells/well. After 12 h, compounds of designated concentrations (3, 10, 30 and 100 μM) were added. After 24 h, cells were incubated with culture medium containing 10 μl of 5 mg/ml MTT solution (Sigma-Aldrich) for 4 h at 37 °C. Plates were then centrifuged to remove the supernatants, and the crystals were dissolved in 150 μL of DMSO. The spectrophotometrical absorbance of the samples was measured using a quantified microplate reader at a wavelength of 490 nm.

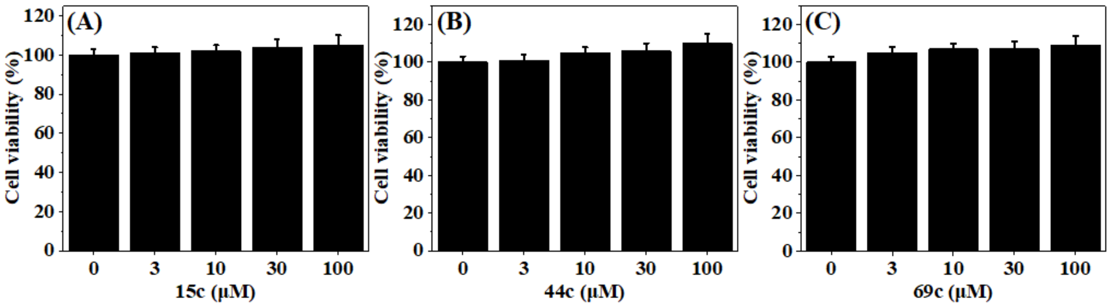

Figure S46. A) Cell viability was assessed by MTT assay in BV-2 cells after treatment with various concentrations of compound **15c** (0,3,10 ,30 and 100 μM) for 24 h. Each experiment was repeated three times in triplicate. Statistical analysis was performed using one-way analysis of variance (ANOVA) with Dunnett' post hoc test. B) Cell viability was assessed by MTT assay in BV-2 cells after treatment with various concentrations of compound **44c** (0, 3,10 ,30 and 100 μM) for 24 h. Each experiment was repeated three times in triplicate. Statistical analysis was performed using one-way analysis of variance (ANOVA) with Dunnett' post hoc test. C) Cell viability was assessed by MTT assay in BV-2 cells after treatment with various concentrations of compound **69c** (0, 3,10 ,30 and 100 μM) for 24 h. Each experiment was repeated three times in triplicate. Statistical analysis was performed using one-way analysis of variance (ANOVA) with Dunnett' post hoc test.

6.2 Enzyme-linked immunosorbent assay

To evaluate the anti-inflammatory bioactivity of this new type of naphthylindole with reported reference compound, we detected the effect of **indomethacin** (an indole derivative, non-steroidal anti-inflammatory drug),

compound **15c**, and compound **47c** on the expression of 1L-1β and TNF-α in BV-2 microglial cells by using a commercial ELISA kit (PEPROTECH, USA). BV-2 microglial cells were pretreated with **indomethacin** (10μM) and compounds **15c** (10μM), **47c** (1μM) for 1 h before being stimulated with LPS (1 μg/mL) for another 24 h. **Indomethacin** and compound **15c** both attenuated LPS-induced 1L-1β (Figure 37, A) and TNF-α (Figure 37, B) secretion in BV-2 microglial cells (LPS 1 μg/mL) compared with the LPS group (only LPS), **P* < 0.05, ***P* < 0.01, versus the LPS group. Compound **47c** also attenuated LPS-induced 1L-1β (Figure S47, A) and TNF-α (Figure S47, B) activation in BV-2 microglial cells (LPS 1 μg/mL).

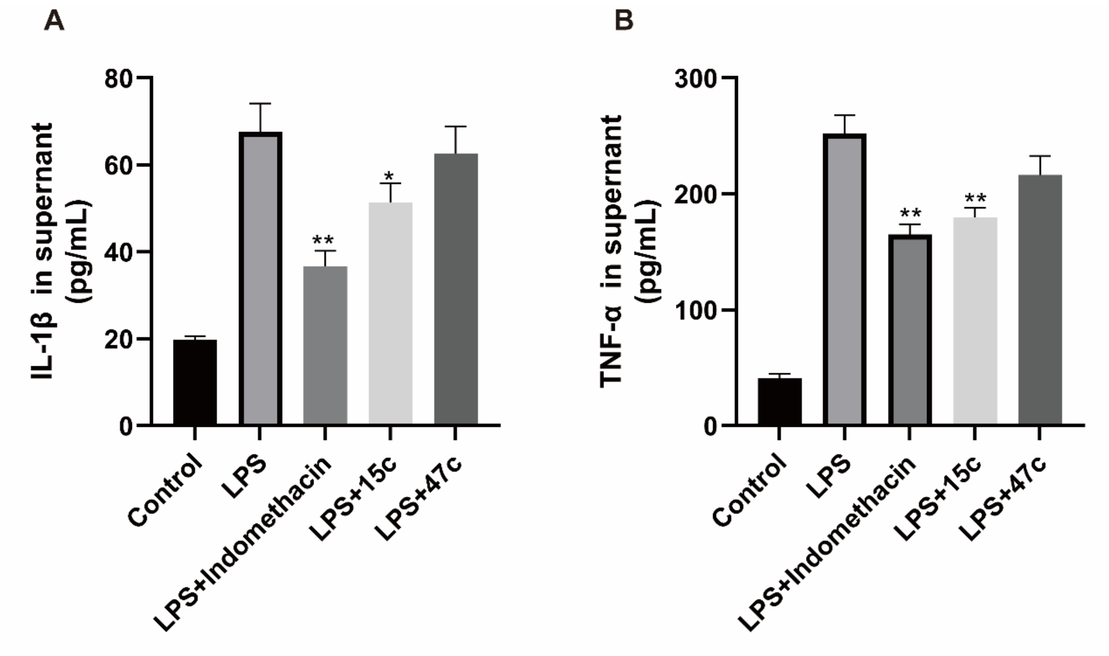

Figure S47 The BV-2 cells were pretreated with positive control indomethacin (non-steroidal anti-inflammatory drug, 10μM) and compounds **15c** (10μM), **47c** (1μM) for 1 h, then incubated with LPS (1 μg/mL) for 24 h. The concentrations of IL-1β (A) and TNF-α (B) in the supernatants were quantified using a commercial ELISA kit (PEPROTECH, USA). (A) IL-1β (B) TNF-α. *P < 0.05, **P < 0.01, versus the LPS group.

6.3 ELISA

Supernatants of BV-2 microglial cells were collected and processed (1500 rpm, 10 min, 4 °C). Il-1β and TNF-α, levels were evaluated by ELISA, according to the manufacturer's instructions (PeproTech,USA). Cytokine levels were measured in a plate reader at 405 nm, with wavelength correction at 650 nm. Cytokine concentrations (pg/mL) were determined using a standard calibration curve.

7. X-ray Crystallographic Data of 15c

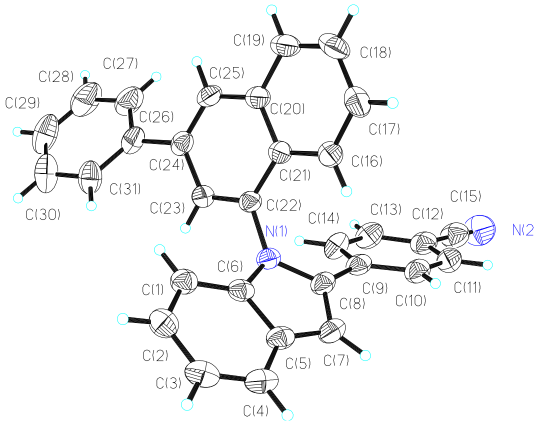

Figure S48. X-ray crystal structure of compound 15c (CCDC number: 2082723)

Table S12. Summary of X-ray crystallographic data for compound 15c.

Empirical formula	$C_{31}H_{20}N_2$
Formula weight	420.49
Temperature/K	293(2)
Crystal system	monoclinic
Space group	P21/c
a/Å	18.4424(11)
b/Å	17.3145(11)
c/Å	7.3180(5)
α/°	90.00
β/°	93.009(2)
γ/°	90.00
Volume/Å^3	2333.6(3)
Z	4
ρ_{calc}g/cm^3	1.197
μ/mm^{-1}	0.070
F(000)	880.0
Crystal size/mm^3	0.30 × 0.28 × 0.25

Radiation	MoKα (λ = 0.71073)
2Θ range for data collection/°	4.7 to 50.02
Index ranges	-21 ≤ h ≤ 21, -20 ≤ k ≤ 20, -8 ≤ l ≤ 8
Reflections collected	22244
Independent reflections	4097 [R(int) = 0.0281]
Data/restraints/parameters	4097/0/298
Goodness-of-fit on F^2	1.085
Final R indexes [I>=2σ (I)]	R_1 = 0.0393, wR_2 = 0.1025
Final R indexes [all data]	R_1 = 0.0422, wR_2 = 0.1048
Largest diff. peak/hole / e $Å^{-3}$	0.15/-0.20

8. Preparation of Substrates

8.1. Synthesis of the *o*-Amino Arylalkynes

A)

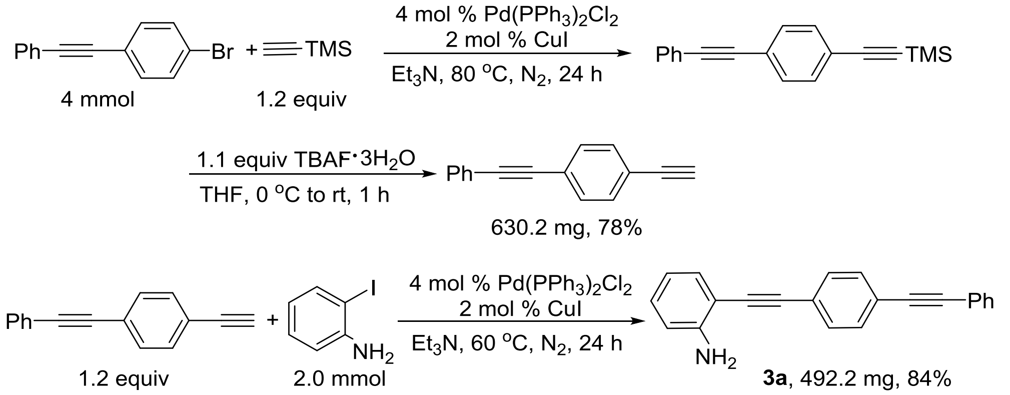

The preparations of this compound was performed according to a literature reference.[12] A Schlenk tube of 100 mL equipped with a magnetic stir bar was charged with $Pd(PPh_3)_2Cl_2$ (112.2 mg, 0.16 mmol, 4 mol %), CuI (15.2 mg, 0.08 mmol, 2 mol %), and 1-bromo-4-(phenylethynyl)benzene (1.028 g, 4 mmol) in triethylamine (16.0 mL); then, ethynyltrimethylsilane (490.0 mg, 5.0 mmol) were added. The reaction mixture was stirred at 80 °C 24 h under N_2. After the starting material was consumed, the mixture was quenched with saturated NH_4Cl solution and extracted with ethyl acetate. The combined organic extracts were washed with water and brine, and dried over anhydrous Na_2SO_4 and concentrated in vacuo. Then the reaction mixture was filtered through a pad of silica gel and washed with diethyl ether. The solvent was evaporated under the reduced pressure to afford trimethyl((4-(phenylethynyl)phenyl)ethynyl)silane as a crude product, which was used directly without further purification for the next step.

To a solution of the above crude product in in THF (16 mL) was added $TBAF{\cdot}3H_2O$ (1.271 g, 4.4 mmol, 1.1 equiv) at 0 °C. The resulting solution was warmed up to room temperature and stirred for 1 h. Then the mixture was quenched with NH_4Cl solution and extracted with dichloromethane. The combined organic extracts were washed with water and brine, and dried over anhydrous Na_2SO_4 and concentrated in vacuo. The crude product was purified by flash column chromatography on silica gel to give the 1-ethynyl-4-(phenylethynyl)benzene (630.2 mg, 78%) as a white solid. ^1H NMR (400 MHz, $CDCl_3$) δ 7.62–7.57 (m, 2H), 7.53 (s, 4H), 7.42–7.36 (m, 3H), 3.23 (s, 1H). ^{13}C NMR (101 MHz, $CDCl_3$) δ 131.9, 131.5, 131.3, 128.4, 128.3, 123.6, 122.8, 121.8, 91.3, 88.8, 83.2, 78.9.

A Schlenk tube of 50 mL equipped with a magnetic stir bar was charged with $Pd(PPh_3)_2Cl_2$ (56.1 mg, 0.08 mmol, 4 mol%), CuI (7.6 mg, 0.04 mmol, 2 mol %), and 1-ethynyl-4-(phenylethynyl)benzene (404.0 mg, 2.4 mmol) in triethylamine (8 mL); then, *o*-iodoaniline (438.0 mg, 2 mmol) were added. The reaction mixture was stirred at

60 °C for 24 h under N_2. After the starting material was consumed, the mixture was quenched with saturated NH_4Cl solution and extracted with ethyl acetate. The combined organic extracts were washed with water and brine, and dried over anhydrous Na_2SO_4 and concentrated in vacuo. The crude product was purified by flash column chromatography on silica gel to give the desired 2-((4-(phenylethynyl)phenyl)ethynyl)aniline (492.2 mg, 84%) as a white solid. ^{1}H NMR (400 MHz, $CDCl_3$) δ 7.59–7.47 (m, 6H), 7.41–7.32 (m, 4H), 7.16 (t, *J* = 7.6 Hz, 1H), 6.83–6.60 (m, 2H), 4.29 (s, 2H). ^{13}C NMR (101 MHz, $CDCl_3$) δ 147.8, 132.1, 131.5, 131.5, 131.3, 129.9, 128.4, 128.3, 123.1, 122.9, 118.0, 114.3, 107.6, 94.4, 91.2, 89.0, 87.8.

B)

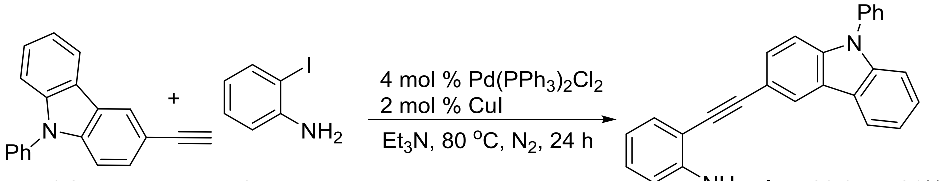

1.2 equiv 2 mmol **4a**, 580.0 mg 81%

A Schlenk tube of 50 mL equipped with a magnetic stir bar was charged with $Pd(PPh_3)_2Cl_2$ (56.1 mg, 0.2 mmol, 4 mol %), CuI (7.6 mg, 0.1 mmol, 2 mol %), and 3-bromo-9-phenyl-9*H*-carbazole (640.8 mg, 2.4 mmol) in triethylamine (8 mL); then, *o*-iodoaniline (438.0 mg, 2 mmol) were added. The reaction mixture was stirred at 60 °C for 24 h under N_2. After the starting material was consumed, the mixture was quenched with saturated NH_4Cl solution and extracted with ethyl acetate. The combined organic extracts were washed with water and brine, and dried over anhydrous Na_2SO_4 and concentrated in vacuo. The crude product was purified by flash column chromatography on silica gel to give the desired 2-((9-phenyl-9H-carbazol-3-yl)ethynyl)aniline (580.0 mg, 81%) as a white solid. ^{1}H NMR (400 MHz, $CDCl_3$) δ 8.35 (s, 1H), 8.15 (d, *J* = 7.7 Hz, 1H), 7.63 (t, *J* = 7.7 Hz, 2H), 7.57 (t, *J* = 7.7 Hz, 3H), 7.52-7.46 (m, 1H), 7.43 (t, *J* = 8.7 Hz, 3H), 7.39 – 7.29 (m, 1H), 7.16 (t, *J* = 7.7 Hz, 1H), 6.78 – 6.73 (m, 2H), 4.34 (s, 2H). ^{13}C NMR (101 MHz, $CDCl_3$) δ 147.6, 141.3, 140.4, 137.2, 132.0, 129.9, 129.4, 129.2, 127.7, 127.0, 126.4, 123.7, 123.3, 122.8, 120.4, 120.4, 117.9, 114.4, 114.2, 109.9, 109.8, 108.5, 95.8, 84.1. HRMS (EI) m/z: [M]+ calcd for $C_{26}H_{18}N_2$ 358.1470; found 358.1472.

General procedure for the synthesis of ortho-alkynylanilines

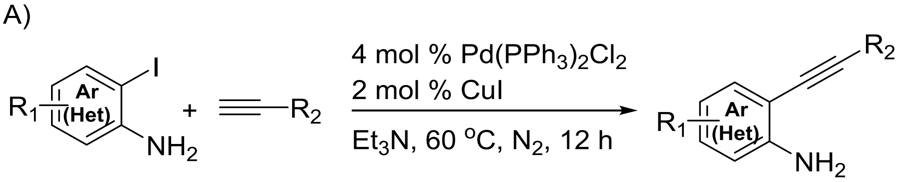

The preparations of 1-(2-bromo-4-(dimethylamino)phenyl)ethanone was performed according to a literature reference.[14] To a 100 mL Schlenk tube charged with 2-Iodoaniline (2 mmol, 1.0 equiv), $Pd(PPh_3)_2Cl_2$ (0.04 mmol, 2 mol %), and CuI (0.08 mmol, 4 mol %) in degassed Et_3N (5 mL) was added aryl alkynes (2.4 mmol, 1.2 equiv), and the resulting solution was stirred at 60 °C for 12 h. Upon completion, the solvent was removed under reduced pressure, and the residue was extracted with EtOAc (3 x 5 mL). The combined organic layer was dried over Na_2SO_4 and concentrated. The residue was purified by a silica gel flash chromatography (petroleum ether / ethyl acetate = 10 / 1) to afford products.

2-((4-iodophenyl)ethynyl)aniline (5a)

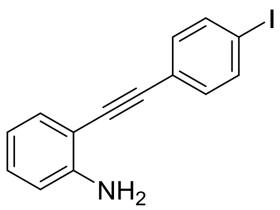

The title compound was prepared according to the general procedure and purified by column chromatography on silica gel and eluted with petroleum ether/ ethyl acetate (20/1) to afford a white powder in 78% yield (497.6 mg). ^{1}H NMR (400 MHz, $CDCl_3$) δ 7.71 (d, J = 7.9 Hz, 2H), 7.39 (d, J = 7.5 Hz, 1H), 7.27 (d, J = 8.0 Hz, 2H), 7.18 (t, J = 7.6 Hz, 1H), 6.80–6.70 (m, 2H), 4.28 (s, 2H). ^{13}C NMR (101 MHz, $CDCl_3$) δ 147.7, 137.4, 132.8, 132.1, 129.9, 122.7, 118.0, 114.3, 107.4, 93.9, 93.7, 87.3. HRMS (EI) m/z: [M]+ calcd for $C_{14}H_{10}I$ 318.9858; found 318.9854.

2-(dec-1-yn-1-yl)aniline (6a)

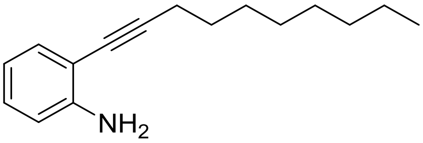

The title compound was prepared according to the general procedure and purified by column chromatography on silica gel and eluted with petroleum ether/ ethyl acetate (20/1) to afford a yellow oil in 88% yield (403.4 mg). ^{1}H NMR (400 MHz, $CDCl_3$) δ 7.34–7.18 (m, 1H), 7.09 (t, J = 7.7 Hz, 1H), 6.68 (t, J = 7.6 Hz, 2H), 4.17 (s, 2H), 2.48 (t, J = 7.0 Hz, 2H), 1.63 (dd, J = 14.4, 7.2 Hz, 2H), 1.53 – 1.43 (m, 2H), 1.40-1.27 (m, 8H), 0.92 (t, J = 5.8 Hz, 3H). ^{13}C NMR (101 MHz, $CDCl_3$) δ 147.5, 131.9, 128.6, 117.7, 114.0, 108.9, 95.7, 76.9, 31.7, 29.1, 29.0, 28.9, 22.6, 19.5, 14.0. HRMS (EI) m/z: [M]+ calcd for $C_{16}H_{23}N$ 229.1830; found 229.1831.

methyl 11-(2-aminophenyl)undec-10-ynoate (7a)

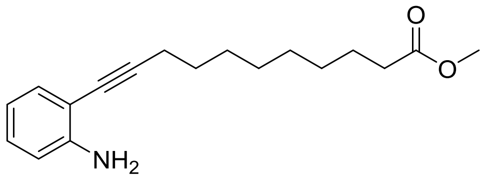

The title compound was prepared according to the general procedure and purified by column chromatography on silica gel and eluted with petroleum ether/ ethyl acetate (20/1) to afford a white powder in 86% yield (493.6 mg). ^{1}H NMR (400 MHz, $CDCl_3$) δ 7.22 (d, *J* = 7.6 Hz, 1H), 7.04 (t, *J* = 7.6 Hz, 1H), 6.67-6.59 (m, 2H), 4.16 (s, 2H), 3.64 (s, 3H), 2.44 (t, *J* = 7.0 Hz, 2H), 2.29 (t, *J* = 7.5 Hz, 2H), 1.66 – 1.52 (m, 4H), 1.44 (s, 2H), 1.31 (s, 6H). ^{13}C NMR (101 MHz, $CDCl_3$) δ 174.0, 147.5, 131.8, 128.6, 117.5, 113.9, 108.7, 95.4, 51.2, 33.8, 28.9, 28.9, 28.7, 28.6, 24.7, 19.4. HRMS (EI) m/z: [M]+ calcd for $C_{18}H_{25}NO_2$ 287.1885; found 287.1882.

2-(3-morpholinoprop-1-yn-1-yl)aniline (8a)

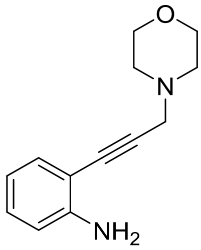

The title compound was prepared according to the general procedure and purified by column chromatography on silica gel and eluted with petroleum ether/ ethyl acetate (20/1) to afford a white powder in 88% yield (380.4 mg). ^{1}H NMR (400 MHz, $CDCl_3$) δ 7.24 (s, 1H), 7.08 (t, *J* = 7.7 Hz, 1H), 6.65 (t, *J* = 9.1 Hz, 2H), 4.16 (s, 2H), 3.74 (d, *J* = 3.7 Hz, 4H), 3.55 (s, 2H), 2.62 (s, 4H). ^{13}C NMR (101 MHz, $CDCl_3$) δ 147.7, 132.1, 129.3, 117.5, 114.0, 107.4, 89.0, 82.0, 66.6, 52.2, 48.0. HRMS (EI) m/z: [M]+ calcd for $C_{13}H_{16}N_2O$ 216.1263; found 216.1268.

4-(phenylethynyl)pyridin-3-amine (9a)

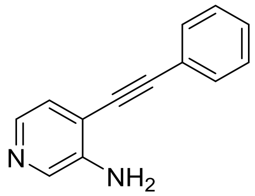

The title compound was prepared according to the general procedure and purified by column chromatography on silica gel and eluted with petroleum ether/ ethyl acetate (20/1) to afford a white powder in 81% yield (314.3 mg). ^{1}H NMR (400 MHz, $CDCl_3$) δ 8.15 (s, 1H), 7.96 (d, *J* = 4.4 Hz, 1H), 7.53 (d, *J* = 3.0 Hz, 2H), 7.37 (s, 3H), 7.18 (d, *J* = 4.3 Hz, 1H), 4.32 (s, 2H). ^{13}C NMR (101 MHz, $CDCl_3$) δ 143.0, 138.8, 136.9, 131.5, 128.9, 128.4, 124.9, 122.1, 114.5, 98.2, 83.2. HRMS (EI) m/z: [M]+ calcd for $C_{13}H_{10}N_2$ 194.0844; found 194.0845.

4-(thiophen-2-ylethynyl)pyridin-3-amine (10a)

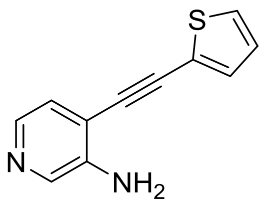

The title compound was prepared according to the general procedure and purified by column chromatography on silica gel and eluted with petroleum ether/ ethyl acetate (20/1) to afford a white powder in 83% yield (332.0 mg). ^{1}H NMR (400 MHz, $CDCl_3$) δ 8.15 (s, 1H), 7.95 (s, 1H), 7.57 (s, 1H), 7.32 (s, 1H), 7.18 (dd, *J* = 11.0, 4.1 Hz, 2H), 4.25 (s, 2H). ^{13}C NMR (101 MHz, $CDCl_3$) δ 143.0, 138.9, 136.9, 129.6, 129.5, 125.7, 124.9, 121.2, 114.6, 93.3, 82.8. HRMS (EI) m/z: [M]+ calcd for $C_{13}H_{10}N_2$ 200.0408; found 200.0405.

8.2. Synthesis of the *o*-Carbonyl Arylalkynes

C)

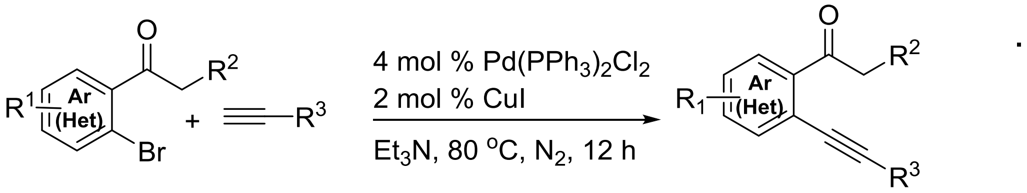

The preparations of 1-(2-bromo-4-(dimethylamino)phenyl)ethanone was performed according to a literature reference.[13] To a 100 mL Schlenk tube charged with 1-(2-bromophenyl)ethanone (398.1 mg, 2.0 mmol), $Pd(PPh_3)_2Cl_2$ (0.04 mmol, 2 mol %), and CuI (0.08 mmol, 4 mol %) in degassed Et_3N (5 mL) was added alkynes (2.4 mmol, 1.2 equiv), and the resulting solution was stirred at 80 °C for 12 h. Upon completion, the solvent was removed under reduced pressure, and the residue was extracted with EtOAc (3 x 5 mL). The combined organic layer was dried over Na_2SO_4 and concentrated. The residue was purified by a silica gel flash chromatography (petroleum ether / ethyl acetate = 20 / 1) to afford products in good yields ranging from 75% to 93%.

1-(4-methoxy-2-(phenylethynyl)phenyl)ethan-1-one (2b)

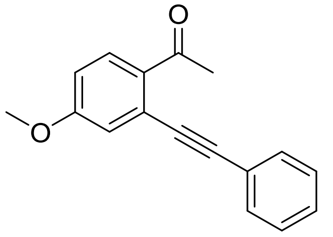

The title compound was prepared according to the general procedure and purified by column chromatography on silica gel and eluted with petroleum ether/ ethyl acetate (20/1) to afford a white powder in 93% yield (465.0 mg). ^{1}H NMR (400 MHz, $CDCl_3$) δ 7.84 (d, *J* = 8.8 Hz, 1H), 7.60–7.54 (m, 3.0 Hz, 2H), 7.39 – 7.35 (m, 3H), 7.11 (d, *J* = 2.6 Hz, 1H), 6.91 (dd, *J* = 8.8, 2.6 Hz, 1H), 3.87 (s, 3H), 2.77 (s, 3H). HRMS (EI) m/z: [M]+ calcd for $C_{17}H_{14}O_2$ 250.0994; found 250.0992.

1- (4-methoxy-5-methyl-2-(phenylethynyl)phenyl)ethan-1-one (3b)

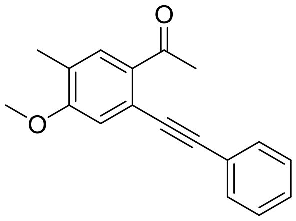

The title compound was prepared according to the general procedure and purified by column chromatography on silica gel and eluted with petroleum ether/ ethyl acetate (20/1) to afford a white powder in 91% yield (480.5 mg). ^{1}H NMR (400 MHz, $CDCl_3$) δ 7.66 (s, 1H), 7.58-7.53 (m, 2H), 7.37–7.32 (m, 3H), 6.99 (s, 1H), 3.87 (s, 3H), 2.77 (s, 3H), 2.23 (s, 3H). ^{13}C NMR (101 MHz, $CDCl_3$) δ 198.2, 159.9, 132.4, 131.7, 131.3, 128.5, 128.3, 127.4, 122.9, 121.4, 114.3, 94.2, 89.3, 55.4, 29.7, 16.0. HRMS (EI) m/z: [M]+ calcd for $C_{18}H_{16}O_2$ 264.1150; found 264.1153.

1-(4-methoxy-5-methyl-2-(phenylethynyl)phenyl)propan-1-one (4b)

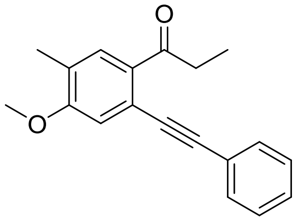

The title compound was prepared according to the general procedure and purified by column chromatography on silica gel and eluted with petroleum ether/ ethyl acetate (20/1) to afford a white powder in 88% yield (489.3 mg). ^{1}H NMR (400 MHz, $CDCl_3$) δ 7.62 (s, 1H), 7.58–7.54 (m, 2H), 7.38–7.34 (m, 3H), 7.00 (s, 1H), 3.88 (s, 3H), 3.18 (q, J = 7.3 Hz, 2H), 2.24 (s, 3H), 1.23 (t, J = 7.3 Hz, 3H). ^{13}C NMR (101 MHz, $CDCl_3$) δ 202.0, 159.7, 132.6, 131.5, 131.4, 128.5, 128.4, 127.6, 123.0, 120.9, 114.4, 93.7, 89.2, 55.5, 34.7, 16.1, 8.7. HRMS (EI) m/z: [M]+ calcd for $C_{19}H_{18}O_2$ 278.1307; found 278.1304.

1-(3-(phenylethynyl)pyridin-2-yl)ethan-1-one (5b)

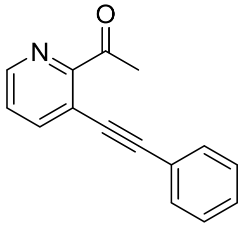

The title compound was prepared according to the general procedure and purified by column chromatography on silica gel and eluted with petroleum ether/ ethyl acetate (20/1) to afford a white powder in 85% yield (375.7 mg). ^{1}H NMR (400 MHz, $CDCl_3$) δ 8.58 (s, 1H), 7.94 (d, J = 7.8 Hz, 1H), 7.61 (s, 2H), 7.46–7.32 (m, 4H), 2.74 (s, 3H). ^{13}C NMR (101 MHz, $CDCl_3$) δ 199.1, 154.3, 147.2, 141.6, 131.9, 128.9, 128.3, 125.3, 122.7, 118.5, 96.4, 86.1, 27.4. HRMS (EI) m/z: [M]+ calcd for $C_{15}H_{11}NO$ 221.0841; found 221.0839.

1-(3-(phenylethynyl)furan-2-yl)ethan-1-one (6b)

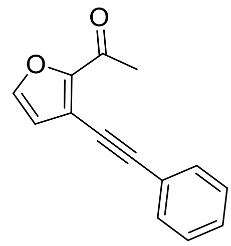

The title compound was prepared according to the general procedure and purified by column chromatography on silica gel and eluted with petroleum ether/ ethyl acetate (20/1) to afford a white powder in 84% yield (353.0 mg). ^{1}H NMR (400 MHz, $CDCl_3$) δ 7.54 (s, 3H), 7.38 (s, 3H), 6.64 (s, 1H), 2.66 (s, 3H). ^{13}C NMR (101 MHz, $CDCl_3$) δ 185.8, 152.6, 145.5, 131.6, 129.1, 128.4, 122.3, 115.5, 114.4, 97.6, 80.8, 27.5. HRMS (EI) m/z: [M]+ calcd for $C_{14}H_{10}O_2$ 210.0681; found 210.0684.

1-(3-(pyridin-3-ylethynyl)thiophen-2-yl)ethan-1-one (7b)

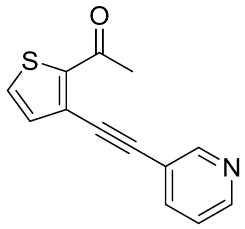

The title compound was prepared according to the general procedure and purified by column chromatography on silica gel and eluted with petroleum ether/ ethyl acetate (20/1) to afford a white powder in 75% yield (340.5 mg). ^{1}H NMR (400 MHz, $CDCl_3$) δ 8.76 (s, 1H), 8.58 (s, 1H), 7.81 (d, J = 7.8 Hz, 1H), 7.57–7.53 (m, 1H), 7.33–7.28 (m, 1H), 7.27–7.22 (m, 1H), 2.78 (s, 3H). ^{13}C NMR (101 MHz, $CDCl_3$) δ 190.4, 151.9, 149.2, 145.9, 138.2, 132.7, 131.8, 124.3, 123.1, 119.6, 92.7, 87.5, 28.8. HRMS (EI) m/z: [M]+ calcd for $C_{13}H_{19}NOS$ 227.0405; found 227.0406.

9. Characterization Data for the Products

1-(3-phenylnaphthalen-1-yl)-2-(p-tolyl)-1H-indole (1c)

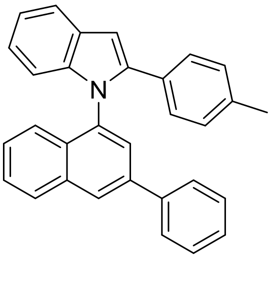

The title compound was prepared according to the general procedure and purified by column chromatography on silica gel and eluted with petroleum ether/dichloromethane (10/1) to afford a white powder in 94% yield (76.9 mg). mp 192–193 °C. ^{1}H NMR (400 MHz, $CDCl_3$) δ 8.19 (s, 1H), 8.04 (d, *J* = 8.2 Hz, 1H), 7.83 (d, *J* = 7.8 Hz, 1H), 7.75–7.71 (m, 1H), 7.68 (d, *J* = 7.5 Hz, 2H), 7.57 (t, *J* = 7.5 Hz, 1H), 7.51 (t, *J* = 7.9 Hz, 3H), 7.42 (t, *J* = 7.4 Hz, 2H), 7.30–7.21 (m, 3H), 7.16 (t, *J* = 7.6 Hz, 1H), 7.01-6.94 (m, 4H), 2.26 (s, 3H). ^{13}C NMR (101 MHz, $CDCl_3$) δ 142.2, 140.1, 139.9, 138.2, 137.1, 135.9, 134.5, 130.4, 129.5, 128.9, 128.8, 128.5, 128.3, 128.0, 127.6, 127.2, 127.0, 126.9, 126.7, 126.0, 123.5, 122.0, 120.5, 120.3, 111.1, 102.8, 21.0. HRMS (EI) m/z: $[M]^+$ calcd for $C_{31}H_{23}N$ 409.1830; found 409.1828.

2-phenyl-1-(3-phenylnaphthalen-1-yl)-1H-indole (2c)

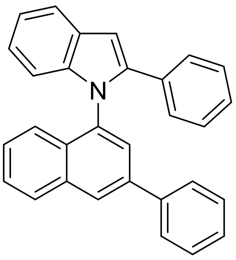

The title compound was prepared according to the general procedure and purified by column chromatography on silica gel and eluted with petroleum ether/dichloromethane (10/1) to afford a white powder in 95% yield (75.0 mg) mp 187–188 °C. ^{1}H NMR (400 MHz, $CDCl_3$) δ 8.13 (s, 1H), 7.99 (d, *J* = 8.2 Hz, 1H), 7.78 (d, *J* = 7.8 Hz, 1H), 7.67–7.65 (m, 1H), 7.62–7.58 (m, 2H), 7.56–7.41 (m, 5H), 7.41–7.34 (m, 2H), 7.32–7.28 (m, 2H), 7.21 (t, *J* = 7.4 Hz, 1H), 7.15–7.11 (m, 3H), 6.98 (s, 1H), 6.93 (d, *J* = 8.2 Hz, 1H). ^{13}C NMR δ (101 MHz, $CDCl_3$) 142.1, 140.2, 139.9, 138.3, 135.8, 134.6, 132.5, 130.4, 128.9, 128.6, 128.3, 128.2, 128.1, 127.7, 127.4, 127.2, 127.0, 126.9 126.8, 126.1, 123.5, 122.3, 120.7, 120.5, 111.2, 103.3. HRMS (EI) m/z: $[M]^+$ calcd for $C_{30}H_{21}N$ 395.1674; found 395.1680.

1-(3-phenylnaphthalen-1-yl)-2-(m-tolyl)-1H-indole (3c)

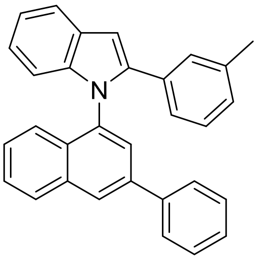

The title compound was prepared according to the general procedure and purified by column chromatography on silica gel and eluted with petroleum ether/dichloromethane (10/1) to afford a white powder in 81% yield (66.3 mg). mp 174–175 °C. ^{1}H NMR (400 MHz, $CDCl_3$) δ 8.13 (s, 1H), 7.99 (d, *J* = 7.8 Hz, 1H), 7.77 (d, *J* = 7.7 Hz, 1H), 7.67 (s, 1H), 7.62 (d, *J* = 7.5 Hz, 2H), 7.52 (t, *J* = 7.3 Hz, 1H),

7.49–7.42 (m, 3H), 7.41–7.34 (m, 2H), 7.24–7.17 (m, 2H), 7.11 (t, *J* = 7.3 Hz, 1H), 7.02–6.89 (m, 5H), 2.18 (s, 3H). ^{13}C NMR (101 MHz, $CDCl_3$) δ 142.3, 140.1, 139.9, 138.2, 137.6, 135.9, 134.5, 132.3, 130.4, 129.0, 128.9, 128.5, 128.2, 128.1, 127.9, 127.6, 127.2, 126.9, 126.9, 126.7, 126.1, 125.1, 123.5, 122.2, 120.6, 120.4, 111.1, 103.1, 21.3. HRMS (EI) m/z: $[M]^+$ calcd for $C_{31}H_{23}N$ 409.1830; found 409.1832.

1-(3-phenylnaphthalen-1-yl)-2-(o-tolyl)-1H-indole (4c)

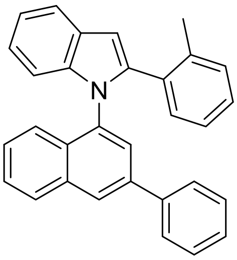

The title compound was prepared according to the general procedure and purified by column chromatography on silica gel and eluted with petroleum ether/dichloromethane (10/1) to afford a white powder in 68% yield (55.6 mg). mp 162–163 °C. ^{1}H NMR (400 MHz, $CDCl_3$) δ 8.04 (s, 1H), 7.95 (d, *J* = 8.2 Hz, 1H), 7.83 (d, *J* = 7.9 Hz, 1H), 7.58–7.50 (m, 5H), 7.50–7.38 (m, 4H), 7.30–7.23 (m, 2H), 7.21–7.09 (m, 3H), 7.03–6.95 (m, 2H), 6.86 (s, 1H), 2.33 (s, 3H). ^{13}C NMR (101 MHz, $CDCl_3$) δ 141.1, 140.0, 138.9, 138.0, 137.4, 135.2, 134.4, 132.2, 131.1, 130.0, 129.9, 128.8, 128.5, 128.1, 128.0, 127.5, 127.1, 126.7, 126.5, 125.9, 124.9, 123.7, 122.0, 120.4, 120.4, 111.3, 104.5, 20.6. HRMS (EI) m/z: $[M]^+$ calcd for $C_{31}H_{23}N$ 409.1830; found 409.1824.

2-(4-pentylphenyl)-1-(3-phenylnaphthalen-1-yl)-1H-indole (5c)

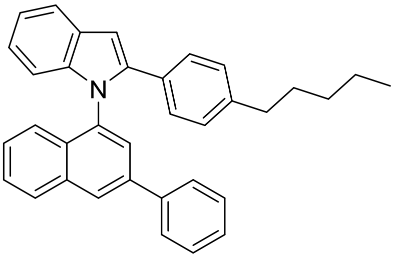

The title compound was prepared according to the general procedure and purified by column chromatography on silica gel and eluted with petroleum ether/dichloromethane (10/1) to afford a white powder in 85% yield (79.1 mg). mp 156–157 °C.^{1}H NMR (400 MHz, $CDCl_3$) δ 8.13 (s, 1H), 8.00 (d, *J* = 8.2 Hz, 1H), 7.77 (d, *J* = 7.8 Hz, 1H), 7.64–7.62 (m, 1H), 7.60–7.57 (m, 2H), 7.56–7.47 (m, 2H), 7.47–7.41 (m, 2H), 7.42–7.34 (m, 2H), 7.23–7.17 (m, 3H), 7.13–7.08 (m, 1H), 6.97–6.88 (m, 4H), 2.58–2.40 (m, 2H), 1.59–1.44 (m, 2H), 1.33–1.14 (m, 4H), 0.84 (t, *J* = 7.0 Hz, 3H). ^{13}C NMR (101 MHz, $CDCl_3$) δ 142.3, 142.1, 140.1, 139.9, 138.3, 135.9, 134.5, 130.4, 129.7, 128.8, 128.5, 128.3, 128.2, 128.0, 127.6, 127.2, 126.9, 126.9, 126.8, 126.0, 123.6, 122.0, 120.5, 120.3, 111.1, 102.8, 35.4, 31.3, 30.8, 22.4, 13.9. HRMS (EI) m/z: $[M]^+$ calcd for $C_{35}H_{31}N$ 465.2457; found 465.2461.

2-(4-(tert-butyl)phenyl)-1-(3-phenylnaphthalen-1-yl)-1H-indole (6c)

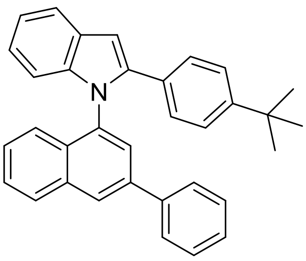

The title compound was prepared according to the general procedure and purified by column chromatography on silica gel and eluted with petroleum ether/dichloromethane (10/1) to afford a white powder in 84% yield (75.8 mg). mp 202–203 °C.^{1}H NMR (400 MHz, $CDCl_3$) ^{1}H NMR (400 MHz, $CDCl_3$) δ 8.12 (s, 1H), 8.00 (d, *J* = 8.2 Hz, 1H), 7.75 (d, *J* = 7.8 Hz, 1H), 7.59–7.57 (m, 1H), 7.57–7.49 (m, 4H), 7.45–7.34 (m, 4H), 7.22–7.12 (m, 5H), 7.08 (t, *J* = 7.4 Hz, 1H), 6.95 (s, 1H), 6.86 (d, *J* = 8.2 Hz, 1H), 1.21 (s, 9H). ^{13}C NMR (101 MHz, $CDCl_3$) δ 150.3, 142.1, 140.1, 140.0, 138.4, 135.9, 134.6, 130.4, 129.5, 128.9, 128.5, 128.3, 127.8, 127.6, 127.2, 127.0, 126.9, 126.9, 126.1, 125.1, 123.7, 122.1, 120.6, 120.3, 111.2, 102.9, 34.4, 31.1. HRMS (EI) m/z: $[M]^+$ calcd for $C_{34}H_{29}N$ 451.2300; found 451.2298.

2-(4-fluorophenyl)-1-(3-phenylnaphthalen-1-yl)-1H-indole (7c)

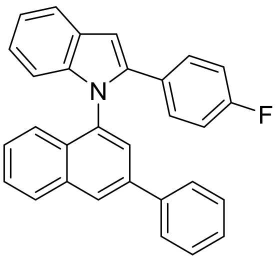

The title compound was prepared according to the general procedure and purified by column chromatography on silica gel and eluted with petroleum ether/dichloromethane (10/1) to afford a white powder in 81% yield (66.9 mg). mp 210–211 °C. ^{1}H NMR (400 MHz, $CDCl_3$) δ 8.13 (s, 1H), 7.99 (d, *J* = 8.2 Hz, 1H), 7.78 (d, *J* = 7.8 Hz, 1H), 7.67–7.65 (m, 1H), 7.62–7.58 (m, 2H), 7.53 (t, *J* = 7.5 Hz, 1H), 7.50–7.42 (m, 3H), 7.41–7.34 (m, 2H), 7.32–7.28 (m, 2H), 7.21 (t, *J* = 7.4 Hz, 1H), 7.15–7.10 (m, 3H), 6.98 (s, 1H), 6.93 (d, *J* = 8.2 Hz, 1H). ^{13}C NMR (101 MHz, $CDCl_3$) δ 162.1 (d, *J* = 247.5 Hz), 141.0, 140.1, 139.8, 138.3, 135.6, 134.6, 130.3, 129.8 (d, *J* = 8.1 Hz), 128.9, 128.6 (d, *J* = 3.4 Hz), 128.6, 128.1, 127.7, 127.2, 127.1, 127.0, 126.7, 126.2, 123.3, 122.4, 120.6 (d, *J* = 28.6 Hz), 115.3, 115.1, 111.2, 103.2. ^{19}F NMR (376 MHz, $CDCl_3$): δ -114.2 (s, 1F). HRMS (EI) m/z: $[M]^+$ calcd for $C_{30}H_{20}FN$ 413.1580; found 413.1577.

2-(4-chlorophenyl)-1-(3-phenylnaphthalen-1-yl)-1H-indole (8c)

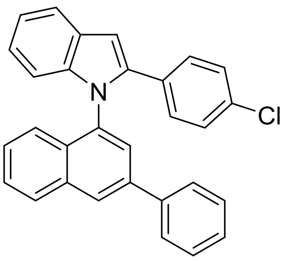

The title compound was prepared according to the general procedure and purified by column chromatography on silica gel and eluted with petroleum ether/dichloromethane (10/1) to afford a white powder in 86% yield (73.8 mg). mp 210–211 °C. ^{1}H NMR (400 MHz, $CDCl_3$) δ 8.15 (s, 1H), 8.00 (d, J = 8.2 Hz, 1H), 7.77 (d, J = 7.8 Hz, 1H), 7.67–7.65 (m, 1H), 7.63 (d, J = 8.1 Hz, 2H), 7.54 (t, J = 7.3 Hz, 1H), 7.46 (t, J = 7.5 Hz, 2H), 7.43–7.34 (m, 3H), 7.24–7.18 (m, 3H), 7.15–7.05 (m, 3H), 6.96 (s, 1H), 6.92

(d, J = 8.2 Hz, 1H). ^{13}C NMR (101 MHz, $CDCl_3$) δ 140.7, 140.3, 139.7, 138.3, 135.5, 134.6, 133.3, 130.9, 130.3, 129.2, 128.9, 128.6, 128.4, 128.1, 127.8, 127.2, 127.1, 127.0, 126.6, 126.3, 123.2, 122.6, 120.8, 120.5, 111.2, 103.5. HRMS (EI) m/z: $[M]^+$ calcd for $C_{30}H_{20}ClN$ 429.1284; found 429.1280.

2-(3-chlorophenyl)-1-(3-phenylnaphthalen-1-yl)-1H-indole (9c)

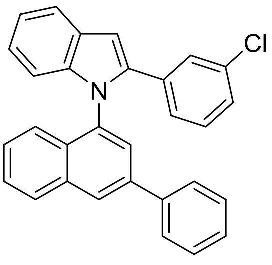

The title compound was prepared according to the general procedure and purified by column chromatography on silica gel and eluted with petroleum ether/dichloromethane (10/1) to afford a white powder in 77% yield (66.1 mg). mp 159–160 °C.^{1}H NMR (400 MHz, $CDCl_3$) δ 8.16 (s, 1H), 8.00 (d, *J* = 8.3 Hz, 1H), 7.78 (d, *J* = 7.8 Hz, 1H), 7.70–7.68(m, 1H), 7.66–7.62 (m, 2H), 7.56–7.51 (m, 1H), 7.50–7.43 (m, 3H), 7.42–7.36 (m, 3H) 7.25–7.20 (m, 1H), 7.17–7.11 (m, 1H), 7.10–7.07 (m, 1H), 7.05–7.02 (m, 1H), 7.00–6.97 (m, 2H), 6.94 (d, *J* = 8.4 Hz, 1H). ^{13}C NMR (101 MHz, $CDCl_3$) δ 140.4, 140.3, 139.8, 138.3, 135.4, 134.6, 134.2, 134.0, 130.2, 129.3, 128.9, 128.6, 128.2, 128.0, 127.7, 127.3, 127.2, 127.2, 127.0, 126.7, 126.4, 125.9, 123.2, 122.7, 120.8, 120.7, 111.2, 103.9. HRMS (EI) m/z: $[M]^+$ calcd for $C_{30}H_{20}ClN$ 429.1284; found 429.1288.

2-(2-chlorophenyl)-1-(3-phenylnaphthalen-1-yl)-1H-indole (10c)

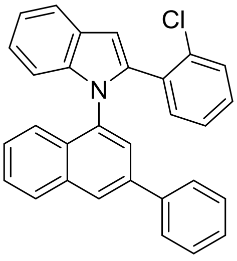

The title compound was prepared according to the general procedure and purified by column chromatography on silica gel and eluted with petroleum ether/dichloromethane (10/1) to afford a white powder in 72% yield (61.8 mg). mp 166–167 °C.^{1}H NMR (400 MHz, $CDCl_3$) δ 8.04 (s, 1H), 7.92 (d, *J* = 8.6 Hz, 1H), 7.81 (d, *J* = 7.8 Hz, 1H), 7.73-7.69 (m, 1H), 7.58 (d, *J* = 7.8 Hz, 2H), 7.53–7.43 (m, 4H), 7.41–7.35 (m, 2H), 7.31 (d, *J* = 8.0 Hz, 1H), 7.27–7.20 (m, 2H), 7.15 (t, *J* = 7.6 Hz, 1H), 7.11–7.04 (m, 1H), 7.01–6.94 (m, 3H). ^{13}C NMR (101 MHz, $CDCl_3$) δ 139.9, 139.0, 138.4, 137.9, 135.0, 134.4, 134.3, 132.3, 131.8, 130.1, 129.6, 129.2, 128.9, 128.4, 127.8, 127.6, 127.1, 126.7, 126.6, 126.5, 126.0, 125.9, 123.7, 122.4, 120.7, 120.6, 111.2, 105.4. HRMS (EI) m/z: $[M]^+$ calcd for $C_{30}H_{20}ClN$ 429.1284; found 429.1281.

1- (4-bromophenyl)-1-(3-phenylnaphthalen-1-yl)-1H-indole (11c)

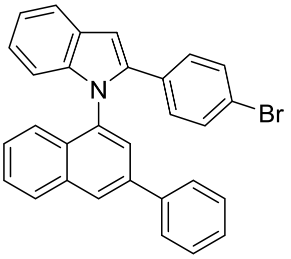

The title compound was prepared according to the general procedure and purified by column chromatography on silica gel and eluted with petroleum ether/dichloromethane (10/1) to afford a white powder in 79% yield (74.7 mg). mp 234–235 °C. ^{1}H NMR (400 MHz, $CDCl_3$) δ 8.16 (s, 1H), 8.00 (d, J = 8.2 Hz, 1H), 7.77 (d, J = 7.7 Hz, 1H), 7.67–7.65 (m, 1H), 7.63 (d, J = 7.8 Hz, 2H), 7.58–7.51 (m, 1H), 7.50–7.44 (m, 2H), 7.43–7.35 (m, 3H), 7.28–7.18 (m, 3H), 7.17–7.08 (m, 3H), 6.96 (s, 1H), 6.92 (d, J = 8.2 Hz, 1H). ^{13}C NMR (101 MHz, $CDCl_3$) δ 140.8, 140.3, 139.7, 138.3, 135.5, 134.6, 131.4, 131.3, 130.3, 129.5, 128.9, 128.6, 128.1, 127.8, 127.2, 127.2, 127.1, 126.6, 126.3, 123.2, 122.5, 121.5, 120.8, 120.6, 111.2, 103.6. HRMS (EI) m/z: $[M]^+$ calcd for $C_{30}H_{20}BrN$ 473.0799; found 473.0795.

2-(4-iodophenyl)-1-(3-phenylnaphthalen-1-yl)-1H-indole (12c)

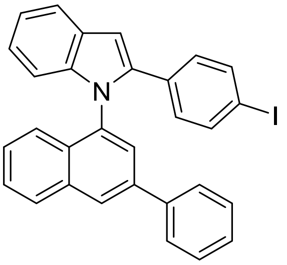

The title compound was prepared according to the general procedure and purified by column chromatography on silica gel and eluted with petroleum ether/dichloromethane (10/1) to afford a white powder in 79% yield (82.3 mg). mp 244–245 °C.^{1}H NMR (400 MHz, $CDCl_3$) δ 8.15 (s, 1H), 7.99 (d, J = 8.3 Hz, 1H), 7.75 (d, J = 7.7 Hz, 1H), 7.67–7.58 (m, 3H), 7.53 (t, J = 6.8 Hz, 1H), 7.49–7.41 (m, 4H), 7.41–7.33 (m, 3H), 7.20 (t, J = 7.3 Hz, 1H), 7.11 (t, J = 7.5 Hz, 1H), 7.01 (d, J = 7.7 Hz, 2H), 6.96 (s, 1H), 6.90 (d, J = 8.0 Hz, 1H). ^{13}C NMR (101 MHz, $CDCl_3$) δ 140.8, 140.3, 139.7, 138.3, 137.3, 135.5, 134.6, 132.0, 130.3, 129.6, 128.9, 128.6, 128.1, 127.8, 127.2, 127.2, 127.1, 126.6, 126.3, 123.2, 122.6, 120.8, 120.6, 111.2, 103.6, 93.2. HRMS (EI) m/z: $[M]^+$ calcd for $C_{30}H_{20}IN$ 521.0640; found 521.0642.

2-(4-methoxyphenyl)-1-(3-phenylnaphthalen-1-yl)-1H-indole (13c)

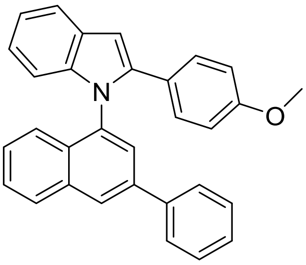

The title compound was prepared according to the general procedure and purified by column chromatography on silica gel and eluted with petroleum ether/dichloromethane (10/1) to afford a white powder in 88% yield (74.8 mg). mp 205–206 °C.^{1}H NMR (400 MHz, $CDCl_3$) δ 8.13 (s, 1H), 7.98 (d, J = 8.2 Hz, 1H), 7.74 (d, J = 7.8 Hz, 1H), 7.67–7.65 (m, 1H), 7.62 (d, J = 7.7 Hz, 2H), 7.52 (t, J = 7.4 Hz, 1H), 7.47–7.41 (m, 3H), 7.39–7.33 (m, 2H), 7.22–7.15 (m, 3H), 7.08 (t, J = 7.7 Hz, 1H), 6.90–6.85 (m, 2H), 6.65 (d, J = 8.7 Hz, 2H), 3.68 (s, 3H). ^{13}C NMR (101 MHz, $CDCl_3$) δ 158.9, 142.0, 140.0, 139.9, 138.3,

135.9, 134.5, 130.4, 129.4, 128.9, 128.5, 128.3, 127.7, 127.2, 127.0, 126.9, 126.7, 126.1, 125.0, 123.5, 121.9, 120.58, 120.2, 113.6, 111.0, 102.3, 55.1. HRMS (EI) m/z: $[M]^+$ calcd for $C_{31}H_{23}NO$ 425.1780; found 425.1782.

2-(4-nitrophenyl)-1-(3-phenylnaphthalen-1-yl)-1H-indole (14c)

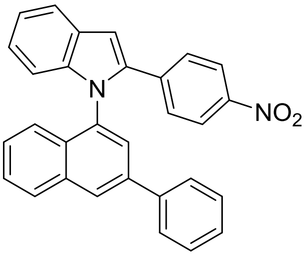

The title compound was prepared according to the general procedure and purified by column chromatography on silica gel and eluted with petroleum ether/dichloromethane (10/1) to afford a yellow powder in 68% yield (59.8 mg). mp 244–245 °C. ^{1}H NMR (400 MHz, $CDCl_3$) δ 8.22 (s, 1H), 8.05 (d, *J* = 8.2 Hz, 1H), 7.99 (d, *J* = 8.6 Hz, 2H), 7.83 (d, *J* = 7.7 Hz, 1H), 7.73–7.70 (m, 1H), 7.66 (d, *J* = 7.4 Hz, 2H), 7.62-7.55 (m, 1H), 7.52–7.38 (m, 7H), 7.32–7.24 (m, 1H), 7.24–7.14 (m, 2H), 6.99 (d, *J* = 8.1 Hz, 1H). ^{13}C NMR (101 MHz, $CDCl_3$) δ 146.4, 140.9, 139.5, 139.2, 138.8, 138.4, 135.2, 134.7, 130.1, 129.0, 128.8, 128.1, 127.9, 127.8, 127.4, 127.3, 127.1, 126.6, 126.5, 123.7, 123.5, 122.9, 121.3, 121.1, 111.4, 105.8. HRMS (EI) m/z: $[M]^+$ calcd for $C_{30}H_{20}N_2O_2$ 440.1525; found 440.1527.

2-(1-(3-phenylnaphthalen-1-yl)-1H-indol-2-yl)benzonitrile (15c)

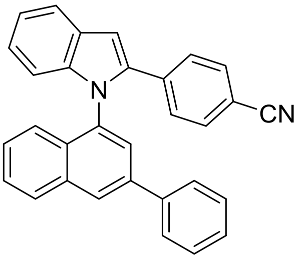

The title compound was prepared according to the general procedure and purified by column chromatography on silica gel and eluted with petroleum ether/dichloromethane (10/1) to afford a pale yellow powder in 76% yield (63.8 mg). mp 222–223 °C. ^{1}H NMR (400 MHz, $CDCl_3$) δ 8.18 (s, 1H), 8.02 (d, *J* = 8.2 Hz, 1H), 7.79 (d, *J* = 7.8 Hz, 1H), 7.66 (s, 1H), 7.62 (d, *J* = 7.9 Hz, 2H), 7.56 (t, *J* = 7.2 Hz, 1H), 7.47 (t, *J* = 7.4 Hz, 2H), 7.44–7.33 (m, 7H), 7.23 (d, *J* = 7.7 Hz, 1H), 7.16 (t, *J* = 7.6 Hz, 1H), 7.08 (s, 1H), 6.95 (d, *J* = 8.2 Hz, 1H). ^{13}C NMR (101 MHz, $CDCl_3$) δ 140.7, 139.6, 139.5, 138.4, 136.8, 135.2, 134.6, 131.9, 130.0, 129.0, 128.7, 128.1, 127.9, 127.3, 127.2, 127.1, 126.5, 126.5, 123.4, 123.0, 121.1, 121.0, 118.6, 111.3, 110.5, 105.3. HRMS (EI) m/z: $[M]^+$ calcd for $C_{31}H_{20}N_2$ 420.1626; found 420.1624.

1-(4-(1-(3-phenylnaphthalen-1-yl)-1H-indol-2-yl)phenyl)ethan-1-one (16c)

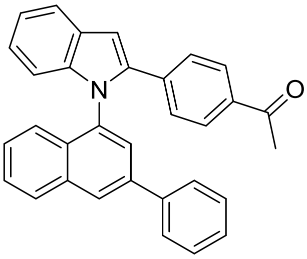

The title compound was prepared according to the general procedure and purified by column chromatography on silica gel and eluted with petroleum ether/dichloromethane (10/1) to afford a pale yellow powder in 75% yield (65.6 mg). mp 189–190 °C. ^{1}H NMR (400 MHz, $CDCl_3$) δ 8.16 (s, 1H), 8.00 (d, *J* = 8.2 Hz, 1H), 7.79 (d, *J* = 7.8 Hz, 1H), 7.72–7.66 (m, 3H), 7.62 (d, *J* = 7.4 Hz, 2H), 7.54 (t, *J* = 7.4 Hz, 1H), 7.45 (t, *J* = 7.7 Hz, 3H), 7.41–7.34 (m, 4H), 7.22 (t, *J* = 7.4 Hz, 1H), 7.14 (t, *J* = 7.6 Hz, 1H), 7.08 (s, 1H), 6.93 (d, *J* = 8.2 Hz, 1H), 2.47 (s, 3H). ^{13}C NMR (101 MHz, $CDCl_3$) δ 197.4, 140.7, 140.6, 139.7, 138.4, 137.0, 135.6, 135.5, 134.6, 130.2, 129.0, 128.7, 128.3, 128.0, 127.8, 127.3, 127.2, 127.1, 126.6, 126.4, 123.2, 123.1, 121.0, 120.8, 111.3, 104.8, 26.4. HRMS (EI) m/z: $[M]^+$ calcd for $C_{32}H_{23}NO$ 437.1780; found 437.1785.

2-(4-(phenylethynyl)phenyl)-1-(3-phenylnaphthalen-1-yl)-1H-indole (17c)

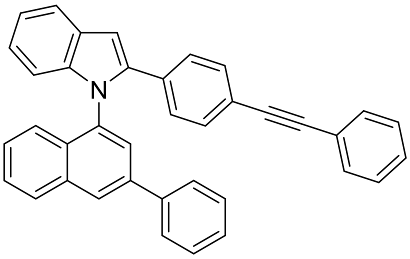

The title compound was prepared according to the general procedure and purified by column chromatography on silica gel and eluted with petroleum ether/dichloromethane (10/1) to afford a white powder in 80% yield (79.2 mg). mp 199–200 °C. ^{1}H NMR (400 MHz, $CDCl_3$) δ 8.13 (s, 1H), 7.97 (d, *J* = 8.2 Hz, 1H), 7.76 (d, *J* = 7.8 Hz, 1H), 7.67 (s, 1H), 7.61 (d, *J* = 7.5 Hz, 2H), 7.50 (t, *J* = 7.4 Hz, 1H), 7.47–7.40 (m, 5H), 7.35 (t, *J* = 7.3 Hz, 2H), 7.32-7.24 (m, 7H), 7.23–7.17 (m, 1H), 7.11 (t, *J* = 7.5 Hz, 1H), 7.00 (s, 1H), 6.92 (d, *J* = 8.2 Hz, 1H). ^{13}C NMR (101 MHz, $CDCl_3$) δ 141.3, 140.4, 139.7, 138.4, 135.7, 134.6, 132.3, 131.5, 131.4, 130.2, 128.9, 128.6, 128.2, 128.2, 127.8, 127.7, 127.2, 127.1, 127.0, 126.6, 126.2, 123.3, 123.1, 122.6, 122.0, 120.8, 120.6, 111.2, 103.8, 90.2, 89.1. HRMS (EI) m/z: $[M]^+$ calcd for $C_{38}H_{25}N$ 495.1987; found 495.1979.

2-octyl-1-(3-phenylnaphthalen-1-yl)-1H-indole (18c)

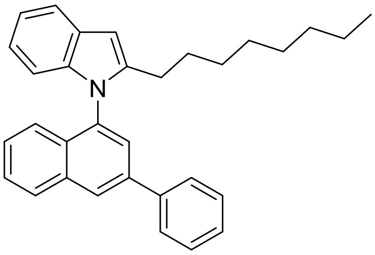

The title compound was prepared according to the general procedure and purified by column chromatography on silica gel and eluted with petroleum ether/dichloromethane (10/1) to afford a sticky pale yellow liquid in 92% yield (79.3 mg). ^{1}H NMR (400 MHz, $CDCl_3$) δ 8.26 (s, 1H), 8.06 (d, *J* = 8.2 Hz, 1H), 7.86 (s, 1H), 7.80 (d, *J* = 7.6 Hz, 2H), 7.72 (d, *J* = 7.8 Hz, 1H), 7.61–7.50 (m, 3H), 7.48–7.35 (m, 2H), 7.19 (t, *J* = 9.5 Hz, 2H), 7.07 (t, *J* = 7.5 Hz, 1H), 6.88 (d, *J* =

8.1 Hz, 1H), 6.60 (s, 1H), 2.55 (t, *J* = 7.5 Hz, 2H), 1.66–1.55 (m, 2H), 1.32–1.02 (m, 10H), 0.87 (t, *J* = 6.9 Hz, 3H). ^{13}C NMR (101 MHz, $CDCl_3$) δ 143.0, 139.8, 139.0, 138.4, 135.1, 134.6, 130.6, 129.0, 128.5, 128.1, 127.7, 127.2, 127.0, 127.0, 126.5, 126.4, 123.2, 121.0, 119.9, 119.6, 110.2, 100.0, 31.7, 29.2, 29.0, 29.0, 28.5, 26.9, 22.5, 14.0. HRMS (EI) m/z: $[M]^+$ calcd for $C_{32}H_{33}N$ 431.2613; found 431.2611.

3- (tert-butyl)-1-(3-phenylnaphthalen-1-yl)-1H-indole (19c)

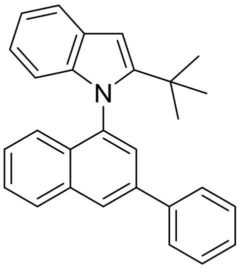

The title compound was prepared according to the general procedure and purified by column chromatography on silica gel and eluted with petroleum ether/dichloromethane (10/1) to afford a sticky pale yellow liquid in 80% yield (60.0 mg). ^{1}H NMR (400 MHz, $CDCl_3$) δ 8.24 (s, 1H), 8.01 (d, *J* = 8.4 Hz, 1H), 7.99–7.95 (m, 1H), 7.78 (d, *J* = 7.9 Hz, 2H), 7.68 (d, *J* = 7.8 Hz, 1H), 7.52 (t, *J* = 7.6 Hz, 3H), 7.46–7.39 (m, 1H), 7.36–7.29 (m, 1H), 7.13 (t, *J* = 7.4 Hz, 1H), 7.03–6.94 (m, 2H), 6.65 (s, 1H), 6.56 (d, *J* = 8.2 Hz, 1H), 1.24 (s, 9H). ^{13}C NMR (101 MHz, $CDCl_3$) δ 151.3, 140.8, 139.8, 138.0, 137.3, 134.4, 131.6, 129.0, 128.3, 128.2, 127.8, 127.2, 126.9, 126.9, 126.6, 123.9, 121.4, 119.9, 119.7, 110.4, 99.7, 33.4, 30.8. HRMS (EI) m/z: $[M]^+$ calcd for $C_{28}H_{25}N$ 375.1987; found 375.1990.

2-cyclopropyl-1-(3-phenylnaphthalen-1-yl)-1H-indole (20c)

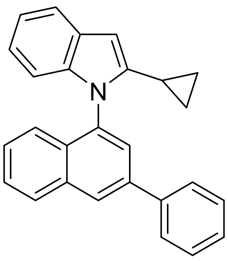

The title compound was prepared according to the general procedure and purified by column chromatography on silica gel and eluted with petroleum ether/dichloromethane (10/1) to afford a sticky pale yellow liquid in 83% yield (59.6 mg). ^{1}H NMR (400 MHz, $CDCl_3$) δ 8.25 (s, 1H), 8.05 (d, *J* = 8.2 Hz, 1H), 7.93–7.90 (m, 1H), 7.88–7.74 (m, 2H), 7.67 (d, *J* = 7.8 Hz, 1H), 7.61–7.48 (m, 3H), 7.48–7.36 (m, 2H), 7.31–7.25 (m, 1H), 7.19–7.12 (m, 1H), 7.11–7.02 (m, 1H), 6.87 (d, *J* = 8.1 Hz, 1H), 6.35 (s, 1H), 1.68–1.47 (m, 1H), 0.8–0.73 (m, 3H), 0.73–0.64 (m, 1H). ^{13}C NMR (101 MHz, $CDCl_3$) δ 145.1, 139.9, 139.2, 138.4, 135.3, 134.6, 130.7, 129.0, 128.4, 127.9, 127.7, 127.2, 126.9, 126.9, 126.6, 126.3, 123.5, 121.1, 120.0, 119.7, 110.1, 96.9, 8.3, 8.1, 7.6. HRMS (EI) m/z: $[M]^+$ calcd for $C_{27}H_{21}N$ 359.1674; found 359.1670.

4-cyclohexyl-1-(3-phenylnaphthalen-1-yl)-1H-indole (21c)

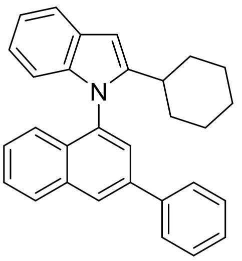

The title compound was prepared according to the general procedure and purified by column chromatography on silica gel and eluted with petroleum ether/dichloromethane (10/1) to afford a sticky pale yellow liquid in 86% yield (69.0 mg). ^{1}H NMR (400 MHz, $CDCl_3$) δ 8.24 (s, 1H), 8.04 (d, J = 8.2 Hz, 1H), 7.87–7.84 (m, 1H), 7.82–7.76 (m, 2H), 7.69 (d, J = 7.8 Hz, 1H), 7.60–7.50 (m, 3H), 7.43 (t, J = 7.4 Hz, 1H), 7.37 (t, J = 7.6 Hz, 1H), 7.20–7.10 (m, 2H), 7.03 (t, J = 7.6 Hz, 1H), 6.79 (d, J = 8.2 Hz, 1H), 6.58 (s, 1H), 2.46–2.36 (m, 1H), 2.07–1.98 (m, 1H), 1.82–1.74 (m, 1H), 1.74–1.67 (m, 1H), 1.66–1.56 (m, 2H), 1.54–1.42 (m, 2H), 1.27–0.90 (m, 3H). ^{13}C NMR (101 MHz, $CDCl_3$) δ 148.6, 139.9, 138.9, 138.3, 135.2, 134.6, 130.8, 129.0, 128.5, 128.1, 127.8, 127.2, 127.1, 127.0, 126.5, 126.4, 123.2, 121.0, 119.9, 119.7, 110.4, 98.2, 36.0, 34.3, 33.1, 26.3, 25.9. HRMS (EI) m/z: [M]$^+$ calcd for $C_{30}H_{27}N$ 401.2143; found 401.2138.

Methyl 9-(1-(3-phenylnaphthalen-1-yl)-1H-indol-2-yl)nonanoate (22c)

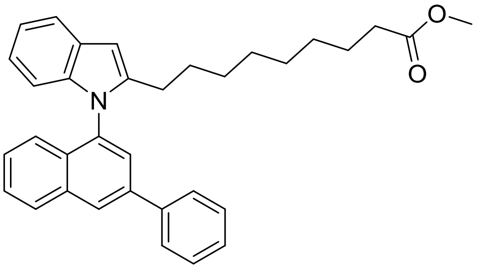

The title compound was prepared according to the general procedure and purified by column chromatography on silica gel and eluted with petroleum ether/dichloromethane (5/1) to afford a sticky pale yellow liquid in 78% yield (76.3 mg). ^{1}H NMR (400 MHz, $CDCl_3$) δ 8.33 (s, 1H), 8.10 (d, J = 8.2 Hz, 1H), 7.94 (s, 1H), 7.86 (d, J = 7.5 Hz, 2H), 7.80 (d, J = 7.7 Hz, 1H), 7.65–7.54 (m, 3H), 7.52–7.40 (m, 2H), 7.32–7.21 (m, 2H), 7.13 (t, J = 7.5 Hz, 1H), 6.95 (d, J = 8.1 Hz, 1H), 6.69 (s, 1H), 3.75 (s, 3H), 2.64 (t, J = 7.3 Hz, 2H), 2.35 (t, J = 7.4 Hz, 2H), 1.72–1.58 (m, 4H), 1.40–1.18 (m, 8H). ^{13}C NMR (101 MHz, $CDCl_3$) δ 173.9, 142.7, 139.7, 139.0, 138.2, 135.1, 134.6, 130.6, 128.9, 128.4, 128.0, 127.6, 127.1, 126.9, 126.9, 126.3, 126.2, 123.1, 121.0, 119.8, 119.6, 110.1, 100.0, 51.2, 33.8, 28.8, 28.8, 28.4, 26.8, 24.7. HRMS (EI) m/z: [M]$^+$ calcd for $C_{34}H_{35}NO_2$ 489.2688; found489.2679.

4-((1-(3-phenylnaphthalen-1-yl)-1H-indol-2-yl)methyl)morpholine (23c)

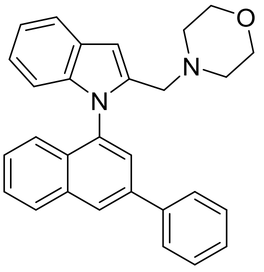

The title compound was prepared according to the general procedure and purified by column chromatography on silica gel and eluted with petroleum ether/dichloromethane (5/1) to afford a sticky pale yellow liquid in 79% yield (66.0 mg). ^{1}H NMR (400 MHz, $CDCl_3$) δ 8.21 (s, 1H), 8.03 (d, J = 8.2 Hz, 1H), 7.90 (s, 1H), 7.78 (d, J = 7.6 Hz, 2H), 7.71 (d, J = 7.8 Hz, 1H), 7.60–7.47 (m, 3H), 7.42 (t, J = 7.3 Hz, 1H), 7.35 (t, J = 7.6 Hz, 1H), 7.23–7.02 (m, 3H), 6.90 (d, J = 8.1 Hz, 1H), 6.70 (s, 1H), 3.53–3.28 (m, 6H), 2.24 (s, 4H). ^{13}C NMR (101 MHz, $CDCl_3$) δ 139.9, 139.4, 138.1, 137.8, 135.1, 134.5, 130.7, 129.0, 128.4, 127.8, 127.4, 127.1, 126.8, 126.8, 126.4, 126.1, 123.4, 121.9, 120.2, 120.1, 110.6, 103.2, 66.7, 54.7, 53.0. HRMS (EI) m/z: $[M]^+$ calcd for $C_{29}H_{26}N_2O$ 418.2045; found 418.2042.

1-(3-phenylnaphthalen-1-yl)-2-(thiophen-3-yl)-1H-indole (24c)

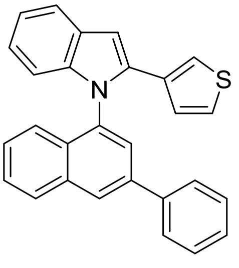

The title compound was prepared according to the general procedure and purified by column chromatography on silica gel and eluted with petroleum ether/dichloromethane (10/1) to afford a white powder in 88% yield (70.6 mg). mp 207-208 °C. ^{1}H NMR (400 MHz, $CDCl_3$) δ 8.27 (s, 1H), 8.06 (d, J = 8.1 Hz, 1H), 7.86–7.76 (m, 2H), 7.73 (d, J = 7.8 Hz, 2H), 7.61–7.46 (m, 3H), 7.45–7.35 (m, 3H), 7.28–7.22(m, 1H), 7.19–7.09 (m, 3H), 7.08–7.03 (m, 1H), 6.97–6.90 (m, 1H), 6.75–6.70 (m, 1H). ^{13}C NMR (101 MHz, $CDCl_3$) δ 140.0, 139.7, 138.4, 137.0, 135.8, 134.6, 132.8, 130.5, 128.9, 128.5, 128.0, 127.7, 127.3, 127.2, 127.1, 126.8, 126.5, 125.0, 123.3, 122.3, 121.4, 120.6, 120.3, 110.8, 102.3. HRMS (EI) m/z: $[M]^+$ calcd for $C_{28}H_{19}NS$ 401.1238; found 401.1233.

1- (3-phenylnaphthalen-1-yl)-2-(pyridin-3-yl)-1H-indole (25c)

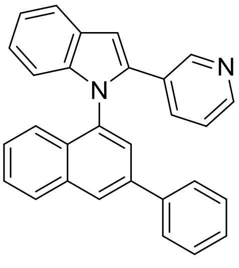

The title compound was prepared according to the general procedure and purified by column chromatography on silica gel and eluted with petroleum ether/dichloromethane (10/1) to afford a pale yellow powder in 88% yield (70.0 mg). mp 165–166 °C. ^{1}H NMR (400 MHz, $CDCl_3$) δ 8.69 (s, 1H), 8.37 (s, 1H), 8.16 (s, 1H), 8.00 (d, J = 8.2 Hz, 1H), 7.81 (d, J = 7.8 Hz, 1H), 7.69 (s, 1H), 7.62 (d, J = 7.4 Hz, 2H), 7.53 (t, J = 7.2 Hz, 1H), 7.49–7.42 (m, 3H), 7.41–7.34 (m, 3H), 7.27–7.20 (m, 1H), 7.16 (t, J = 7.5 Hz, 1H), 7.05 (s, 1H), 7.04–6.98 (m, 1H), 6.96 (d, J = 8.1 Hz, 1H). ^{13}C NMR (101 MHz, $CDCl_3$) δ 148.9, 148.2, 140.4, 139.6, 138.4, 138.3, 135.1, 134.7,

134.6, 130.2, 128.9, 128.6, 128.0, 127.7, 127.3, 127.1, 127.1, 126.7, 126.5, 123.0, 122.9, 120.9, 120.7, 111.2, 104.1. HRMS (EI) m/z: $[M]^+$ calcd for $C_{29}H_{20}N_2$ 396.1626; found 396.1628.

9-phenyl-3-(1-(3-phenylnaphthalen-1-yl)-1H-indol-2-yl)-9H-carbazole (26c)

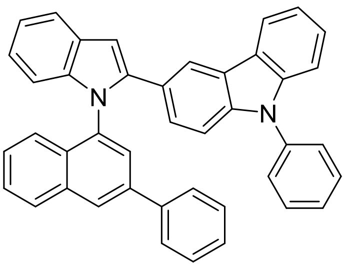

The title compound was prepared according to the general procedure and purified by column chromatography on silica gel and eluted with petroleum ether/dichloromethane (5/1) to afford a white powder in 63% yield (70.6 mg). mp 255-256 °C. ^{1}H NMR (400 MHz, $CDCl_3$) δ 8.16 (s, 1H), 8.11 (s, 1H), 7.96 (d, *J* = 8.1 Hz, 1H), 7.87 (d, *J* = 7.7 Hz, 1H), 7.83–7.73 (m, 2H), 7.61 (d, *J* = 7.6 Hz, 2H), 7.54–7.46 (m, 4H), 7.46–7.36 (m, 5H), 7.36–7.30 (m, 3H), 7.30–7.25 (m, 2H), 7.24–7.18 (m, 2H), 7.14–7.07 (m, 2H), 7.03 (s, 1H), 6.94 (d, *J* = 8.1 Hz, 1H). ^{13}C NMR (101 MHz, $CDCl_3$) δ 143.1, 141.1, 140.2, 140.1, 139.9, 138.3, 137.3, 136.2, 134.5, 130.7, 129.7, 128.8, 128.5, 128.5, 127.6, 127.4, 127.2, 127.0, 126.9, 126.9, 126.3, 126.1, 126.0, 124.5, 123.5, 123.2, 121.9, 120.5, 120.2, 120.1, 120.0, 111.1, 109.8, 109.4, 102.6. HRMS (EI) m/z: $[M]^+$ calcd for $C_{42}H_{28}N_2$ 560.2252; found 560.2255.

5-cyclohexyl-1-(3-phenylnaphthalen-1-yl)-1H-indole (27c)

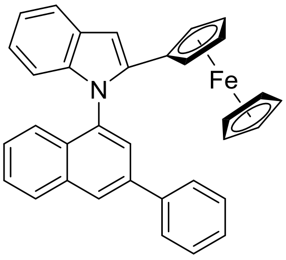

The title compound was prepared according to the general procedure and purified by column chromatography on silica gel and eluted with petroleum ether/dichloromethane (10/1) to afford a red powder in 70% yield (70.4 mg). mp 215–216 °C. ^{1}H NMR (400 MHz, $CDCl_3$) δ 8.29 (s, 1H), 8.06 (d, *J* = 8.2 Hz, 1H), 7.87 (s, 1H), 7.77 (d, *J* = 7.8 Hz, 2H), 7.70 (d, *J* = 7.8 Hz, 1H), 7.57 (t, *J* = 7.5 Hz, 1H), 7.49 (t, *J* = 7.5 Hz, 2H), 7.40 (t, *J* = 7.5 Hz, 2H), 7.31 (d, *J* = 8.4 Hz, 1H), 7.17 (t, *J* = 7.4 Hz, 1H), 7.05 (t, *J* = 7.6 Hz, 1H), 6.95 (s, 1H), 6.81 (d, *J* = 8.1 Hz, 1H), 4.11 (s, 1H), 4.05 (s, 1H), 4.01 (s, 1H), 3.97–3.91 (m, 6H). ^{13}C NMR (101 MHz, $CDCl_3$) δ 140.2, 140.0, 139.7, 138.3, 136.0, 134.5, 130.9, 129.0, 128.5, 128.3, 127.8, 127.2, 127.1, 127.1, 126.9, 126.5, 123.5, 121.4, 120.4, 119.5, 110.4, 100.8, 69.7, 68.4, 68.3, 67.6, 67.1.HRMS (EI) m/z: $[M]^+$ calcd for $C_{34}H_{25}FeN$ 503.1336; found 503.1339.

1-(naphthalen-2-yl)-1-(3-phenylnaphthalen-1-yl)-1H-indole (28c)

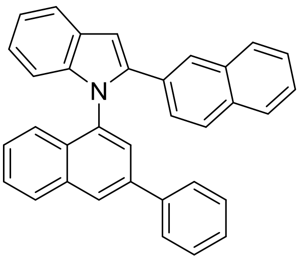

The title compound was prepared according to the general procedure and purified by column chromatography on silica gel and eluted with petroleum ether/dichloromethane (10/1) to afford a white powder in 82% yield (73.0 mg). mp 237–238 °C.^{1}H NMR (400 MHz, $CDCl_3$) δ 8.14 (s, 1H), 8.0–7.95 (m, 1H), 7.84–7.79 (m, 2H), 7.77–7.73 (m, 1H), 7.69–7.64 (s, 1H), 7.61–7.48 (m, 6H), 7.44–7.33 (m, 7H), 7.25–7.20 (m, 1H), 7.17–7.08 (m, 2H), 6.99–6.92 (m, 1H). ^{13}C NMR (101 MHz, $CDCl_3$) δ 142.0, 140.4, 139.8, 138.3, 135.9, 134.6, 133.0, 132.3, 130.4, 129.9, 128.8, 128.5, 128.3, 128.0, 127.6, 127.4, 127.1, 127.1, 127.1, 126.9, 126.7, 126.2, 126.0, 125.9, 125.9, 123.4, 122.4, 120.7, 120.5, 111.2, 103.8. HRMS (EI) m/z: $[M]^+$ calcd for $C_{34}H_{23}N$ 445.1830; found 445.1831.

2-phenyl-1-(3-(p-tolyl)naphthalen-1-yl)-1H-indole (29c)

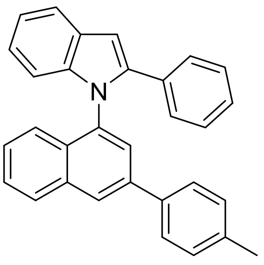

The title compound was prepared according to the general procedure and purified by column chromatography on silica gel and eluted with petroleum ether/dichloromethane (10/1) to afford a white powder in 93% yield (76.1 mg). mp 214–215 °C. ^{1}H NMR (400 MHz, $CDCl_3$) δ 8.12 (s, 1H), 7.99 (d, J = 8.2 Hz, 1H), 7.79 (d, J = 7.8 Hz, 1H), 7.68–7.65 (m, 1H), 7.58–7.44 (m, 5H), 7.40–7.35 (m, 1H), 7.33–7.27 (m, 3H), 7.22 (t, J = 7.4 Hz, 1H), 7.14–7.10 (m, 4H), 6.98 (s, 1H), 6.94 (d, J = 8.2 Hz, 1H), 2.41 (s, 3H). ^{13}C NMR (101 MHz, $CDCl_3$) δ 142.1, 140.1, 138.2, 137.6, 137.0, 135.7, 134.6, 132.5, 130.2, 129.6, 128.4, 128.2, 128.2, 128.1, 127.3, 127.0, 126.8, 126.8, 126.7, 125.7, 123.5, 122.2, 120.6, 120.4, 111.2, 103.2, 21.0. HRMS (EI) m/z: $[M]^+$ calcd for $C_{31}H_{23}N$ 409.1830; found 409.1837.

2-phenyl-1-(3-(m-tolyl)naphthalen-1-yl)-1H-indole (30c)

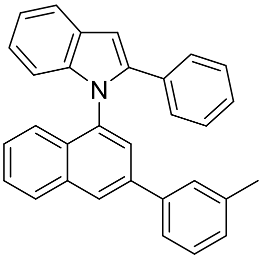

The title compound was prepared according to the general procedure and purified by column chromatography on silica gel and eluted with petroleum ether/dichloromethane (10/1) to afford a white powder in 82% yield (67.1 mg). mp 171–172 °C. ^{1}H NMR (400 MHz, $CDCl_3$) δ 8.18 (s, 1H), 8.03 (d, J = 8.1 Hz, 1H), 7.84 (d, J = 7.8 Hz, 1H), 7.74–7.71 (m, 1H), 7.59–7.52 (m, 2H), 7.49–7.45 (m, 2H), 7.44–7.34 (m, 4H), 7.31–7.23 (m, 2H), 7.21–7.15 (m, 4H), 7.04 (s, 1H), 7.00 (d, J = 8.2 Hz, 1H), 2.47 (s, 3H). ^{13}C NMR (101 MHz, $CDCl_3$) δ

142.1, 140.1, 139.8, 138.5, 138.4, 135.7, 134.5, 132.4, 130.3, 128.8, 128.5, 128.4, 128.2, 128.1, 128.1, 128.0, 127.3, 126.9, 126.8, 126.8, 126.0, 124.2, 123.4, 122.2, 120.6, 120.4, 111.2, 103.2, 21.4. HRMS (EI) m/z: $[M]^+$ calcd for $C_{31}H_{23}N$ 409.1830; found 409.1829.

2-phenyl-1-(3-(o-tolyl)naphthalen-1-yl)-1H-indole (31c)

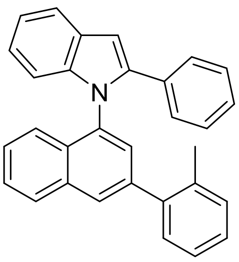

The title compound was prepared according to the general procedure and purified by column chromatography on silica gel and eluted with petroleum ether/dichloromethane (10/1) to afford a white powder in 65% yield (53.2 mg). mp 163–164 °C. ^{1}H NMR (400 MHz, $CDCl_3$) δ 8.13 (s, 1H), 7.99 (d, *J* = 8.2 Hz, 1H), 7.78 (d, *J* = 7.8 Hz, 1H), 7.66 (s, 1H), 7.56–7.44 (m, 2H), 7.45–7.27 (m, 6H), 7.27–7.17 (m, 2H), 7.15–7.09 (m, 4H), 6.98 (s, 1H), 6.93 (d, *J* = 8.2 Hz, 1H), 2.42 (s, 3H). ^{13}C NMR (101 MHz, $CDCl_3$) δ 142.1, 140.2, 139.9, 138.6, 138.4, 135.7, 134.6, 132.5, 130.3, 128.8, 128.5, 128.4, 128.3, 128.2, 128.1, 128.0, 127.3, 126.9, 126.9, 126.9, 126.1, 124.3, 123.5, 122.3, 120.7, 120.5, 111.2, 103.3, 21.5. HRMS (EI) m/z: $[M]^+$ calcd for $C_{31}H_{23}N$ 409.1830; found 409.1831.

1-(3-(4-pentylphenyl)naphthalen-1-yl)-2-phenyl-1H-indole (32c)

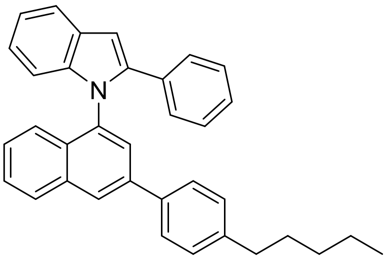

The title compound was prepared according to the general procedure and purified by column chromatography on silica gel and eluted with petroleum ether/dichloromethane (10/1) to afford a white powder in 87% yield (80.9 mg). mp 170–171 °C.^{1}H NMR (400 MHz, $CDCl_3$) δ 8.10 (s, 1H), 7.96 (d, *J* = 8.2 Hz, 1H), 7.76 (d, *J* = 7.8 Hz, 1H), 7.66–7.63 (m, 1H), 7.53–7.42 (m, 4H), 7.37–7.31 (m, 1H), 7.30–7.25 (m, 3H), 7.24–7.22 (m, 1H), 7.19 (t, *J* = 7.6 Hz, 1H), 7.12–7.07 (m, 4H), 6.96 (s, 1H), 6.91 (d, *J* = 8.2 Hz, 1H), 2.70–2.62 (t, *J* = 7.6 Hz, 2H), 1.70–1.60 (m, 2H), 1.38–1.31 (m, 4H), 0.93 (t, *J* = 6.7 Hz, 3H). ^{13}C NMR (101 MHz, $CDCl_3$) δ 142.6, 142.1, 140.2, 138.2, 137.1, 135.7, 134.6, 132.5, 130.2, 129.0, 128.4, 128.2, 128.1, 128.1, 127.3, 127.0, 126.8, 126.8, 126.7, 125.7, 123.4, 122.2, 120.6, 120.4, 111.2, 103.2, 35.5, 31.4, 31.1, 22.5, 14.0. HRMS (EI) m/z: $[M]^+$ calcd for $C_{35}H_{31}N$ 465.2457; found 465.2454.

1-(3-(4-(tert-butyl)phenyl)naphthalen-1-yl)-2-phenyl-1H-indole (33c)

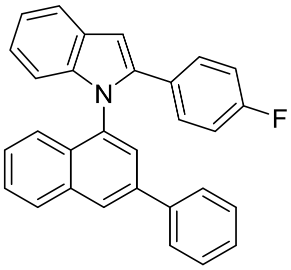

The title compound was prepared according to the general procedure and purified by column chromatography on silica gel and eluted with petroleum ether/dichloromethane (10/1) to afford a white powder in 90% yield (81.2 mg). mp 199–200 °C. ^{1}H NMR (400 MHz, $CDCl_3$) δ 8.15 (s, 1H), 7.99 (d, *J* = 8.2 Hz, 1H), 7.80 (d, *J* = 7.8 Hz, 1H), 7.70–7.68 (m, 1H), 7.58 (d, *J* = 8.3 Hz, 2H), 7.54–7.45 (m, 4H), 7.40–7.35 (m, 1H), 7.33-7.28 (m, 2H), 7.25–7.20 (m, 1H), 7.16–7.11 (m, 4H), 6.99 (s, 1H), 6.94 (d, *J* = 8.2 Hz, 1H), 1.39 (s, 9H). ^{13}C NMR (101 MHz, $CDCl_3$) δ 150.8, 142.1, 140.1, 138.1, 136.9, 135.7, 134.6, 132.4, 130.2, 128.5, 128.2, 128.1, 128.1, 127.3, 126.8, 126.8, 126.6, 125.8, 125.8, 123.5, 122.2, 120.6, 120.4, 111.2, 103.2, 34.5, 31.3. HRMS (EI) m/z: $[M]^+$ calcd for $C_{34}H_{29}N$ 451.2300; found 451.2302.

(3-(4-methoxyphenyl)naphthalen-1-yl)-2-phenyl-1H-indole (34c)

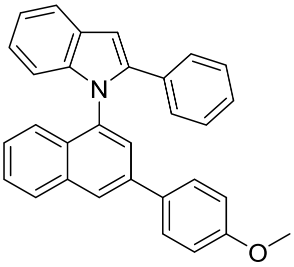

The title compound was prepared according to the general procedure and purified by column chromatography on silica gel and eluted with petroleum ether/dichloromethane (10/1) to afford a white powder in 92% yield (78.2 mg). mp 218–219 °C. ^{1}H NMR (400 MHz, $CDCl_3$) δ 8.06 (s, 1H), 7.96 (d, *J* = 8.2 Hz, 1H), 7.76 (d, *J* = 7.7 Hz, 1H), 7.60 (s, 1H), 7.55–7.48 (m, 3H), 7.45 (d, *J* = 8.4 Hz, 1H), 7.34 (t, *J* = 7.6 Hz, 1H), 7.30–7.25 (m, 2H), 7.20 (t, *J* = 7.4 Hz, 1H), 7.06–7.13 (m, 4H), 7.00–6.94 (m, 3H), 6.91 (d, *J* = 8.2 Hz, 1H), 3.84 (s, 3H). ^{13}C NMR (100 MHz, $CDCl_3$) δ 159.4, 142.1, 140.1, 137.9, 135.7, 134.6, 132.5, 132.3, 130.0, 128.4, 128.2, 128.2, 128.1, 127.3, 126.8, 126.7, 126.5, 125.3, 123.5, 122.2, 120.6, 120.48, 114.3, 111.2, 103.2, 55.3. HRMS (EI) m/z: $[M]^+$ calcd for $C_{31}H_{23}NO$ 425.1780; found 425.1781.

1-(3-(4-fluorophenyl)naphthalen-1-yl)-2-phenyl-1H-indole (35c)

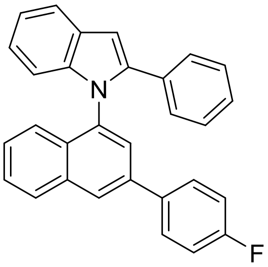

The title compound was prepared according to the general procedure and purified by column chromatography on silica gel and eluted with petroleum ether/dichloromethane (10/1) to afford a white powder in 86% yield (71.0 mg). mp 210–211 °C. ^{1}H NMR (400 MHz, $CDCl_3$) δ 8.07 (s, 1H), 7.98 (d, *J* = 8.2 Hz, 1H), 7.79 (d, *J* = 7.8 Hz, 1H), 7.58–7.49 (m, 5H), 7.43–7.36 (m, 1H), 7.32–7.27 (m, 2H), 7.25–7.19 (m, 1H), 7.16–7.09 (m, 6H), 6.99 (s, 1H), 6.92 (d, *J* = 8.2 Hz, 1H). ^{13}C NMR (101 MHz, $CDCl_3$) δ 162.7

(d, J= 247.3 Hz), 142.1, 140.2, 137.4, 136.10 (d, J= 3.3 Hz), 136.0, 134.6, 132.5, 130.3, 128.9, 128.8, 128.5, 128.3, 128.2, 128.2, 127.4, 127.1, 127.1, 126.6, 126.0, 123.6, 122.4, 120.70 (d, J= 17.5 Hz), 115.87 (d, J= 21.5 Hz), 111.2, 103.4. ^{19}F NMR (376 M, $CDCl_3$): δ -114.7 (s, 1F). HRMS (EI) m/z: $[M]^+$ calcd for $C_{30}H_{20}FN$ 413.1580; found 413.1582.

1-(3-(4-chlorophenyl)naphthalen-1-yl)-2-phenyl-1H-indole (36c)

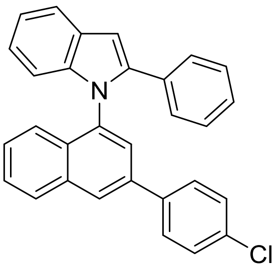

The title compound was prepared according to the general procedure and purified by column chromatography on silica gel and eluted with petroleum ether/dichloromethane (10/1) to afford a white powder in 89% yield (76.4 mg). mp 212–213 °C.^{1}H NMR (400 MHz, $CDCl_3$) δ 8.06 (s, 1H), 7.97 (d, J = 8.2 Hz, 1H), 7.76 (d, J = 7.8 Hz, 1H), 7.56–7.44 (m, 5H), 7.41-7.35 (m, 3H), 7.28–7.23 (m, 3H), 7.19 (t, J = 7.4 Hz, 1H), 7.13–7.09 (m, 4H), 6.96 (s, 1H), 6.89 (d, J = 8.0 Hz, 1H). ^{13}C NMR (101 MHz, $CDCl_3$) δ 142.0, 140.1, 138.3, 137.0, 136.0, 134.5, 133.8, 132.4, 130.4, 129.0, 128.5, 128.4, 128.2, 128.2, 128.6 , 127.3, 127.2, 127.1, 126.4, 126.0, 123.6, 122.3, 120.7, 120.5, 111.1, 103.4. HRMS (EI) m/z: $[M]^+$ calcd for $C_{30}H_{20}ClN$ 429.1284; found 429.1285.

1- (3-(3-chlorophenyl)naphthalen-1-yl)-2-phenyl-1H-indole (37c)

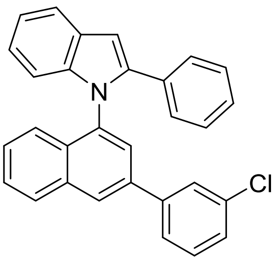

The title compound was prepared according to the general procedure and purified by column chromatography on silica gel and eluted with petroleum ether/dichloromethane (10/1) to afford a white powder in 77% yield (66.1 mg). mp 180–181 °C.^{1}H NMR (400 MHz, $CDCl_3$) δ 8.10 (s, 1H), 8.00 (d, J = 8.1 Hz, 1H), 7.79 (d, J = 7.6 Hz, 1H), 7.60–7.51 (m, 4H), 7.46–7.39 (m, 2H), 7.38–7.33 (m, 2H), 7.31–7.27 (m, 2H), 7.26–7.20 (m, 1H), 7.16–7.10 (m, 4H), 6.99 (s, 1H), 6.92 (d, J = 8.2 Hz, 1H). ^{13}C NMR (101MHz, $CDCl_3$) δ 142.0, 141.7, 140.1, 136.8, 136.0, 134.8, 134.4, 132.4, 130.5, 130.1, 128.6, 128.3, 128.2, 128.1, 127.6, 127.4, 127.3, 127.1, 126.4, 126.3, 125.3, 123.5, 122.3, 120.7, 120.5, 111.1, 103.4, 77.0. HRMS (EI) m/z: $[M]^+$ calcd for $C_{30}H_{20}ClN$ 429.1284; found 429.1290.

2-phenyl-1-(3-(4-(trifluoromethyl)phenyl)naphthalen-1-yl)-1H-indole (38c)

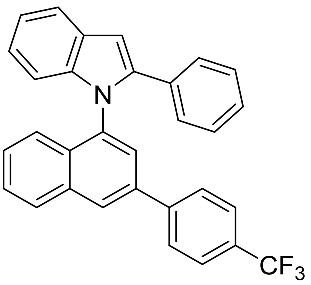

The title compound was prepared according to the general procedure and purified by column chromatography on silica gel and eluted with petroleum ether/dichloromethane (10/1) to afford a white powder in 85% yield (78.7 mg). mp 222-223 °C. ^{1}H NMR (400 MHz, $CDCl_3$) δ 8.13 (s, 1H), 8.01 (d, *J* = 8.2 Hz, 1H), 7.77 (d, *J* = 7.8 Hz, 1H), 7.67 (q, *J* = 8.6 Hz, 4H), 7.60–7.52 (m, 3H), 7.46–7.38 (m, 1H), 7.28–7.24 (m, 2H), 7.21 (t, *J* = 7.4 Hz, 1H), 7.16–7.07 (m, 4H), 6.97 (s, 1H), 6.90 (d, *J* = 8.2 Hz, 1H). ^{13}C NMR (101 MHz, $CDCl_3$) δ 143.4, 142.1, 140.1, 136.8, 136.2, 134.4, 132.4, 130.7, 128.7, 128.3, 128.2, 128.1, 127.5, 127.4, 127.4, 127.2, 126.6, 126.4, 125.8 (q, *J* = 1.7 Hz), 123.6, 122.4, 120.8, 120.6, 111.1, 103.5. ^{19}F NMR (376 MHz, $CDCl_3$) δ -62.46 (s, 1F). HRMS (EI) m/z: $[M]^+$ calcd for $C_{31}H_{20}$ F_3C 463.1548; found 463.1545.

2-phenyl-1-(3-(thiophen-3-yl)naphthalen-1-yl)-1H-indole (39c)

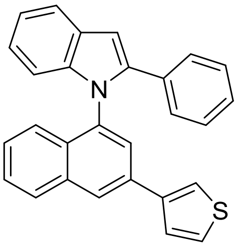

The title compound was prepared according to the general procedure and purified by column chromatography on silica gel and eluted with petroleum ether/dichloromethane (10/1) to afford a white powder in 76% yield (61.0 mg). mp 176–177 °C.^{1}H NMR (400 MHz, $CDCl_3$) δ 8.13 (s, 1H), 7.96 (d, *J* = 8.2 Hz, 1H), 7.80 (d, *J* = 7.9 Hz, 1H), 7.68–7.65 (m, 1H), 7.51 (t, *J* = 7.5 Hz, 1H), 7.48–7.43 (m, 3H), 7.42–7.39 (m, 1H), 7.39–7.34 (m, 1H), 7.33–7.30 (m, 2H), 7.23 (t, *J* = 7.5 Hz, 1H), 7.16–7.10 (m, 4H), 6.99 (s, 1H), 6.92 (d, *J* = 8.2 Hz, 1H). ^{13}C NMR (101MHz, $CDCl_3$) δ 142.0, 141.1, 140.1, 135.8, 134.6, 133.0, 132.4, 130.3, 128.3, 128.2, 128.1, 128.1, 127.3, 127.0, 126.8, 126.6, 126.1, 126.1, 125.1, 123.5, 122.3, 121.0, 120.7, 120.5, 111.2, 103.2. HRMS (EI) m/z: $[M]^+$ calcd for $C_{28}H_{19}NS$ 401.1238; found 401.1233.

1-(3-(tert-butyl)naphthalen-1-yl)-2-phenyl-1H-indole (40c)

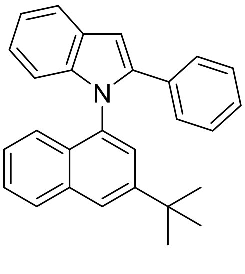

The title compound was prepared according to the general procedure and purified by column chromatography on silica gel and eluted with petroleum ether/dichloromethane (10/1) to afford a sticky pale yellow liquid in 84% yield (63.0 mg). ^{1}H NMR (400 MHz, $CDCl_3$) δ 7.92 (d, *J* = 8.4 Hz, 1H), 7.83 (s, 1H), 7.79 (d, *J* = 7.8 Hz, 1H), 7.53–7.47 (m, 2H), 7.39–7.33 (m, 2H), 7.25–7.19 (m, 3H), 7.15–7.10 (m, 4H), 6.97 (s, 1H), 6.92 (d, *J*= 8.2 Hz, 1H), 1.28 (s, 9H). ^{13}C NMR (101 MHz, $CDCl_3$) δ 148.4, 142.2, 139.8, 134.7, 134.2, 132.5, 129.2, 128.3,

128.2, 128.2, 127.9, 127.1, 126.6, 126.3, 126.2, 123.3, 123.1, 122.1, 120.5, 120.4, 111.4, 103.0, 34.7, 30.8. HRMS (EI) m/z: $[M]^+$ calcd for $C_{28}H_{27}N$ 377.2143; found 377.2145.

1-(3-hexylnaphthalen-1-yl)-2-phenyl-1H-indole (41c)

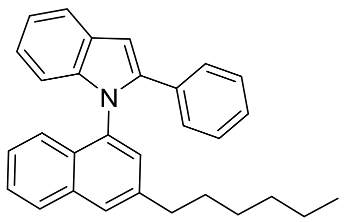

The title compound was prepared according to the general procedure and purified by column chromatography on silica gel and eluted with petroleum ether/dichloromethane (10/1) to afford a white powder in 80% yield (64.5 mg). mp 155–156 °C.^{1}H NMR (400 MHz, $CDCl_3$) δ 7.92 (d, *J* = 8.2 Hz, 1H), 7.82 (d, *J* = 7.8 Hz, 1H), 7.74 (s, 1H), 7.55–7.50 (m, 2H), 7.39–7.35 (m, 1H), 7.31–7.27 (m, 3H), 7.24 (s, 1H), 7.19–7.14 (m, 4H), 7.01 (s, 1H), 6.93 (d, *J* = 8.2 Hz, 1H), 2.77 (t, *J* = 7.4 Hz, 2H), 1.35–1.29 (m, 8H), 0.95 (t, *J* = 6.7 Hz, 3H). ^{13}C NMR (101 MHz, $CDCl_3$) δ 142.1, 140.4, 140.2, 135.1, 134.5, 132.6, 129.7, 128.7, 128.2, 128.0, 127.8, 127.2, 127.0, 126.5, 126.1, 123.5, 122.2, 120.6, 120.5, 111.3, 103.1, 35.7, 31.7, 31.0, 28.6, 22.6, 14.1. HRMS (EI) m/z: $[M]^+$ calcd for $C_{30}H_{29}N$ 403.2300; found 403.2302.

3-(3-cyclohexylnaphthalen-1-yl)-2-phenyl-1H-indole (42c)

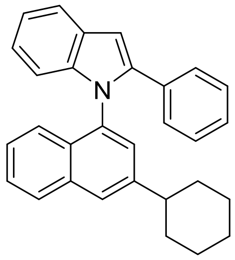

The title compound was prepared according to the general procedure and purified by column chromatography on silica gel and eluted with petroleum ether/dichloromethane (10/1) to afford a sticky pale yellow liquid in 85% yield (68.2 mg). ^{1}H NMR (400 MHz, $CDCl_3$) δ 7.92 (d, *J* = 8.2 Hz, 1H), 7.80 (d, *J* = 7.8 Hz, 1H), 7.74 (s, 1H), 7.53–7.44 (m, 2H), 7.35 (t, *J* = 7.6 Hz, 1H), 7.28–7.20 (m, 4H), 7.18–7.11 (m, 4H), 6.99 (s, 1H), 6.91 (d, *J* = 8.2 Hz, 1H), 2.71–2.59 (m, 1H), 1.97–1.83 (m, 4H), 1.78 (d, *J* = 12.9 Hz, 1H), 1.60(s, 1H), 1.47–1.36 (m, 4H). ^{13}C NMR (101 MHz, $CDCl_3$) δ 145.4, 142.1, 140.0, 134.9, 134.4, 132.5, 129.7, 128.2, 128.1, 127.9, 127.9, 127.5, 127.1, 126.3, 126.0, 125.0, 123.4, 122.1, 120.5, 120.4, 111.3, 102.9, 44.1, 34.2, 33.9, 26.7, 26.7, 26.0. HRMS (EI) m/z: $[M]^+$ calcd for $C_{30}H_{27}N$ 401.2143; found 401.2140.

1-(3-cyclopropylnaphthalen-1-yl)-2-phenyl-1H-indole (43c)

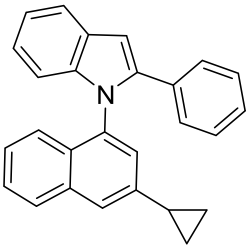

The title compound was prepared according to the general procedure and purified by column chromatography on silica gel and eluted with petroleum ether/dichloromethane (10/1) to afford a sticky pale yellow liquid in 81% yield (58.2 mg). ^{1}H NMR (400 MHz, $CDCl_3$) δ 7.90 (d, *J* = 8.2 Hz, 1H), 7.82 (d, *J* = 7.8 Hz, 1H), 7.69 (s, 1H), 7.54–7.46 (m, 2H), 7.38–7.32 (m, 1H), 7.32–7.23 (m, 3H), 7.21–7.13 (m, 4H), 7.04 (d, *J*= 1.5 Hz, 1H), 7.01 (s, 1H), 6.92 (d, *J* = 8.2 Hz, 1H), 2.15–1.93 (m, 1H), 1.06–1.01 (m, 2H), 0.82–0.75 (m, 1H), 0.63–0.58 (m, 1H). ^{13}C NMR (101 MHz, $CDCl_3$) δ 142.0, 141.5, 140.0, 135.2, 134.3, 132.5, 129.5, 128.1, 128.0, 127.5, 127.2, 126.5, 125.9, 125.5, 124.5, 123.4, 122.1, 120.5, 120.4, 111.2, 103.0, 15.4, 9.6, 9.1. HRMS (EI) m/z: $[M]^+$ calcd for $C_{27}H_{21}N$ 359.1674; found 359.1675.

2-(4-(2-phenyl-1H-indol-1-yl)naphthalen-2-yl)ethan-1-ol (44c)

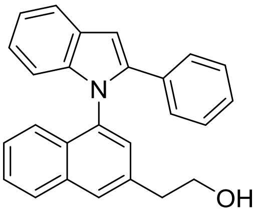

The title compound was prepared according to the general procedure and purified by column chromatography on silica gel and eluted with petroleum ether/dichloromethane (10/1) to afford a sticky pale yellow liquid in 59% yield (42.8 mg). ^{1}H NMR (400 MHz, $CDCl_3$) δ 7.96 (d, *J* = 8.2 Hz, 1H), 7.84 (d, *J* = 7.6 Hz, 1H), 7.80 (s, 1H) , 7.63 (d, *J* = 8.4 Hz, 1H), 7.58 (t, *J* = 7.5 Hz, 1H), 7.48–7.39 (m, 1H), 7.32–7.24 (m, 3H), 7.23–7.12 (m, 5H), 7.03 (s, 1H), 6.94 (d, *J* = 8.2 Hz, 1H), 3.78 (t, *J* = 6.3 Hz, 2H), 3.02–2.90 (m, 2H). ^{13}C NMR (101 MHz, $CDCl_3$) δ 142.0, 139.9, 136.0, 135.3, 134.4, 132.5, 129.8, 128.5, 128.3, 128.2, 128.0, 127.9, 127.8, 127.3, 126.7, 126.5, 123.5, 122.2, 120.6, 120.5, 111.2, 103.3, 63.2, 38.8. HRMS (EI) m/z: $[M]^+$ calcd for $C_{26}H_{21}NO$ 363.1623; found 363.1624.

1-(3-(cyclohex-1-en-1-yl)naphthalen-1-yl)-2-phenyl-1H-indole (45c)

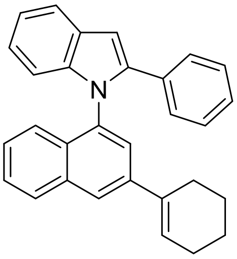

The title compound was prepared according to the general procedure and purified by column chromatography on silica gel and eluted with petroleum ether/dichloromethane (10/1) to afford a white powder in 75% yield (59.9 mg). mp 196–197 °C.^{1}H NMR (400 MHz, $CDCl_3$) δ 7.92–7.85 (m, 2H), 7.78 (d, *J* = 7.8 Hz, 1H), 7.55 (s, 1H), 7.50–7.41 (m, 1H), 7.39 (d, *J* = 8.4 Hz, 1H), 7.36–7.32 (m, 3H), 7.33–7.27 (m, 1H), 7.23–7.19 (m, 1H), 7.15–7.11 (m, 3H), 6.98–6.96 (m, 1H), 6.90 (d, *J* = 7.9 Hz, 1H), 6.20 (s, 1H), 2.57–2.40 (m, 2H), 2.23 (s, 2H), 1.86–1.1.78 (m, 2H), 1.74–1.64 (m, 2H). ^{13}C NMR (101 MHz, $CDCl_3$) δ 142.0, 140.2, 139.5, 135.3, 135.0, 134.3, 132.5,

130.1, 128.3, 128.1, 128.1, 128.0, 127.2, 126.5, 126.3, 126.2, 125.0, 123.4, 123.3, 122.1, 120.5, 120.3, 111.2, 103.0, 27.1, 25.9, 22.9, 22.0. HRMS (EI) m/z: $[M]^+$ calcd for $C_{30}H_{25}N$ 399.1987; found 399.1983.

5-methyl-2-phenyl-1-(3-phenylnaphthalen-1-yl)-1H-indole (46c)

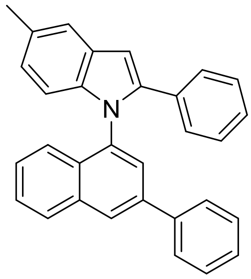

The title compound was prepared according to the general procedure and purified by column chromatography on silica gel and eluted with petroleum ether/dichloromethane (10/1) to afford a white powder in 90% yield (73.6 mg). mp 189–190 °C. ^1H NMR (400 MHz, $CDCl_3$) δ 8.14 (s, 1H), 8.00 (d, *J* = 8.2 Hz, 1H), 7.69–7.59 (m, 4H), 7.56–7.43 (m, 4H), 7.41–7.34 (m, 2H), 7.31–7.23 (m, 2H), 7.14–7.07 (m, 3H), 7.05 (d, *J* = 8.0 Hz, 1H), 6.93 (s, 1H), 6.71 (s, 1H), 2.34 (s, 3H). ^{13}C NMR (101 MHz, CDCl3) δ 141.6, 140.6, 139.9, 138.3, 136.0, 134.5, 132.6, 132.2, 130.4, 128.9, 128.5, 128.0, 128.0, 127.6, 127.2, 127.1, 127.0, 126.9, 126.7, 126.0, 123.6, 122.4, 120.1, 111.0, 103.1, 21.7. HRMS (EI) m/z: $[M]^+$ calcd for $C_{31}H_{23}N$ 409.1830; found 409.1828.

5-fluoro-2-phenyl-1-(3-phenylnaphthalen-1-yl)-1H-indole (47c)

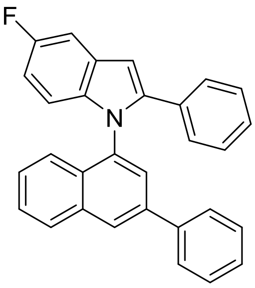

The title compound was prepared according to the general procedure and purified by column chromatography on silica gel and eluted with petroleum ether/dichloromethane (10/1) to afford a white powder in 80% yield (66.1 mg). mp 167–168 °C. ^1H NMR (400 MHz, $CDCl_3$) δ 8.15 (s, 1H), 8.00 (d, *J* = 8.2 Hz, 1H), 7.66 (s, 1H), 7.61 (d, *J* = 7.4 Hz, 2H), 7.55 (t, *J* = 7.4 Hz, 1H), 7.50–7.35 (m, 6H), 7.33–7.28 (m, 2H), 7.17–7.11 (m, 3H), 6.94 (s, 1H), 6.91–6.80 (m, 2H). ^{13}C NMR (101 MHz, $CDCl_3$) δ 158.4 (d, *J* = 235.4 Hz), 143.6, 139.7, 138.3, 136.7, 135.6, 134.6, 132.1, 130.2, 128.9, 128.6, 128.5, 128.4, 128.1 (d, *J* = 1.9 Hz), 127.7, 127.6, 127.2, 127.1, 127.0, 126.7, 126.3, 111.9 (d, *J* = 9.6 Hz), 110.6, 110.4, 105.2 (d, *J* =23.6 Hz), 103.1 (d, *J* = 4.5 Hz). ^{19}F NMR (376 MHz, $CDCl_3$) δ -123.6 (s, 1F). HRMS (EI) m/z: $[M]^+$ calcd for $C_{30}H_{20}FN$ 413.1580; found 413.1585.

1-chloro-2-phenyl-1-(3-phenylnaphthalen-1-yl)-1H-indole (48c)

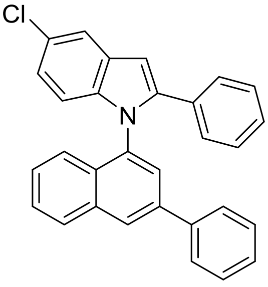

The title compound was prepared according to the general procedure and purified by column chromatography on silica gel and eluted with petroleum ether/dichloromethane (10/1) to afford a white powder in 83% yield (71.2 mg). mp 156–157 °C. ^1H NMR (400 MHz, $CDCl_3$) δ 8.19 (s, 1H), 8.02 (d, *J* = 8.2 Hz, 1H), 7.79(s, 1H), 7.69 (s, 1H), 7.63 (d, *J* = 8.1 Hz, 2H), 7.56 (t, *J* = 7.4 Hz, 1H), 7.53–7.45

(m, 3H), 7.44–7.38 (m, 2H), 7.36–7.31 (m, 2H), 7.19–7.15 (m, 3H), 7.11 (d, *J* = 8.7 Hz, 1H), 6.95 (s, 1H), 6.86 (d, *J* = 8.7 Hz, 1H). ^{13}C NMR (101MHz, $CDCl_3$) δ 143.3, 139.6, 138.5, 138.3, 135.3, 134.5, 131.9, 130.1, 129.2, 128.9, 128.6, 128.1, 127.7, 127.6, 127.1, 127.0, 126.6, 126.3, 126.2, 123.1, 122.5, 119.8, 112.2, 102.6. HRMS (EI) m/z: $[M]^+$ calcd for $C_{30}H_{20}ClN$ 429.1284; found 429.1286.

2-(4-(tert-butyl)phenyl)-5-methyl-1-(3-phenylnaphthalen-1-yl)-1H-indole (49c)

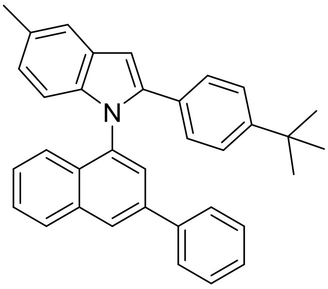

The title compound was prepared according to the general procedure and purified by column chromatography on silica gel and eluted with petroleum ether/dichloromethane (10/1) to afford a white powder in 88% yield (81.8 mg). mp 191-192 °C. ^{1}H NMR (400 MHz, $CDCl_3$) δ 8.13 (s, 1H), 8.01 (d, *J* = 8.2 Hz, 1H), 7.62–7.51 (m, 6H), 7.48–7.31 (m, 4H), 7.21 (d, *J* = 8.5 Hz, 2H), 7.16 (d, *J* = 8.5 Hz, 2H), 6.96–6.86 (m, 2H), 6.78 (d, *J* = 8.3 Hz, 1H), 2.50 (s, 3H), 1.22 (s, 9H). ^{13}C NMR (101 MHz, $CDCl_3$) δ 150.1, 142.1, 140.0, 138.6, 138.3, 136.1, 134.5, 130.4, 129.8, 129.6, 128.8, 128.5, 128.5, 127.7, 127.6, 127.2, 126.9, 126.8, 126.8, 126.0, 125.1, 123.7, 123.6, 120.0, 110.9, 102.5, 34.4, 31.1, 21.4. HRMS (EI) m/z: [M]+ calcd for $C_{35}H_{31}N$ 465.2457; found 465.2459.

2-(4-methoxyphenyl)-5-methyl-1-(3-phenylnaphthalen-1-yl)-1H-indole (50c)

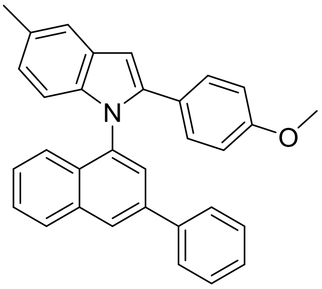

The title compound was prepared according to the general procedure and purified by column chromatography on silica gel and eluted with petroleum ether/dichloromethane (10/1) to afford a white powder in 90% yield (79.0 mg). mp 211–212 °C. ^{1}H NMR (400 MHz, $CDCl_3$) δ 8.14 (s, 1H), 7.99 (d, *J* = 8.2 Hz, 1H), 7.68–7.61 (m, 3H), 7.57–7.50 (m, 2H), 7.50–7.43 (m, 3H), 7.38 (t, *J* = 7.5 Hz, 2H), 7.22 (d, *J* = 8.6 Hz, 2H), 6.94 (d, *J* = 8.3 Hz, 1H), 6.86–6.78 (m, 2H), 6.66 (d, *J* = 8.6 Hz, 2H), 3.68 (s, 3H), 2.50 (s, 3H). ^{13}C NMR (101 MHz, $CDCl_3$) δ 158.8, 142.0, 139.9, 138.5, 138.2, 136.1, 134.5, 130.4, 129.8, 129.3, 128.9, 128.5, 128.5, 127.6, 127.2, 126.9, 126.8, 126.6, 125.9, 125.1, 123.6, 123.5, 119.9, 113.6, 110.7, 101.9, 55.0, 21.4. HRMS (EI) m/z: $[M]^+$ calcd for $C_{32}H_{25}NO$ 439.1936; found 439.1937.

2-(3-chlorophenyl)-5-methyl-1-(3-phenylnaphthalen-1-yl)-1H-indole (51c)

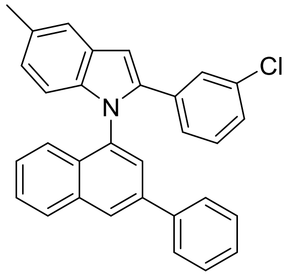

The title compound was prepared according to the general procedure and purified by column chromatography on silica gel and eluted with petroleum ether/dichloromethane (10/1) to afford a white powder in 78% yield (69.1 mg). mp 177–178 °C. ^{1}H NMR (400 MHz, $CDCl_3$) δ 8.15 (s, 1H), 8.00 (d, *J* = 8.2 Hz, 1H), 7.67 (s, 1H), 7.63 (d, *J* = 7.4 Hz, 2H), 7.59–7.51 (m, 2H), 7.47 (t, *J* = 7.6 Hz, 2H), 7.44–7.35 (m, 4H), 7.08 (d, *J* = 7.7 Hz, 1H), 7.04–6.94 (m, 3H), 6.92 (s, 1H), 6.83 (d, *J* = 8.4 Hz, 1H), 2.50 (s, 3H). ^{13}C NMR (101 MHz, $CDCl_3$) δ 140.48, 139.86, 138.87, 138.38, 135.67, 134.61, 134.37, 133.98, 130.32, 130.19, 129.28, 128.95, 128.6, 128.26, 128.1, 127.7, 127.2, 127.1, 127.0, 126.6, 126.3, 125.9, 124.3, 123.3, 120.3, 110.9, 103.5, 21.4. HRMS (EI) m/z: $[M]^+$ calcd for $C_{31}H_{22}ClN$ 443.1441; found 443.1443.

6-methyl-2-phenyl-1-(3-phenylnaphthalen-1-yl)-1H-indole (52c)

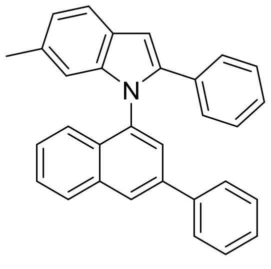

The title compound was prepared according to the general procedure and purified by column chromatography on silica gel and eluted with petroleum ether/dichloromethane (10/1) to afford a white powder in 89% yield (72.8 mg). mp 169–170 °C. ^{1}H NMR (400 MHz, $CDCl_3$) δ 8.15 (s, 1H), 8.00 (d, *J* = 8.2 Hz, 1H), 7.72–7.60 (m, 4H), 7.57–7.42 (m, 4H), 7.42–7.34 (m, 2H), 7.31–7.27 (m, 2H), 7.14–7.09 (m, 3H), 7.05 (d, *J* = 8.0 Hz, 1H), 6.93 (s, 1H), 6.72 (s, 1H), 2.35 (s, 3H). ^{13}C NMR (101 MHz, $CDCl_3$) δ 141.60, 140.66, 139.93, 138.30, 136.03, 134.56, 132.65, 132.26, 130.49, 128.92, 128.52, 128.09, 128.06, 127.68, 127.22, 127.15, 127.01, 126.93, 126.75, 126.07, 123.60, 122.45, 120.15, 111.05, 103.15, 21.78. HRMS (EI) m/z: $[M]^+$ calcd for $C_{31}H_{23}N$ 409.1830; found 409.1825.

6-chloro-2-phenyl-1-(3-phenylnaphthalen-1-yl)-1H-indole (53c)

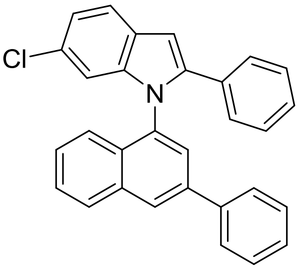

The title compound was prepared according to the general procedure and purified by column chromatography on silica gel and eluted with petroleum ether/dichloromethane (10/1) to afford a white sticky liquid in 81% yield (69.5 mg). mp 176-177 °C. ^{1}H NMR (400 MHz, $CDCl_3$) δ 8.06 (d, *J* = 8.2 Hz, 1H), 7.98 (d, *J* = 7.9 Hz, 2H), 7.82 (s, 1H), 7.74 (d, *J* = 7.5 Hz, 2H), 7.59–7.48 (m, 6H), 7.47–7.35 (m, 5H), 6.87 (s, 2H), 6.79 (d, J = 8.2 Hz, 1H). ^{13}C NMR (101 MHz, $CDCl_3$) δ 147.8, 140.4, 138.8, 136.7,

135.6, 135.0, 133.1, 131.5, 128.9, 128.6, 128.5, 128.4, 127.6, 127.3, 126.8, 126.4, 123.5, 122.6, 122.0, 120.6, 118.6, 112.7, 107.4, 96.4, 84.7. HRMS (EI) m/z: $[M]^+$ calcd for $C_{30}H_{20}ClN$ 429.1284; found 429.1288.

1-(3-cyclopropylnaphthalen-1-yl)-6-methyl-2-phenyl-1H-indole (54c)

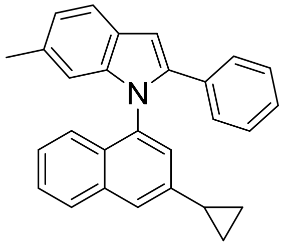

The title compound was prepared according to the general procedure and purified by column chromatography on silica gel and eluted with petroleum ether/dichloromethane (10/1) to afford a sticky pale yellow liquid in 80% yield (59.7 mg). ^{1}H NMR (400 MHz, $CDCl_3$) δ 7.87 (d, *J* = 8.1 Hz, 1H), 7.73–7.63 (m, 2H), 7.54–7.45 (m, 2H), 7.37–7.32 (m, 1H), 7.29–7.24 (m, 2H), 7.18–7.13 (m, 3H), 7.08 (d, *J* = 8.0 Hz, 1H), 7.05–7.03 (m, 1H), 6.95 (s, 1H), 6.71 (s, 1H), 2.38 (s, 3H), 2.07–1.95 (m, 1H), 1.07–0.94 (m, 2H), 0.86–0.71 (m, 1H), 0.68–0.51 (m, 1H). ^{13}C NMR (101 MHz, $CDCl_3$) δ 141.57, 141.49, 140.53, 135.43, 134.32, 132.65, 132.11, 129.64, 128.01, 127.52, 127.02, 126.58, 125.98, 125.93, 125.53, 124.41, 123.52, 122.35, 120.10, 111.08, 102.96, 21.75, 15.42, 9.68, 9.22. HRMS (EI) m/z: $[M]^+$ calcd for $C_{28}H_{23}N$ 373.1830; found 373.1832.

2-cyclopropyl-1-(6-methoxy-7-methyl-3-phenylnaphthalen-1-yl)-1H-indole (55c)

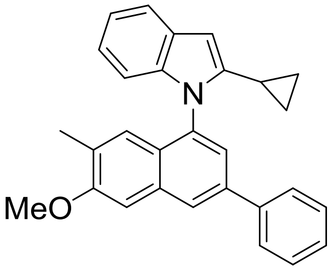

The title compound was prepared according to the general procedure and purified by column chromatography on silica gel and eluted with petroleum ether/dichloromethane (10/1) to afford a sticky pale yellow liquid in 84% yield (67.7 mg). ^{1}H NMR (400 MHz, $CDCl_3$) δ 8.20 (s, 1H), 7.88–7.83 (m, 2H), 7.81–7.79 (m, 1H), 7.74 (d, *J* = 7.8 Hz, 1H), 7.57 (t, *J* = 7.6 Hz, 2H), 7.46 (t, *J* = 7.4 Hz, 1H), 7.35 (s, 1H), 7.26–7.20 (m, 1H), 7.18–7.10 (m, 2H), 7.01 (d, *J* = 8.1 Hz, 1H), 6.42 (s, 1H), 4.06 (s, 3H), 2.33 (s, 3H), 1.71–1.62 (m, 1H), 0.93–0.76 (s, 4H). ^{13}C NMR (101 MHz, $CDCl_3$) δ 157.6, 145.1, 140.2, 139.1, 137.9, 134.8, 134.4, 129.9, 128.8, 127.9, 127.5, 127.1, 125.9 124.8, 121.0, 119.8 119.6, 110.1, 104.9, 96.6, 55.3, 16.9, 8.4, 8.1, 7.7. HRMS (EI) m/z: $[M]^+$ calcd for $C_{29}H_{25}NO$ 403.1936; found 403.1935.

5-(6-fluoro-3-phenylnaphthalen-1-yl)-2-phenyl-1H-indole (56c)

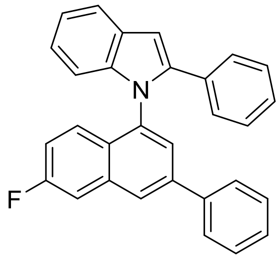

The title compound was prepared according to the general procedure and purified by column chromatography on silica gel and eluted with petroleum ether/dichloromethane (10/1) to afford a pale yellow powder in 91% yield (75.2 mg). mp 225–226 °C. ^{1}H NMR (400 MHz, $CDCl_3$) δ 8.05 (s, 1H), 7.78 (d, *J* = 7.8 Hz, 1H), 7.63 (s, 1H), 7.62–7.57 (m, 3H), 7.45 (t, *J* = 7.4 Hz, 3H), 7.38 (t, *J* = 7.3 Hz, 1H), 7.31–7.27 (m, 2H), 7.22 (t, *J* = 7.4 Hz, 1H), 7.17–7.10 (m, 5H), 6.97 (s, 1H), 6.93 (d, *J* = 8.1 Hz, 1H). ^{13}C NMR (101 MHz, $CDCl_3$) δ 161.2 (d, *J* = 247.9 Hz), 142.0, 140.1, 139.6 (d, *J* = 13.0 Hz), 136.0, 135.5 (d, *J* = 9.3 Hz), 132.3, 128.9, 128.3, 128.1 (d, *J* = 5.6 Hz), 127.9, 127.4, 127.3, 127.2, 126.3, 126.2, 126.0 (d, J=2.0Hz), 125.3 (d, *J* = 5.4 Hz), 122.4, 120.7 (d, *J* = 23.2 Hz), 117.4, 117.2, 111.7, 111.5, 111.0, 103.4. ^{19}F NMR (376 MHz, $CDCl_3$) δ -113.3 (s, 1F). HRMS (EI) m/z: $[M]^+$ calcd for $C_{30}H_{20}FN$ 413.1580; found 413.1581.

1-(7-fluoro-3-phenylnaphthalen-1-yl)-2-phenyl-1H-indole (57c)

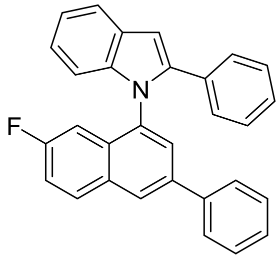

The title compound was prepared according to the general procedure and purified by column chromatography on silica gel and eluted with petroleum ether/dichloromethane (10/1) to afford a pale yellow powder in 87% yield (71.9 mg). mp 228–229 °C. ^{1}H NMR (400 MHz, $CDCl_3$) δ 8.12 (s, 1H), 7.98 (dd, *J* = 9.0, 5.5 Hz, 1H), 7.80 (d, *J* = 7.8 Hz, 1H), 7.73 (s, 1H), 7.60 (d, *J* = 7.4 Hz, 2H), 7.46 (t, *J* = 7.5 Hz, 2H), 7.38 (t, *J* = 7.3 Hz, 1H), 7.34–7.27 (m, 3H), 7.26–7.21 (m, 1H), 7.18–7.13 (m, 4H), 7.09 (dd, *J* = 10.2, 2.2 Hz, 1H), 6.99 (s, 1H), 6.95 (d, *J* = 8.2 Hz, 1H). ^{13}C NMR (101 MHz, $CDCl_3$) δ 161.4 (d, *J* = 248.2 Hz), 141.9, 139.9, 139.6, 137.7 (d, *J* = 2.6 Hz), 135.5 (d, *J* = 5.7 Hz), 132.3, 131.6, 131.4 (d, *J* = 9.0 Hz), 131.1, 131.0, 128.9, 128.3, 128.1, 127.7, 127.4, 127.1, 125.9, 122.5, 120.7 (d, J = 21.9 Hz), 117.6, 117.4, 110.9, 107.3, 107.1, 103.6. ^{19}F NMR (376 MHz, $CDCl_3$) δ -111.4 (s, 1F). HRMS (EI) m/z: $[M]^+$ calcd for $C_{30}H_{20}FN$ 413.1580; found 413.1577.

1-(6-methoxy-3-phenylnaphthalen-1-yl)-2-phenyl-1H-indole (58c)

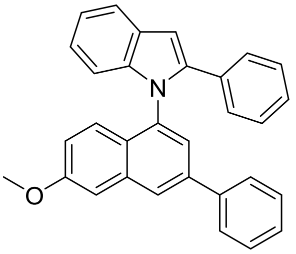

The title compound was prepared according to the general procedure and purified by column chromatography on silica gel and eluted with petroleum ether/dichloromethane (10/1) to afford a pale yellow powder in 91% yield (77.4 mg). mp 180–181 °C. ^{1}H NMR (400 MHz, $CDCl_3$) δ 8.09 (s, 1H), 7.84 (d, *J* = 7.7 Hz, 1H), 7.66 (d, *J* = 7.4 Hz, 2H), 7.59 (s, 1H), 7.52–7.36 (m, 6H), 7.33 (s, 1H),

7.30–7.25 (m, 1H), 7.22–7.14 (m, 4H), 7.09 (d, *J* = 9.1 Hz, 1H), 7.09–6.98 (m, 2H), 3.96 (s, 3H). ^{13}C NMR (101 MHz, $CDCl_3$) δ 158.3, 142.0, 140.1, 140.0, 138.9, 136.0, 135.8, 132.5, 128.8, 128.2, 128.1, 127.6, 127.3, 127.1, 125.8, 125.1, 124.9, 124.3, 122.2, 120.6, 120.4, 119.7, 111.1, 106.4, 103.2, 55.2. HRMS (EI) m/z: $[M]^+$ calcd for $C_{31}H_{23}NO$ 425.1780; found 425.1777.

1-(6-methoxy-7-methyl-3-phenylnaphthalen-1-yl)-2-phenyl-1H-indole (59c)

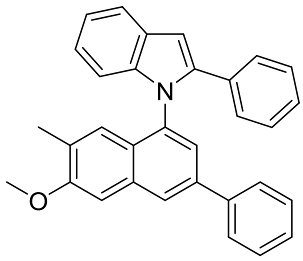

The title compound was prepared according to the general procedure and purified by column chromatography on silica gel and eluted with petroleum ether/dichloromethane (10/1) to afford a pale yellow powder in 85% yield (74.6 mg). mp 188–189 °C. ^{1}H NMR (400 MHz, $CDCl_3$) δ 8.01 (s, 1H), 7.79 (d, *J* = 7.8 Hz, 1H), 7.60 (d, *J* = 7.8 Hz, 2H), 7.48–7.40 (m, 3H), 7.37–7.28 (m, 3H), 7.27–7.19 (m, 3H), 7.16–7.09 (m, 4H), 6.99–6.94 (m, 2H), 3.98 (s, 3H), 2.22 (s, 3H). ^{13}C NMR (101 MHz, $CDCl_3$) δ 157.5, 142.0, 140.2, 140.2, 137.9, 135.0, 134.8, 132.6, 130.0, 128.8, 128.2, 128.1, 127.4, 127.2, 127.1, 125.6, 124.6, 124.2,124.2,122.2, 120.5, 120.4, 111.3, 105.0, 103.0, 55.3, 17.0. HRMS (EI) m/z: $[M]^+$ calcd for $C_{32}H_{25}NO$ 439.1936; found 439.1938.

1-(6-methoxy-2,7-dimethyl-3-phenylnaphthalen-1-yl)-2-(m-tolyl)-1H-indole (60c)

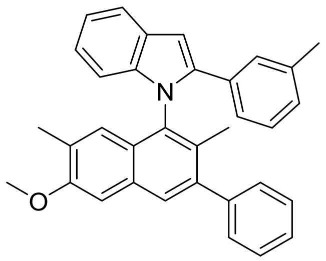

The title compound was prepared according to the general procedure and purified by column chromatography on silica gel and eluted with petroleum ether/dichloromethane(10/1) to afford a white powder in 79% yield (73.8 mg). mp 200–201 °C. ^{1}H NMR (400 MHz, $CDCl_3$) δ 7.80 (d, *J* = 7.8 Hz, 1H), 7.76 (s, 1H), 7.47–7.41 (m, 2H), 7.40–7.32 (m, 3H), 7.26–7.18 (m, 3H), 7.17–7.07 (m, 3H), 6.98 (d, *J* = 8.4 Hz, 3H), 6.82 (d, *J* = 8.1 Hz, 1H), 3.96 (s, 3H), 2.28 (s, 3H), 2.24 (s, 3H), 1.76 (s, 3H). ^{13}C NMR (101 MHz, $CDCl_3$) δ 157.0, 141.9, 141.6, 140.8, 138.8, 137.0, 132.9, 132.2, 131.1, 130.0, 129.8, 129.3, 128.8, 128.3, 128.0, 127.7, 127.4, 126.9, 126.6, 124.2, 122.0, 120.3, 120.2, 111.0, 104.4, 101.8, 55.2, 21.0, 17.0, 15.7. HRMS (EI) m/z: $[M]^+$ calcd for $C_{34}H_{29}NO$ 467.2249; found 467.2245.

2-(4-fluorophenyl)-1-(6-methoxy-2,7-dimethyl-3-phenylnaphthalen-1-yl)-1H-indole (61c)

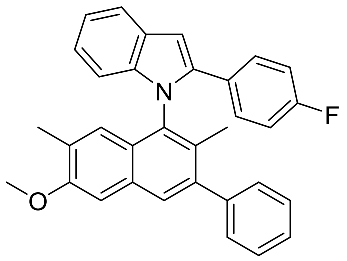

The title compound was prepared according to the general procedure and purified by column chromatography on silica gel and eluted with petroleum ether/dichloromethane(10/1) to afford a light yellow solid in 88% yield (82.9 mg). mp 184-185 °C. ^{1}H NMR (400 MHz, $CDCl_3$) δ 7.76 (d, J = 7.8 Hz, 1H), 7.72 (s, 1H), 7.45–7.38 (m, 2H), 7.37–7.32 (m, 1H), 7.29 (d, J = 7.5 Hz, 2H), 7.23–7.16 (m, 3H), 7.14–7.08 (m, 2H), 7.04 (s, 1H), 6.93 (s, 1H), 6.87–6.75 (m, 3H), 3.95 (s, 3H), 2.20 (s, 3H), 1.67 (s, 3H). ^{13}C NMR (101 MHz, $CDCl_3$) δ 157.1, 141.5, 140.9, 140.8, 138.8, 132.5, 132.3, 131.1, 130.2, 129.4, 129.3, 128.9, 128.2, 128.1, 127.9, 127.0, 126.5, 124.0, 122.3, 120.5, 120.4, 115.3, 115.0, 111.2, 104.6, 102.2, 55.3, 17.15, 15.8. ^{1}H NMR (400 MHz, $CDCl_3$) ^{19}F NMR (376 MHz, $CDCl_3$) δ -57.54 (s). HRMS (EI) m/z: $[M]^+$ calcd for $C_{33}H_{26}FNO$ 471.1988; found 471.1985.

2-(2-chlorophenyl)-1-(6-methoxy-2,7-dimethyl-3-phenylnaphthalen-1-yl)-1H-indole (62c)

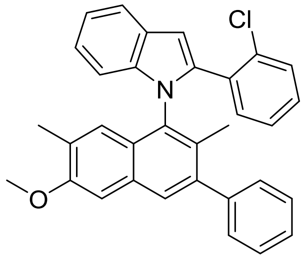

The title compound was prepared according to the general procedure and purified by column chromatography on silica gel and eluted with petroleum ether/dichloromethane(10/1) to afford a white powder in 72% yield (70.2 mg). mp 179–180°C. ^{1}H NMR (400 MHz, $CDCl_3$) δ 7.85 (d, *J* = 7.8 Hz, 1H), 7.67 (s, 1H), 7.48–7.42 (m, 2H), 7.39 (d, *J* = 7.5 Hz, 2H), 7.34 (d, *J* = 7.3 Hz, 2H), 7.28–7.23 (m, 1H), 7.19 (t, *J* = 7.4 Hz, 1H), 7.11 (s, 1H), 7.09–7.03 (m, 3H), 7.01 (d, *J* = 7.6 Hz, 1H), 6.93–6.87 (m, 2H), 3.92 (s, 3H), 2.25 (s, 3H), 1.94 (s, 3H). ^{13}C NMR (101 MHz, $CDCl_3$) δ 156.9, 141.6, 140.6, 137.9, 137.5, 133.6, 132.0, 131.9, 131.2, 131.1, 130.0, 129.8, 129.3, 128.6, 128.0, 127.7, 127.6, 126.9, 126.5, 125.5, 124.2, 122.4, 120.7, 120.4, 111.2, 105.8, 104.3, 55.2, 16.9, 16.3. HRMS (EI) m/z: $[M]^+$ calcd for $C_{33}H_{26}ClNO$ 487.1703; found 487.1705.

1-(6-methoxy-2,7-dimethyl-3-phenylnaphthalen-1-yl)-2-(4-methoxyphenyl)-1H-indole (63c)

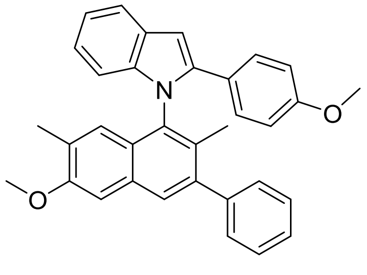

The title compound was prepared according to the general procedure and purified by column chromatography on silica gel and eluted with petroleum ether/dichloromethane(10/1) to afford a white powder in 78% yield (75.4 mg). mp 205-206 °C. δ 7.82–7.70 (m, 2H), 7.46–7.40 (m, 2H), 7.40–7.32 (m, 3H), 7.25–7.17 (t, *J* = 8.3 Hz, 3H), 7.16 (s, 1H), 7.14–7.08 (m, 2H), 6.93 (s, 1H), 6.80 (d, *J* = 8.1 Hz, 1H), 6.71 (d, *J* = 8.1 Hz, 2H), 3.96 (s, 3H), 3.73 (s, 3H), 2.23 (s, 3H), 1.74 (s, 3H).

^{13}C NMR (101 MHz, $CDCl_3$) δ 158.9, 157.0, 141.7, 141.5, 140.8, 138.7, 132.8, 132.2, 131.1, 130.0, 129.3, 128.8, 128.3, 128.0, 127.7, 126.9, 126.6, 125.3, 124.2, 121.8, 120.3, 120.1, 113.6, 111.0, 104.5, 101.4, 55.2, 55.0, 17.0, 15.7. HRMS (EI) m/z: $[M]^+$ calcd for $C_{34}H_{29}NO_2$ 483.2198; found 483.2195.

6-phenyl-8-(2-phenyl-1H-indol-1-yl)quinoline (64c)

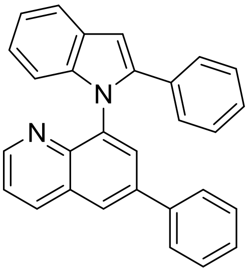

The title compound was prepared according to the general procedure and purified by column chromatography on silica gel and eluted with petroleum ether/ethyl acetate(5/1) to afford a light yellow solid in 76% yield (60.2 mg). mp 170–171 °C ^1H NMR (400 MHz, $CDCl_3$) δ 8.85 (s, 1H), 8.25 (d, *J* = 8.3 Hz, 1H), 8.06 (s, 1H), 7.86 (s, 1H), 7.77 (d, *J* = 7.7 Hz, 1H), 7.57 (d, *J* = 7.4 Hz, 2H), 7.49-7.30 (m, 4H), 7.30–7.35 (m, 2H), 7.20 (t, *J* = 7.3 Hz, 1H), 7.17–7.08 (m, 4H), 6.99 (d, *J* = 7.2 Hz, 2H). ^{13}C NMR (101 MHz, $CDCl_3$) δ 150.8, 144.0, 142.4, 140.1, 139.2, 138.9, 136.8, 136.2, 133.1, 129.7, 129.4, 128.9, 128.5, 128.4, 128.0, 127.9, 127.2, 127.0, 125.4, 122.1, 122.0, 120.7, 120.5, 111.22, 103.70. HRMS (EI) m/z: $[M]^+$ calcd for $C_{29}H_{20}N_2$ 396.1626; found 396.1624.

1-(5-phenylbenzofuran-7-yl)-2-(pyridin-3-yl)-1H-indole (65c)

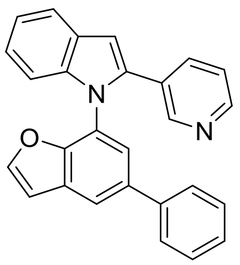

The title compound was prepared according to the general procedure and purified by column chromatography on silica gel and eluted with petroleum ether/ethyl acetate(5/1) to afford a light yellow solid in 78% yield (60.2 mg). mp 175–176 °C. ^1H NMR (400 MHz, $CDCl_3$) δ 8.71 (s, 1H), 8.43 (s, 1H), 7.84 (s, 1H), 7.79–7.73 (m, 1H), 7.56–7.48 (m, 5H), 7.44 (t, *J* = 7.2 Hz, 2H), 7.35 (t, *J* = 7.0 Hz, 1H), 7.28–7.19 (m, 3H), 7.09 (s, 1H), 6.99 (s, 1H), 6.83 (s, 1H). ^{13}C NMR (101 MHz, $CDCl_3$) δ 149.2, 149.0, 148.3, 146.2, 140.35, 139.3, 137.6, 137.4, 134.9, 130.0, 128.8, 128.3, 127.3, 127.3, 123.4, 123.0, 122.8, 122.6, 121.1, 120.9, 119.5, 110.9, 107.2, 104.8. HRMS (EI) m/z: $[M]^+$ calcd for $C_{27}H_{18}N_2O$ 386.1419; found 386.1417.

2-phenyl-1-(5-phenylbenzo[b]thiophen-7-yl)-1H-indole (66c)

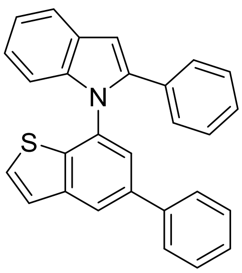

The title compound was prepared according to the general procedure and purified by column chromatography on silica gel and eluted with petroleum ether/dichloromethane (10/1) to afford a white powder in 81% yield (64.96 mg). mp 213–214 °C. ^1H NMR (400 MHz, $CDCl_3$) δ 8.08–8.05 (m, 1H), 7.75 (d, *J*= 7.7 Hz, 1H), 7.56–7.50 (m, 2H), 7.48–7.46(m, 1H), 7.45–7.40 (m, 4H), 7.37–7.33 (m, 3H), 7.23–7.15 (m, 5H), 7.13–7.09

(m, 1H), 6.93 (s, 1H). ^{13}C NMR (101 MHz, $CDCl_3$) δ 141.9, 141.0, 140.2, 138.7, 138.7, 137.6, 133.7, 132.4, 128.8, 128.5, 128.1, 128.0, 128.0, 127.4, 127.2, 124.3, 124.2, 122.3, 121.5, 120.9, 120.6, 111.1, 104.0. HRMS (EI) m/z: $[M]^+$ calcd for $C_{28}H_{19}NS$ 401.1238; found 401.1239.

1-(5-phenylbenzo[b]thiophen-7-yl)-2-(pyridin-3-yl)-1H-indole (67c)

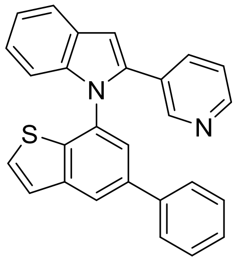

The title compound was prepared according to the general procedure and purified by column chromatography on silica gel and eluted with petroleum ether/dichloromethane (10/1) to afford a white powder in 77% yield (61.9 mg). mp 155–156 °C. ^{1}H NMR (400 MHz, $CDCl_3$) δ 8.71 (s, 1H), 8.41 (s, 1H), 8.10 (s, 1H), 7.79 (d, *J* = 7.6 Hz, 1H), 7.57 (d, *J* = 7.2 Hz, 2H), 7.53 (d, *J* = 6.8 Hz, 2H), 7.48–7.41 (m, 4H), 7.37 (t, *J* = 7.0 Hz, 1H), 7.29–7.18 (m, 2H), 7.14 (d, *J* = 7.9 Hz, 1H), 7.06 (s, 1H), 7.01 (s, 1H). ^{13}C NMR (101 MHz, $CDCl_3$) δ 148.7, 148.4, 142.0, 139.9, 138.9, 138.9, 137.4, 137.1, 134.6, 133.0, 128.8, 128.4, 128.3, 128.1, 127.5, 127.2, 124.4, 124.0, 122.9, 122.9, 121.9, 121.1, 120.8, 111.0, 104.9. HRMS (EI) m/z: $[M]^+$ calcd for $C_{27}H_{18}N_2S$ 402.1191; found 402.1195.

1-(4-(1-(5-phenylbenzo[b]thiophen-7-yl)-1H-indol-2-yl)phenyl)ethan-1-one (68c)

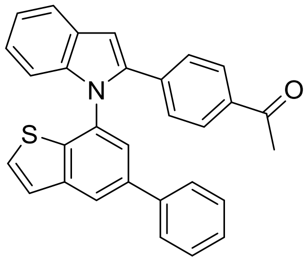

The title compound was prepared according to the general procedure and purified by column chromatography on silica gel and eluted with petroleum ether/dichloromethane (10/1) to afford a white powder in 69% yield (61.1 mg). mp 179–180 °C. ^{1}H NMR (400 MHz, $CDCl_3$) δ 8.10 (s, 1H), 7.75 (d, *J* = 7.1 Hz, 3H), 7.56 (d, *J* = 7.2 Hz, 2H), 7.51 (s, 1H), 7.46–7.39 (m, 6H), 7.39–7.32 (m, 1H), 7.24–7.16 (m, 2H), 7.12 (d, *J* = 7.7 Hz, 1H), 7.03 (s, 1H), 2.96–2.03 (m, 3H). ^{13}C NMR (101 MHz, $CDCl_3$) δ 197.4, 142.0, 140.0, 139.4, 139.3, 138.9, 137.5, 137.0, 135.6, 133.5, 128.9, 128.3, 128.2, 127.6, 127.2, 124.4, 123.9, 123.1, 121.7, 121.2, 121.0, 111.1, 105.6, 26.4. HRMS (EI) m/z: $[M]^+$ calcd for $C_{30}H_{21}NOS$ 443.1344; found 443.1341.

4-(1-(5-phenylbenzo[b]thiophen-7-yl)-1H-indol-2-yl)benzonitrile (69c)

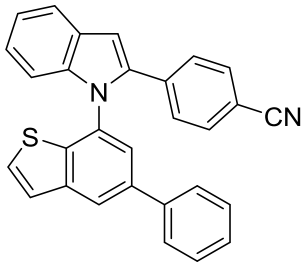

The title compound was prepared according to the general procedure and purified by column chromatography on silica gel and eluted with petroleum ether/dichloromethane (10/1) to afford a white powder in 75% yield (63.9 mg). mp 221–222 °C. ^{1}H NMR (400 MHz, $CDCl_3$) δ 8.13 (s, 1H), 7.78 (d, *J* = 7.2 Hz, 1H), 7.58 (d, *J* = 7.2 Hz, 2H), 7.51 (s, 1H), 7.49–7.35 (m, 9H), 7.26–7.19 (m, 2H),

7.14 (d, *J* = 7.6 Hz, 1H), 7.05 (s, 1H). ^{13}C NMR (101 MHz, CDCl$_3$) δ 142.1, 139.8, 139.3, 139.0, 138.4, 137.3, 136.8, 133.1, 132.0, 128.9, 128.2, 128.1, 127.9, 127.6, 127.2, 124.5, 123.8, 123.5, 121.9, 121.4, 121.1, 118.6, 111.2, 110.6, 106.1. HRMS (EI) m/z: [M]$^+$ calcd for $C_{29}H_{18}N_2S$ 426.1191; found 426.1190.

2-(4-(tert-butyl)phenyl)-5-methyl-1-(5-phenylbenzo[b]thiophen-7-yl)-1H-indole (70c)

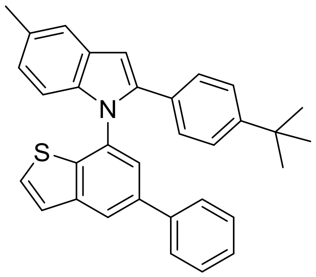

The title compound was prepared according to the general procedure and purified by column chromatography on silica gel and eluted with petroleum ether/dichloromethane (10/1) to afford a white powder in 85% yield (80.1 mg). mp 182–183 °C. ^{1}H NMR (400 MHz, CDCl$_3$) δ 8.11 (s, 1H), 7.62 (s, 1H), 7.54 (d, *J* = 7.2 Hz, 2H), 7.50–7.43 (m, 5H), 7.40 (d, *J* = 6.8 Hz, 1H), 7.34 (d, *J* = 7.8 Hz, 2H), 7.31–7.21 (m, 2H), 7.08 (s, 2H), 6.94 (s, 1H), 2.58 (s, 3H), 1.33 (s, 9H). ^{13}C NMR (101 MHz, CDCl$_3$) δ 150.2, 141.7, 141.1, 140.3, 138.6, 137.5, 136.9, 134.0, 130.0, 129.6, 128.8, 128.7, 128.0, 127.7, 127.3, 127.2, 125.1, 124.3, 124.3, 123.6, 121.3, 120.2, 110.8, 103.2, 34.4, 31.1, 21.4. HRMS (EI) m/z: [M]$^+$ calcd for $C_{33}H_{29}NS$ 471.2021; found 471.2023.

2-(tert-butyl)-1-(5-phenylbenzo[b]thiophen-7-yl)-1H-indole (71c)

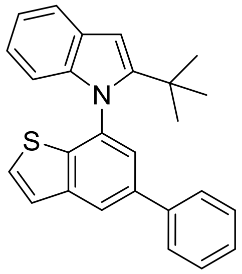

The title compound was prepared according to the general procedure and purified by column chromatography on silica gel and eluted with petroleum ether/dichloromethane (10/1) to afford a sticky pale yellow liquid in 87% yield (66.3 mg). ^{1}H NMR (400 MHz, CDCl$_3$) δ 8.19 (s, 1H), 7.75–7.69 (m, 3H), 7.65 (d, *J* = 7.7 Hz, 1H), 7.53–7.44 (m, 4H), 7.40 (t, *J* = 7.1 Hz, 1H), 7.13 (t, *J* = 7.3 Hz, 1H), 7.03 (t, *J* = 7.6 Hz, 1H), 6.69–6.55 (m, 2H), 1.31 (s, 9H). ^{13}C NMR (101 MHz, CDCl$_3$) δ 150.2, 141.6, 140.1, 139.7, 139.3, 138.5, 135.1, 128.9, 128.0, 127.5, 127.3, 127.2, 126.2, 124.2, 122.1, 121.4, 120.1, 119.8, 110.1, 100.2, 33.3, 30.6. HRMS (EI) m/z: [M]$^+$ calcd for $C_{26}H_{23}NS$ 381.1551; found 381.1550.

2-(tert-butyl)-1-(5-hexylbenzo[b]thiophen-7-yl)-1H-indole (72c)

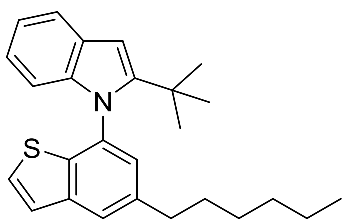

The title compound was prepared according to the general procedure and purified by column chromatography on silica gel and eluted with petroleum ether/dichloromethane (10/1) to afford a yellow liquid in 80% yield (62.3 mg). ^{1}H NMR (400 MHz, CDCl$_3$) δ 7.76 (s, 1H), 7.63 (d, *J* = 7.8 Hz, 1H), 7.40–7.34 (m, 2H), 7.30 (s, 1H), 7.17–7.07 (m, 1H), 7.05–6.97 (m, 1H), 6.62–6.51 (m, 2H), 2.85–2.76 (m, 2H), 1.78–1.67

(m, 2H), 1.39–1.29 (m, 6H), 1.28–1.24 (m, 9H), 0.94–0.87 (m, 3H). ^{13}C NMR (101 MHz, CDCl$_3$) δ 150.2, 141.3, 140.0, 139.2, 138.1, 134.4, 127.7, 127.3, 127.3, 123.7, 123.3, 121.3, 120.0, 119.8, 110.1, 100.0, 35.6, 33.3, 31.6, 31.5, 30.6, 28.8, 22.5, 14.0. HRMS (EI) m/z: [M]$^+$ calcd for $C_{26}H_{31}NS$ 389.2177; found 389.2172.

2-cyclopropyl-1-(5-hexylbenzo[b]thiophen-7-yl)-1H-indole (73c)

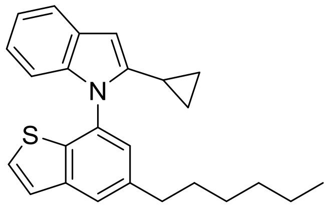

The title compound was prepared according to the general procedure and purified by column chromatography on silica gel and eluted with petroleum ether/dichloromethane (10/1) to afford a yellow liquid in 82% yield (61.2 mg). ^{1}H NMR (400 MHz, CDCl$_3$) δ 7.75 (s, 1H), 7.60 (d, *J* = 7.7 Hz, 1H), 7.40 (dd, *J* = 12.4, 5.4 Hz, 2H), 7.12 (t, *J* = 7.4 Hz, 1H), 7.05 (t, *J* = 7.5 Hz, 1H), 6.89 (d, *J* = 8.0 Hz, 1H), 2.81 (t, *J* = 7.6 Hz, 2H), 1.77–1.67 (m, 2H), 1.67–1.58 (m, 1H), 1.41–1.29 (m, 6H), 0.94–0.85 (m, 3H), 0.85–0.66 (m, 4H). ^{13}C NMR (101 MHz, CDCl$_3$) δ 144.1, 141.5, 140.3, 137.6, 136.6, 132.4, 128.1, 127.2, 125.8, 123.9, 122.9, 120.9, 120.0, 119.8, 110.1, 97.4, 35.6, 31.7, 31.6, 28.8, 22.5, 14.0, 8.5, 8.0, 7.4. HRMS (EI) m/z: [M]$^+$ calcd for $C_{25}H_{27}NS$ 373.1864; found 373.1863.

1-(5-hexylbenzo[b]thiophen-7-yl)-2-phenyl-1H-indole (74c)

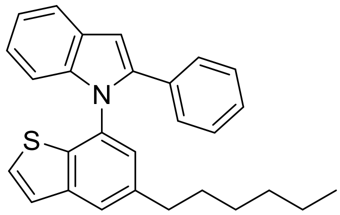

The title compound was prepared according to the general procedure and purified by column chromatography on silica gel and eluted with petroleum ether/dichloromethane (10/1) to afford a sticky pale yellow liquid in 84% yield (68.7 mg). ^{1}H NMR (400 MHz, CDCl$_3$) δ 7.74 (d, *J* = 7.6 Hz, 1H), 7.65 (s, 1H), 7.38 (d, *J* = 5.4 Hz, 1H), 7.33 (d, *J* = 5.4 Hz, 1H), 7.29–7.24 (m, 2H), 7.23–7.12 (m, 5H), 7.05 (d, *J* = 8.0 Hz, 1H), 7.00 (s, 1H), 6.90 (s, 1H), 2.67 (t, *J* = 7.5 Hz, 2H), 1.57–1.50 (m, 2H), 1.30–1.19 (m, 6H), 0.88 (t, *J* = 6.7 Hz, 3H). ^{13}C NMR (101 MHz, CDCl$_3$) δ 141.5, 141.0, 140.2, 138.6, 136.0, 133.0, 132.5, 128.5, 128.0, 128.0, 127.3, 125.8, 123.8, 122.7, 122.1, 120.7, 120.5, 111.1, 103.7, 35.5, 31.6, 31.4, 28.5, 22.5, 14.0.HRMS (EI) m/z: [M]$^+$ calcd for $C_{28}H_{27}NS$ 409.1864; found 409.1866.

2-phenyl-1-(3-(p-tolyl)naphthalen-1-yl)-1H-pyrrolo[2,3-c]pyridine (75c)

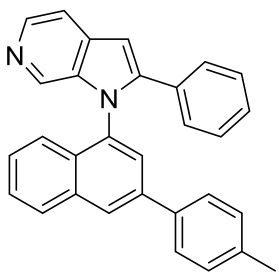

The title compound was prepared according to the general procedure and purified by column chromatography on silica gel and eluted with petroleum ether/ethyl acetate(5/1) to afford a light yellow solid in 85% yield (69.7 mg). mp 199–200 °C. ^{1}H NMR (400 MHz, $CDCl_3$) δ 8.42–8.24 (m, 2H), 8.14 (s, 1H), 7.98 (d, *J* = 8.1 Hz, 1H), 7.65 (s, 1H), 7.58–7.44 (m, 3H), 7.37 (s, 2H), 7.30 (d, *J* = 6.7 Hz, 2H), 7.26–7.20 (m, 2H), 7.20–7.09 (m, 3H), 6.94 (s, 1H), 2.39 (s, 3H). ^{13}C NMR (101 MHz, $CDCl_3$) δ 145.5, 139.6, 138.2, 137.7, 136.7, 134.6, 134.4, 134.3, 133.0, 131.3, 129.8, 129.6, 128.6, 128.5, 128.3, 128.3, 127.2, 127.1, 127.0, 126.5, 126.4, 122.8, 114.7, 102.3, 21.0. HRMS (EI) m/z: $[M]^+$ calcd for $C_{30}H_{22}N_2$ 410.1783; found 410.1785.

2-phenyl-1-(5-phenylbenzo[b]thiophen-7-yl)-1H-pyrrolo[2,3-c]pyridine(76c)

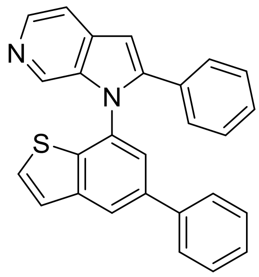

The title compound was prepared according to the general procedure and purified by column chromatography on silica gel and eluted with petroleum ether/dichloromethane (10/1) to afford a white powder in 79% yield (63.5 mg). mp 149–150 °C. ^{1}H NMR (400 MHz, $CDCl_3$) δ 8.50 (s, 1H), 8.37 (s, 1H), 8.10 (s, 1H), 7.64 (s, 1H), 7.52 (d, *J* = 7.2 Hz, 2H), 7.47 (s, 1H), 7.46–7.39 (m, 4H), 7.38–7.32 (m, 3H), 7.24–7.17(m, 3H), 6.90 (s, 1H). ^{13}C NMR (101 MHz, $CDCl_3$) δ 144.3, 142.1, 139.9, 138.9, 137.2, 134.1, 133.4, 132.4, 131.3, 128.8, 128.4, 128.3, 128.1, 127.6, 127.2, 124.5, 124.0, 122.1, 103.0. HRMS (EI) m/z: $[M]^+$ calcd for $C_{27}H_{18}N_2S$ 402.1191; found 402.1193.

2-phenyl-1-(5-phenylbenzofuran-7-yl)-1H-pyrrolo[2,3-c]pyridine (77c)

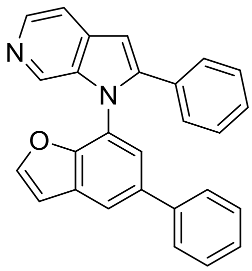

The title compound was prepared according to the general procedure and purified by column chromatography on silica gel and eluted with petroleum ether/dichloromethane (10/1) to afford a white powder in 85% yield (65.6 mg). mp 135–136 °C. ^{1}H NMR (400 MHz, $CDCl_3$) δ 8.57 (s, 1H), 8.41–8.32 (m, 1H), 7.84 (s, 1H), 7.65–7.60 (m, 1H), 7.53 (s, 1H), 7.48–7.44 (m, 2H), 7.44–7.38 (m, 3H), 7.37–7.31 (m, 3H), 7.29–7.21 (m, 3H), 6.95–6.81 (m, 2H). ^{13}C NMR (101 MHz, $CDCl_3$) δ 149.0, 146.3, 144.6, 140.2, 139.9, 137.3, 135.6, 134.1, 133.4, 131.4, 130.0, 128.8, 128.6, 128.3, 128.2, 127.3, 127.3, 123.4, 121.9, 119.7, 114.8, 107.2, 103.0. HRMS (EI) m/z: $[M]^+$ calcd for $C_{27}H_{18}N_2O$ 386.1419; found 386.1418.

1-(5-(pyridin-3-yl)benzo[b]thiophen-7-yl)-2-(thiophen-2-yl)-1H-pyrrolo[2,3-c]pyridine (78c)

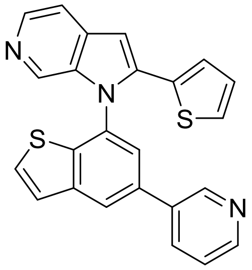

The title compound was prepared according to the general procedure and purified by column chromatography on silica gel and eluted with petroleum ether/dichloromethane (10/1) to afford a white powder in 59% yield (48.3 mg). mp 136–136 °C. ^{1}H NMR (400 MHz, $CDCl_3$) δ 8.86 (s, 1H), 8.80–8.20 (m, 3H), 8.16–8.13 (m, 1H), 7.84 (d, *J* = 7.9 Hz, 1H), 7.70–7.55 (m, 1H), 7.50–7.47 (m, 3H), 7.35 (s, 1H), 7.17 (dd, *J* = 5.1, 2.9 Hz, 1H), 7.03 (dd, *J* = 5.1, 1.2 Hz, 1H), 6.95–6.93 (m, 1H), 6.90 (s, 1H). ^{13}C NMR (101 MHz, $CDCl_3$) δ 148.7, 148.2, 142.2, 139.9, 139.1, 135.6, 134.4, 133.1, 132.7, 131.4, 129.5, 129.5, 128.8, 128.3, 127.1, 125.8, 125.7, 124.4, 123.6, 123.6, 122.5, 102.2. HRMS (EI) m/z: $[M]^+$ calcd for $C_{24}H_{15}N_3S_2$ 409.0707; found 409.0710.

1-(6-methoxy-2,7-dimethyl-3-phenylnaphthalen-1-yl)-2-phenyl-1H-indole (79c)

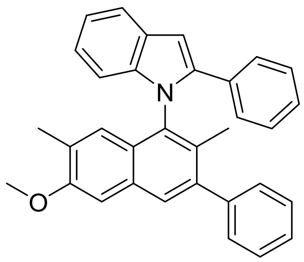

The title compound was prepared according to the general procedure and purified by column chromatography on silica gel and eluted with petroleum ether/dichloromethane(10/1) to afford a white powder in 85% yield (77.0 mg). mp 188–189 °C. The ee (40% ee) of **79c** was determined by HPLC analysis using a Daicel CHIRALPACK OD column; 1% *i*-Pr-OH in hexane; flow rate, 1.0 mL/min; 35 °C; retention times: 6.1 min (minor), 7.1 min (major). ^{1}H NMR (400 MHz, $CDCl_3$) δ 7.77 (d, J = 7.8 Hz, 1H), 7.71 (s, 1H), 7.43–7.31 (m, 3H), 7.30–7.25 (m, 4H), 7.23–7.06 (m, 7H), 6.99 (s, 1H), 6.79 (d, J = 8.1 Hz, 1H), 3.94 (s, 3H), 2.20 (s, 3H), 1.68 (s, 3H). ^{13}C NMR (101 MHz, $CDCl_3$) δ 157.0, 141.9, 141.6, 140.9, 138.9, 132.8, 132.7, 132.3, 131.1, 130.1, 129.3, 128.3, 128.1, 128.0, 127.8, 127.6, 127.3, 127.0, 126.6, 124.2, 122.2, 120.4, 120.4, 111.2, 104.5, 102.3, 55.3, 17.1, 15.8 .HRMS (EI) m/z: $[M]^+$ calcd for $C_{33}H_{27}NO$ 453.2093; found 453.2092.

Racemic:

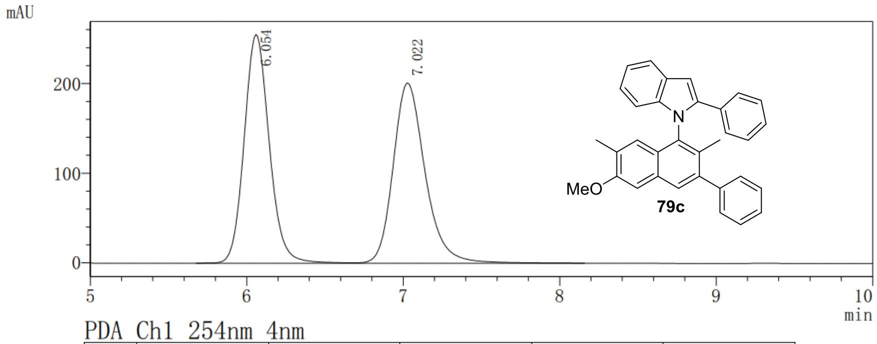

PDA Ch1 254nm 4nm

Peak	Ret Time	Width	Area	Height	Area %
1	6.054	0.300	2838998	255173	49.883
2	7.022	0.372	2852361	201014	50.117

Enantioselective:

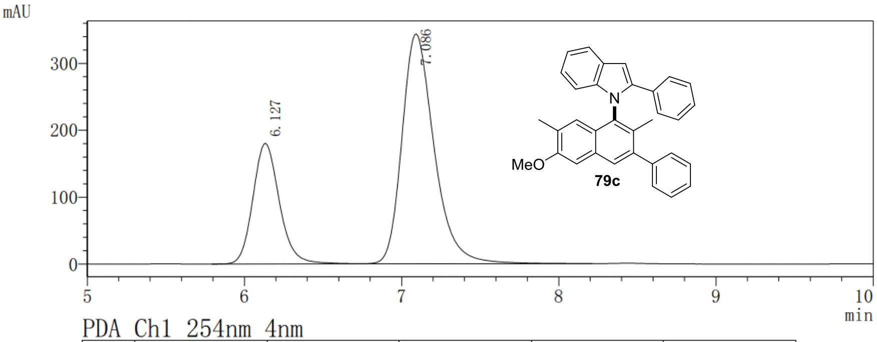

PDA Ch1 254nm 4nm

Peak	Ret Time	Width	Area	Height	Area %
1	6.127	0.314	2111986	180253	30.099
2	7.086	0.374	4904769	343611	69.901

1-(6-methoxy-2,7-dimethyl-3-phenylnaphthalen-1-yl)-2-(o-tolyl)-1H-indole (80c)

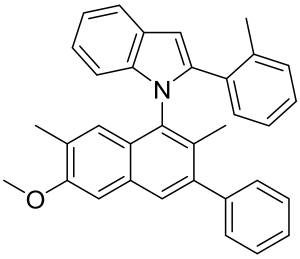

The title compound was prepared according to the general procedure and purified by column chromatography on silica gel and eluted with petroleum ether/dichloromethane(10/1) to afford a white powder in 70% yield (65.4 mg). mp 197–198 °C. The ee (56% ee) of **80c** was determined by HPLC analysis using a Daicel CHIRALPACK OD column; 1% *i*-Pr-OH in hexane; flow rate, 1.0 mL/min; 35 °C; retention times: 9.2 min (minor), 10.0 min (major). ^{1}H NMR (400 MHz, $CDCl_3$) δ 7.82 (d, *J* = 7.7 Hz, 1H), 7.64 (s, 1H), 7.46–7.40 (m, 2H), 7.40–7.34 (m, 1H), 7.31 (d, *J* = 7.4 Hz, 2H), 7.25–7.12 (m, 3H), 7.10–7.04 (m, 2H), 7.01 (s, 1H), 6.94 (d, *J* = 7.7 Hz, 1H), 6.90–6.80 (m, 3H), 3.92 (m, 3H), 2.54 (s, 3H), 2.23 (s, 3H), 1.86 (s, 3H). ^{13}C NMR (101 MHz, $CDCl_3$) δ 156.8, 141.7, 140.6, 140.2, 137.9, 136.7, 132.2, 132.1, 131.7, 130.9, 130.4, 129.9, 129.5, 129.2, 128.0, 127.9, 127.5, 126.9, 126.6, 124.7, 124.3, 121.9, 120.2, 120.2, 111.2, 104.4, 55.2, 20.9, 16.9, 16.3. HRMS (EI) m/z: $[M]^+$ calcd for $C_{34}H_{29}NO$ 467.2249; found 467.2247.

Racemic:

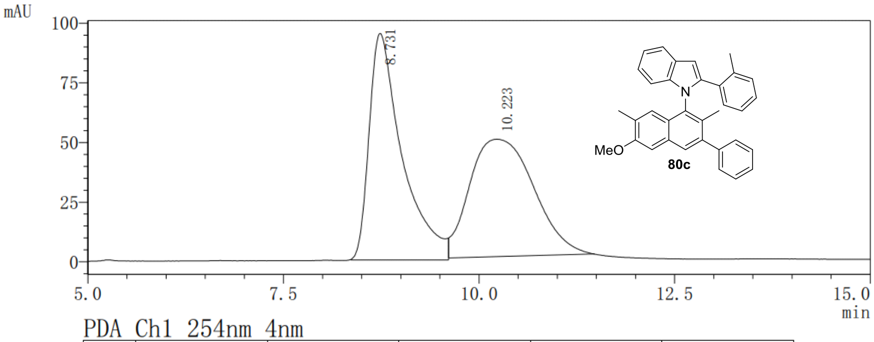

PDA Ch1 254nm 4nm

Peak	Ret Time	Width	Area	Height	Area %
1	8.731	0.701	2789350	94986	49.847
2	10.223	1.435	2806469	49208	50.153

Enantioselective:

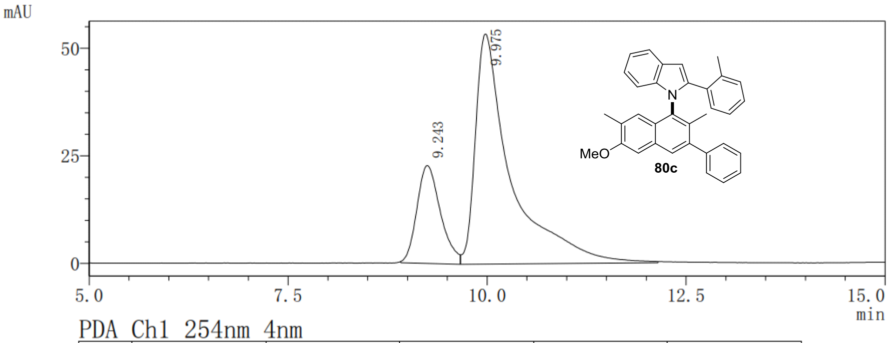

PDA Ch1 254nm 4nm

Peak	Ret Time	Width	Area	Height	Area %
1	9.243	0.546	469462	22768	21.933
2	9.975	0.627	1670981	53511	78.067

1-(3-chlorophenyl)-1-(6-methoxy-2,7-dimethyl-3-phenylnaphthalen-1-yl)-1H-indole (81c)

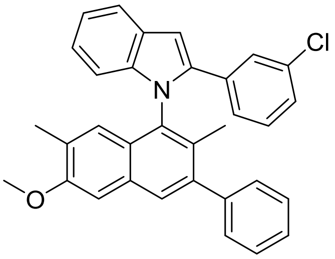

The title compound was prepared according to the general procedure and purified by column chromatography on silica gel and eluted with petroleum ether/dichloromethane(10/1) to afford a white powder in 75% yield (73.1 mg). mp 185–186 °C. The ee (43%) of **81c** was determined by HPLC analysis using a Daicel CHIRALPACK OD column; 1% *i*-Pr-OH in hexane; flow rate, 1.0 mL/min; 35 ^{o}C; retention times: 9.0 min (minor), 9.6 min (major). ^{1}H NMR (400 MHz, $CDCl_3$) δ 7.78 (d, *J* = 7.8 Hz, 1H), 7.73 (s, 1H), 7.47–7.40 (m, 2H), 7.39–7.30 (m, 4H), 7.21 (t, *J* = 7.3 Hz, 1H), 7.16–7.09 (m, 3H), 7.06–6.99 (m, 3H), 6.97 (s, 1H), 6.81 (d, *J* = 8.0 Hz, 1H), 3.94 (s, 3H), 2.19 (s, 3H), 1.73 (s, 3H). ^{13}C NMR (101 MHz, $CDCl_3$) δ 157.0, 141.5, 140.9, 140.0, 139.0, 134.4, 133.9, 132.3, 131.1, 130.2, 129.3, 128.1, 128.0, 128.0, 127.6, 127.2, 127.0, 126.4, 125.3, 123.8, 122.7, 120.6, 111.2, 104.6, 103.0, 55.3, 17.1, 15.7. HRMS (EI) m/z: $[M]^+$ calcd for $C_{33}H_{26}ClNO$ 487.1703; found 487.1701.

Racemic:

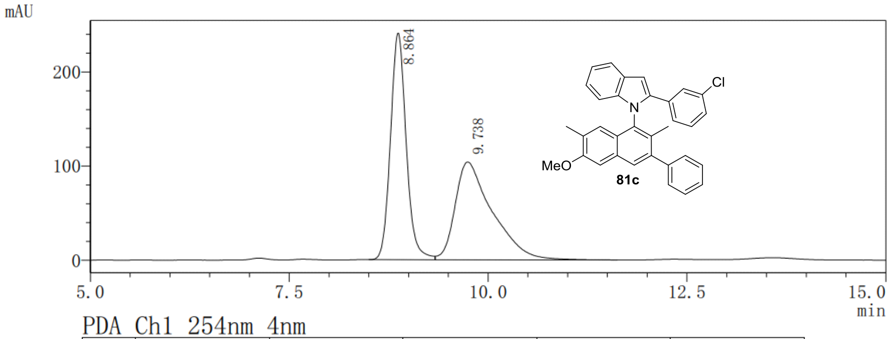

PDA Ch1 254nm 4nm

Peak	Ret Time	Width	Area	Height	Area %
1	8.864	0.365	3316544	240627	49.626
2	9.738	0.793	3366525	103887	50.374

Enantioselective:

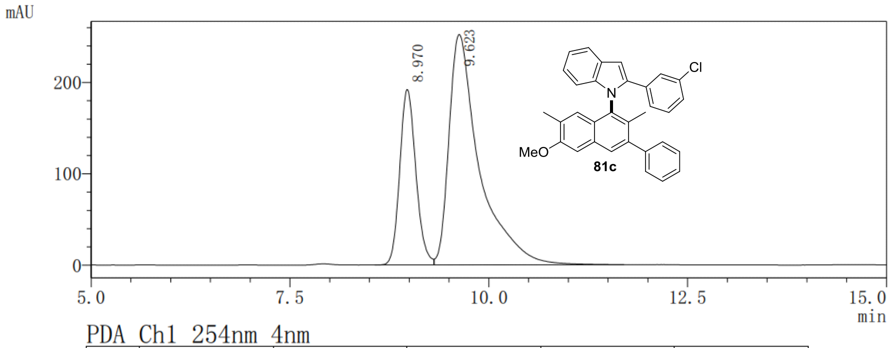

PDA Ch1 254nm 4nm

Peak	Ret Time	Width	Area	Height	Area %
1	8.970	0.380	2715769	191907	28.776
2	9.623	0.601	6721864	252377	71.224

3-methyl-2-phenyl-1H-inden-1-one (1p)[15]

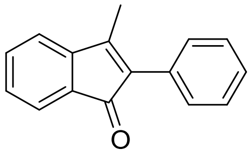

The title compound was prepared according to the general procedure and purified by column chromatography on silica gel and eluted with petroleum ether/dichloromethane(20/1) to afford a sticky yellow liquid. ^{1}H NMR (400 MHz, $CDCl_3$) δ 7.47 (d, *J* = 7.0 Hz, 1H), 7.44–7.36 (m, 5H), 7.35–7.30 (m, 1H), 7.27–7.20 (m, 1H), 7.14 (d, *J* = 7.1 Hz, 1H), 2.30 (s, 3H). ^{13}C NMR (101 MHz, $CDCl_3$) δ 196.3, 154.6, 145.8, 133.5, 133.4, 131.1, 130.3, 129.5, 128.8, 128.2, 127.6, 122.0, 119.3, 12.5.

1-(2-aminophenyl)-2-(p-tolyl)ethan-1-one (1g)[16]

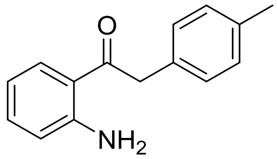

The title compound was prepared according to the general procedure and purified by column chromatography on silica gel and eluted with petroleum ether/dichloromethane(10/1) to afford a crystalline white solid. ^{1}H NMR (400 MHz, $CDCl_3$) δ 7.84 (d, *J* = 8.1 Hz, 1H), 7.28–7.22 (m, 1H), 7.14 (s, 4H), 6.69–6.57 (m, 2H), 6.28 (s, 2H), 4.22 (s, 2H), 2.33 (s, 3H). ^{13}C NMR (101 MHz, $CDCl_3$) δ 200.1, 150.8, 136.2, 134.3, 132.2, 131.5, 129.2, 129.2, 117.5, 117.3, 115.7, 45.6, 21.0. HRMS (EI) m/z: $[M]^+$ calcd for $C_{15}H_{15}NO$ 225.1154.

2-(4-(4-methylbenzyl)-3-(p-tolyl)quinolin-2-yl)aniline (1h)[3]

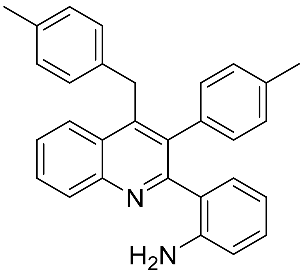

The title compound was prepared according to the general procedure and purified by column chromatography on silica gel and eluted with petroleum ether/dichloromethane(10/1) to afford a light yellow solid, mp 200-201 ºC. ^{1}H NMR (400 MHz, $CDCl_3$) δ 8.19 (d, *J* = 8.4 Hz, 1H), 7.96 (d, *J* = 8.4 Hz, 1H), 7.69 (t, *J* = 7.5 Hz, 1H), 7.48 (t, *J* = 7.5 Hz, 1H), 7.09–6.97 (m, 7H), 6.94 (d, *J* = 7.4 Hz, 2H), 6.80 (d, *J* = 7.5 Hz, 1H), 6.71 (d, *J* = 8.0 Hz, 1H), 6.48 (t, *J* = 7.4 Hz, 1H), 4.39 (s, 2H), 4.29 (s, 2H), 2.30 (d, *J* = 12.8 Hz, 6H). ^{13}C NMR (101 MHz, $CDCl_3$) δ 158.7, 146.8, 144.8, 144.6, 137.0, 136.5, 135.8, 135.3,

10. Copies of ^{1}H, ^{13}C and ^{19}C NMR Spectra of the Products

^{1}H NMR Spectrum of **1c**

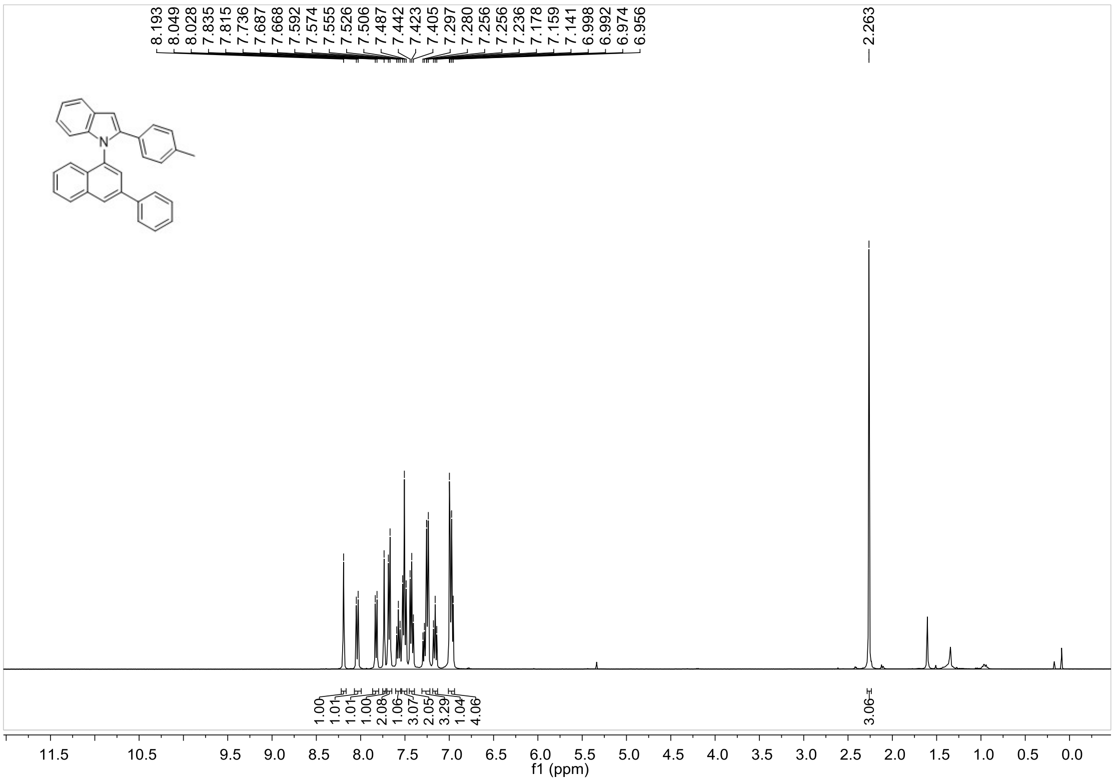

^{13}C NMR Spectrum of **1c**

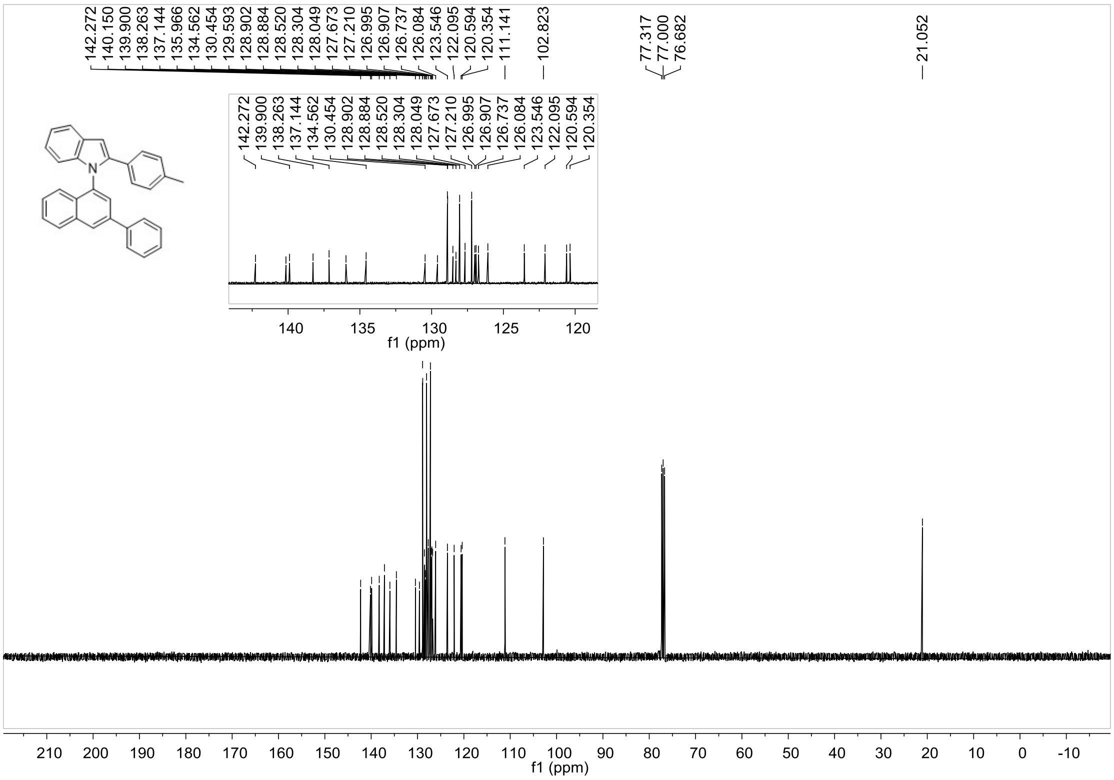

^{1}H NMR Spectrum of **2c**

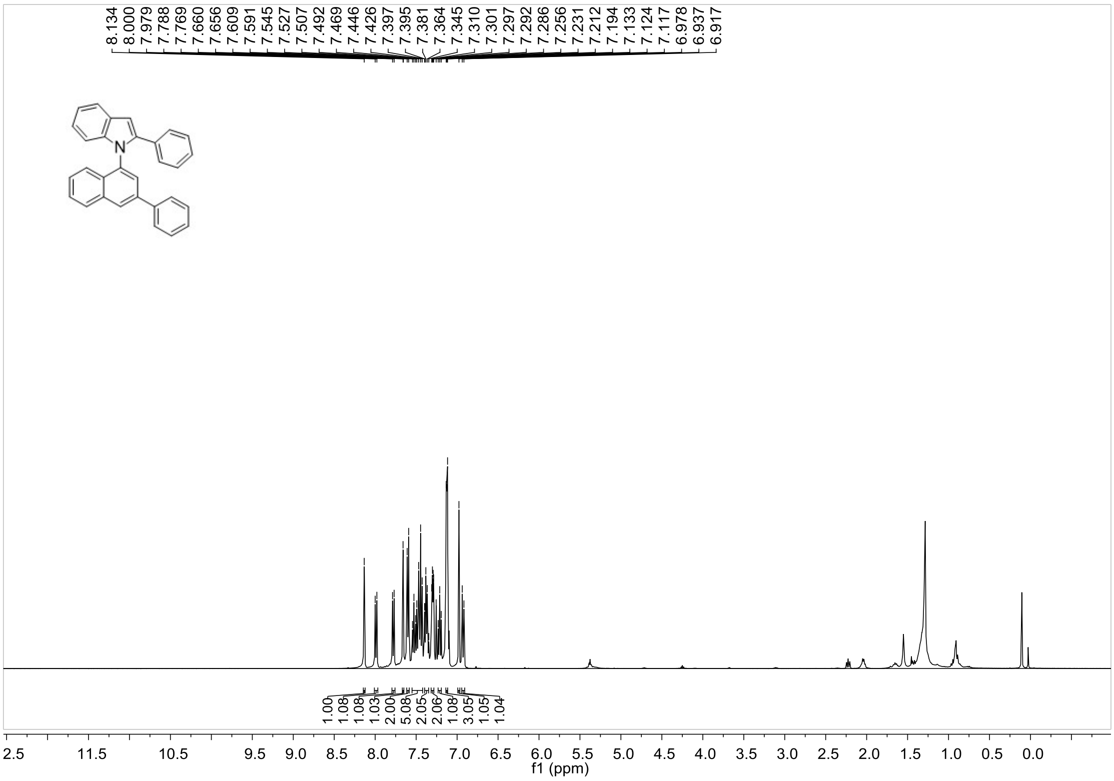

^{13}C NMR Spectrum of **2c**

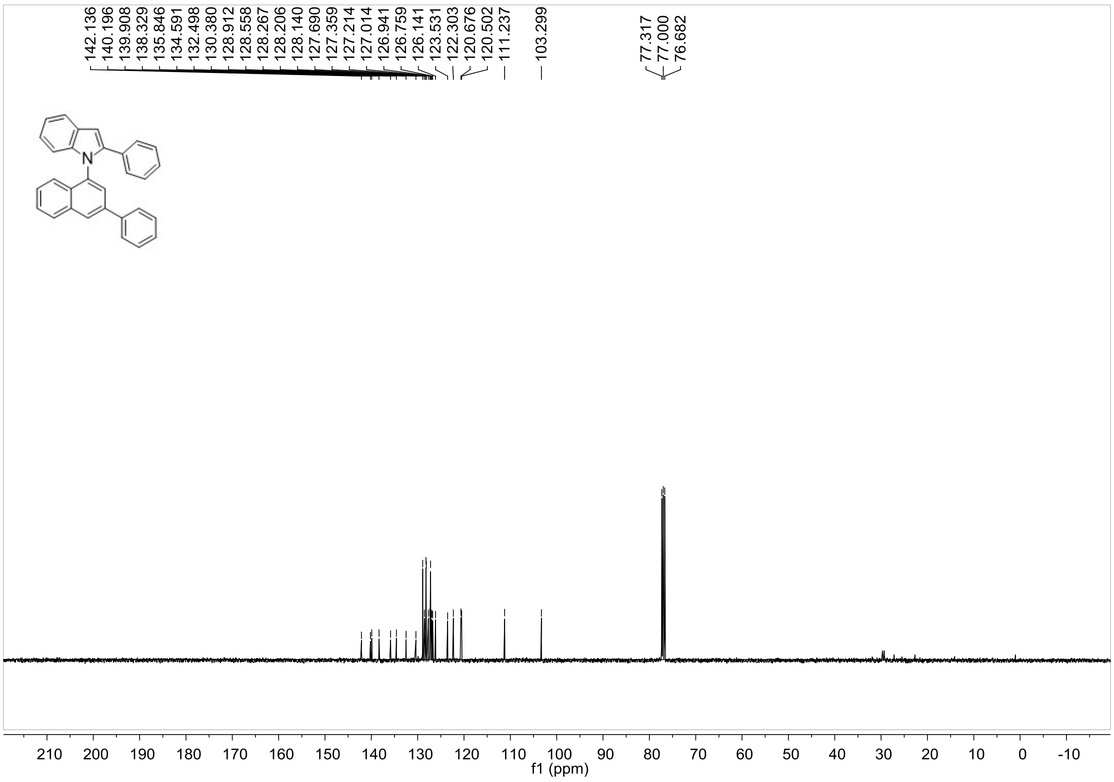

^{1}H NMR Spectrum of **3c**

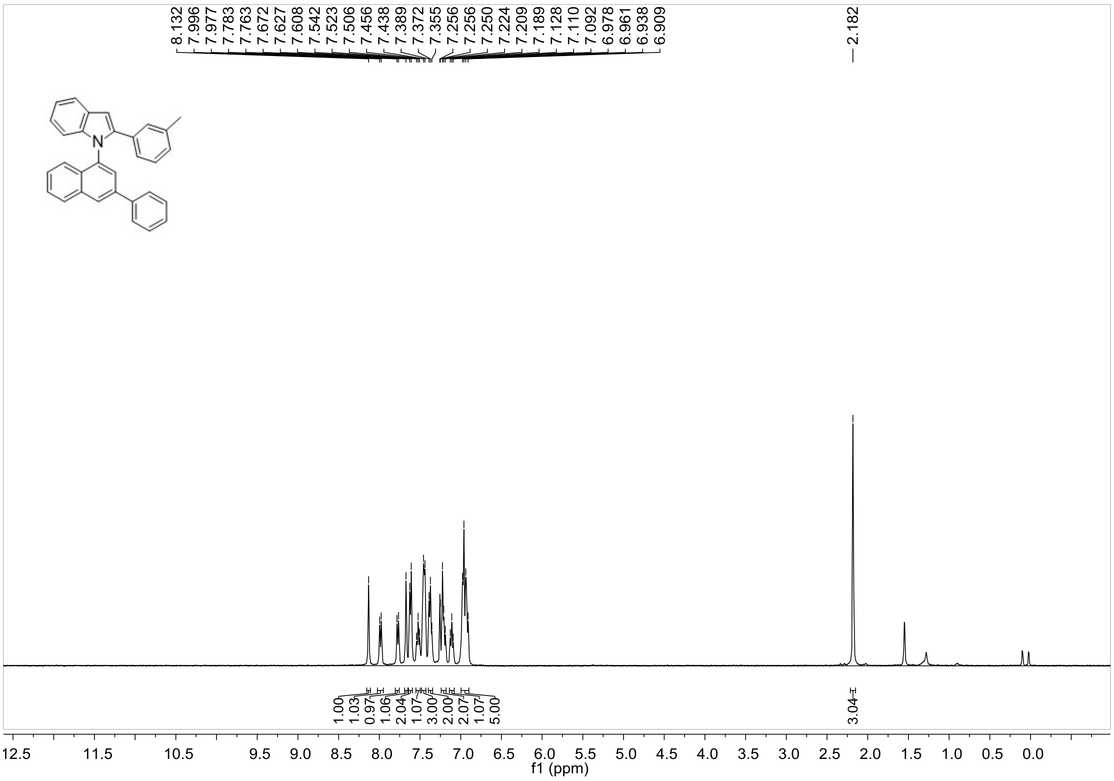

^{13}C NMR Spectrum of **3c**

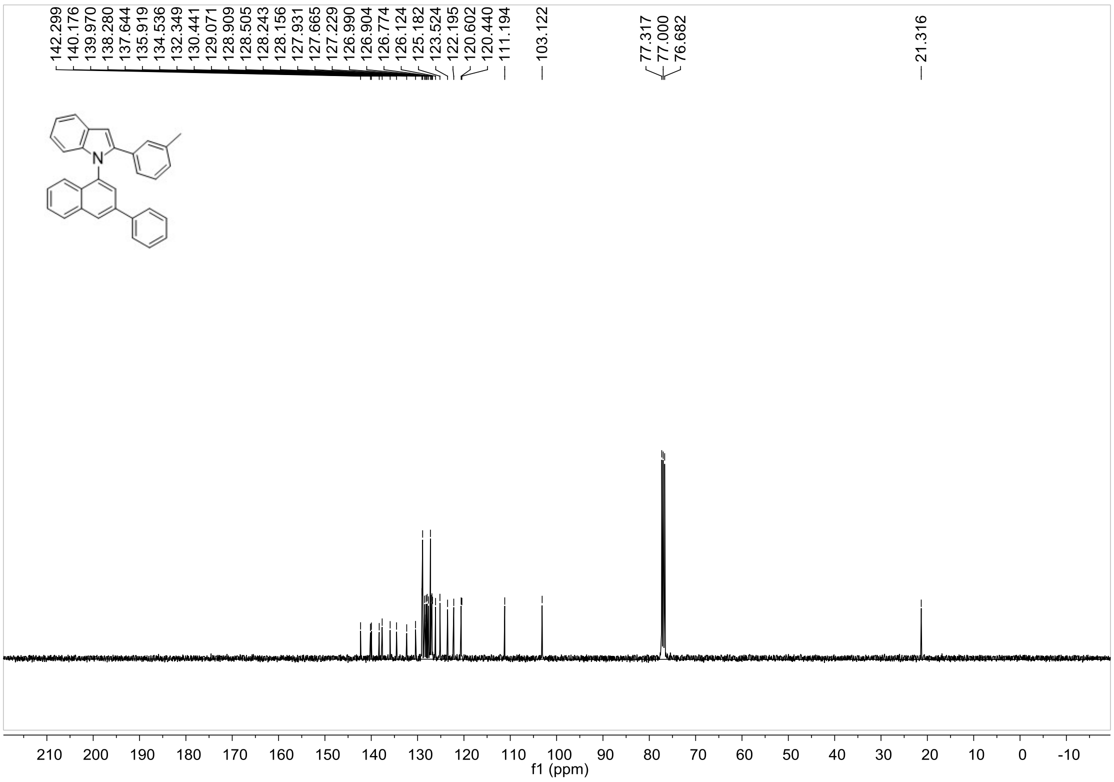

^{1}H NMR Spectrum of **4c**

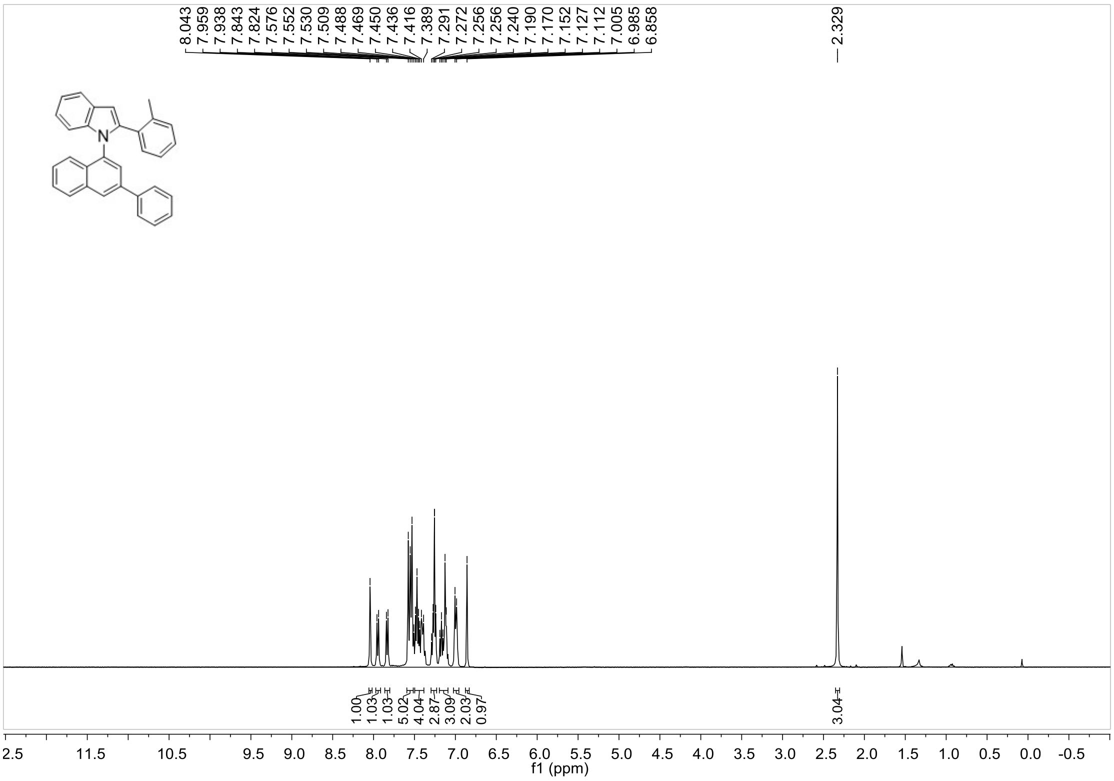

^{13}C NMR Spectrum of **4c**

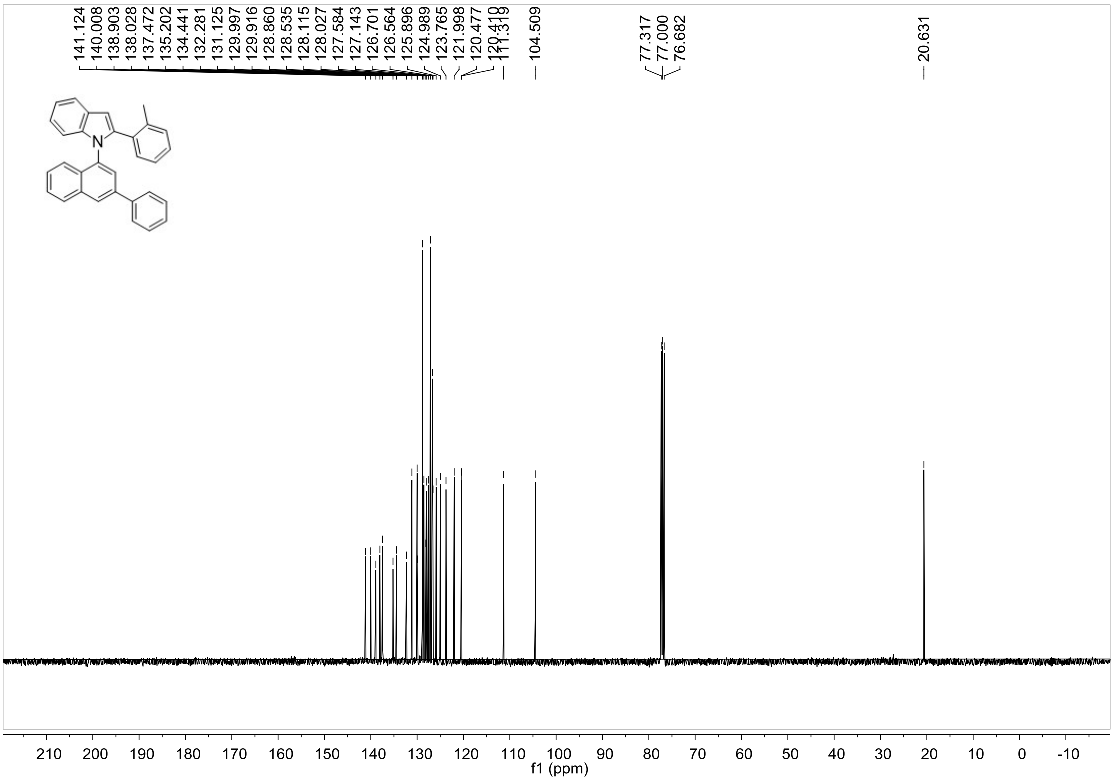

^{1}H NMR Spectrum of **5c**

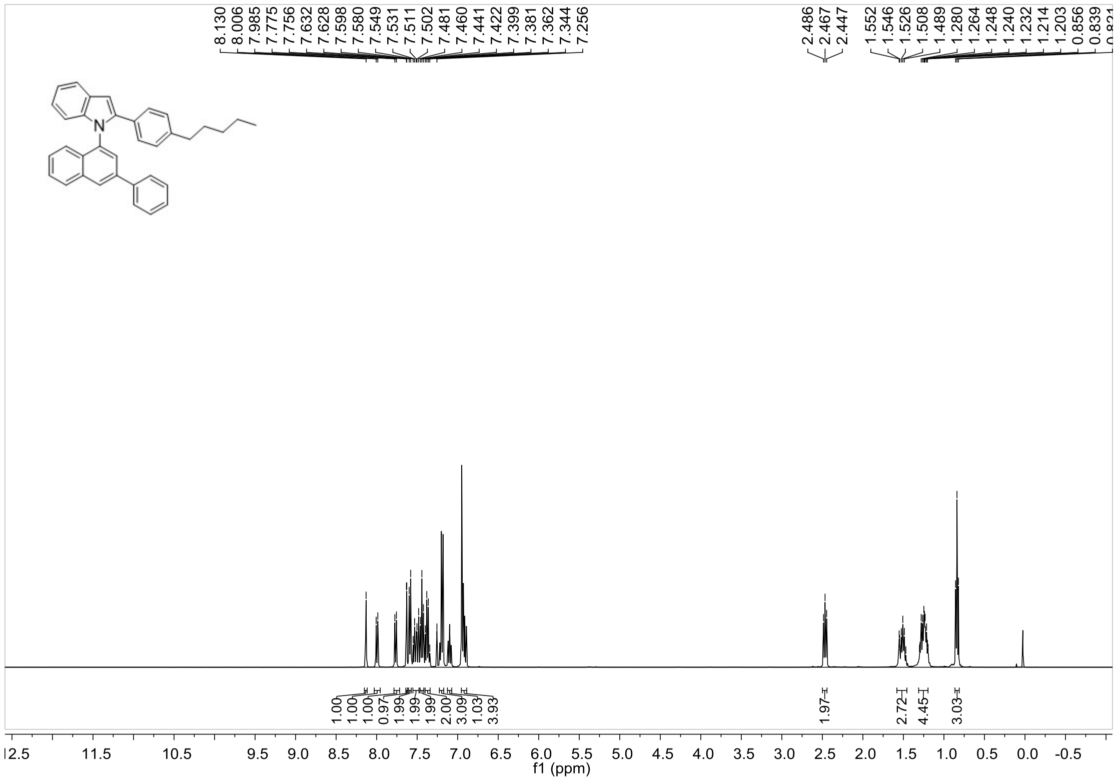

^{13}C NMR Spectrum of **5c**

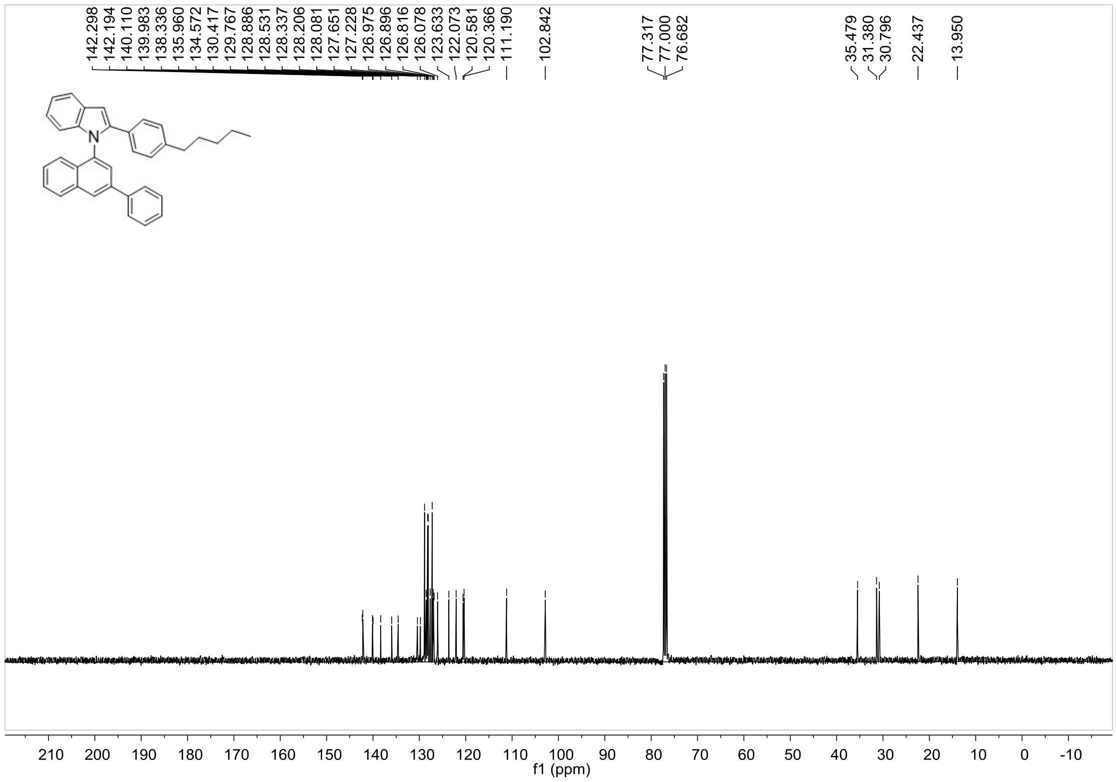

^{1}H NMR Spectrum of **6c**

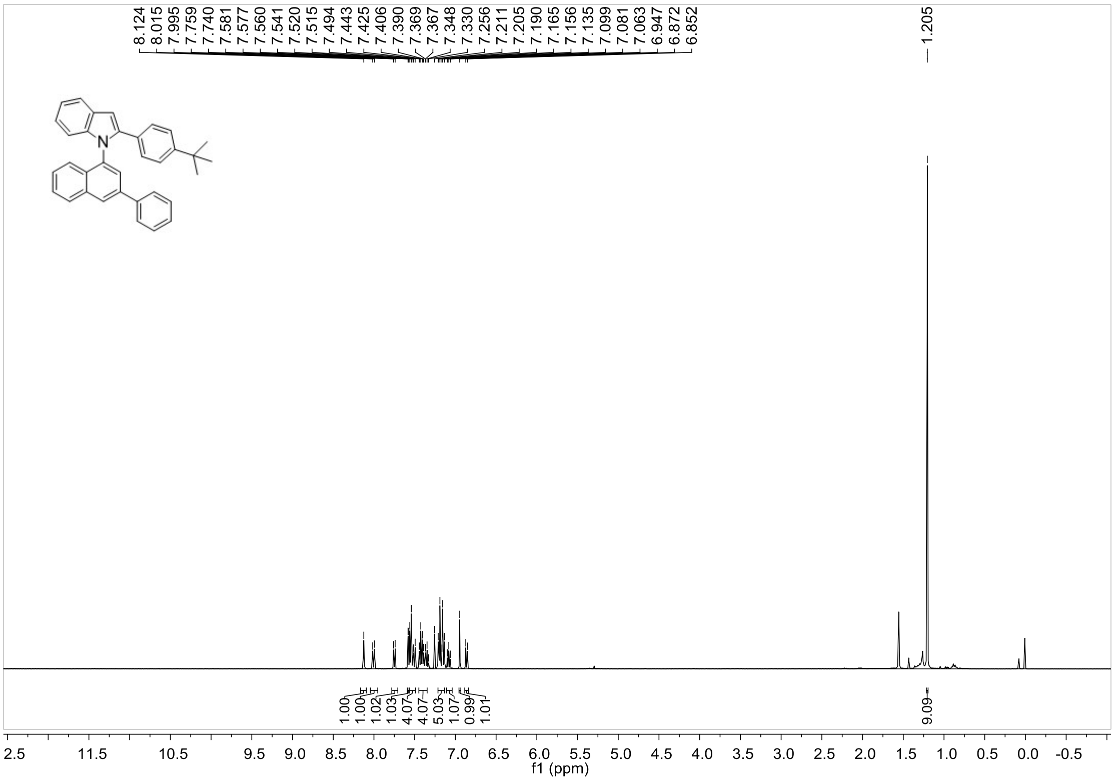

^{13}C NMR Spectrum of **6c**

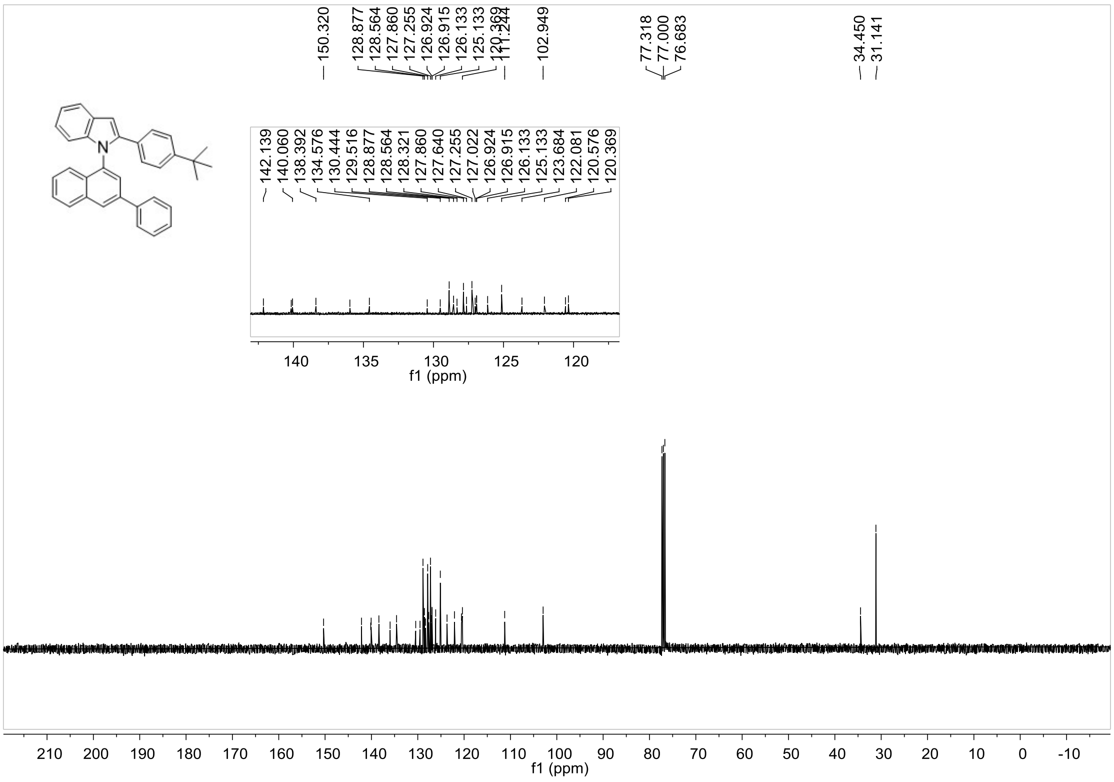

^{1}H NMR Spectrum of **7c**

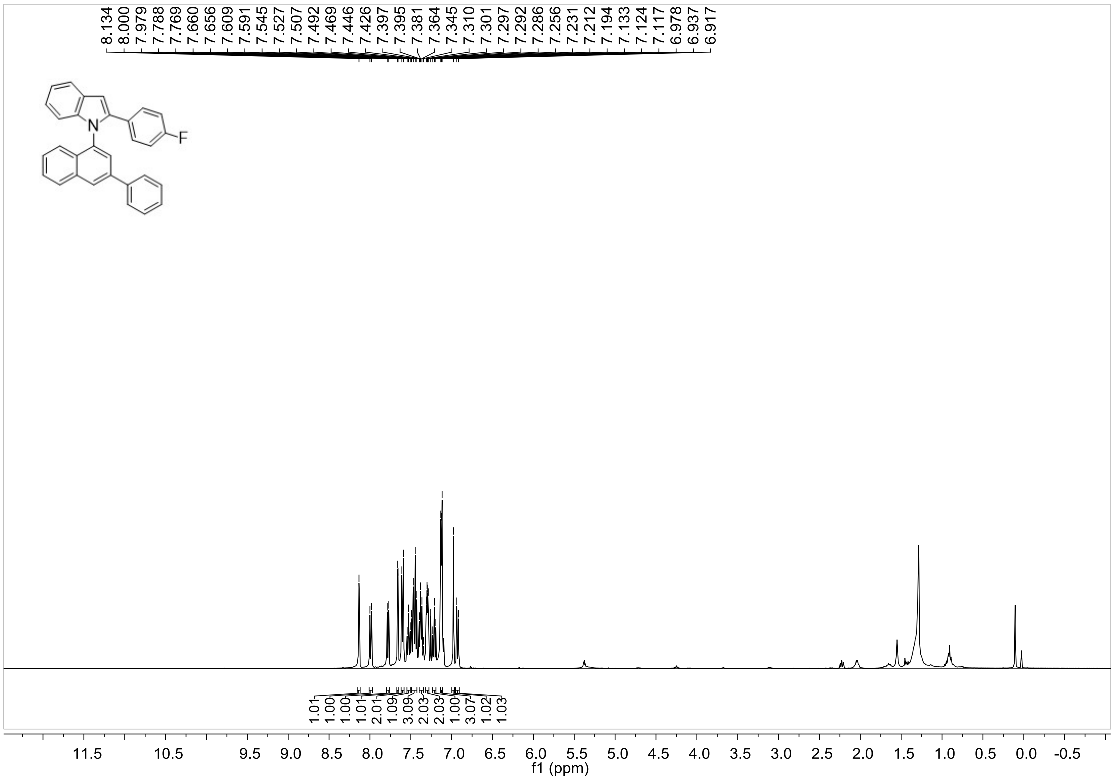

^{13}C NMR Spectrum of **7c**

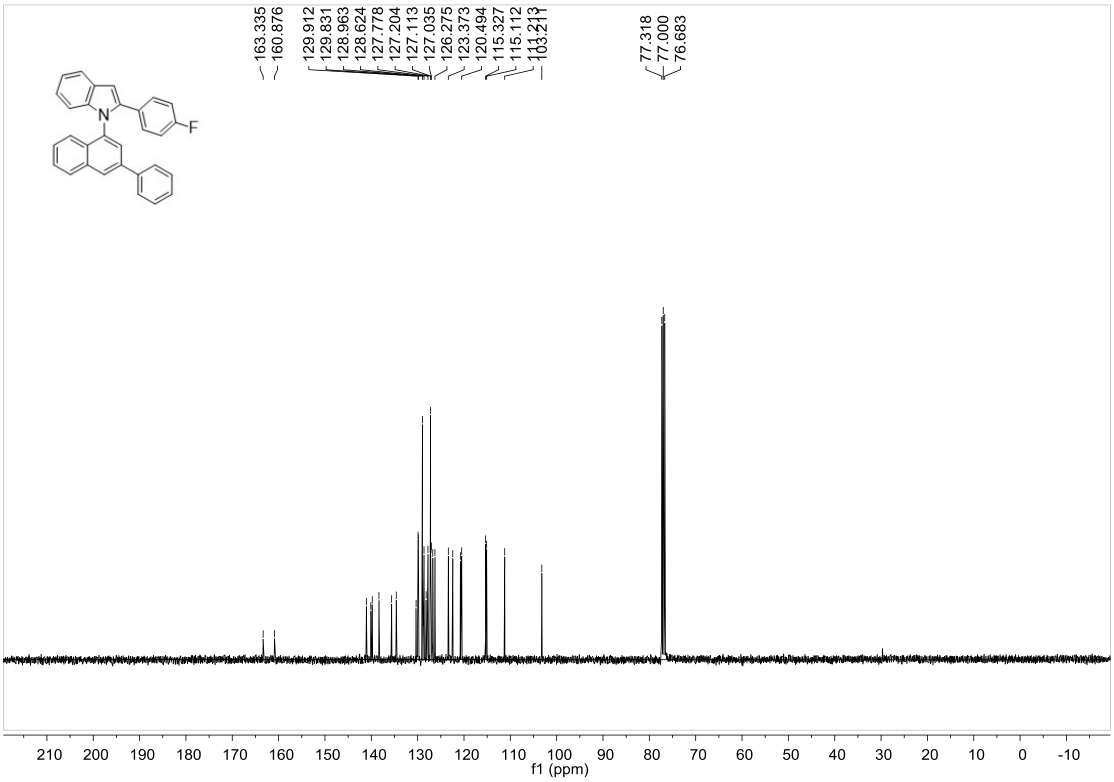

^{19}F NMR Spectrum of **7c**

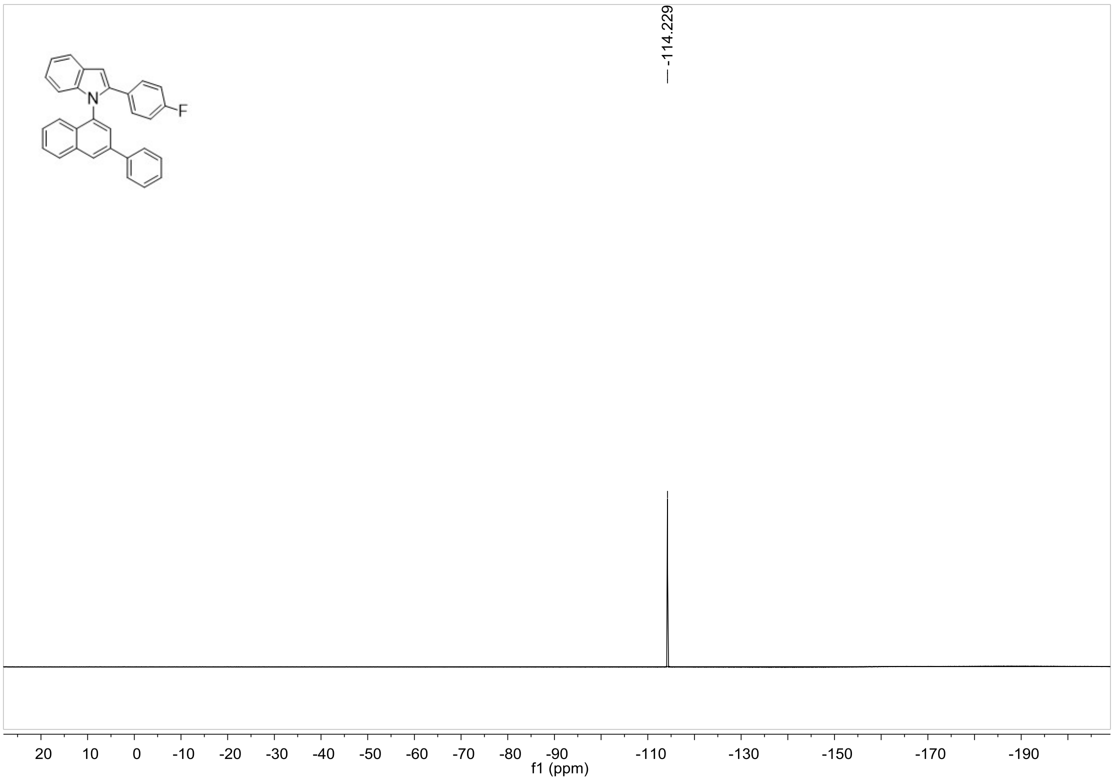

^{1}H NMR Spectrum of **8c**

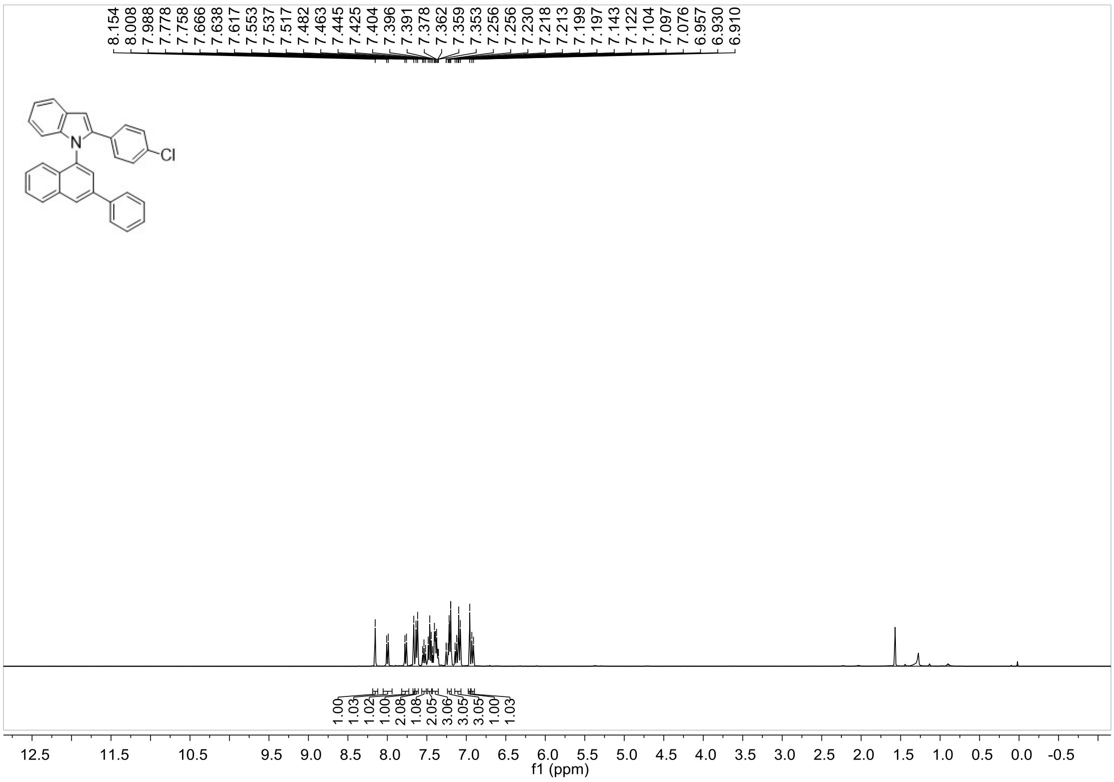

^{13}C NMR Spectrum of **8c**

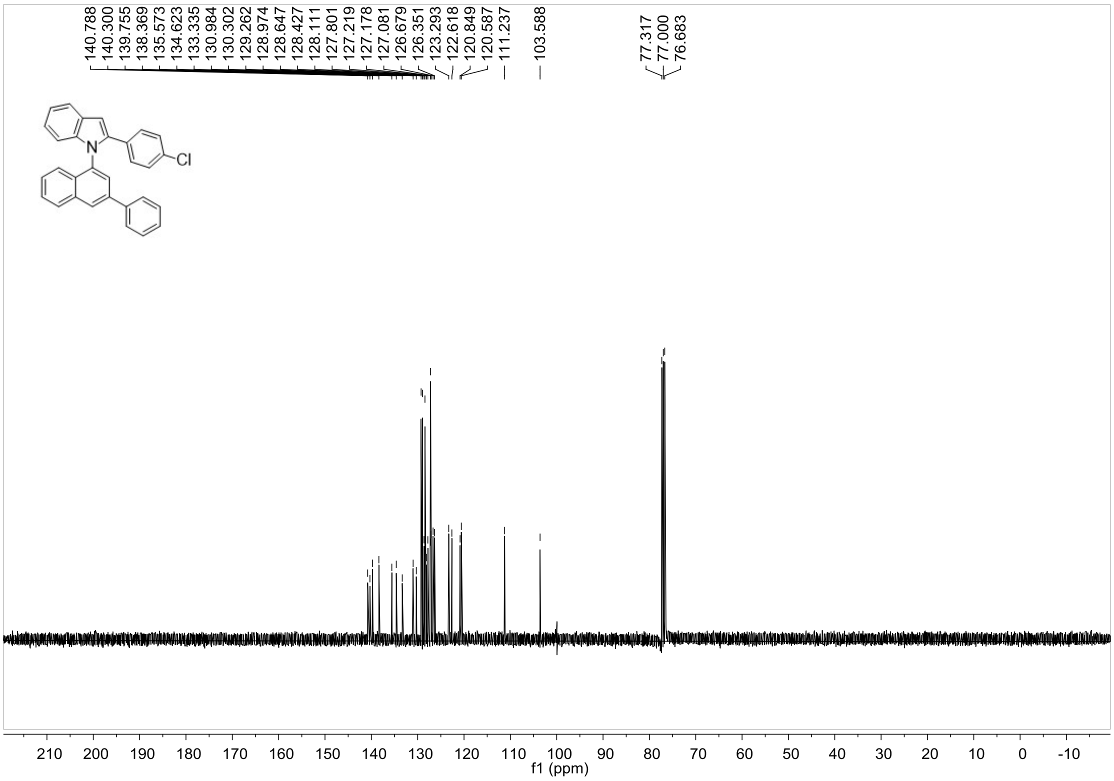

^{1}H NMR Spectrum of **9c**

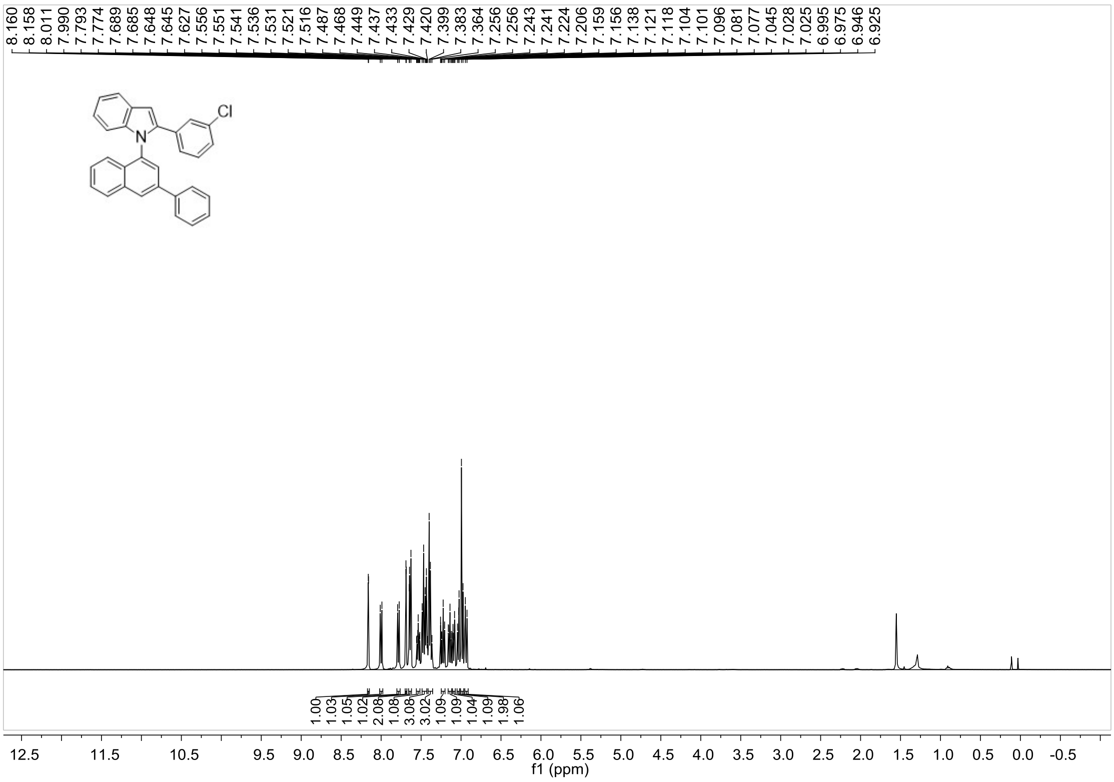

^{13}C NMR Spectrum of **9c**

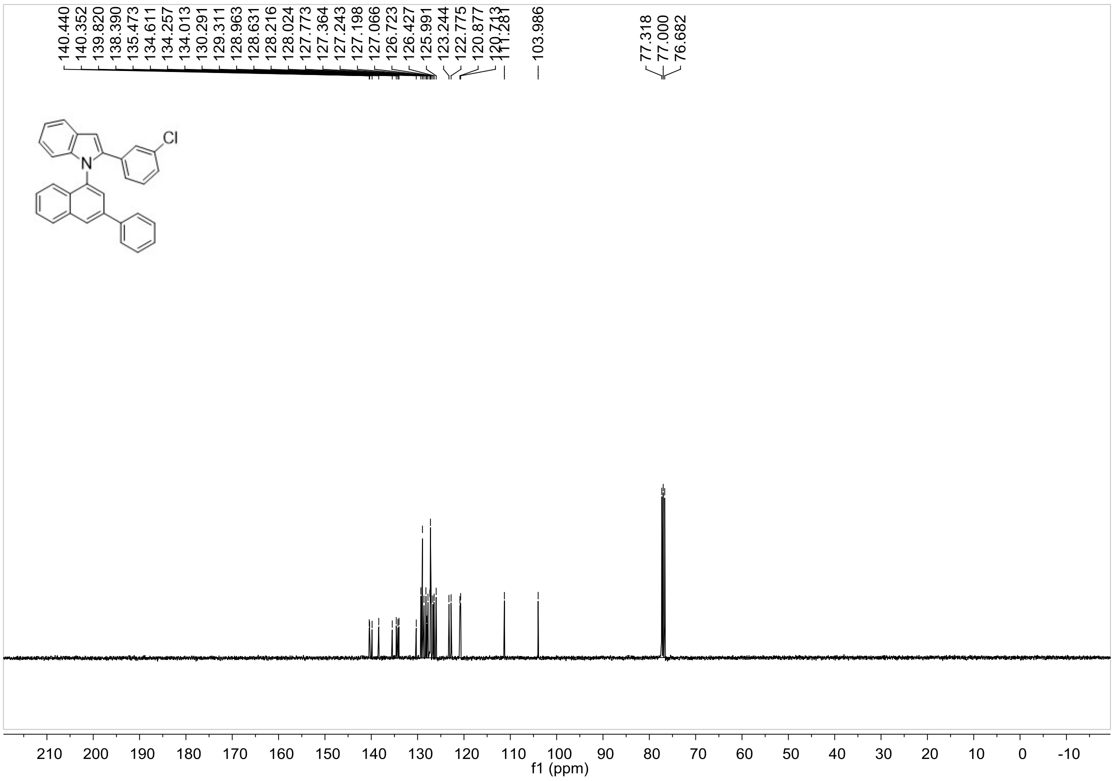

^{1}H NMR Spectrum of **10c**

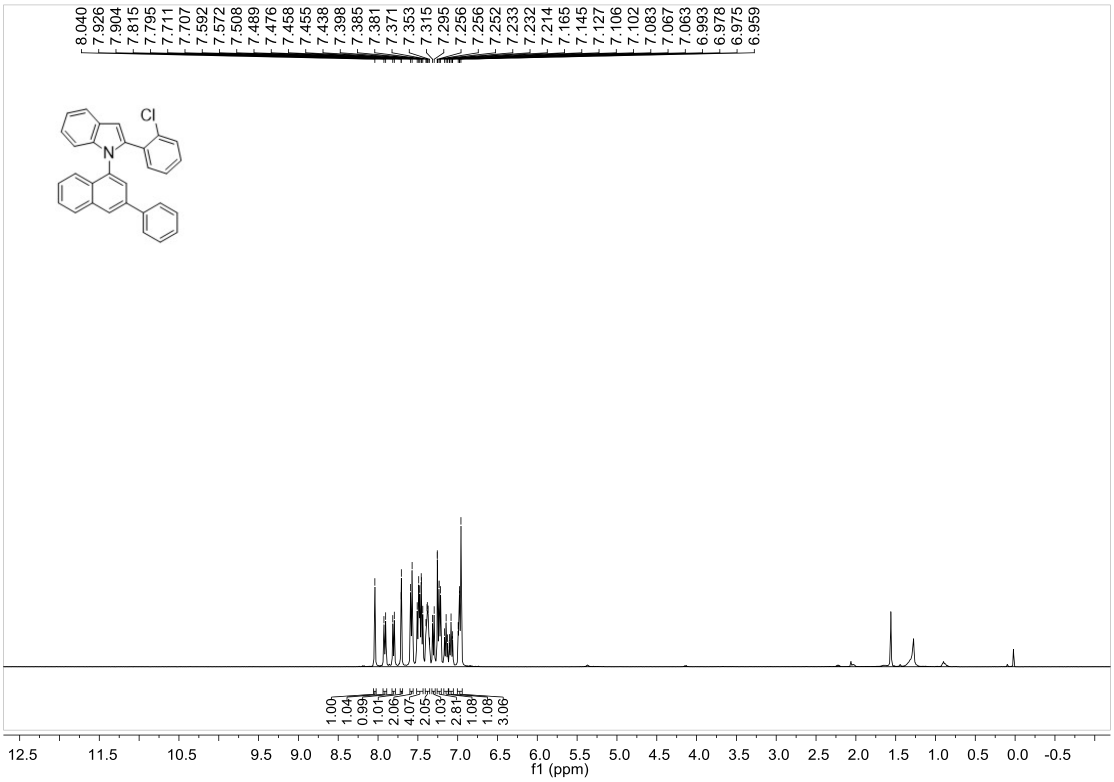

^{13}C NMR Spectrum of **10c**

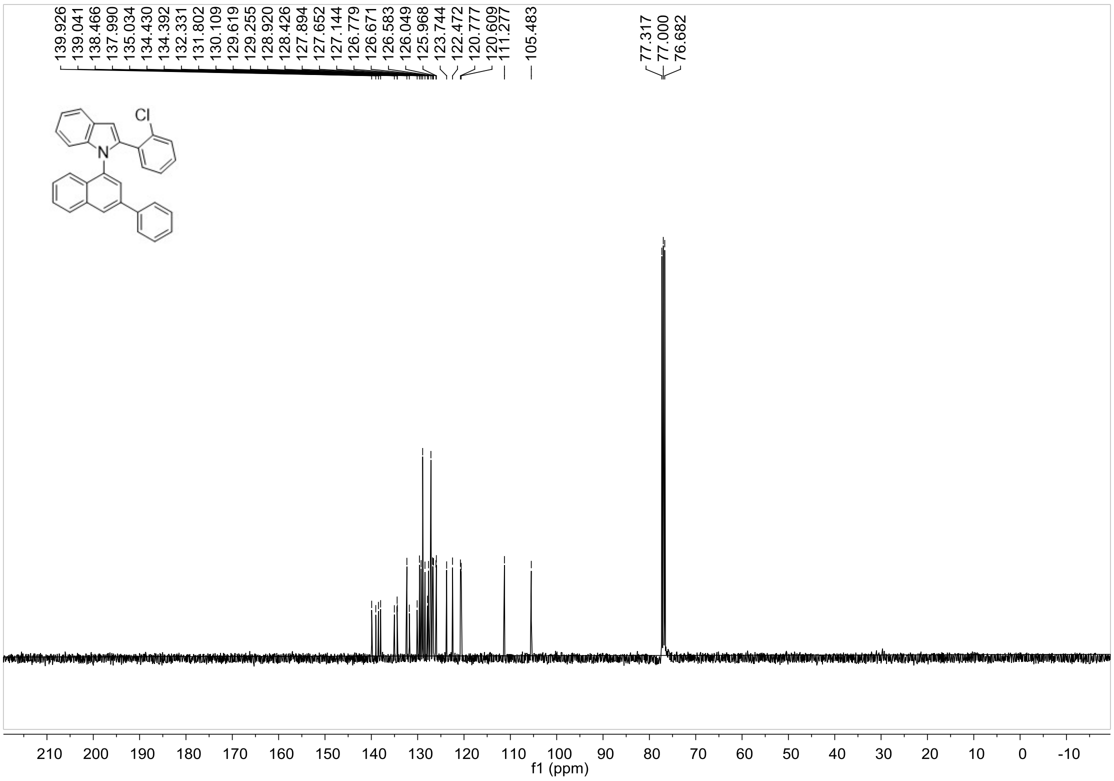

^{1}H NMR Spectrum of **11c**

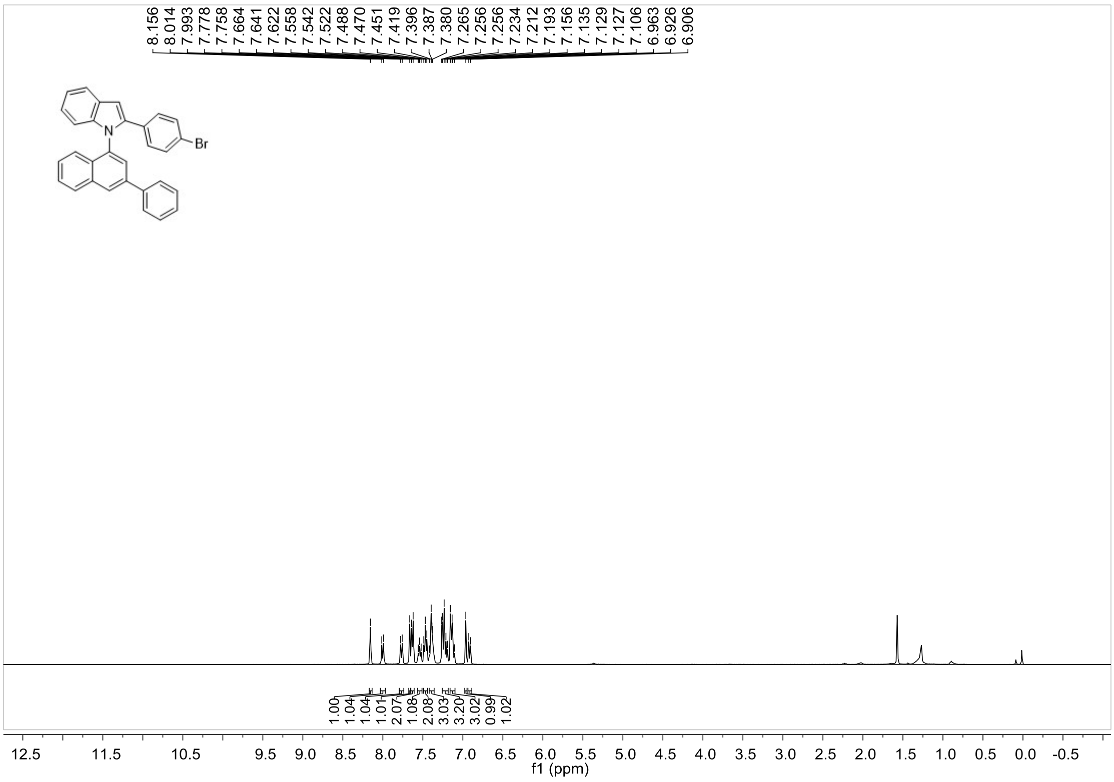

^{13}C NMR Spectrum of **11c**

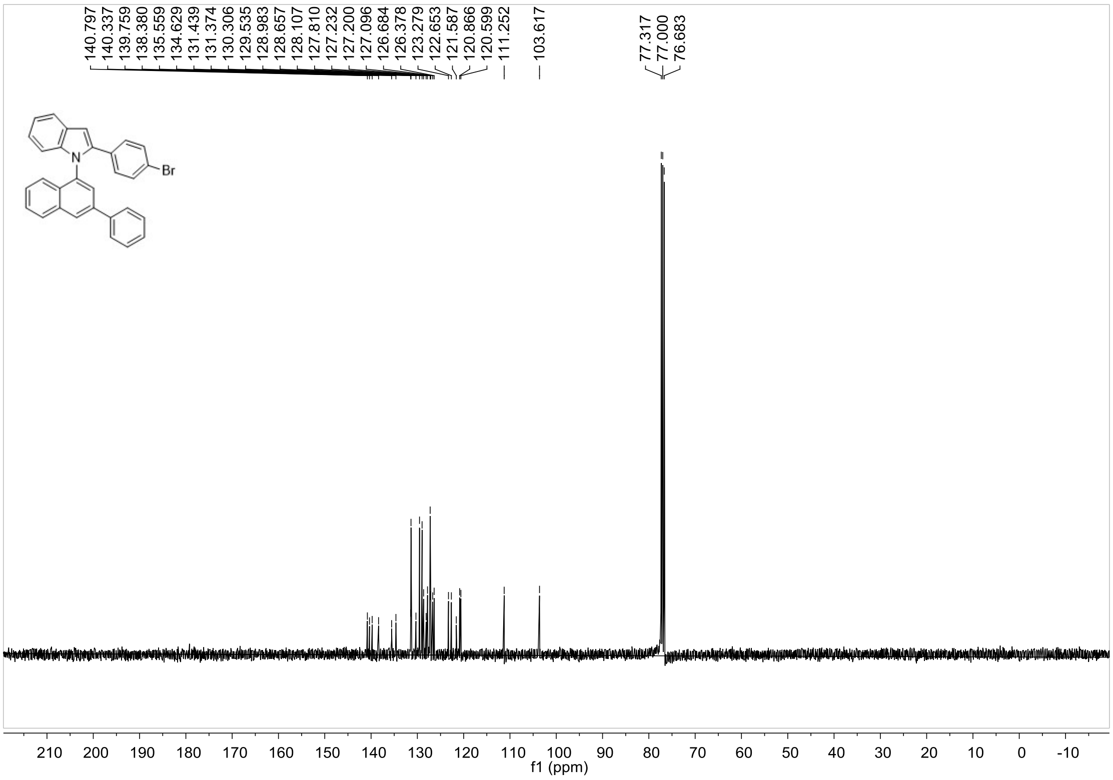

^{1}H NMR Spectrum of **12c**

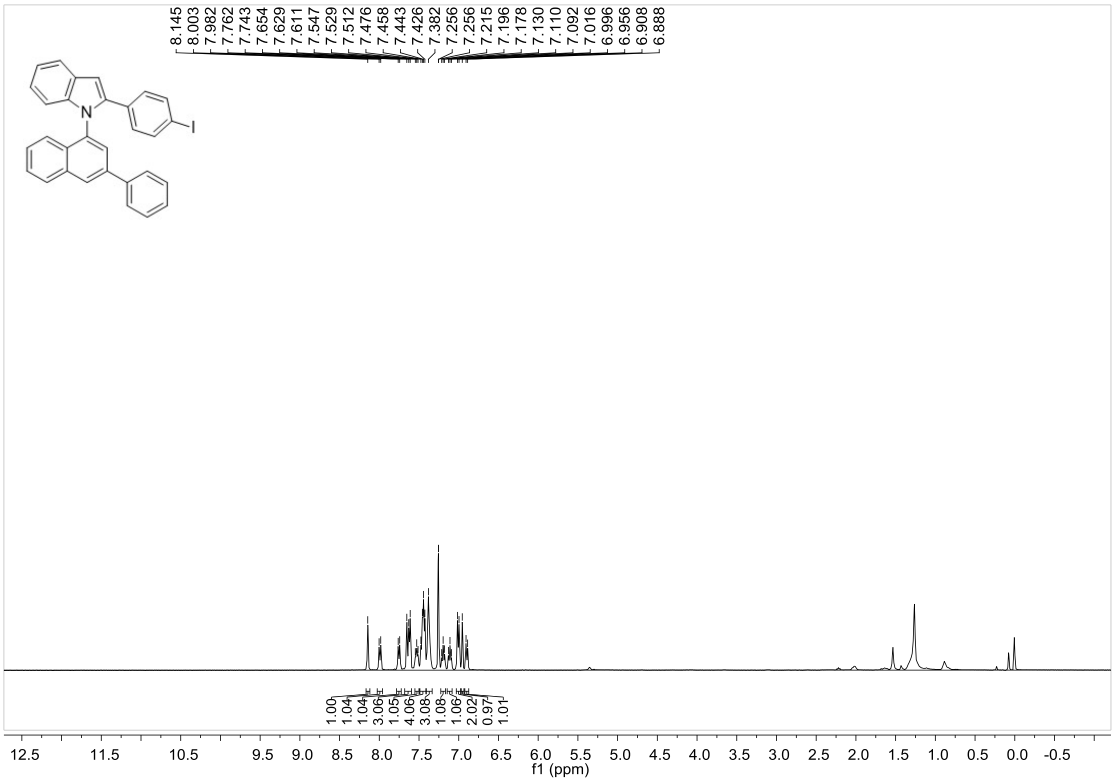

^{13}C NMR Spectrum of **12c**

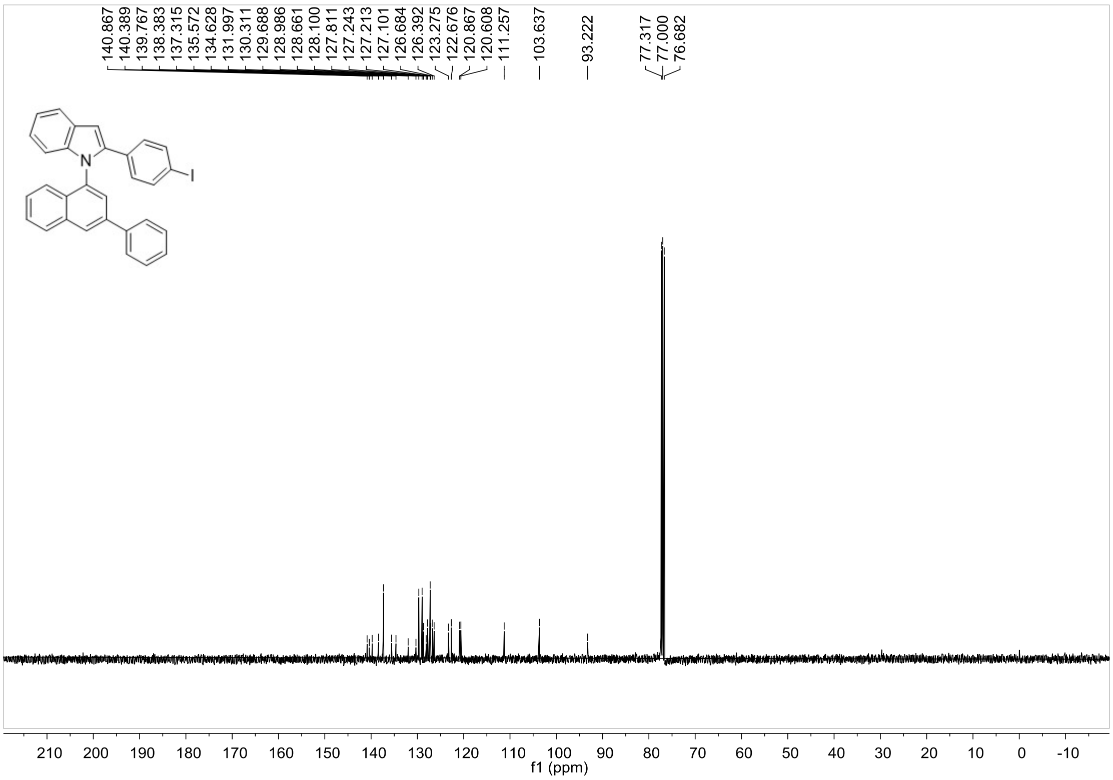

^{1}H NMR Spectrum of **13c**

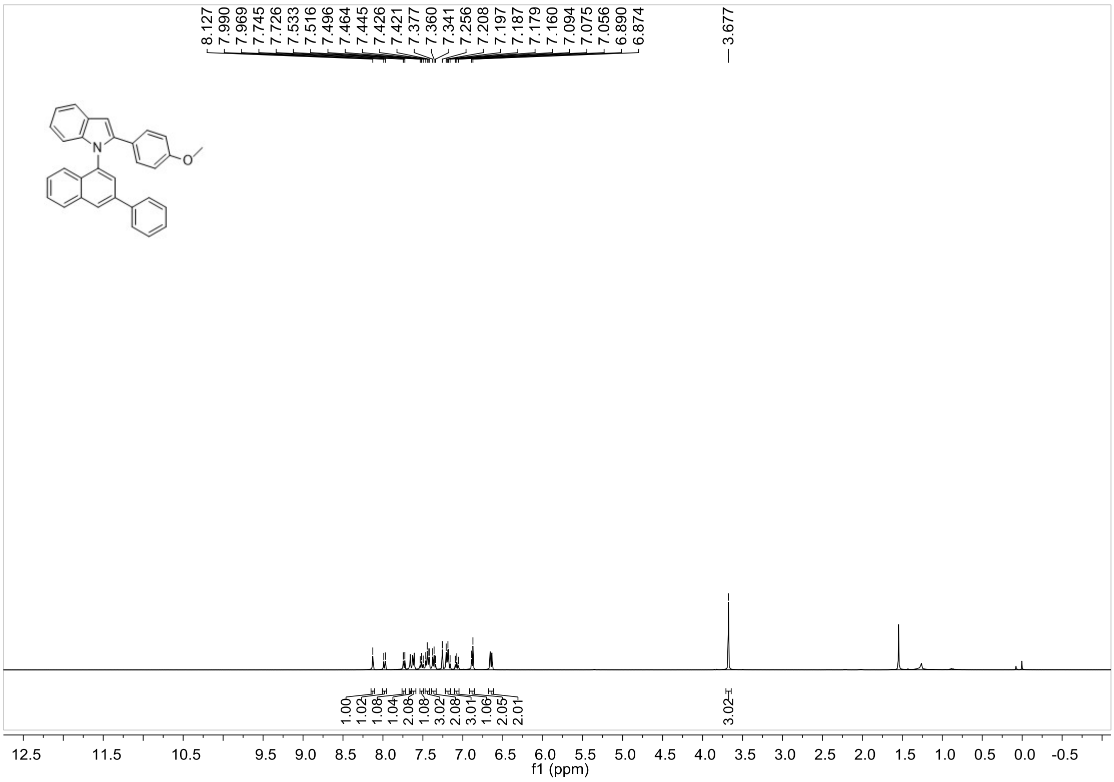

^{13}C NMR Spectrum of **13c**

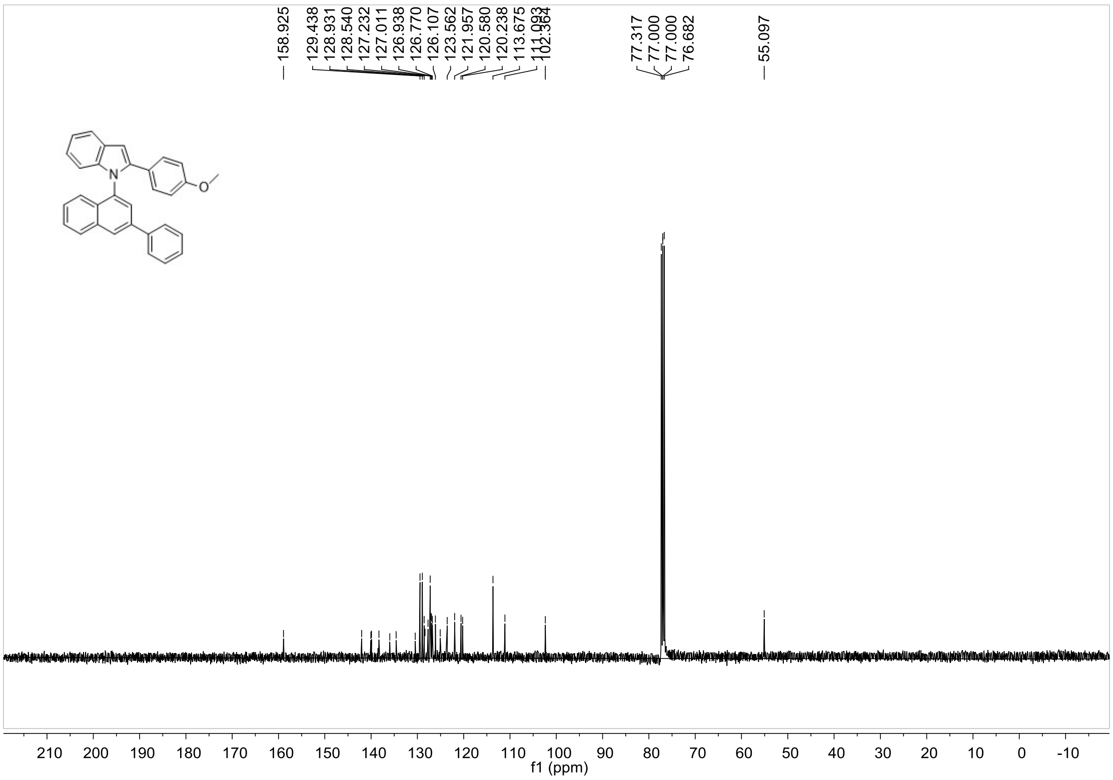

^{1}H NMR Spectrum of **14c**

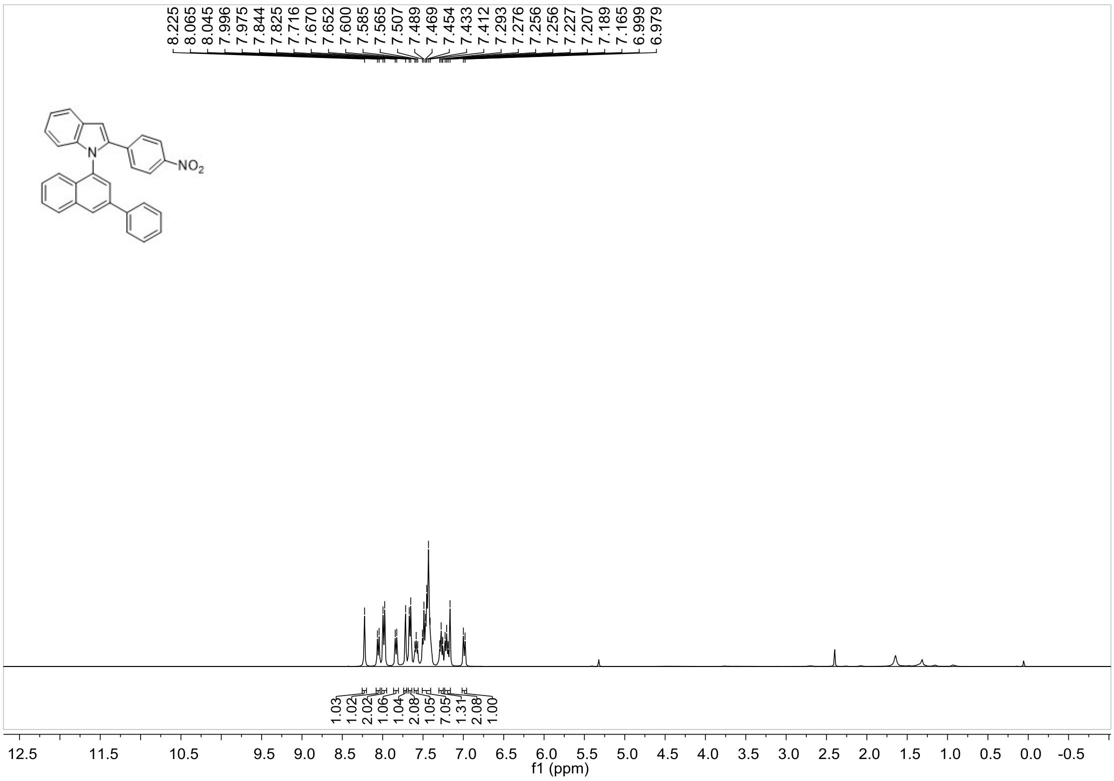

^{13}C NMR Spectrum of **14c**

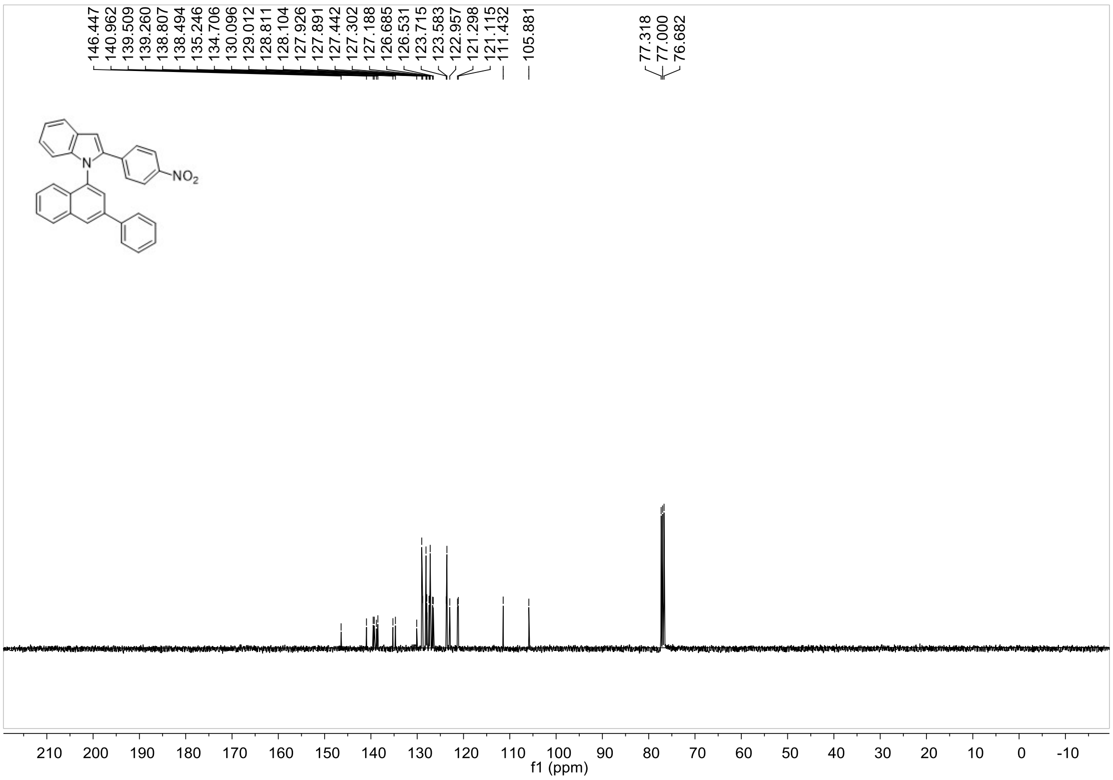

^{1}H NMR Spectrum of **15c**

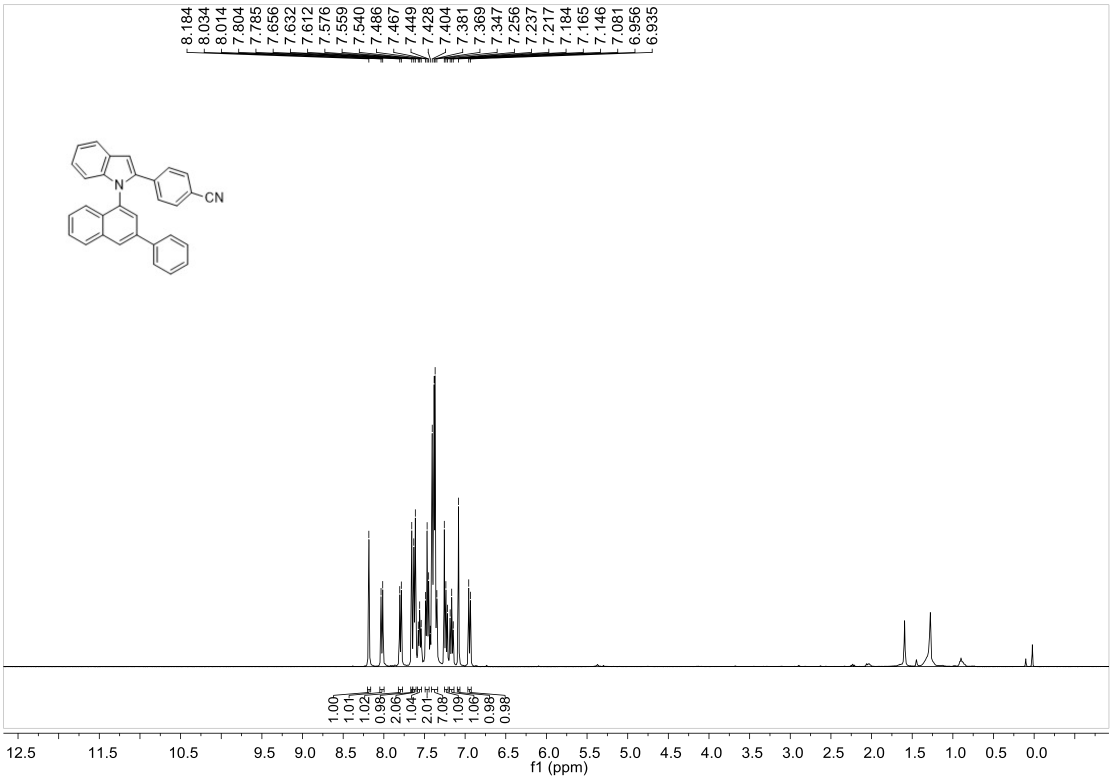

^{13}C NMR Spectrum of **15c**

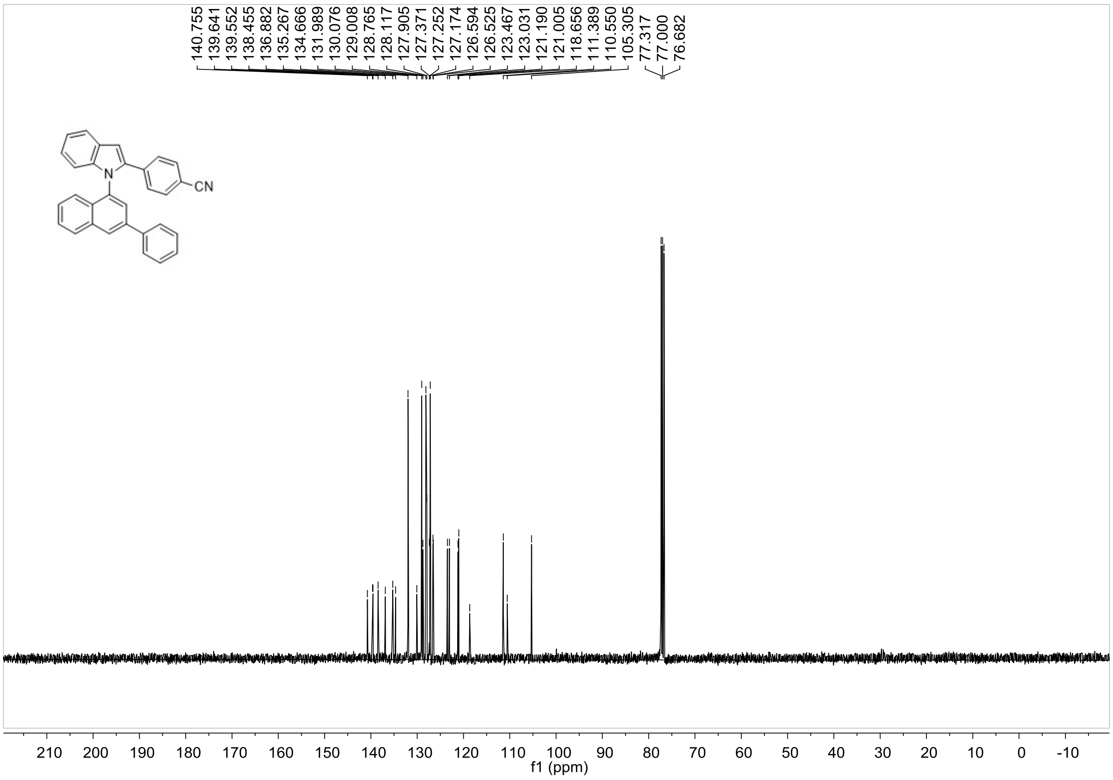

^{1}H NMR Spectrum of **16c**

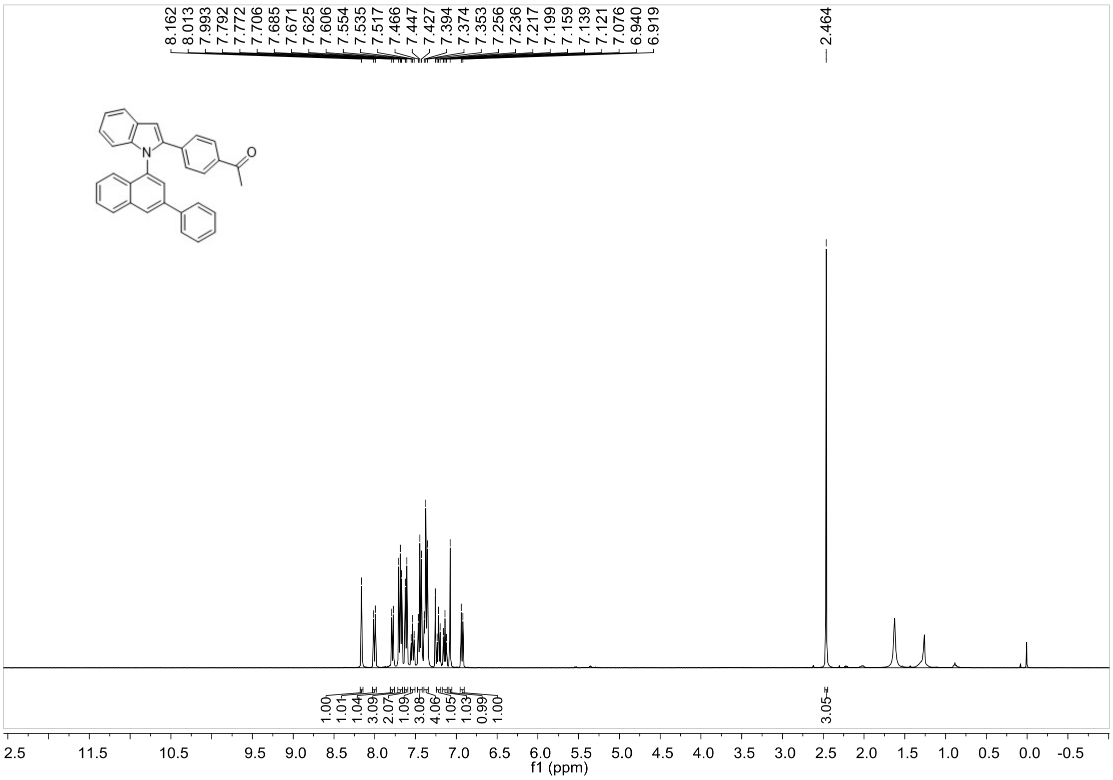

^{13}C NMR Spectrum of **16c**

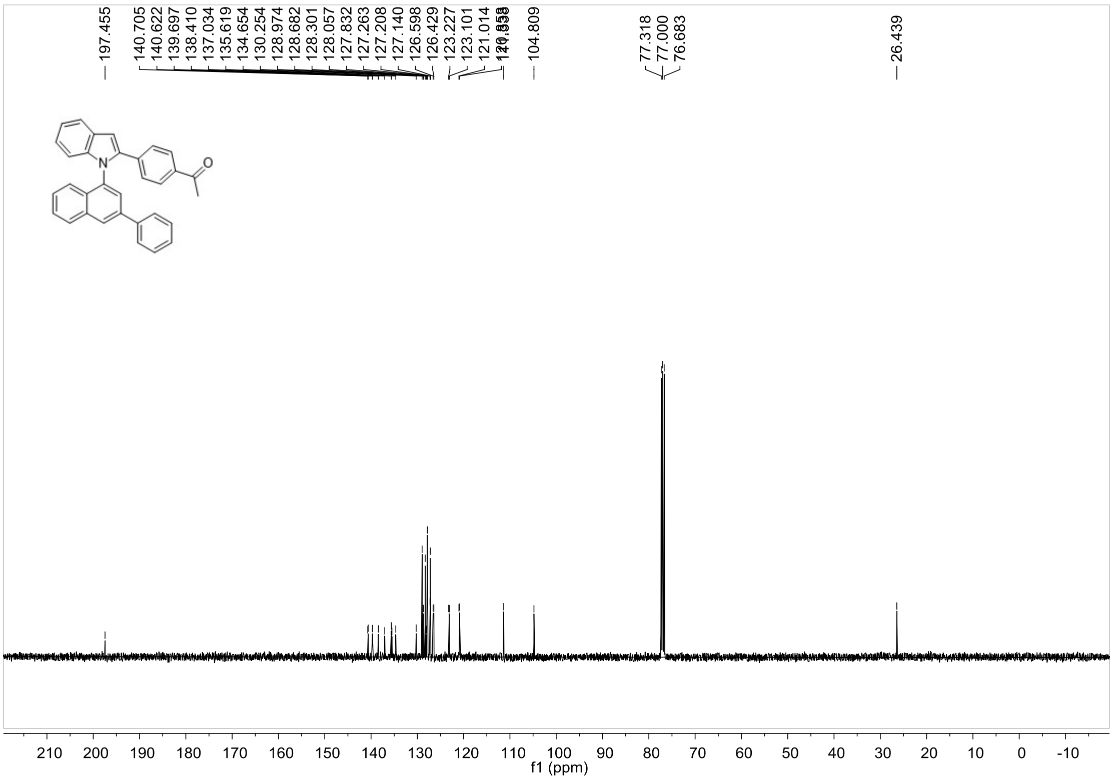

^{1}H NMR Spectrum of **17c**

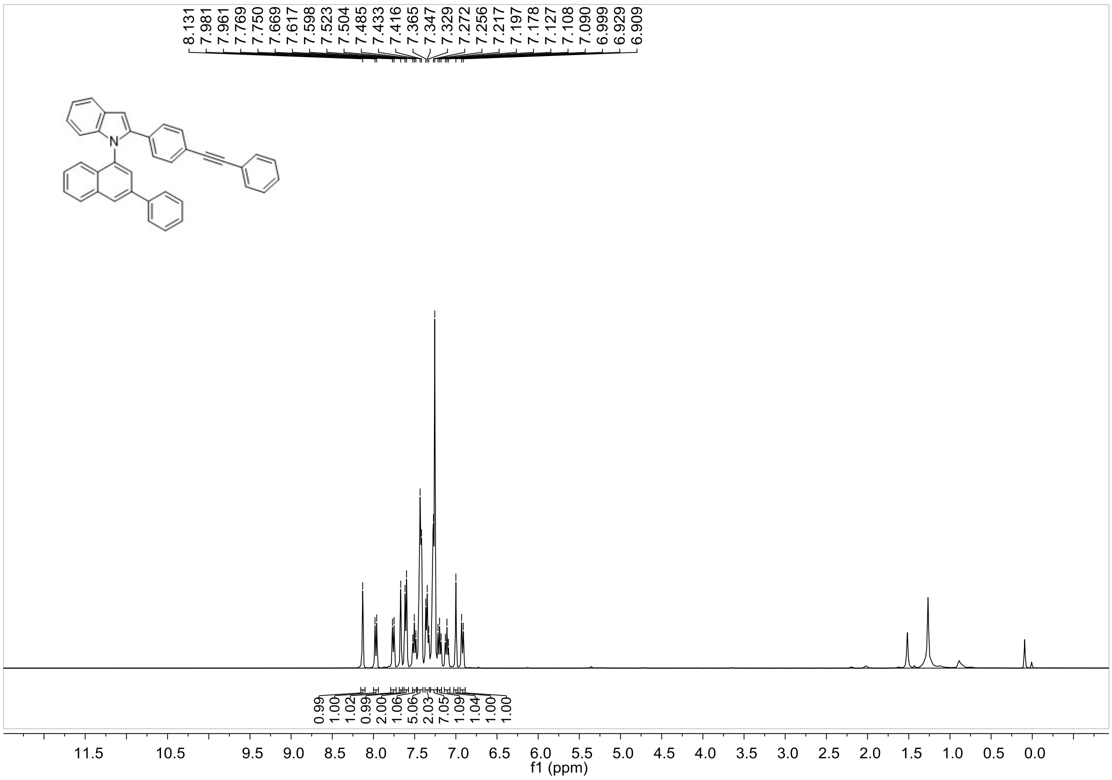

^{13}C NMR Spectrum of **17c**

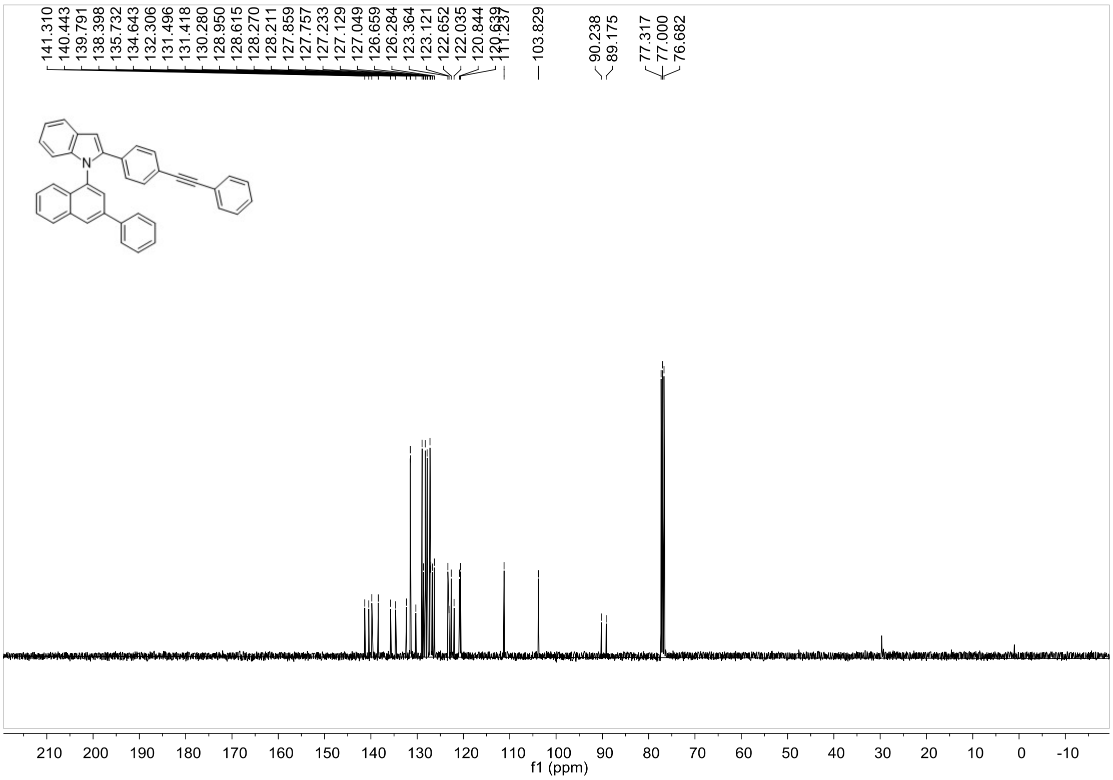

^{1}H NMR Spectrum of **18c**

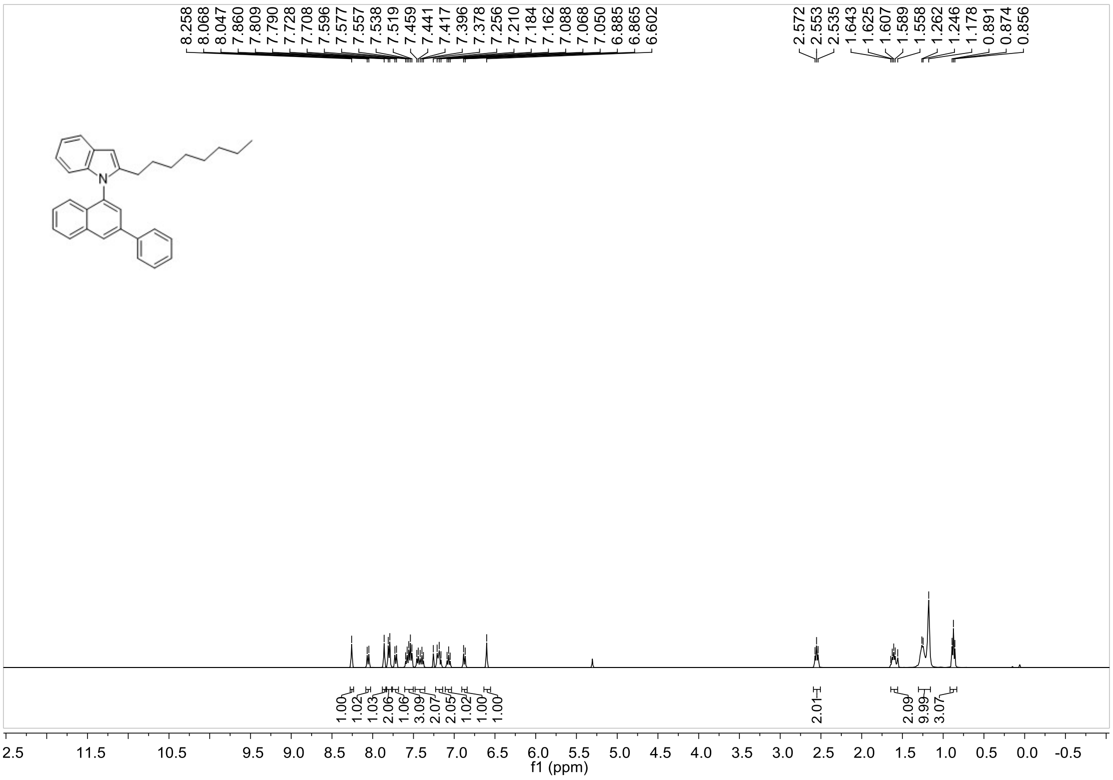

^{13}C NMR Spectrum of **18c**

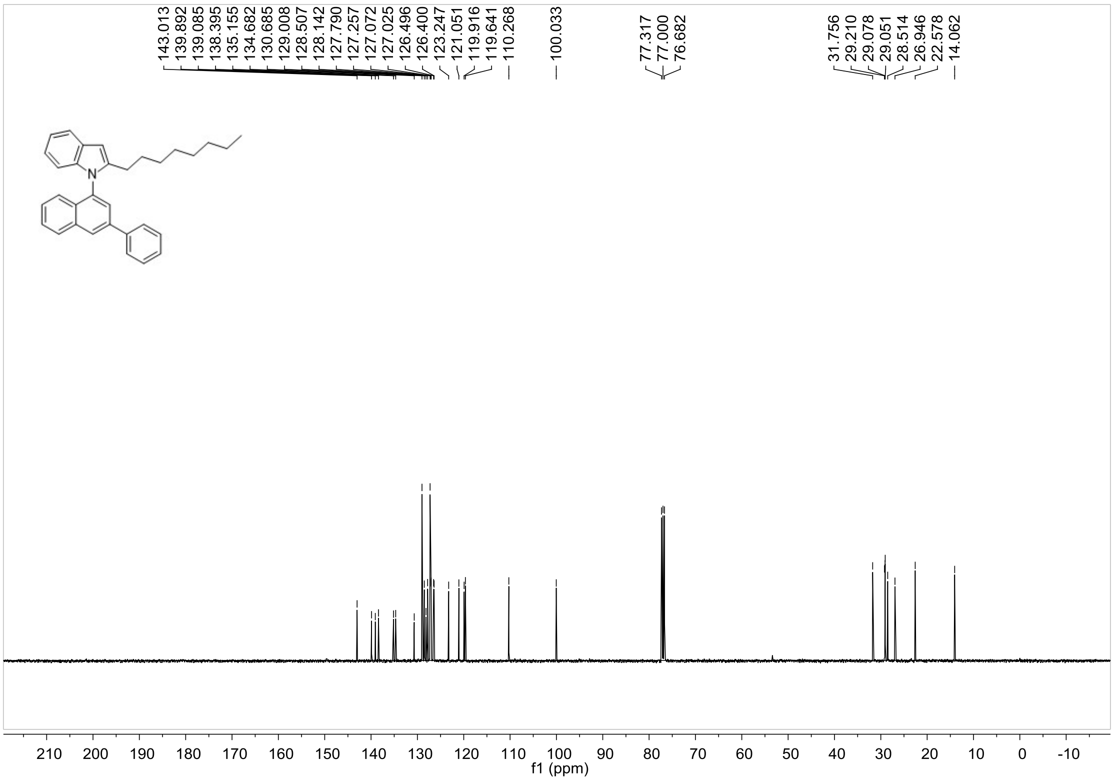

^{1}H NMR Spectrum of **19c**

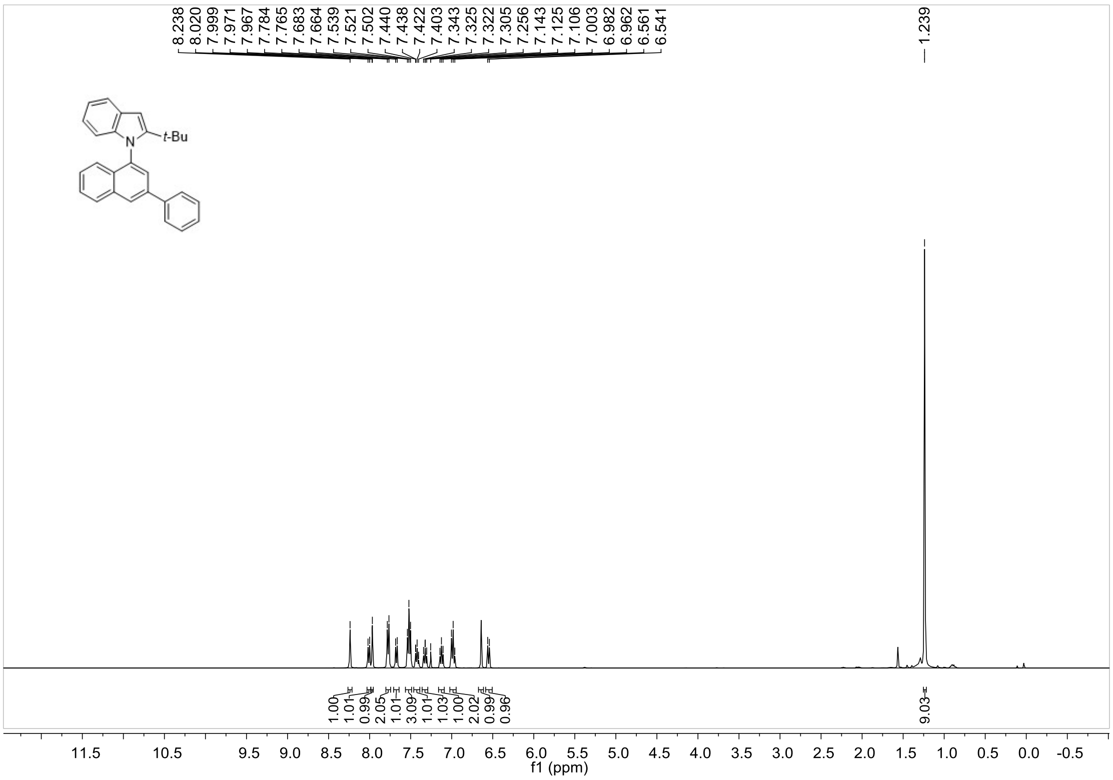

^{13}C NMR Spectrum of **19c**

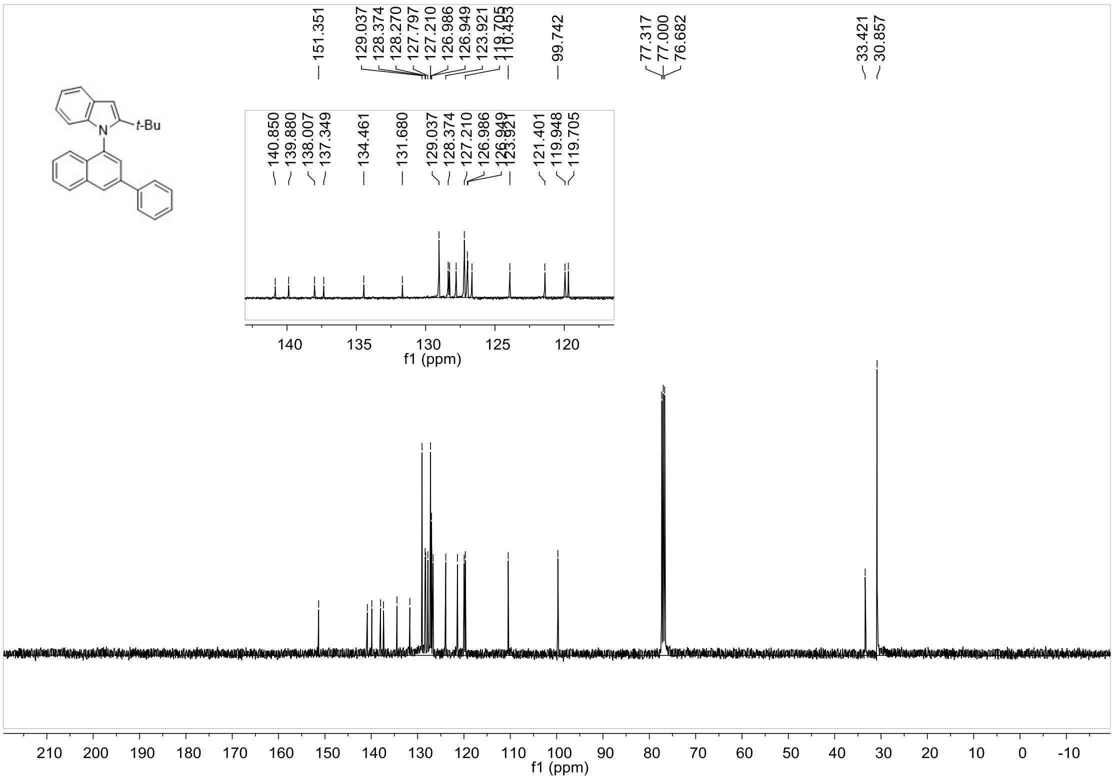

^{1}H NMR Spectrum of **20c**

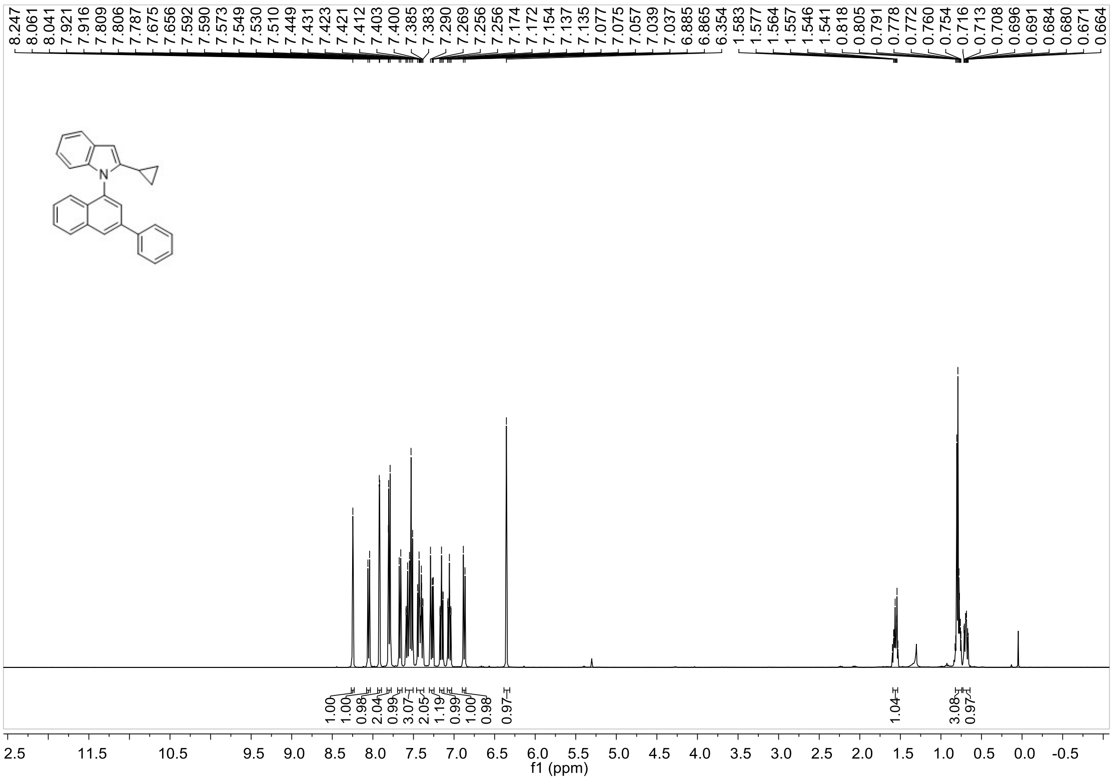

^{13}C NMR Spectrum of **20c**

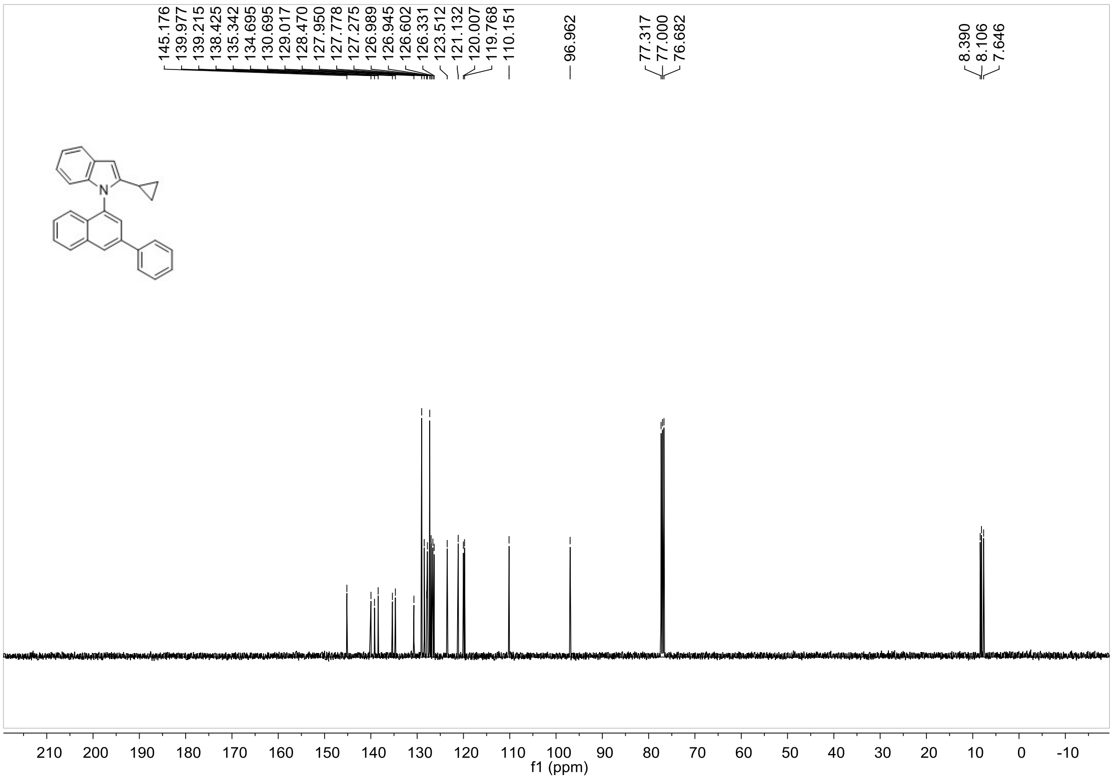

^{1}H NMR Spectrum of **21c**

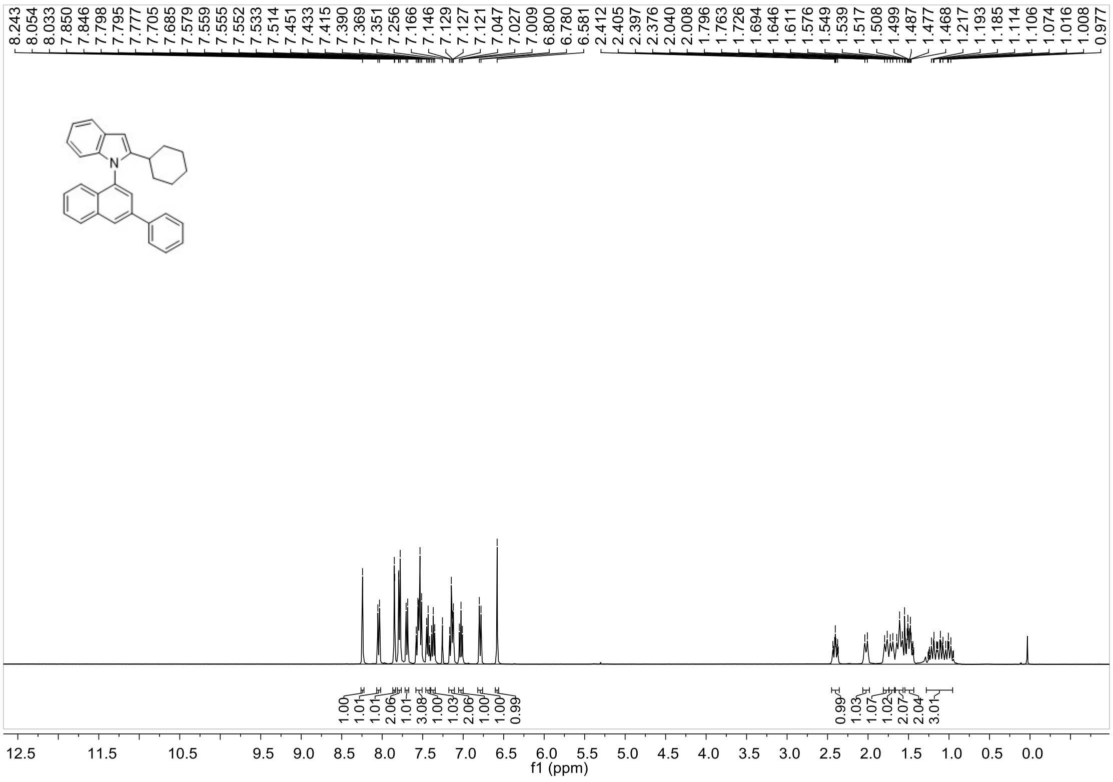

^{13}C NMR Spectrum of **21c**

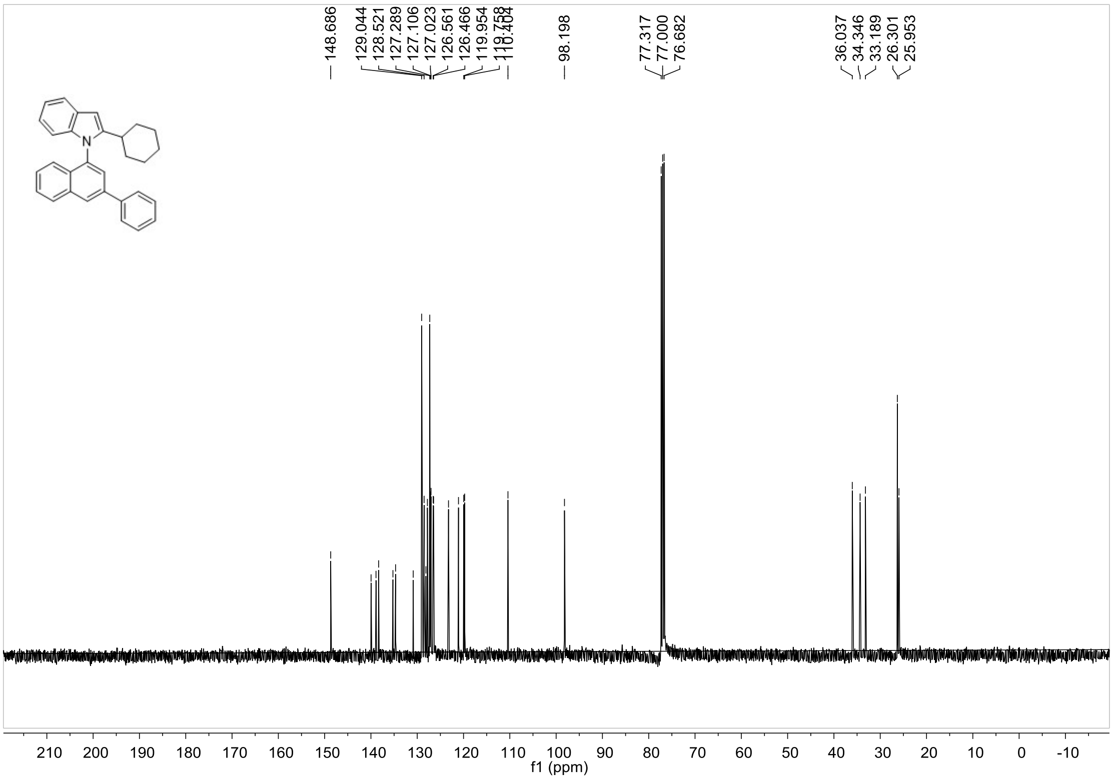

^{1}H NMR Spectrum of **22c**

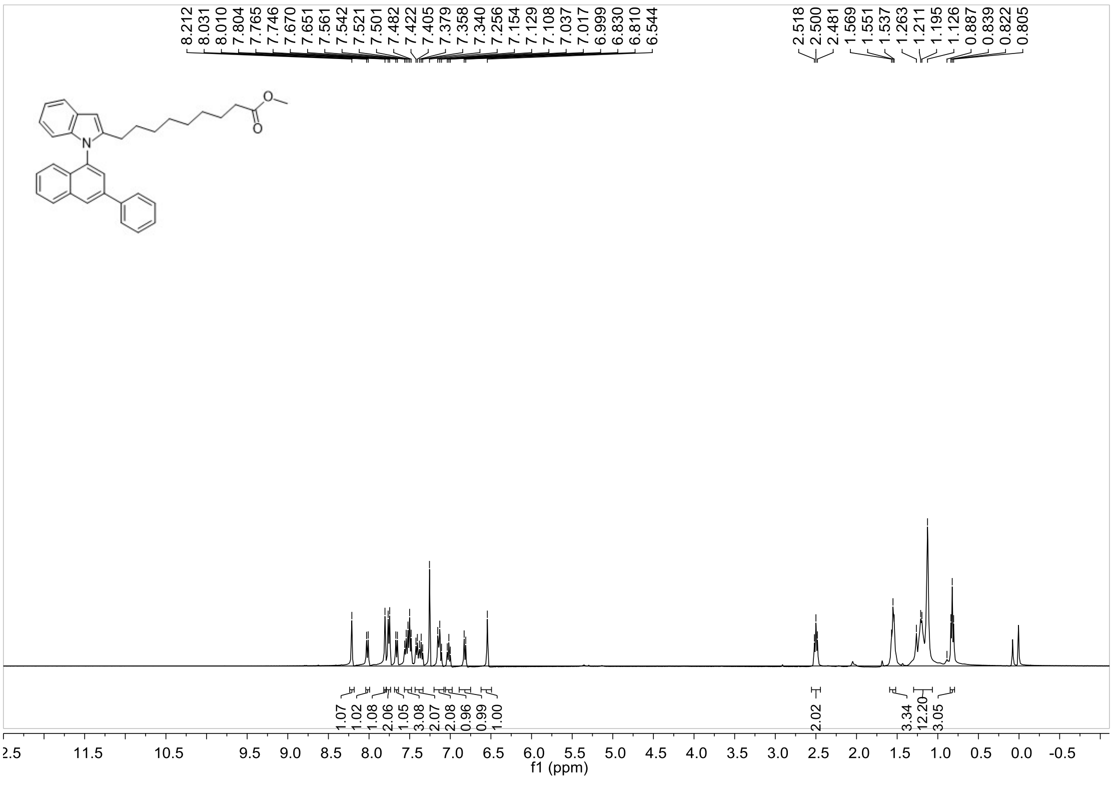

^{13}C NMR Spectrum of **22c**

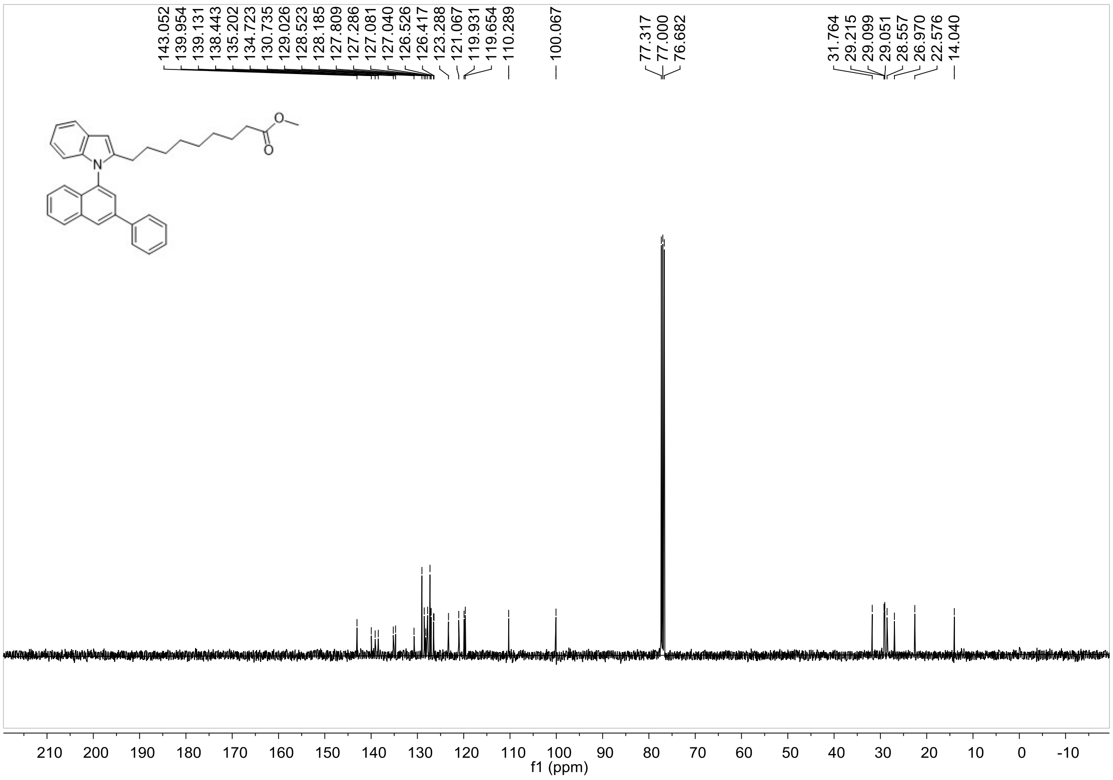

^{1}H NMR Spectrum of **23c**

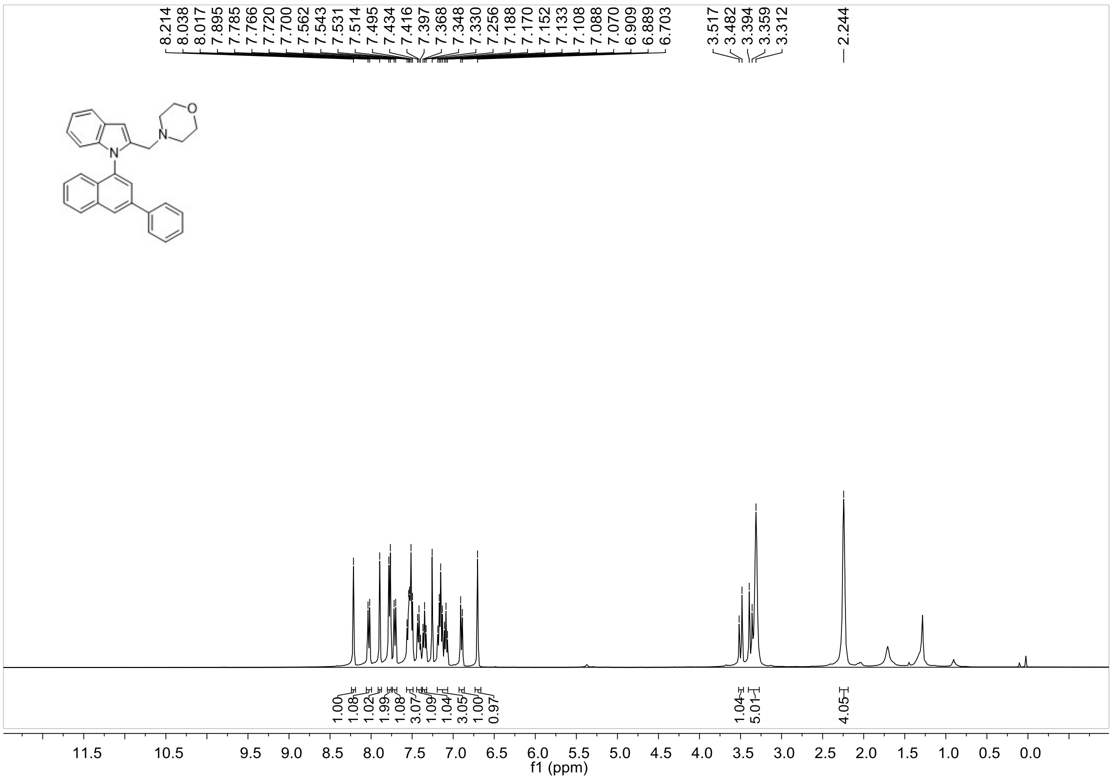

^{13}C NMR Spectrum of **23c**

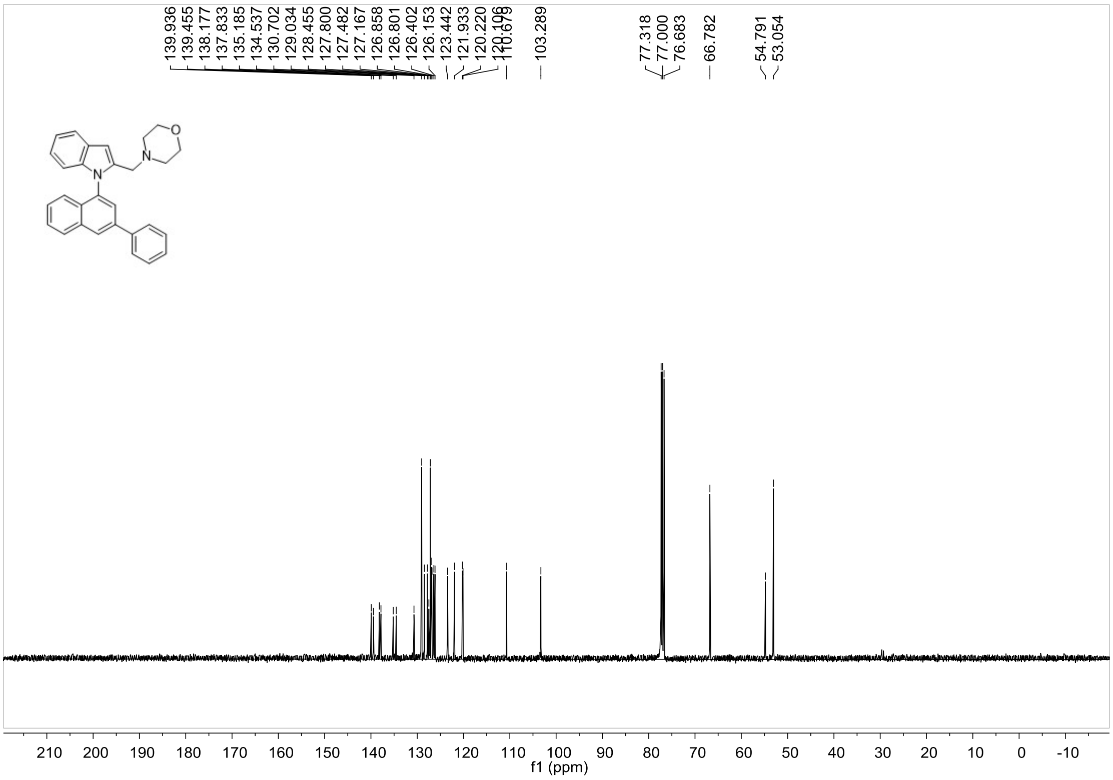

^{1}H NMR Spectrum of **24c**

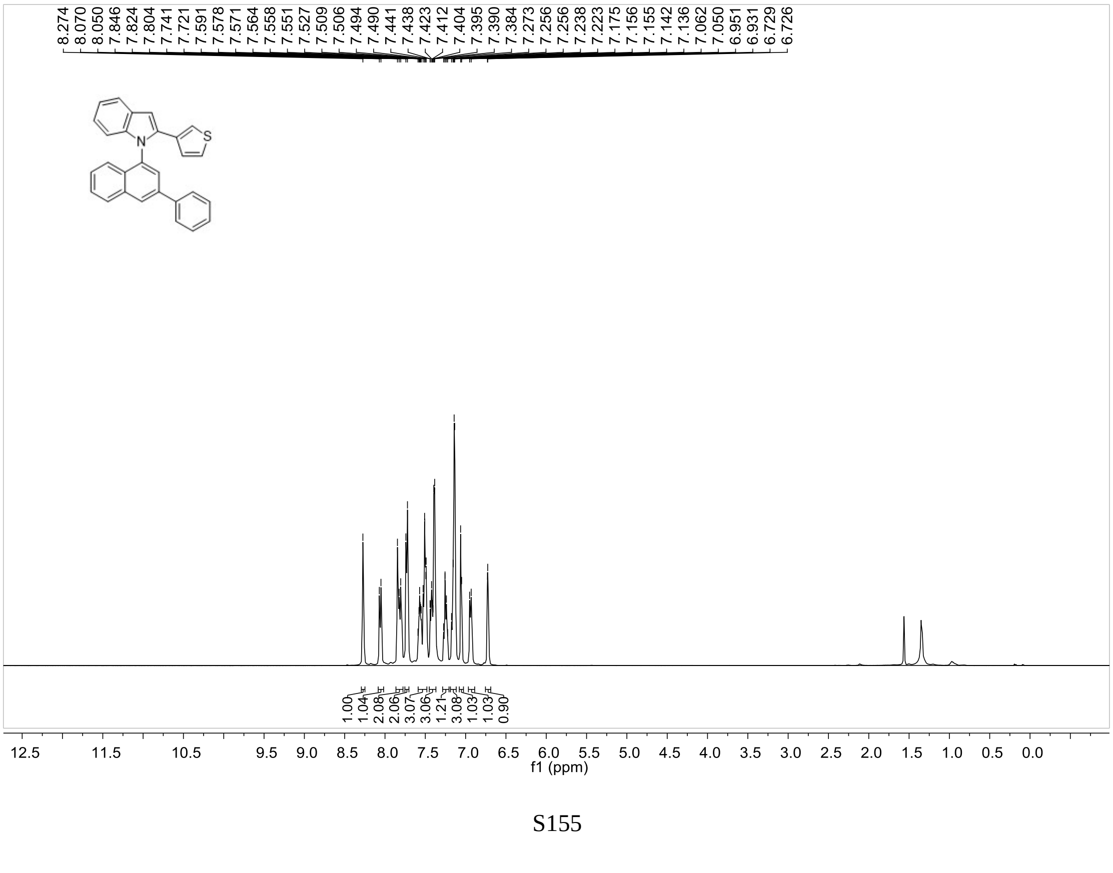

^{13}C NMR Spectrum of **24c**

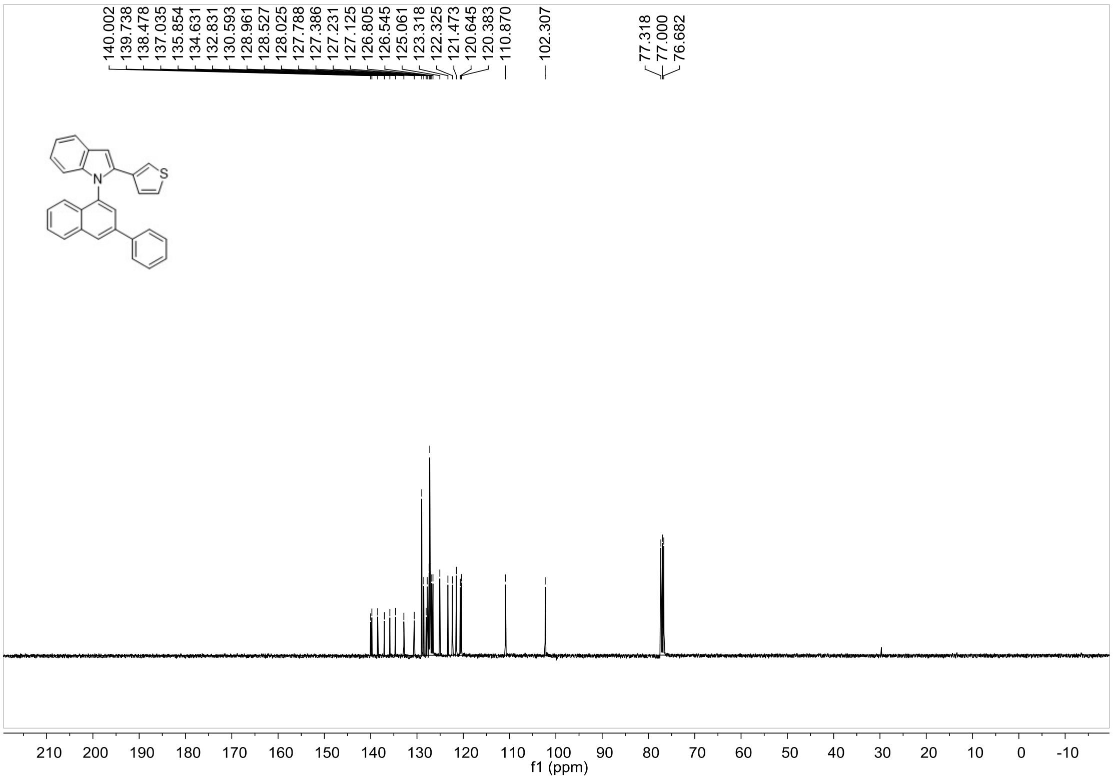

^{1}H NMR Spectrum of **25c**

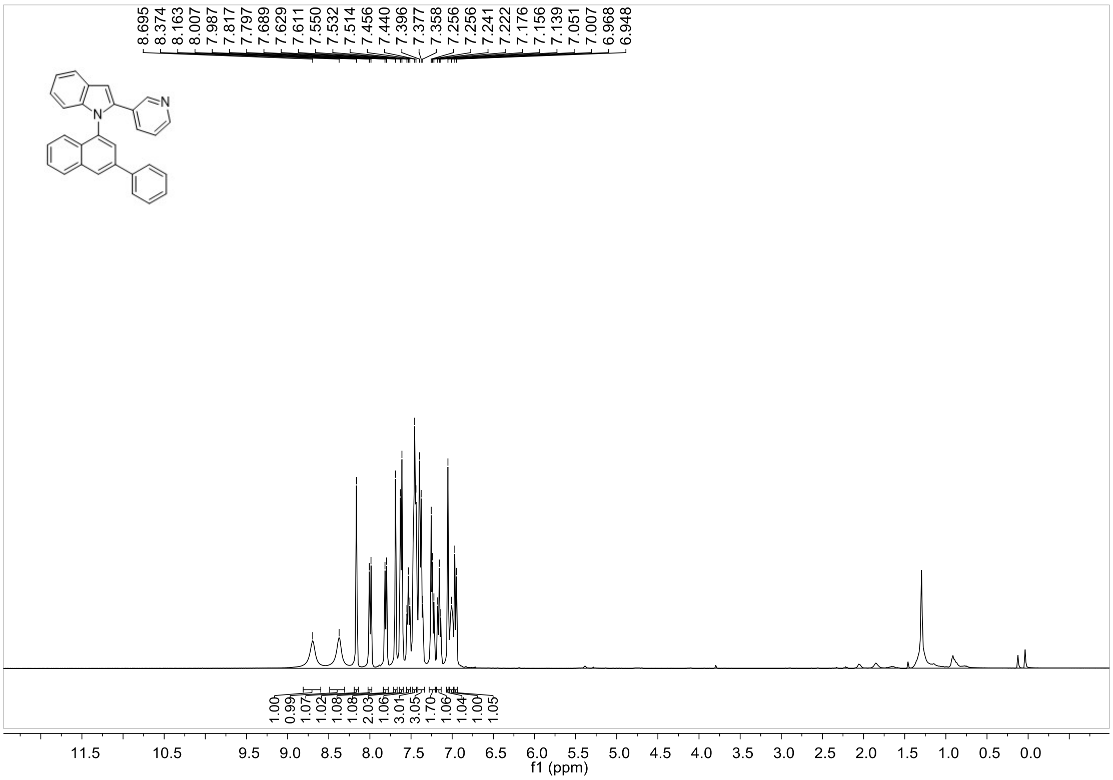

^{13}C NMR Spectrum of **25c**

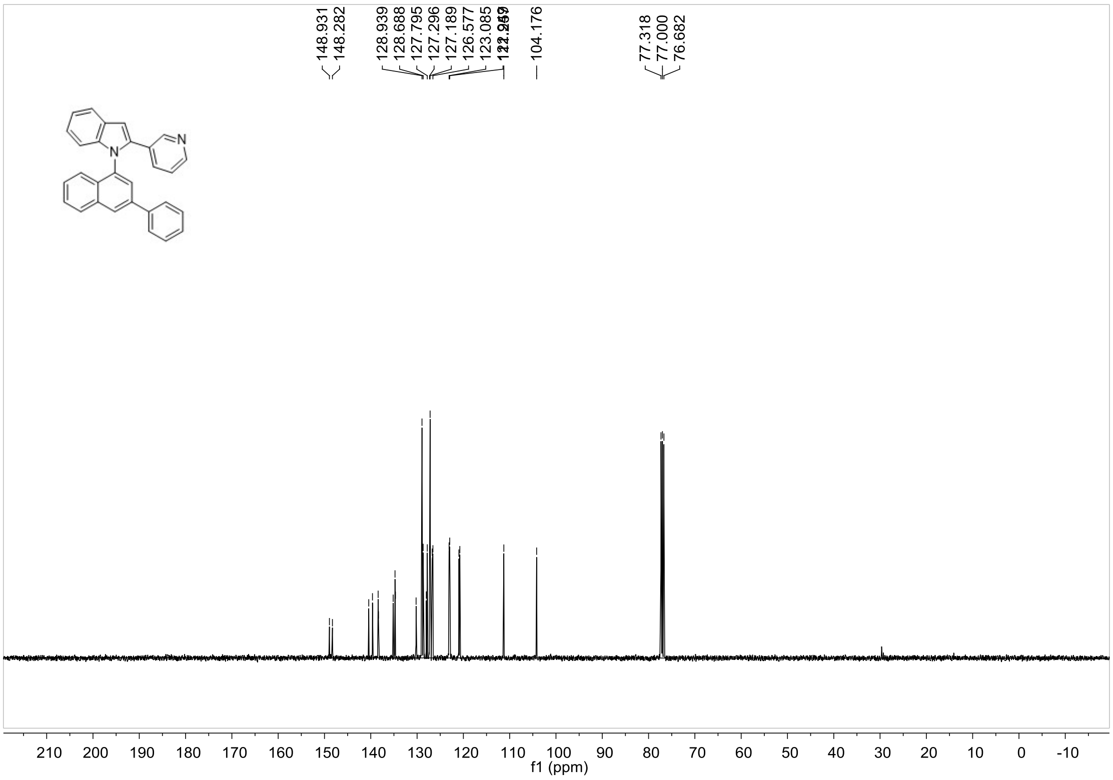

^{1}H NMR Spectrum of **26c**

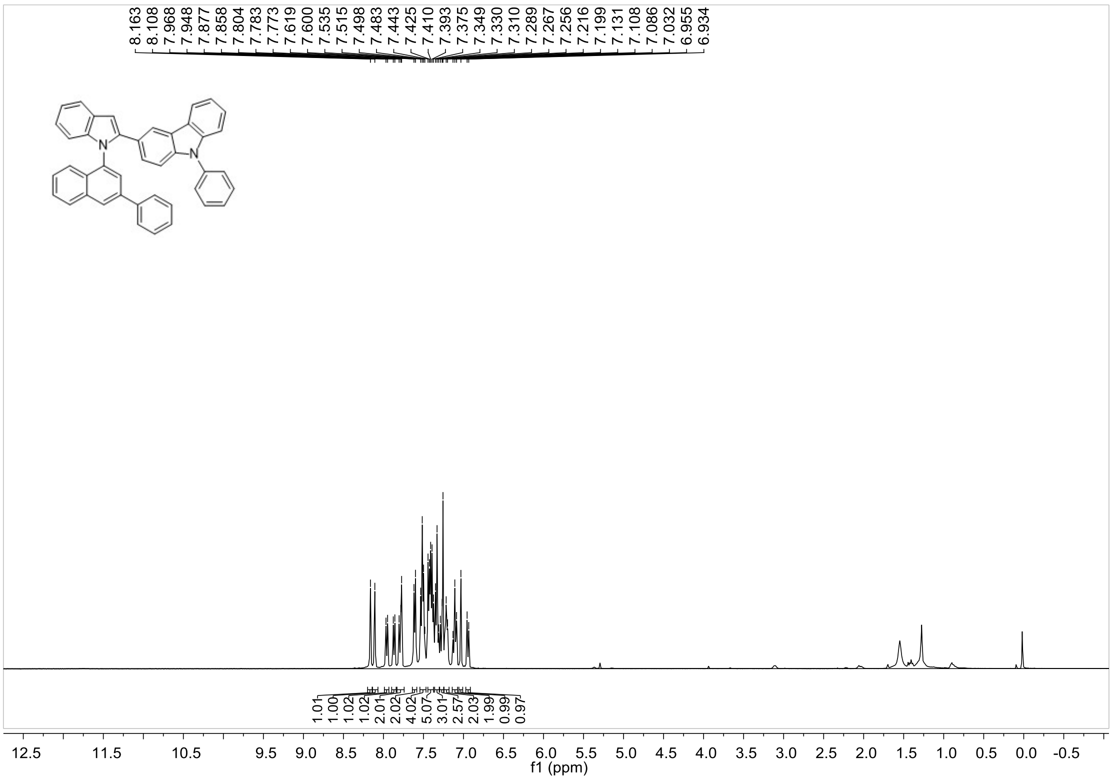

^{13}C NMR Spectrum of **26c**

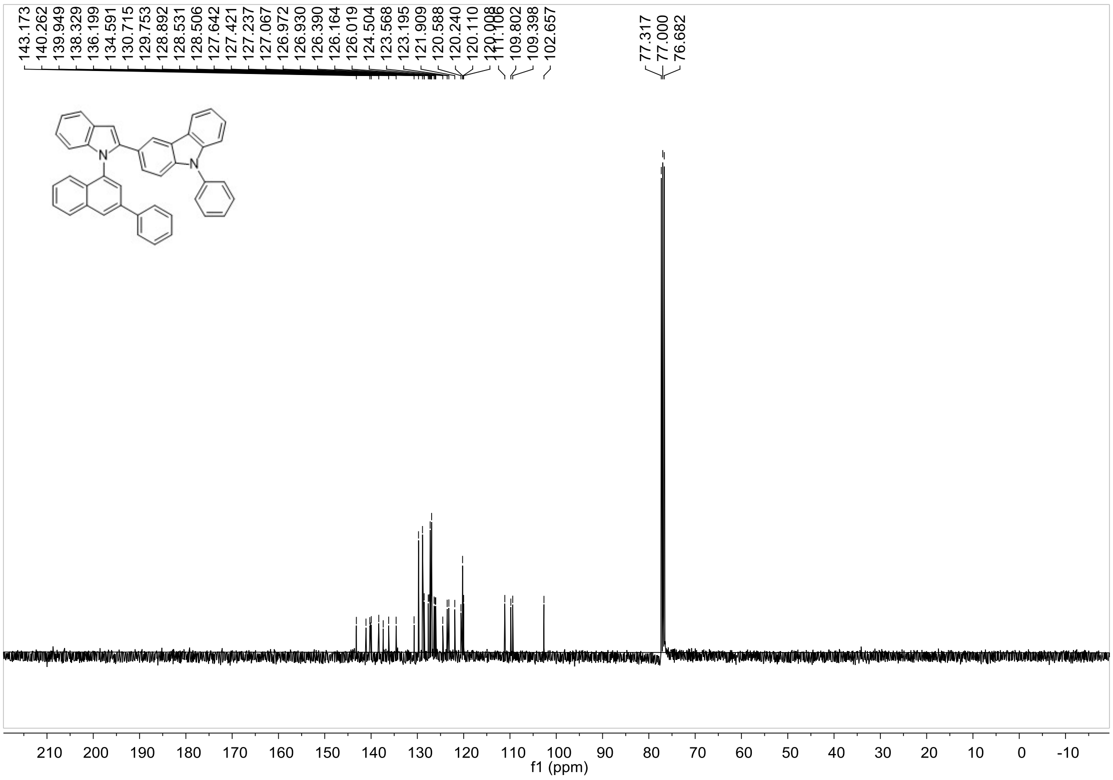

^{1}H NMR Spectrum of **27c**

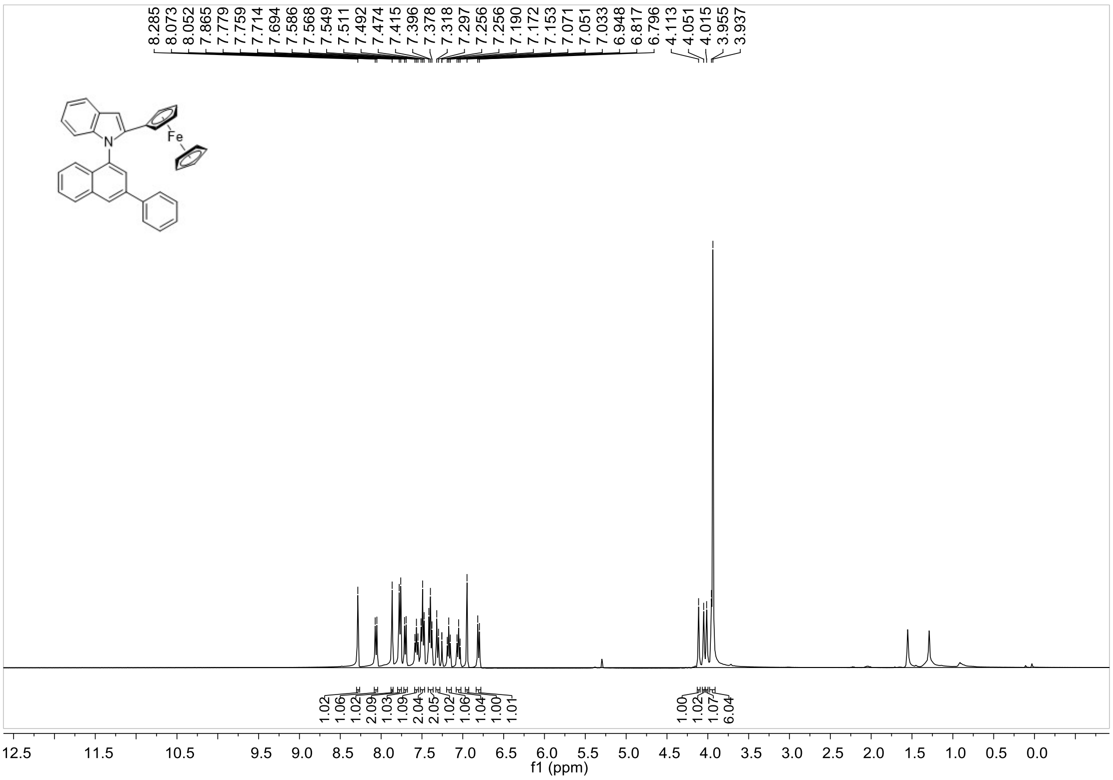

^{13}C NMR Spectrum of **27c**

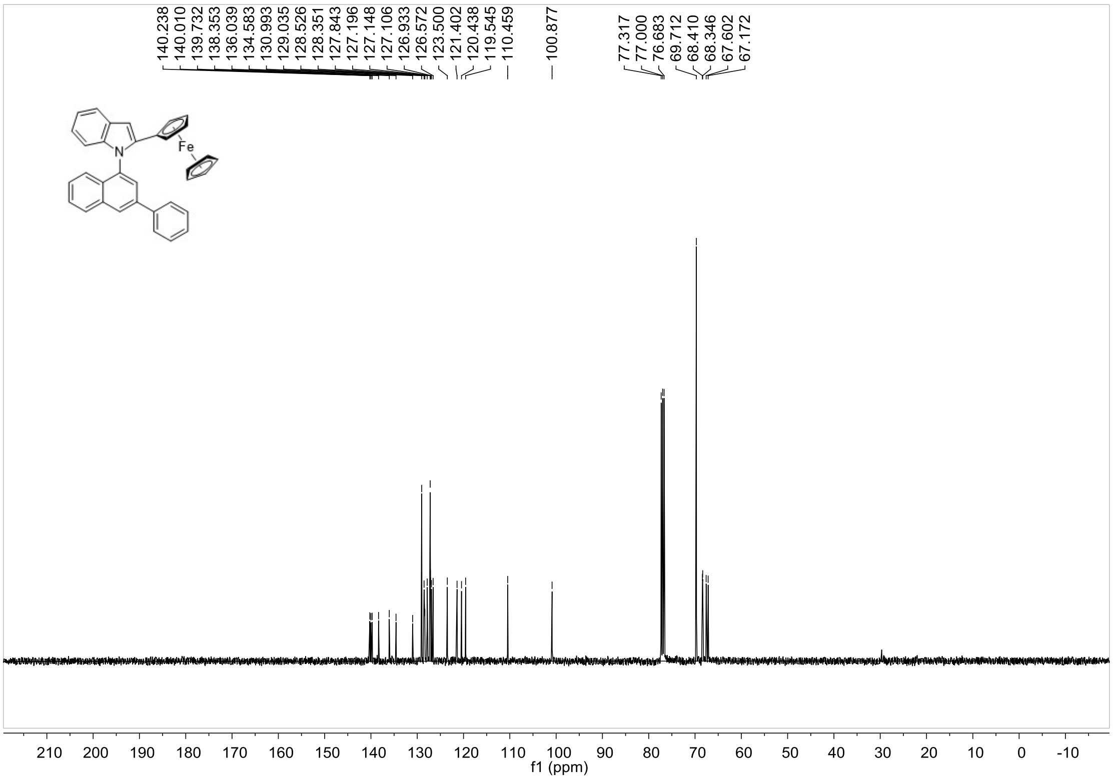

^{1}H NMR Spectrum of **28c**

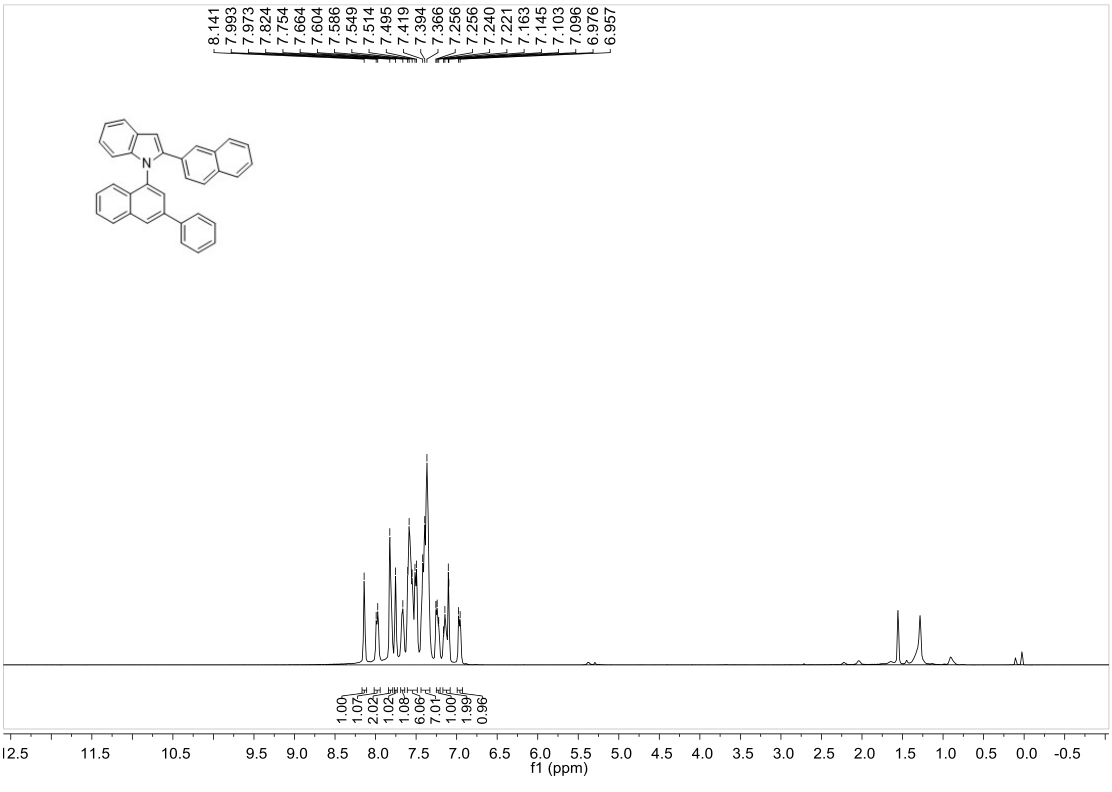

^{13}C NMR Spectrum of **28c**

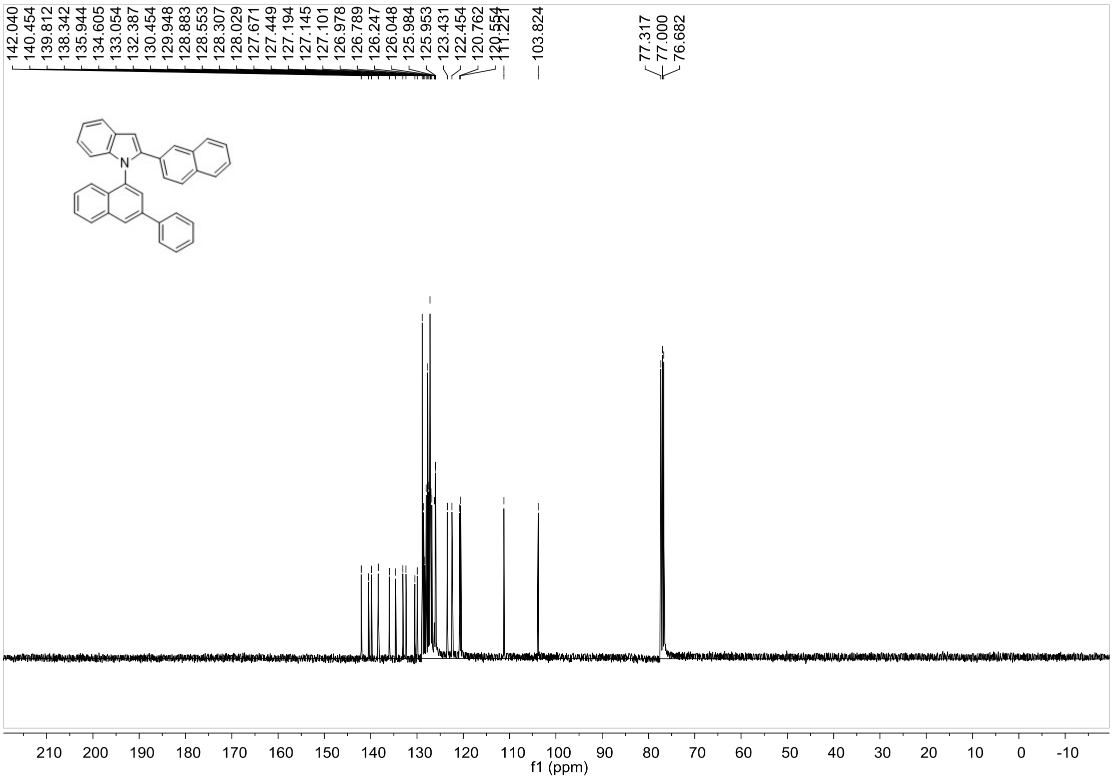

^{1}H NMR Spectrum of **29c**

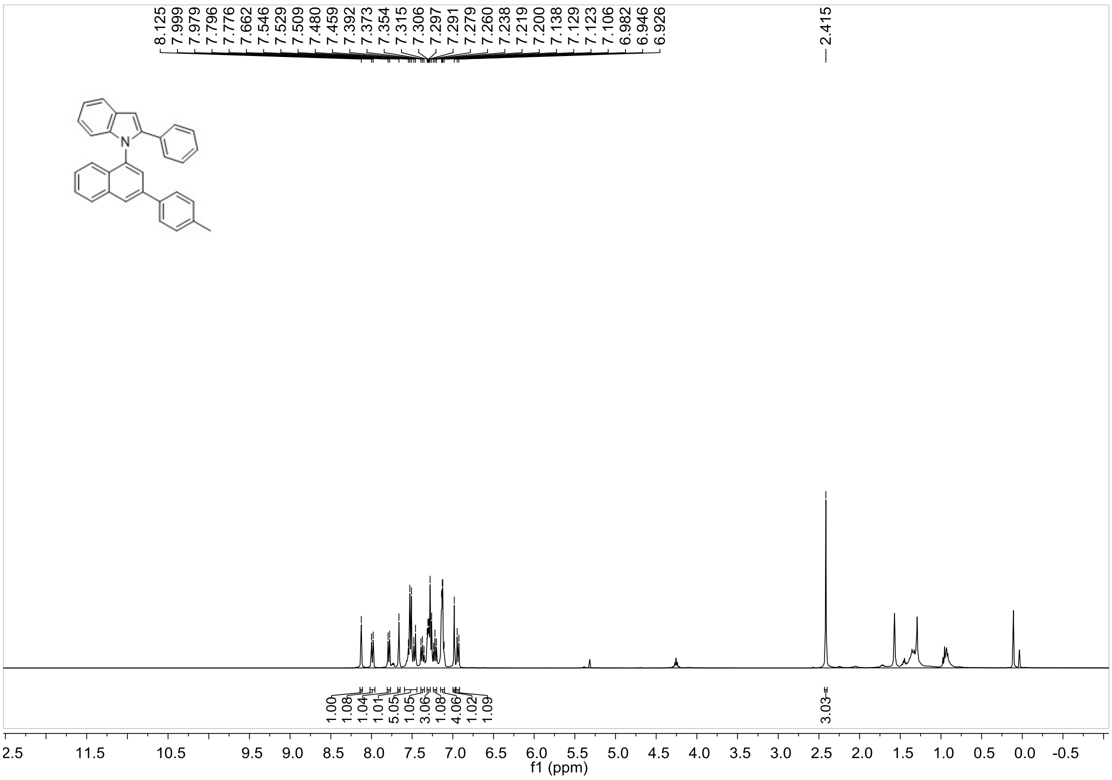

^{13}C NMR Spectrum of **29c**

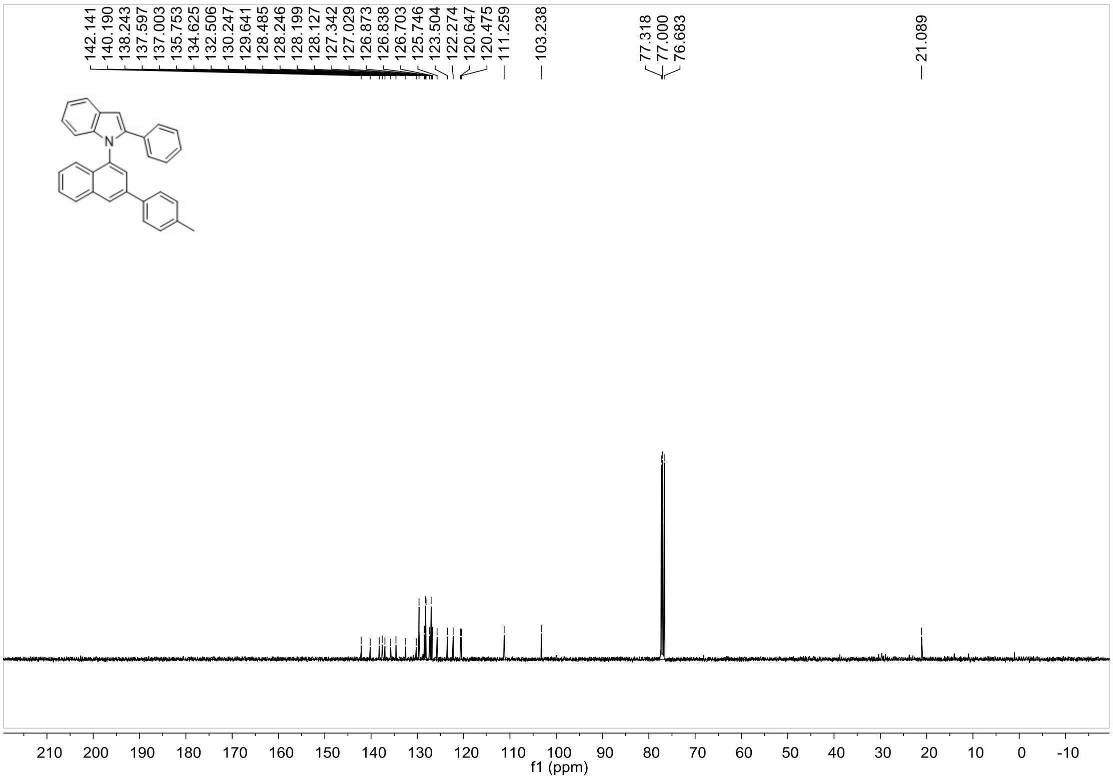

^{1}H NMR Spectrum of **30c**

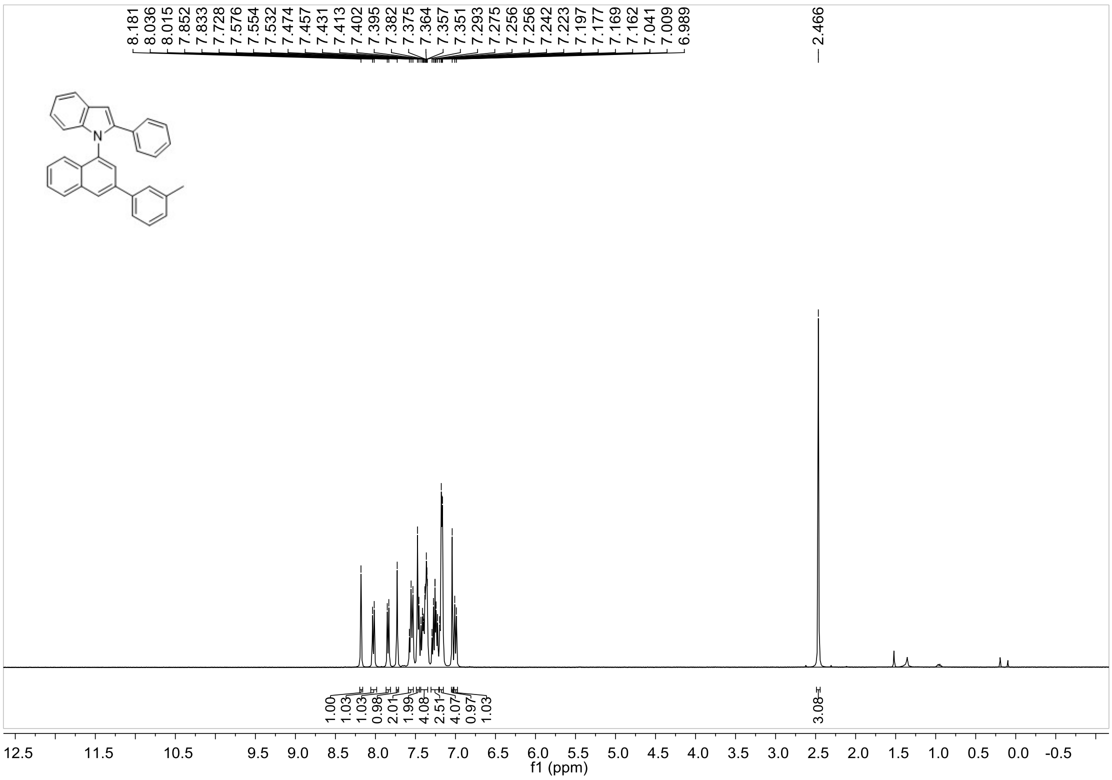

^{13}C NMR Spectrum of **30c**

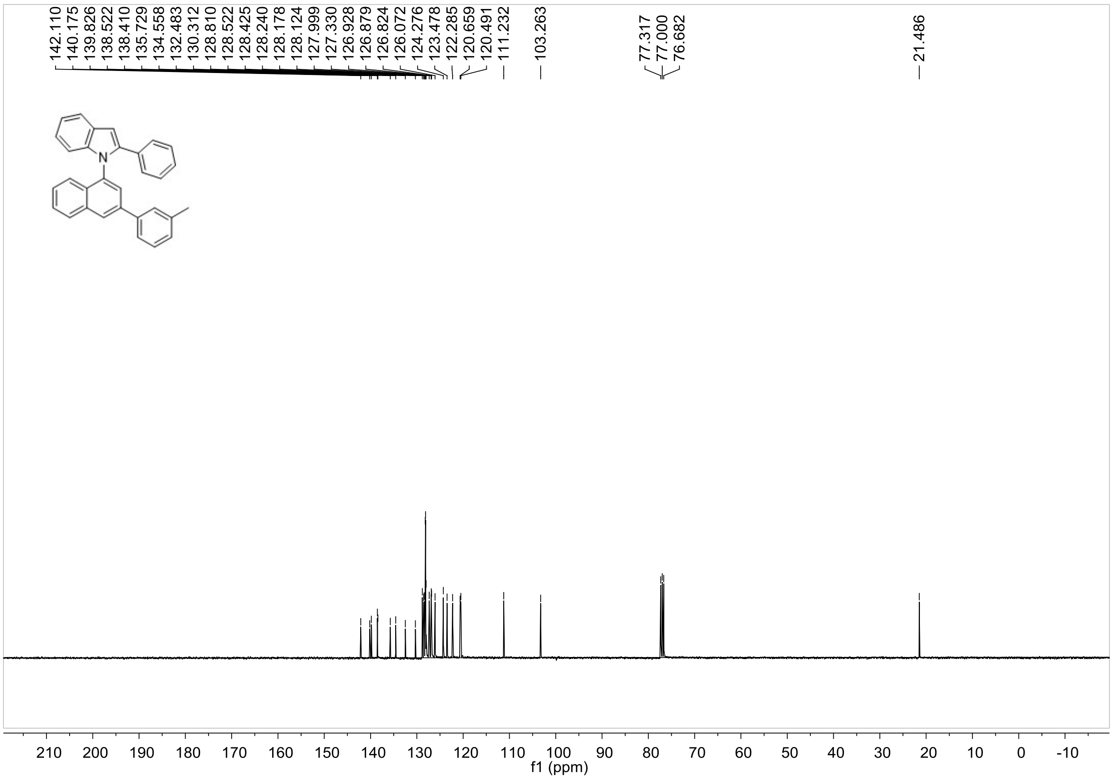

^{1}H NMR Spectrum of **31c**

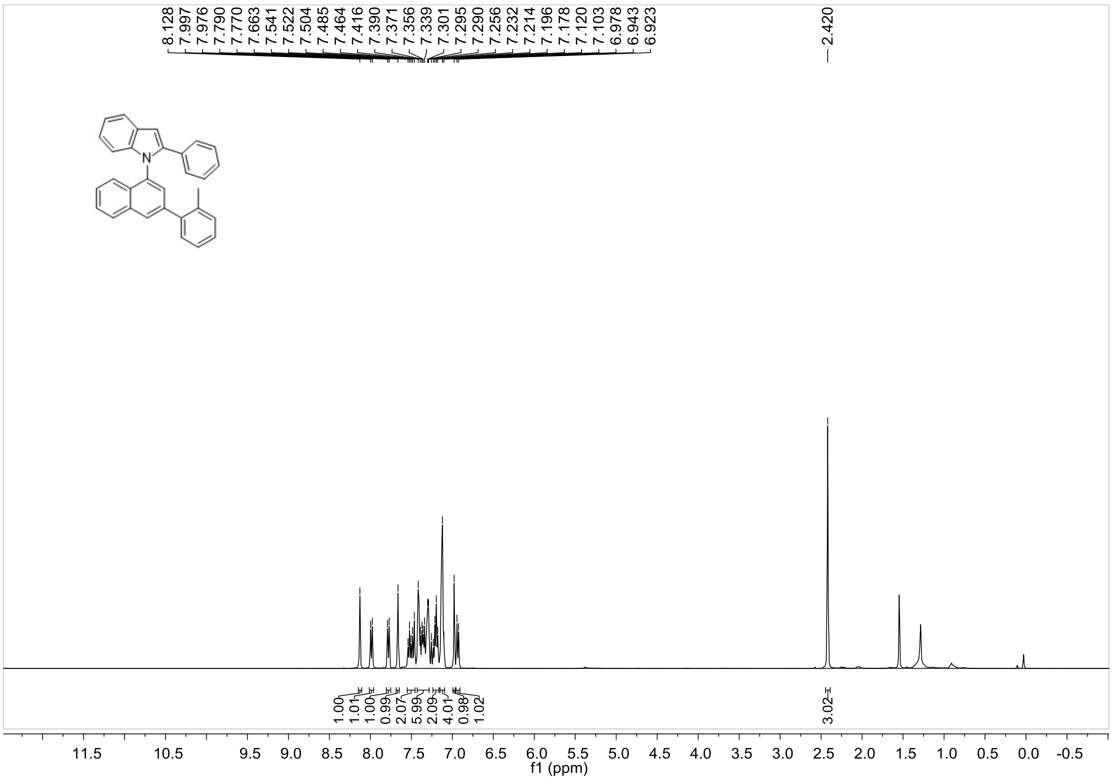

^{13}C NMR Spectrum of **31c**

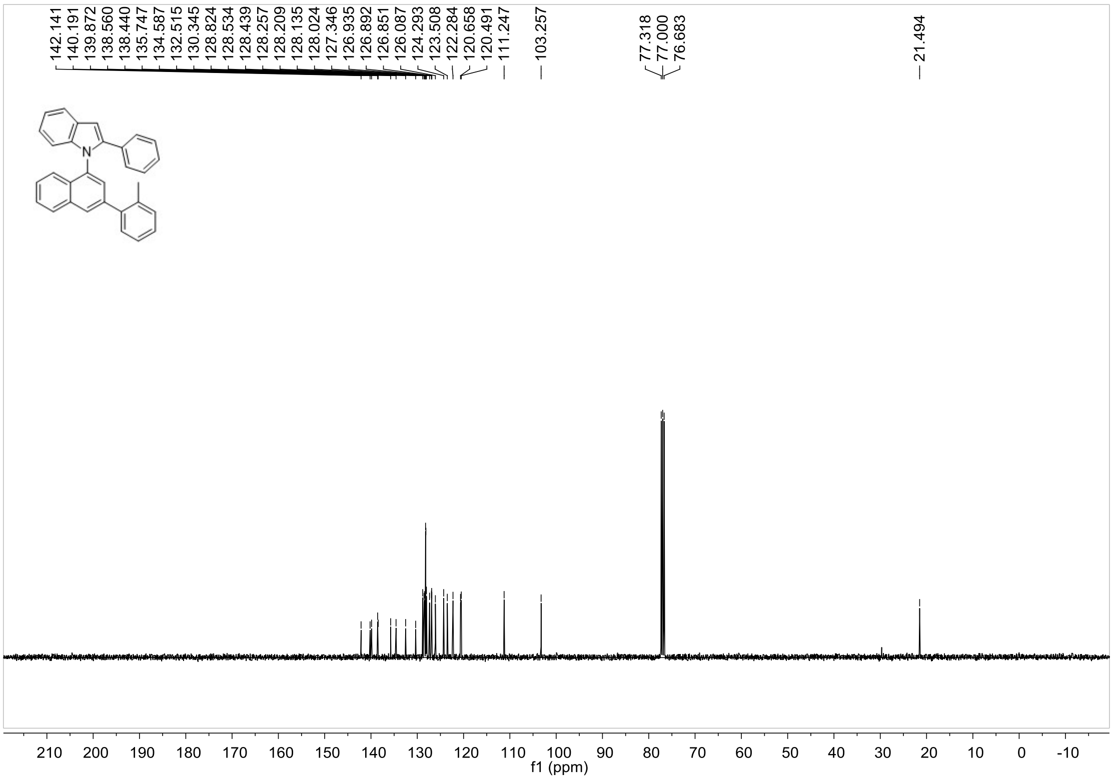

^{1}H NMR Spectrum of **32c**

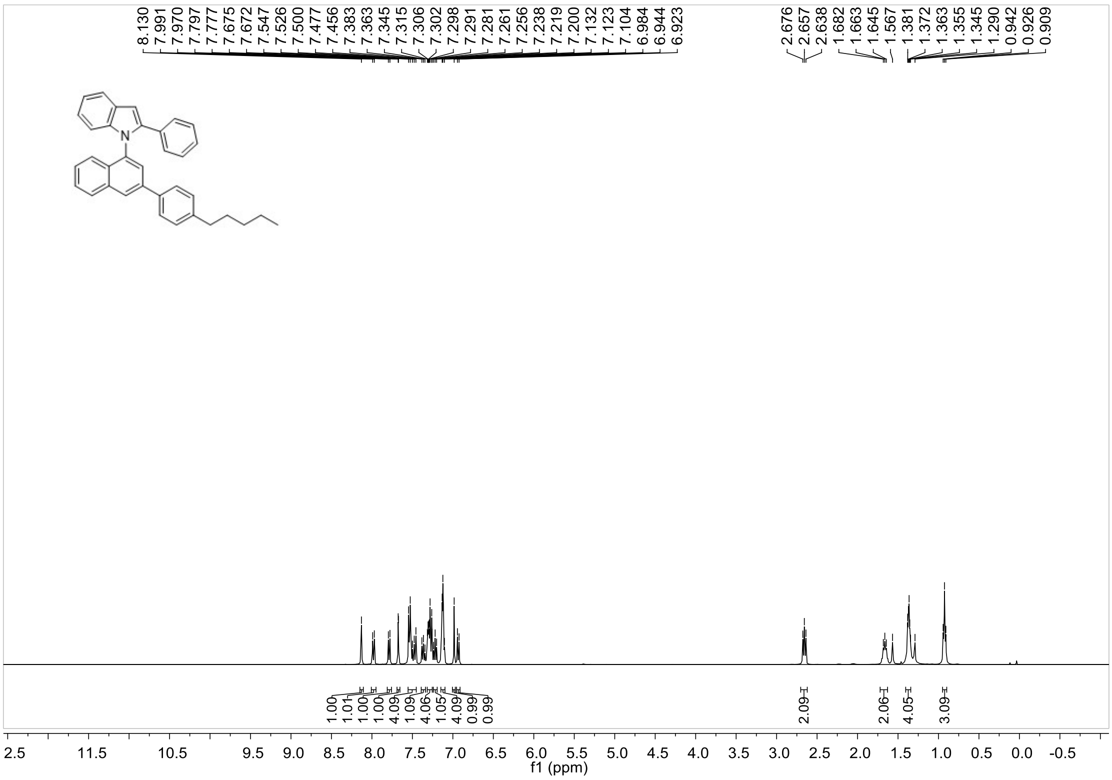

^{13}C NMR Spectrum of **32c**

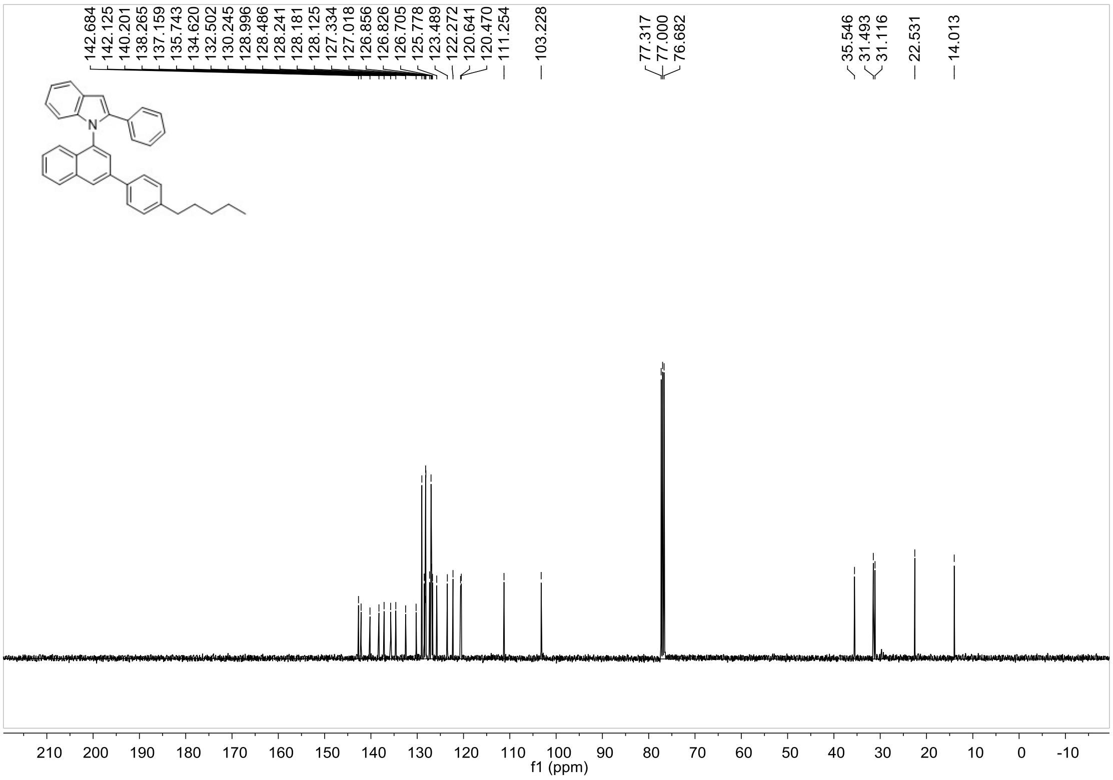

^{1}H NMR Spectrum of **33c**

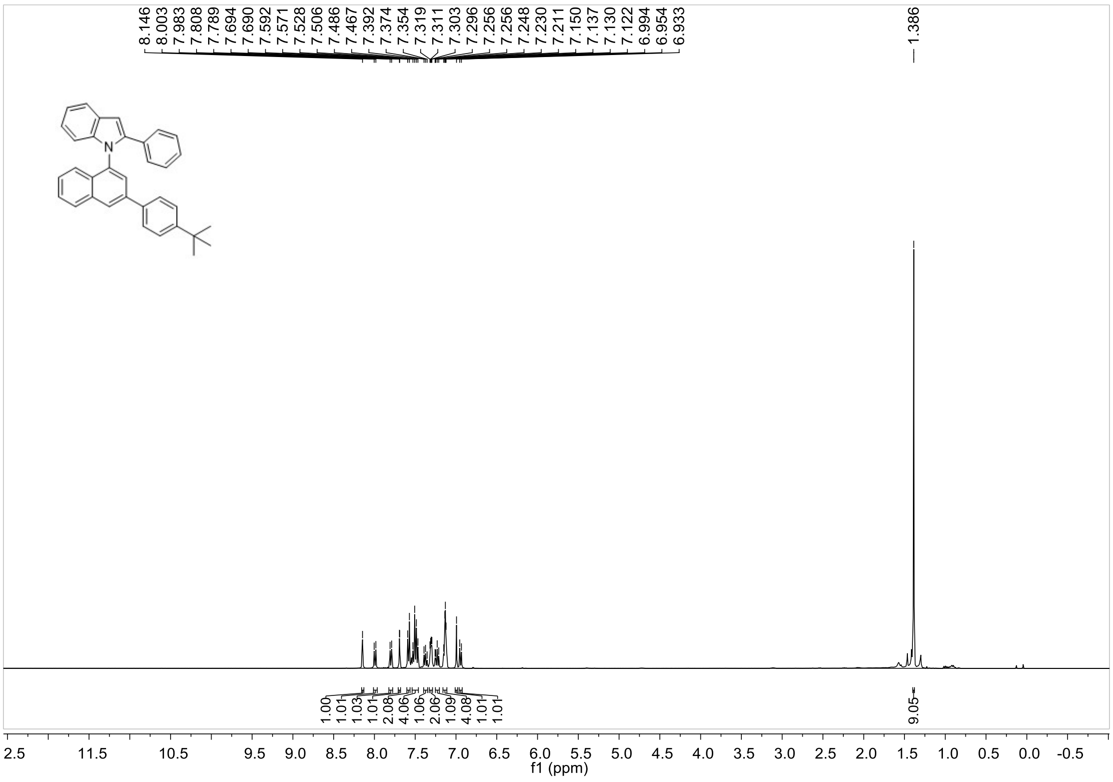

^{13}C NMR Spectrum of **33c**

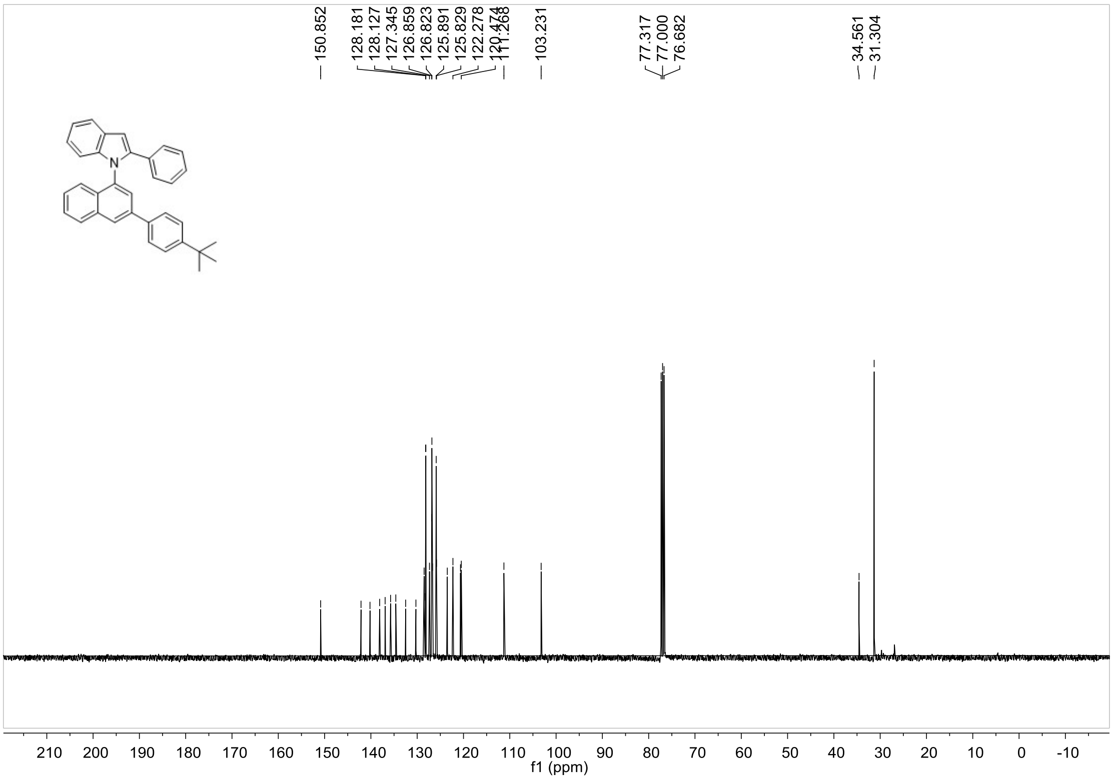

^{1}H NMR Spectrum of **34c**

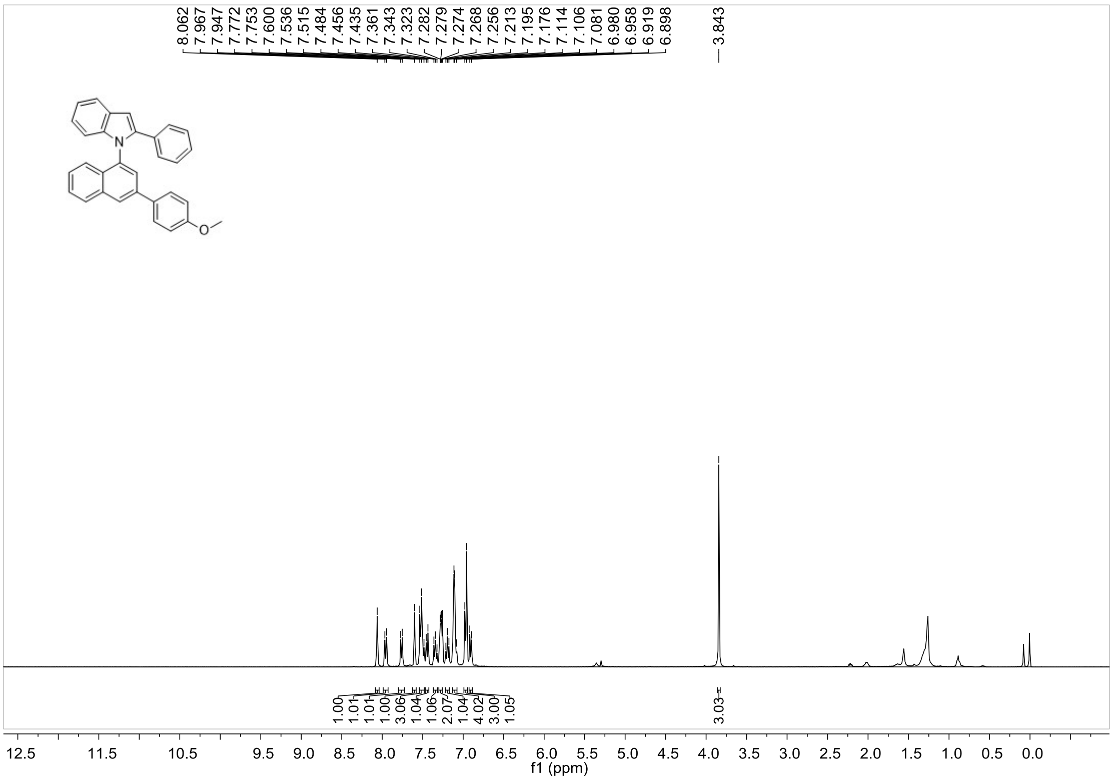

^{13}C NMR Spectrum of **34c**

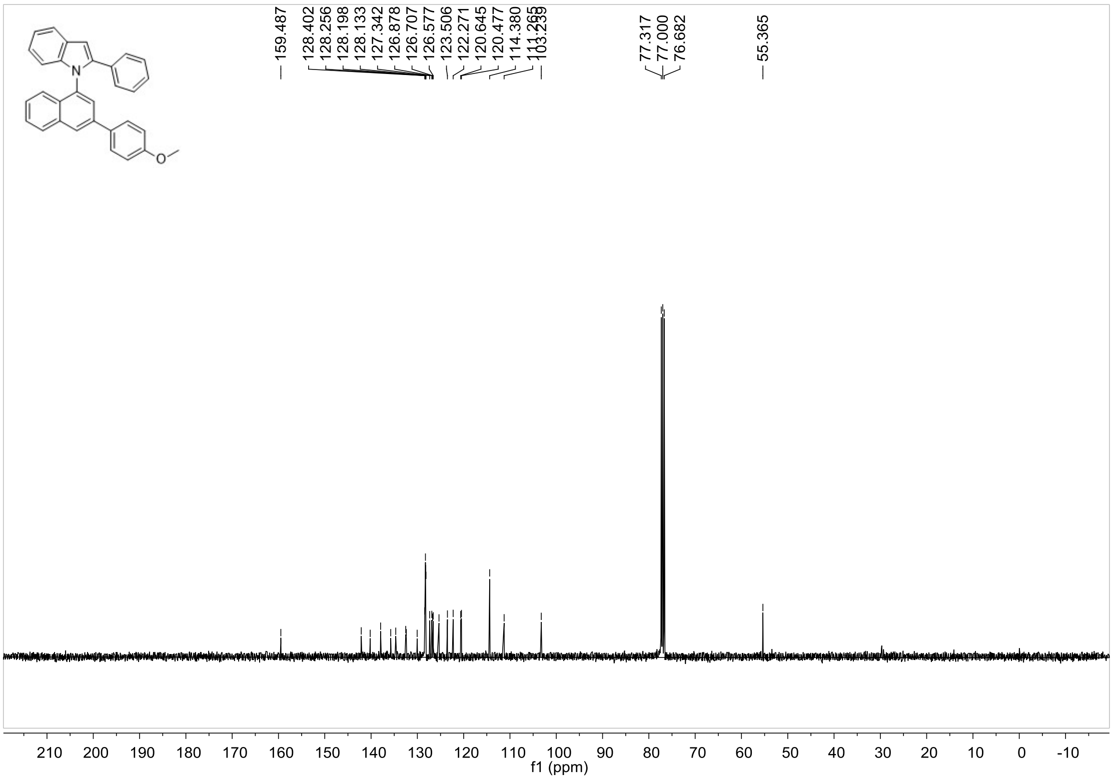

^{1}H NMR Spectrum of **35c**

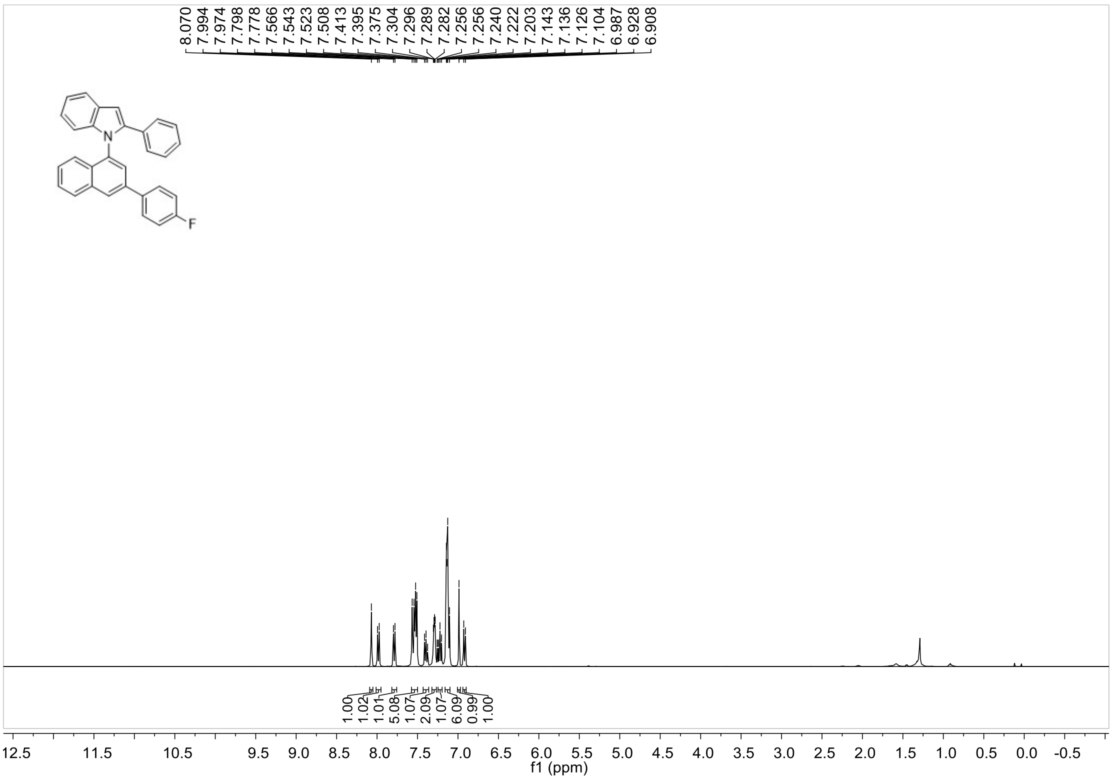

^{13}C NMR Spectrum of **35c**

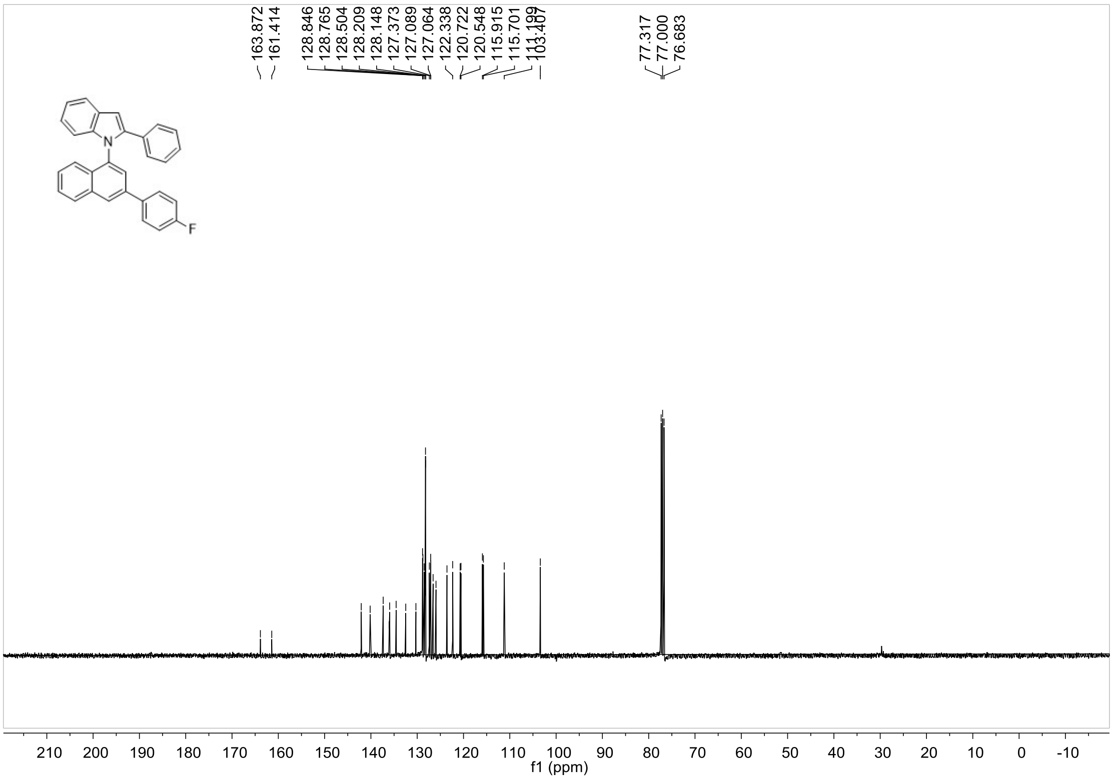

^{19}F NMR Spectrum of **35c**

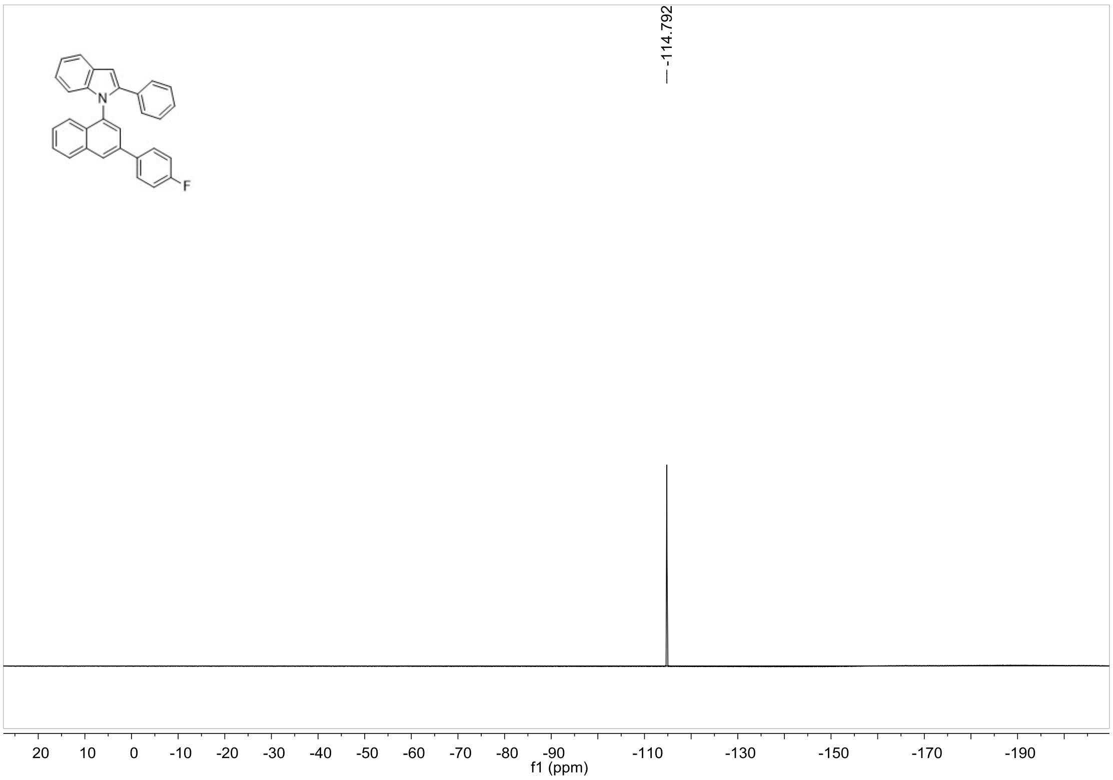

^{1}H NMR Spectrum of **36c**

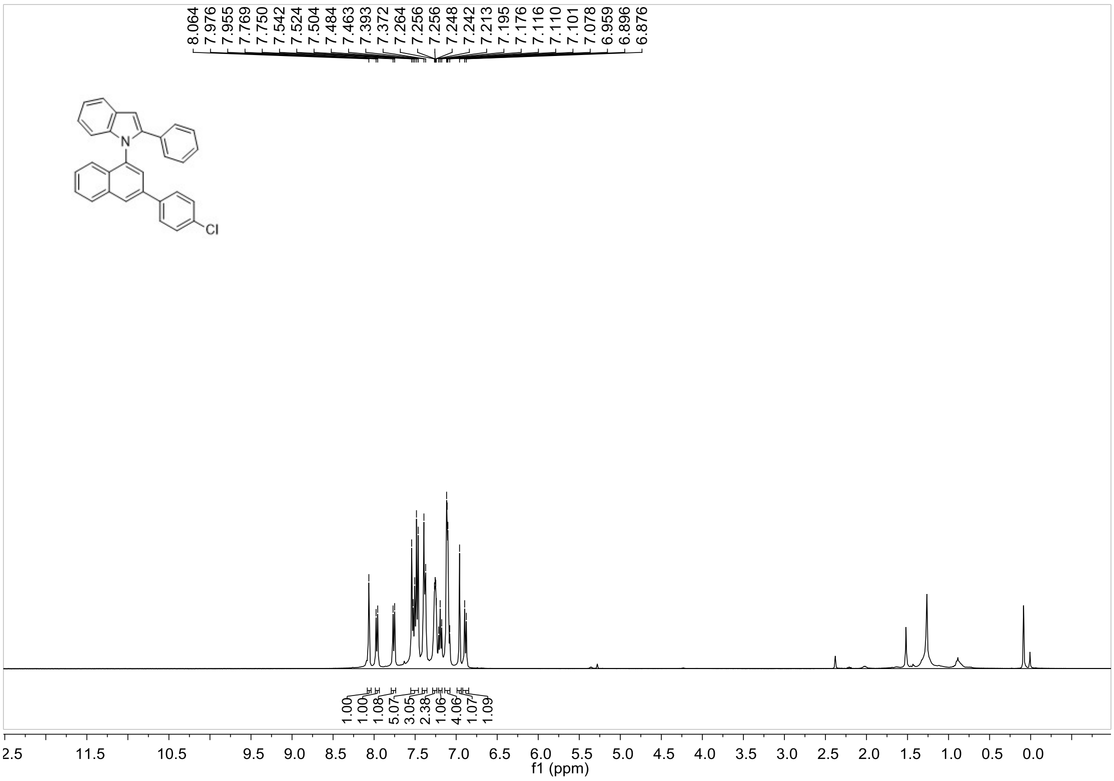

^{13}C NMR Spectrum of **36c**

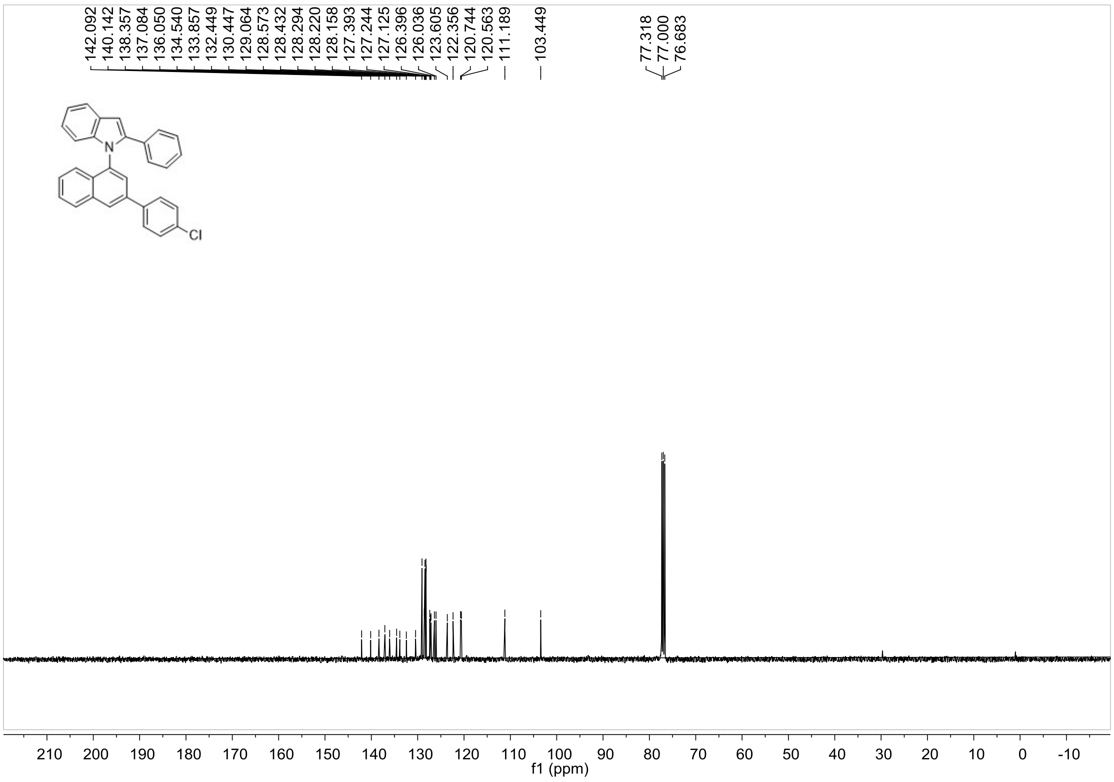

^{1}H NMR Spectrum of **37c**

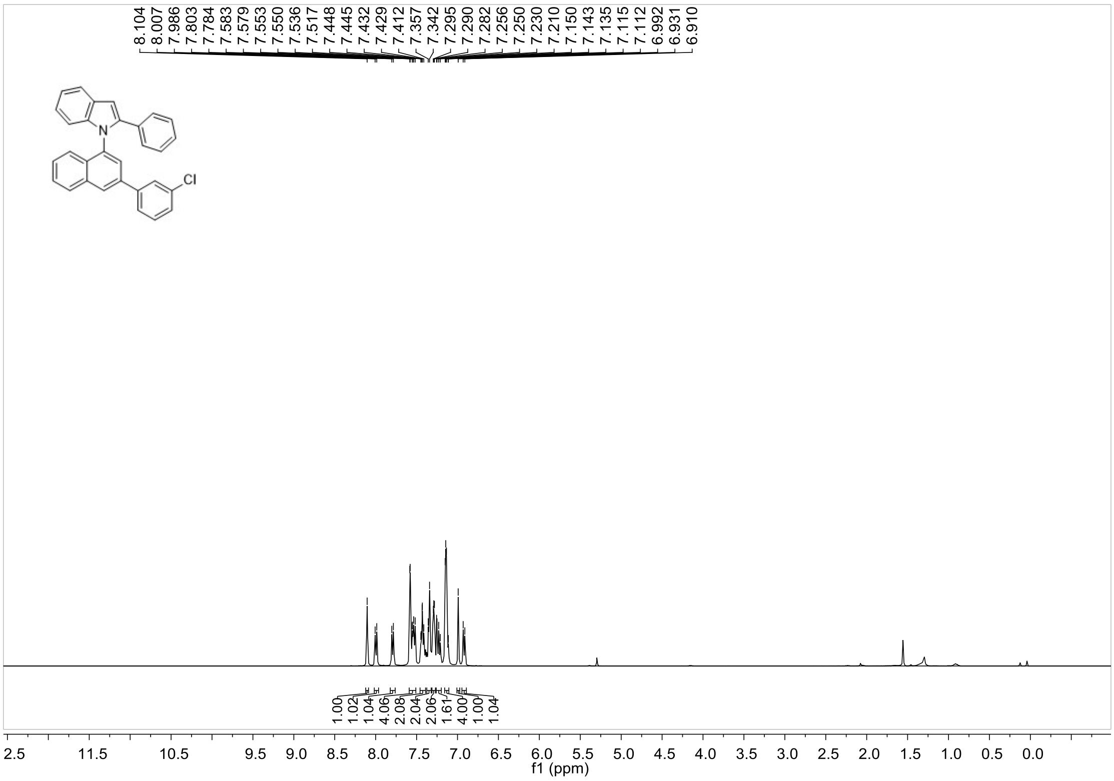

^{13}C NMR Spectrum of **37c**

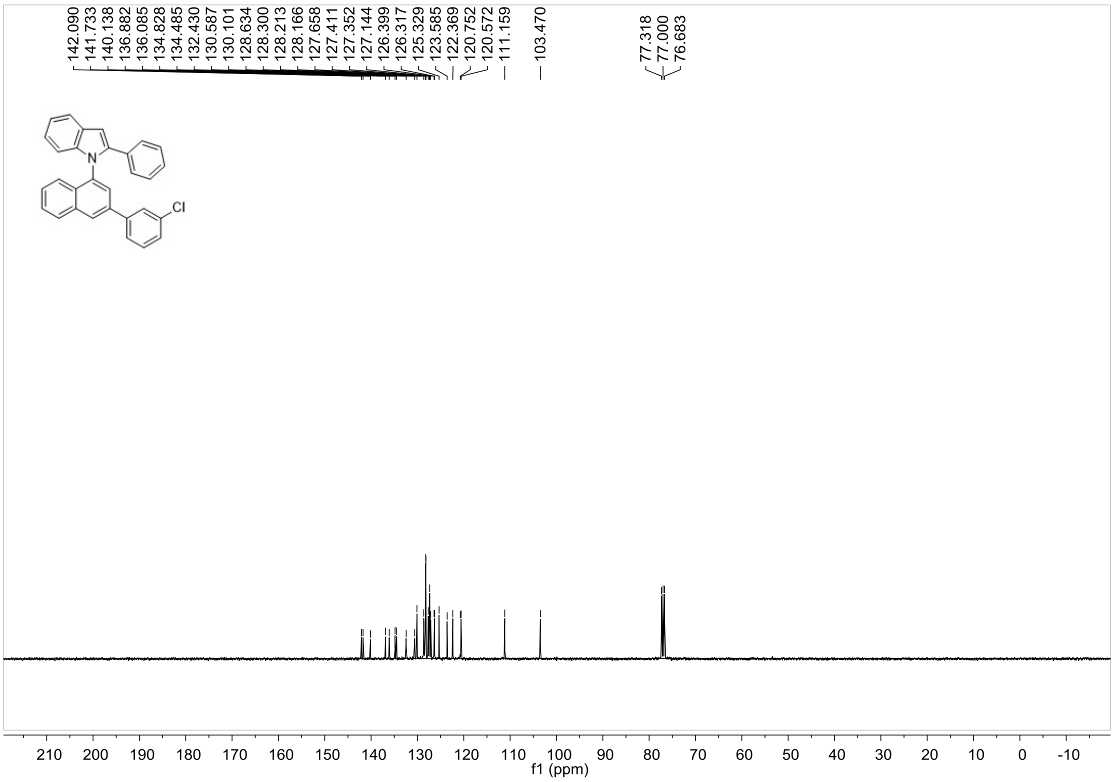

^{1}H NMR Spectrum of **38c**

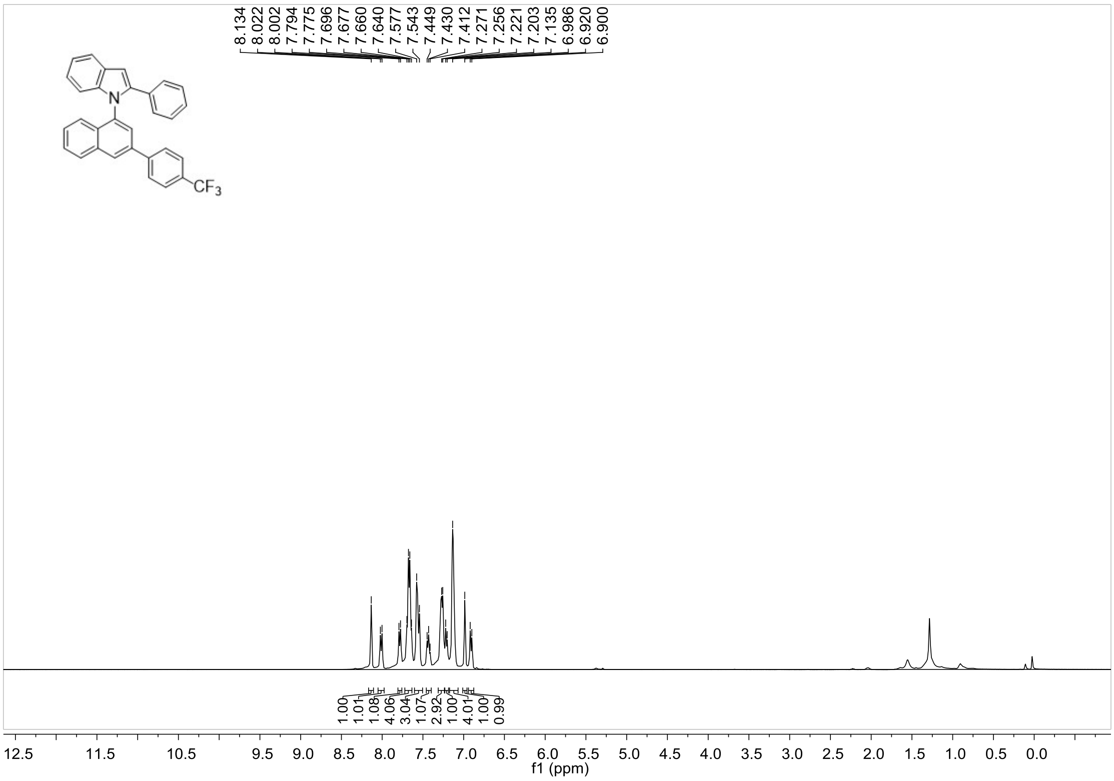

^{13}C NMR Spectrum of **38c**

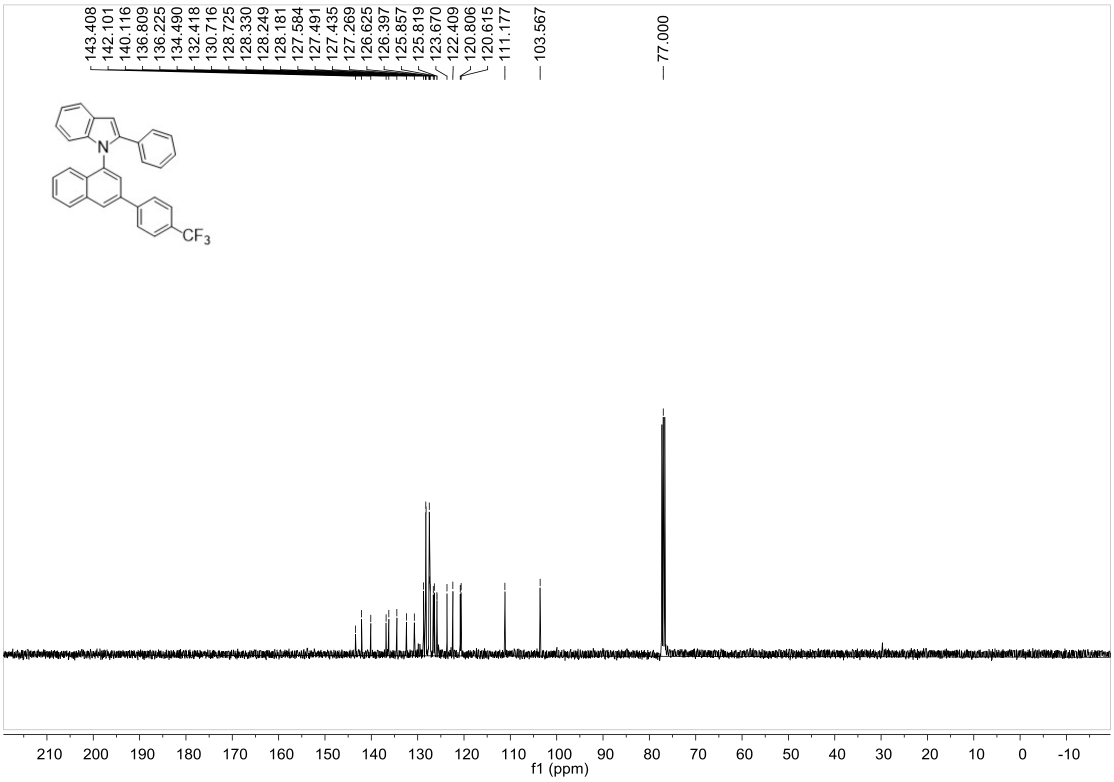

^{19}F NMR Spectrum of **38c**

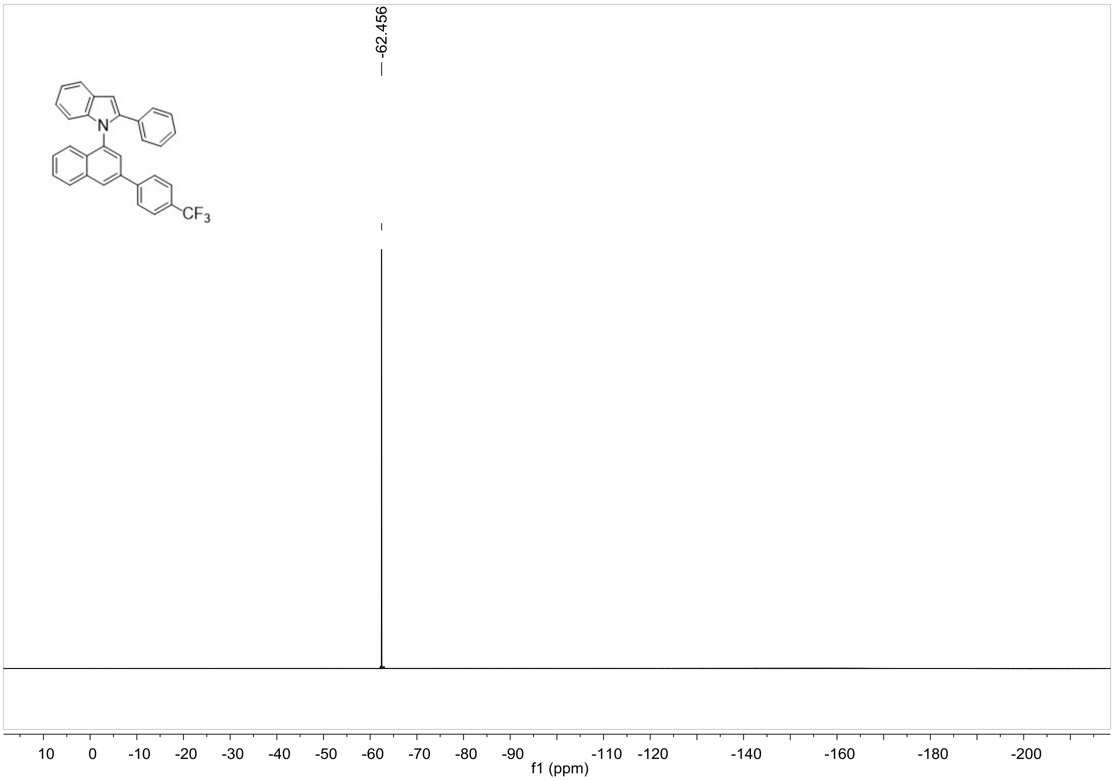

^{1}H NMR Spectrum of **39c**

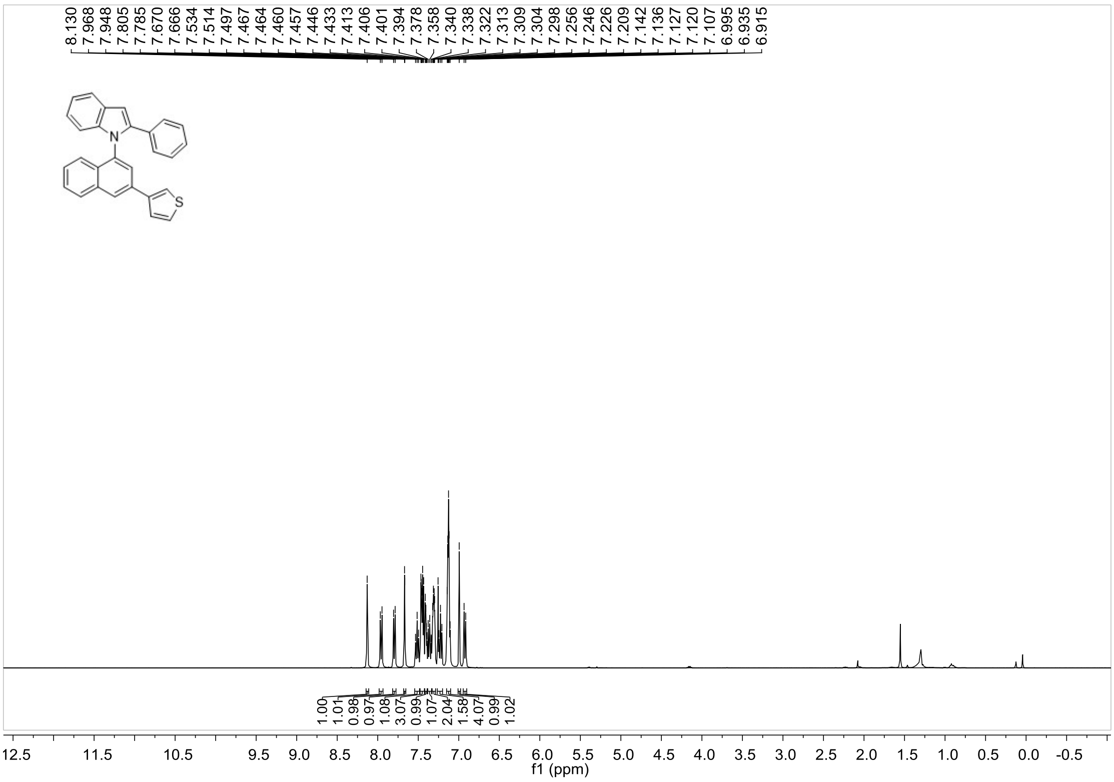

^{13}C NMR Spectrum of **39c**

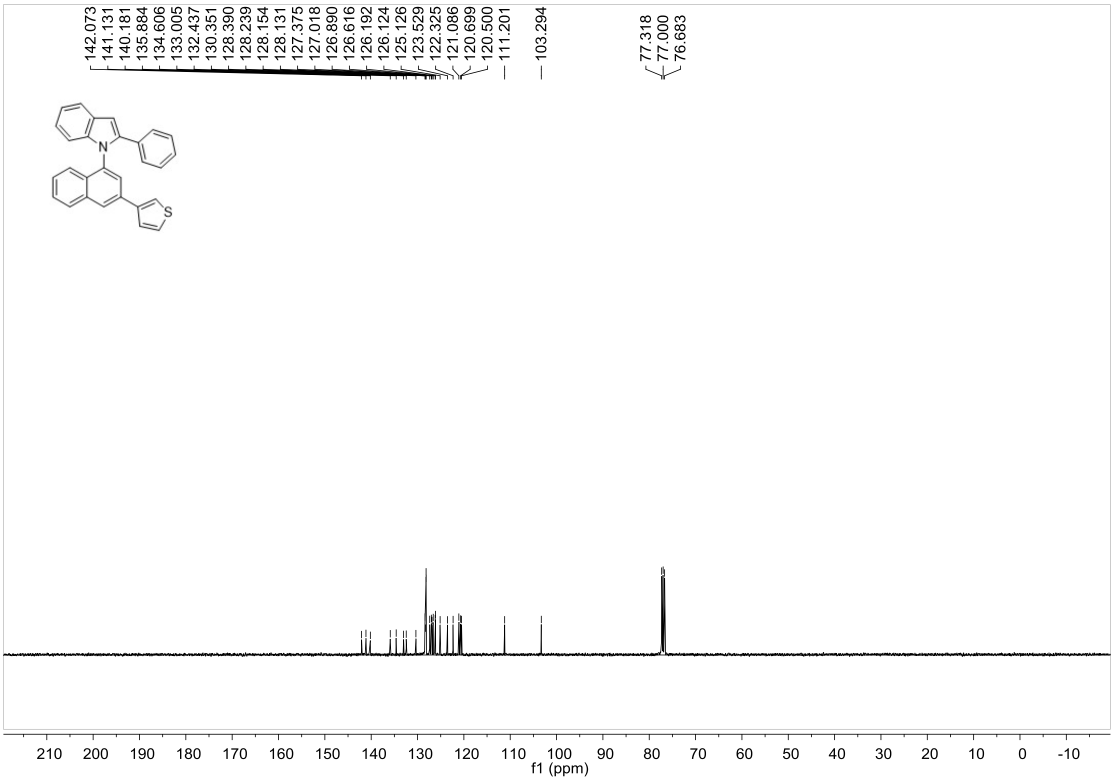

^{1}H NMR Spectrum of **40c**

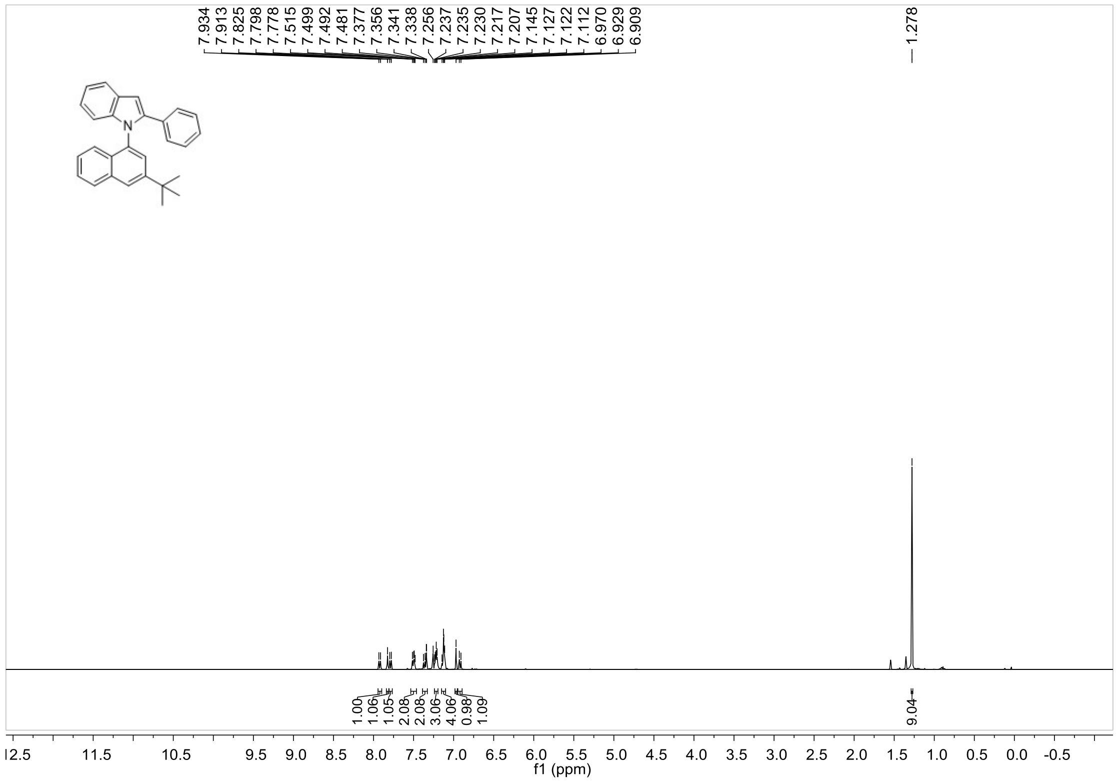

^{13}C NMR Spectrum of **40c**

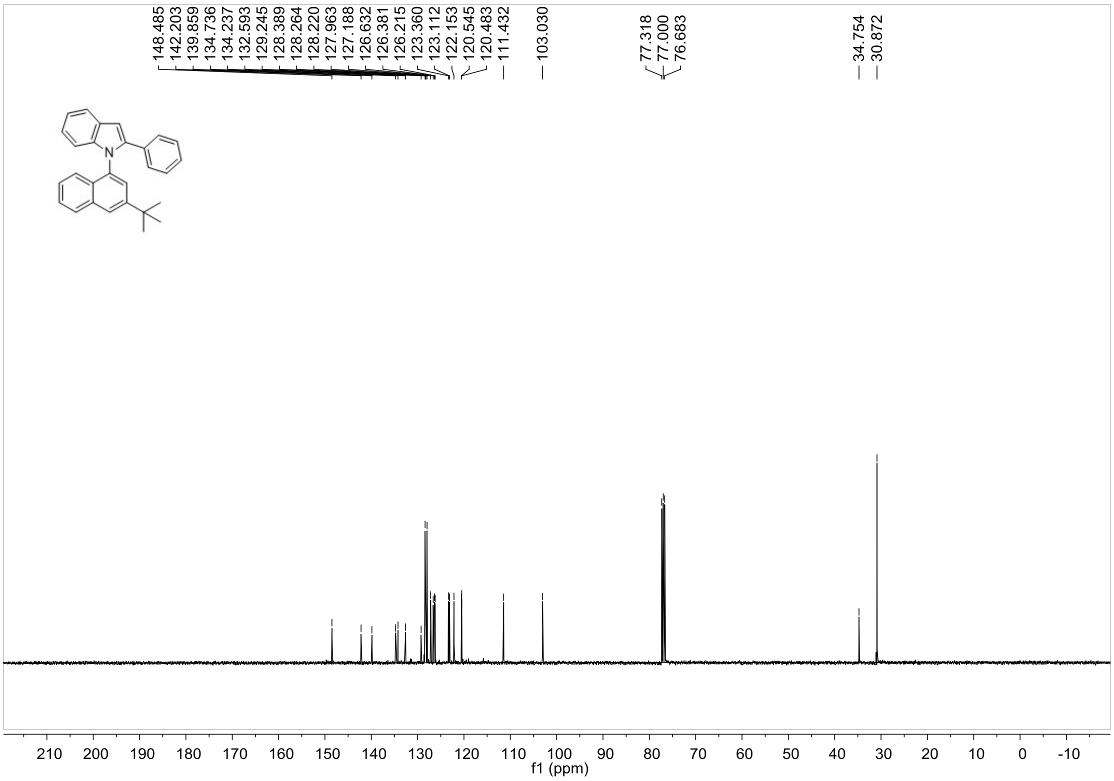

^{1}H NMR Spectrum of **41c**

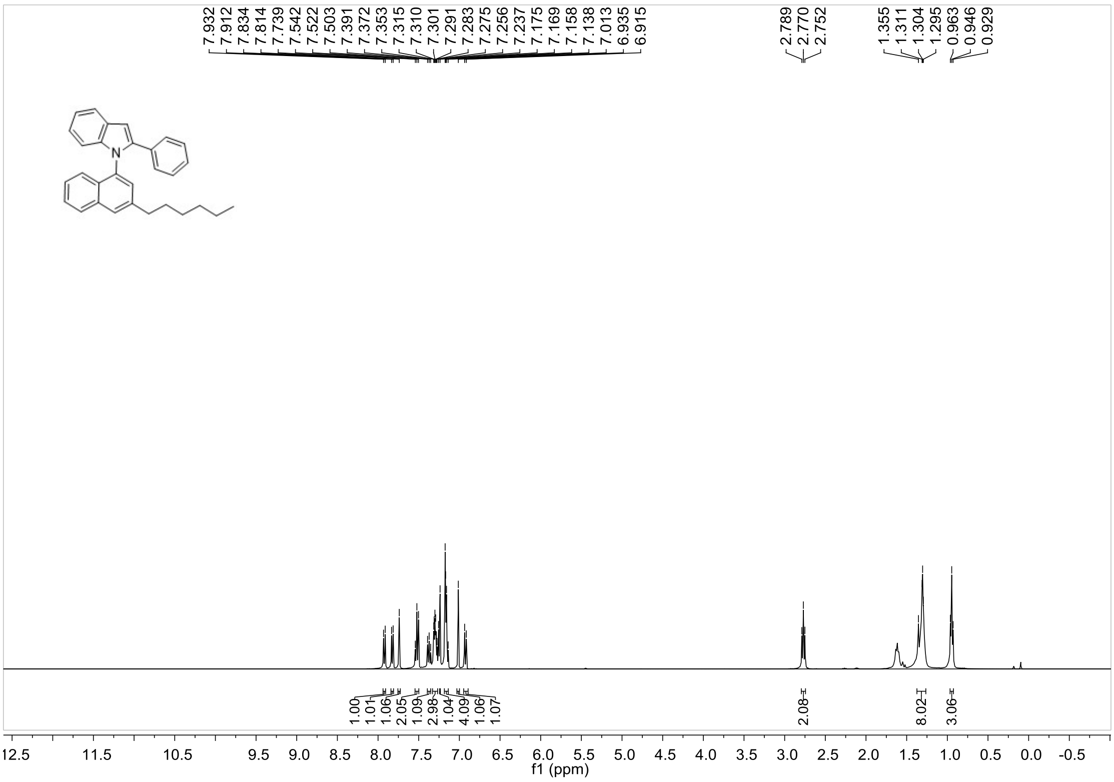

^{13}C NMR Spectrum of **41c**

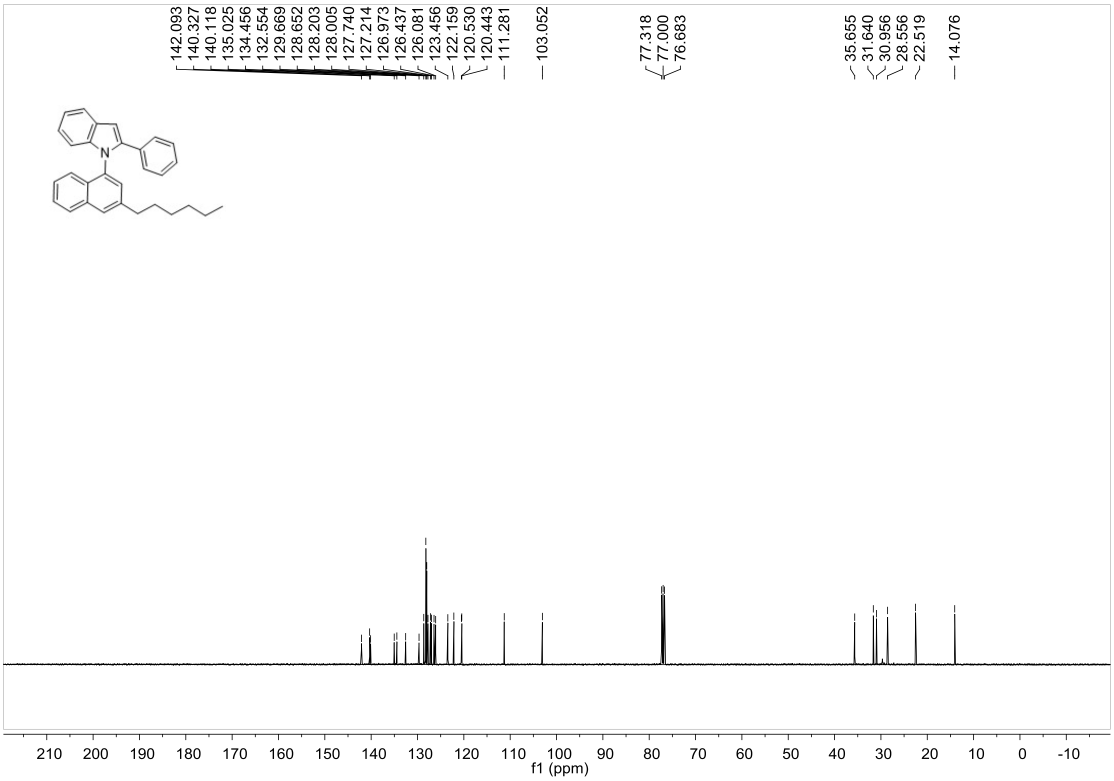

^{1}H NMR Spectrum of **42c**

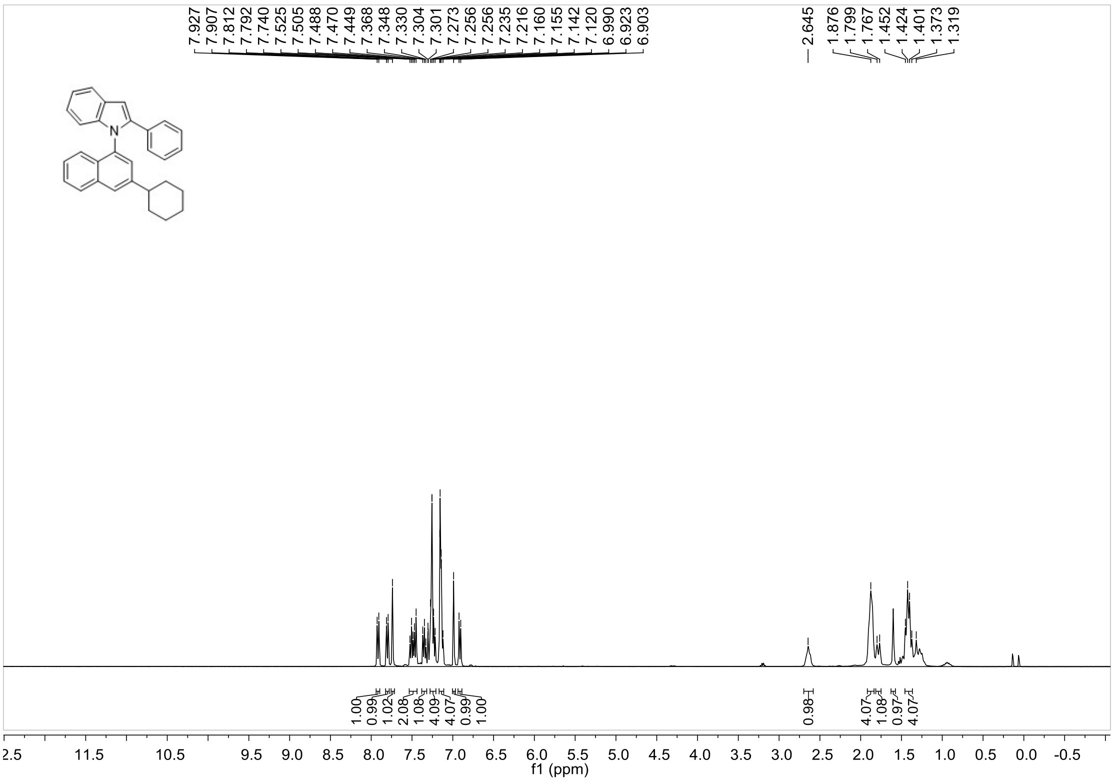

^{13}C NMR Spectrum of **42c**

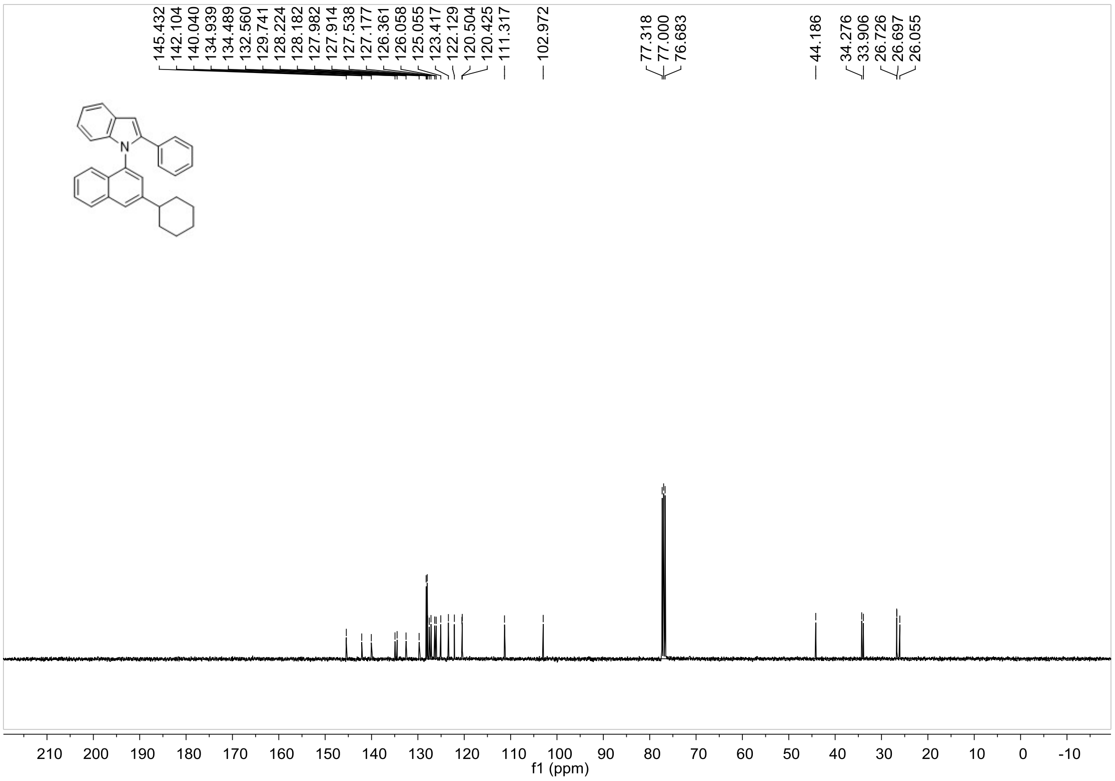

^{1}H NMR Spectrum of **43c**

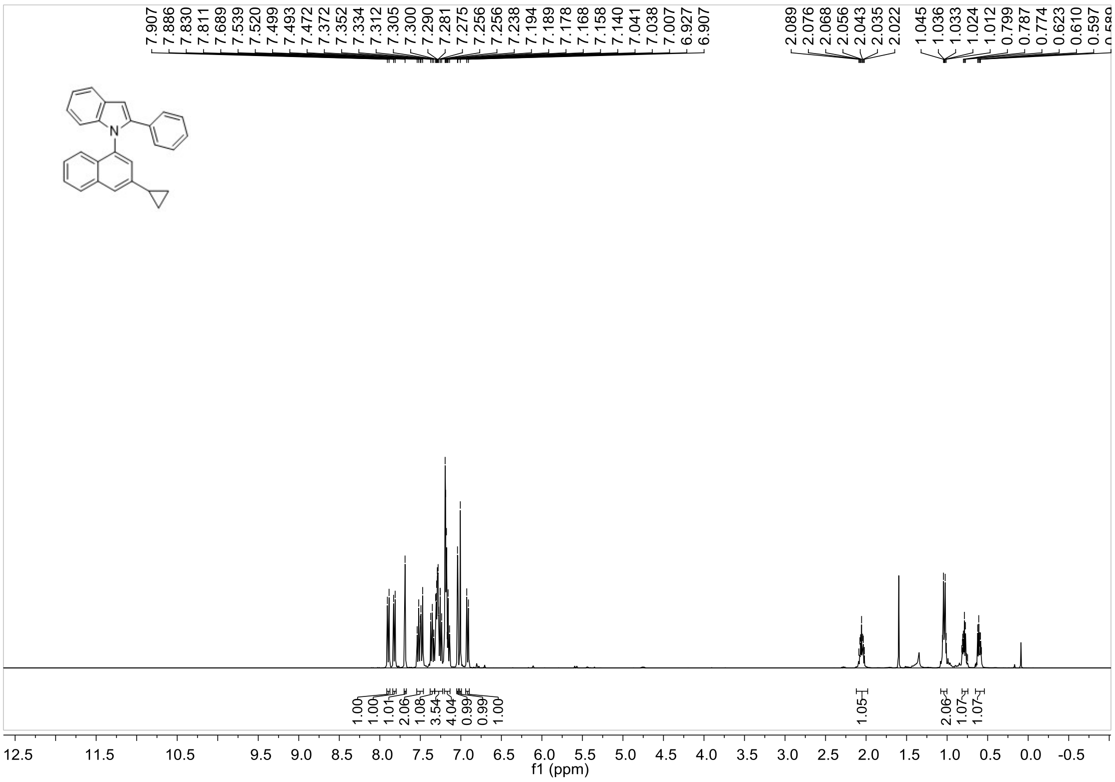

^{13}C NMR Spectrum of **43c**

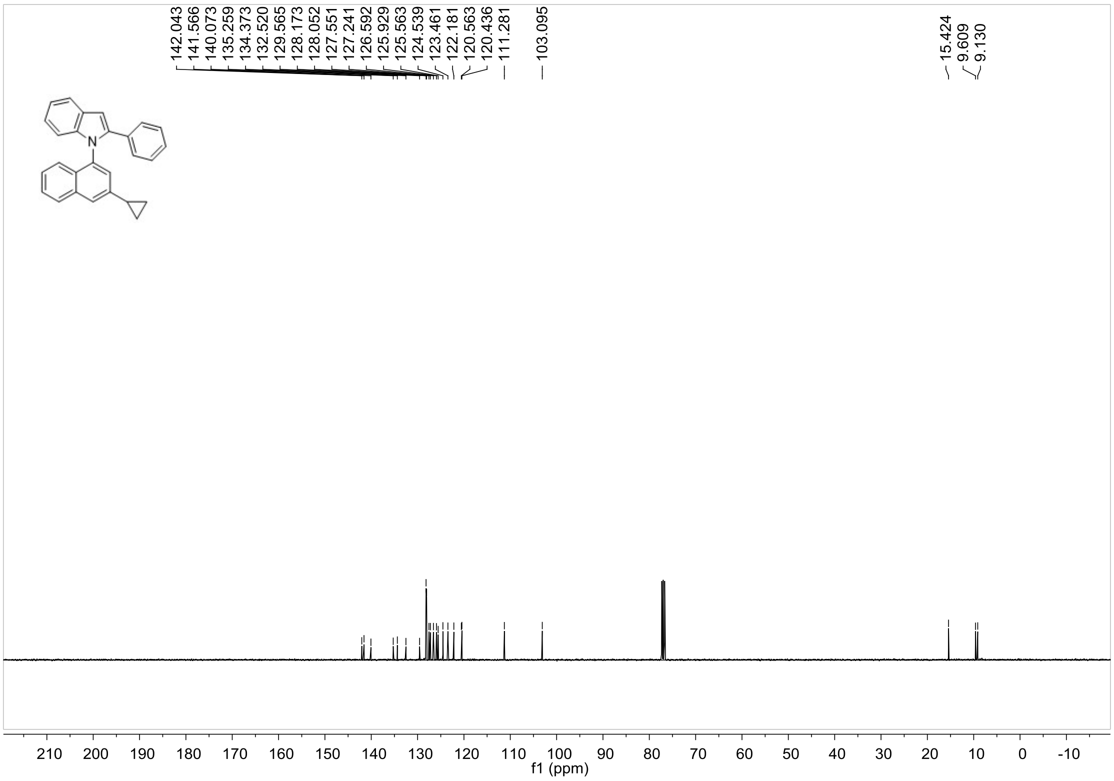

^{1}H NMR Spectrum of **44c**

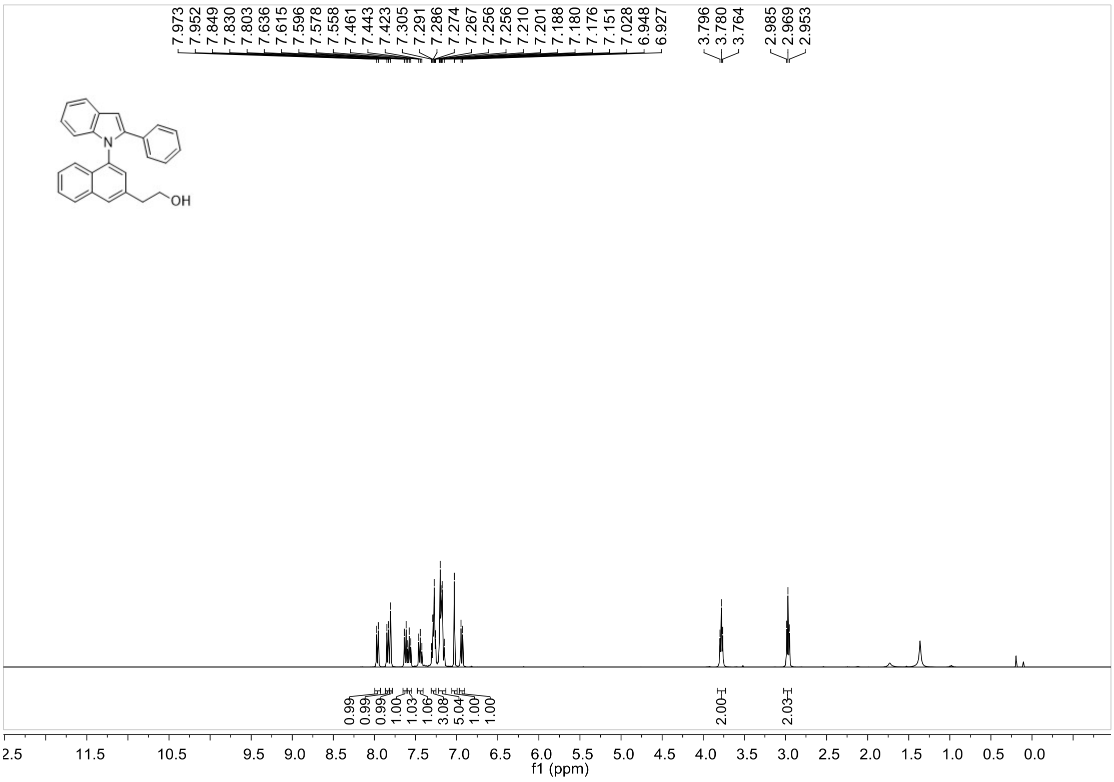

^{13}C NMR Spectrum of **44c**

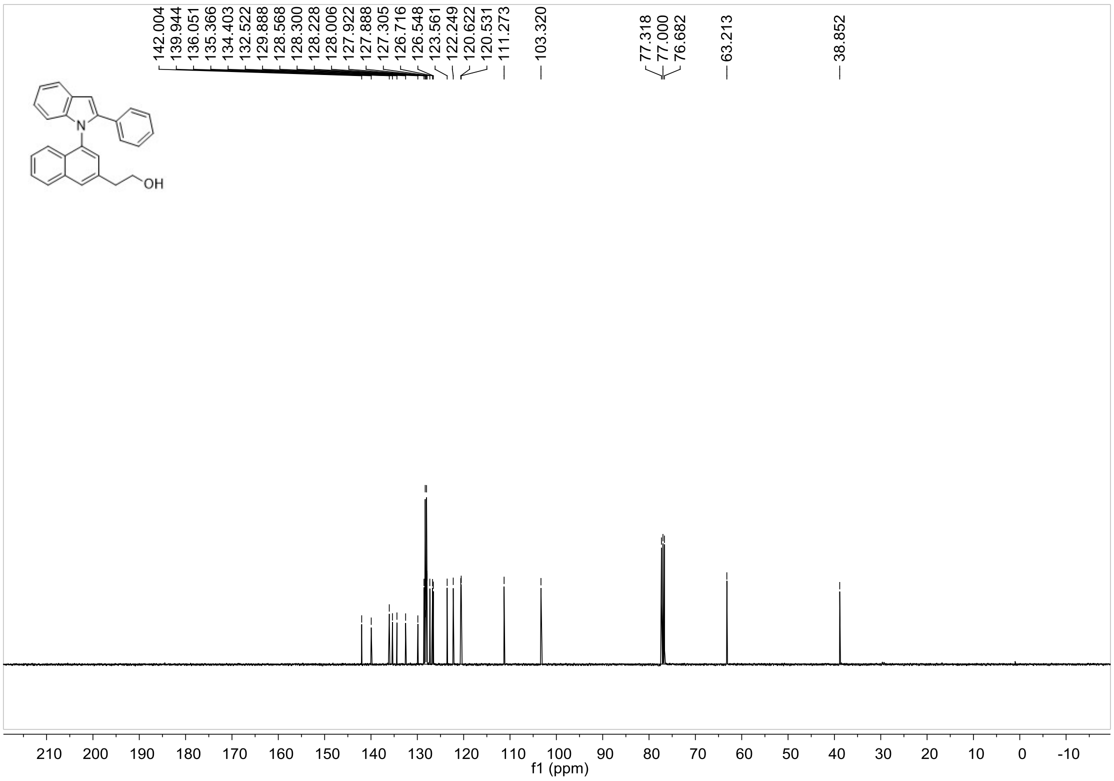

^{1}H NMR Spectrum of **45c**

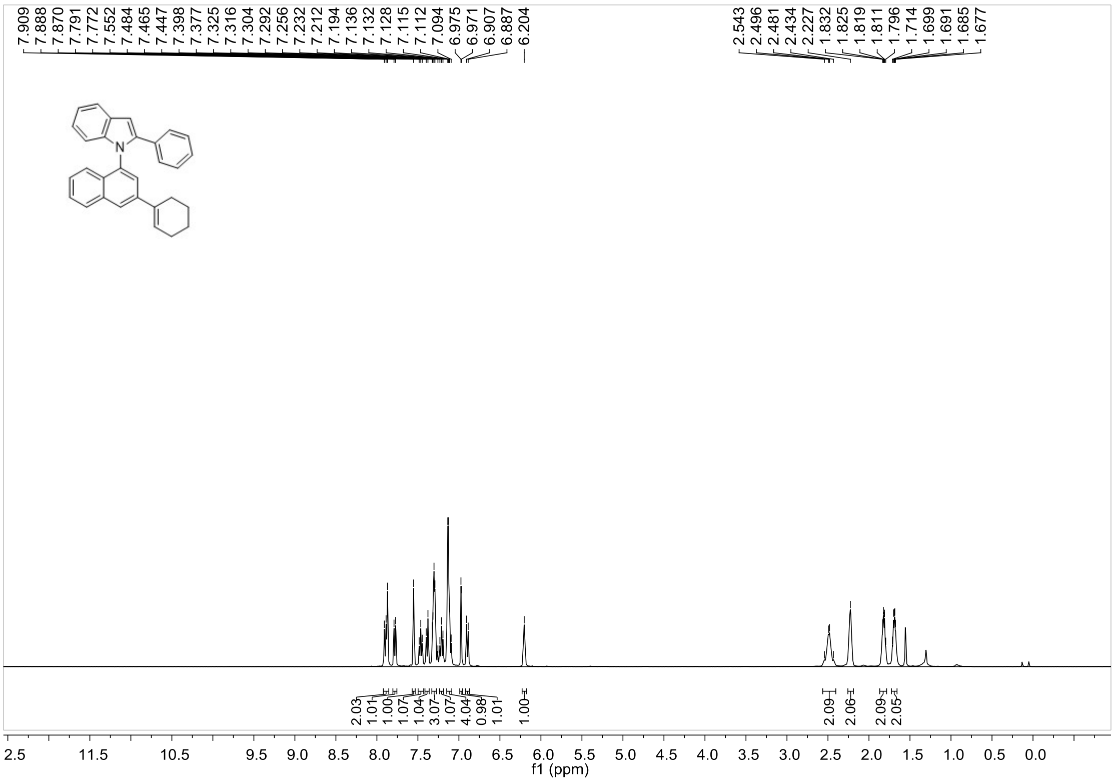

^{13}C NMR Spectrum of **45c**

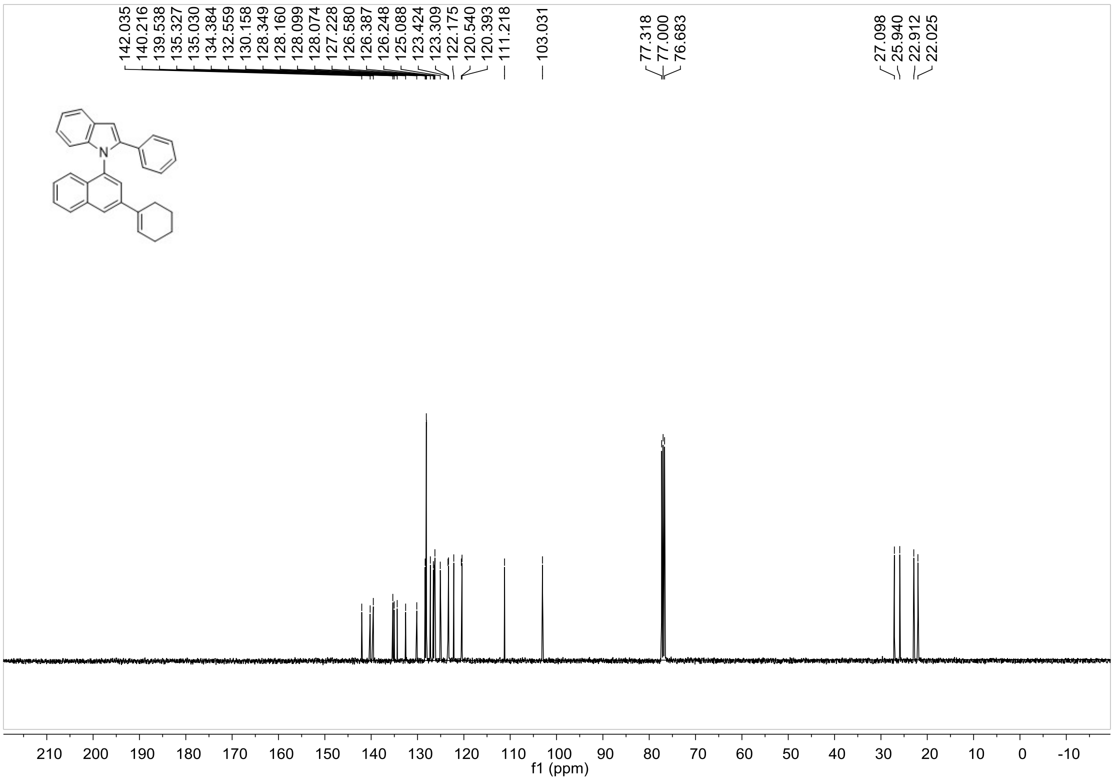

^{1}H NMR Spectrum of **46c**

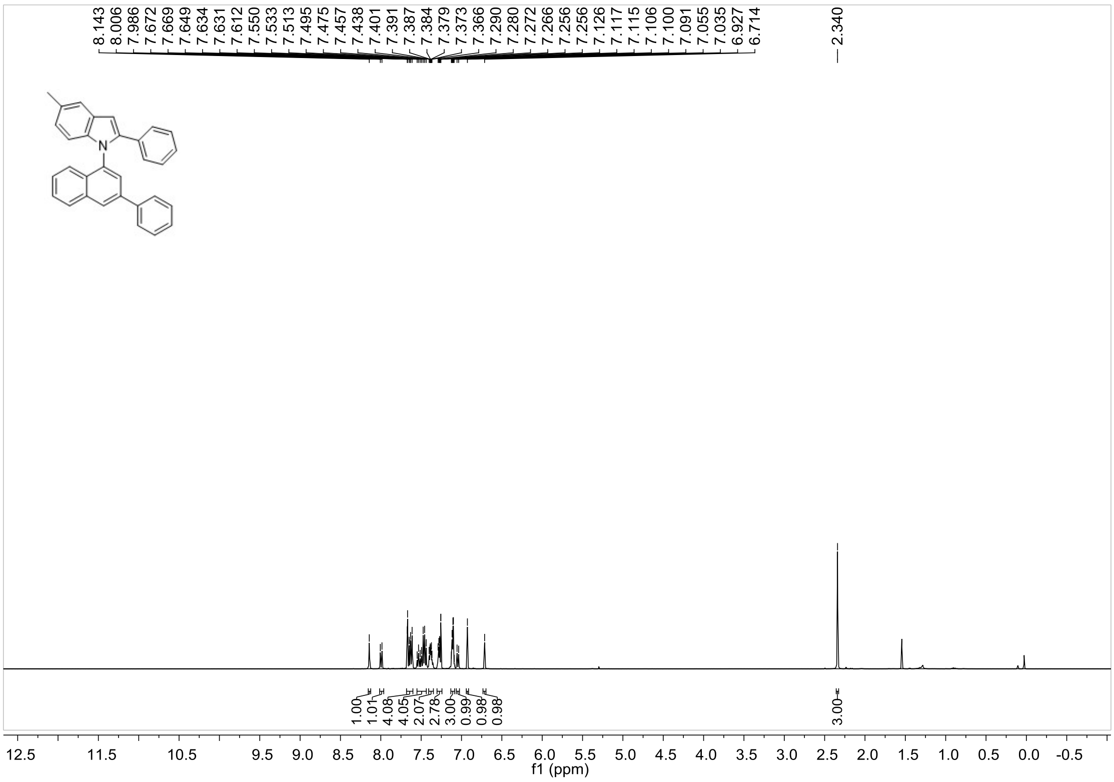

^{13}C NMR Spectrum of **46c**

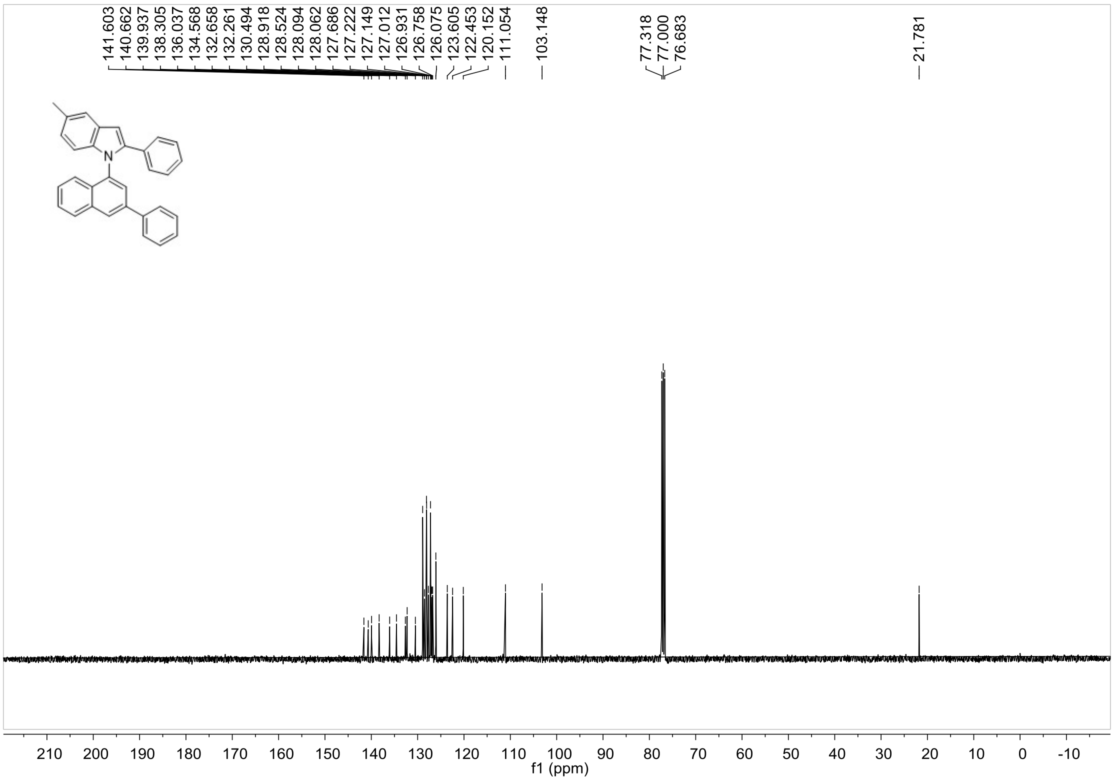

^{1}H NMR Spectrum of **47c**

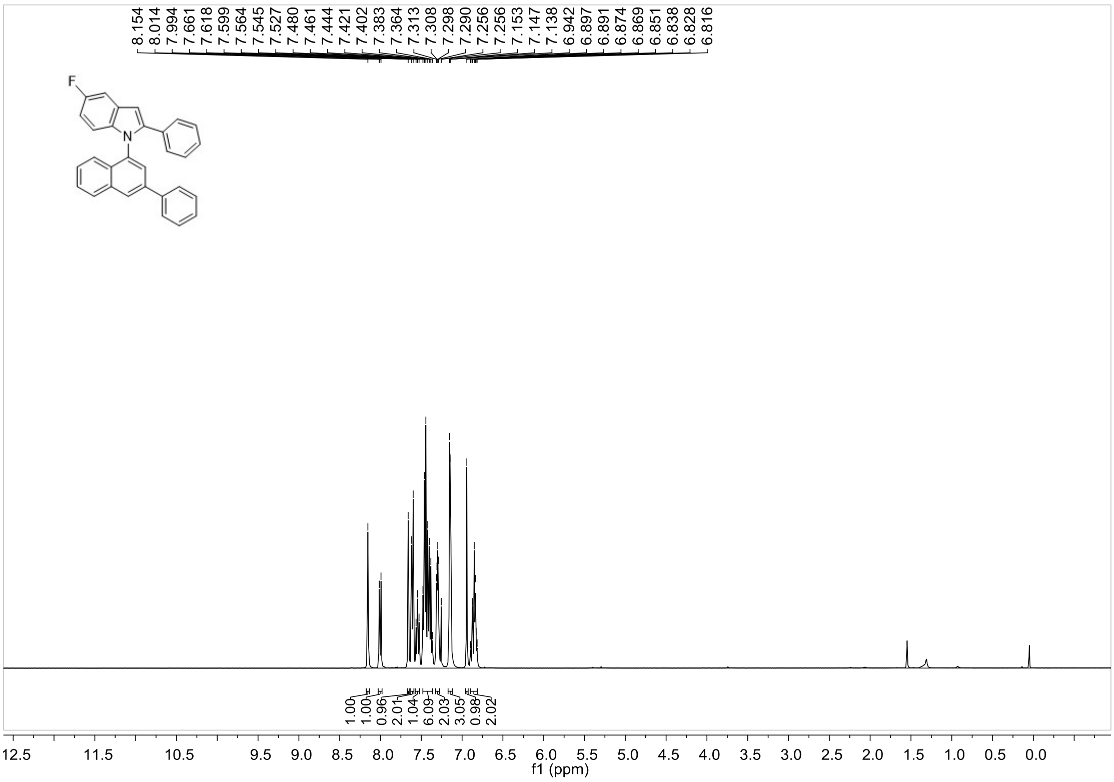

^{13}C NMR Spectrum of **47c**

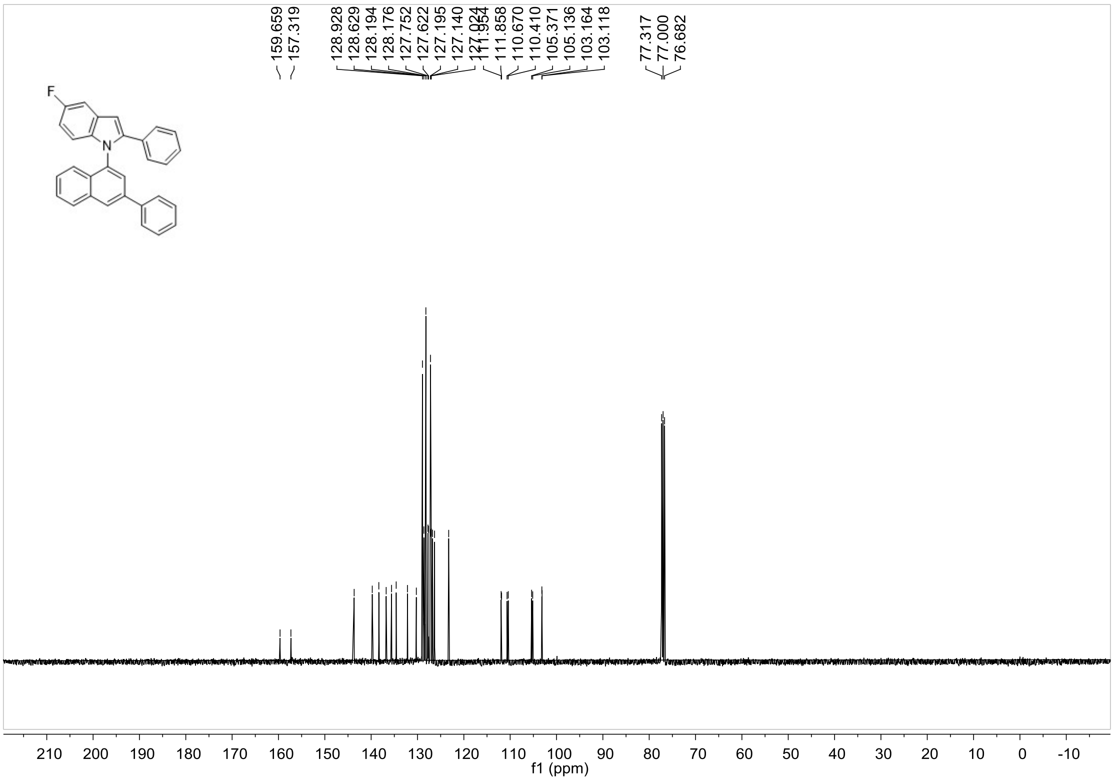

^{19}F NMR Spectrum of **47c**

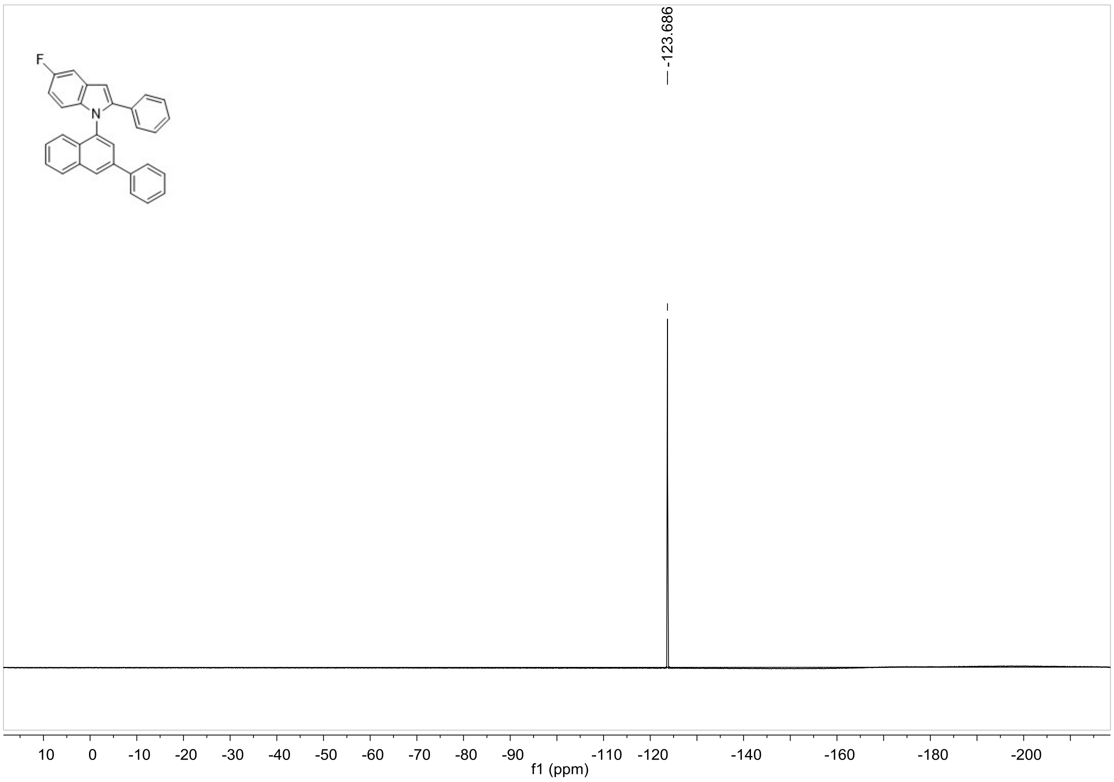

^{1}H NMR Spectrum of **48c**

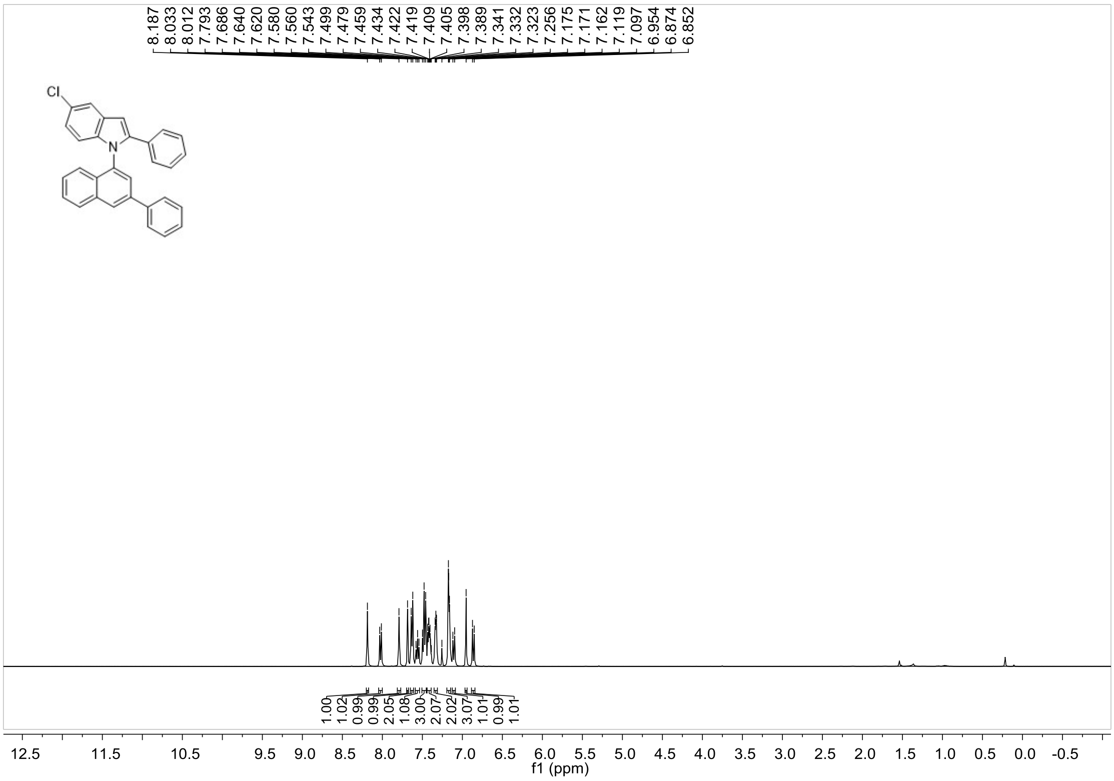

^{13}C NMR Spectrum of **48c**

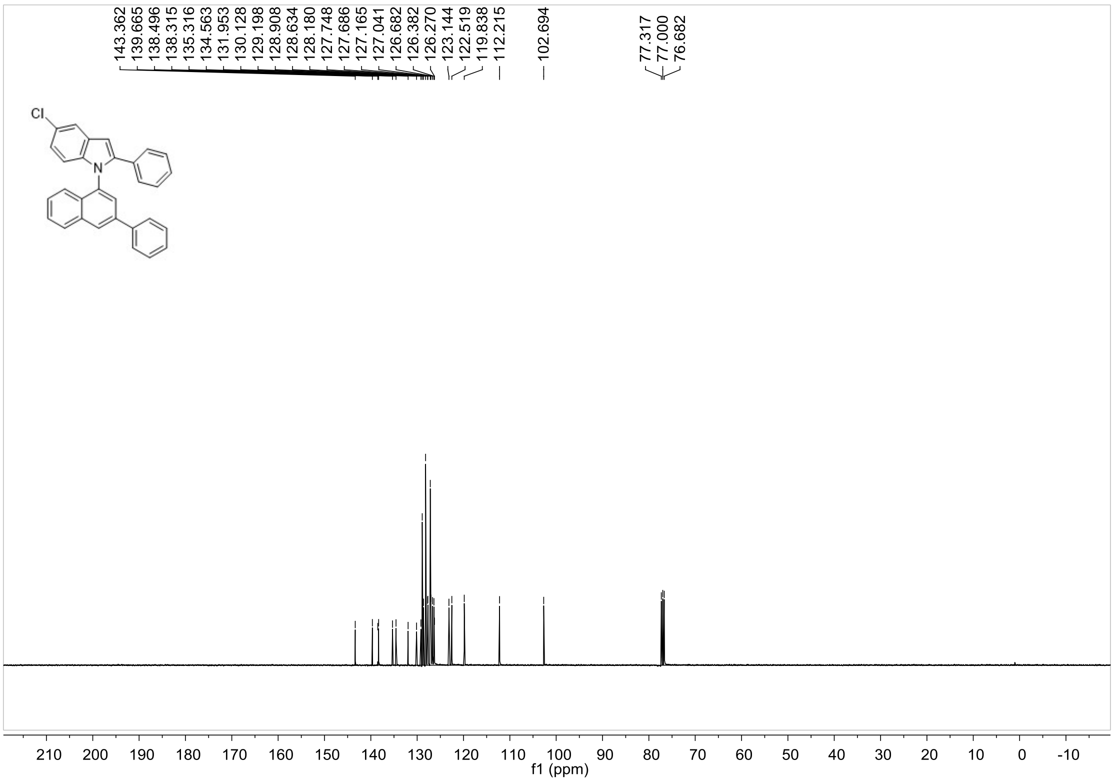

^{1}H NMR Spectrum of **49c**

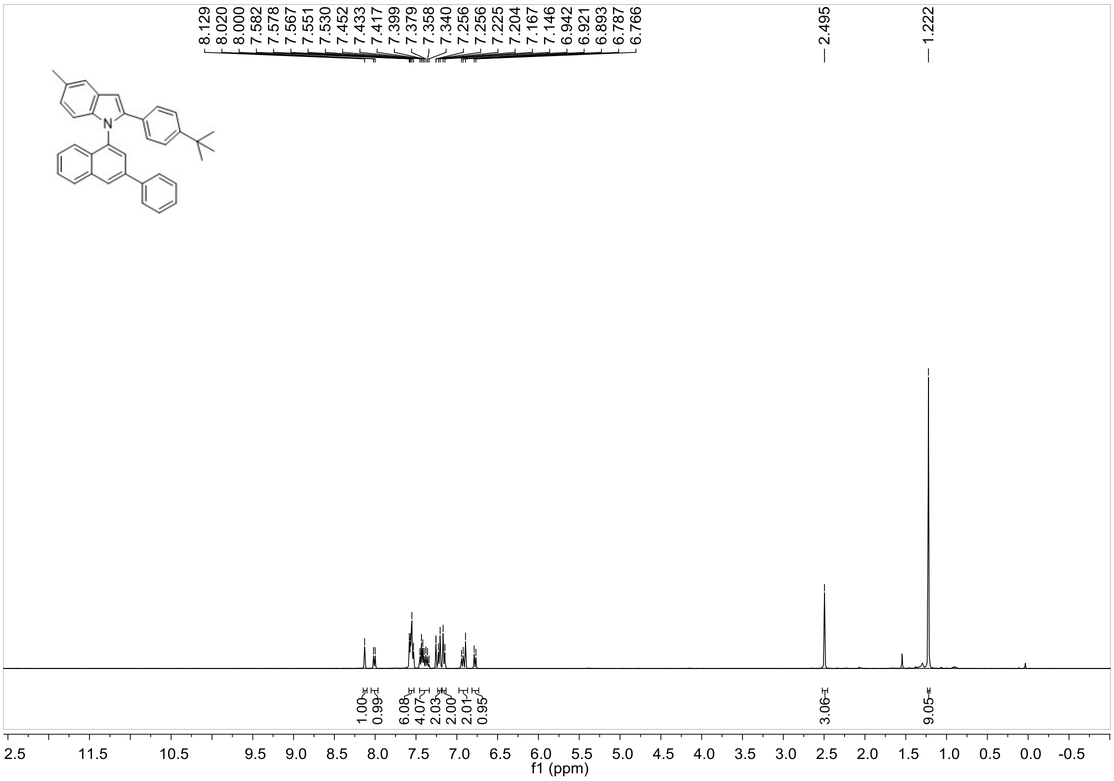

^{13}C NMR Spectrum of **49c**

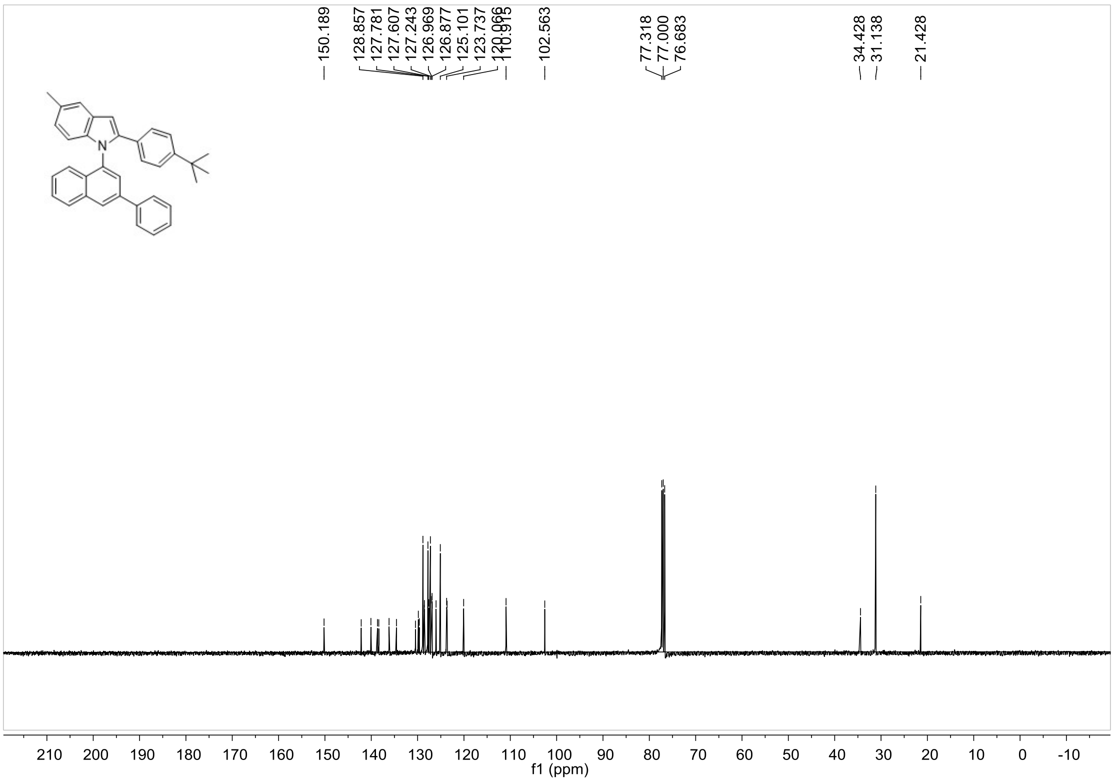

^{1}H NMR Spectrum of **50c**

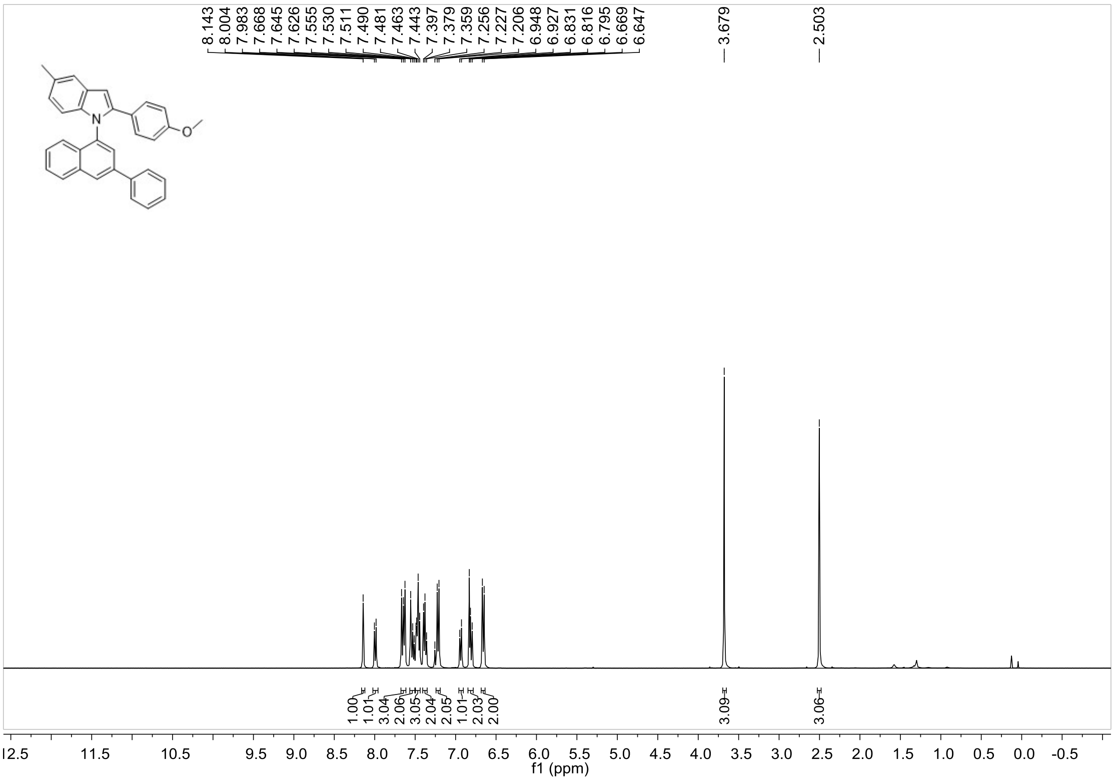

^{13}C NMR Spectrum of **50c**

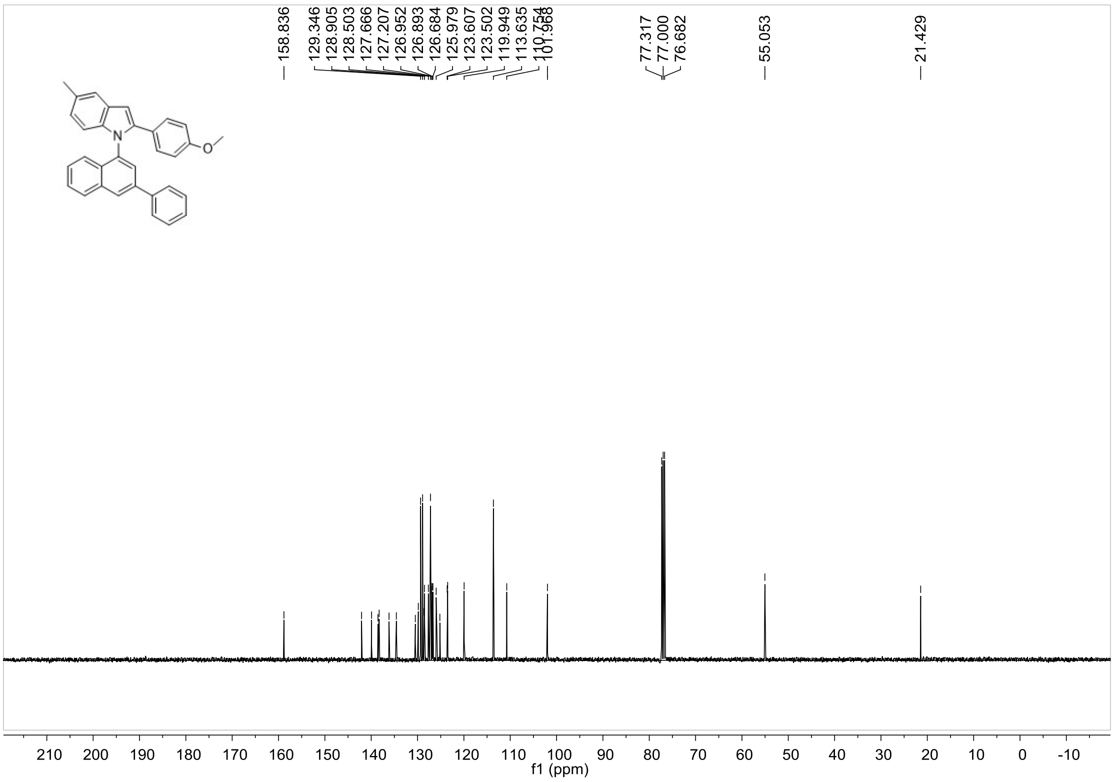

^{1}H NMR Spectrum of **51c**

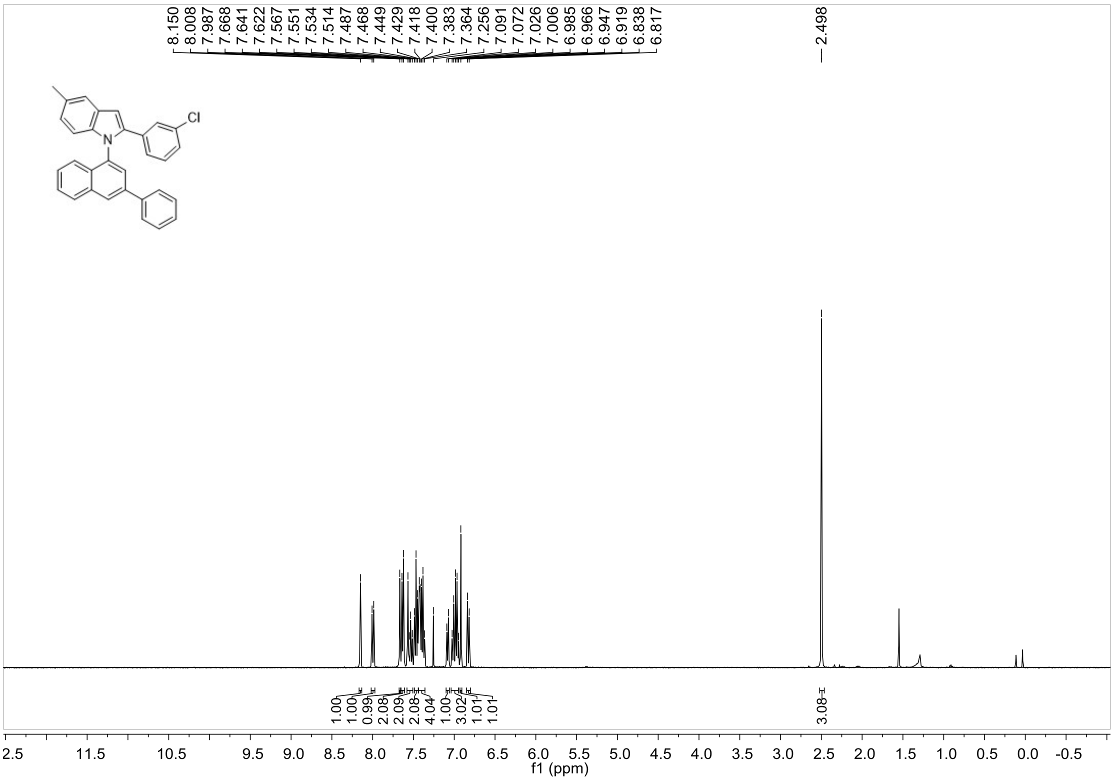

^{13}C NMR Spectrum of **51c**

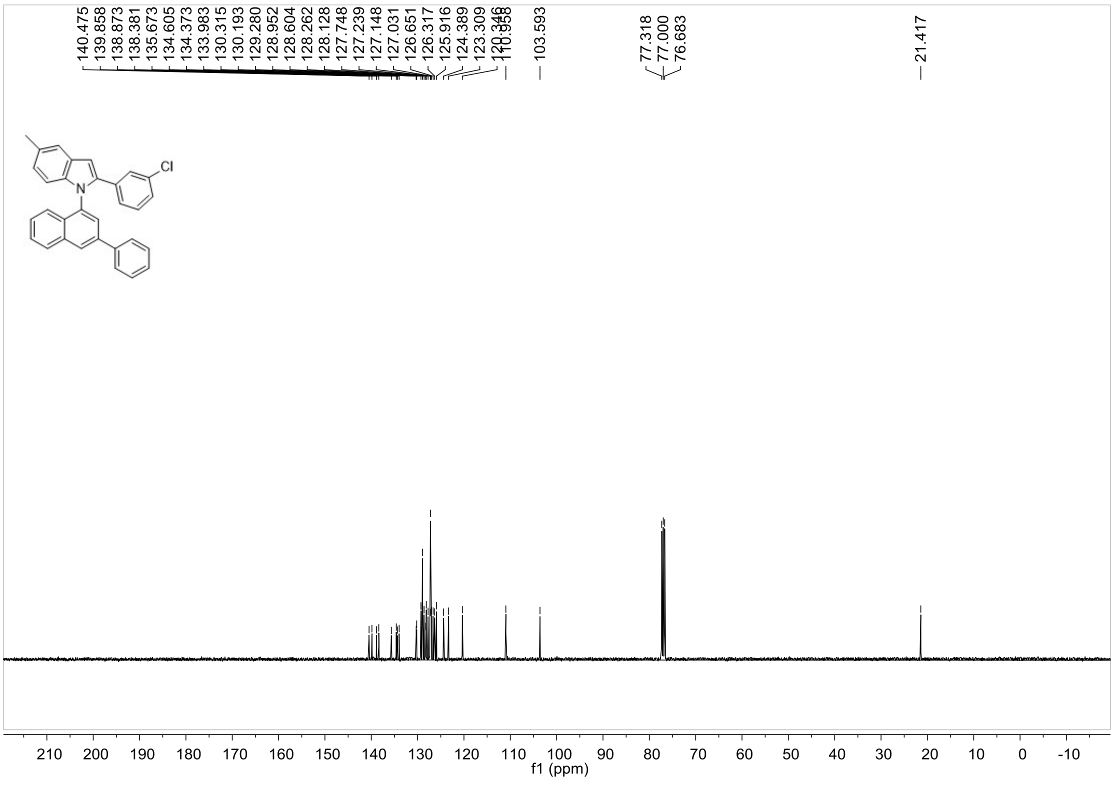

^{1}H NMR Spectrum of **52c**

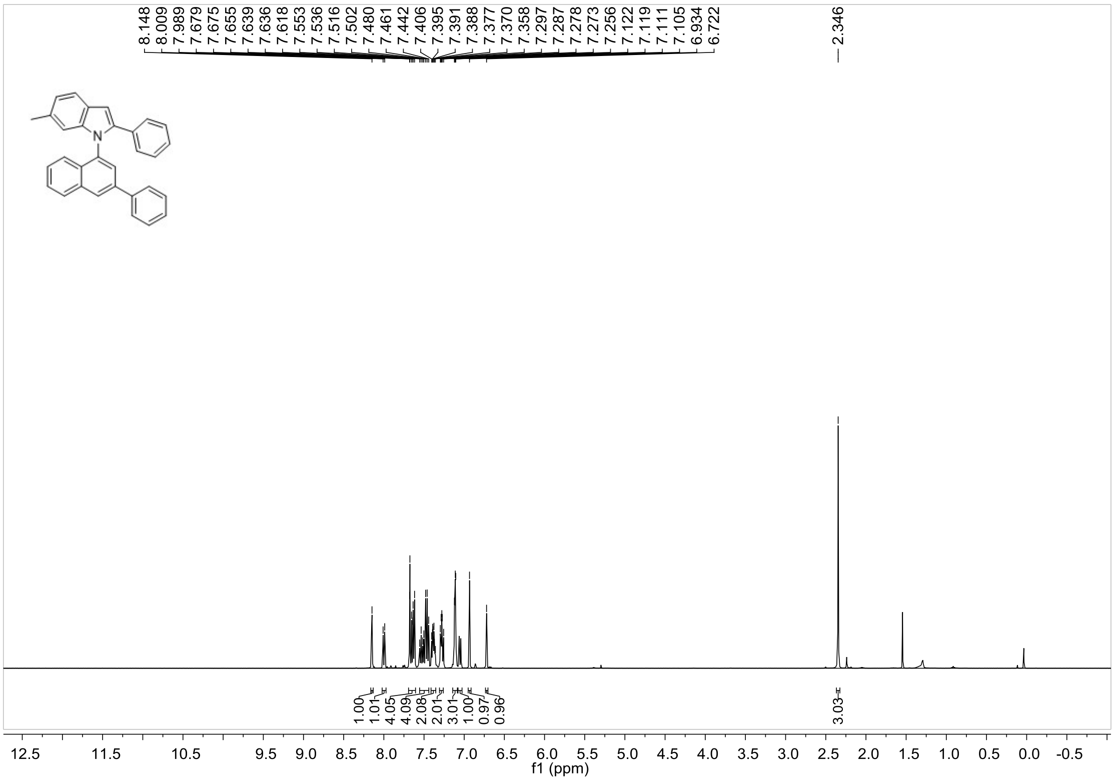

^{13}C NMR Spectrum of **52c**

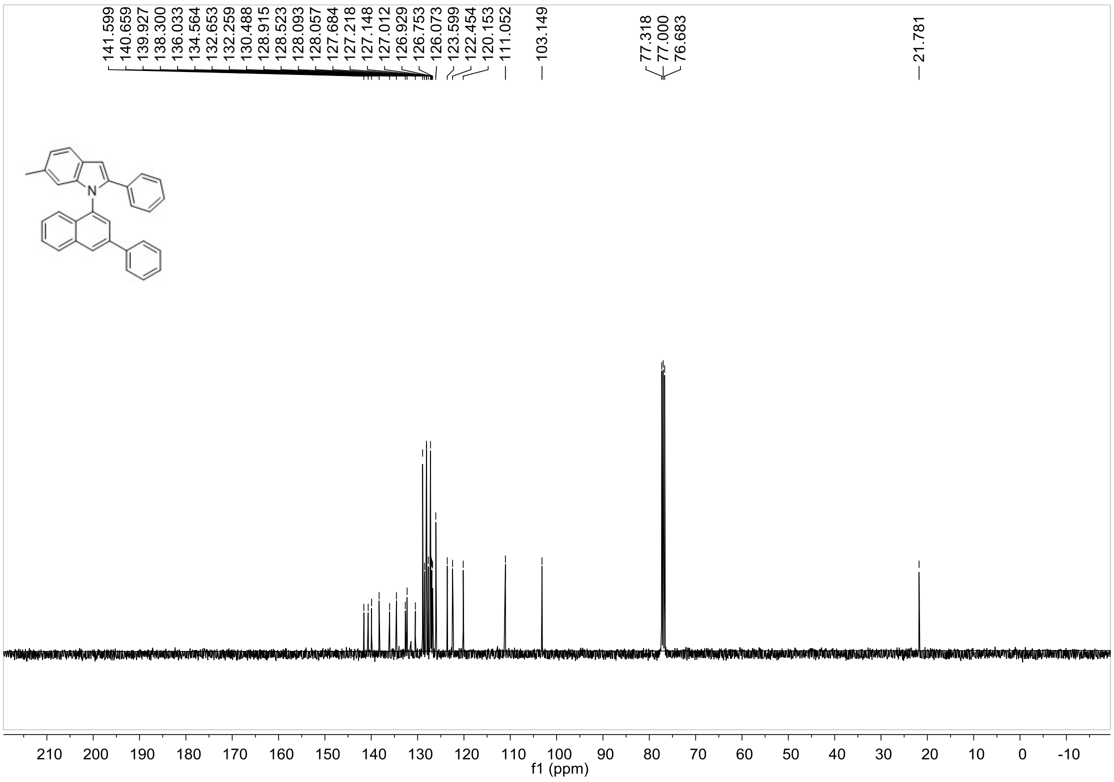

^{1}H NMR Spectrum of **53c**

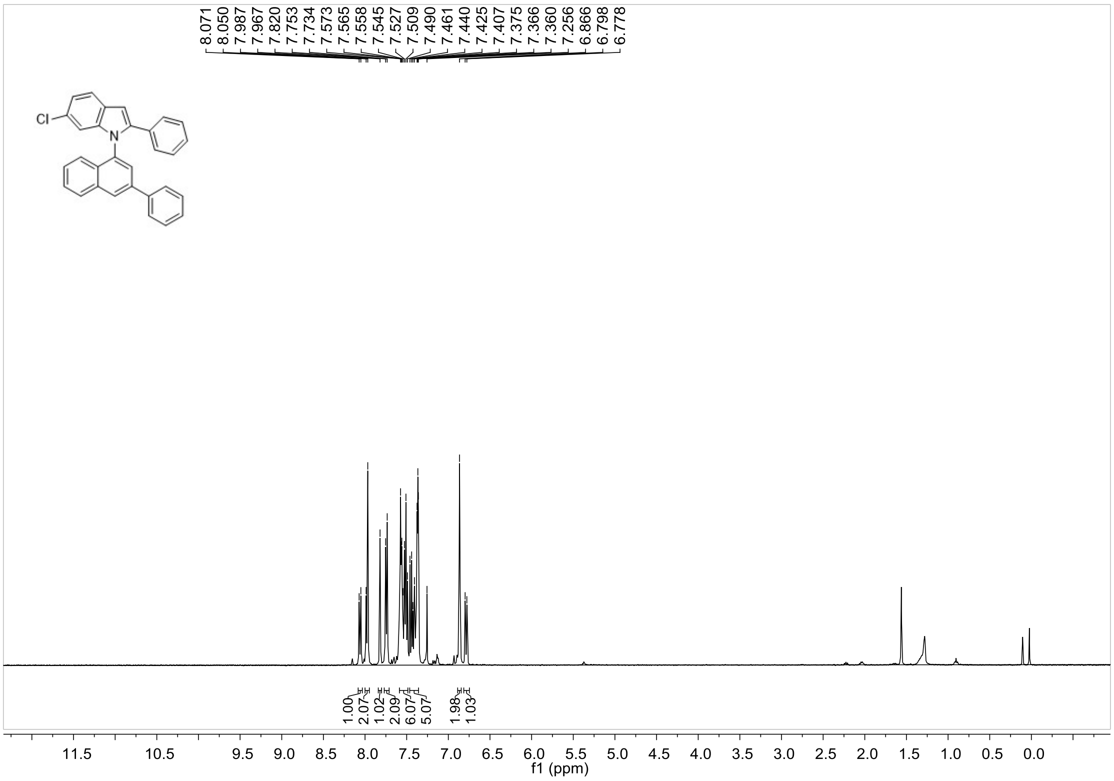

^{13}C NMR Spectrum of **53c**

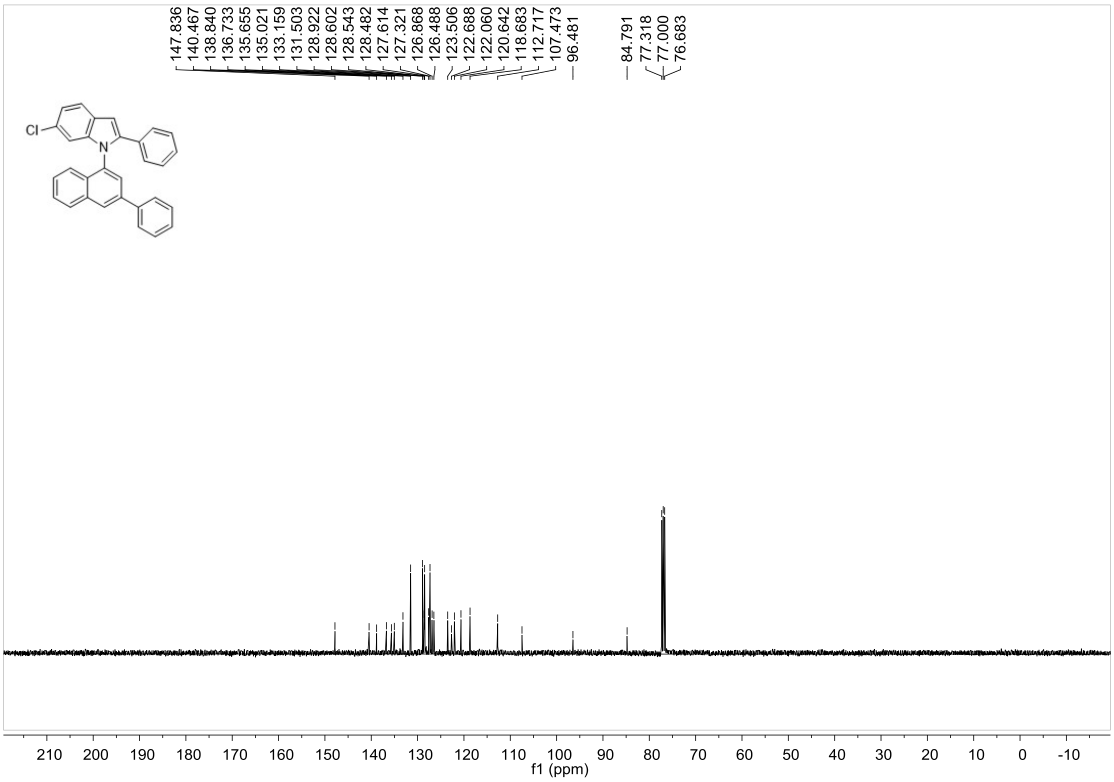

^{1}H NMR Spectrum of **54c**

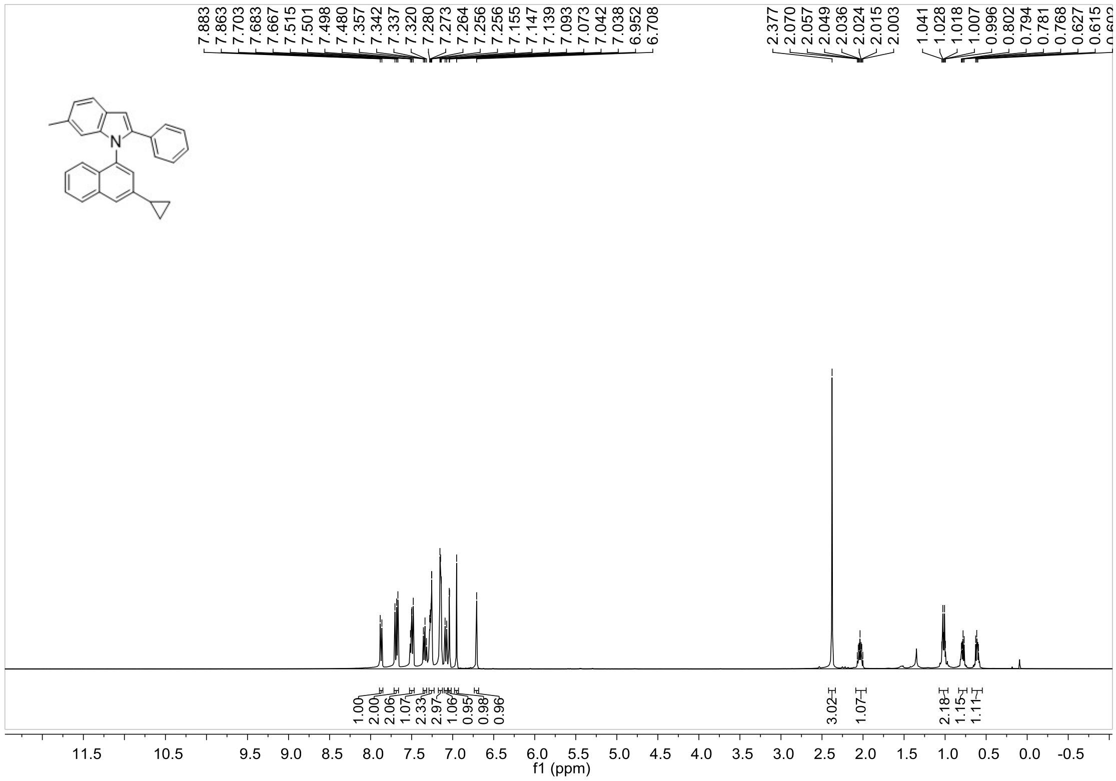

^{13}C NMR Spectrum of **54c**

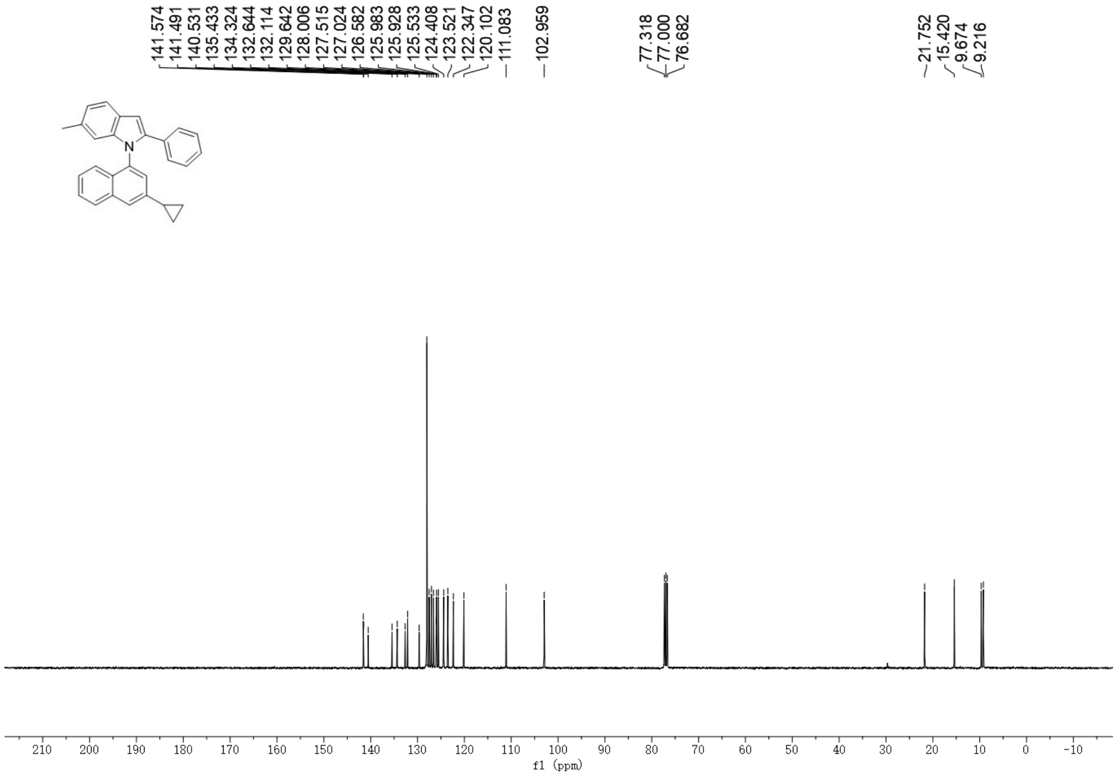

^{1}H NMR Spectrum of **55c**

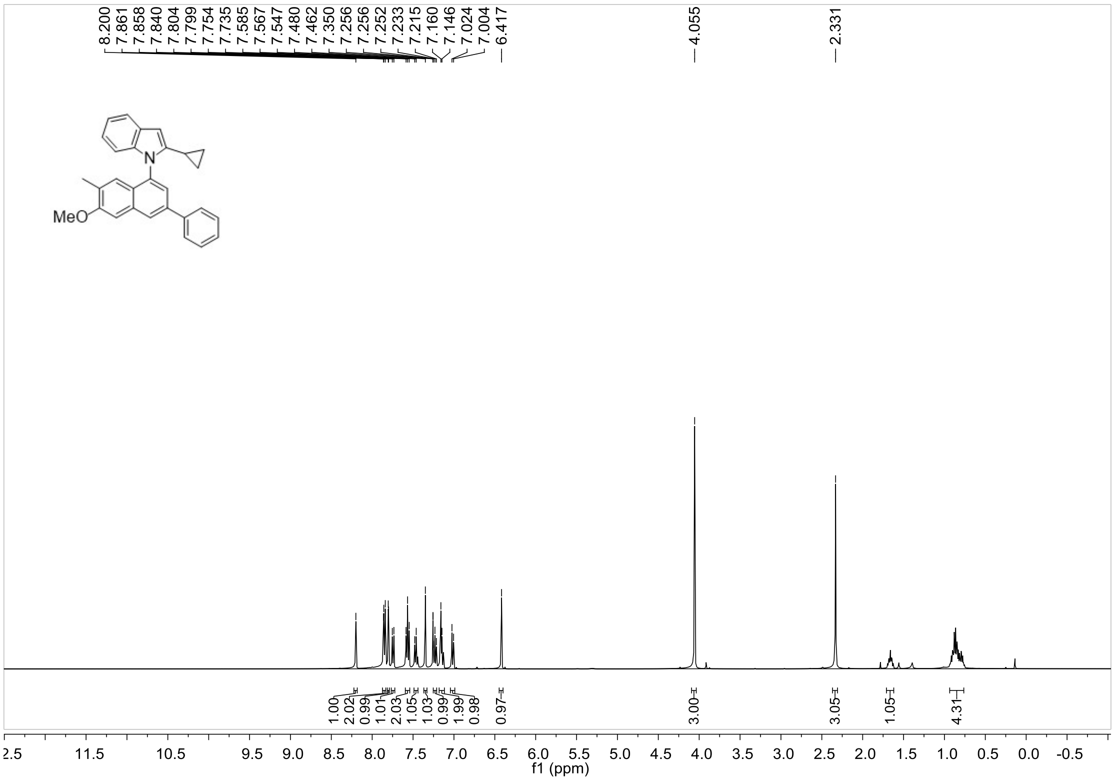

^{13}C NMR Spectrum of **55c**

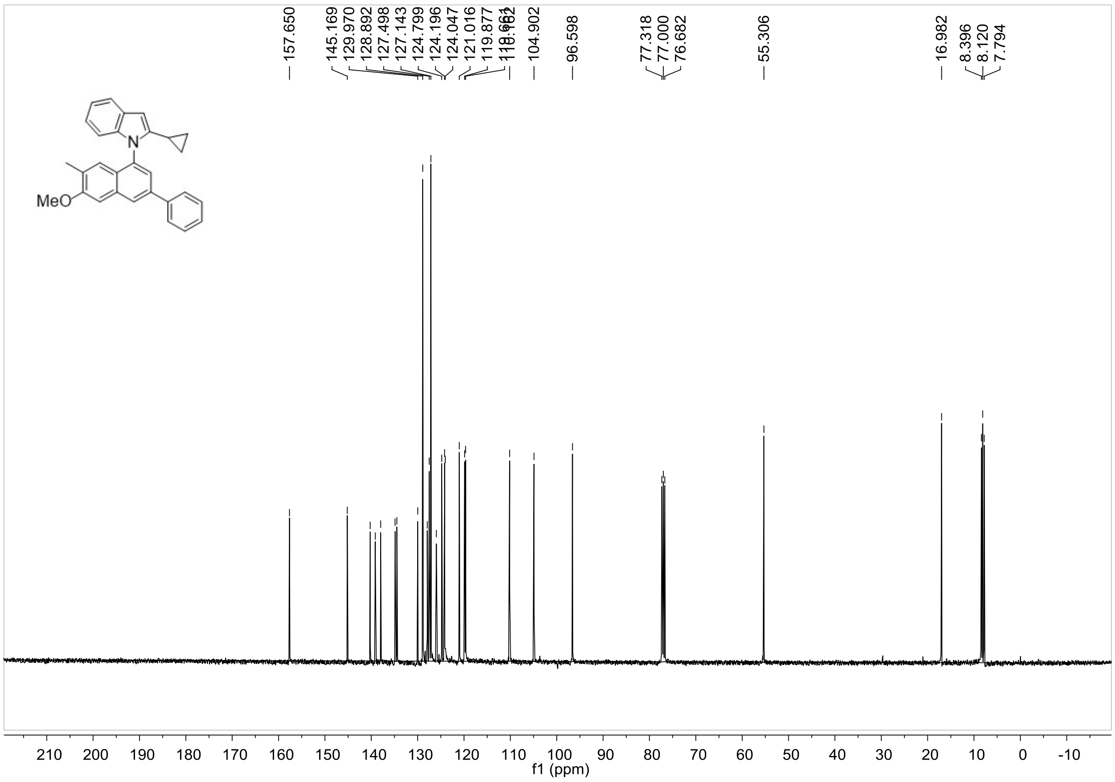

^{1}H NMR Spectrum of **56c**

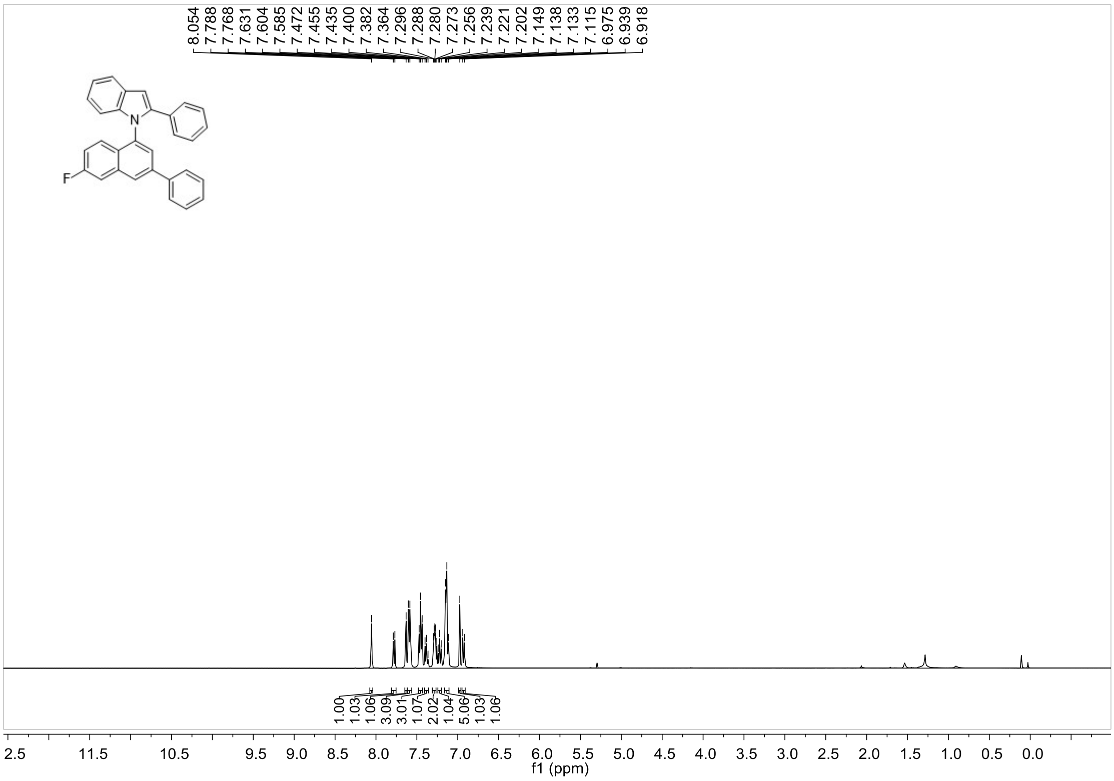

^{13}C NMR Spectrum of **56c**

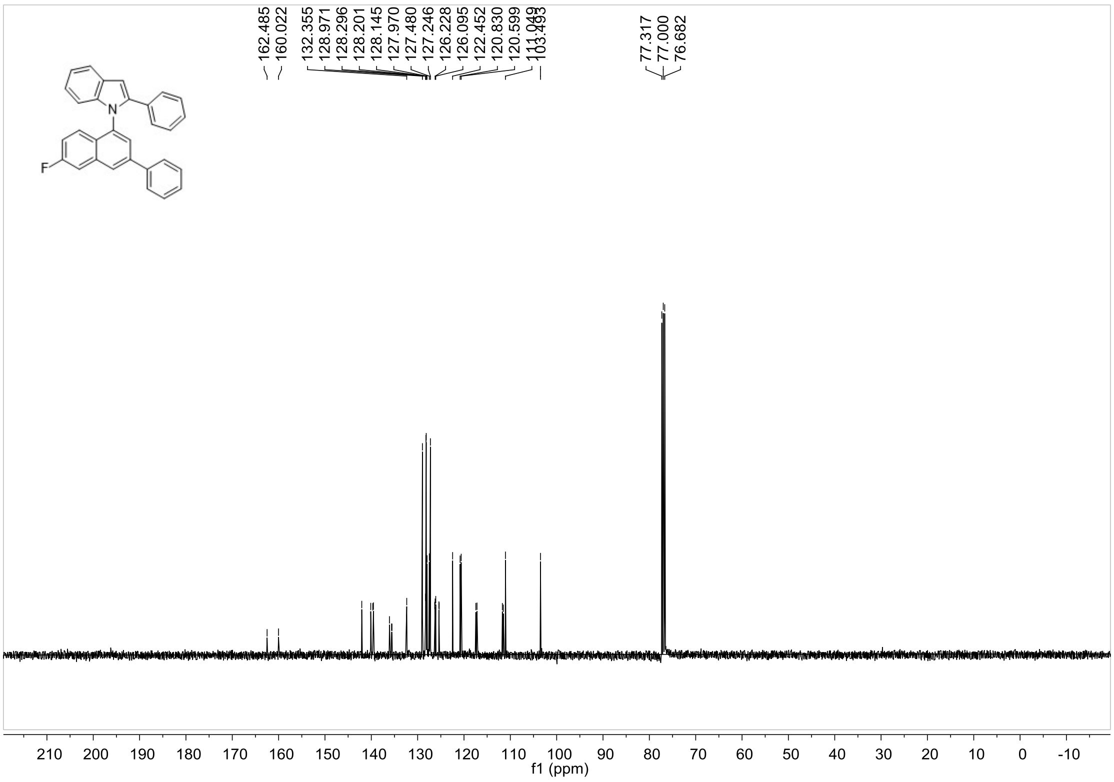

^{19}F NMR Spectrum of **56c**

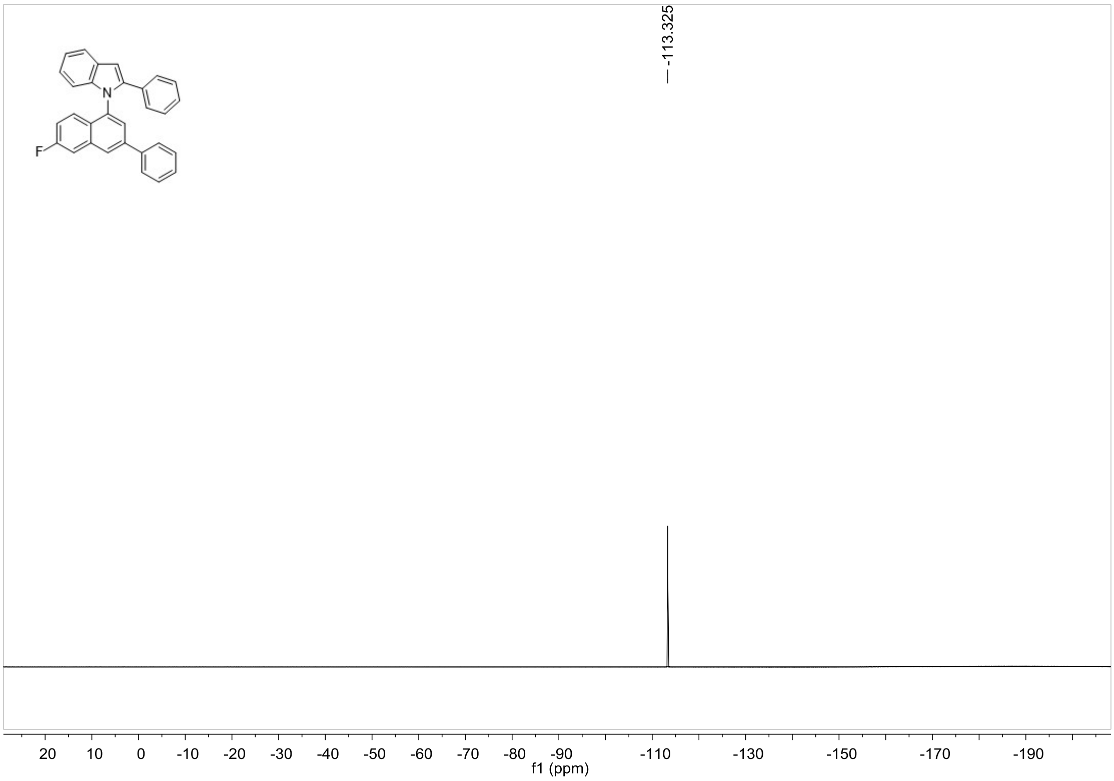

^{1}H NMR Spectrum of **57c**

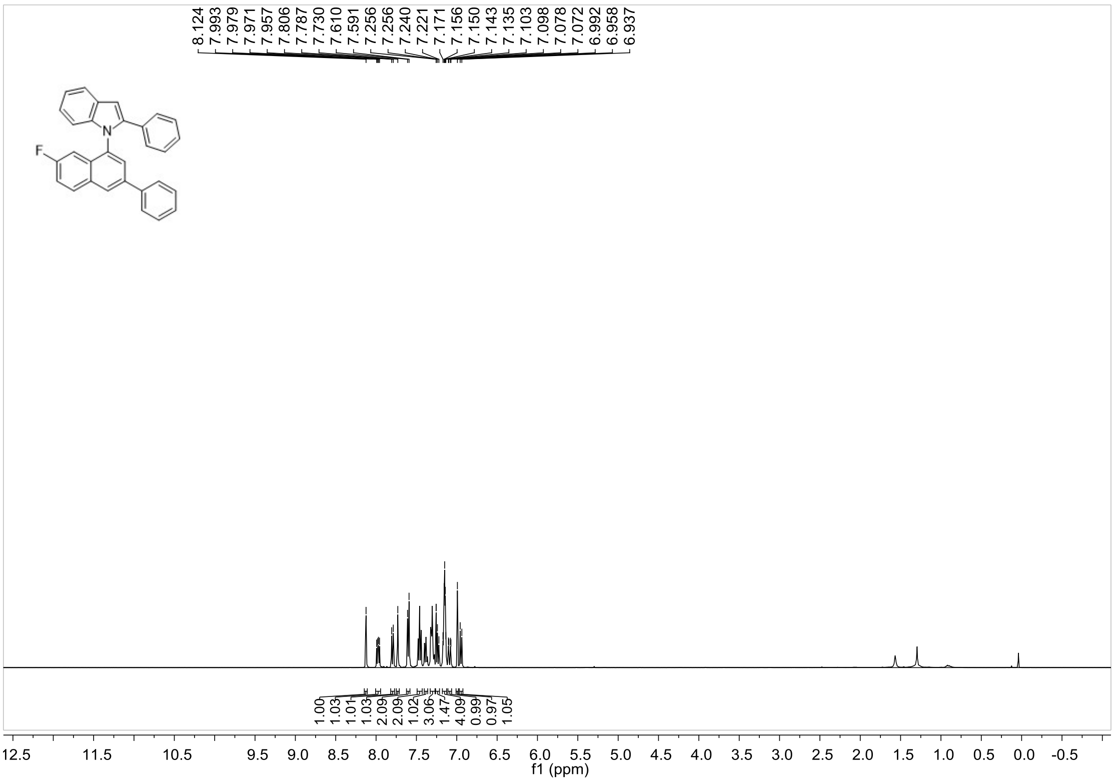

^{13}C NMR Spectrum of **57c**

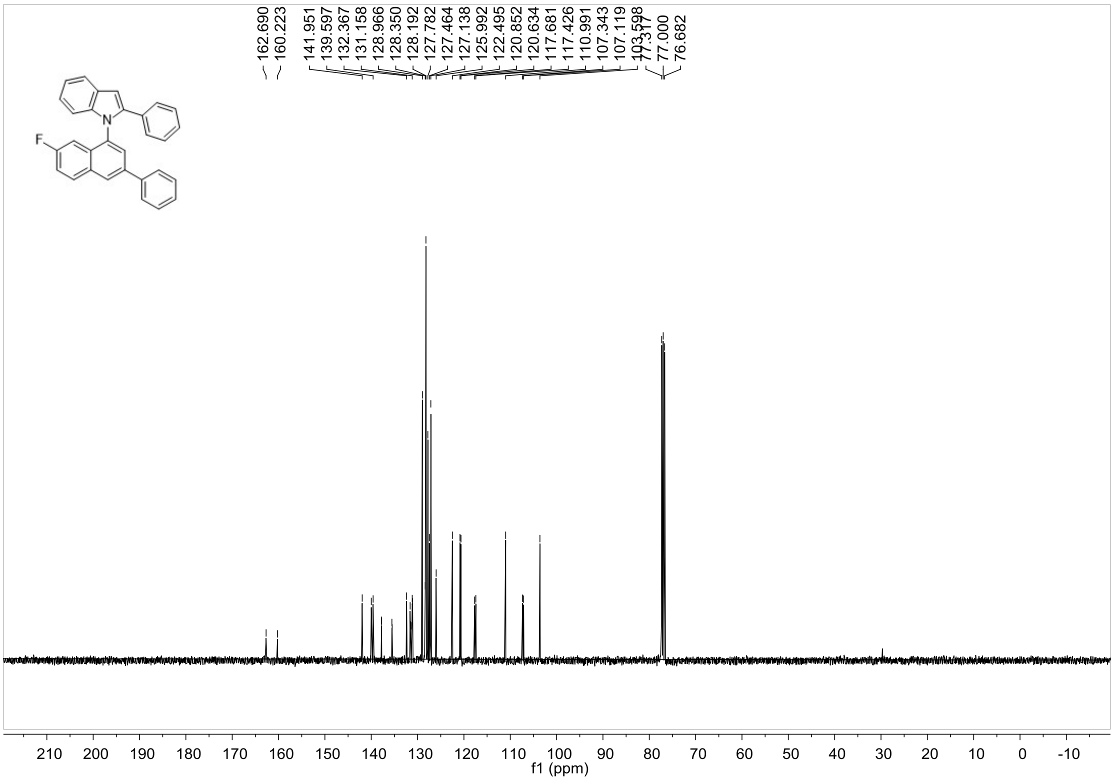

^{19}F NMR Spectrum of **57c**

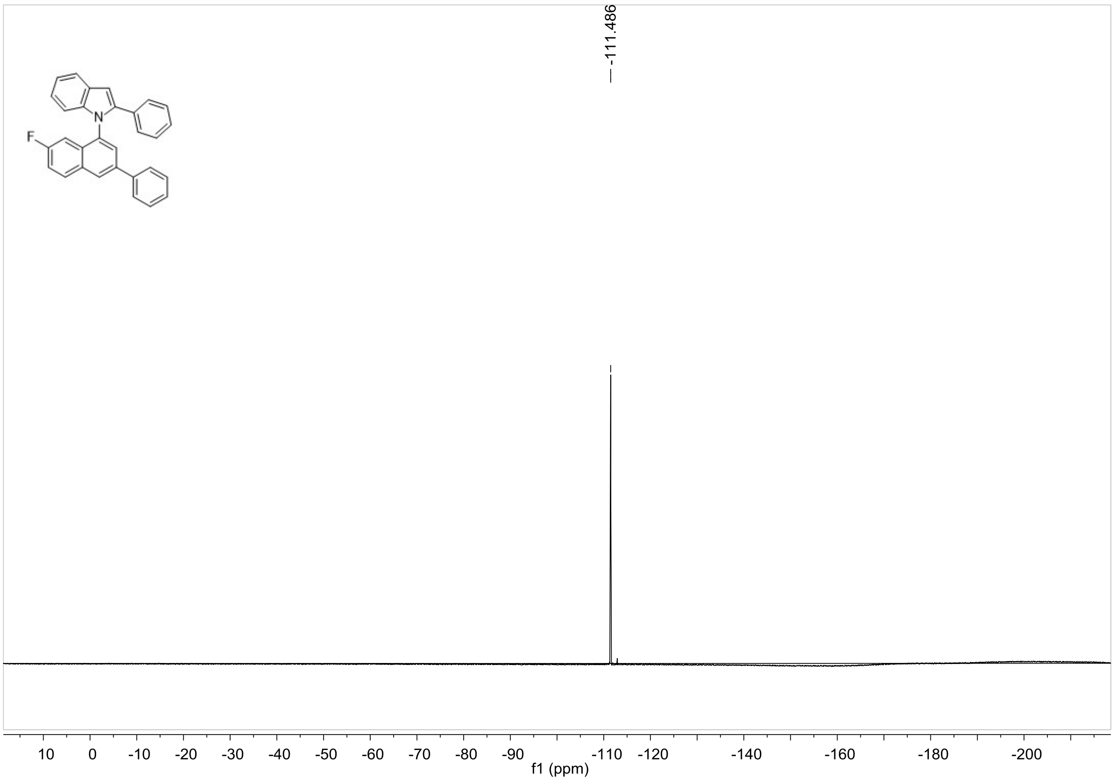

^{1}H NMR Spectrum of **58c**

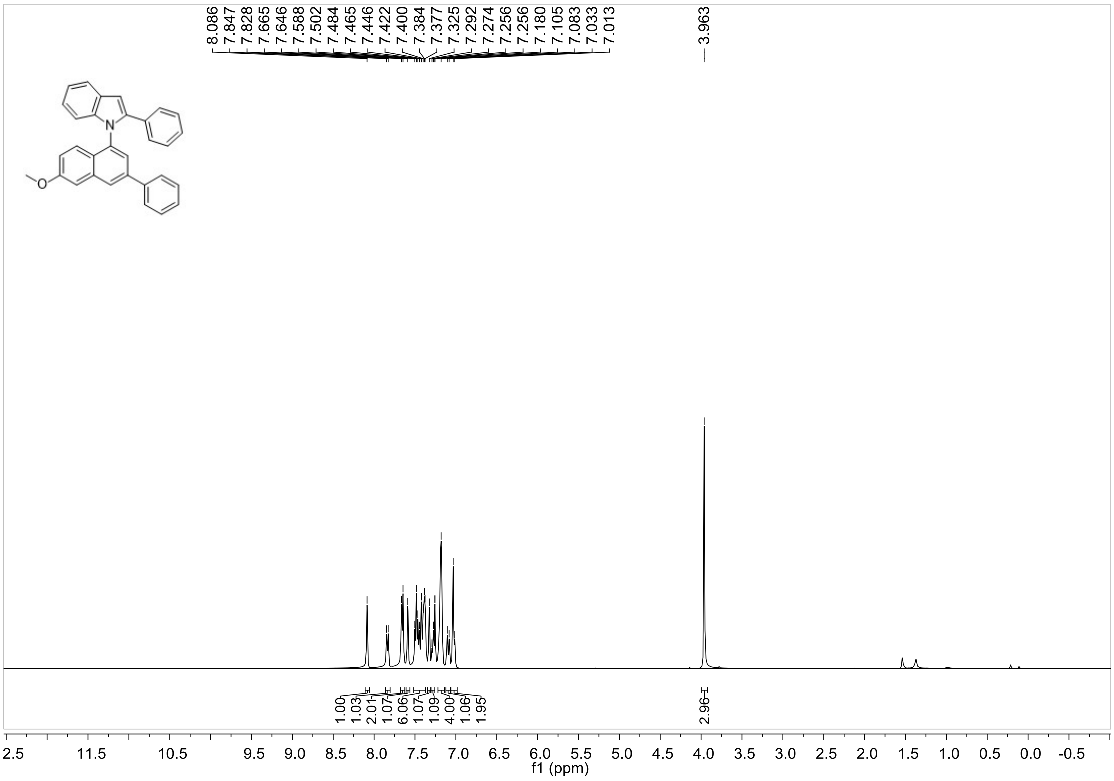

^{13}C NMR Spectrum of **58c**

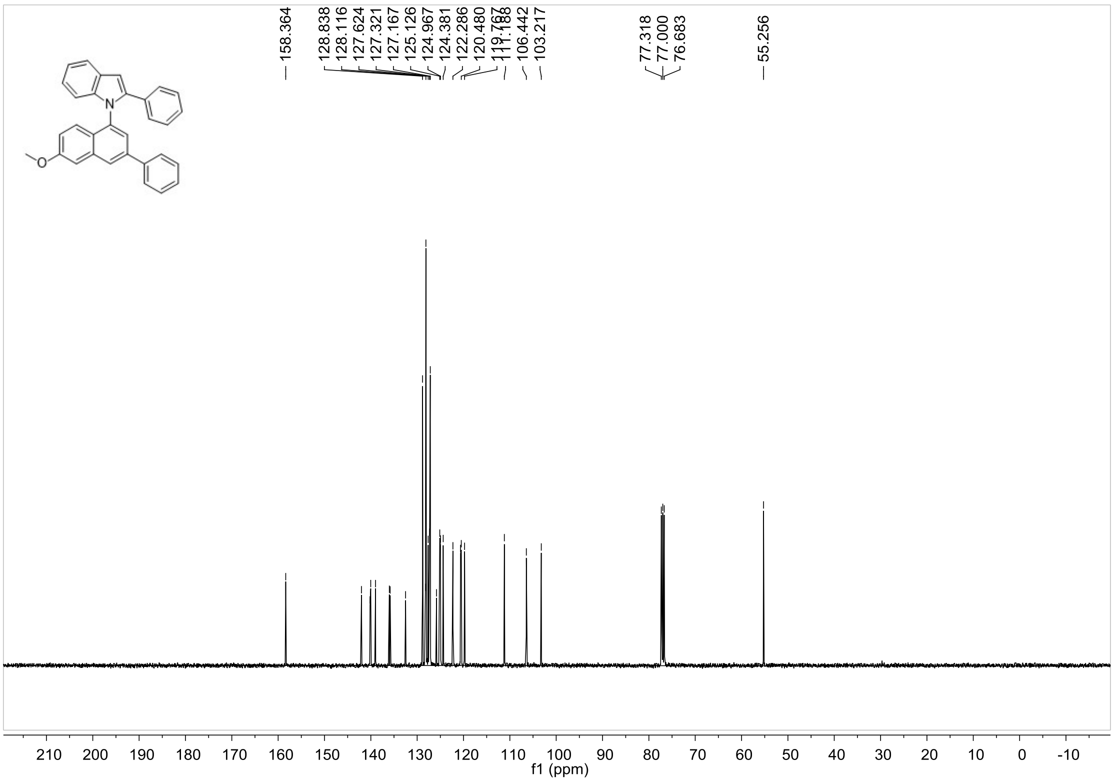

^{1}H NMR Spectrum of **59c**

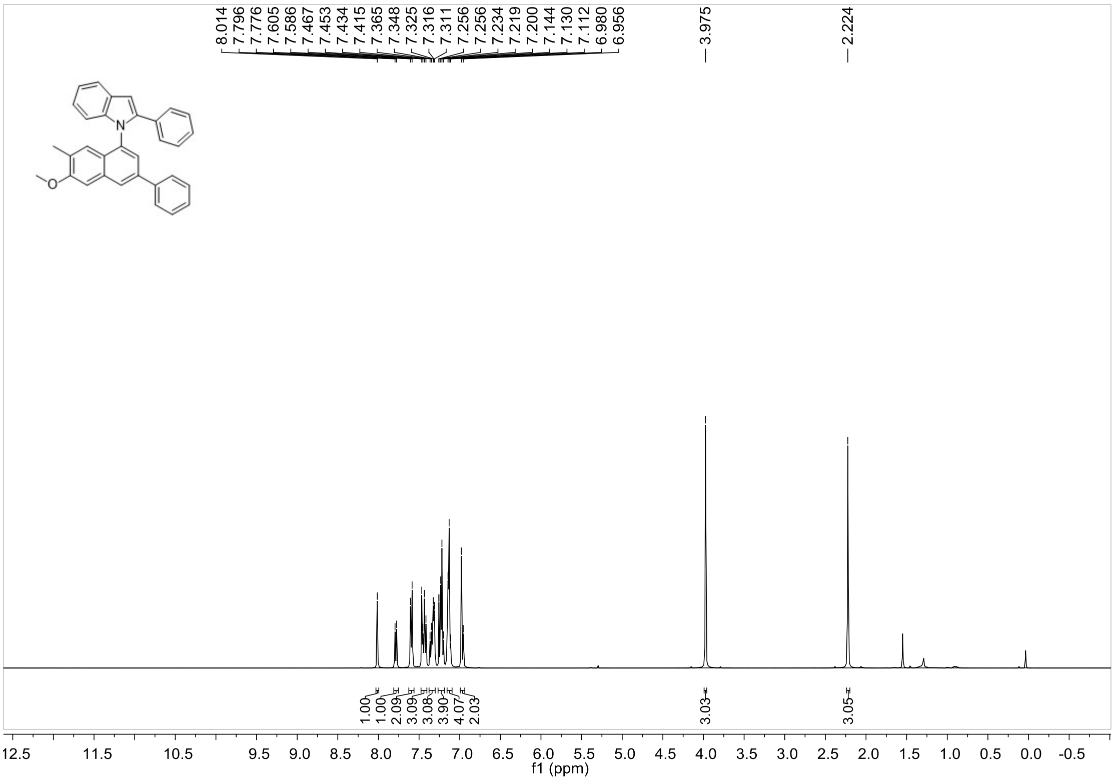

^{13}C NMR Spectrum of **59c**

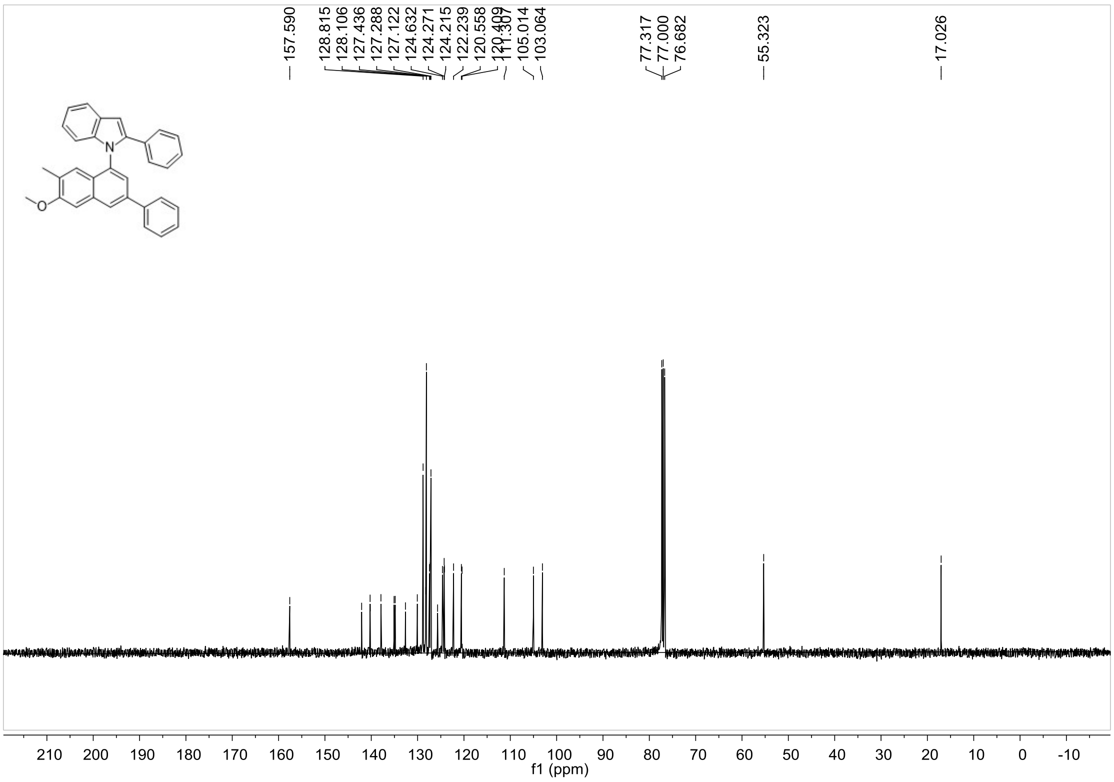

^{1}H NMR Spectrum of **60c**

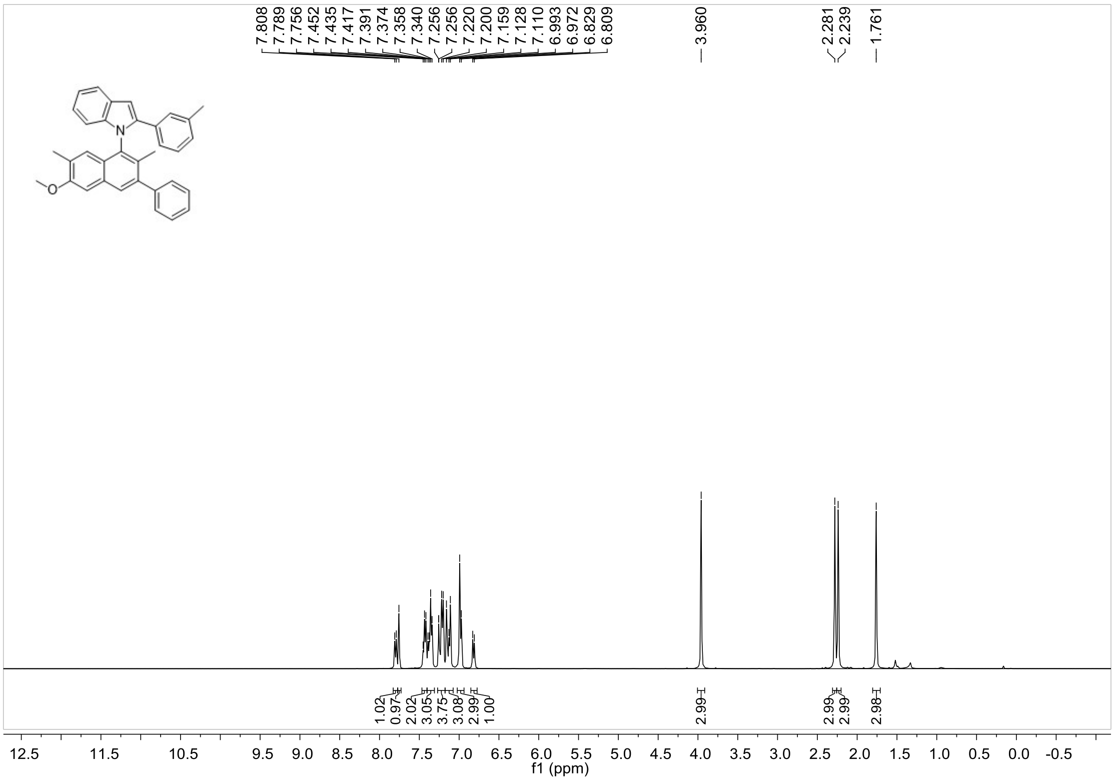

^{13}C NMR Spectrum of **60c**

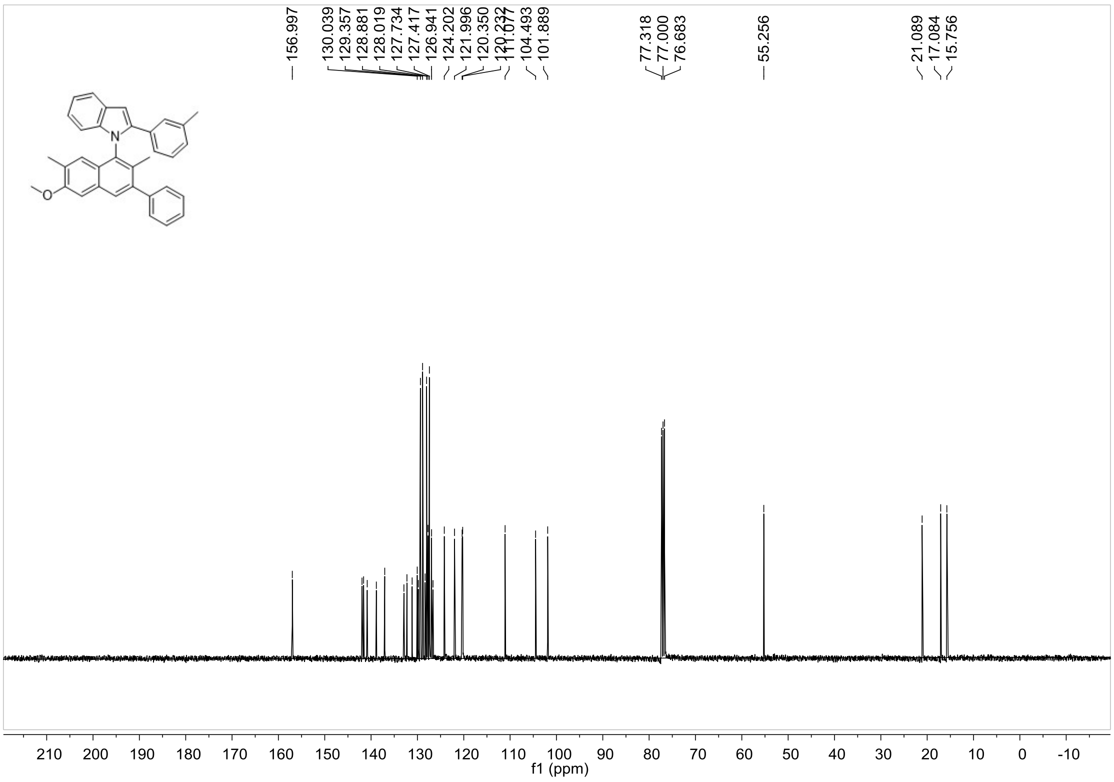

^{1}H NMR Spectrum of **61c**

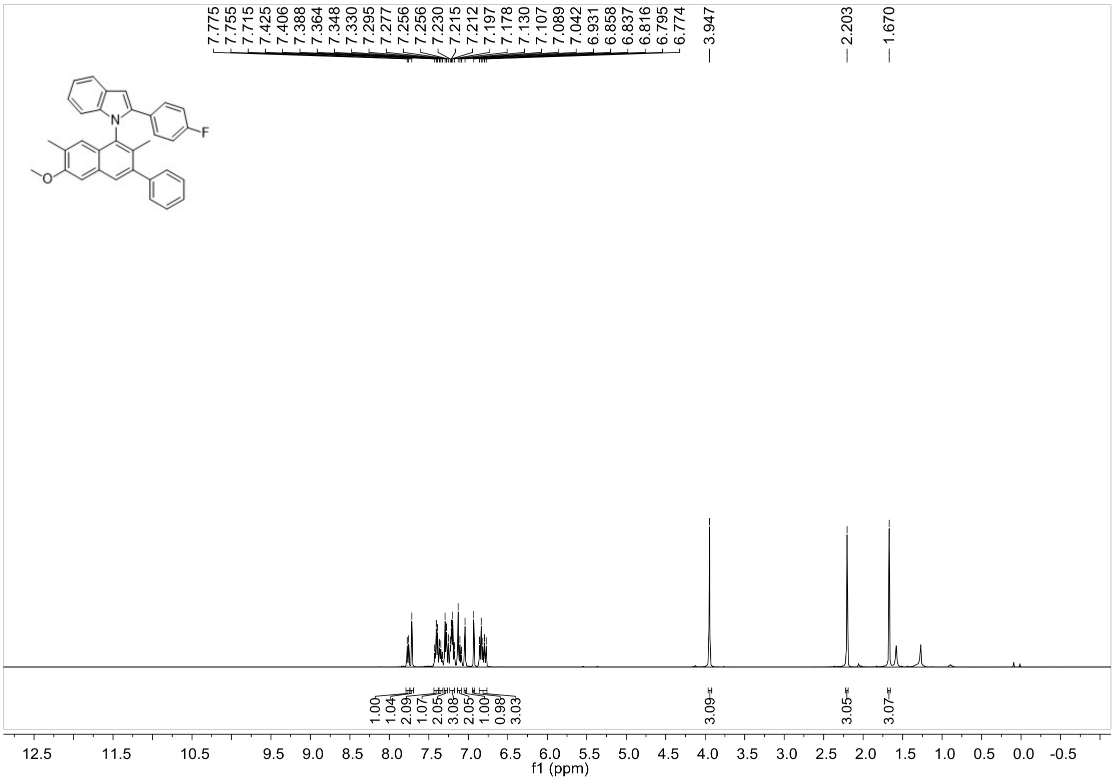

^{13}C NMR Spectrum of **61c**

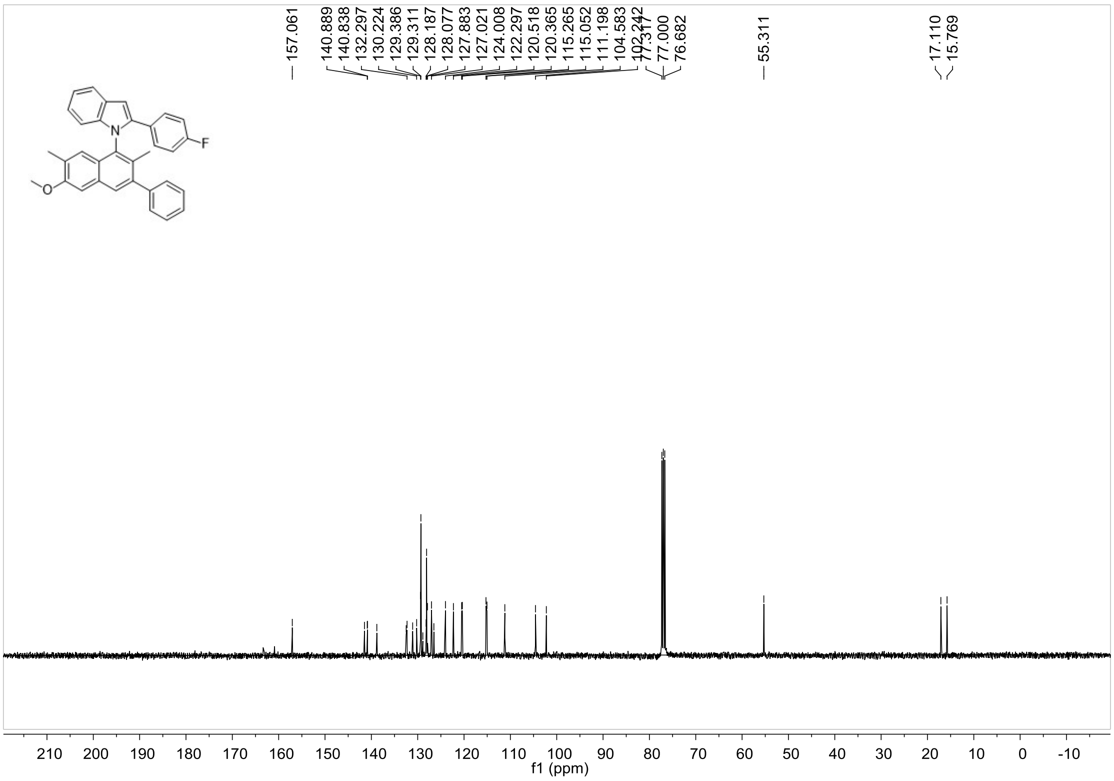

^{19}F NMR Spectrum of **61c**

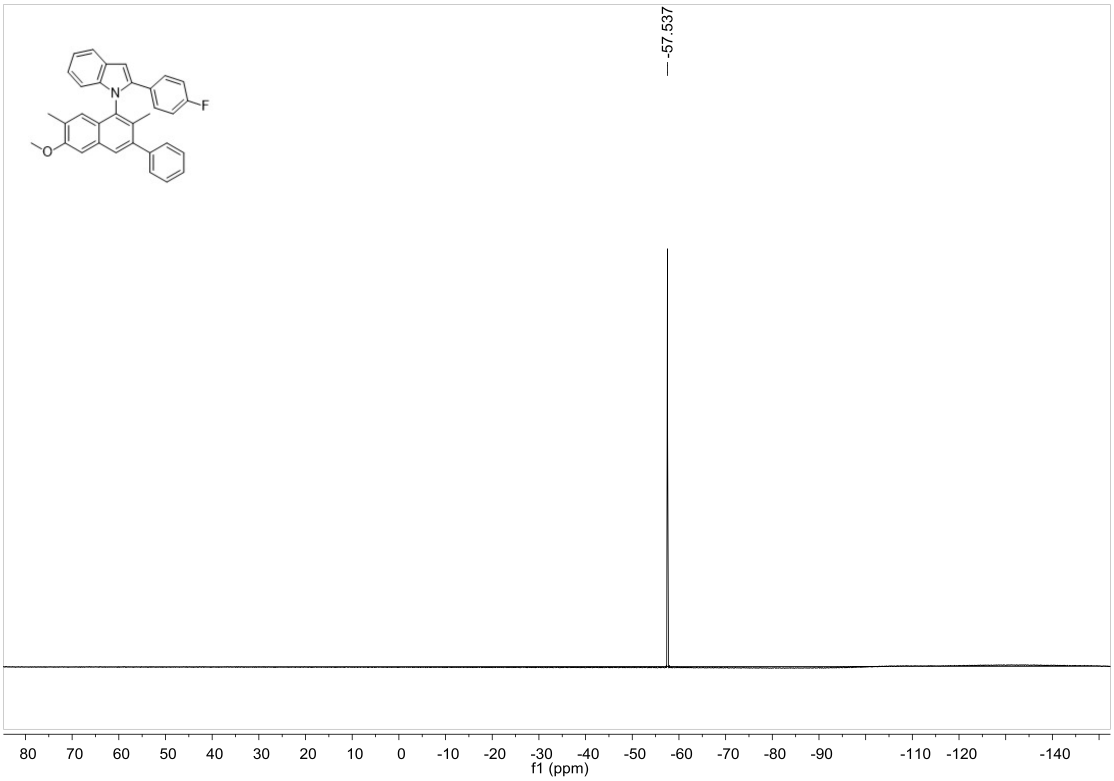

^{1}H NMR Spectrum of **62c**

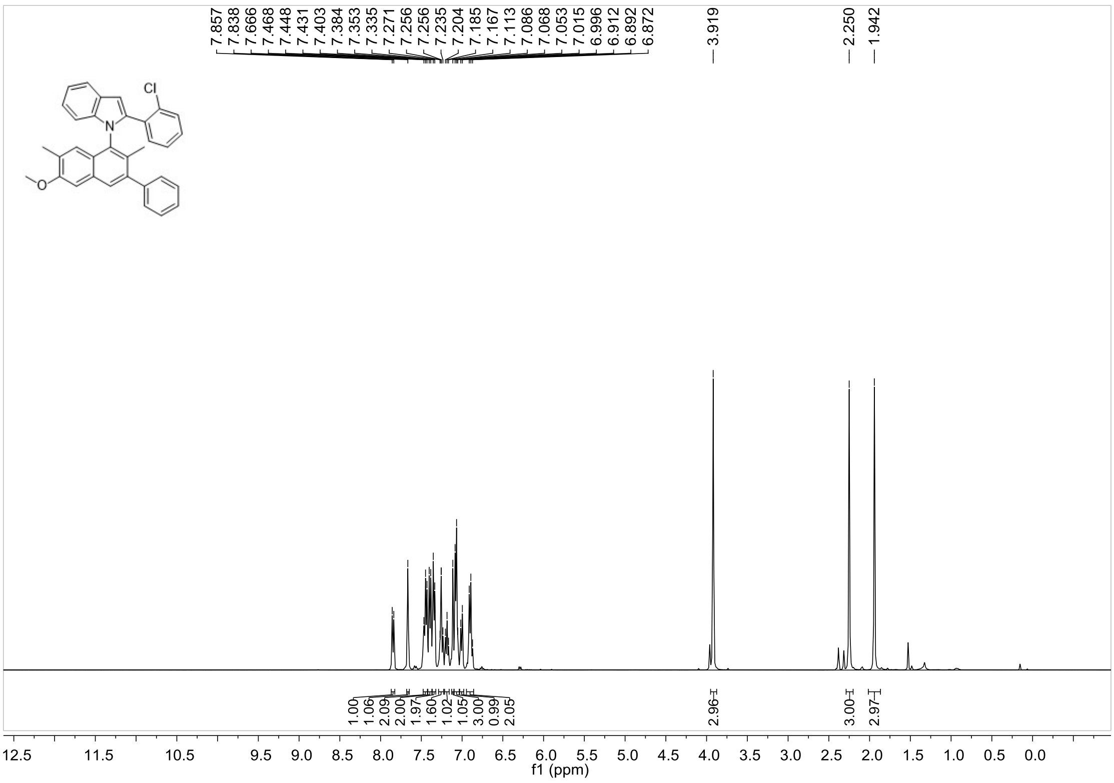

^{13}C NMR Spectrum of **62c**

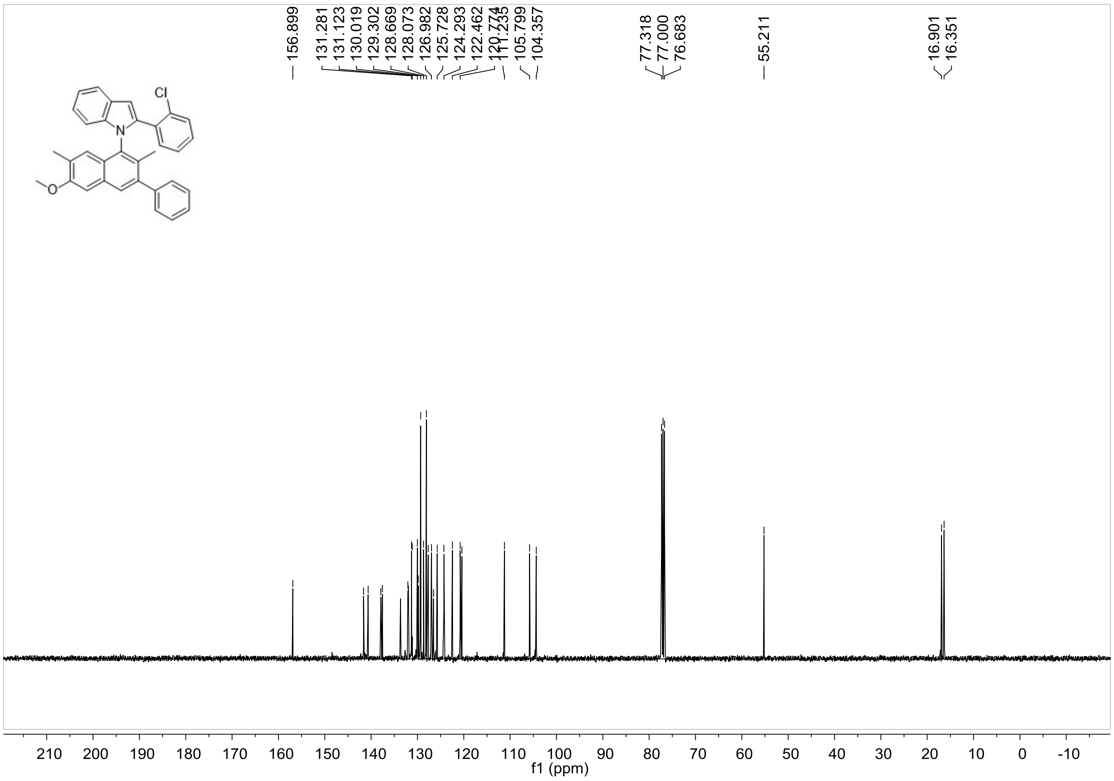

^{1}H NMR Spectrum of **63c**

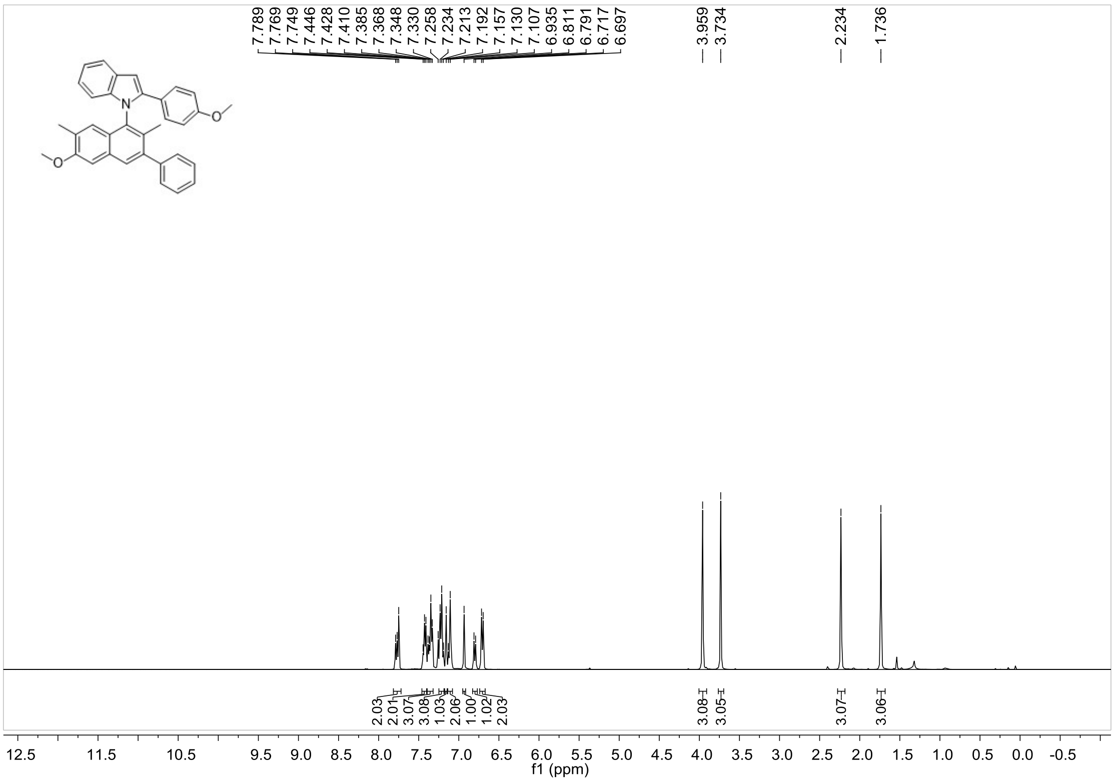

^{13}C NMR Spectrum of **63c**

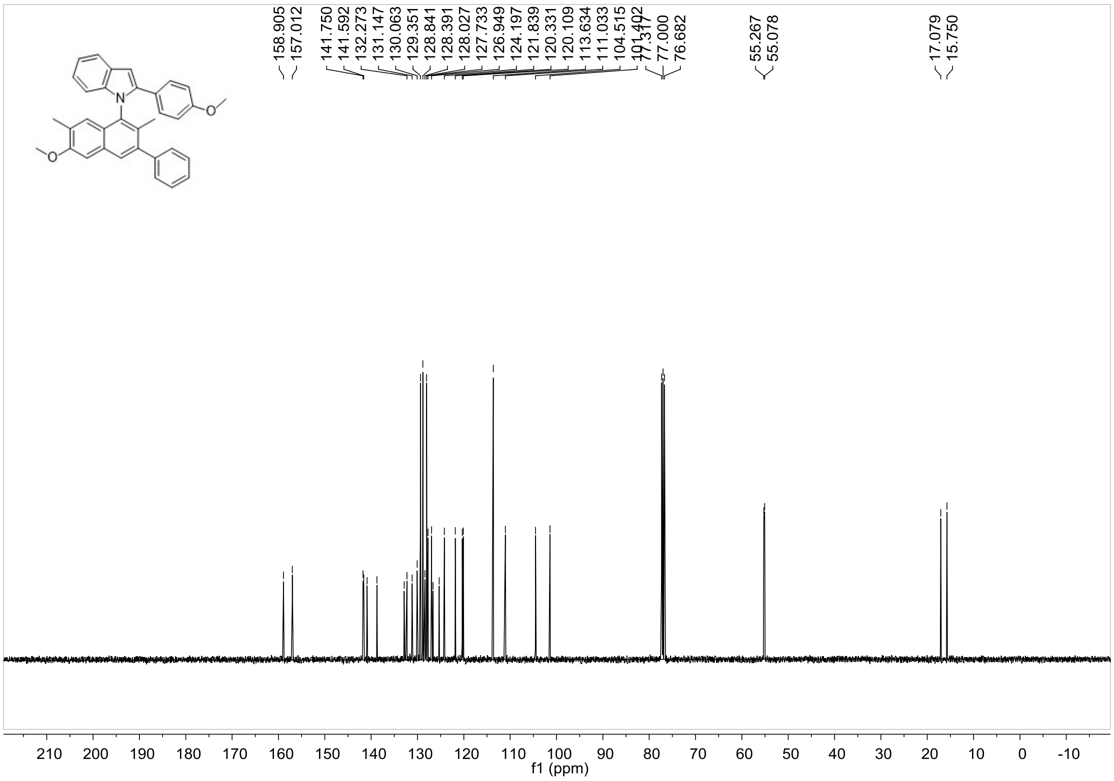

^{1}H NMR Spectrum of **64c**

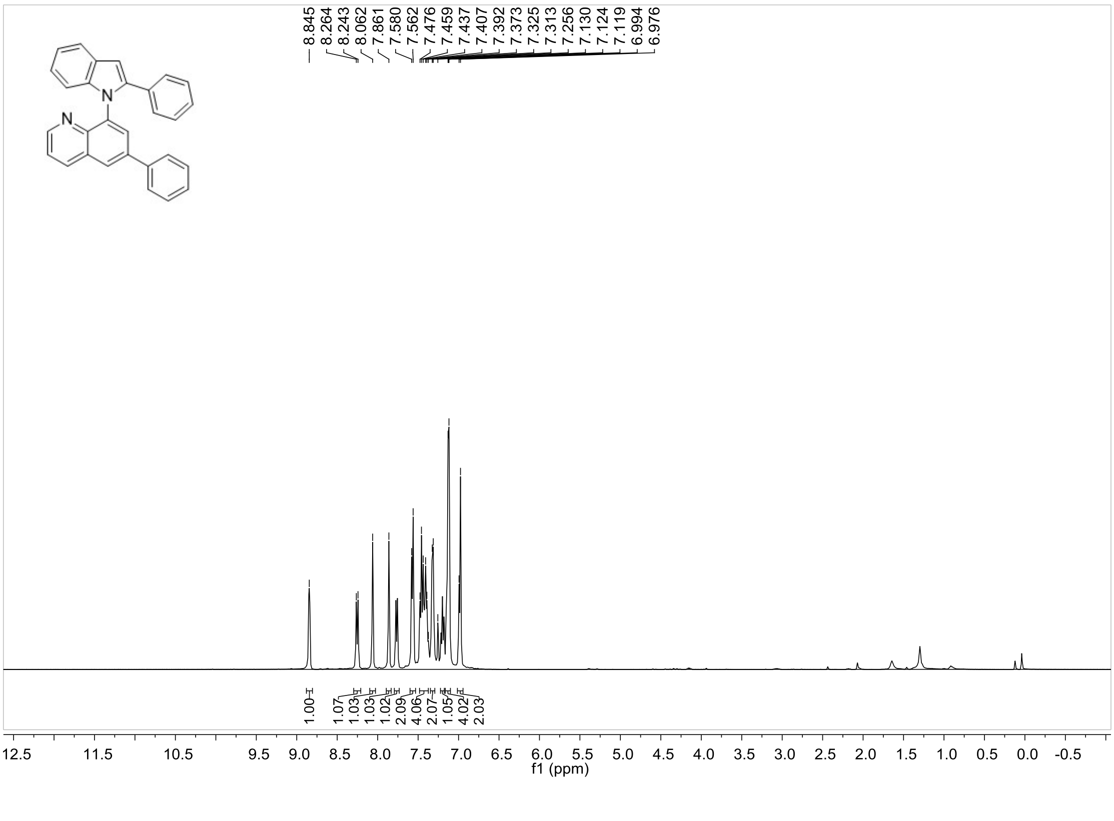

^{13}C NMR Spectrum of **64c**

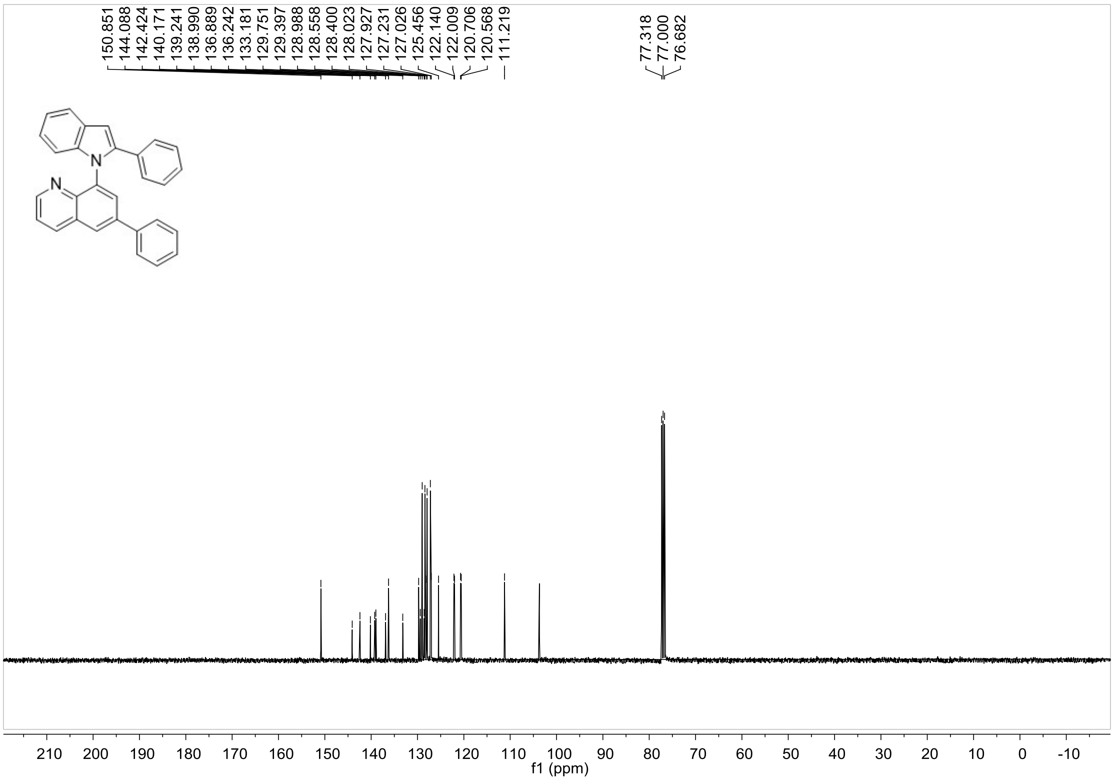

^{1}H NMR Spectrum of **65c**

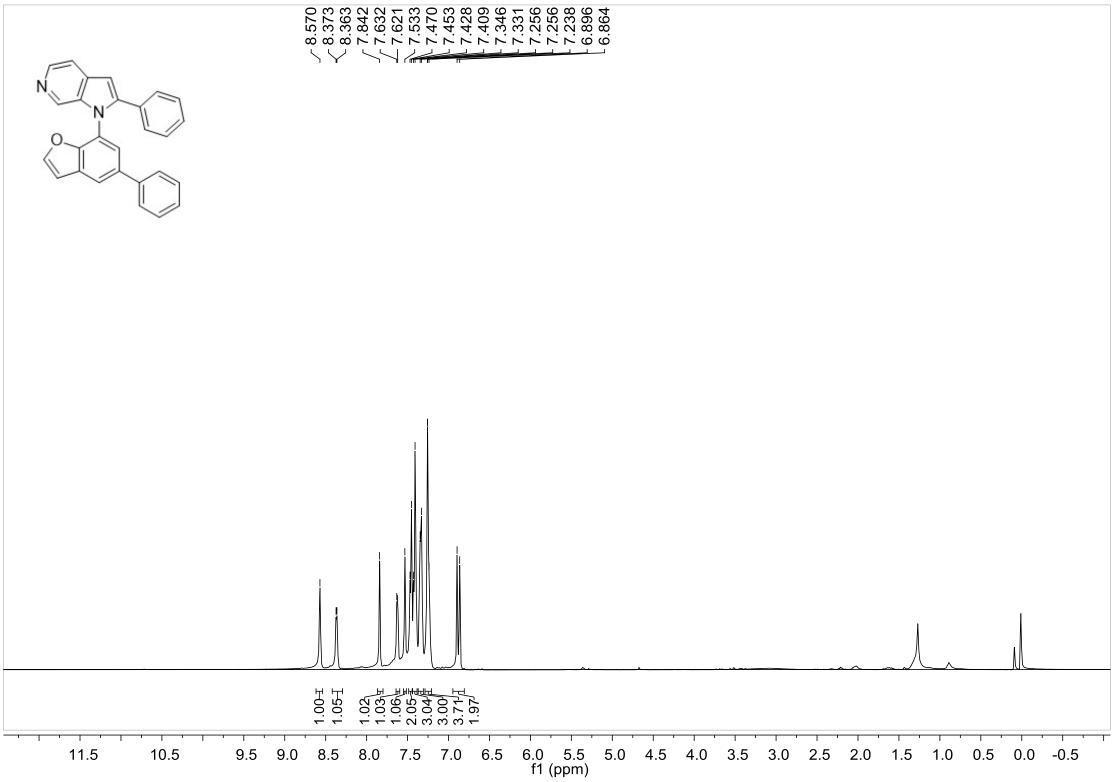

^{13}C NMR Spectrum of **65c**

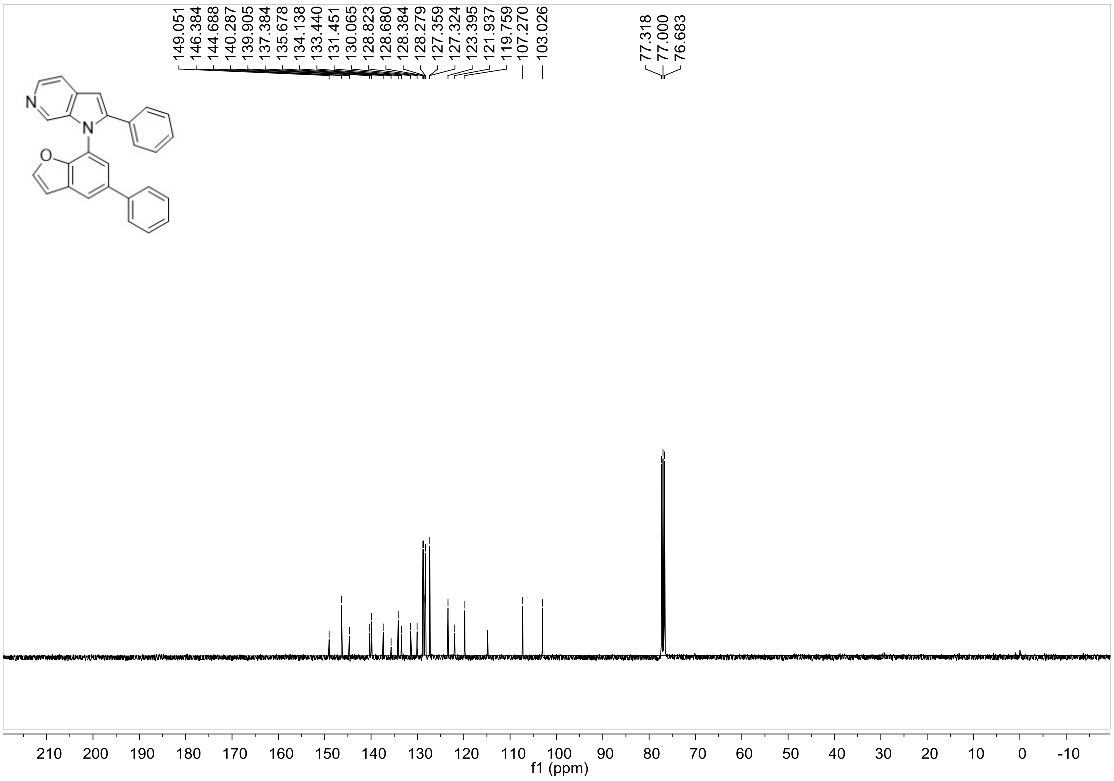

^{1}H NMR Spectrum of **66c**

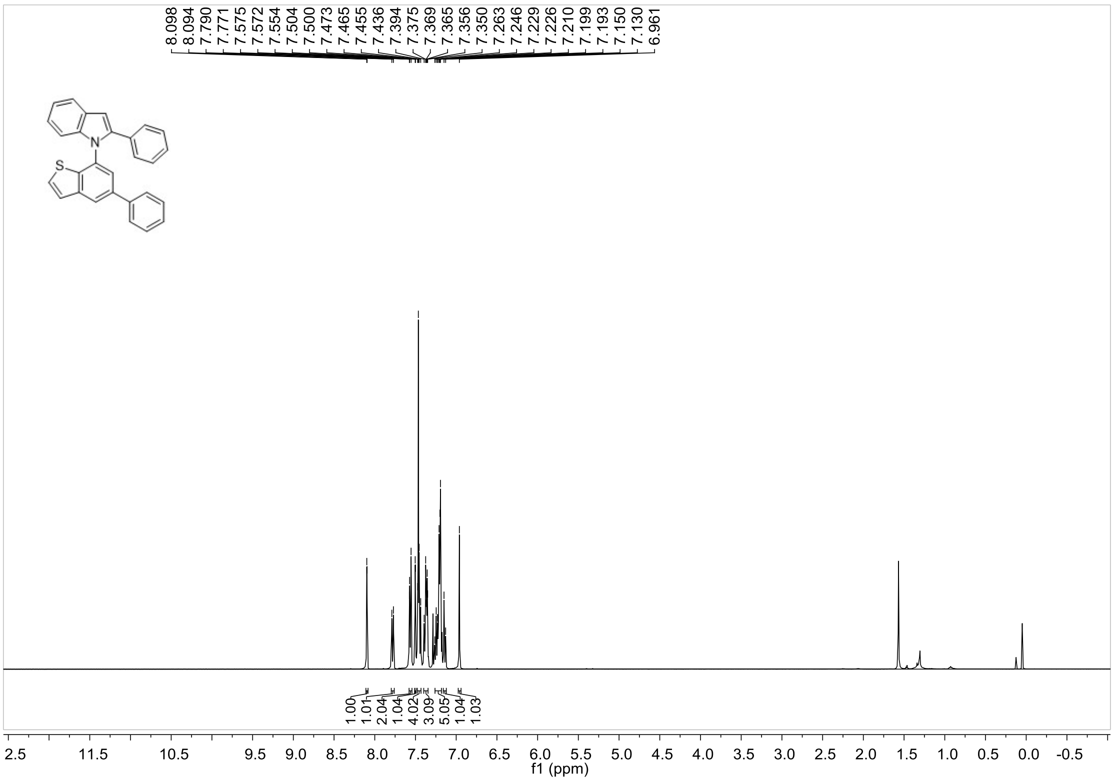

^{13}C NMR Spectrum of **66c**

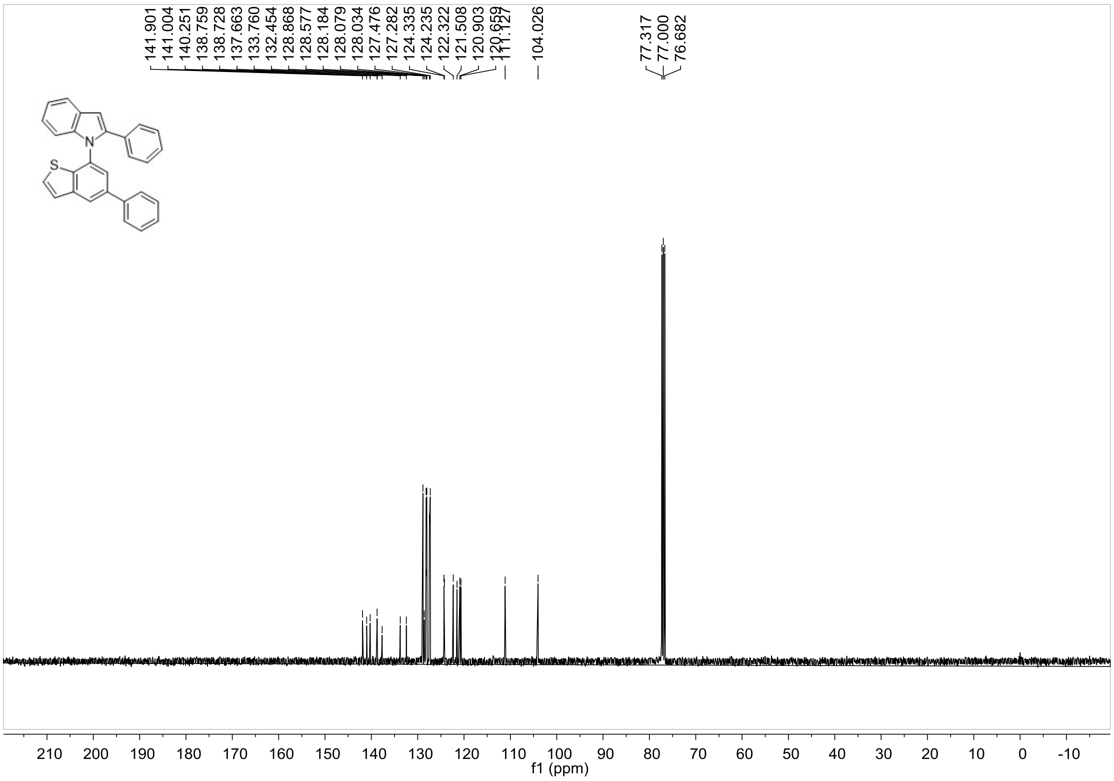

^{1}H NMR Spectrum of **67c**

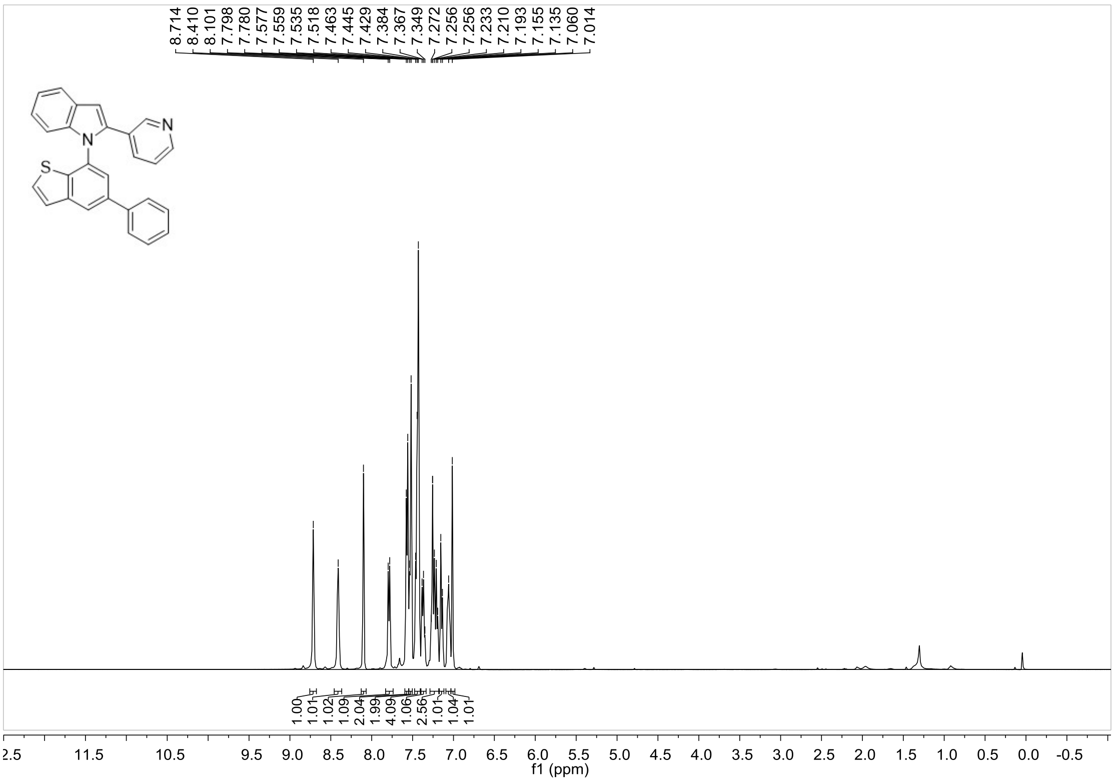

^{13}C NMR Spectrum of **67c**

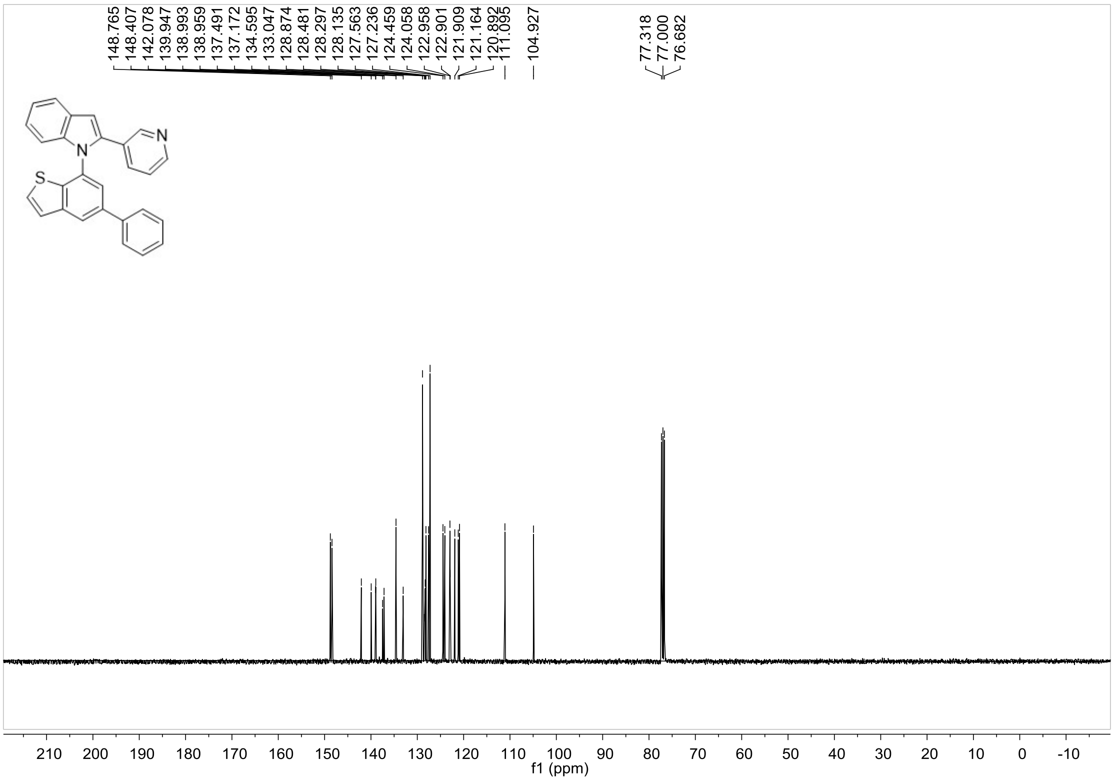

^{1}H NMR Spectrum of **68c**

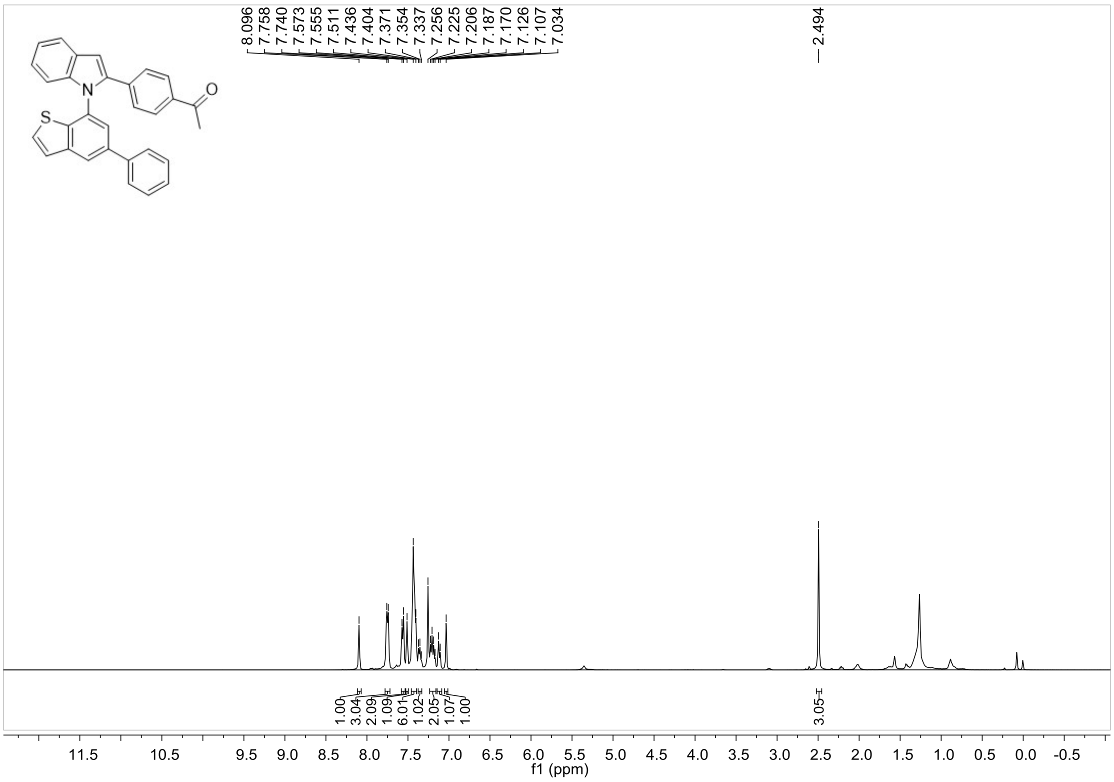

^{13}C NMR Spectrum of **68c**

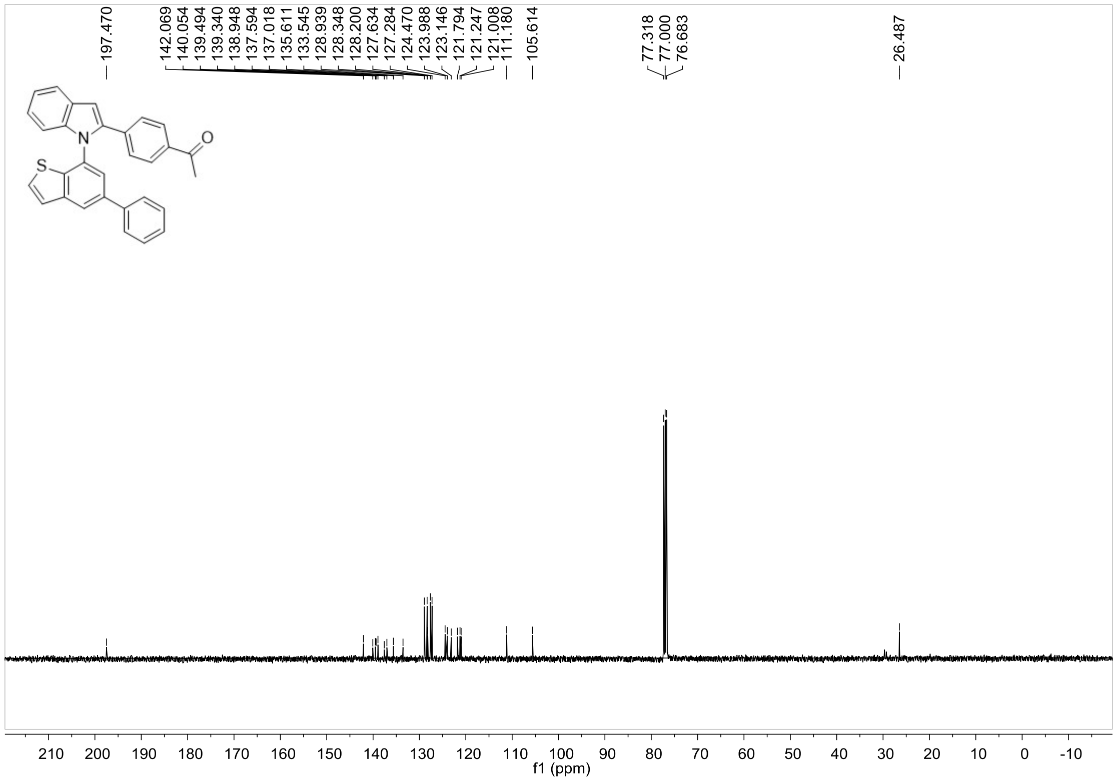

^{1}H NMR Spectrum of **69c**

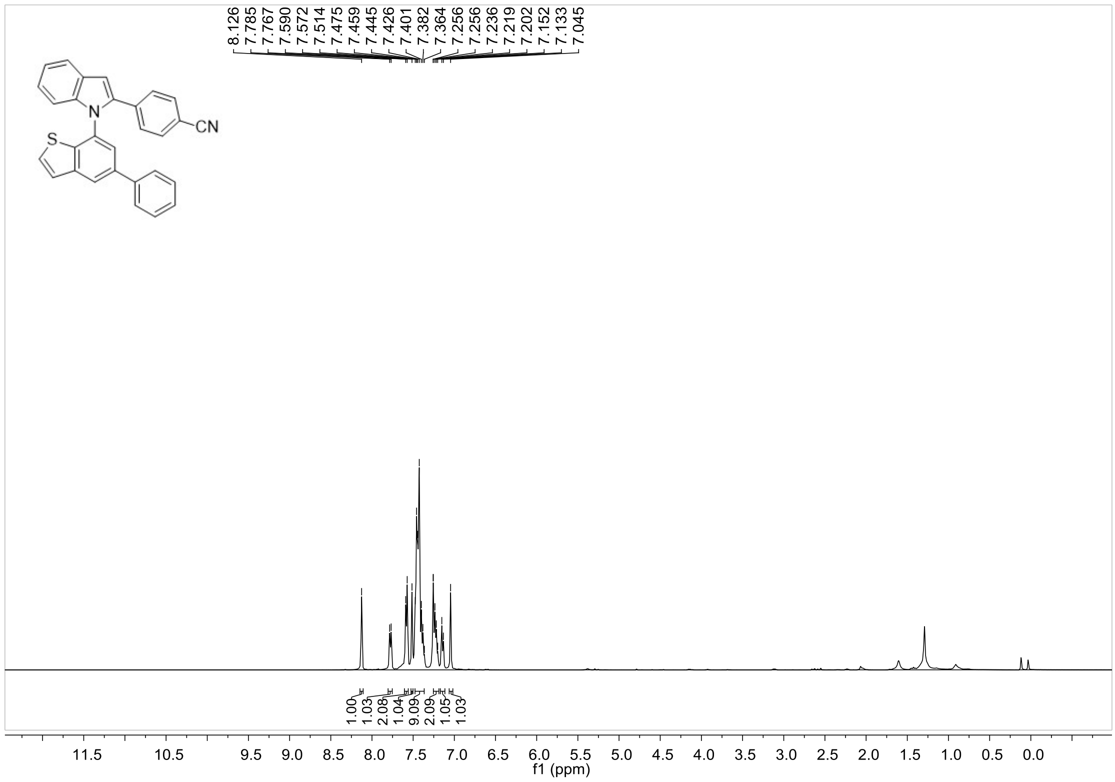

^{13}C NMR Spectrum of **69c**

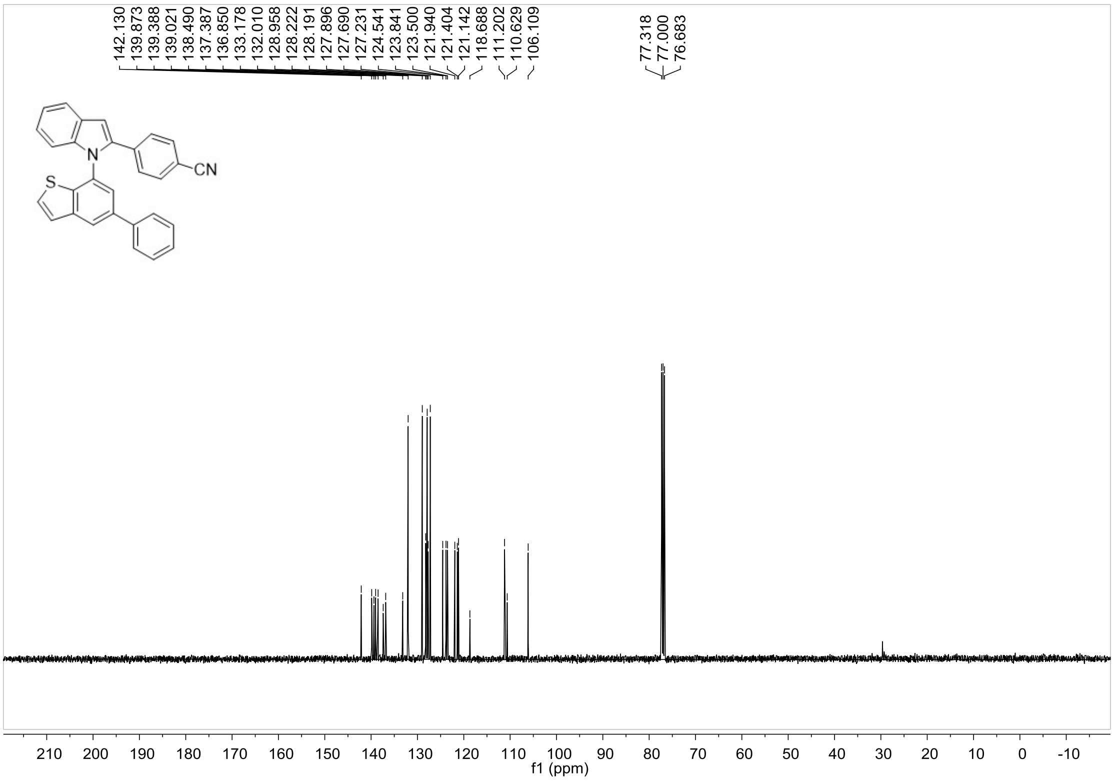

^{1}H NMR Spectrum of **70c**

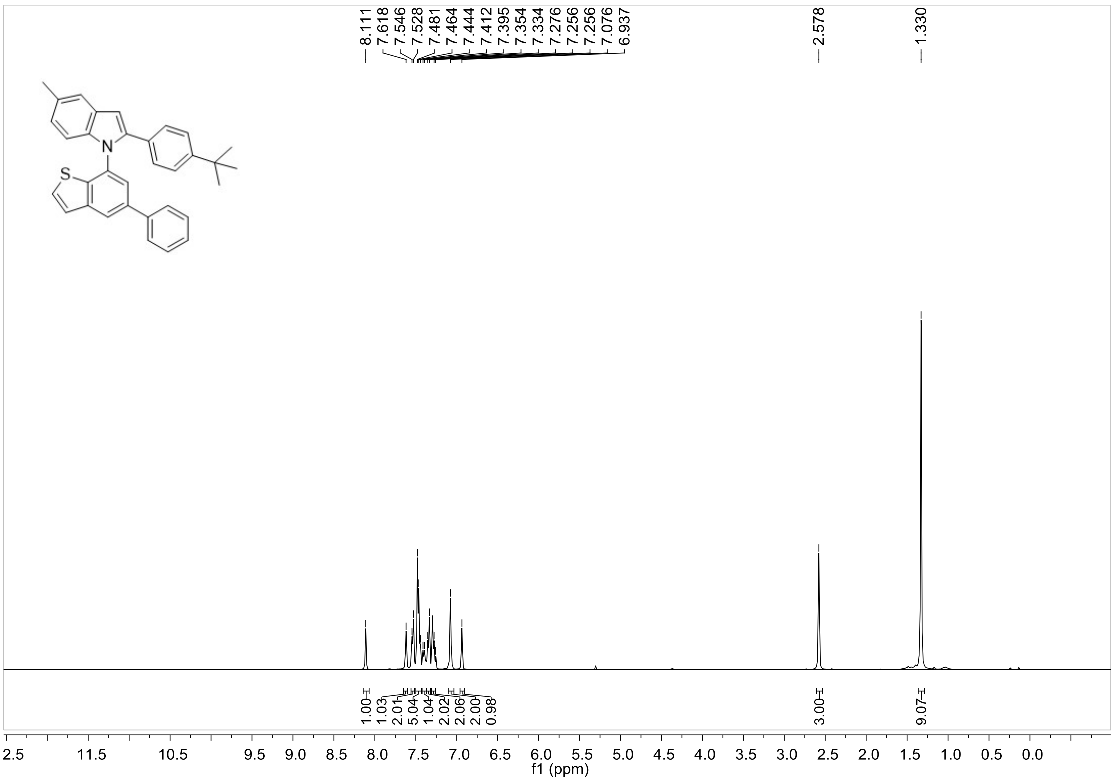

^{13}C NMR Spectrum of **70c**

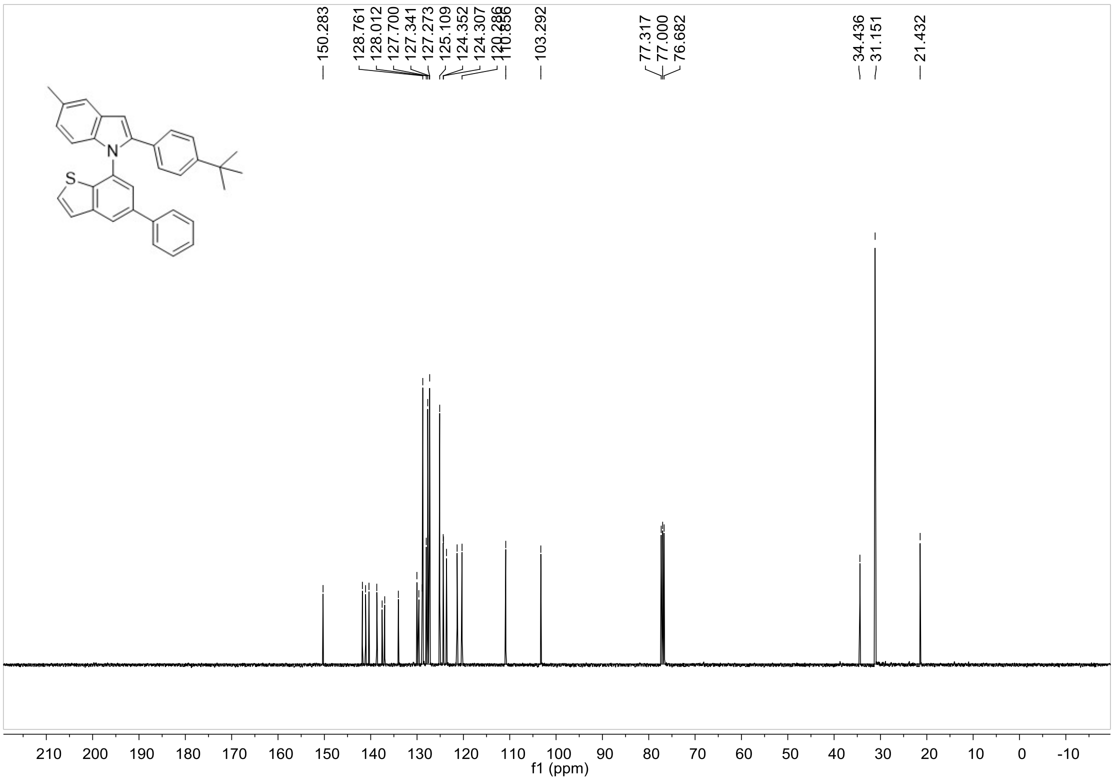

^{1}H NMR Spectrum of **71c**

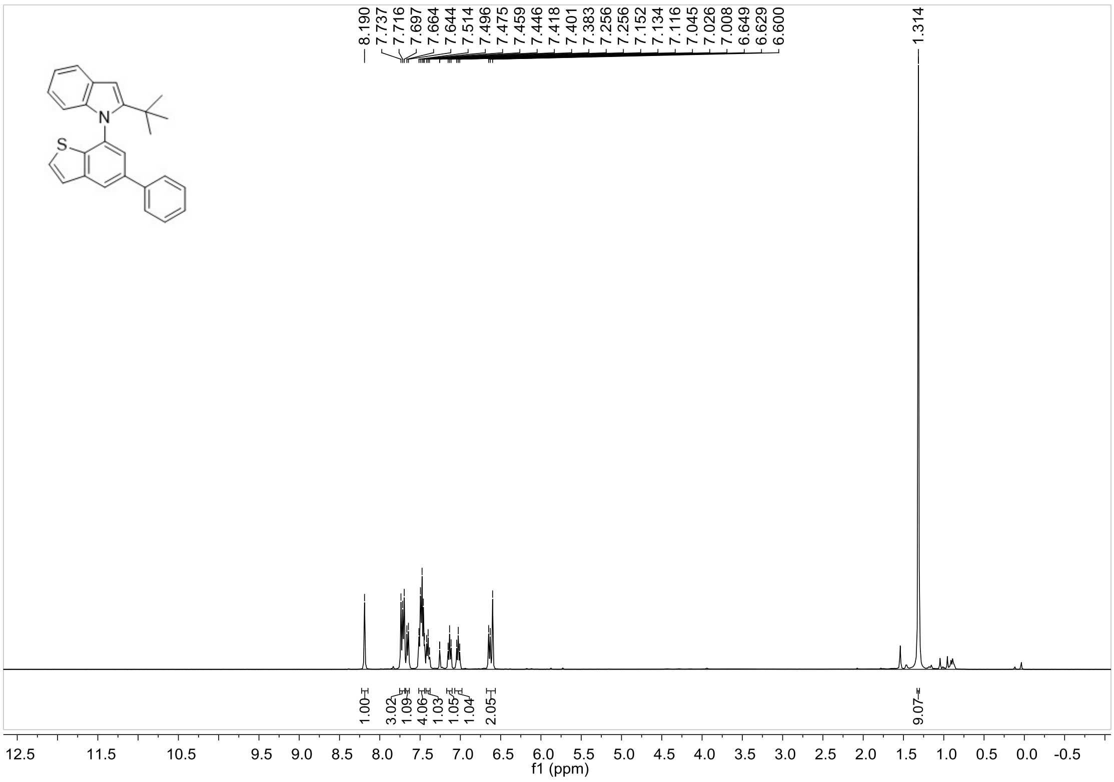

^{13}C NMR Spectrum of **71c**

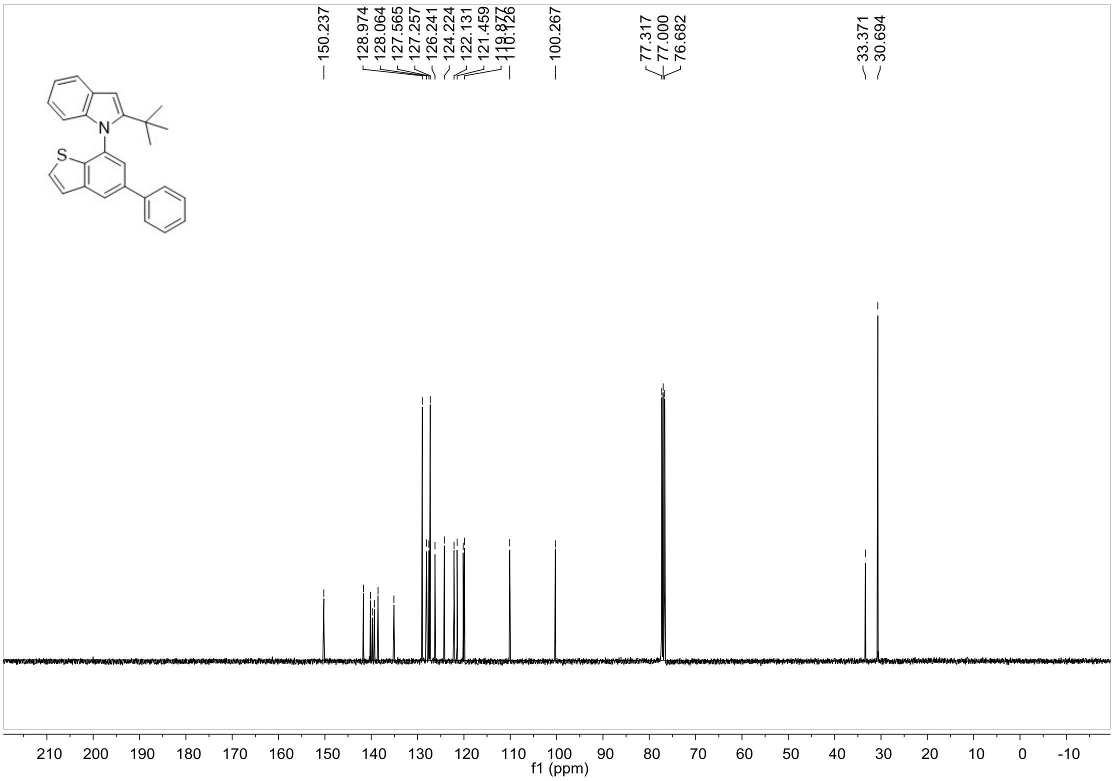

^{1}H NMR Spectrum of **72c**

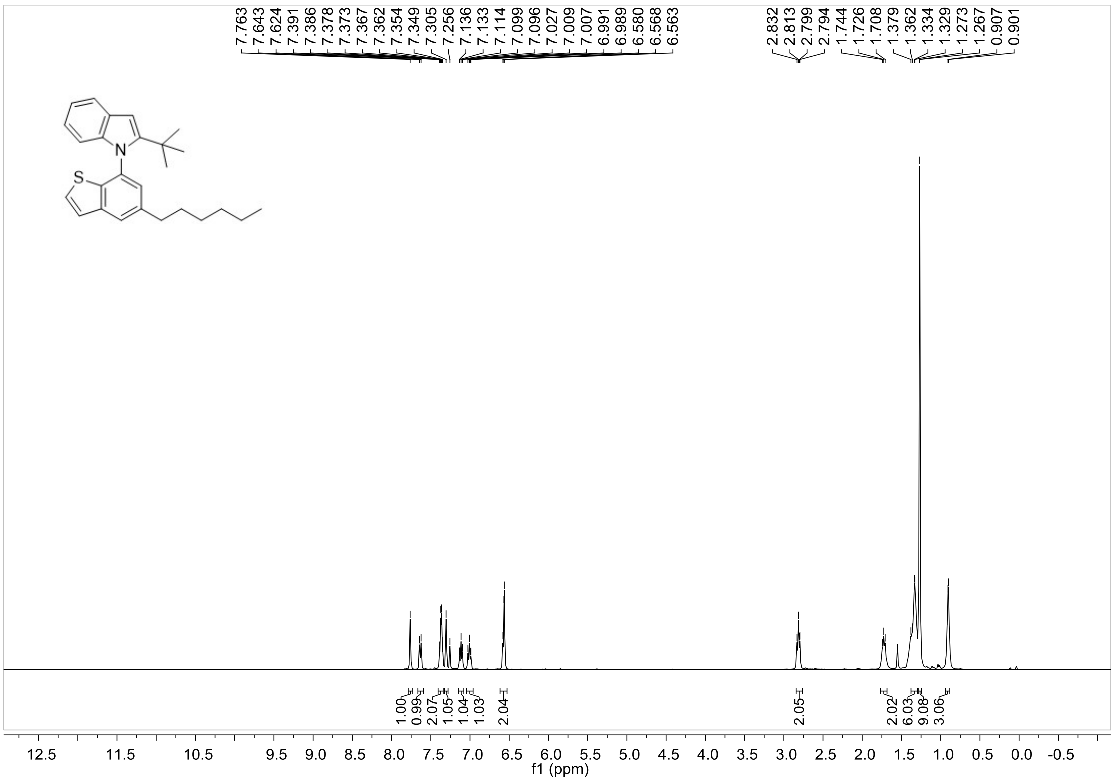

^{13}C NMR Spectrum of **72c**

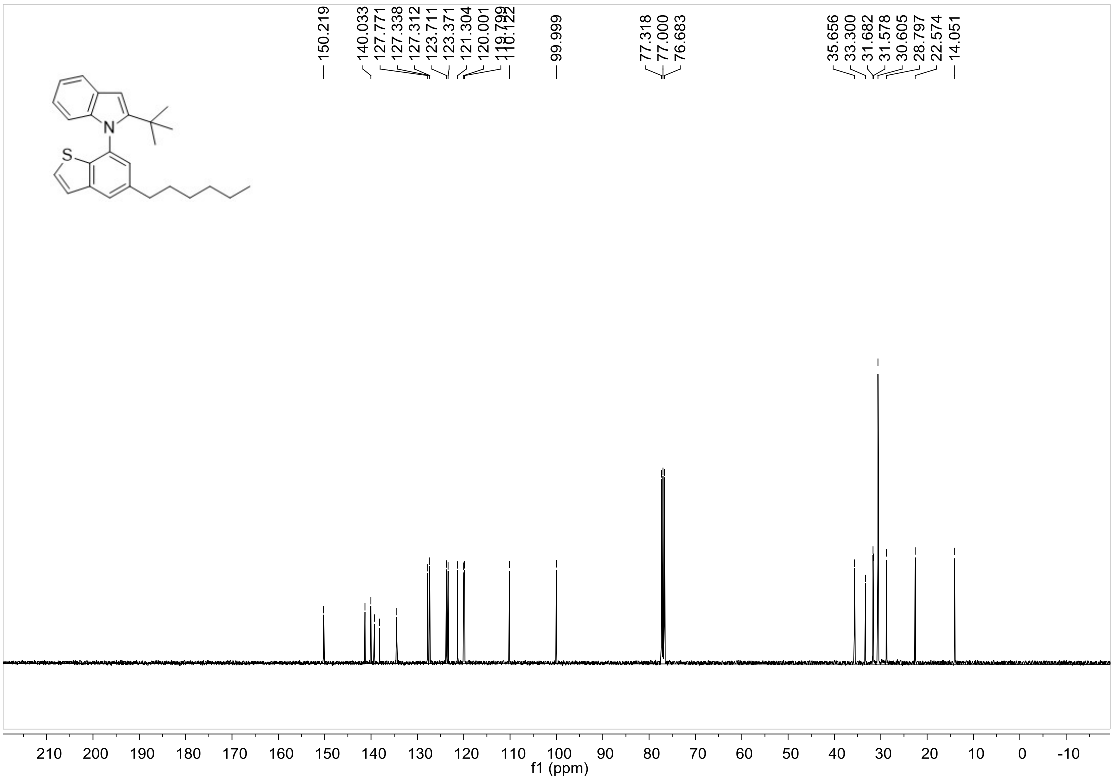

^{1}H NMR Spectrum of **73c**

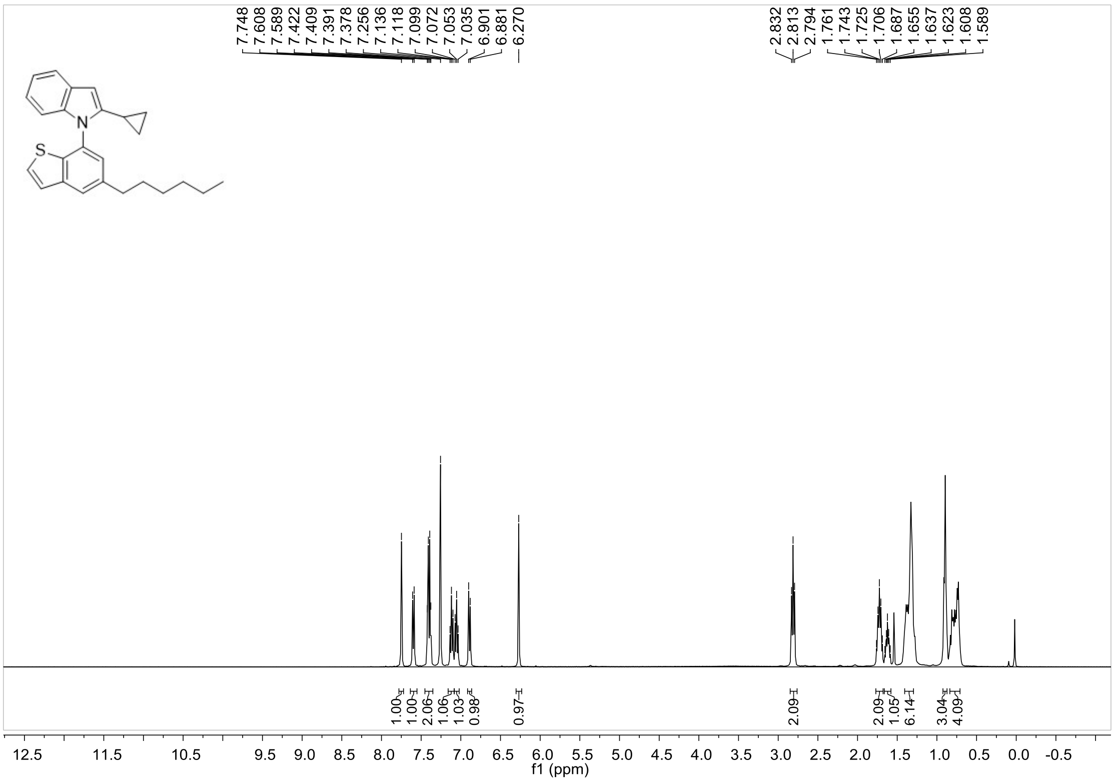

^{13}C NMR Spectrum of **73c**

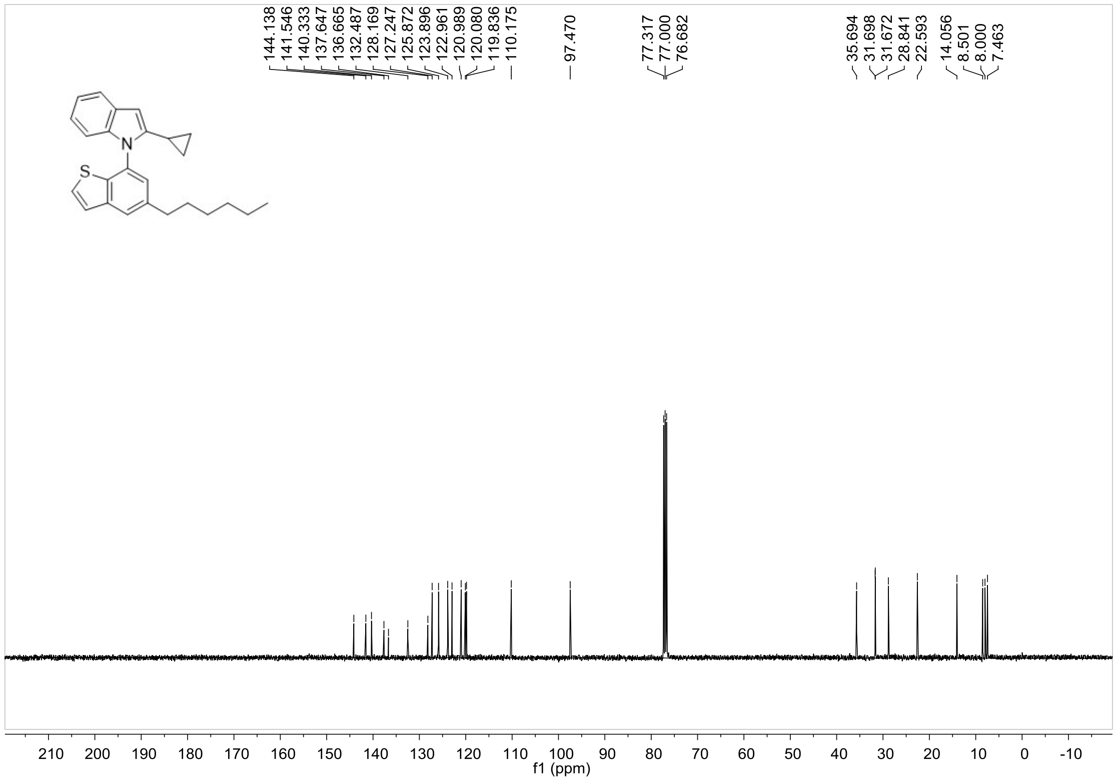

^{1}H NMR Spectrum of **74c**

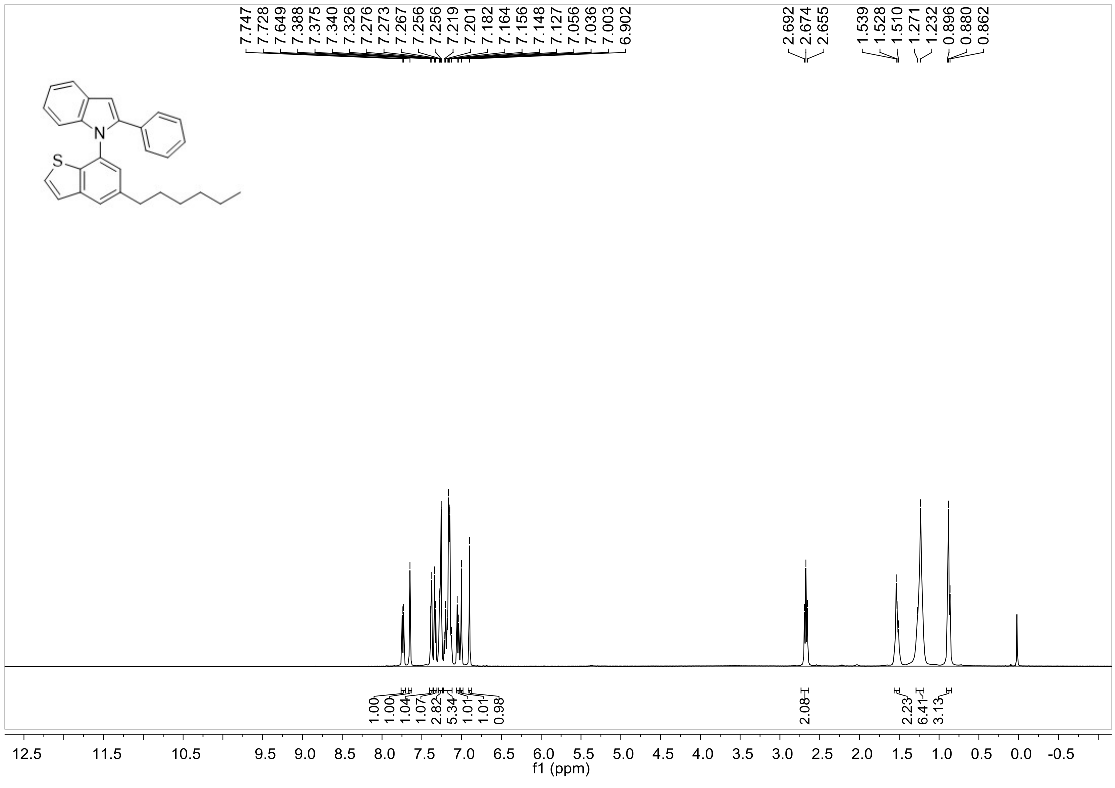

^{13}C NMR Spectrum of **74c**

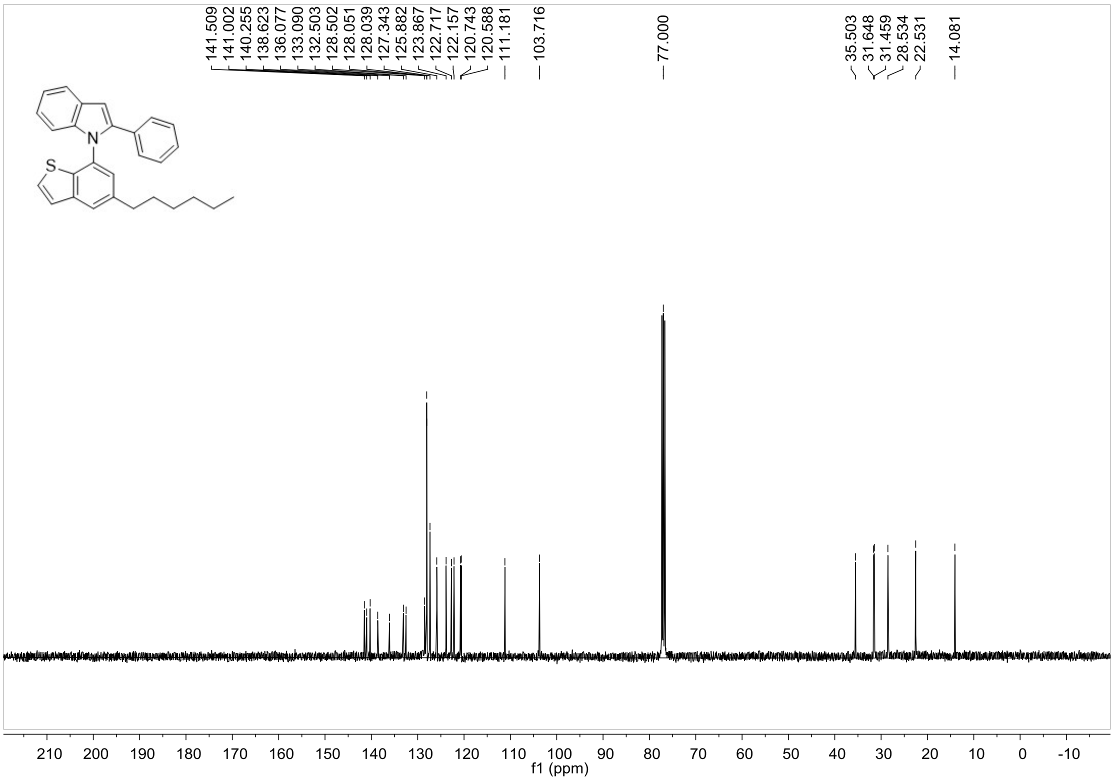

^{1}H NMR Spectrum of **75c**

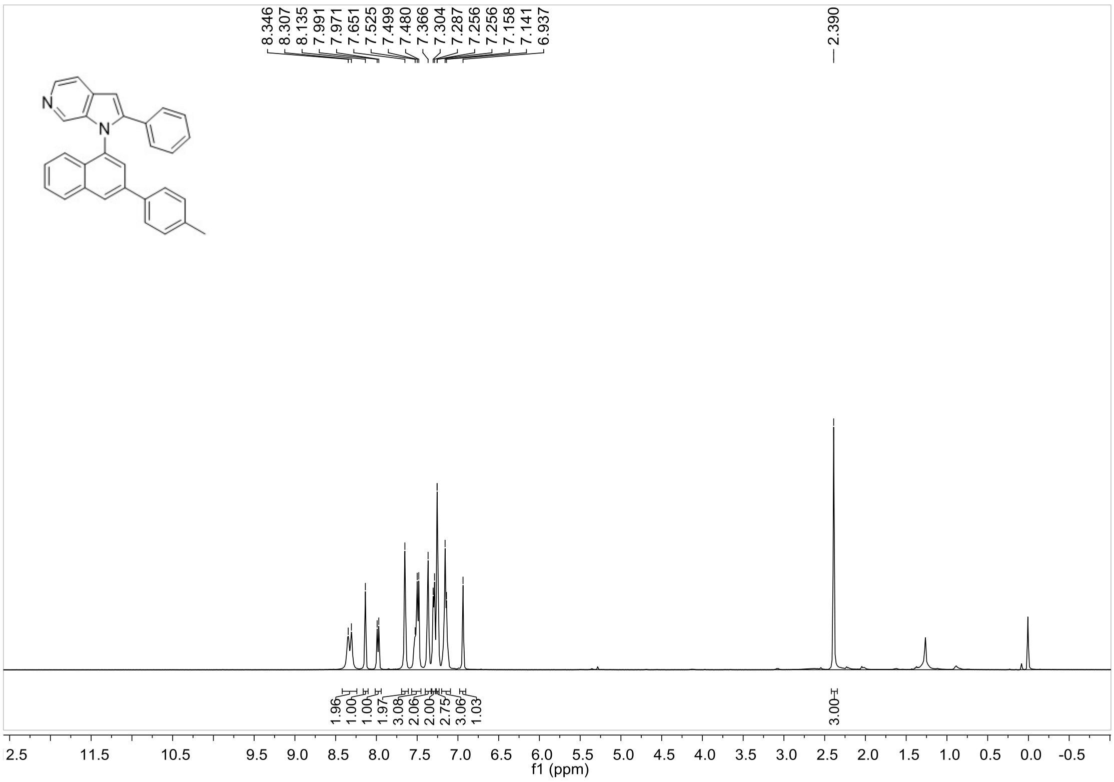

[13] C NMR Spectrum of **75c**

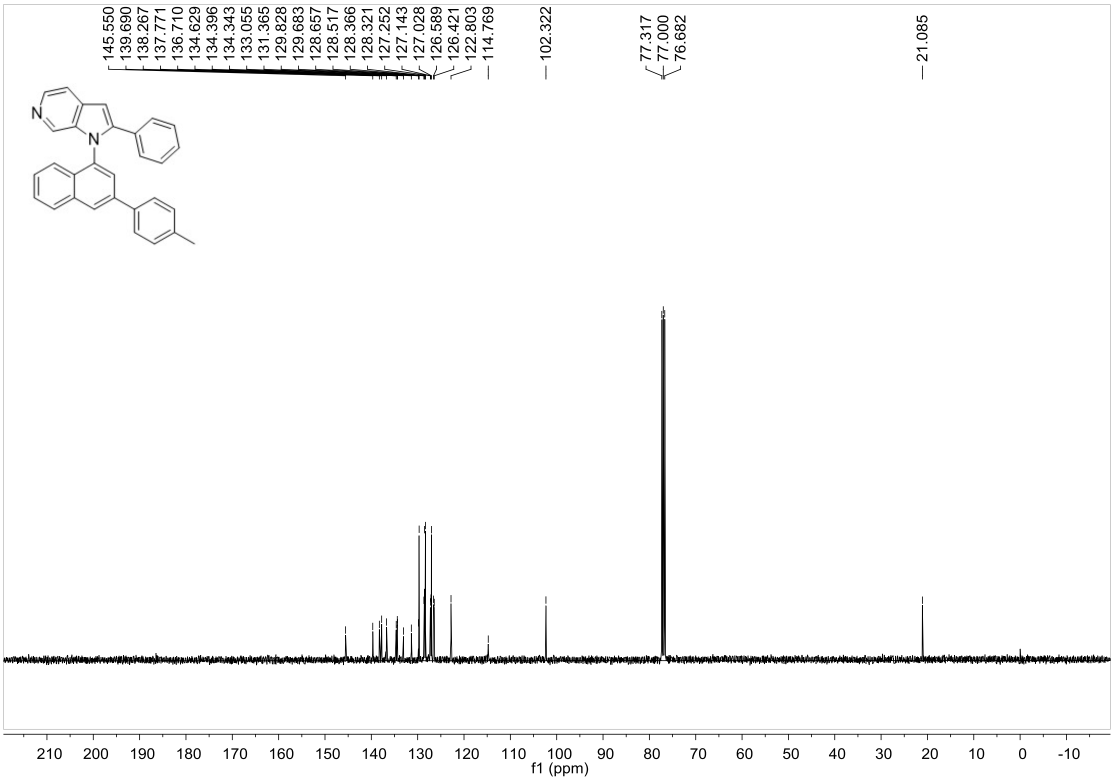

[1] H NMR Spectrum of **76c**

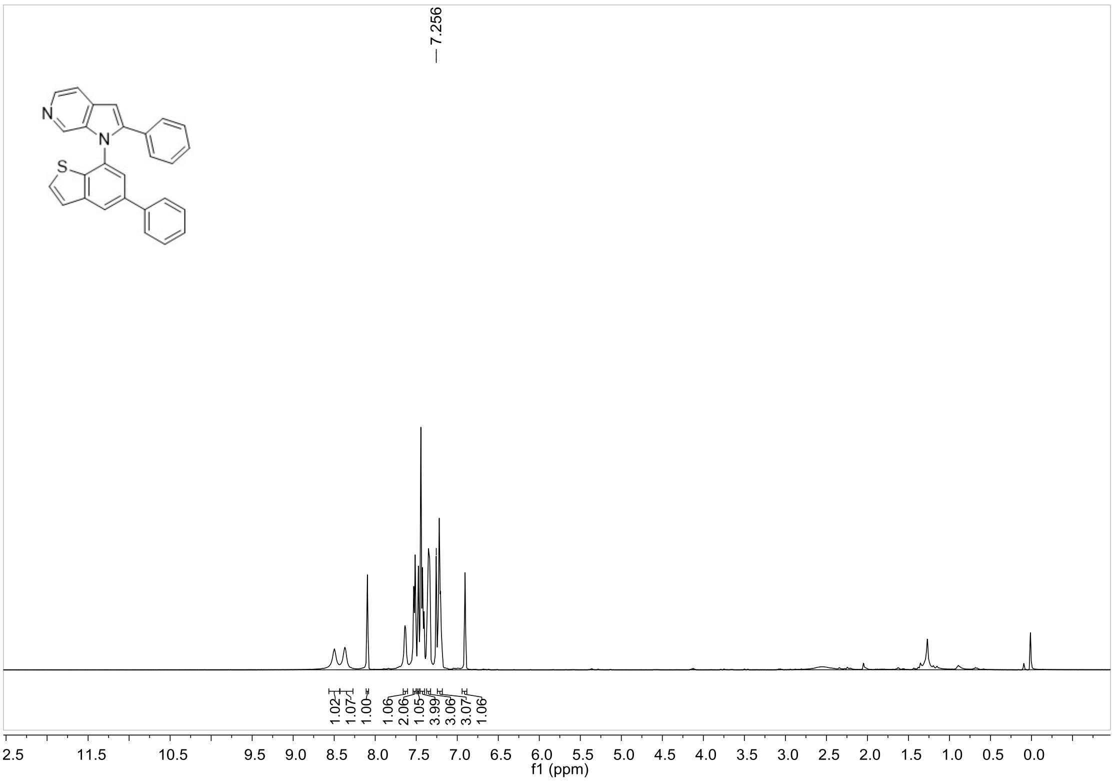

^{13}C NMR Spectrum of **76c**

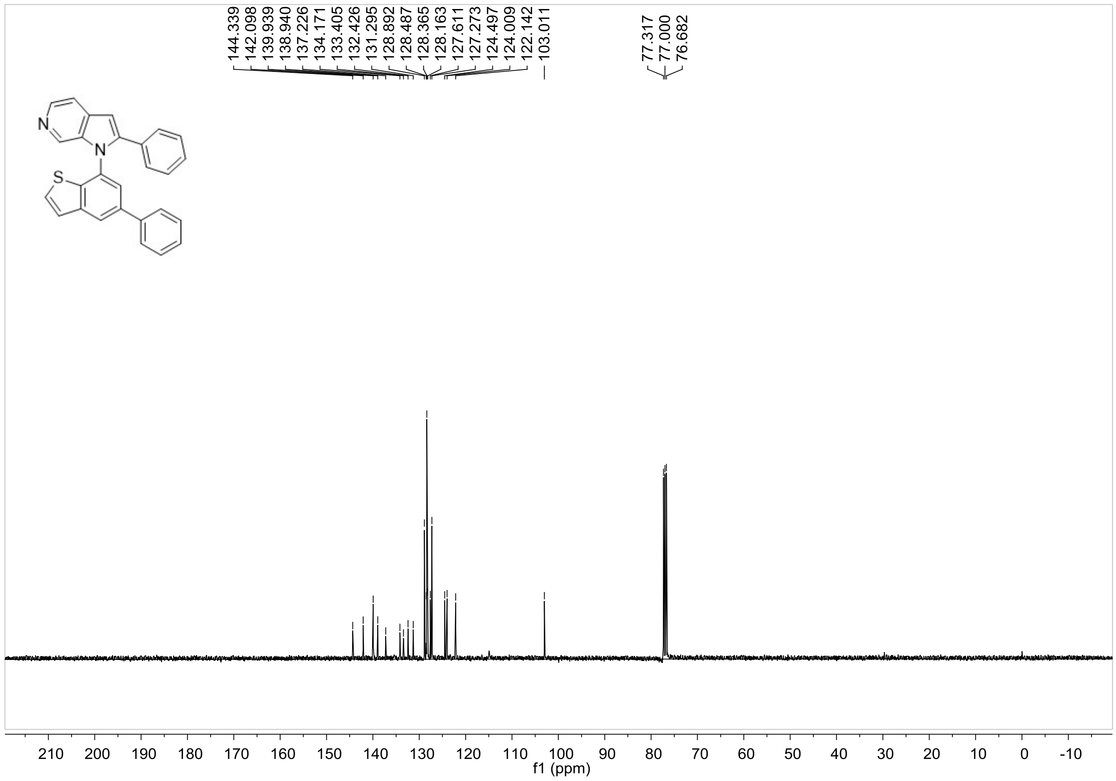

^{1}H NMR Spectrum of **77c**

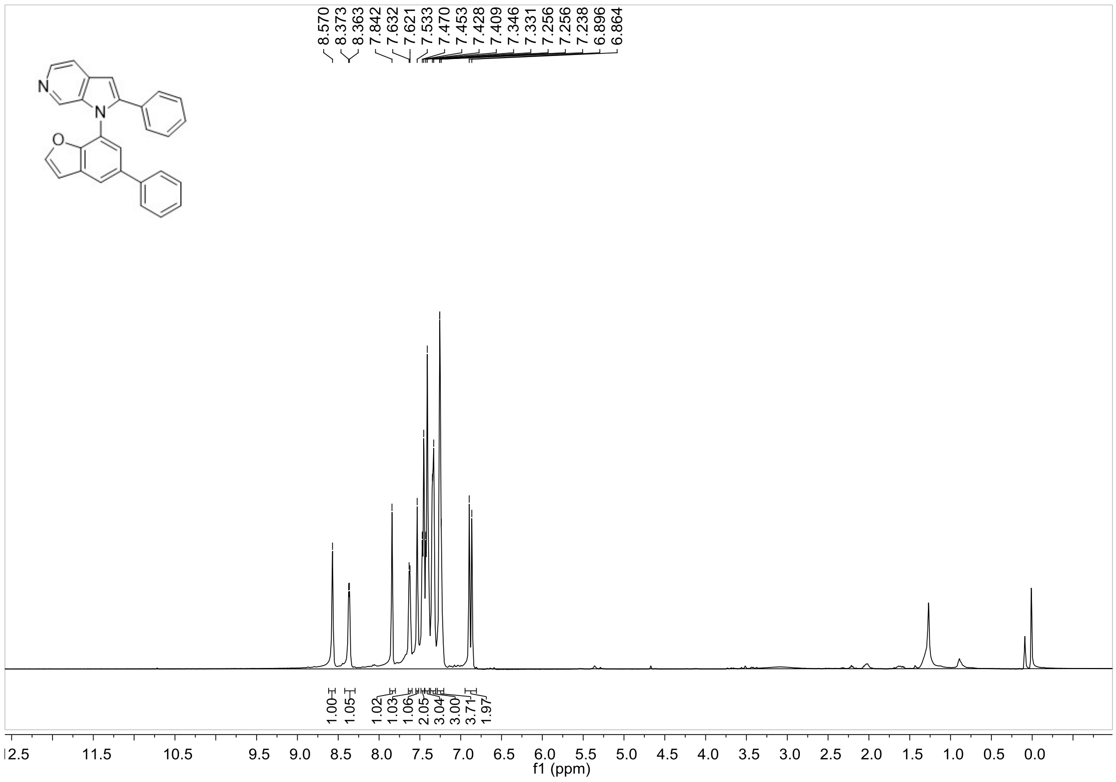

^{13}C NMR Spectrum of **77c**

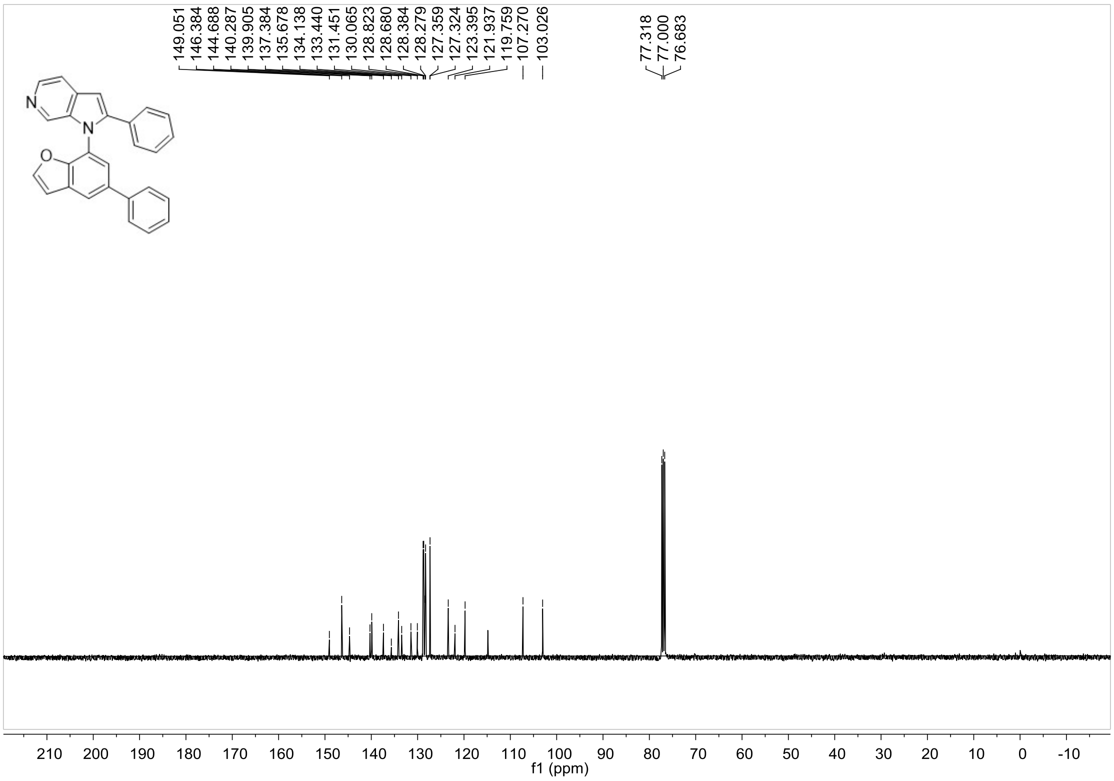

^{1}H NMR Spectrum of **78c**

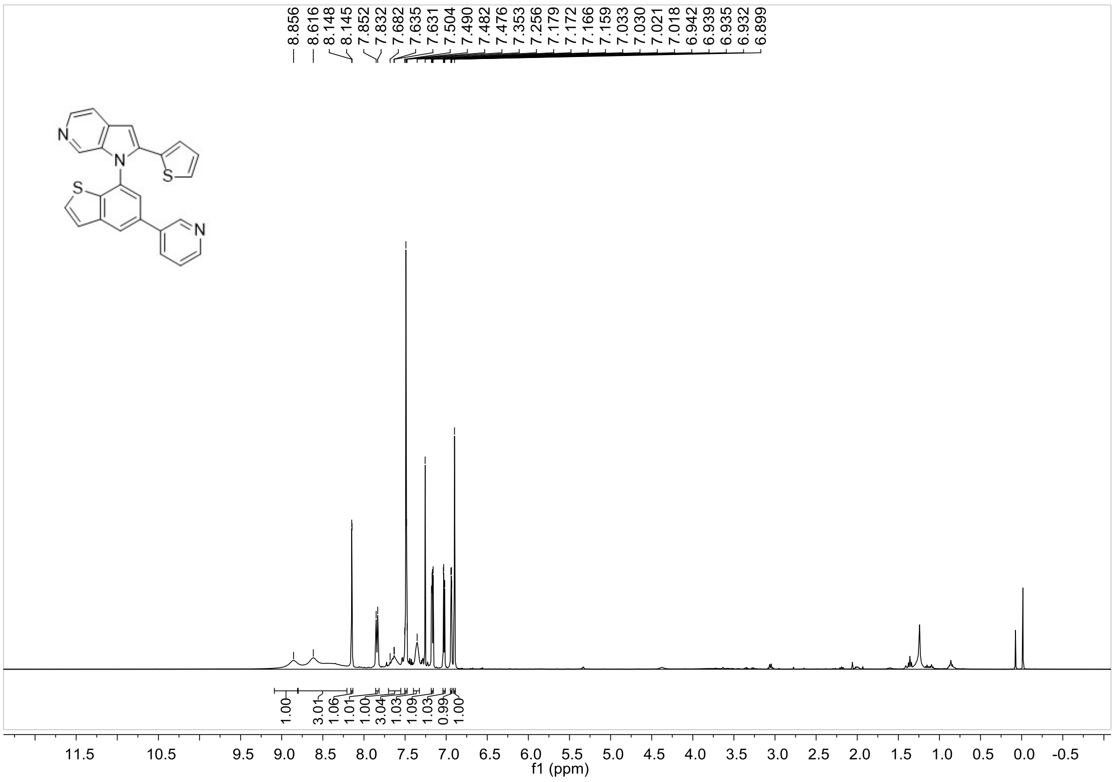

^{13}C NMR Spectrum of **78c**

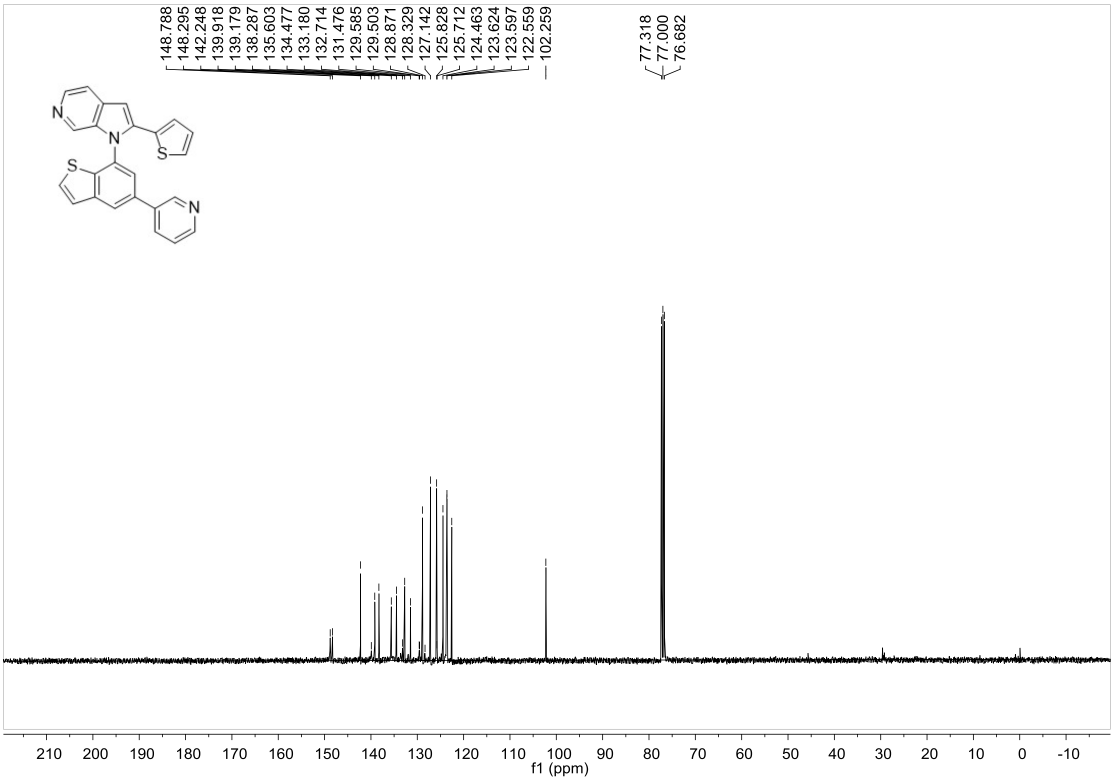

^{1}H NMR Spectrum of **79c**

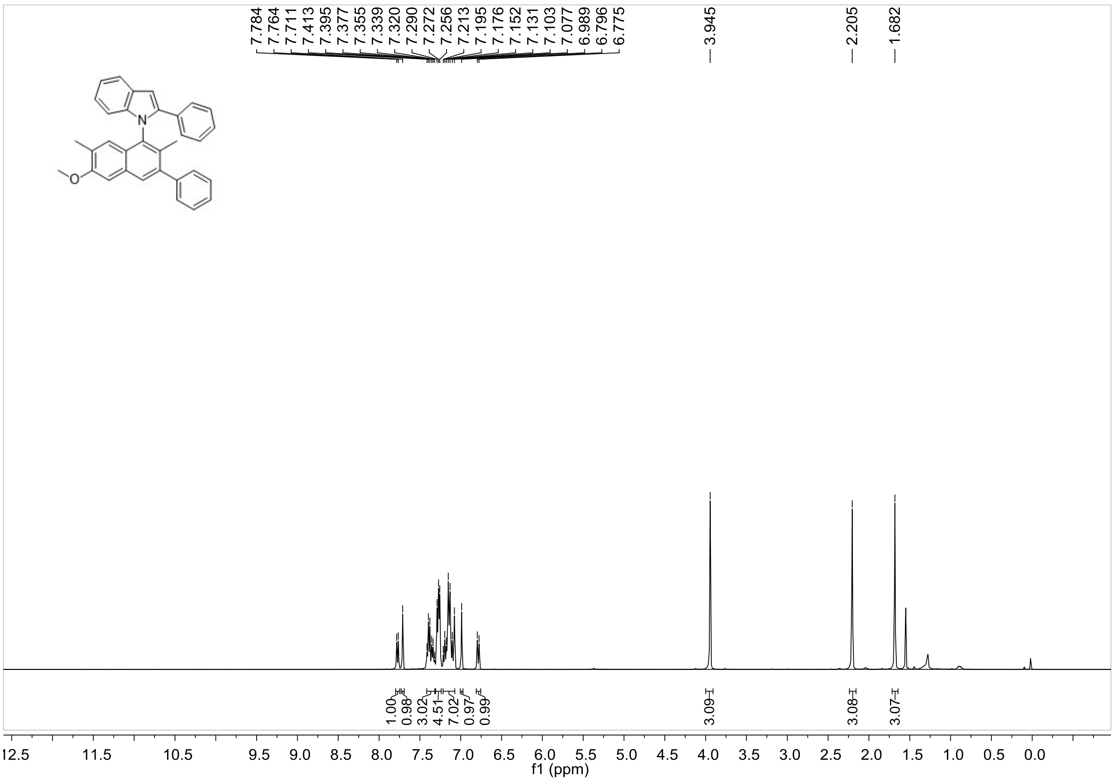

^{13}C NMR Spectrum of **79c**

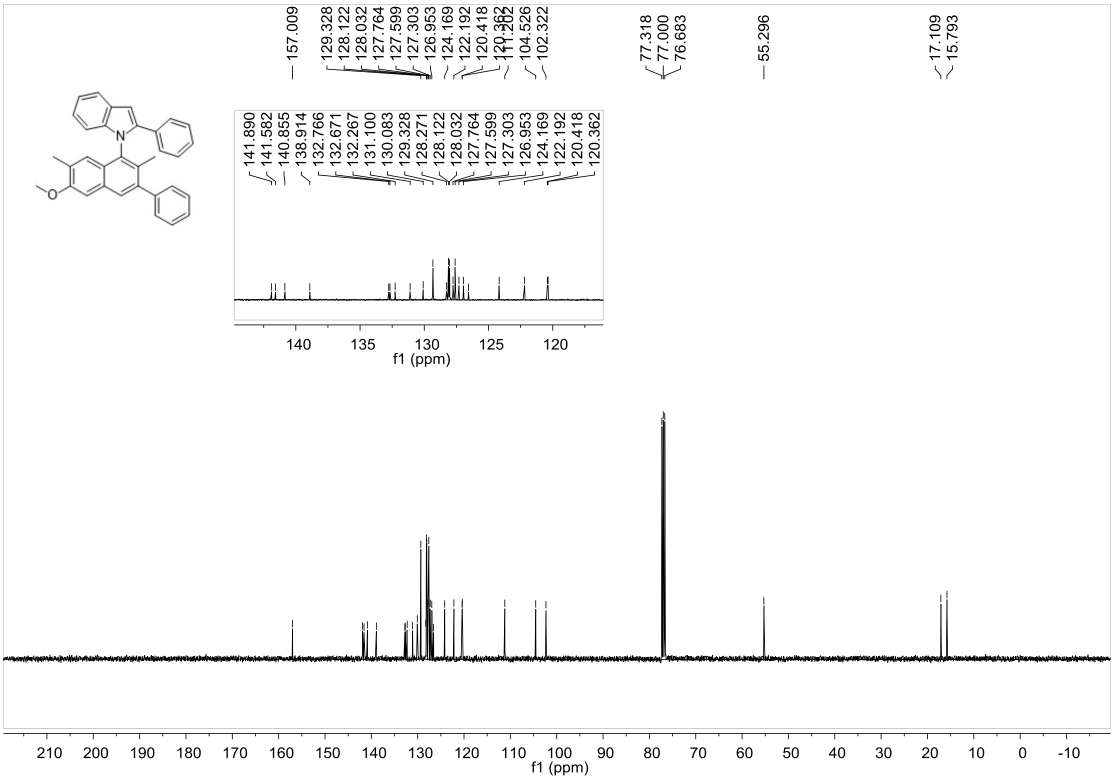

^{1}H NMR Spectrum of **80c**

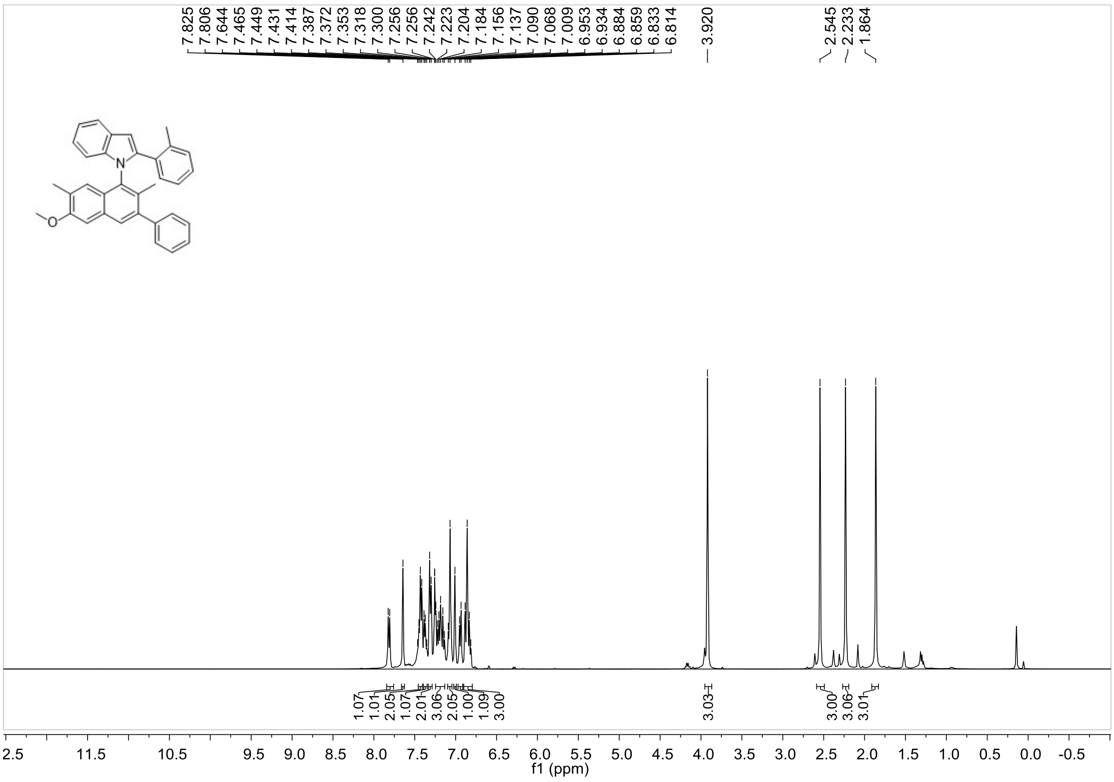

^{13}C NMR Spectrum of **80c**

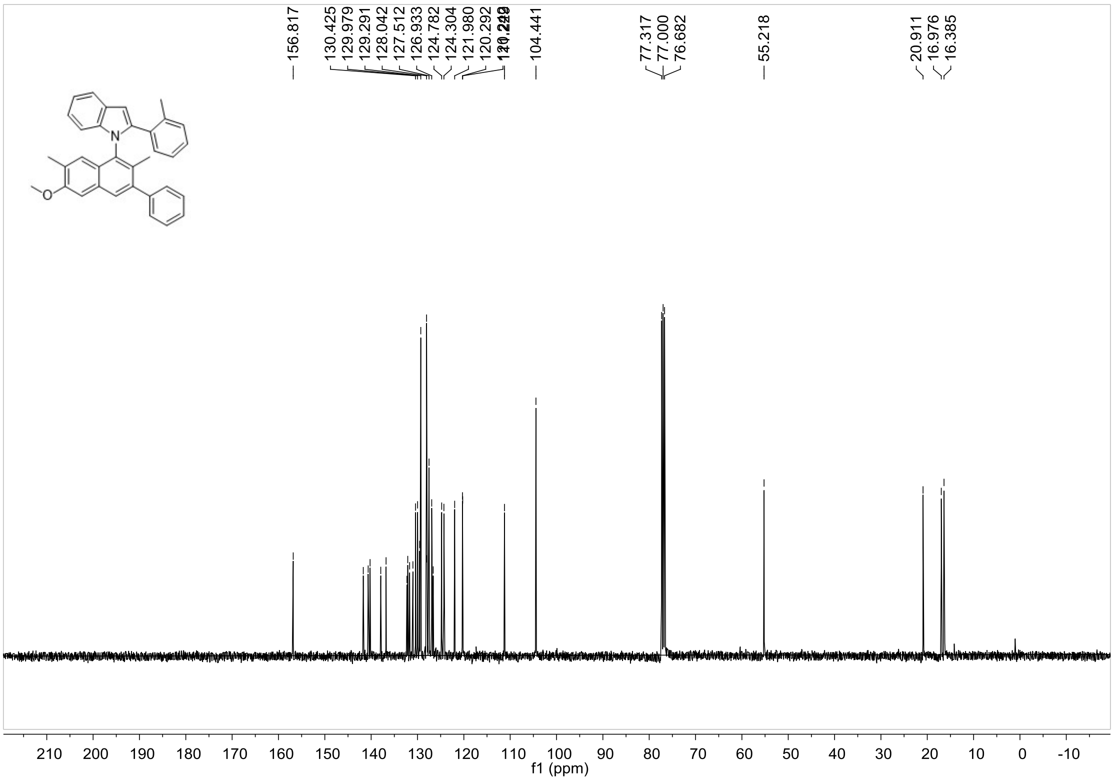

^{1}H NMR Spectrum of **81c**

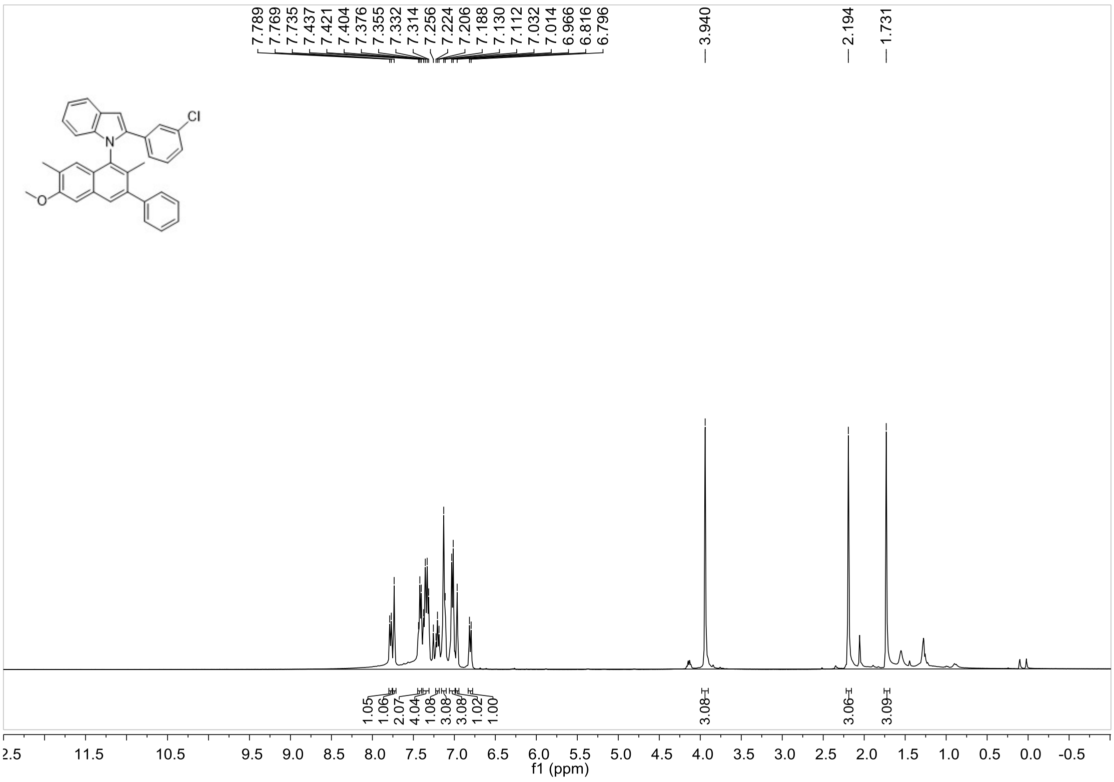

^{13}C NMR Spectrum of **81c**

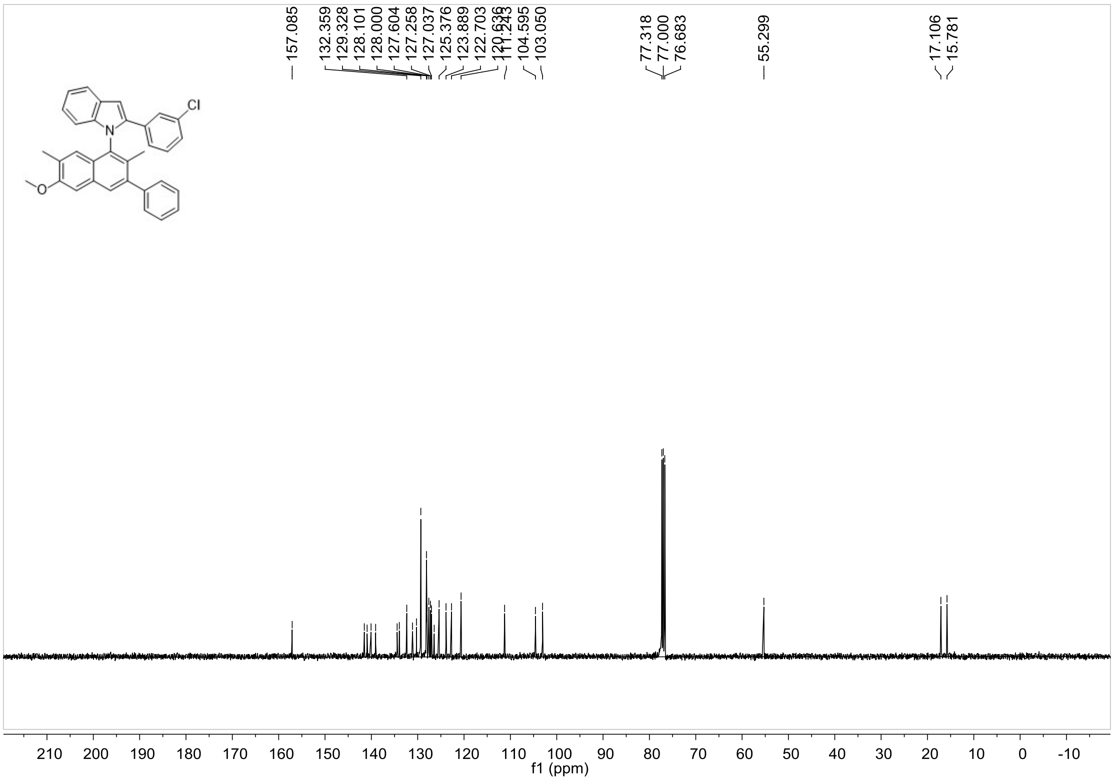

^{1}H NMR Spectrum of **1e**

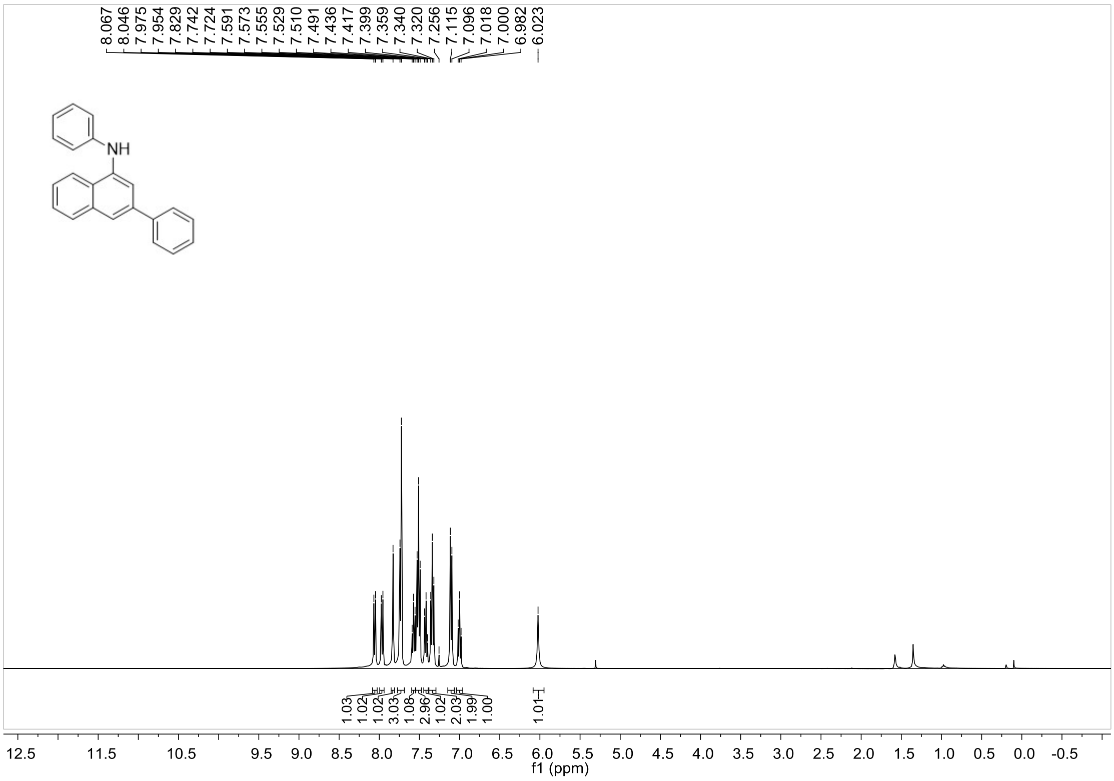

^{13}C NMR Spectrum of **1e**

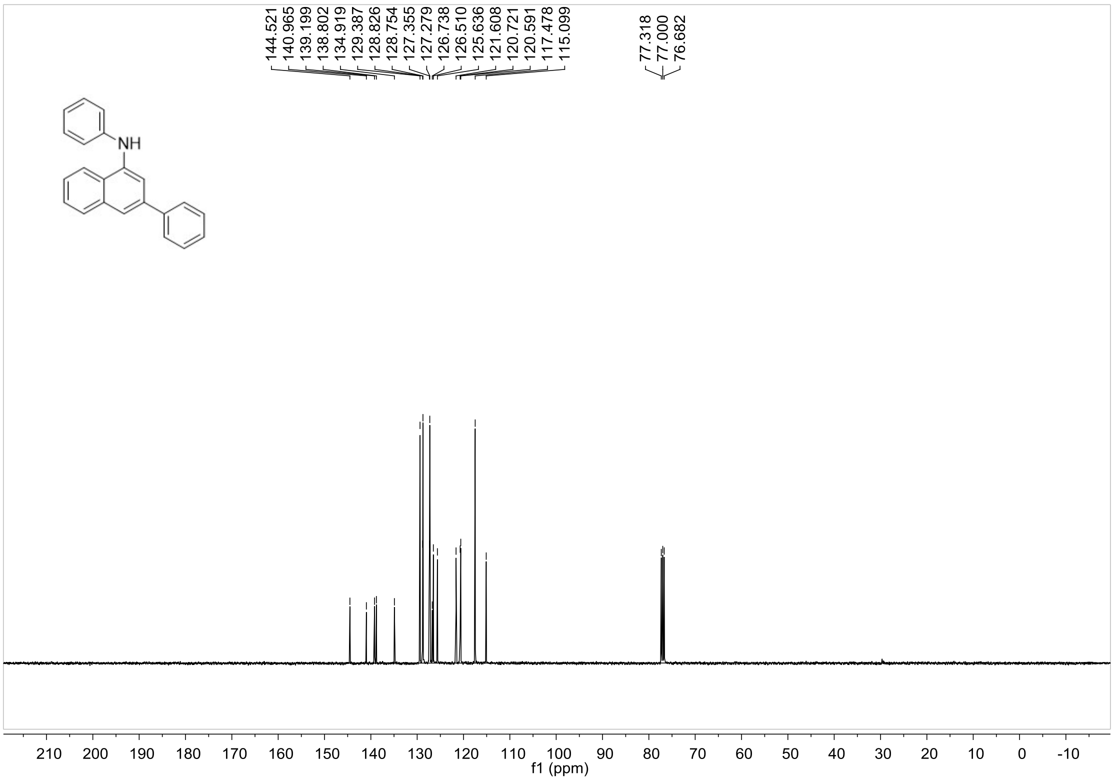

^{1}H NMR Spectrum of **2e**

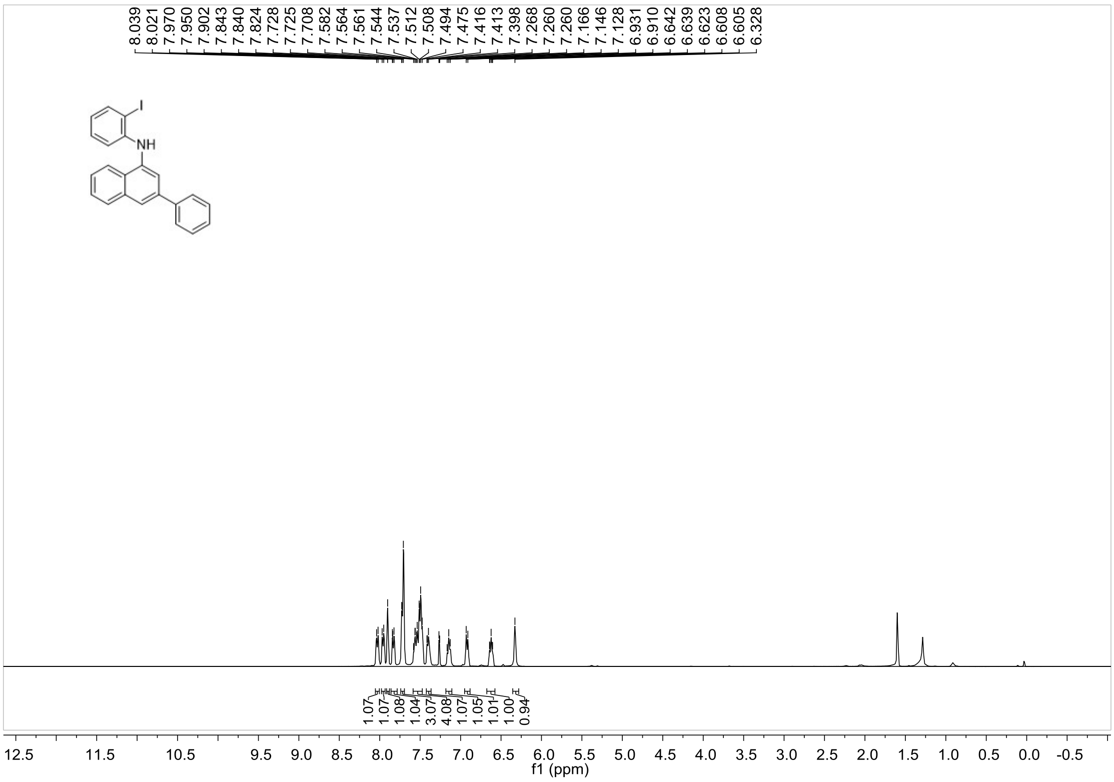

^{13}C NMR Spectrum of **2e**

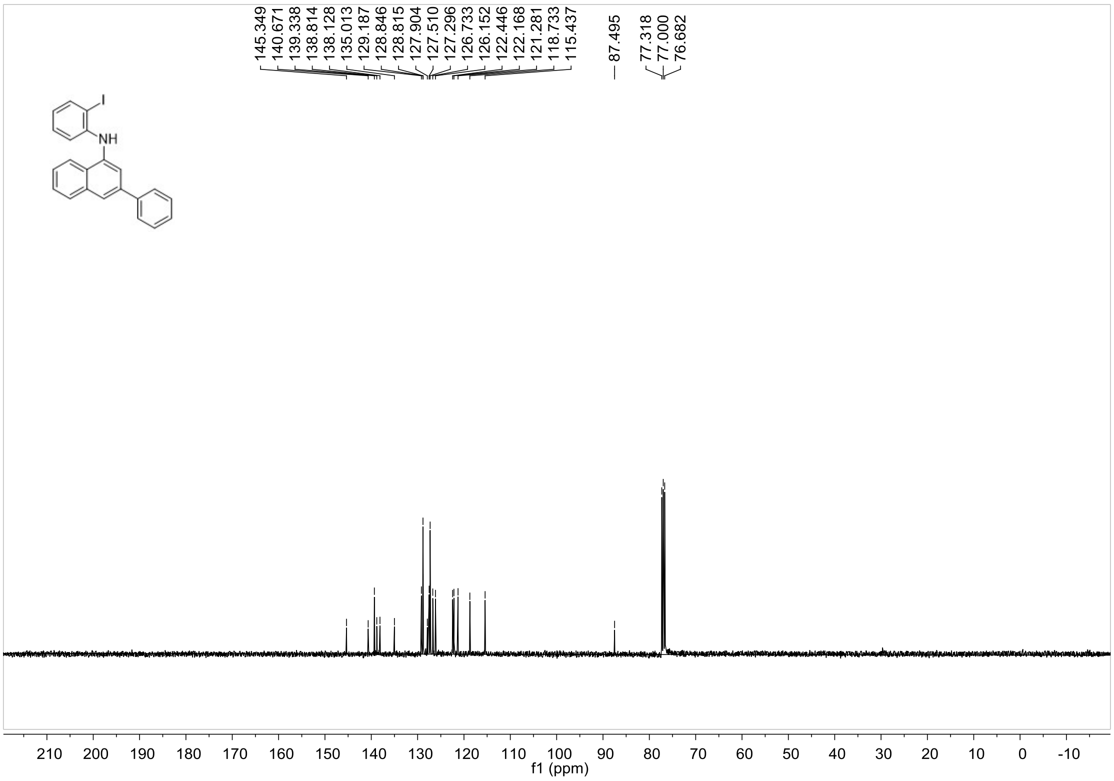

^{1}H NMR Spectrum of **1e'**

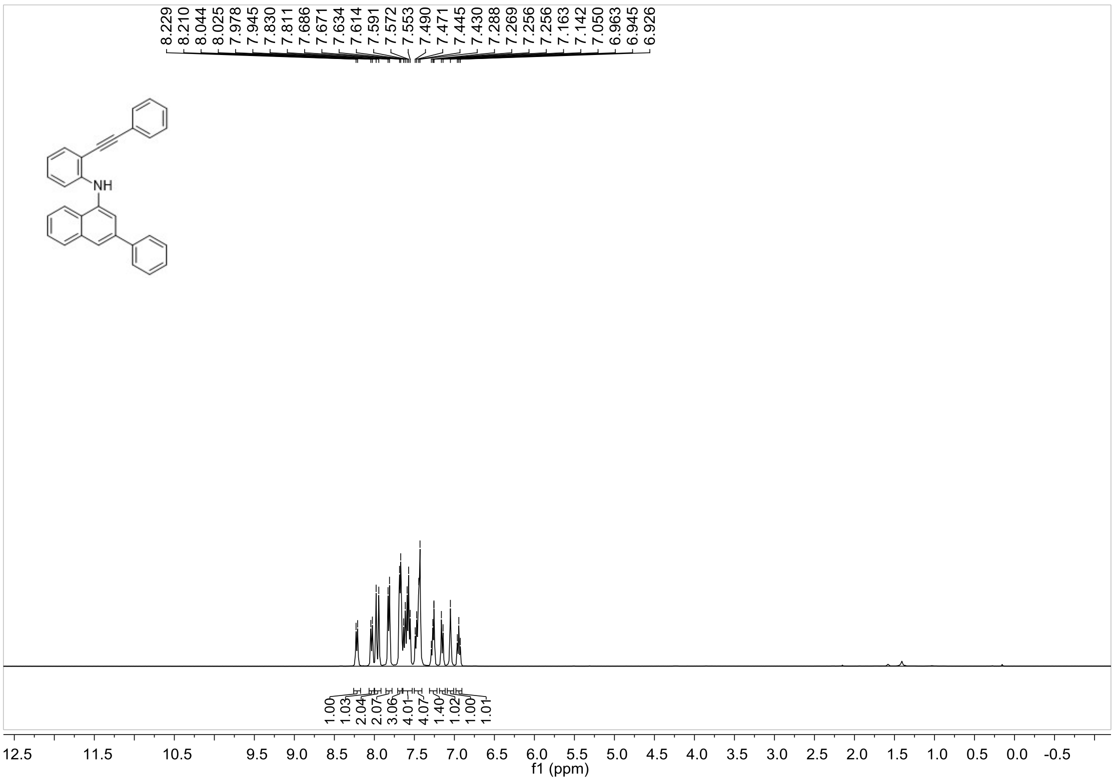

^{13}C NMR Spectrum of **1e'**

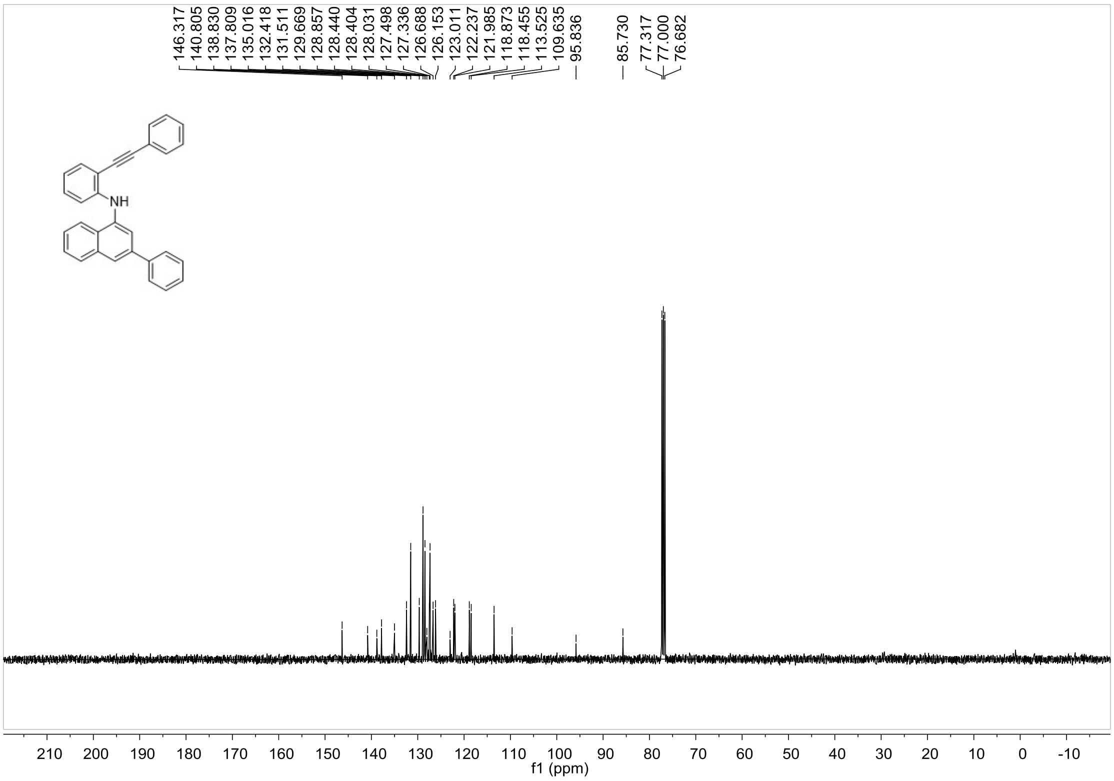

^{1}H NMR Spectrum of **3a**

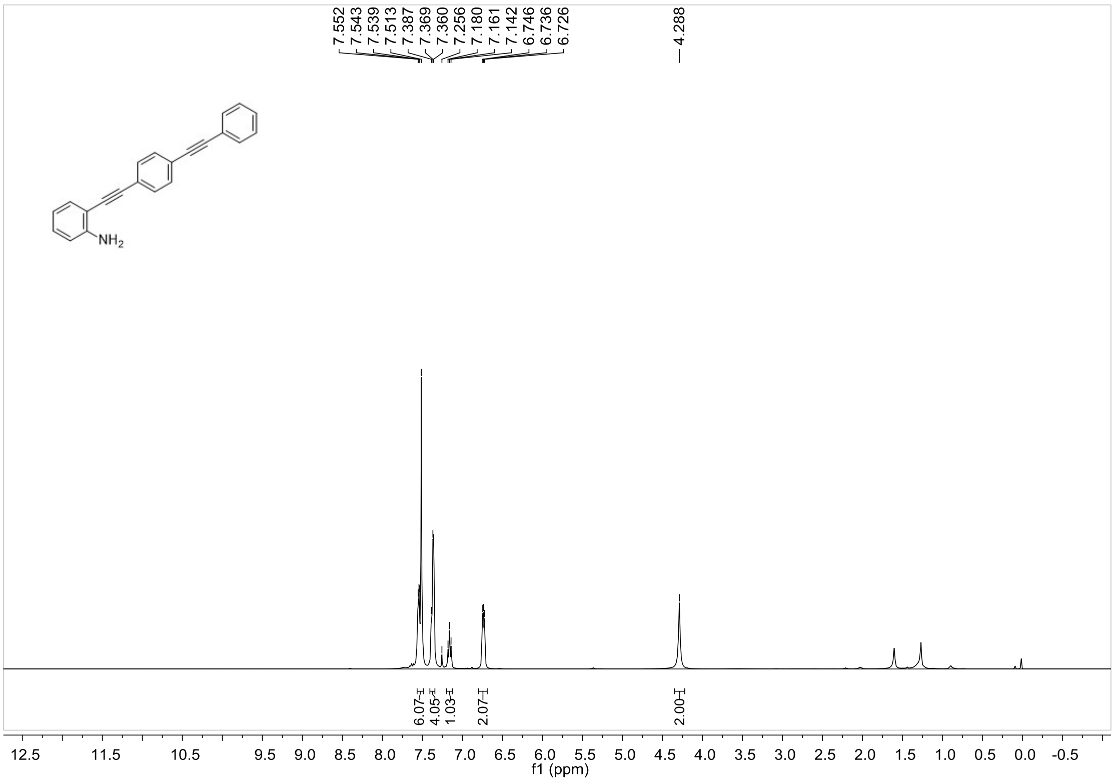

^{13}C NMR Spectrum of **3a**

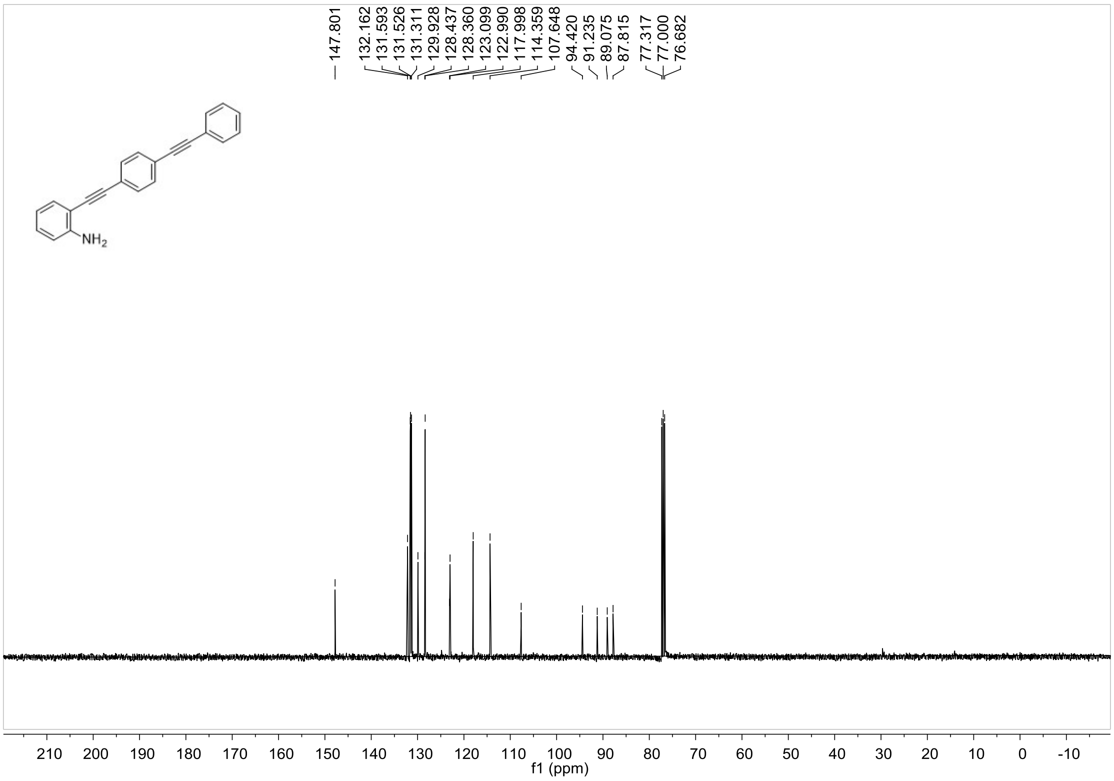

^{1}H NMR Spectrum of **4a**

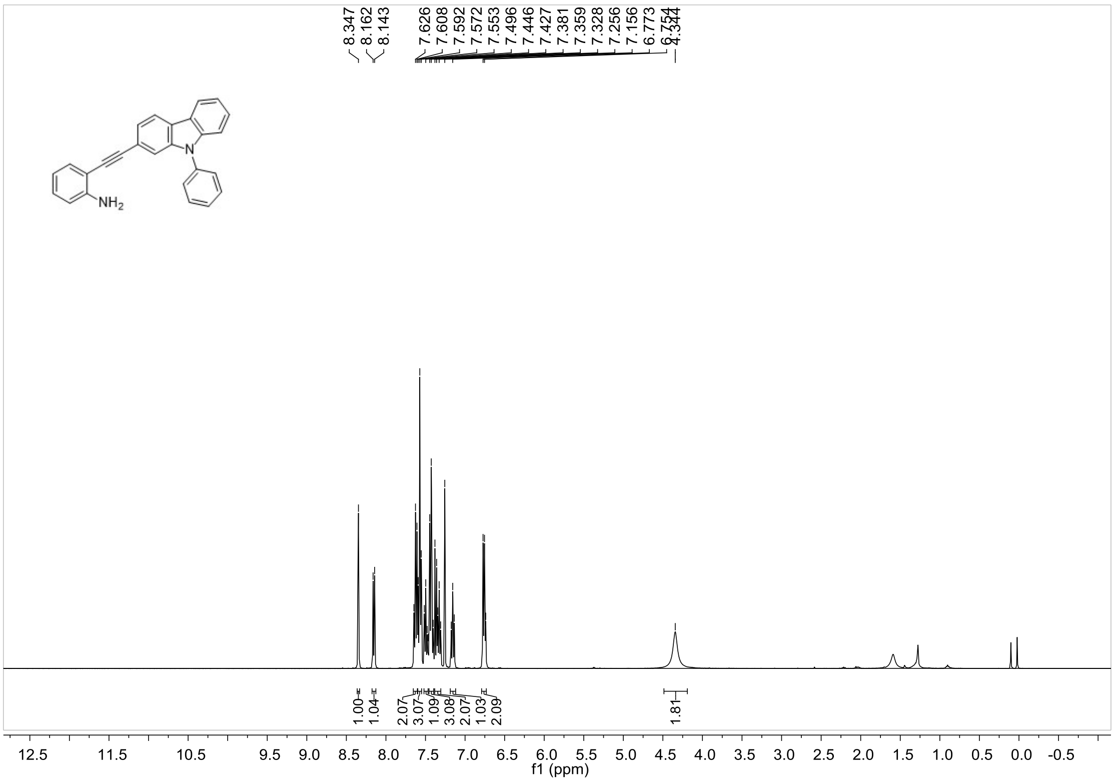

^{13}C NMR Spectrum of **4a**

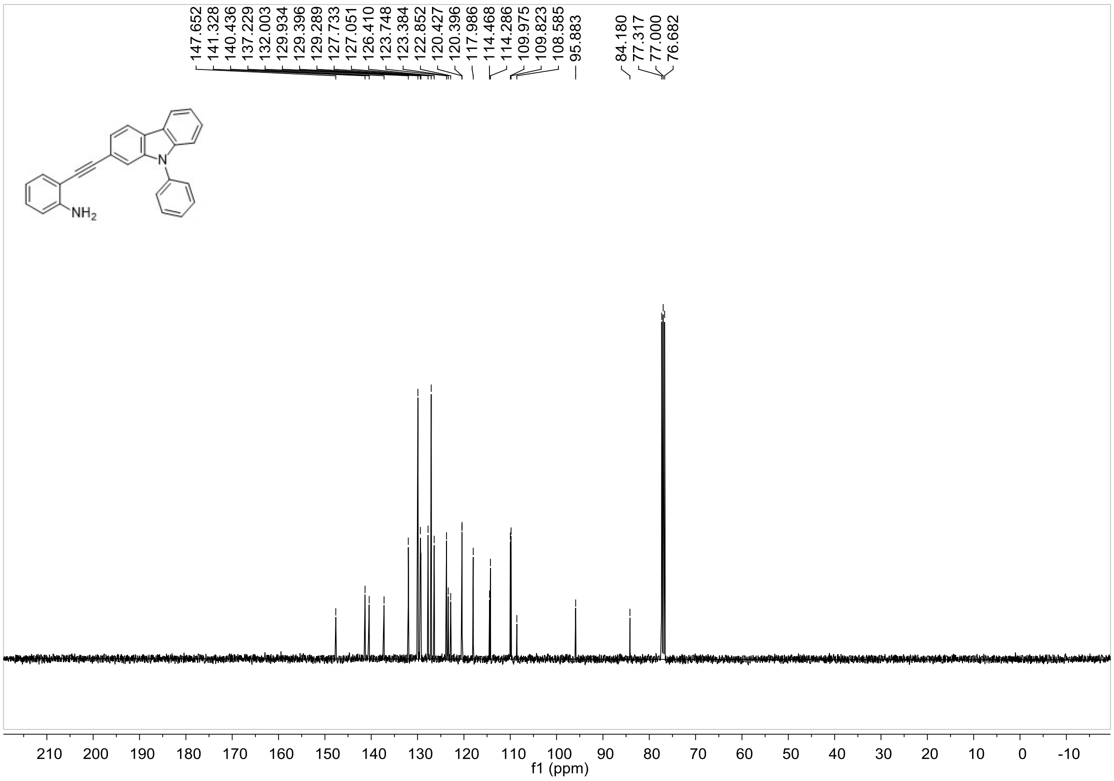

^{1}H NMR Spectrum of **5a**

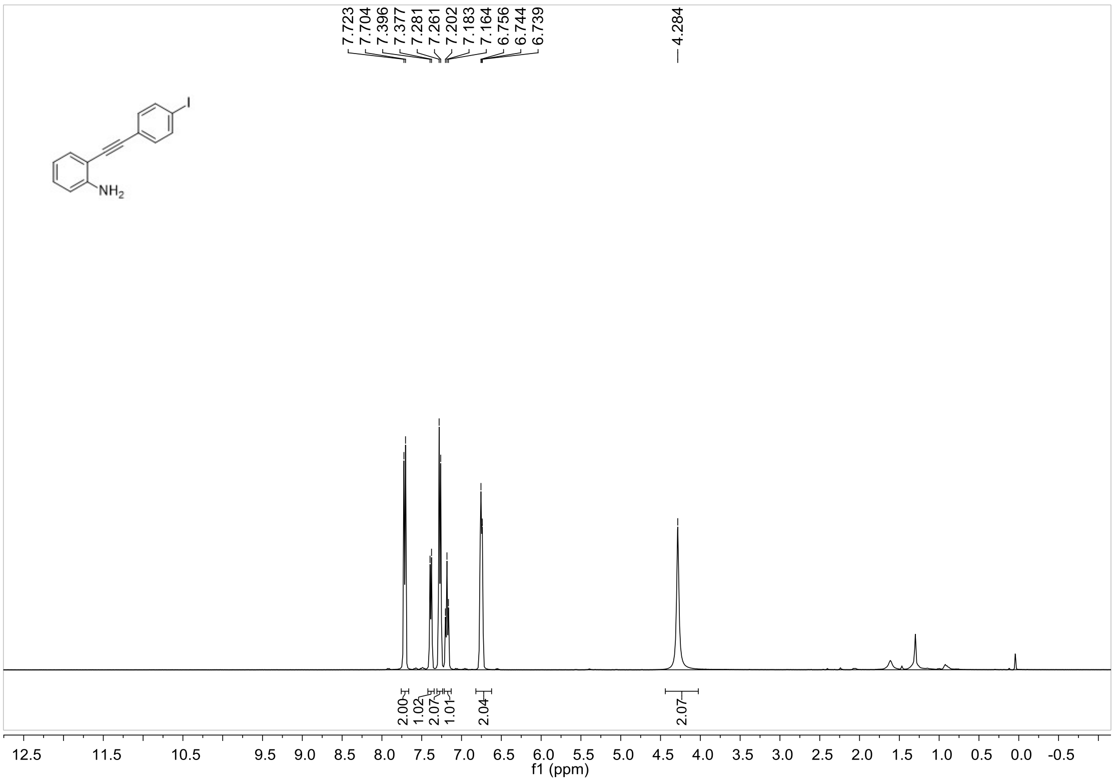

^{13}C NMR Spectrum of **5a**

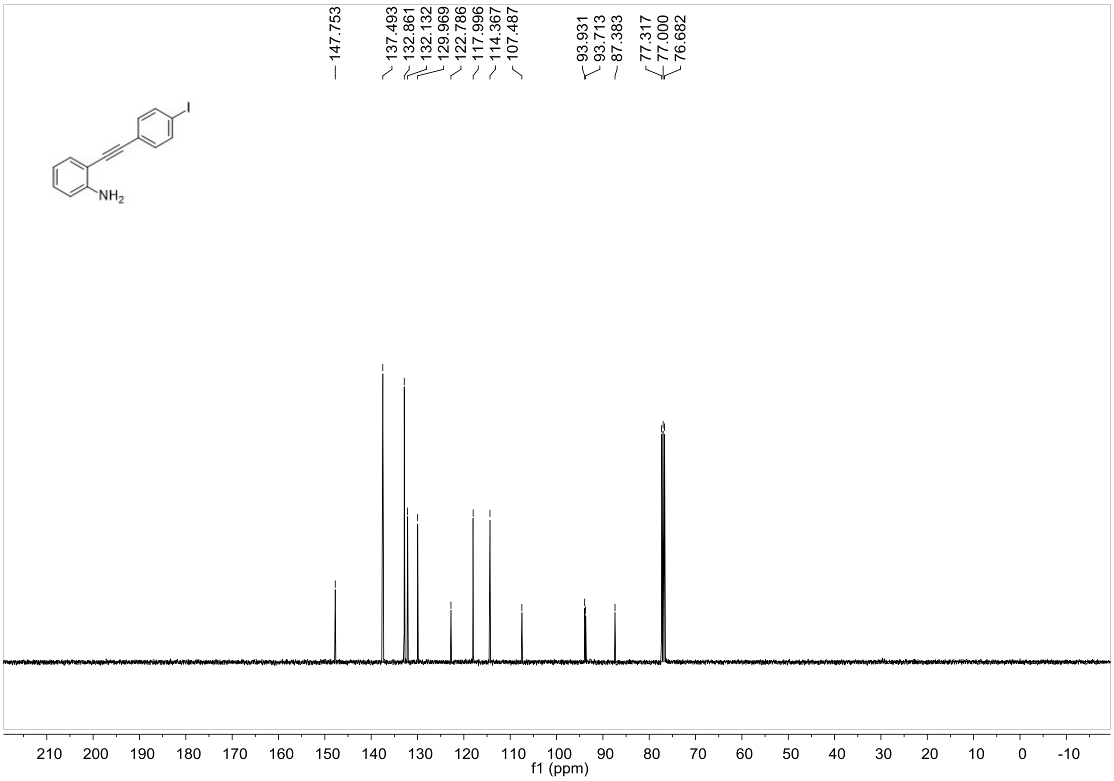

^{1}H NMR Spectrum of **6a**

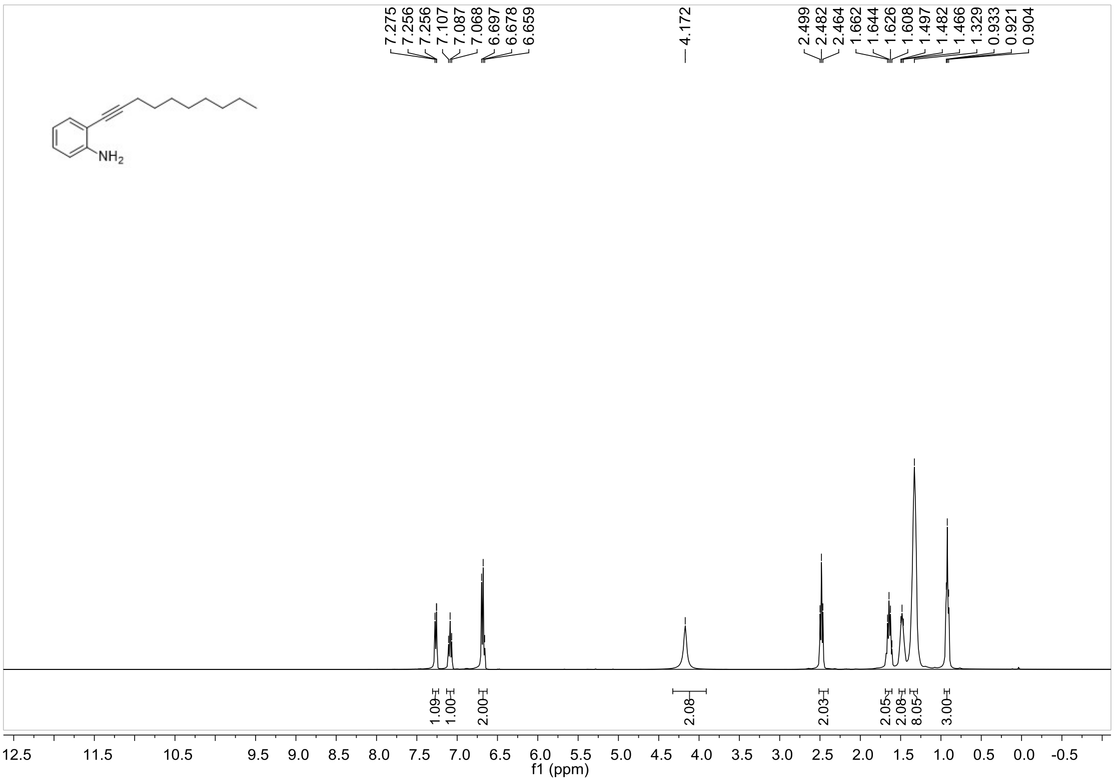

^{13}C NMR Spectrum of **6a**

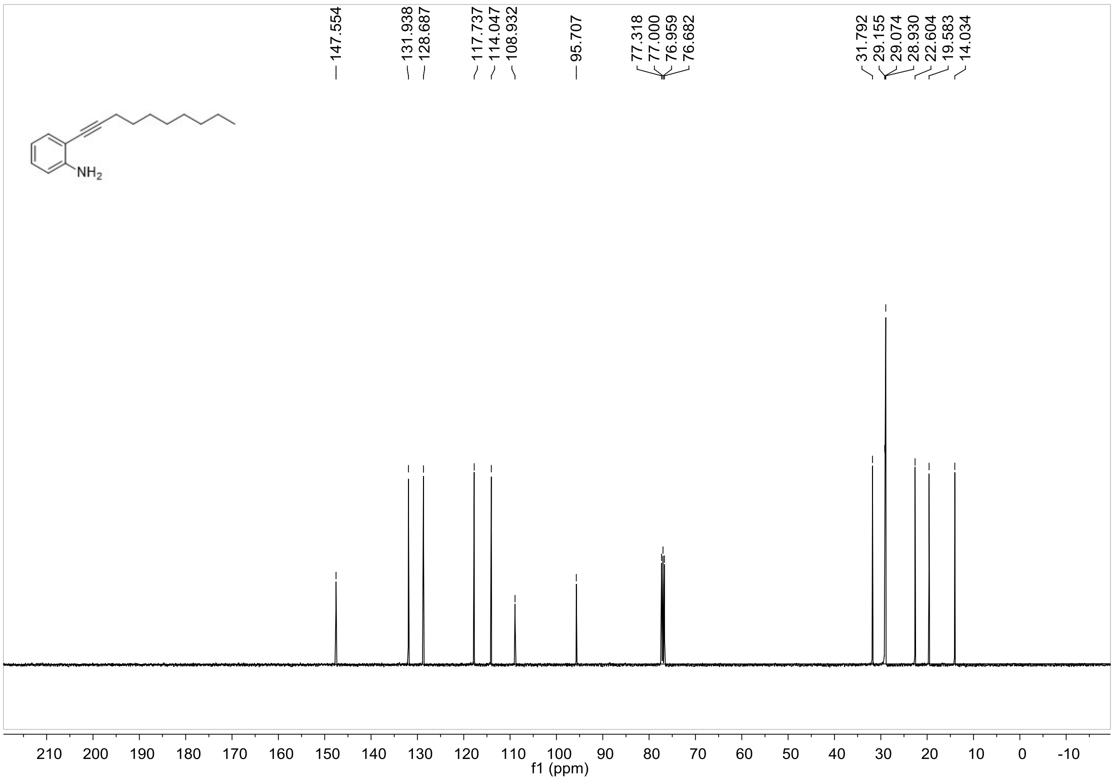

^{1}H NMR Spectrum of **7a**

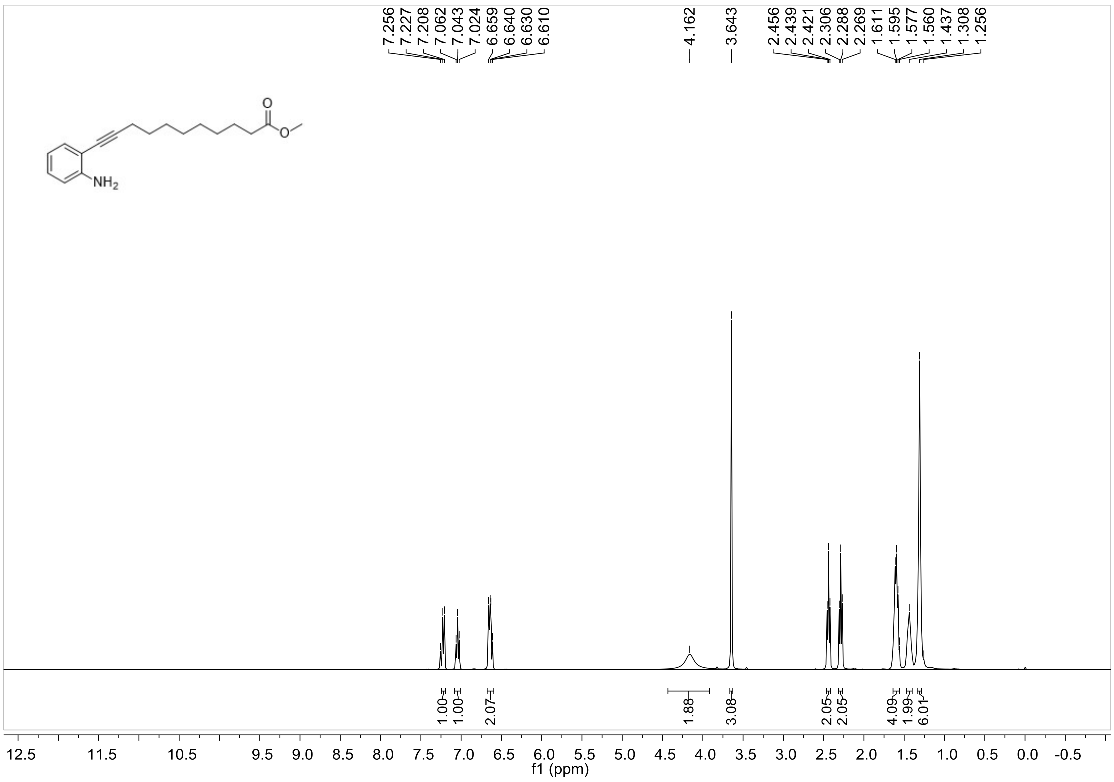

^{13}C NMR Spectrum of **7a**

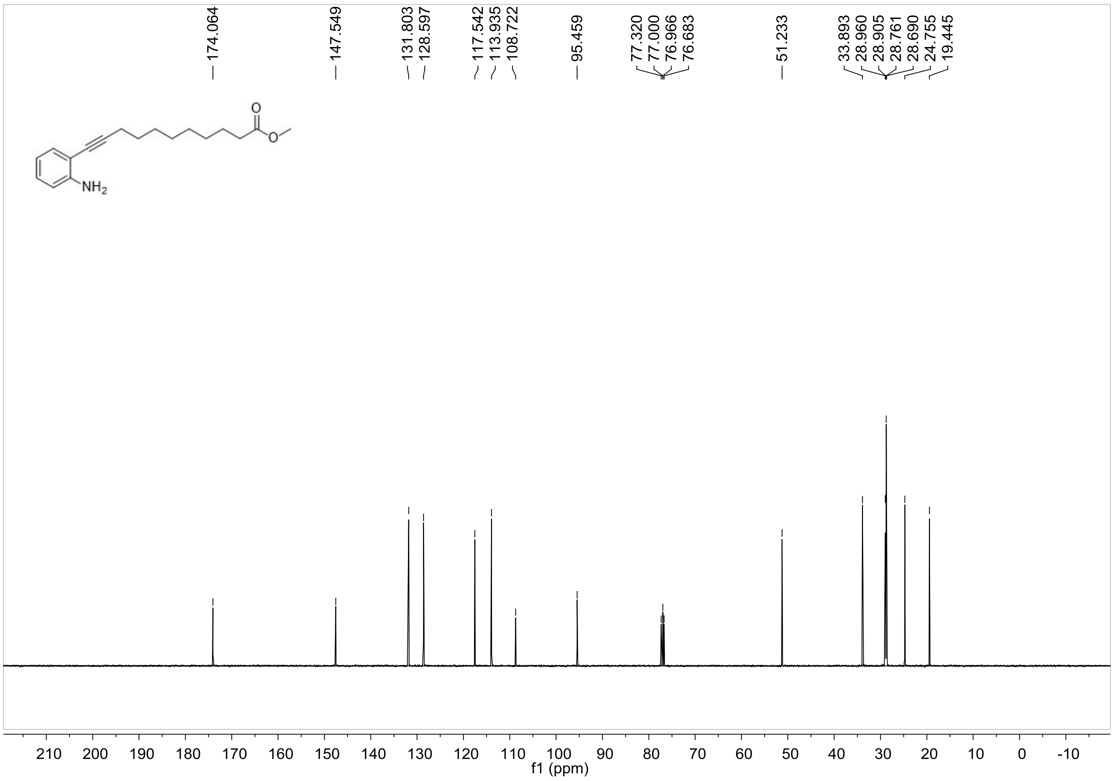

^{1}H NMR Spectrum of **8a**

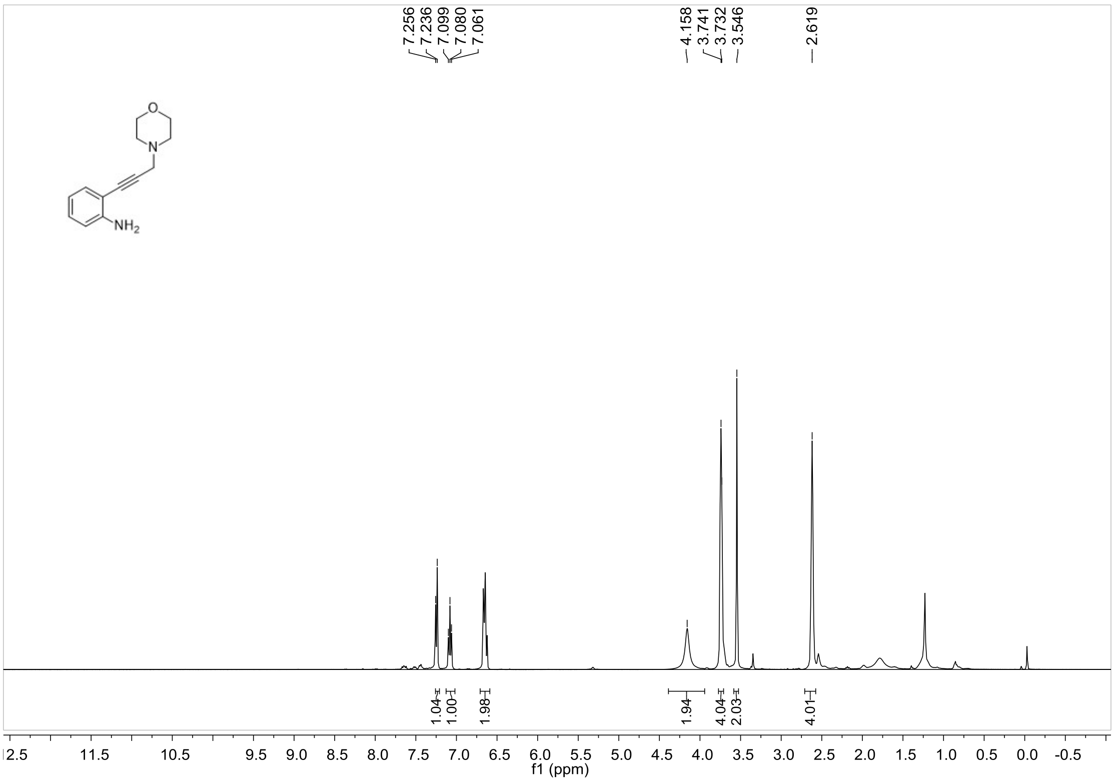

^{13}C NMR Spectrum of **8a**

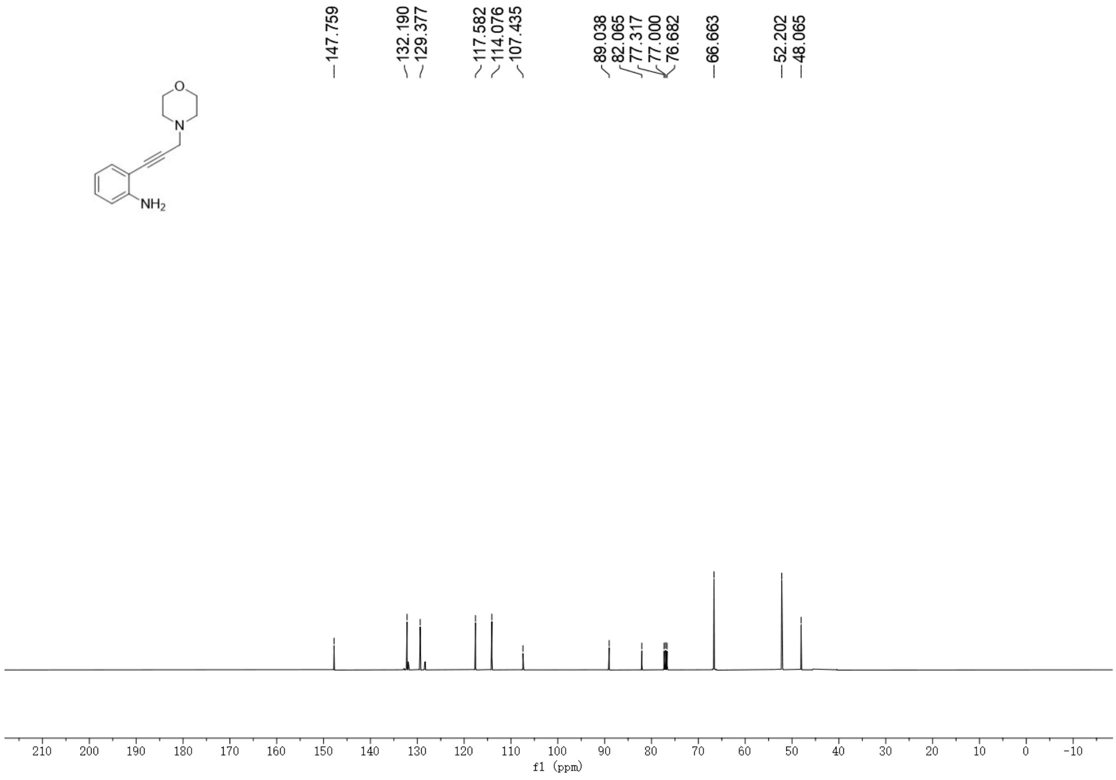

^{1}H NMR Spectrum of **9a**

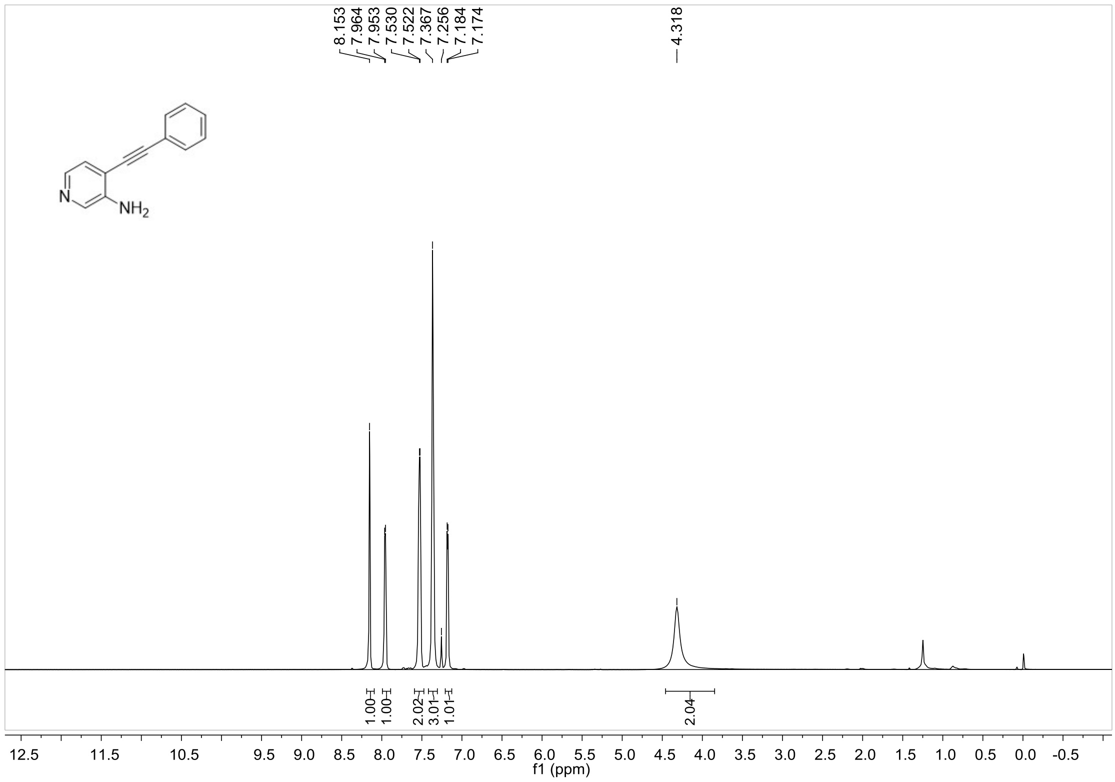

^{13}C NMR Spectrum of **9a**

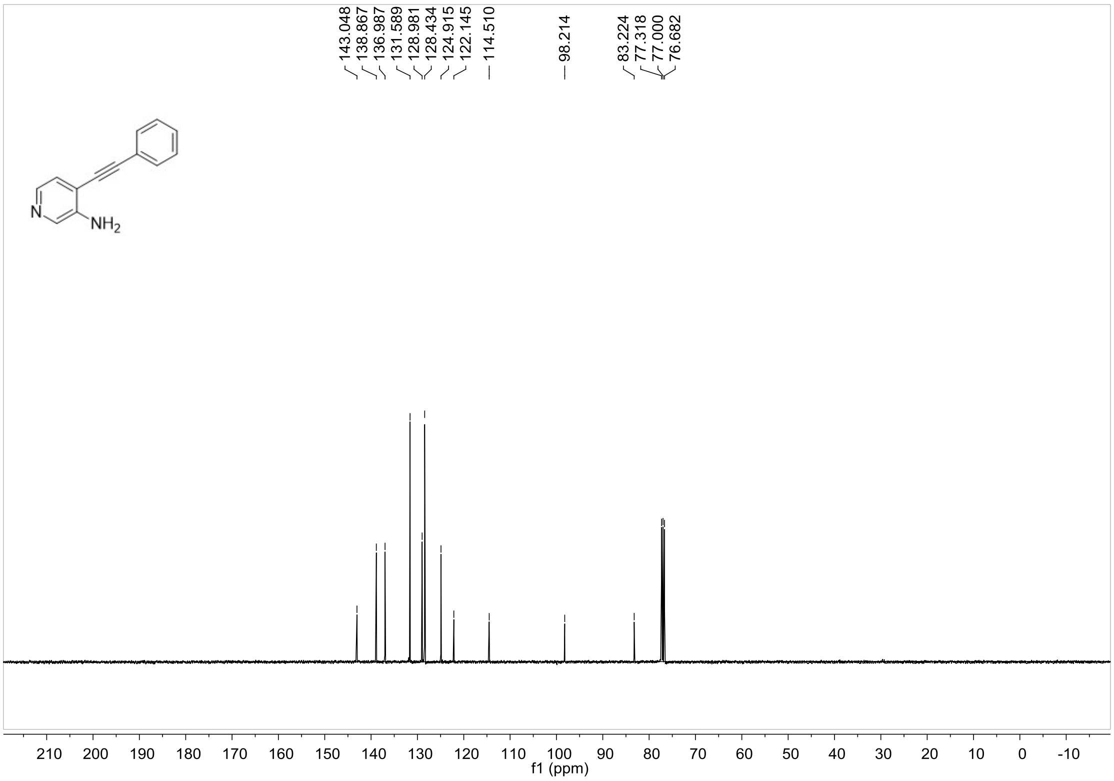

^{1}H NMR Spectrum of **10a**

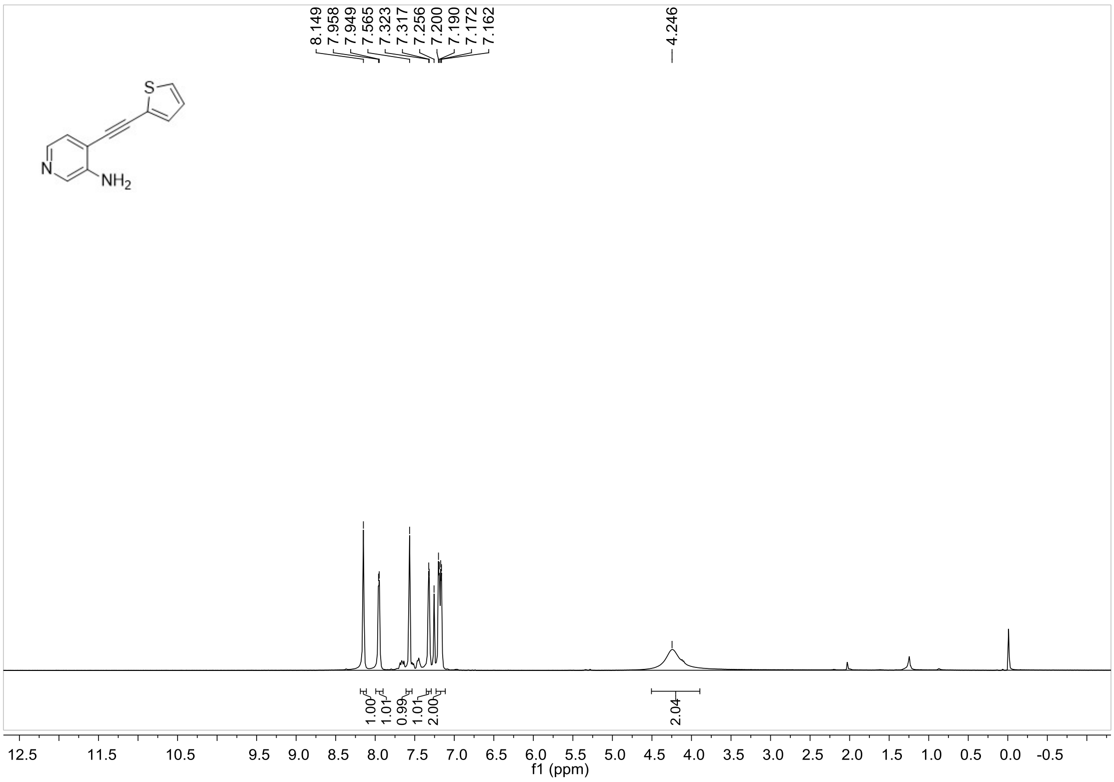

^{13}C NMR Spectrum of **10a**

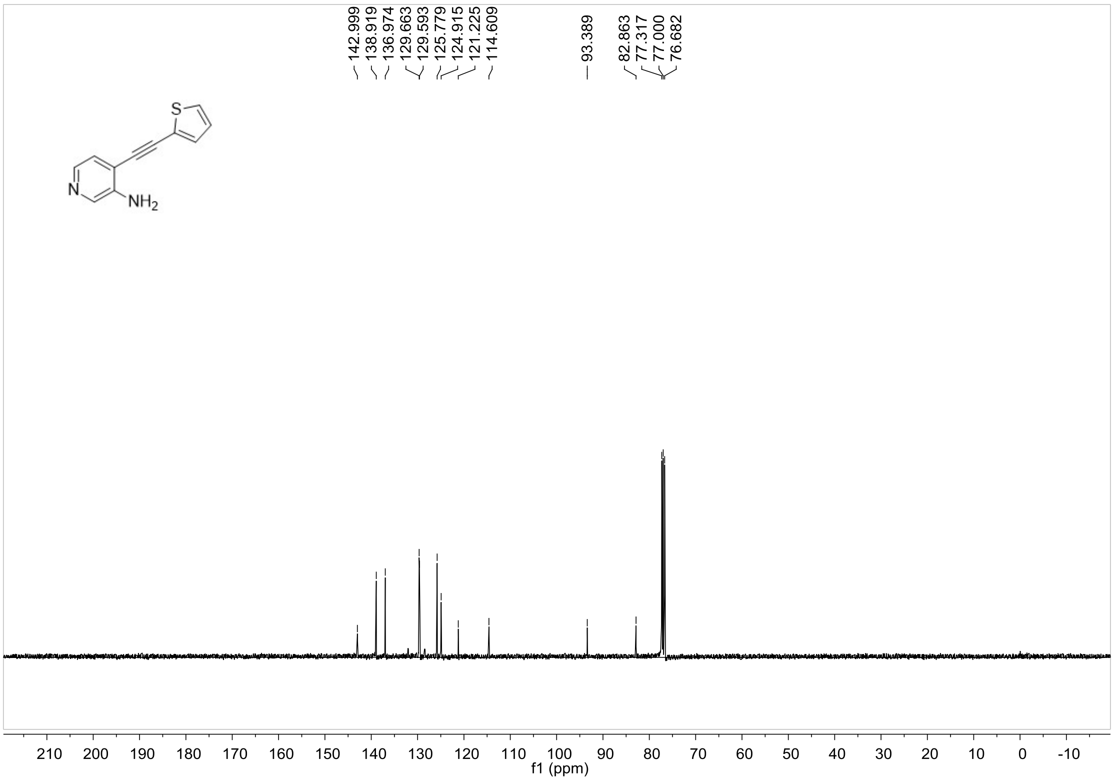

^{1}H NMR Spectrum of **2b**

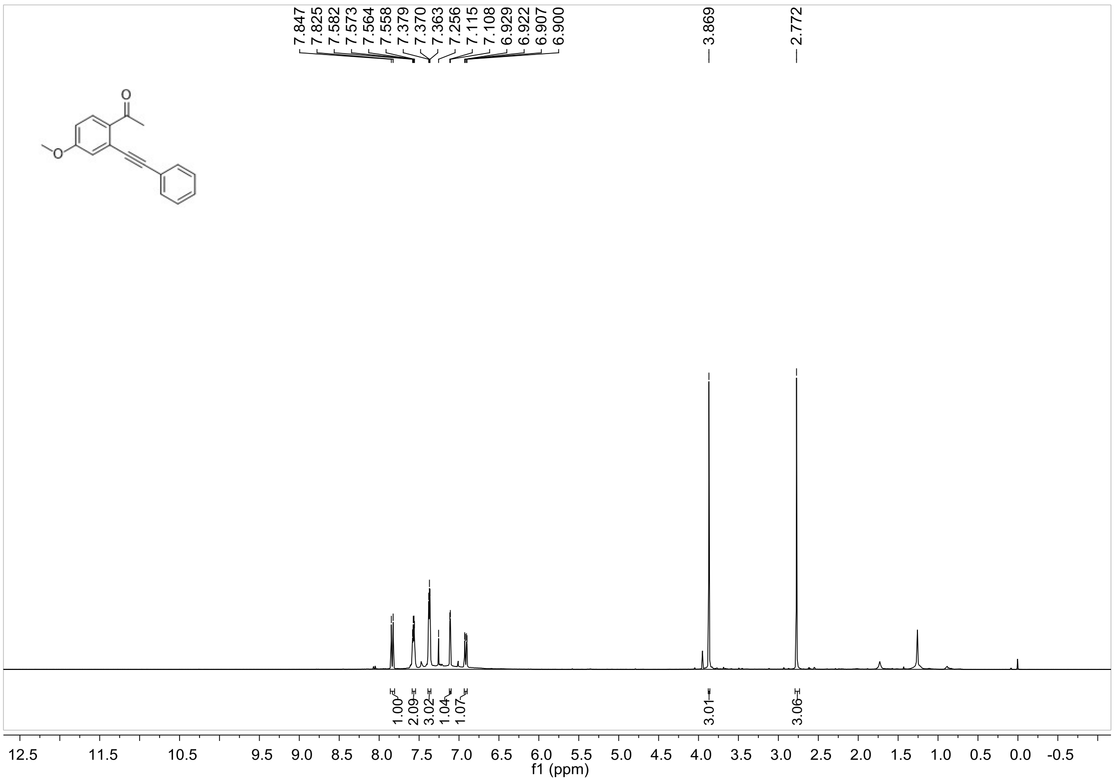

^{1}H NMR Spectrum of **3b**

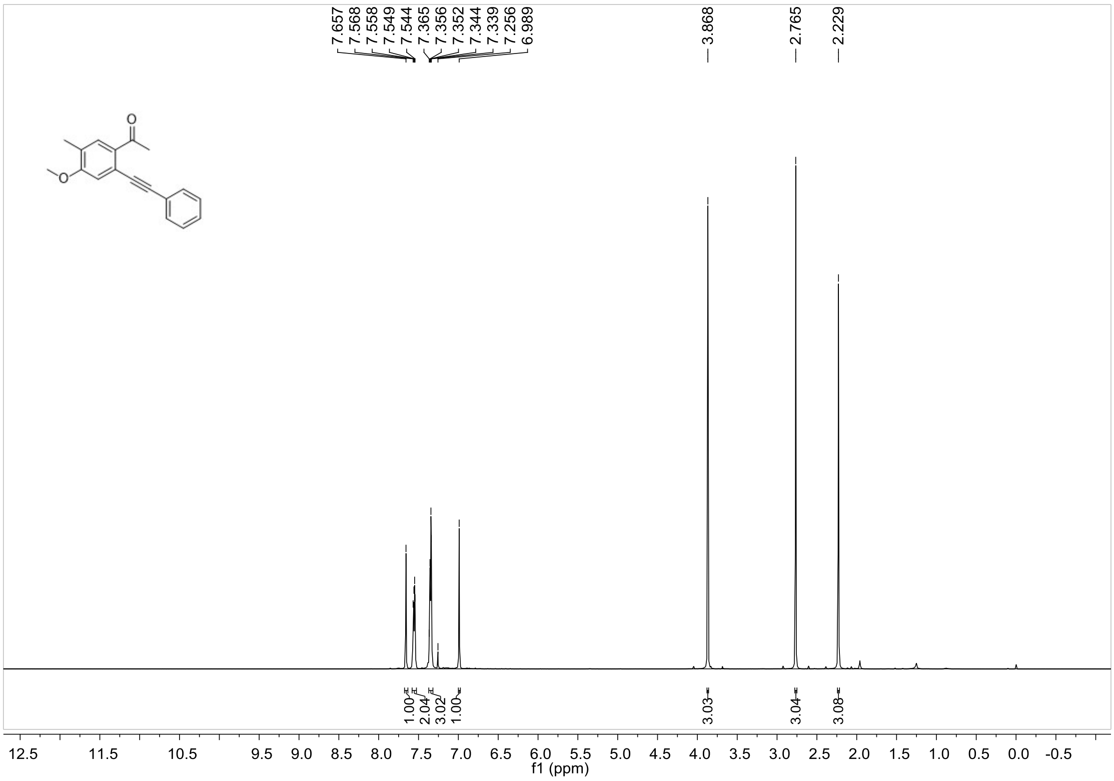

^{13}C NMR Spectrum of 3b

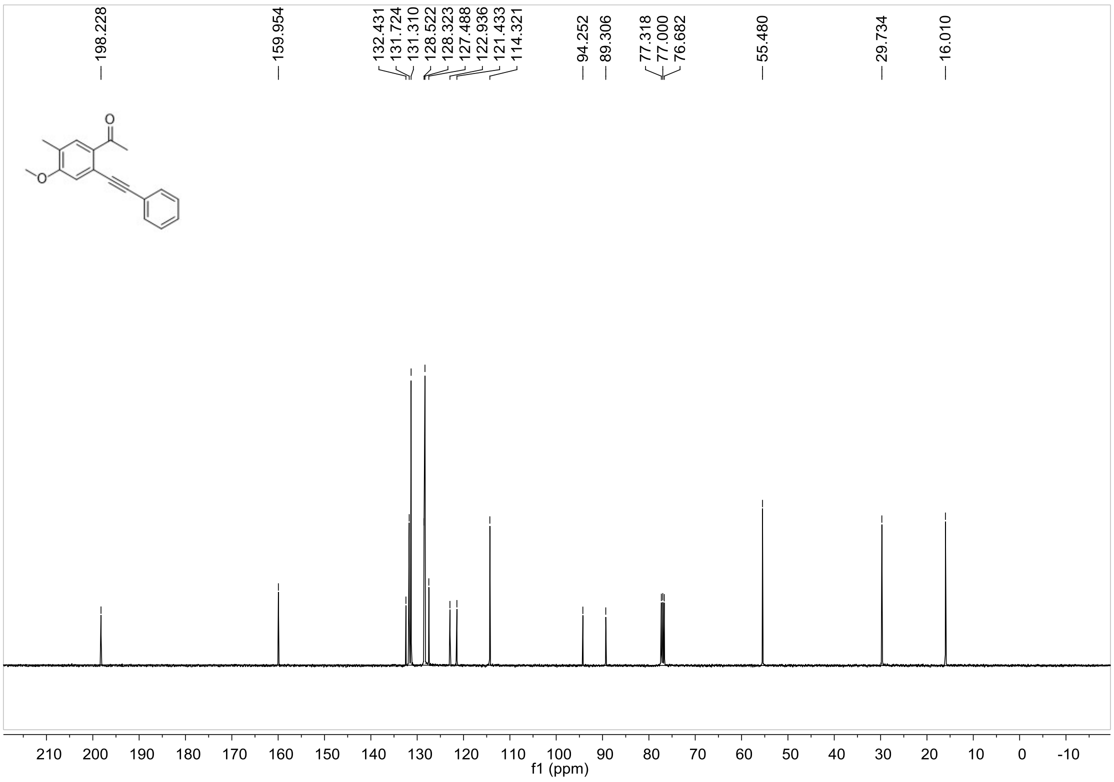

^{1}H NMR Spectrum of **4b**

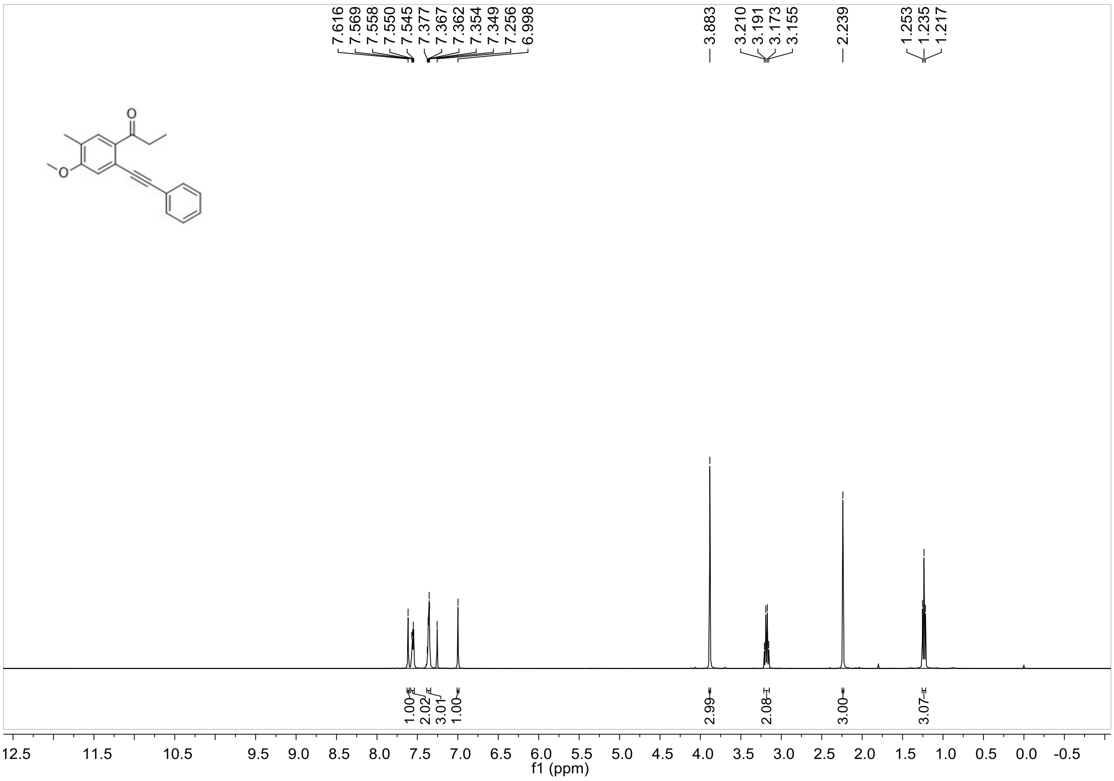

^{13}C NMR Spectrum of **4b**

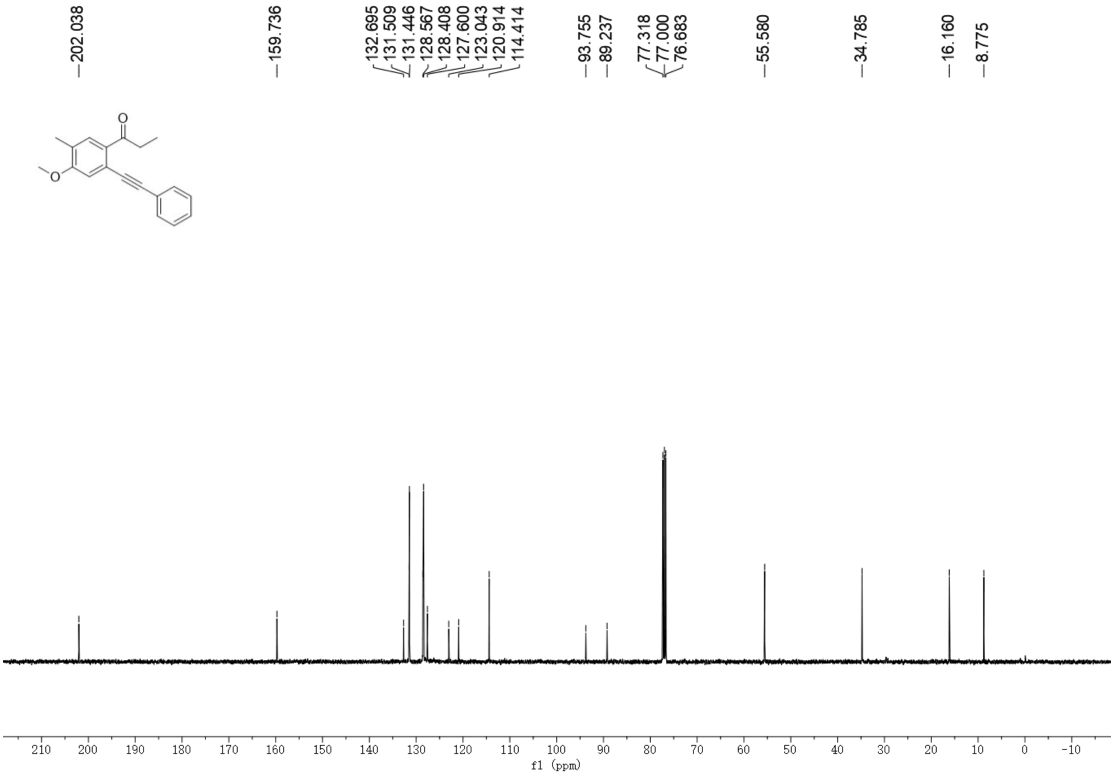

^{1}H NMR Spectrum of **5b**

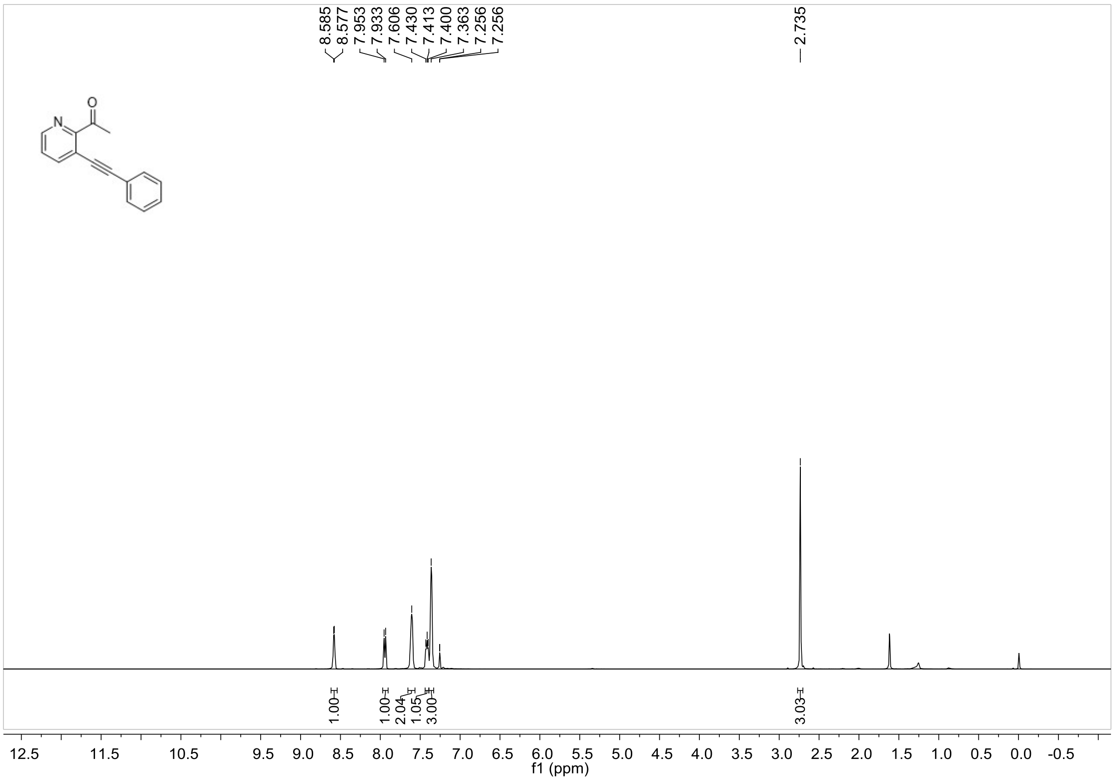

^{13}C NMR Spectrum of **5b**

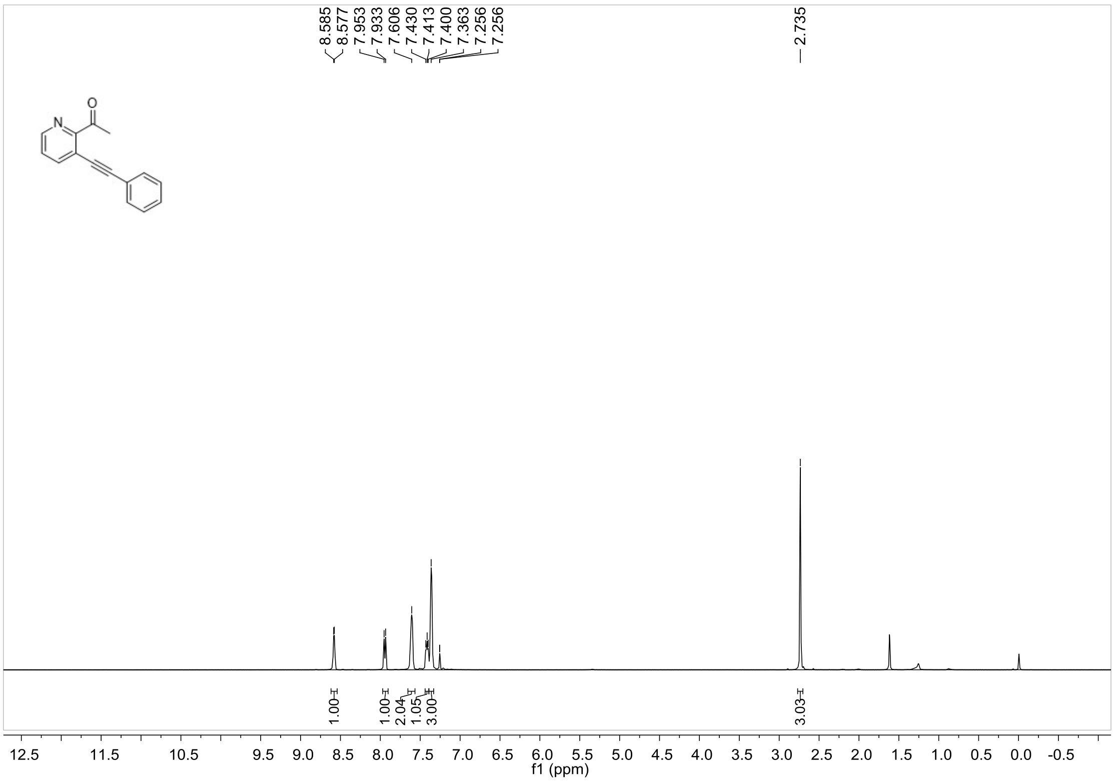

^{1}H NMR Spectrum of **6b**

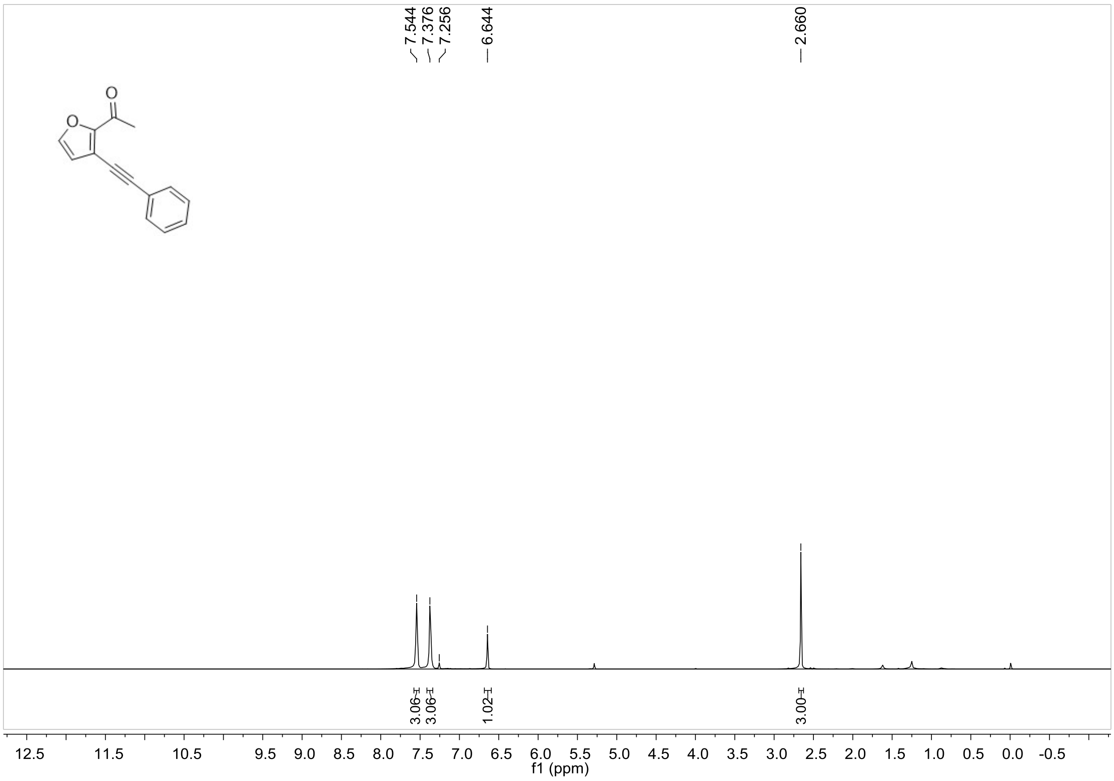

^{13}C NMR Spectrum of **6b**

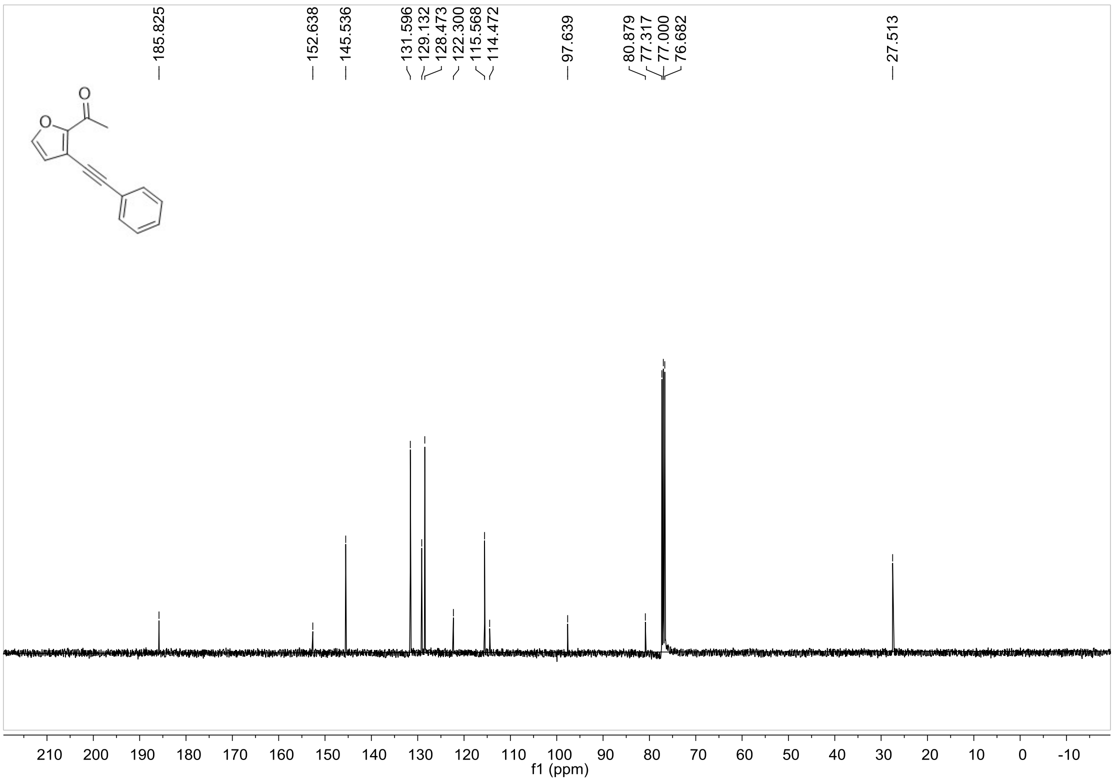

^{1}H NMR Spectrum of **7b**

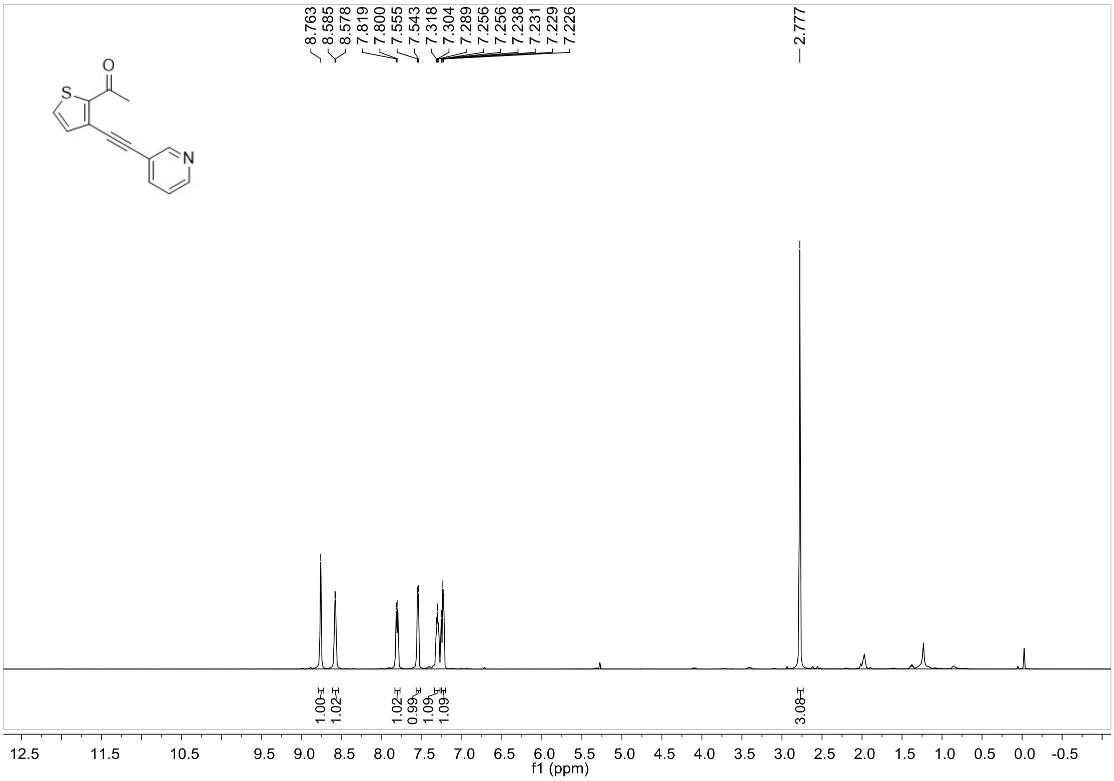

^{13}C NMR Spectrum of **7b**

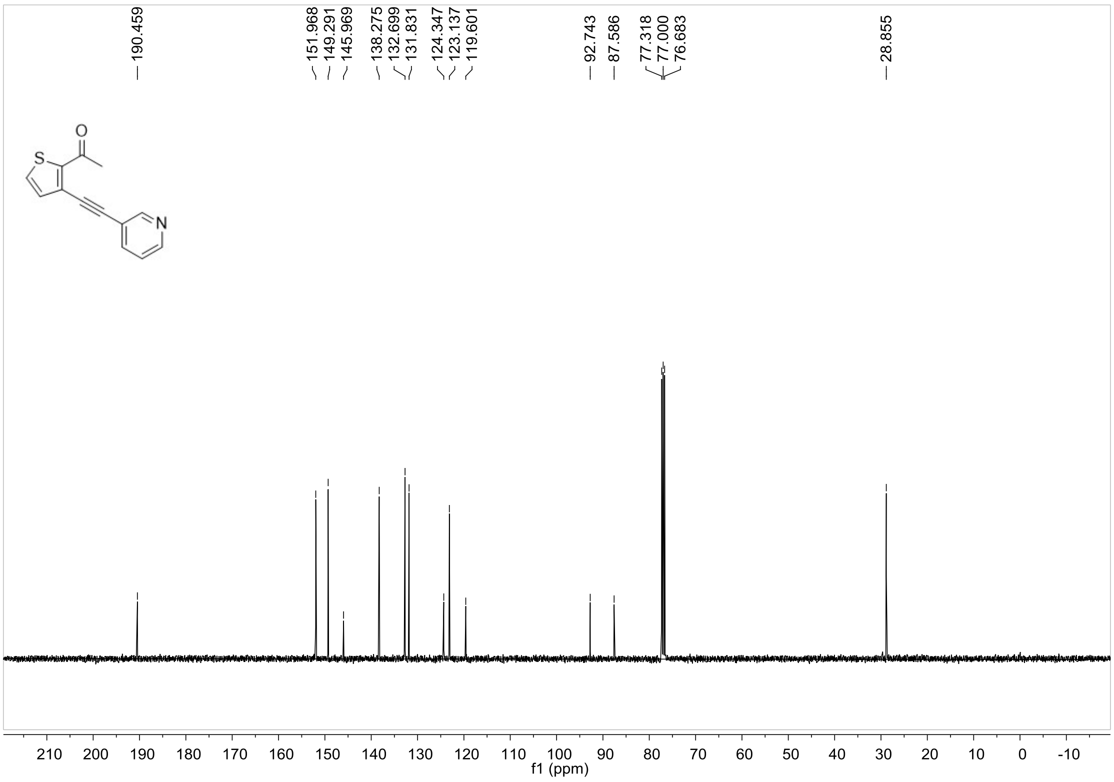

^{1}H NMR Spectrum of **1-ethynyl-4-(phenylethynyl)benzene**

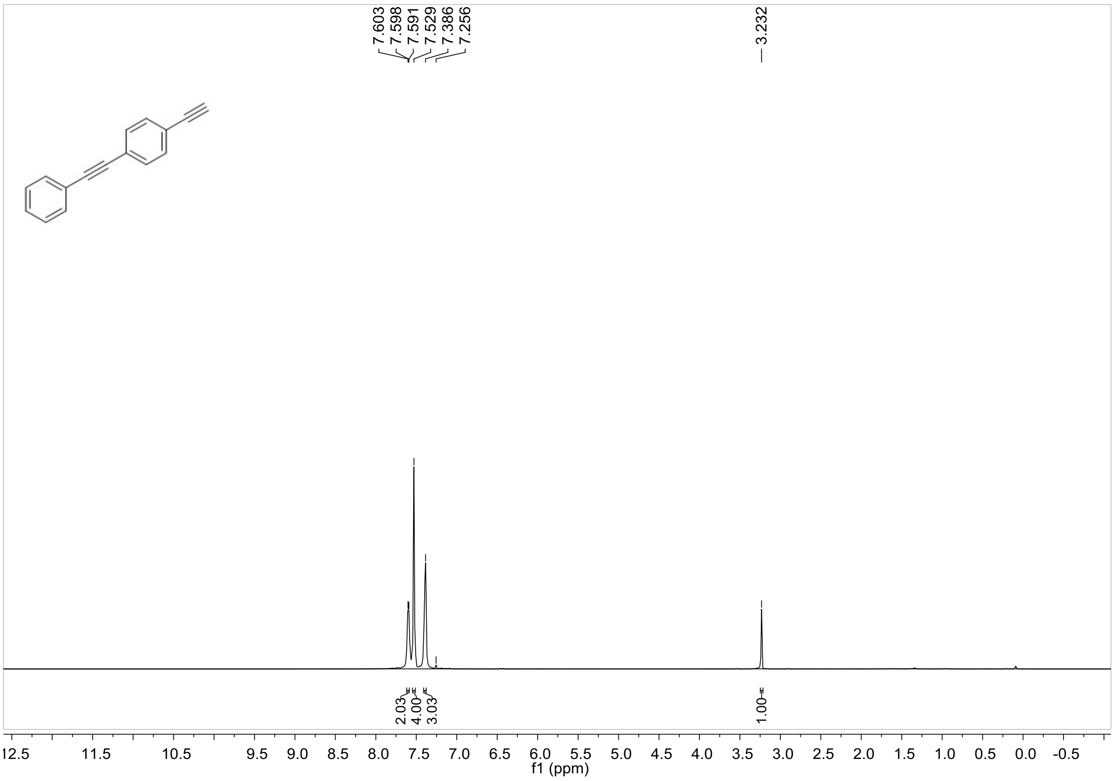

^{13}C NMR Spectrum of **1-ethynyl-4-(phenylethynyl)benzene**

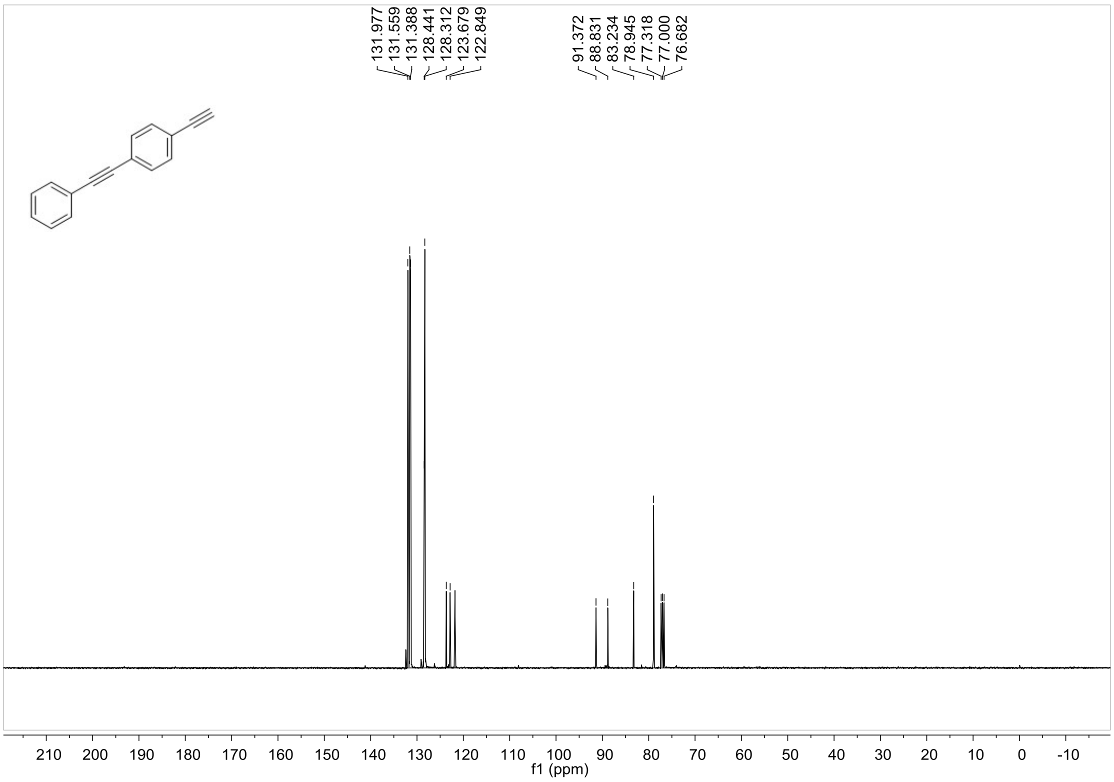

^{1}H NMR Spectrum of ***1b-d₃*** (98% D)

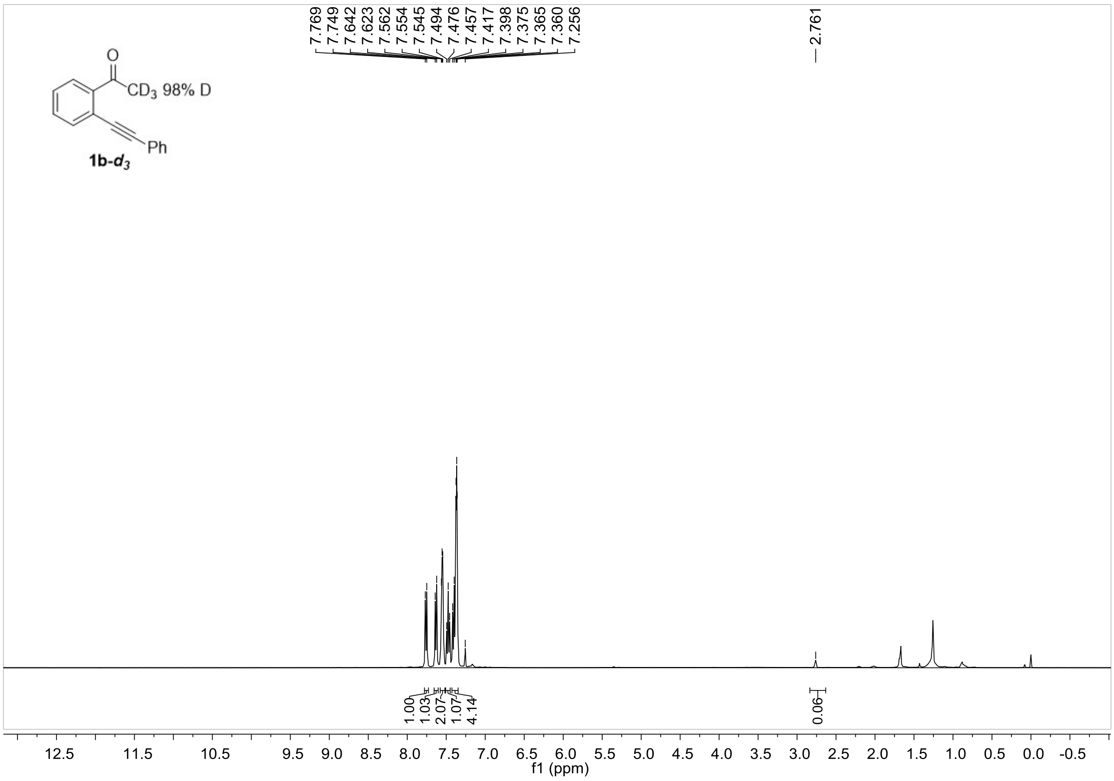

^{1}H NMR Spectrum of ***1b-d₃*** (95% D)

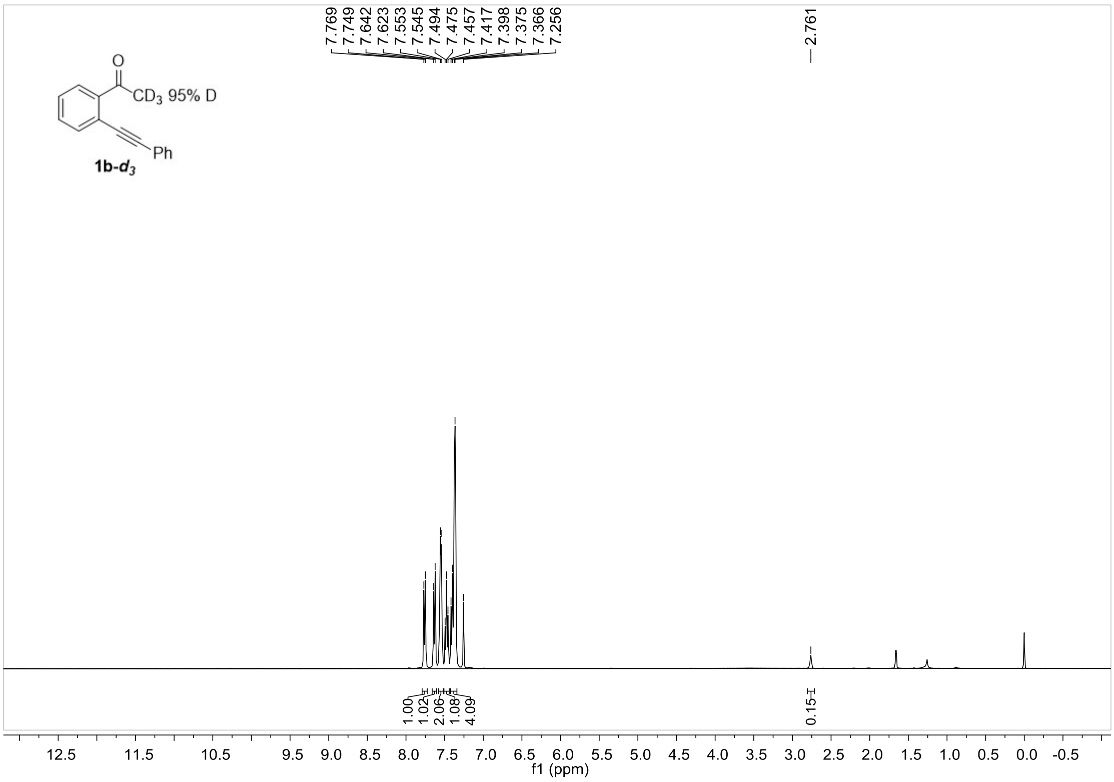

^{1}H NMR Spectrum of ***1b-d$_3$*** (83% D)

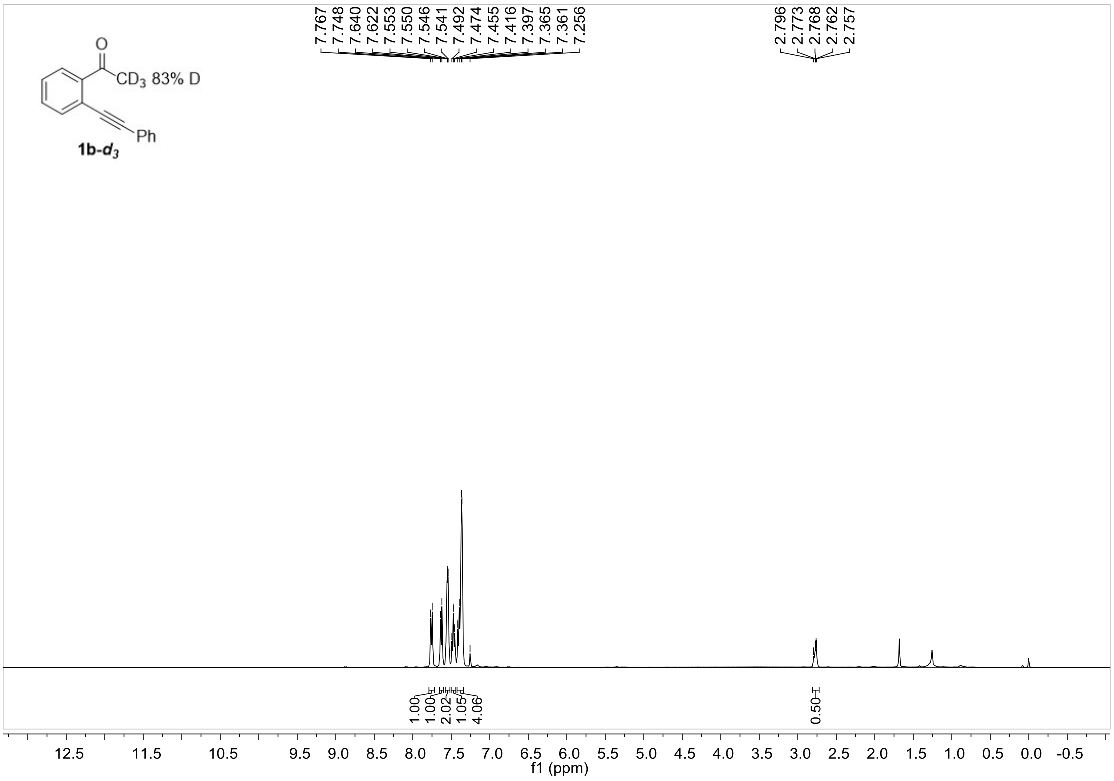

^{1}H NMR Spectrum of ***1b-d$_3$*** (13% D)

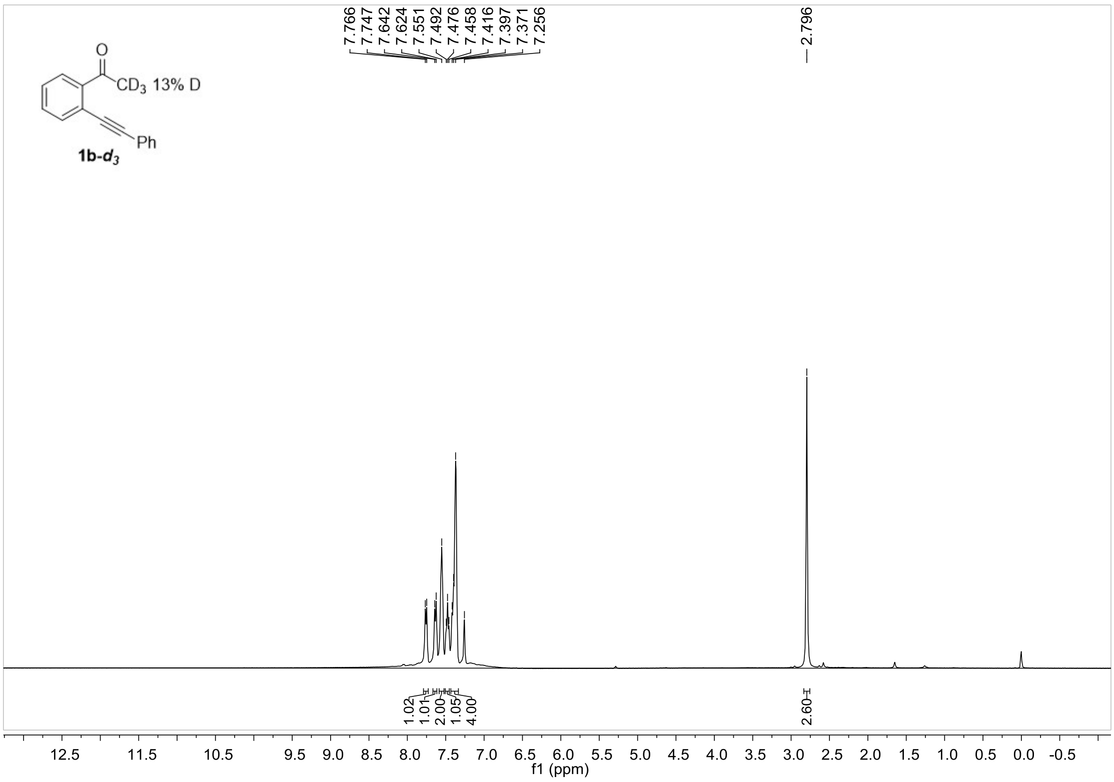

^{1}H NMR Spectrum of ***1b-d*₃** (0% D)

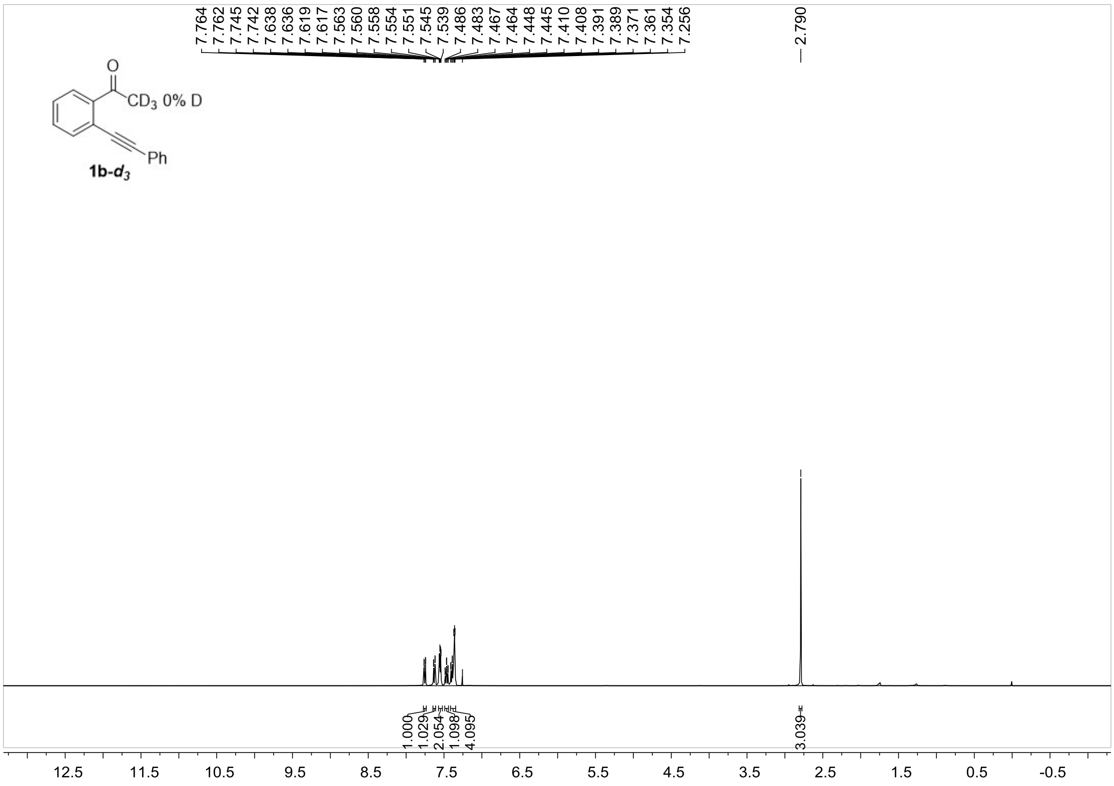

11. References

[1] C. Zhang, M. Liu, M. Ding, H. Xie, F. Zhang, *Org. Lett.* **2017**, *19*, 3418-3421.

[2] a) J. E. Perea-Buceta, T. Wirtanen, O. V. Laukkanen, M. K. Makela, M. Nieger, M. Melchionna, N. Huittinen, J. A. Lopez-Sanchez, J. Helaja, *Angew. Chem., Int. Ed.* **2013**, *52*, 11835-11839; b) C. N. Voth, G. R. Dake, *Eur. J. Org. Chem.* **2020**, *2020*, 744-748; c) P. Rubio-Marques, M. A. Rivero-Crespo, A. Leyva-Perez, A. Corma, *J. Am. Chem. Soc.* **2015**, *137*, 11832-11837; d) B. Li, Z. Ju, M. Zhou, K. Su, D. Yuan, *Angew. Chem., Int. Ed.* **2019**, *58*, 7687-7691; e) W. C. Yang, X. B. Chen, K. L. Song, B. Wu, W. E. Gan, Z. J. Zheng, J. Cao, L. W. Xu, *Org. Lett.* **2021**, *23*, 1309-1314; f) S. Yuan, D. Q. Zhang, J. Y. Zhang, B. Yu, H. M. Liu, *Org. Lett.* **2020**, *22*, 814-817.

[3] R. Jia, B. Li, X. Zhang, X. Fan, *Org. Lett.* **2020**, *22*, 6810-6815.

[4] K. Sakthivel, K. Srinivasan, *Eur. J. Org. Chem.* **2011**, *2011*, 2781-2784.

[5] S. T. Yuan, H. Zhou, L. Zhang, J. B. Liu, G. Qiu, *Org. Biomol. Chem.* **2017**, *15*, 4867-4874.

[6] a) N. Chernyak, S. I. Gorelsky, V. Gevorgyan, *Angew. Chem., Int. Ed.* **2011**, *50*, 2342-2345; b) L. Song, G. Tian, E. V. Van der Eycken, *Chem.--Eur. J.* **2019**, *25*, 7645-7648.

[7] M. E. Domaradzki, Y. Long, Z. She, X. Liu, G. Zhang, Y. Chen, *J. Org. Chem.* **2015**, *80*, 11360-11368.

[8] S. Barbosa, M. Ottone, M. de Almeida, G. Lage, M. Almeida, D. Nelson, W. dos Santos, G. Clososki, N. Lopes, S. Klein, L. Zanatta, *J. Braz. Chem. Soc.* **2018**.

[9] a) T. Miao, Z. Y. Tian, Y. M. He, F. Chen, Y. Chen, Z. X. Yu, Q. H. Fan, *Angew. Chem., Int. Ed.* **2017**, *56*, 4135-4139; b) M. Terada, F. Li, Y. Toda, *Angew. Chem., Int. Ed.* **2014**, *53*, 235-239.

[10] B. Guo, L. Zheng, L. Yang, R. Hua, *J. Org. Chem.* **2014**, *79*, 4352-4357.

[11] M. Zhang, W. Ruan, H. J. Zhang, W. Li, T. B. Wen. *J. Org. Chem.* **2016.** *81*, 1696−1703.

[12] M. Chen, N. Sun, H. Chen, Y. Liu. *Chem. Commun*. **2016**, *52*, 6324–6327.

[13] J. Garcia, J. Sorrentino, E. J. Diller, D. Chapman, T. R. Woydziak. *Synthetic Commun*, **2016**, *46*, 475–481.

[14] L. Z. Yu, Y. Wei, M. Shi. *Chem. Commun*. **2017**, *53*, 8980–8983.

[15] H. Tsukamoto, Y. Kondo. *Org. Lett*. **2007**, *9*, 4227-4230.

[16] H. P. Lin, N. Ibrahim, O. Provot, M. Alami, A. Hamze. *RSC Adv*. **2018**, *8*, 11536-11542.
